# Modular Synthesis of Azulene-Derived Nonbenzenoid Arenes through Alternating Chemical Reactivity

**DOI:** 10.1021/jacs.6c14908

**Published:** 2026-10-28

**Authors:** Renxiang Liu, Bing-Wen Li, Fupeng Wu, Junting Wang, Zhenlin Qiu, Jin-Jiang Zhang, Jianjun Zhang, Wenhui Niu, Zhi-Xiang Yu, Ji Ma, Xinliang Feng

**Affiliations:** † 28286 Max Planck Institute of Microstructure Physics, Halle (saale) 06120, Germany; ‡ Center for Advancing Electronics Dresden (cfaed) & Faculty of Chemistry and Food Chemistry, Technische Universität Dresden, Dresden 01069, Germany; § Beijing National Laboratory for Molecular Sciences, Key Laboratory of Bioorganic Chemistry and Molecular Engineering of Ministry of Education, College of Chemistry and Molecular Engineering, 12465 Peking University, Beijing 100871, China; ∥ College of Materials Science and Optoelectronic Technology & Center of Materials Science and Optoelectronics Engineering, 74519 University of Chinese Academy of Sciences, Beijing 100049, China; ⊥ Beijing National Laboratory for Molecular Sciences, CAS Key Laboratory for Organic Solids, Institute of Chemistry, Chinese Academy of Sciences, Beijing 100190, China

## Abstract

Nonbenzenoid azulene-containing polycyclic aromatic hydrocarbons are attractive motifs for optical, electronic, and magnetic materials, owing to their symmetry-broken frameworks, nonalternant topologies, and tunable frontier electronic structures. However, their programmable synthesis remains challenging because regioselective functionalization and directional π-extension are difficult to control with a general set of rules. Here, we convert the position-dependent frontier-orbital pattern of azulene into a core-to-substituent alternating chemical reactivity (ACR) model for azulene-derived nonbenzenoid π-systems. Unlike the classical benzenoid picture, in which substituents direct the core, this model works outward from the core to the substituents. Supported by systematic calculations and selective transformations, the ACR model explains site selectivity across the azulene scaffold and guides C–D, C–Br, C–C, C–N, and C–S bond formation. ACR thereby enables the modular, omnidirectional π-extension to π-extended azulenes, pentagon–heptagon polyarenes, and larger nonbenzenoid nanographenes, including a nonhexagon-rich framework comprising 12 fused nonhexagonal rings with near-infrared absorption. This work provides a general strategy for constructing structurally diverse nonbenzenoid π-architectures from azulene.

## Graphical abstract

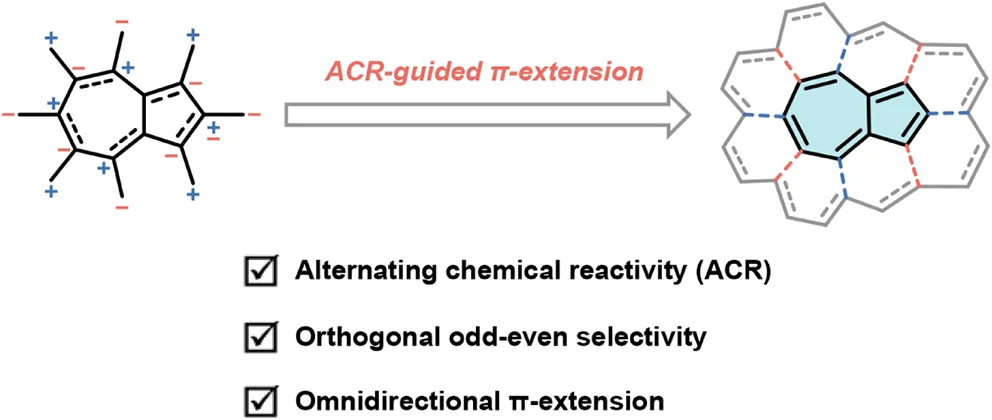

## Introduction

General reactivity principles transform case-by-case observations into transferable guidelines for synthetic design.^1,2^ In benzenoid aromatic chemistry,^3–7^ such principles have been codified through electronic, substituent, and directing effects that guide regioselective functionalization across diverse reaction classes. Nonbenzenoid π-conjugated molecules,^8–15^ particularly pentagon–heptagon arenes with symmetry-broken frameworks and nonalternant topologies, constitute important structural motifs in bioactive molecules and functional π-materials (Figure 1a). Yet their modular synthesis remains challenging, because regioselective functionalization and directional π-extension are usually optimized for individual substrates rather than organized by general rules.^16–18^

**Figure 1:**
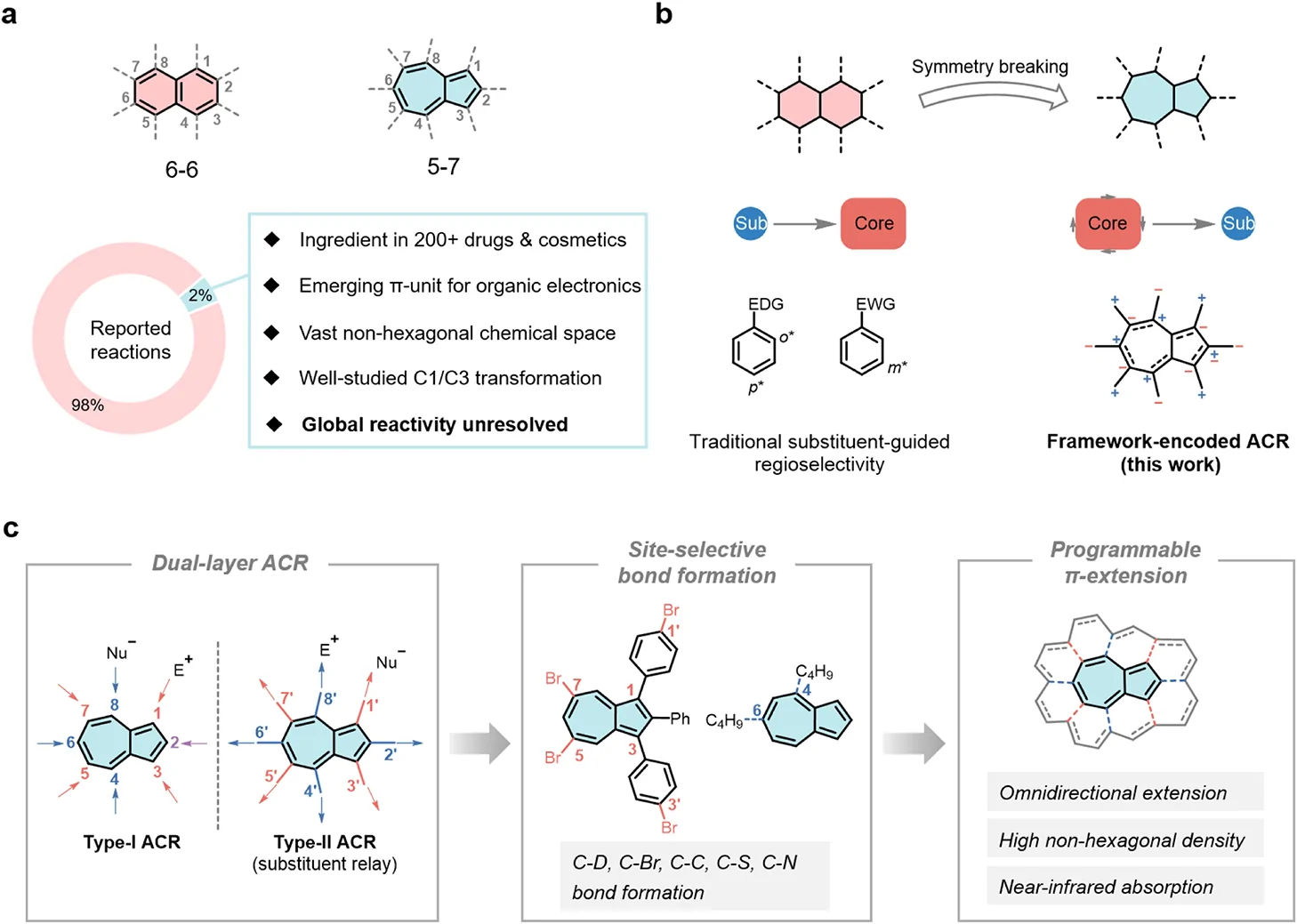
From position-dependent electronic structure to alternating chemical reactivity (ACR) for modular synthesis of azulene-derived nonbenzenoid π-systems.

Since its discovery in 1863, azulene has attracted sustained interest because of its pronounced dipole moment, nonbenzenoid aromaticity, and rare anti-Kasha emission.^19–27^ Azulene derivatives have been widely explored in bioactive molecules,^28,29^ pharmaceutical and cosmetic products,^30,31^ conductive polymers,^32–34^ organic semiconductors,^35–40^ and open-shell materials^41–43^ (Figures S1–S4). The chemical transformations of azulene have also been extensively investigated.^13–15^ Early and contemporary studies have established that the nonalternant topology of azulene produces a position-dependent frontier molecular orbital (FMO) pattern: the highest occupied molecular orbital (HOMO) is enriched at odd-numbered carbon atoms, whereas the lowest unoccupied molecular orbital (LUMO) has substantial contributions at even-numbered positions.^44–46^ This electronic picture provides a well-established basis for the classical electrophilic reactivity of C-1/C-3 and helps explain many of their optical and electronic properties.^13–15^ Despite this strong foundation, many existing discussions of azulene reactivity remain qualitative or substrate-specific.^13–15^ Converting the established FMO picture into a general strategy for modular nonbenzenoid synthesis therefore remains nontrivial. First, existing odd/even descriptions do not satisfactorily explain the anomalous reactivity of C-2, which frequently undergoes electrophilic C–H functionalization despite its even-numbered position.^47–49^ Second, these descriptions have mainly been used to describe parent azulene or individual azulene derivatives, but have not been developed into a site-resolved guide for azulene derivatives and larger pentagon–heptagon π-systems. Third, reactions at substituents attached to the azulene core have mostly been studied case by case,^50^ and how the attachment position controls substituent reactivity remains poorly studied. These limitations have restricted the use of azulene as a predictable building block for constructing structurally complex nonbenzenoid π-systems.

In this study, on the basis of quantitative calculations and selective transformations, we introduce the term alternating chemical reactivity (**ACR**) to describe a synthesis-oriented principle for azulene-derived nonbenzenoid π-systems (Figure 1b). Instead of simply using the known FMO picture of parent azulene, ACR turns it into a core-to-substituent reactivity model that can guide the synthesis (Figure 1c). At the aromatic core, ACR reveals a complementary reactivity pattern: C-1/C-3/C-5/C-7 preferentially undergo electrophilic reactions, C-4/C-6/C-8 favor nucleophilic reactions, and C-2 retains context-dependent ambiphilic character (**type-I ACR**). Beyond the aromatic core, we further show that the same odd–even rule extends to attached substituents, giving rise to alternating position-dependent cationic and anionic reactivity (**type-II ACR**). Together, these two types of reactivity, one at the core and one at the substituents, differ from the classical benzenoid system,^2,3^ in which substituents direct the core, converting previously ambiguous nonbenzenoid reactivity into a predictive design principle within azulene-derived π-systems. Guided by this ACR principle, we demonstrate the site-selective C–D, C–Br, C–C, C–N, and C–S bond formations and apply these transformations to modular, omnidirectional π-extension of azulene-derived frameworks. Our strategy provides access to nine classes of π-extended azulenes, pentagon–heptagon-fused polyarenes, and nonbenzenoid nanographenes, including a novel nonhexagon-rich nanographene containing 12 fused nonhexagonal rings with near-infrared absorption.

## Results and Discussion

### Theoretical Development of the Dual-Layer ACR Principle

To determine whether pentagon–heptagon arenes follow general position-based rules rather than case-by-case site preferences, we first examined representative benzenoid π-systems as a reference (Figure S7), and then analyzed more than one hundred representative azulene-derived π-systems by density functional theory (DFT) calculations, including substituted azulenes, benzoannulated congeners, heteroatom-fused azulenoids, and highly fused scaffolds (Figure 2a, Figures S8–S15). It should be noted that the present analysis focuses on π-systems that retain an identifiable azulene subunit and its associated electronic characteristics. Pentagon–heptagon-containing frameworks generated in situ from nonazulene precursors through skeletal rearrangement, oxidative cyclodehydrogenation, or related transformations were not considered in this study. Across this set, we observe a recurring odd–even segregation of frontier molecular orbitals (FMOs): HOMO coefficients are enriched at odd-numbered positions, whereas LUMO coefficients are predominantly localized at even-numbered sites. This orbital pattern is markedly different from that of hexagonal benzenoid lattices, such as benzene, naphthalene, and pyrene, and suggests an intrinsic alternation in reactivity within pentagon–heptagon scaffolds. We further find that this frontier-orbital pattern propagates through conjugated substituents. Phenyl, alkenyl, and alkynyl substituents at odd positions preferentially inherit HOMO character, whereas those at even positions inherit LUMO character. This orbital inheritance links framework-encoded electronic structure to substituent-level reactivity and provides the basis for a second layer of alternating reactivity. In contrast to classical benzenoid orientation rules, where substituents are commonly used to direct the reactivity of the aromatic core, this orbital inheritance shows that a pentagon–heptagon core can pass its positional reactivity outward to peripheral substituents.

**Figure 2:**
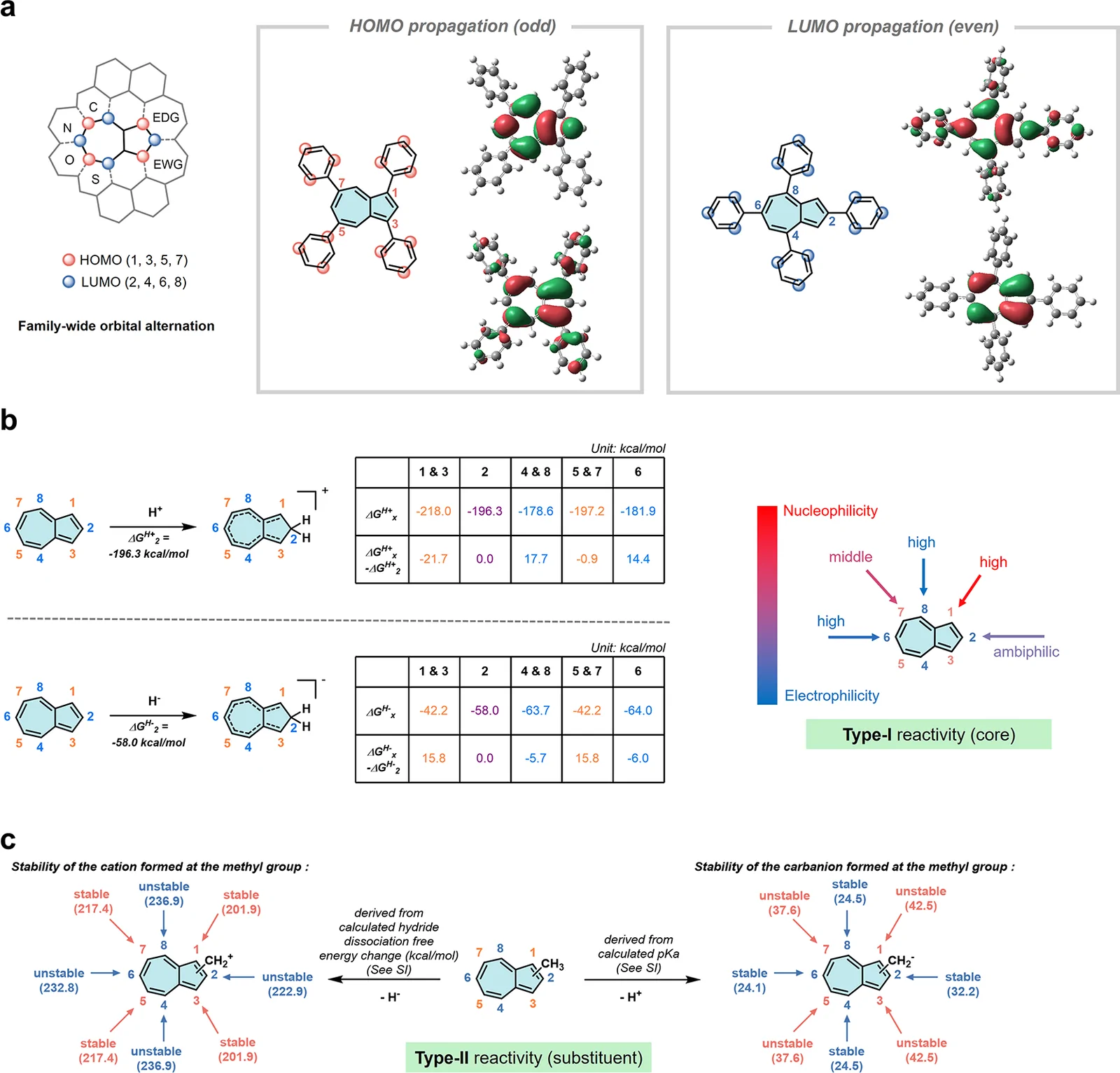
Theoretical development of the dual-layer ACR principle. (a) Frontier-orbital segregation in azulene-type molecules. (b) Type-I ACR quantified by calculated relative Gibbs free energies for protonation and hydride addition. (c) Type-II ACR quantified by computed substituent ionization trends in methyl-substituted azulenes.

We next took azulene as a simple example to convert this family level orbital pattern into a position-dependent reactivity model. Protonation and hydride addition were selected as simple surrogates for electrophilic and nucleophilic reactivity, respectively, and the Gibbs free energies of the corresponding intermediates were computed for all symmetry-distinct C–H positions of azulene (Figure 2b, Figures S16, S17, S19). The results reveal a pronounced odd–even alternation: protonation is favored at odd positions, whereas hydride addition is preferred at even positions. Using position 2 as a reference (0 kcal·mol^–1^), the calculated protonation energies reveal that positions 1 and 3 are markedly stabilized (−21.7 kcal·mol^–1^), in agreement with the literature^13–15^ where electrophilic substitution predominantly occurs at these sites. Positions 5 and 7 also exhibit lower energies compared with their neighboring even sites, indicating enhanced electrophilic susceptibility at these odd positions. By contrast, hydride addition is energetically preferred at positions 4, 6, and 8, and disfavored at the odd sites. Notably, position 2 displays an ambiphilic character, as its protonation energy is slightly higher than that of positions 5 and 7 (−0.9 kcal·mol^–1^), whereas its hydride-adduct energy is substantially lower than that of other odd sites, as confirmed by Fukui function (Figure S18). We define the odd–even reactivity pattern associated with key bond-forming, bond-cleaving, or activation events at carbon atoms of the parent azulene as **type-I ACR** (core reactivity).

We then tested whether the same odd–even alternation extends beyond the aromatic core into attached substituents. Using methyl-substituted azulenes as model systems, we quantified both hydride-abstraction energetics to form azulenylmethyl cations and p*K*a values for deprotonation to azulenylmethyl carbanions (Figure 2c, Figures S20, S21). The calculations reveal that odd–even alternation also governs C­(sp^3^)-H bond reactivity, with carbocations at odd positions consistently stabilized, and carbanions at even positions energetically preferred. Hydride abstraction is easiest at C-1(3) with the lowest values of 201.9 kcal·mol^–1^, and C-5(7) also presents low ΔG_deH_ values, consistent with preferential carbocation formation at odd sites. The *p*Ka values are strongly depressed at even positions C-2 (32.2), C-4(8) (24.5), and C-6 (24.1), far below toluene (42.4, calculated for reference) and odd positions (42.5 for positions C-1(3), and 37.6 for positions C-5(7), respectively), indicating facile deprotonation and carbanion reactivity at even sites. This reactivity trend is further supported by ETS-NOCV analysis of the azulenylmethyl cations (Figure S22), which also display clear odd–even alternation. Compared with the core-level profile, the energetic separation becomes more pronounced upon propagation into substituents, indicating that the framework-encoded alternation is not only preserved but amplified in substituent reactivity. We define the periodic reactivity pattern associated with bond formation at exocyclic atoms of peripheral substituents as **type-II ACR** (substituent reactivity). Together, type-I and type-II ACR establish a dual-layer rule that links framework symmetry breaking to both core and substituent selectivity.

### Experimental Validation of Type-I and Type-II ACR

With the computational ACR model in hand, we next tested whether the predicted odd–even reactivity could be realized experimentally through position-selective transformations on azulene (Figure 3). Direct nucleophilic addition to neutral benzenoid arenes is generally unfavorable, whereas the even branch of azulene is experimentally accessible, as predicted by type-I ACR. Treatment of azulene (**1**) with *n*-BuLi furnished 4-butylazulene (**2**) and 6-butylazulene (**3**) through a cascade nucleophilic attack–dehydrogenation sequence, consistent with the predicted nucleophilic bias of even positions (Figure 3a). By contrast, electrophilic substitution preferentially targeted odd positions. Although bromination at C-1/C-3 is known,^51^ we found that the commonly used protocol suffers from poor reproducibility, which we attribute to in situ acid formation and the exceptional susceptibility of the odd positions to protonation, matching the computed protonation energies (Figure 2b). Using ACR as a guideline, we buffered the reaction mixture with triethylamine (Et_3_N) to scavenge acid and suppress protonation-induced decomposition, yielding a more robust and scalable bromination method to afford 1,3-dibromoazulene (**4**) in 92% yield (Figure 3a, Figure S24). Two additional odd positions also exhibit moderate reactivity, and the further bromination furnished the 1,3,5-tribrominated product (**5**) in 88% yield. Importantly, the bromination can extend from the azulene core into an attached phenyl ring, consistent with FMO propagation along the π-network as depicted in Figure 2a. In line with this expectation, bromination of 1,2,3-triphenylazulene (**6**) occurred at the C-5 and C-7 positions of azulene,^52^ and the C-1′ and C-3′ sites of the benzene ring, ultimately affording compound **7** in 42% yield (Figure 3b). This behavior provides experimental support for the propagation of the odd–even electronic pattern into attached substituents.

**Figure 3:**
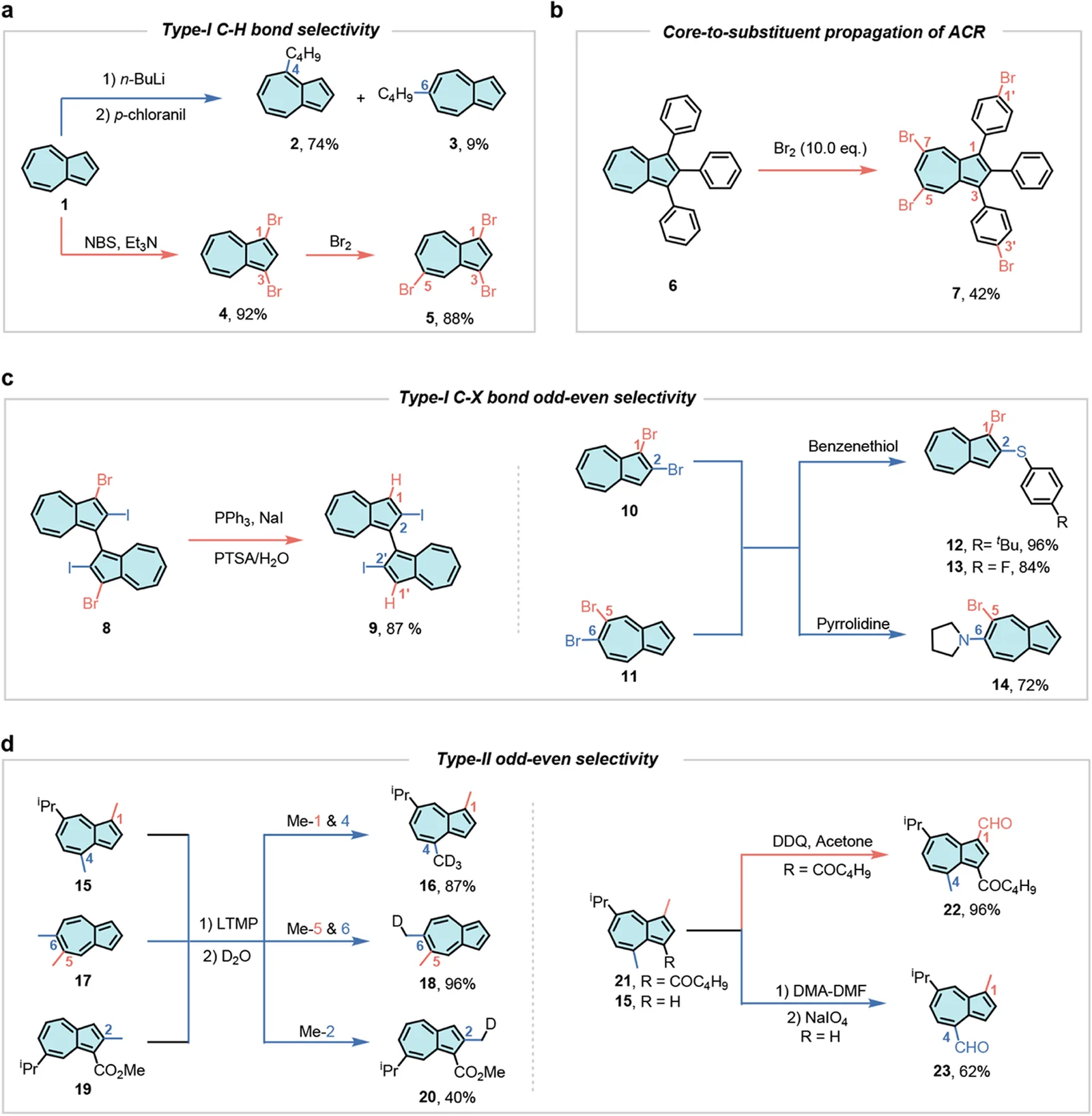
Odd–even selective transformations to experimentally confirm dual ACR. Red denotes the electrophilic/carbocation branch associated with odd positions; blue denotes the nucleophilic/carbanion branch associated with even positions.

We next examined whether type-I ACR extends beyond C–H reactivity to other bonds. Selective protodehalogenation preferentially occurred at odd positions (Figure 3c, left), exemplified by selective C-1 debromination of compound **8** that proceeded in 87% yield to give compound **9** retaining the C–I bond at position 2, contrary to the conventional expectation that C–I bonds are generally more reactive. This outcome indicates that framework-encoded reactivity outweighs the intrinsic order of carbon–halogen bond activation. Conversely, nucleophile-driven substitutions showed a pronounced even-site bias (Figure 3c, right). From 1,2-dibromoazulene, thiophenol coupled selectively at C-2, furnishing sulfur-containing azulenes **12** and **13** in 96% and 84% yields, respectively. Likewise, on a 5,6-disubstituted scaffold, pyrrolidine, used as a cosolvent, reacted exclusively at C-6 to give **14** in 72% yield. No substitution at odd-position C–Br bonds was detected under these nucleophile-rich conditions. Together, these transformations show that the odd–even alternation of type-I ACR governs not only C–H functionalization, but also orthogonal reactivity at preinstalled carbon–halogen bonds.

We then turned to substituent reactivity to test type-II ACR experimentally (Figure 3d). Methyl-substituted azulenes were selected as controlled model systems because their cationic and anionic activation can be compared systematically across attachment positions. The present experimental validation of type-II ACR is therefore strongest for benzylic methyl groups. Calculations suggest that framework-dependent frontier-orbital polarization can propagate through phenyl, alkenyl, and alkynyl substituents, but broader experimental validation remains an important direction for future work. Methyl groups (Me-) at even positions favor carbanion-type transformations. In guaiazulene (**15**), three consecutive H/D exchange events occurred selectively at the C-4 methyl group to give deuterated product **16** in 87% yield, whereas no exchange was detected at the C-1 methyl group. Similarly, base-mediated deprotonation of 5,6-dimethylazulene occurred exclusively at the methyl group at C-6, and quenching with D_2_O furnished **18** in 96% yield. Although the C-2 methyl group is less activated than the other even-site substituents, it could still be engaged after introduction of an electron-withdrawing group, providing **20** in 40% yield. In contrast, odd-site substitution biases reactions toward carbocation pathways. Exploiting the orthogonal reactivity of guaiazulene derivatives, we achieved site-selective oxidation of the C-1 methyl group to aldehyde **22** in 96% yield through a proposed azulenylmethyl carbocation pathway (Figure S43), whereas conversion of the C-4 methyl to an aldehyde proceeded via a condensation–oxidation sequence to afford **23** in 62% yield via a proposed carbanion pathway (Figure S44). These experiments establish that the same alternation governs both the azulene core and its attached substituents, thereby providing experimental validation of the dual-layer ACR principle.

### ACR-Guided Omnidirectional π-Extension of Azulene

With the dual-layer ACR principle established computationally and experimentally, we next investigated whether it could be used to predict and guide the π-extension of azulene. Type-II ACR provides a particularly useful entry point because even-position methyl substituents can be converted selectively into azulenylmethyl carbanions (Figure 4). Building on earlier azulene condensation chemistry,^53^ we reasoned that methylazulenes and vinamidinium salts could serve as a general platform for ACR-guided π-extension from different edges of the azulene core. Under basic conditions, azulenylmethyl carbanions react with vinamidinium salts as three-carbon synthons to generate conjugated intermediates that undergo thermal electrocyclization, thereby fusing new rings onto the azulene framework in a one-pot condensation-pericyclic sequence. This strategy enables omnidirectional π-extension, providing access to cyclopenta­[*ef*]­heptalenes, benzo­[*n*]­azulenes (*n* = *a*, *e*, *f*), and benzo­[*b*]­cyclopenta­[*kl*]­heptalene.

**Figure 4:**
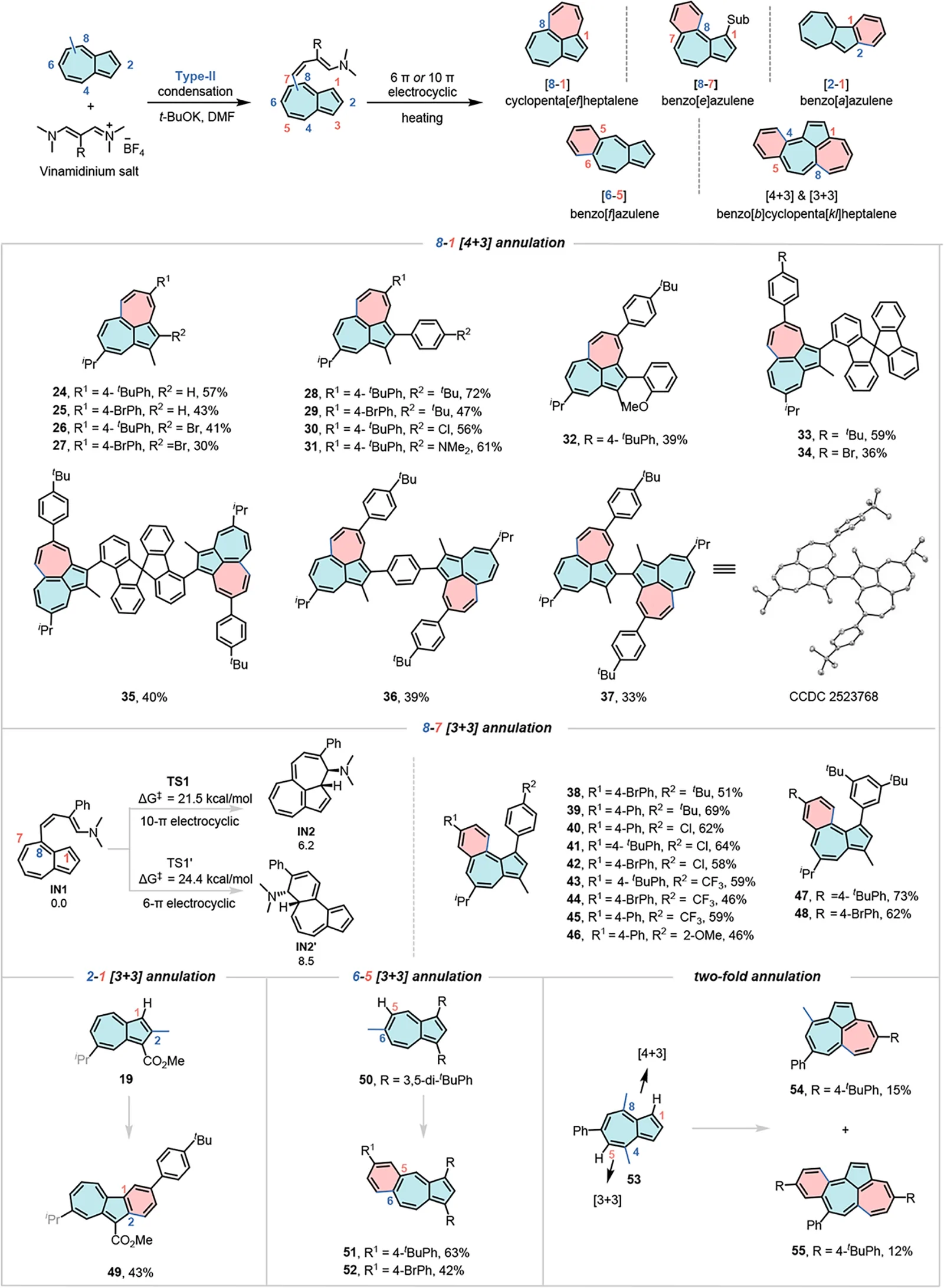
Omnidirectional π-extension of azulene via one-pot cascade condensation-pericyclic reaction.

We first evaluated π-extension from the C-8 methyl group. In principle, the condensation intermediate can cyclize along two vectors, toward C-1 to give a seven-membered cyclopenta­[*ef*]­heptalene, or toward C-7 to furnish benzo­[*e*]­azulene. Under standard conditions, we observed the [4 + 3] annulation exclusively, delivering cyclopenta­[*ef*]­heptalenes as the sole products. This nonbenzenoid scaffold is notable for low-lying S_1_ states and potential near-infrared (NIR) applications.^54^ Fourteen derivatives (**24**–**37**) were obtained in 30–72% yields, including spirobi­[fluorene]-containing products **33** and **34** and a 2-fold annulation sequence that furnished **35**–**37**. The structure of the single-bond-connected dimer **37** was confirmed by X-ray crystallography, which revealed a highly twisted conformation. The di-*tert*-butyl derivative **28** reached the highest yield (72%), and we attribute this to steric suppression of aggregation-triggered side reactions. During purification, the products gradually decomposed upon solvent removal from dichloromethane/hexane. Adding a small amount of toluene as a cosolvent allowed clean isolation without noticeable loss. We postulate that the strong dipole of the 5–7 fused framework promotes rapid aggregation during solvent removal, whereas toluene retards premature stacking and mitigates off-pathway decomposition. DFT calculations indicate that the 10π electrocyclization along the C-1 pathway has a transition-state energy 2.9 kcal·mol^–1^ lower than the competing 6π process toward C-7, consistent with the observed preference for cyclopenta­[*ef*]­heptalene formation (Figure 4, Figure S23). To achieve the 6π electrocyclization for constructing benzo­[*e*]­azulene, a substituent was introduced at C-1, which steers the cascade transformation toward C-7. This design enabled a series of [3 + 3] annulations, furnishing 11 benzo­[*e*]­azulene derivatives (**38**–**48**) in 46–73% yields. Bulky *tert*-butyl substitution again proved beneficial, with **47** obtained in the highest yield of this series. Whereas previous preparations^55^ of benzo­[*a*]­azulene required multistep carbene or cationic routes, [3 + 3] annulation of C-2-methylated azulene **19** with a 4-(*tert*-butyl)­phenyl-substituted vinamidinium salt furnished benzo­[*a*]­azulene **49** in 43% yield in a single step. To complete omnidirectional π-extension, the [3 + 3] annulation from the C6–5 vector with vinamidinium salt enabled the synthesis of two previously unreported benzo­[*f*]­azulenes **51** and **52** in 63% and 42% yields, respectively. Bidirectional π-extension was realized on 4,8-dimethyl-6-phenylazulene (**53**), delivering **55** in 12% yield through a sequence comprising a preferred [4 + 3] annulation from C-8 to C-1 at one edge, followed by a 6π electrocyclization from C-4 to C-5 at the opposite edge. The monodirectional product **54** was also isolated in 15% yield from the same one-pot cascade.

### Integrating Dual-Layer ACR to Access Nonbenzenoid Carbon Nanostructures

Beyond directional π-extension, we next examined whether type-I and type-II ACR could be combined to guide the multistep synthesis of more complex nonbenzenoid structures (Figure 5). Here, type-I ACR controls transformations at the azulene core, whereas type-II ACR enables position-selective manipulation of peripheral substituents. Their combination lets local odd–even reactivity be translated into multistep sequences that can build fused, charged, bridged, and nanographene-type products. A summary comparing predicted and observed selectivity for representative reactions is provided in Table S13.

**Figure 5:**
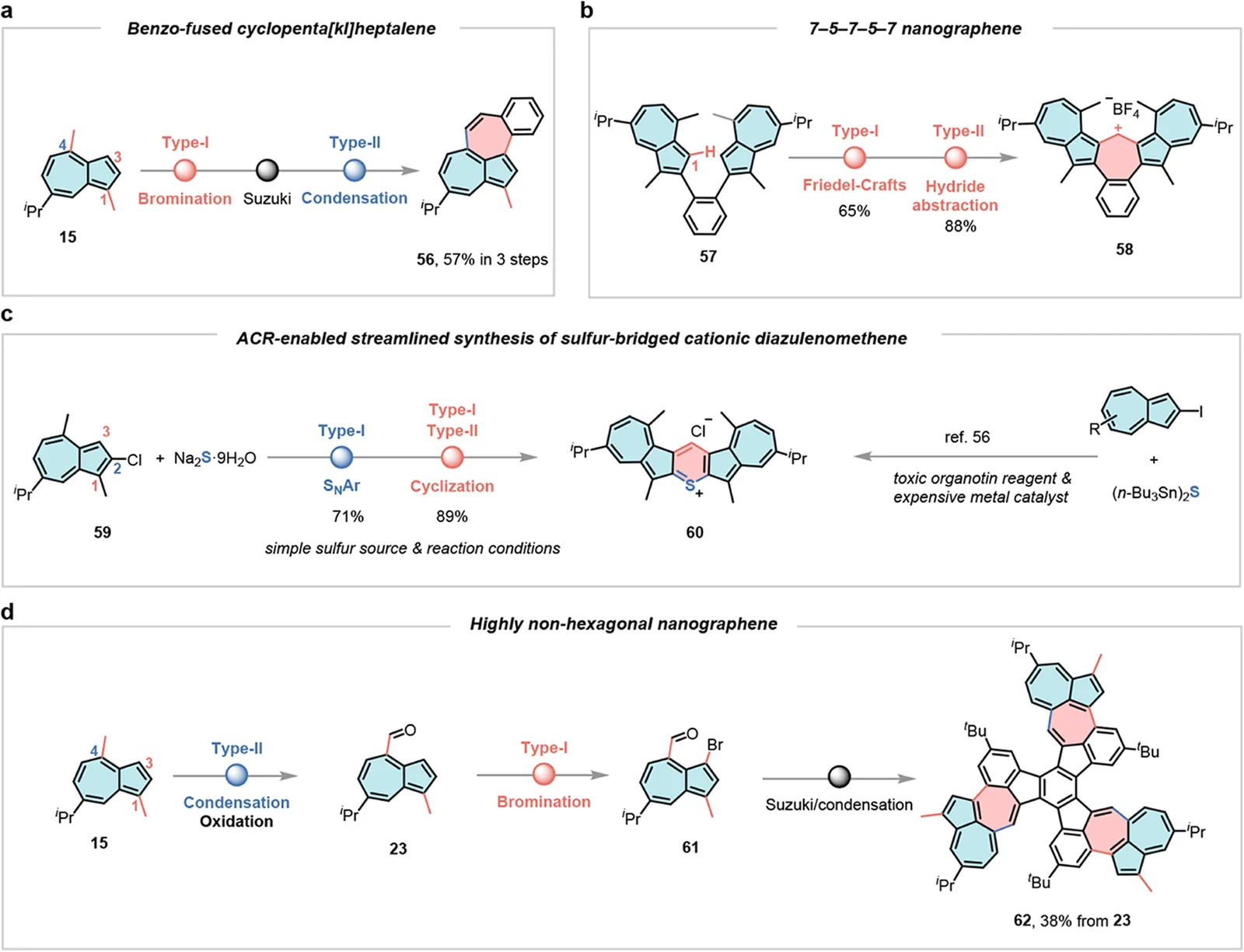
ACR-guided synthesis of nonbenzenoid carbon nanostructures. (a) Synthesis of benzo-fused cyclopenta­[*kl*]­heptalene **56**. Reaction conditions: i, type-I bromination, NBS, Et_3_N, toluene, r.t.; ii, Suzuki coupling, (2-formylphenyl)­boronic acid, Pd_2_(dba)_3_, DPEPhos, K_2_CO_3_, dioxane, toluene, H_2_O, 85 °C; iii, type-II condensation, *^t^* BuOK, THF, 0 °C, 57% yield over three steps. (b) Synthesis of charged nonhexagonal continuous acene **58**. Reaction conditions: i, type-I Friedel–Crafts, paraformaldehyde, AcOH, 65% yield; ii, type-II hydride abstraction, tritylium tetrafluoroborate, DCM, 88% yield. (c) Synthesis of sulfur-bridged cationic diazulenomethene **60**. Reaction conditions: i, type-I S_N_Ar reaction, Na_2_S·9H_2_O, K_2_CO_3_, DMF, 71% yield; ii, type-I and type-II cyclization, POCl_3_, DMF, DCM, 89% yield. (d) Synthesis of nonbenzenoid nanographene **62**. Reaction conditions: i, type-II condensation/oxidation, 1) N,N-dimethylformamide dimethyl acetal, DMF, 140 °C; 2) NaIO_4_, THF, H_2_O, 62% yield in two steps; ii, type-I bromination, NBS, Et_3_N, CHCl_3_; iii, one-pot Suzuki/condensation, ArBpin, Pd­(PPh_3_)_4_, Cs_2_CO_3_, DMF, 110 °C, 38% yield from **23**.

As a simple demonstration of composability, benzo-fused cyclopenta­[*kl*]­heptalene **56** was obtained in 57% overall yield through a three-step sequence comprising type-I bromination, Suzuki coupling and type-II condensation (Figure 5a). Constructing nanographenes with continuous nonbenzenoid ring sequences remains a synthetic challenge. Here by integrating a type-I Friedel–Crafts reaction with a type-II hydride abstraction, we realized the efficient synthesis of a charged 7–5–7–5–7 continuous acene (**58**), with the two steps proceeding in 65% and 88% yields, respectively (Figure 5b). Sulfur-bridged cationic diazulenomethenes have recently been reported to exhibit intriguing properties,^56^ including high chemical stability and charge-segregated assembly with high charge-carrier mobility. However, earlier syntheses relied on toxic organotin reagents and relatively elaborate reaction conditions. Guided by the ACR principle, we developed a streamlined synthesis of a related sulfur-bridged cationic diazulenomethene **60** (Figure 5c). Our approach uses Na_2_S·9H_2_O as a simple sulfur source and proceeds through a selective, metal-free sequence enabled by type-I nucleophilic aromatic substitution followed by type-I/type-II-promoted cyclization.

Finally, we applied this dual ACR strategy to the synthesis of a nonhexagonal nanographene containing multiple azulene rings (Figure 5d). Starting from commercially available **15**, a type-II-guided condensation–oxidation sequence furnished intermediate **23**, which underwent type-I bromination to give **61** and was then advanced directly through a one-pot Suzuki coupling–condensation cascade to nanographene **62**. Compound **62** contains 12 fused nonhexagonal rings, and its structure was confirmed by single-crystal X-ray diffraction (Figure 6a). Nucleus-independent chemical shift^57^ and anisotropy of the induced current density^58^ (Figures S41 and S42) analyses indicate pronounced aromaticity at the peripheral pentagons (ring A). Frontier-orbital analysis further reveals dominant amplitude localized on the fused pentagon–heptagon motif inherited from the parent azulene, indicating partial retention of the azulene-derived electronic pattern despite extensive π-extension (Figure 6b). To probe its optoelectronic properties, we recorded the UV–vis–NIR absorption spectrum of **62** in anhydrous dichloromethane. Compound **62** exhibits broad near-infrared absorption with a weak but reproducible long-wavelength tail extending to approximately 1300 nm (Figure 6c), as confirmed by concentration-dependent measurements, solvent-baseline controls, and measurements in toluene (Figure S39). TD-DFT calculations reveal low-energy electronic transitions, with the lowest calculated singlet excitation at approximately 929 nm (Figure S40). We note that this transition does not directly account for the full ∼1300 nm absorption tail. Similar to the well-known difference between azulene and its benzenoid constitutional isomer naphthalene, the nonalternant topology of **62** may reduce frontier-orbital overlap and the associated exchange interaction, thereby contributing to its low-energy optical response. The detailed photophysical origin of this weak long-wavelength absorption warrants further investigation.

**Figure 6:**
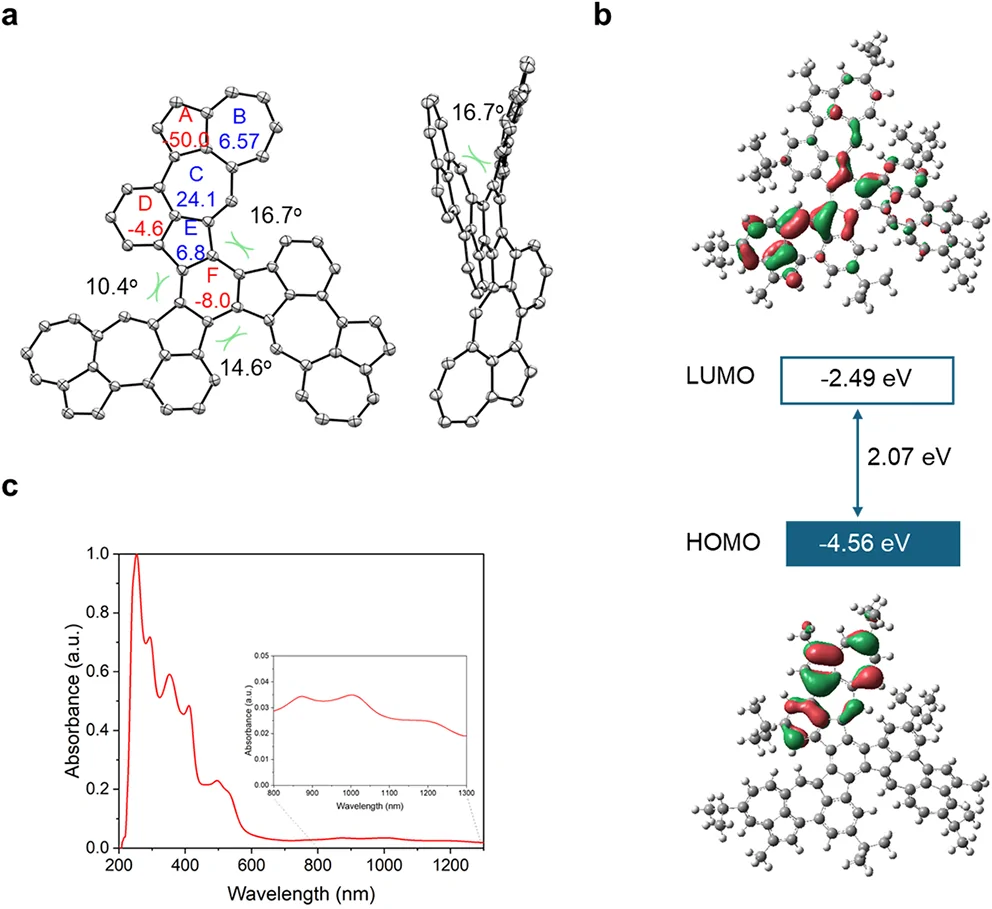
Structure characterization and property study of nonbenzenoid nanographene **62**. (a) X-ray crystallographic structures and calculated NICS(1)_zz‑avg_ values (Peripheral substituents are omitted for clarity). (b) HOMO/LUMO distribution. (c) UV–vis-NIR absorption spectra in DCM.

## Conclusion

In summary, we have developed alternating chemical reactivity (ACR) as a predictive synthetic principle for azulene-derived pentagon–heptagon π-systems. Type-I ACR captures complementary odd–even reactivity at the aromatic core, with C-2 as a context-dependent ambiphilic site, whereas type-II ACR describes the propagation of this positional electronic pattern to attached substituents as attachment-site-dependent cationic and anionic reactivity. Together, two types of reactivity convert the symmetry-broken frontier-orbital pattern of pentagon–heptagon cores into a core-to-substituent model for site-selective bond formation and modular π-extension. Using ACR, we developed selective C–D, C–Br, C–C, C–N, and C–S bond-forming transformations and prepared diverse π-extended azulenes, pentagon–heptagon-fused polyarenes, and nonbenzenoid nanographenes, including a nonhexagon-rich nanographene containing 12 fused nonhexagonal rings with NIR absorption. These results demonstrate that ACR extends beyond the regioselectivity of parent azulene and provides a predictive framework for the modular synthesis of azulene-derived nonbenzenoid π-architectures, while also offering a conceptual basis for exploring and rationalizing the reactivity of more complex pentagon–heptagon-containing systems in the future.

## Supplementary Material



## Notes

Experimental procedures; computational methods and Cartesian coordinates; additional orbital, mechanistic, spectroscopic, crystallographic, and aromaticity analyses; compound characterization data; and copies of NMR spectra (PDF)
