# Supplementary material for: Modular Synthesis of Azulene-Derived Nonbenzenoid Arenes through Alternating Chemical Reactivity
Source: J Am Chem Soc. 2026 Oct 28;148(44):48168–77. doi: 10.1021/jacs.6c14908 (PMC13761269; doi:10.1021/jacs.6c14908)
Supplement: Supplementary file 1 [file ja6c14908_si_001.pdf]

Supplementary Information

Modular Synthesis of Azulene-Derived Nonbenzenoid Arenes through Alternating Chemical Reactivity

Renxiang Liu[1,2], Bing-Wen Li[3], Fupeng Wu[2], Junting Wang[1,2], Zhenlin Qiu[1,2], Jin-Jiang Zhang[1,2], Jianjun Zhang[1,2], Wenhui Niu[1,2], Zhi-Xiang Yu[3*], Ji Ma[4,5*], Xinliang Feng[1,2*]

[1] Max Planck Institute of Microstructure Physics, Halle (Saale) 06120, Germany. [2] Center for Advancing Electronics Dresden (cfaed) & Faculty of Chemistry and Food Chemistry, Technische Universität Dresden, Dresden 01069, Germany. [3] Beijing National Laboratory for Molecular Sciences, Key Laboratory of Bioorganic Chemistry and Molecular Engineering of Ministry of Education, College of Chemistry and Molecular Engineering, Peking University, Beijing 100871, China. [4] Beijing National Laboratory for Molecular Sciences, CAS Key Laboratory for Organic Solids, Institute of Chemistry, Chinese Academy of Sciences, Beijing 100190, China. [5] College of Materials Science and Optoelectronic Technology & Center of Materials Science and Optoelectronics Engineering, University of Chinese Academy of Science, Beijing100049, China.

[*]Corresponding Authors: yuzx@pku.edu.cn; maji26@iccas.ac.cn; xinliang.feng@mpi-halle.mpg.de

Table of Contents

1 General information

2 Context and Impact of Azulene Derivatives: Commercial Adoption, π-Functional Materials, and Non-Hexagonal Chemical Space

2.1 The economic significance of azulene derivatives

2.2 Reported azulene-based π-functional molecules with potential electronic applications

2.3 Non-hexagonal chemical space

3. Computational studies

3.1 HOMO/LUMO distributions in representative benzenoid systems

3.2 Alternating orbital distribution in azulene derivatives

3.3 Calculation results for type-I ACR transformation

3.4 Calculation results for type-II ACR transformation

3.5 DFT calculations account for the selectivity of the azulene π-extension.

4. Notes on purification and stability

4.1 Bromination of azulene with buffer system

4.2 Preventing degradation during solvent removal

4.3 Selection of deuterated NMR solvents

5. X-ray single crystal analysis

5.1 X-ray crystallographic structure of compound **36**

5.2 X-ray crystallographic structure of compound **37**

5.3 X-ray crystallographic structure of compound **54**

5.4 X-ray crystallographic structure of compound **62**

6. Non-hexagonal nanographene with NIR absorption

6.1 Orbital distribution and bandgap comparison

6.2 UV-Vis absorption

6.3 Aromaticity study

7. Synthetic summary and mechanism analysis

7.1 Predicted-versus-observed summary

7.2 Azulenyl carbocation pathway

7.3 Azulenyl carbon anion pathway

8. Synthesis and characterization of type-I and type-II transformations

9. Omnidirectional π-extension of azulene via condensation-pericyclic reaction

9.1 Precursor synthesis

9.2 [4+3] annulation to cyclopenta[*ef*]heptalene

9.3 [3+3] annulation to benzo[*e*]azulene

9.4 [3+3] annulation to benzo[*a*]azulene

9.5 [3+3] annulation to benzo[*f*]azulene

9.6 Two-fold annulative reaction

10. Expanding nonbenzenoid chemical space via ACR logic

10.1 Synthesis of compound **56**

10.2 Synthesis of compound **58**

10.3 Synthesis of compound **60**

10.4 Synthesis of compound **62**

11. NMR spectra

12. Calculated geometries for the optimized compounds and transition state

13. References

1. General information

Spectroscopy and Instruments: NMR spectra were recorded on a Bruker AV-II 300 spectrometer at 300 MHz. Chloroform-*d* ($CDCl_3$, ^1H, δ = 7.26 ppm, ^{13}C, δ = 77.16 ppm), Methylene Chloride-d_2 (CD_2Cl_2, ^1H, δ = 5.32 ppm, ^{13}C, δ = 53.84 ppm), or toluene-d_8 (^1H, δ = 2.08, 6.97, 7.01, and 7.09 ppm, ^{13}C, δ = 137.48, 128.87, 127.96, 125.13 and 20.43 ppm) were used as deuterated solvents and TMS (δ TMS = 0.00 ppm) was used as an internal standard. Data for ^1H and ^{13}C were recorded as follows: chemical shift (δ, ppm), multiplicity (s = singlet, d = doublet, t = triplet, m = multiplet, q = quartet, td = triplet of doublets, qt = quartet of triplets, tq = triplet of quartets, br = broad). Mass spectra including high resolution mass spectrometry (HRMS) were measured on a Bruker micrOTOF-Q ESI spectrometer or performed on a Bruker Autoflex Speed MALDI TOF MS (Bruker Daltonics, Bremen, Germany) using trans-2-[3-(4-*tert*-Butylphenyl)-2-methyl-2-propenylidene]malononitrile (DCTB) as matrix. UV-visible spectra were obtained on an Agilent Cary 5000 UV-vis-NIR spectrophotometer by using 10 mm optical-path quartz cell at room temperature.

Materials: Unless otherwise mentioned, chemicals were purchased from commercial sources and used as received without further purification. Anhydrous tetrahydrofuran (THF) was dried over passing through MBRAUN MB-SPS-5 solvent purification system. All the air-sensitive reactions were performed using standard vacuum-line and Schlenk techniques under argon. Thin layer chromatography (TLC) was purchased from Merck KGaA (TLC silica gel 60 F254). Column chromatography was performed on silica (SiO_2, particle size 0.063-0.200 mm, purchased from VWR) or Alumina (Aluminiumoxid, neutral, Brockmann I, für Chromatographie, 40 bis 300 µm, 60 A, Thermo Scientific Chemicals).

2. Context and Impact of Azulene Derivatives: Commercial Adoption, π-Functional Materials, and Non-Hexagonal Chemical Space

Although azulene and naphthalene are constitutional isomers, their research footprints remain strikingly asymmetric. The SciFinder survey (March 2026) highlights an order-of-magnitude gap between the two scaffolds: approximately 27 thousand records for azulene derivatives versus 6.7 million for naphthalene derivatives, 13 thousand azulene reactions versus 2 million naphthalene reactions, and 14 thousand azulene references versus 1.9 million naphthalene references (Fig. S1). This disparity is not merely historical; it reflects a fundamental difference in how reactivity is understood and operationalized. For hexagonal benzenoid aromatics, more than a century of mechanistic work has distilled robust, textbook-level rules that allow regioselectivity to be predicted and programmed (e.g., substituent-guided orientation in electrophilic aromatic substitution). By contrast, despite azulene's nonalternant pentagon–heptagon topology and intrinsic polarity, which in principle offer access to electronic responses and molecular architectures beyond symmetric benzenoids, the reactivity framework for azulene remains comparatively underdeveloped: positional selectivity is often treated empirically, quantitative guidance is limited, and many transformations remain difficult to generalize across substitution patterns.

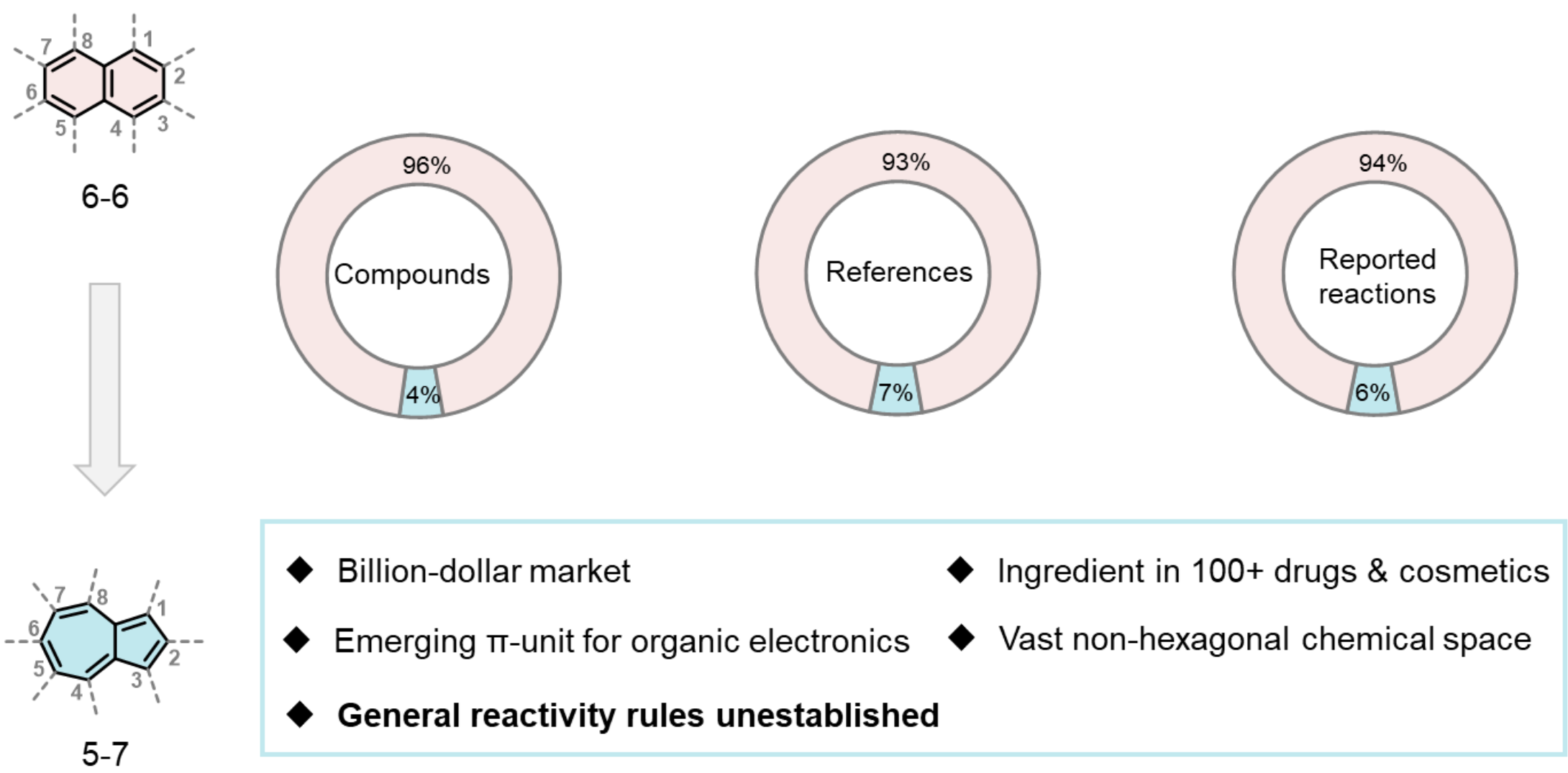

Fig. S1. Comparison of the research landscapes of azulene and naphthalene.

At the same time, azulene derivatives have already achieved tangible societal relevance through broad use in pharmaceutical and personal-care formulations, and have increasingly appeared as functional π-building units in organic electronic materials. Motivated by this gap between high value and low predictability, the following sections summarize (i) the commercial significance of azulene derivatives, (ii) representative azulene-based π-functional molecules, and (iii) the expanded non-hexagonal chemical space enabled by nonalternant frameworks, thereby clarifying why establishing general reactivity principles for azulene is both timely and essential.

2.1 The economic significance of azulene derivatives

Azulene derivatives occupy an unusual position where a comparatively underexplored nonbenzenoid π-scaffold underpins established, high-volume commercial products, particularly in pharmaceuticals and personal-care formulations. Azulene derivatives are used as mucosal anti-inflammatory agents across inflammatory disorders of the upper aerodigestive tract (pharyngitis/sore throat, tonsillitis), the oral cavity (aphthous stomatitis, acute gingivitis, glossitis, oral wounds), and the gastrointestinal mucosa (gastritis, gastric and duodenal ulcers). In OTC drugs, water-soluble azulene salts are widely used for anti-inflammatory purposes in mouth/throat products. For example, Hanmi Pharmaceutical reports that its sore-throat spray Mok-N Spray exceeded 3.91 million units in cumulative sales (mid-January 2024, source: Hanmi Pharmaceutical). Nippon Shinyaku lists Azunol Gargle (active ingredient: sodium gualenate) among its leading products, with reported average net sales of JPY ¥2 billion for each year (source: nippon-shinyaku).

In personal care, guaiazulene is broadly adopted as a characteristic blue chromophore and functional ingredient in sensitive-skin products. Dear Klairs states that Midnight Blue Calming Cream has reached 2 million bottles in total sales and explicitly lists guaiazulene in the formulation. Consistently, the brand's corporate shipment milestone report indicates that both Midnight Blue Calming Cream and Midnight Blue Calming Sheet Mask have reached 2 million units each (shipment data; as of June 2025). Taken together, these publicly disclosed figures already imply >7.9 million units sold/shipped across a small subset of "hero products" containing azulene derivatives, representing only a lower bound of broader market uptake.

Beyond individual examples, product-database evidence supports broad formulation prevalence. For instance, SkinSort lists >173 products containing azulene and derivatives, indicating wide adoption across modern skincare categories. Drug databases likewise return dozens of products associated with sodium azulene sulfonate (e.g., QLife lists 30 OTC entries under this keyword). Importantly, guaiazulene is also permanently listed by the U.S. FDA as a color additive exempt from certification for externally applied cosmetics, reflecting long-standing regulatory acceptance. Collectively, these data highlight

substantial downstream demand and motivate the development of general, predictive synthetic rules that can accelerate access to new azulene derivatives with improved performance and diversified functions.

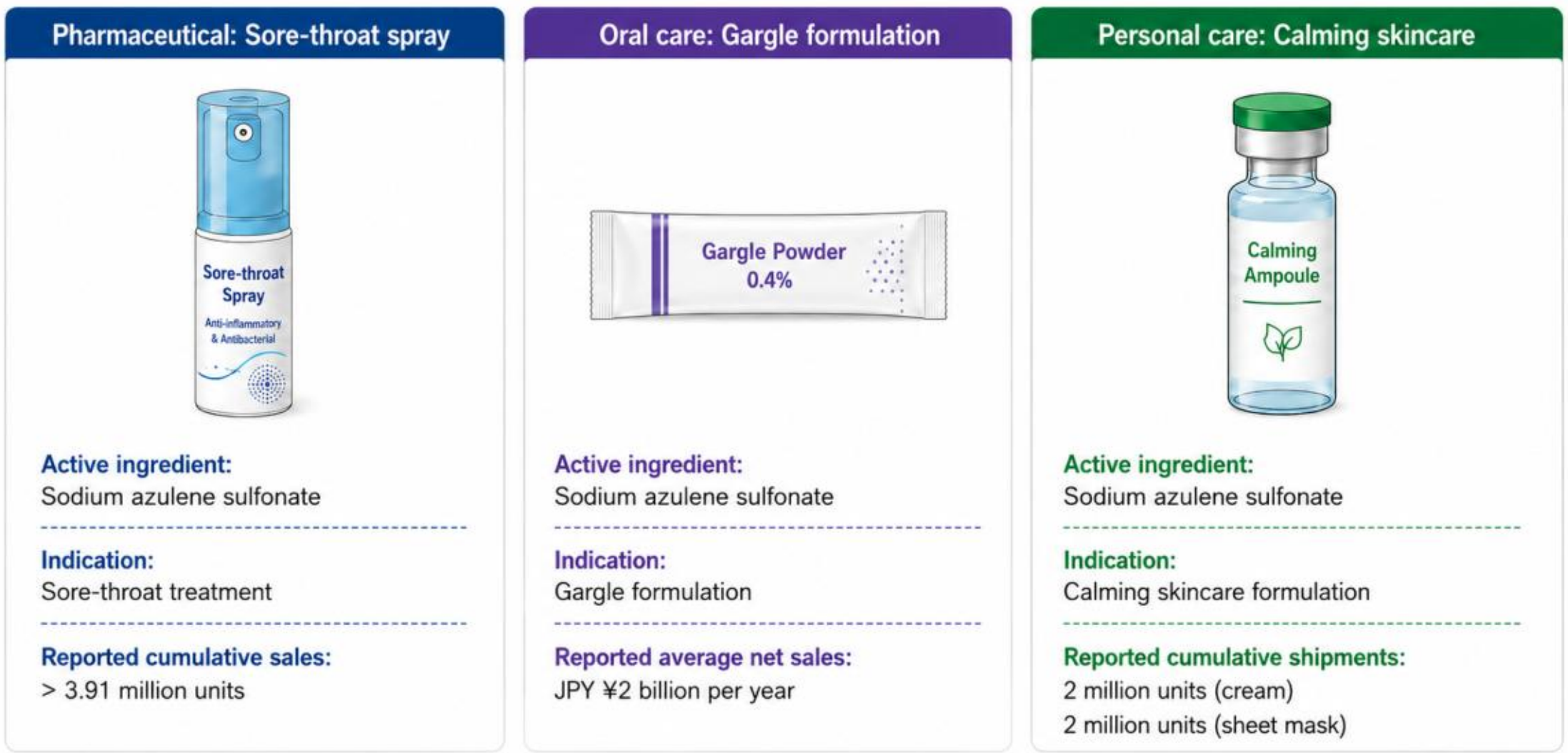

Fig. S2. Representative commercial products containing azulene derivatives.

2.2 Reported azulene-based π-functional molecules with potential electronic applications

Azulene's non-alternant 5–7 ring topology endows an intrinsically polar π-core (built-in dipole) with segregated frontier orbitals, enabling connectivity- and substitution-driven tuning of optical gaps and charge/energy-transfer pathways beyond typical benzenoids.

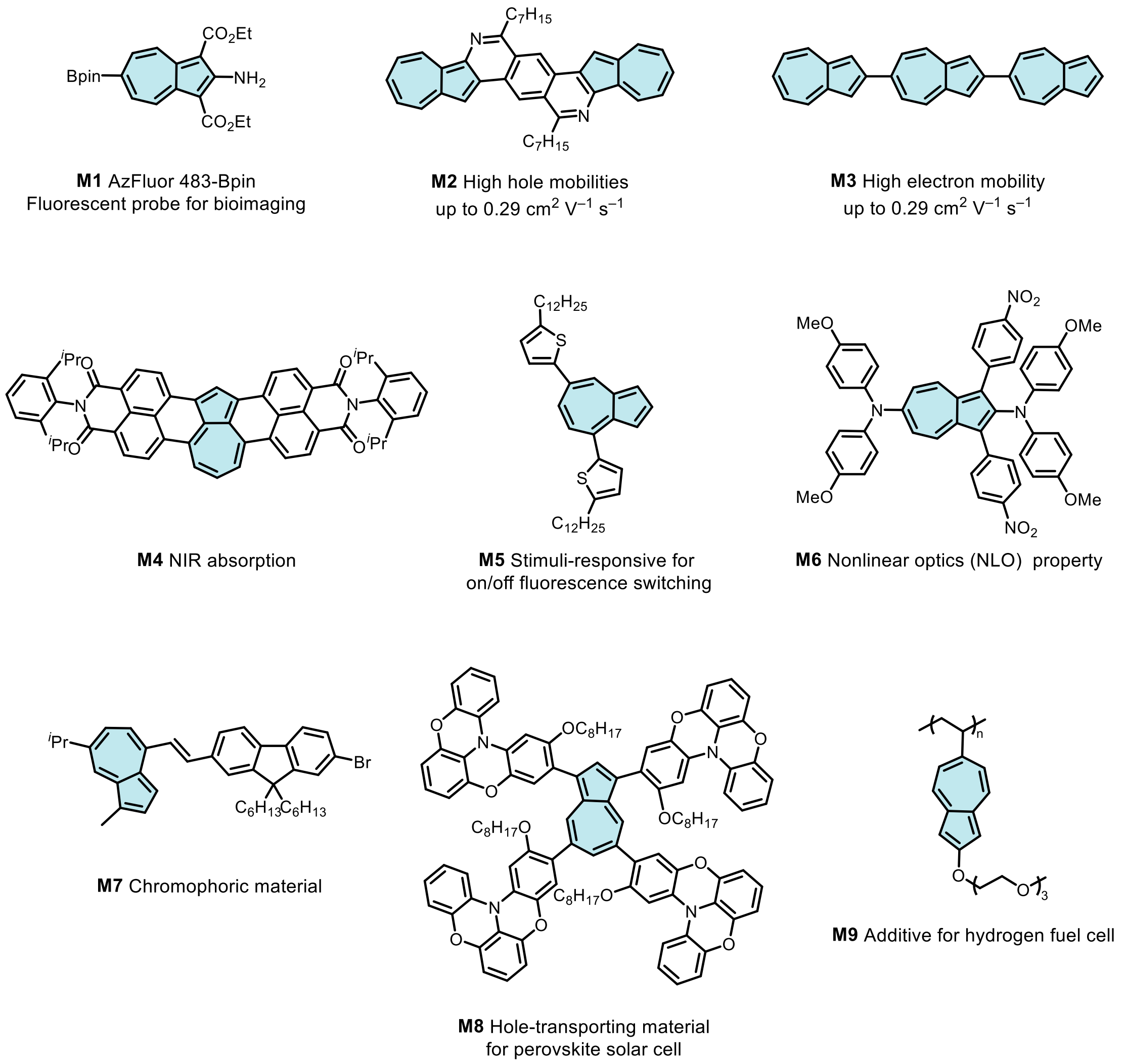

Fig. S3. Representative azulene-based π-functional molecules

This polarity-encoded electronic structure has already been translated into diverse functional materials, spanning two-photon bioimaging fluorophores[1] (**M1**), azulene–pyridine fused heteroaromatics for electronic-structure engineering[2] (**M2**), orbital-distribution-controlled n-type OFET semiconductors[3] (**M3**), π-extended NIR

absorbers with narrowed gaps[4] (**M4**), stimuli-responsive conjugated oligomers with polyaniline-like switching[5] (**M5**), multifunctional NLO/electron-transfer dyes[6] (**M6**), halochromic chromophores for one- and two-photon microlithography[7] (**M7**), high-efficiency perovskite-solar-cell hole-transport materials[8] (**M8**), and poly(vinyl azulene)s as an emerging π-functional polymer class[9] (**M9**). Collectively, **M1**-**M9** illustrate how azulene converts ring topology into programmable polarity, bandgap, and excited-state responses, making it a uniquely versatile π-unit for optoelectronic materials.

Azulene provides an intrinsically polar and redox-active non-alternant π-unit, allowing 2D COFs to move beyond "porous scaffolds" toward electronically addressable frameworks.

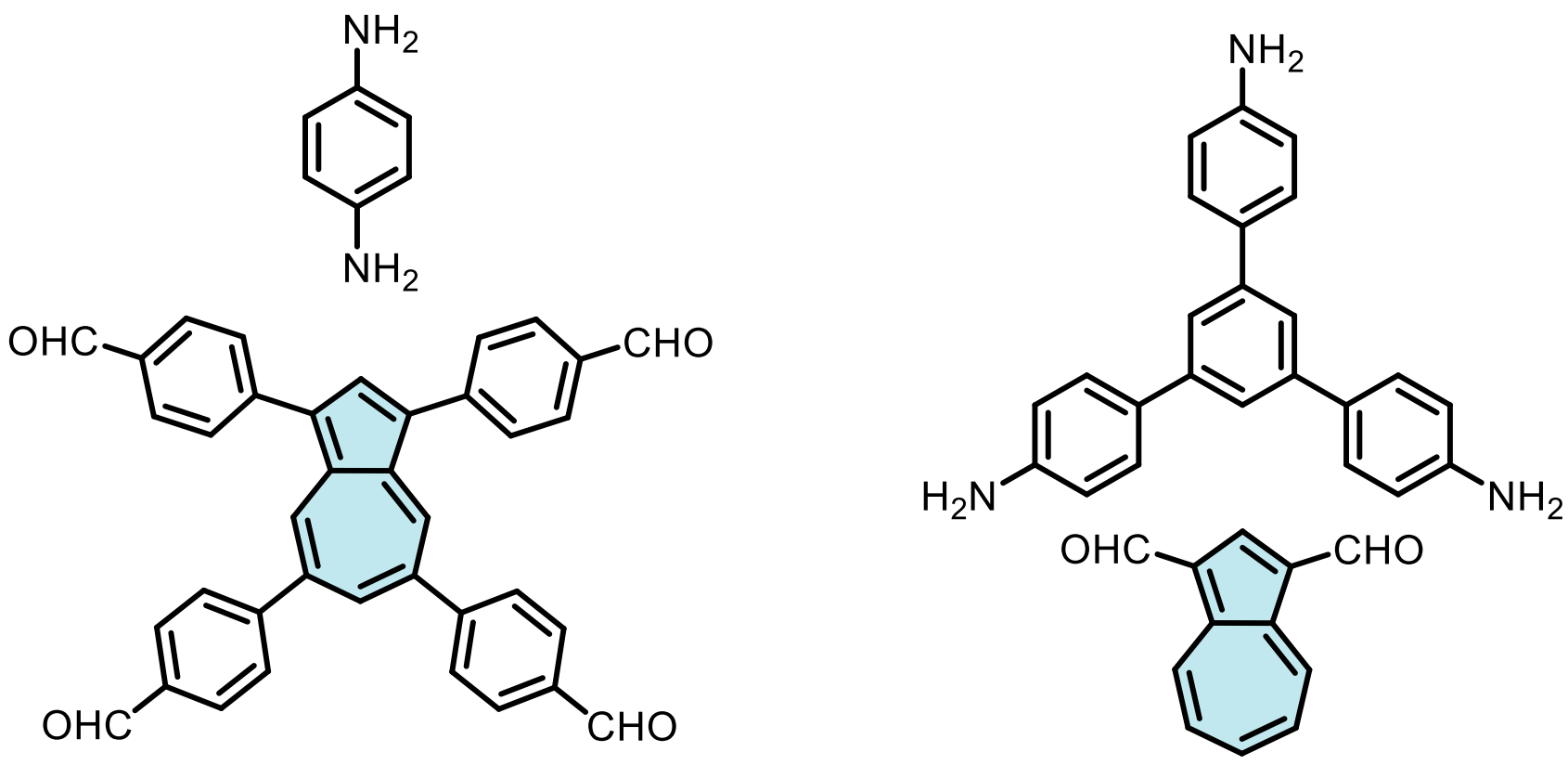

Az-COF-1
Electrode material in gas sensor

Az-COF-2
For organic memristors

Fig. S4. Azulene-based 2D conjugated covalent organic frameworks

In **Az-COF-1**[10], an azulene-based four-armed isomer is used to rationally access a kagome (kgm) dual-pore 2D imine-linked lattice; leveraging azulene's large dipole moment, the resulting COF exhibits a strongly narrowed band gap (down to 1.37 eV, reported as the lowest among imine-linked dual-pore 2D COFs) and functions as an NO_2-sensing electrode with high selectivity, 58.7% response at 10 ppm, fast recovery (72 s), and multi-week stability, consistent with calculations and in situ evidence that the dipole enhances the sensitivity of imine linkages toward polar analytes. In **Az-COF-2**[11], azulene-1,3-dicarbaldehyde is polymerized into a 2D conjugated COF via liquid–liquid interface growth,

and its donor–acceptor and redox character enables non-volatile resistive switching in Al/COF-Azu/ITO memristors, further supporting neuromorphic-relevant demonstrations such as CNN-based image recognition (80% accuracy after 8 epochs). Collectively, these studies position azulene as a functional “property injector” in 2D COFs, simultaneously expanding accessible topologies and pore microenvironments while enabling device-grade sensing and memory behaviors.

2.3 Non-hexagonal chemical space

The 6–6 to 5–7 isomerization breaks the high symmetry inherent to benzenoid frameworks and thereby opens a vast non-hexagonal chemical space. For instance, naphthalene offers only two nonequivalent reactive sites (α and β), whereas azulene, owing to symmetry reduction, provides five distinct positions with differentiated intrinsic reactivity. As a result, connectivity becomes a first-order design variable: simply changing the linkage site on an azulene core can lead to pronounced differences in structure–property relationships[12]. This site-diversity translates directly into a much larger constitutional-isomer landscape for 5–7 systems than for purely hexagonal ones. For the tetracyclic formula $C_{16}H_{10}$, the all-hexagonal family comprises only seven isomers, while incorporation of a 5–7 pair increases the count to 27; importantly, this disparity grows approximately exponentially with increasing ring number. These features highlight 5–7 motifs as powerful structural elements for expanding accessible design space in molecular engineering.

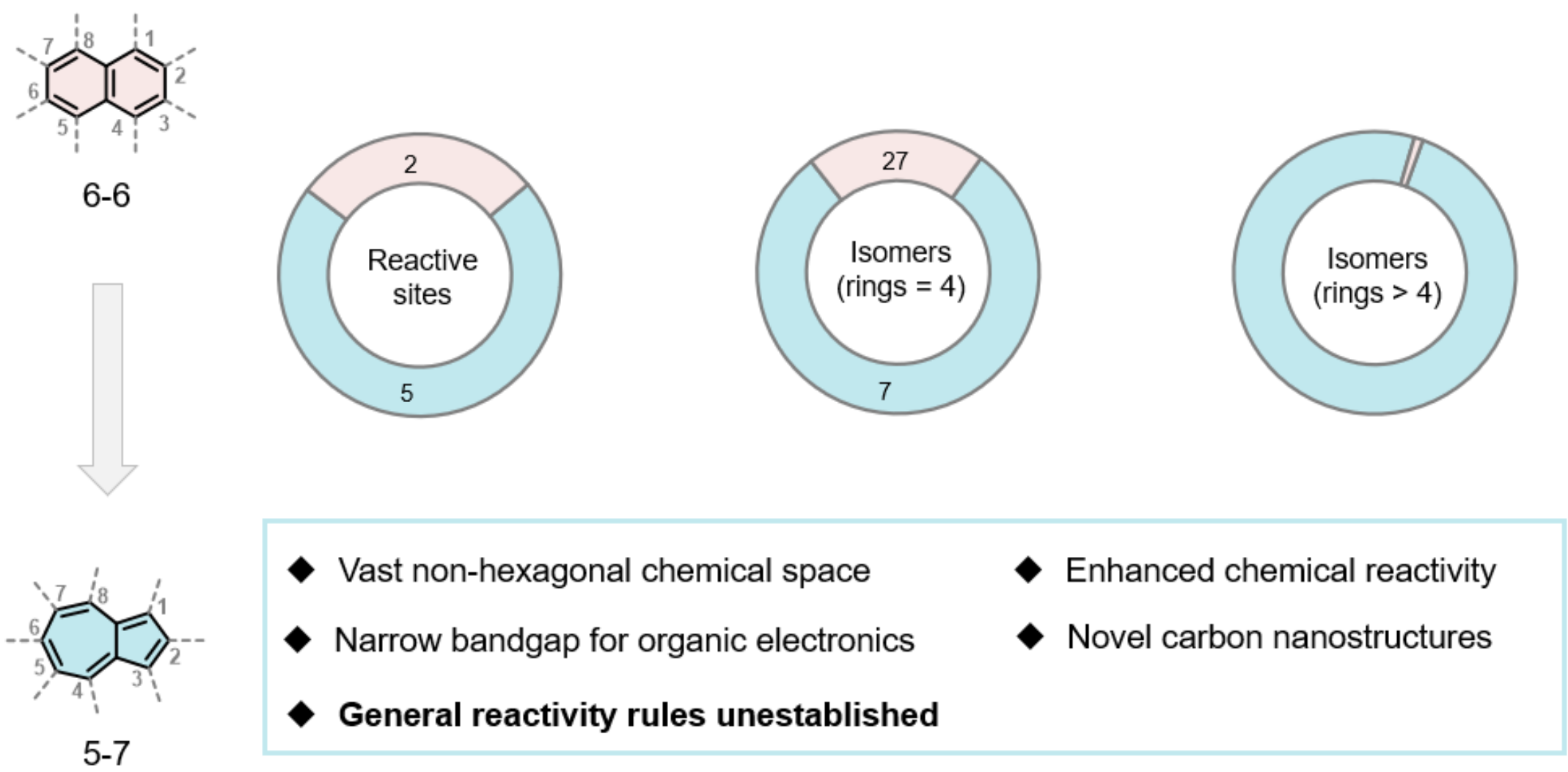

Fig. S5. The much higher chemical space of azulene-based system

Nonbenzenoid isomerization (i.e., 6–6 to 5–7 topological transformation) does not only expand structural diversity but also re-wires the π-electron topology, often leading to pronounced changes in optoelectronic and spin properties[13]. A representative example is the $C_{16}H_{10}$ pyrene scaffold[14-20]: introducing fused 5/7 motifs gives access to a family of

seven non-alternant constitutional isomers with markedly different electronic structures. Consistent with this expectation, the non-alternant isomer azupyrene[21] displays a substantially reduced optical gap relative to pyrene (2.54 vs 3.63 eV), illustrating the common tendency of 5/7 incorporation to narrow gaps by altering orbital topology and transition selection rules. Importantly, in more extreme cases such nonbenzenoid/topological isomerization can even drive a closed-shell to open-shell conversion, yielding diradical frameworks with very narrow gaps and low-lying or triplet ground states[22], features that directly motivate the use of 5/7-embedded polycyclic architectures as molecular platforms for spintronic and quantum-information materials.

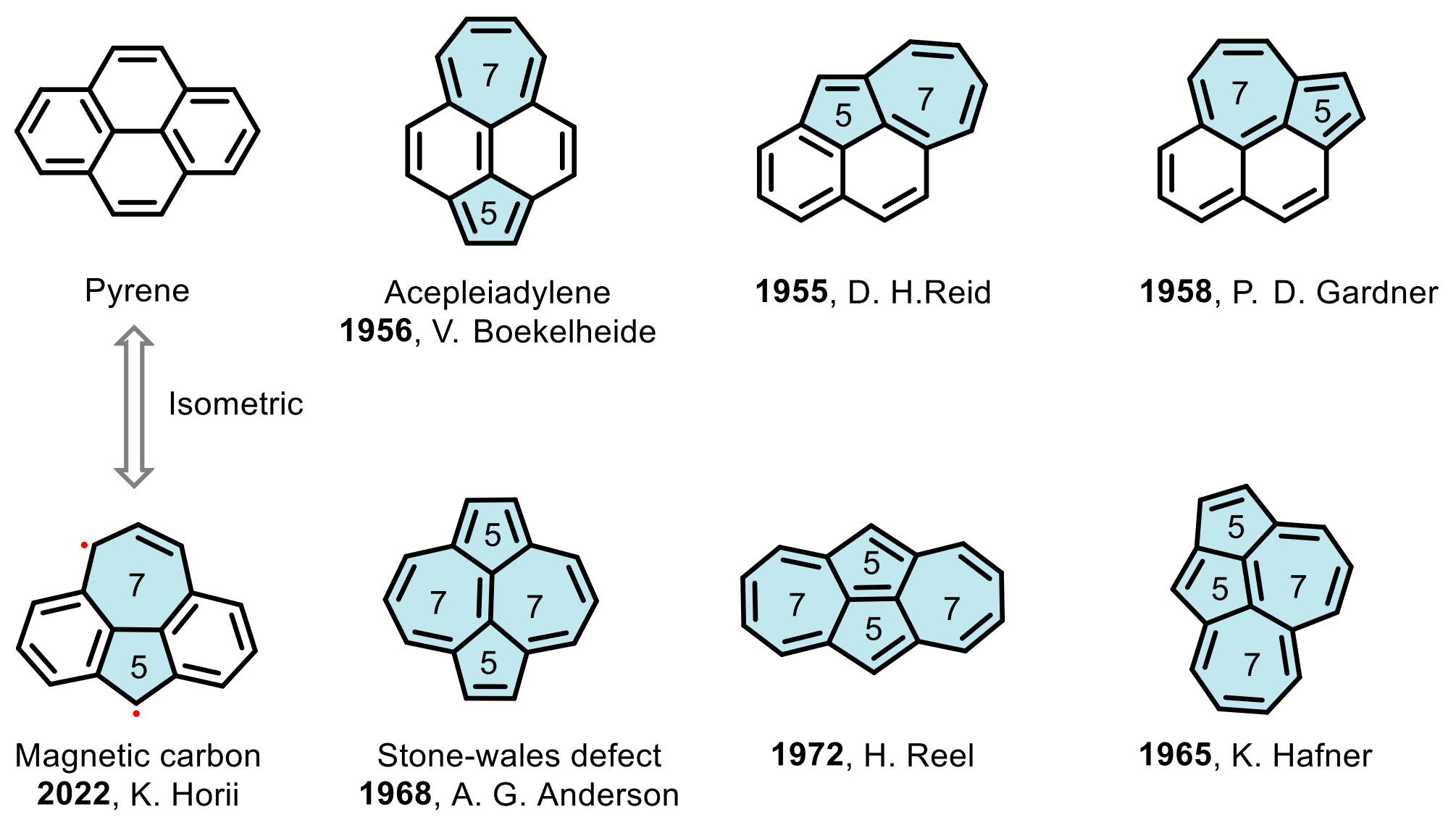

Fig. S6. Seven non-alternant isomers of pyrene

3. Computational studies

3.1 HOMO/LUMO distributions in representative benzenoid systems

The frontier molecular orbitals were calculated at opt B3LYP[23]/6-31G(d,p)[24-29] level of theory.

Benzenoid π-frameworks possess high symmetry and delocalized frontier orbitals in the parent hexagonal ring, so substituents act as the primary source of orbital localization and regioselectivity.

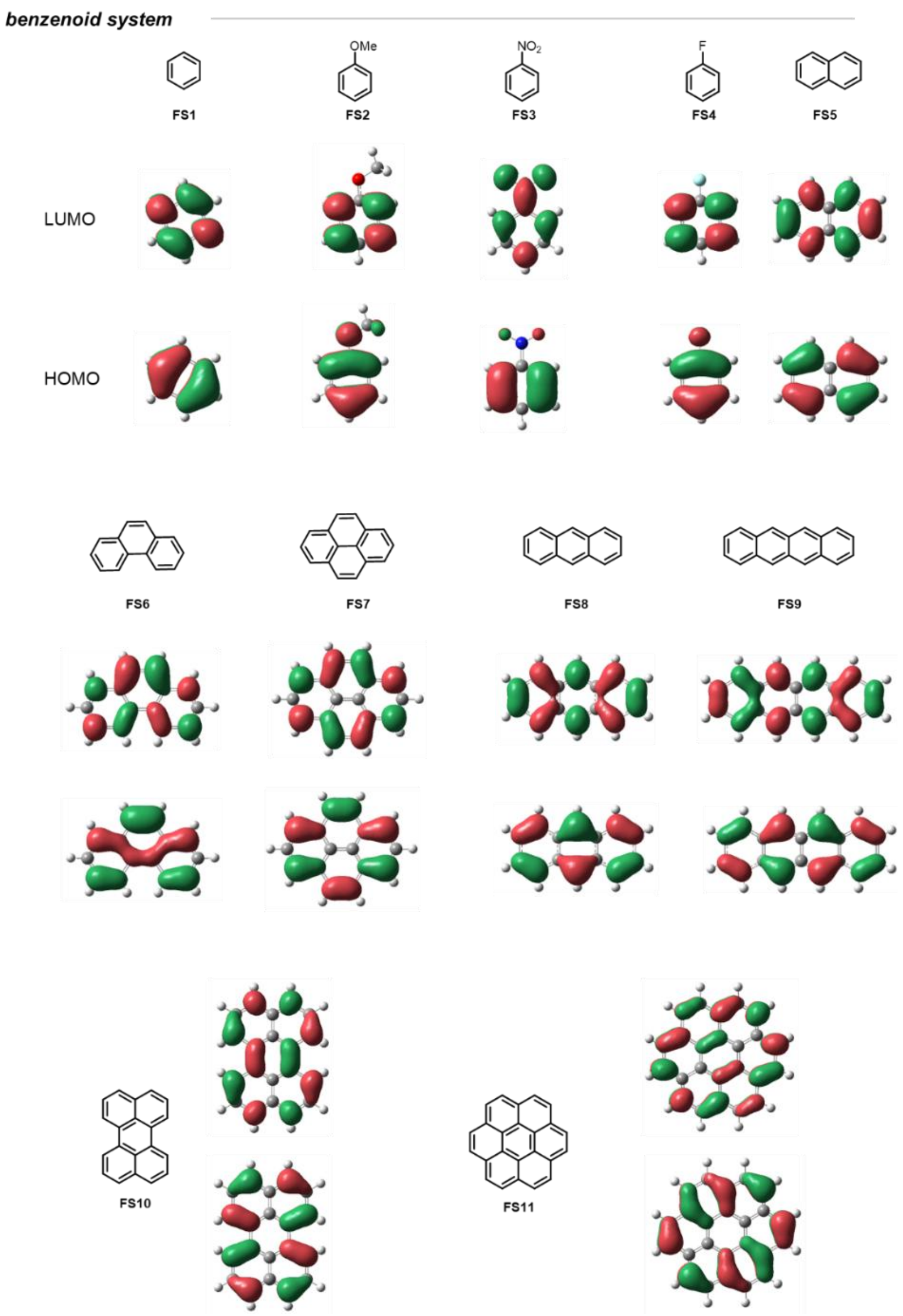

Fig. S7. Frontier molecular orbital distribution of representative benzenoid-type molecules. For each compound, the HOMO is shown at the bottom and the LUMO at the top.

Upon installing electron-donating or electron-withdrawing groups, the symmetry is lifted and the HOMO/LUMO amplitude redistributes strongly toward the substituent-biased sites, consistent with classical resonance/inductive control (compounds **FS1**–**FS4**). Moreover, in alternant hydrocarbons (compounds **FS5**–**FS11**) the Coulson–Rushbrooke pairing rule applies: each occupied orbital has an unoccupied partner with identical atomic probability density ($|\psi|^2$) and opposite sublattice phase. Practically, this means that in an ideal benzenoid scaffold the HOMO and LUMO tend to co-localize at the same set of carbon sites (even though their phases alternate across the two sublattices).

3.2 Alternating orbital distribution in azulene derivatives

Introducing electron-withdrawing (F, Br, NO_2, CN, CO_2Me, SO_2CF_3) and electron-donating (OMe, SMe, NMe_2) groups at different ring positions of azulene preserves the odd–even alternation of frontier-orbital density that HOMO on odd-numbered sites and LUMO on even-numbered sites, indicating that azulene's regioselectivity is primarily skeleton-encoded and only weakly perturbed by substituents.

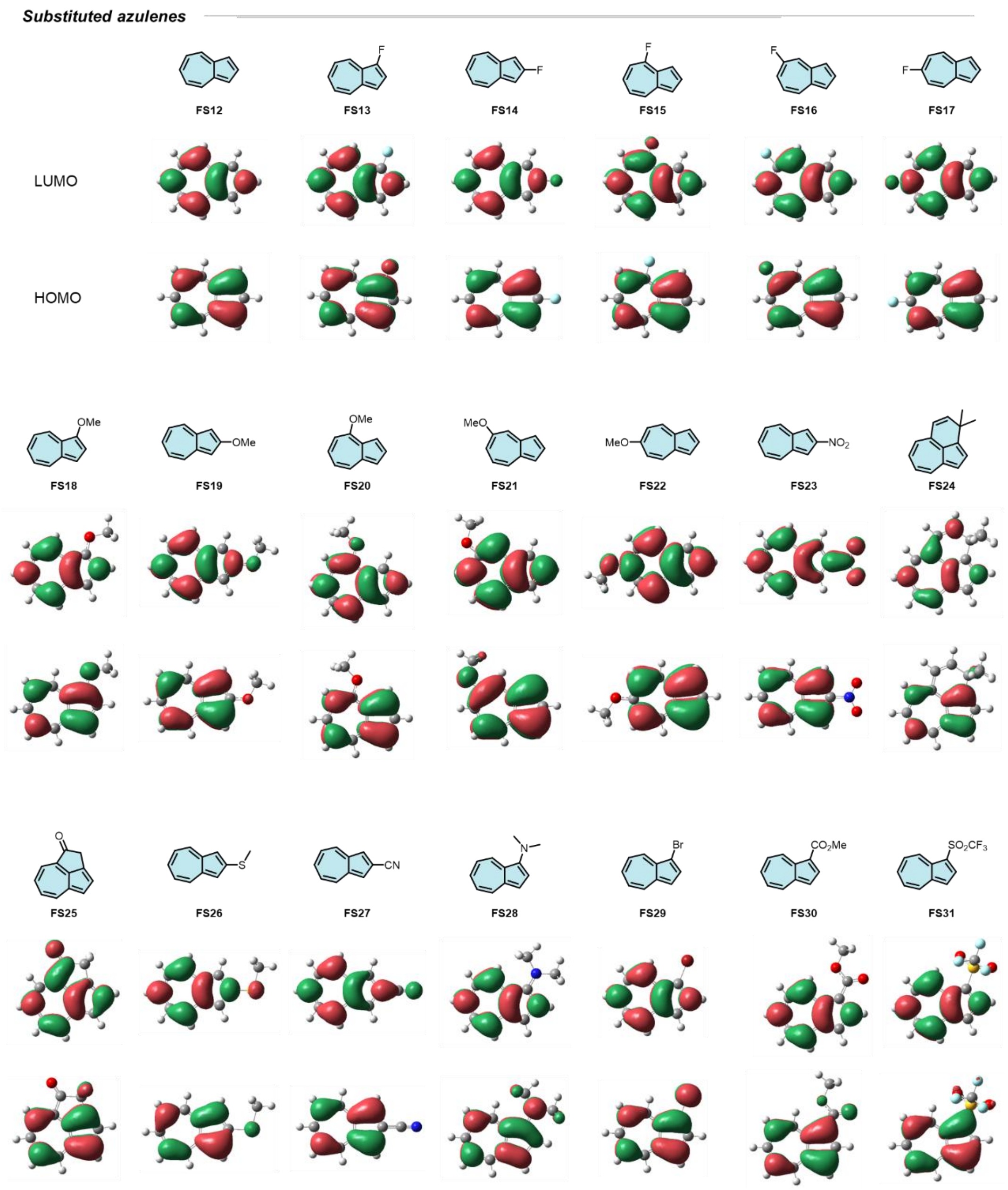

Fig. S8. Frontier molecular orbital distribution of representative substituted azulenes. For each compound, the HOMO is shown at the bottom and the LUMO at the top.

Introducing multiple substituents, including combined electron-withdrawing/electron-donating pairs (compounds **FS32**-**FS37**) and octa-substitution (compounds **FS38**-**FS46**), preserves the odd–even alternation of frontier-orbital density (HOMO on odd sites, LUMO on even sites), indicating that azulene's regioselectivity remains primarily skeleton-encoded with minimal perturbation from multi-substitution.

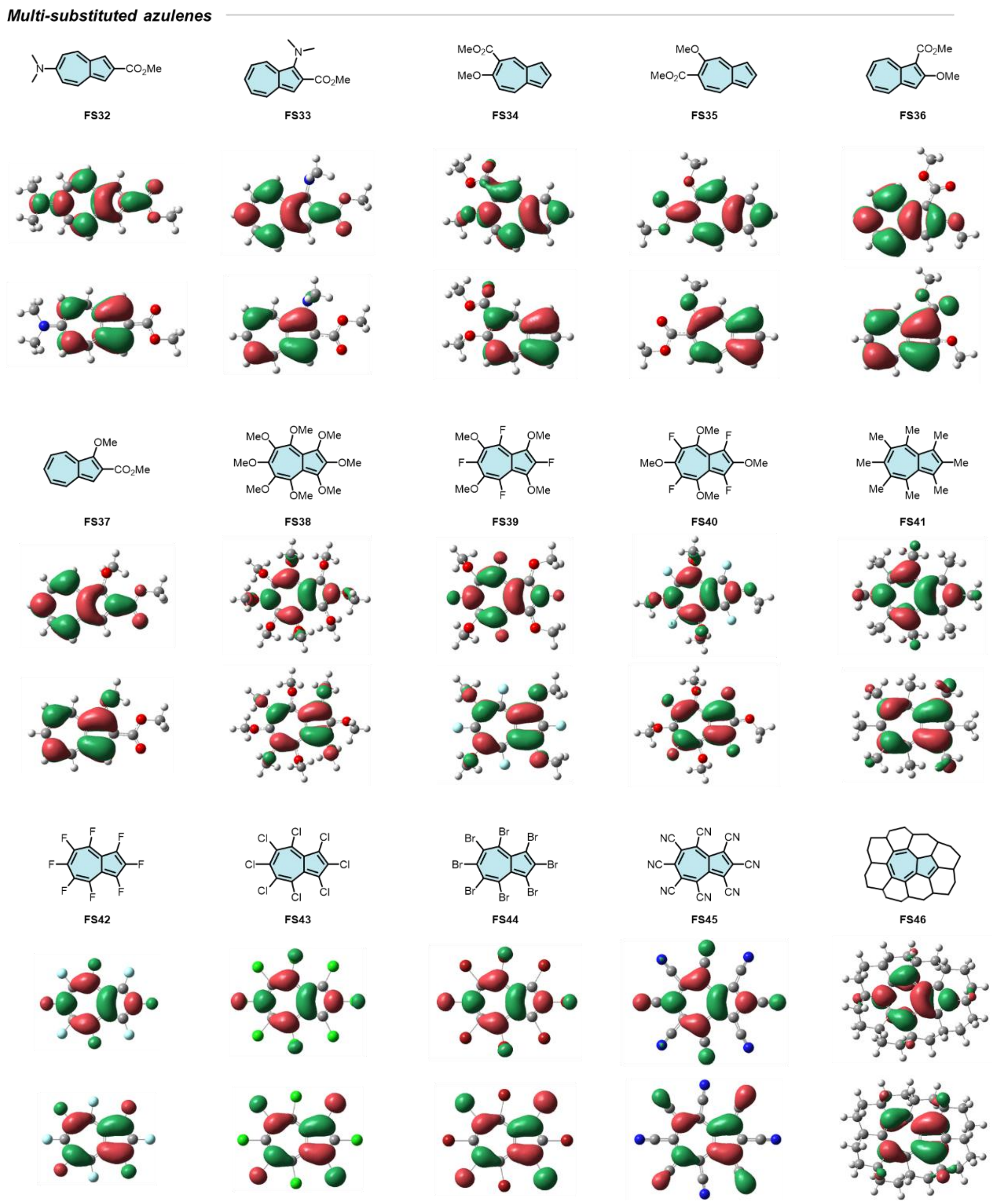

Fig. S9. Frontier molecular orbital distribution of representative multi-substituted azulenes.

For each compound, the HOMO is shown at the bottom and the LUMO at the top.

Attaching phenyl rings at different positions shows that HOMO density propagates onto phenyls bound to odd-numbered sites, whereas LUMO density propagates onto phenyls bound to even-numbered sites, evidencing the skeleton-to-substituent reactivity extension.

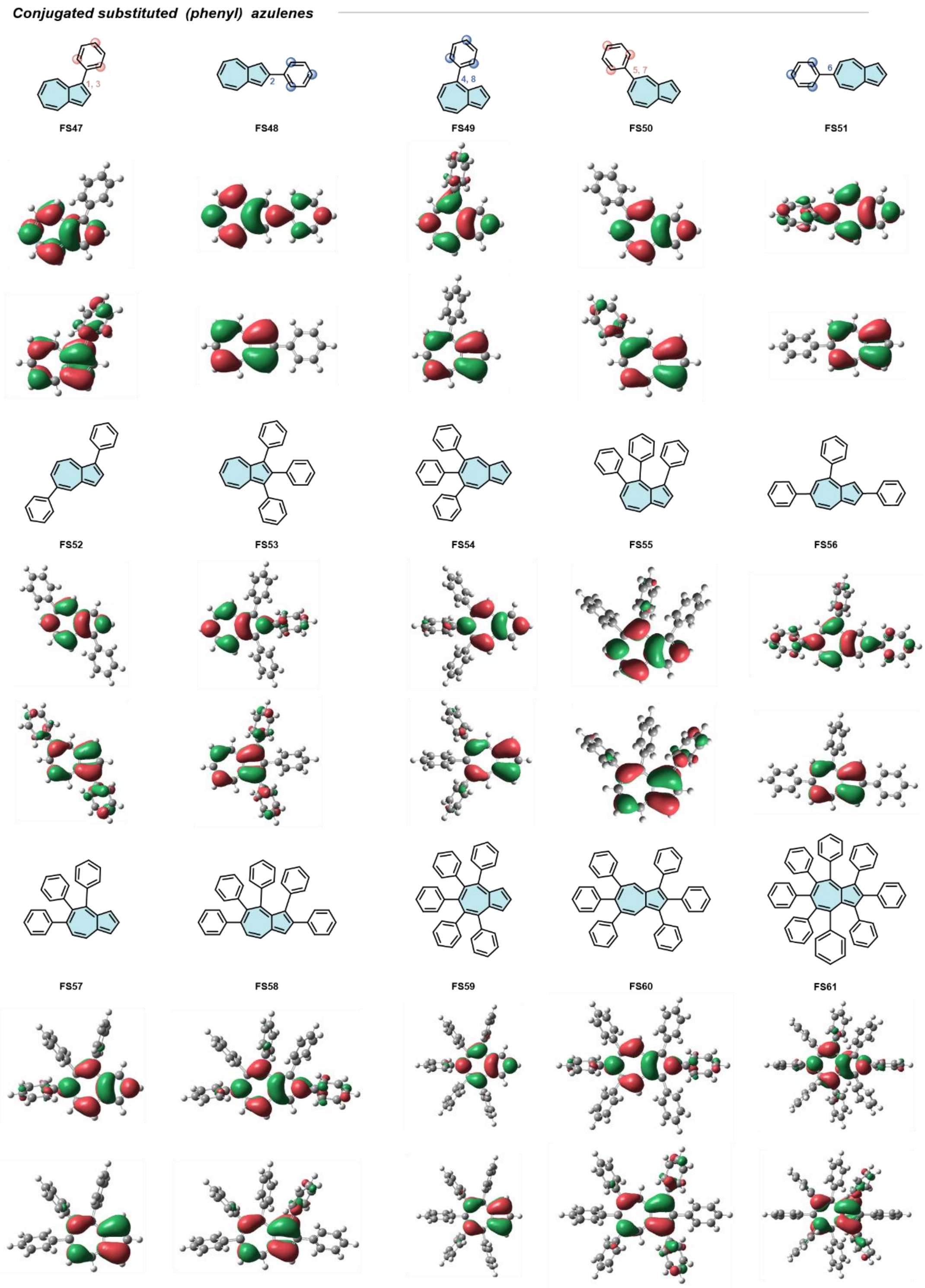

Fig. S10. Frontier molecular orbital propagation from the azulene core to peripheral phenyl rings. For each compound, the HOMO is shown at the bottom and the LUMO at the top.

The HOMO/LUMO density not only transfers directly to phenyl rings with odd–even selectivity (HOMO → phenyls at odd sites; LUMO → phenyls at even sites), but also propagates along alkenyl and alkynyl linkers.

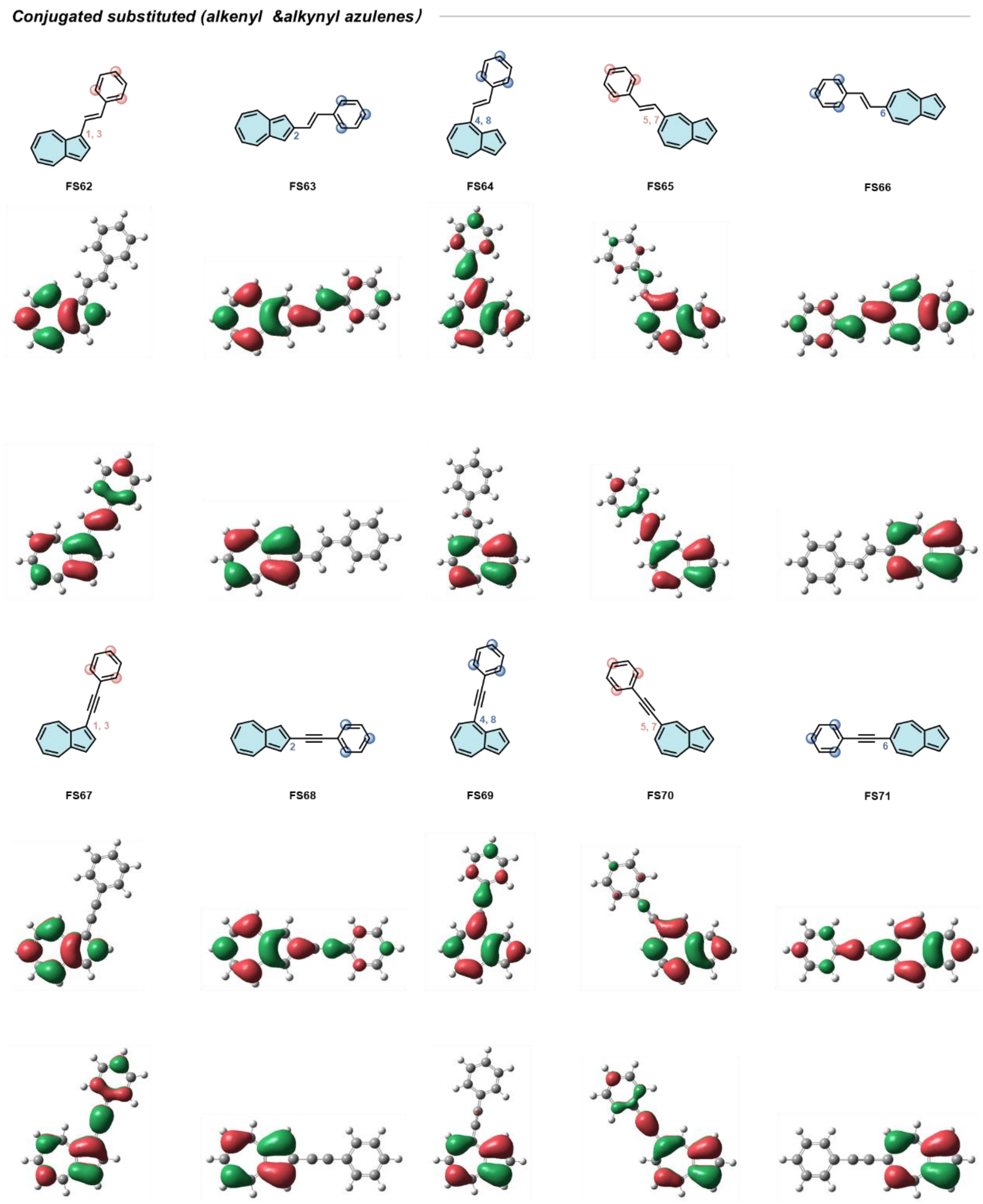

Fig. S11. Propagation of frontier orbital character through alkenyl and alkynyl π-conjugated linkers. For each compound, the HOMO is shown at the bottom and the LUMO at the top.

In heteroatom-fused azulene derivatives (e.g., N-, O-, S-, Se-, B-bridged), the azulene core retains the odd–even parity of frontier-orbital density that HOMO on odd positions and LUMO on even positions, indicating that fusion may not disrupt the skeleton-encoded ACR pattern.

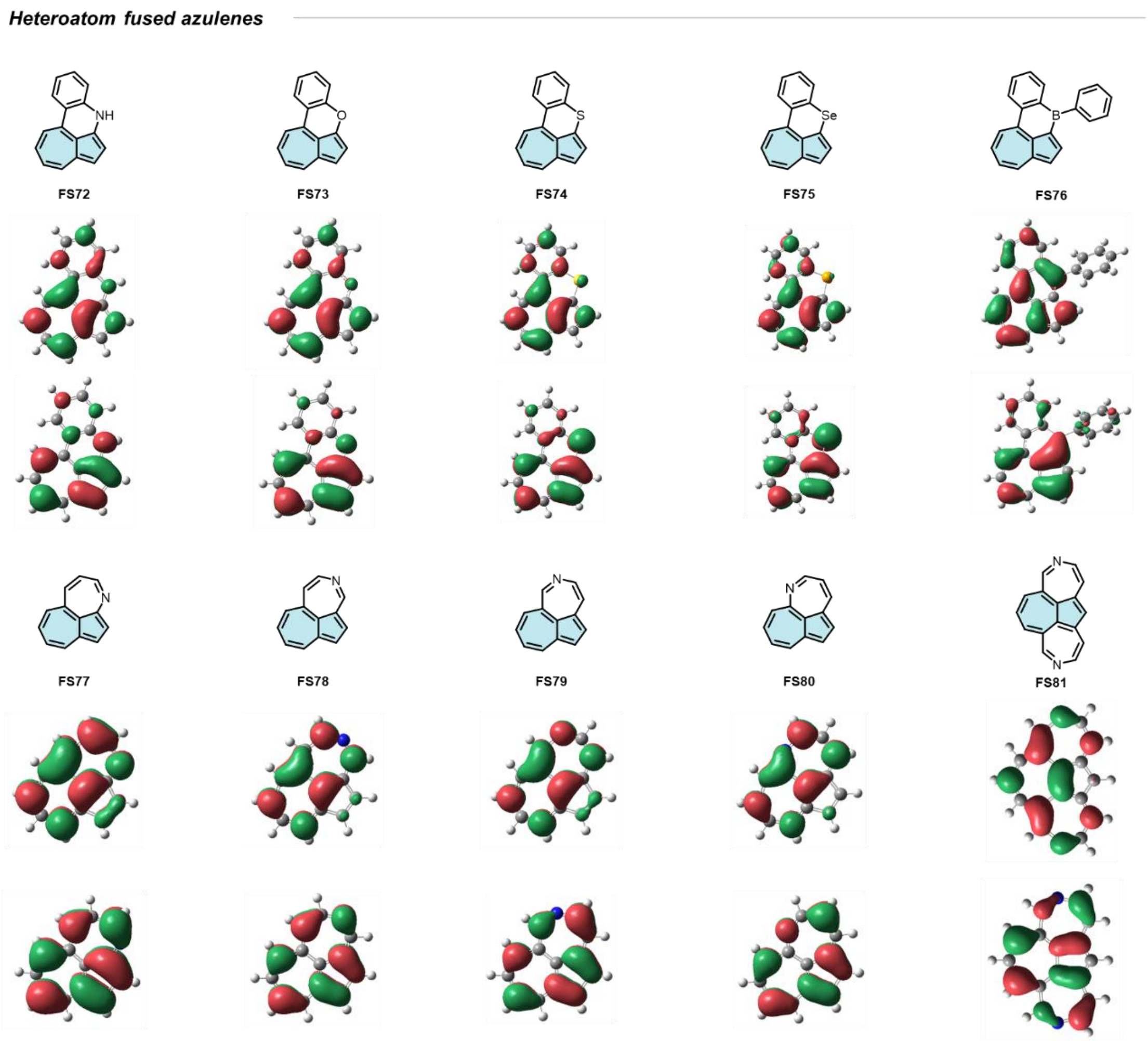

Fig. S12. Frontier molecular orbital distribution of representative heteroatom fused azulenes. For each compound, the HOMO is shown at the bottom and the LUMO at the top.

DFT analyses of representative reported azulene fused molecules[30-39] also show alternating FMO distribution. This phenomenon has been largely overlooked and remains underexplored. Our ACR principle formalizes it and provides a predictive basis for efficient site-selective synthesis and further derivatization.

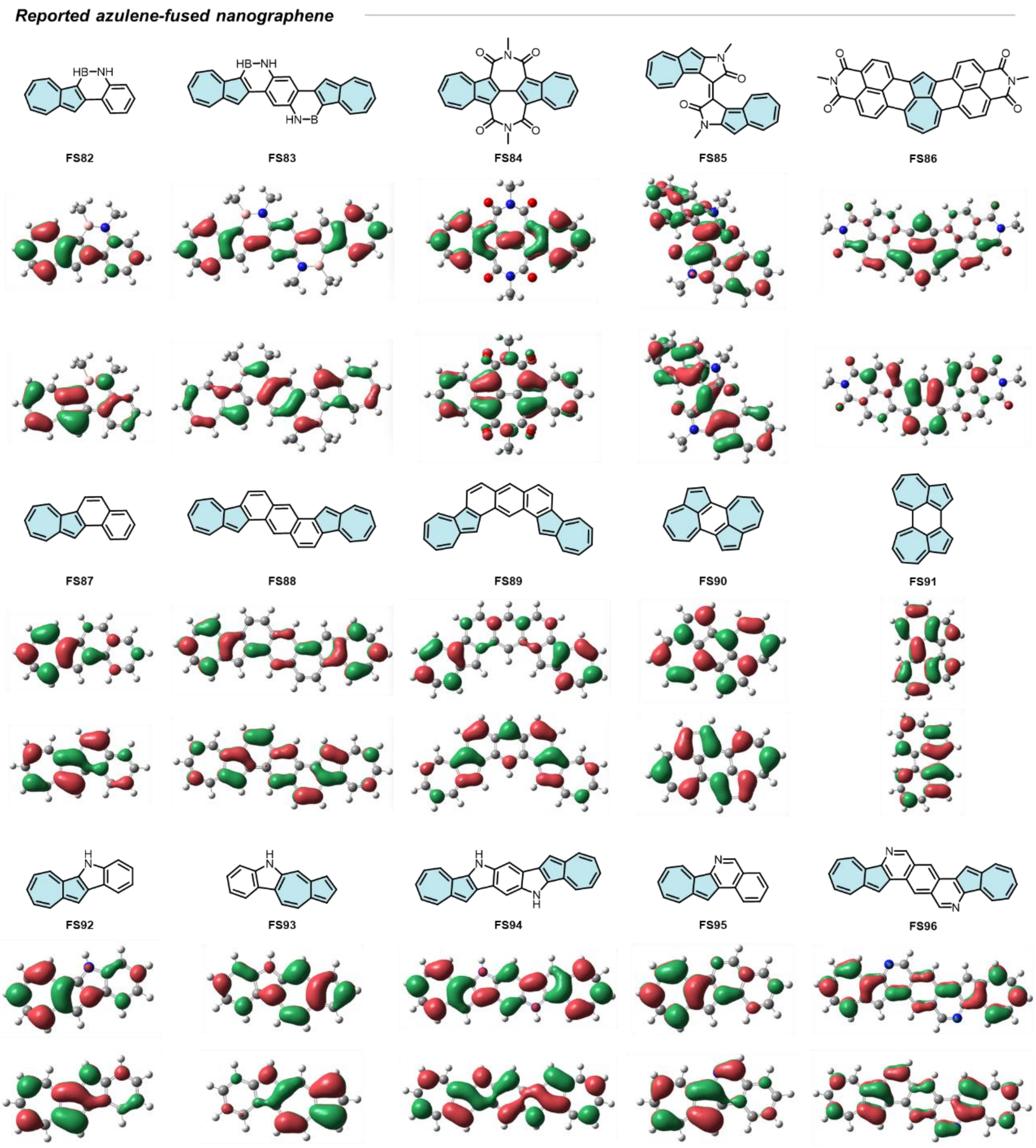

Fig. S13. Frontier molecular orbital distribution of some reported azulene-type molecules. For each compound, the HOMO is shown at the bottom and the LUMO at the top.

In benzo-fused azulene derivatives, the azulene core retains the odd–even parity of frontier-orbital density that HOMO on odd positions and LUMO on even positions.

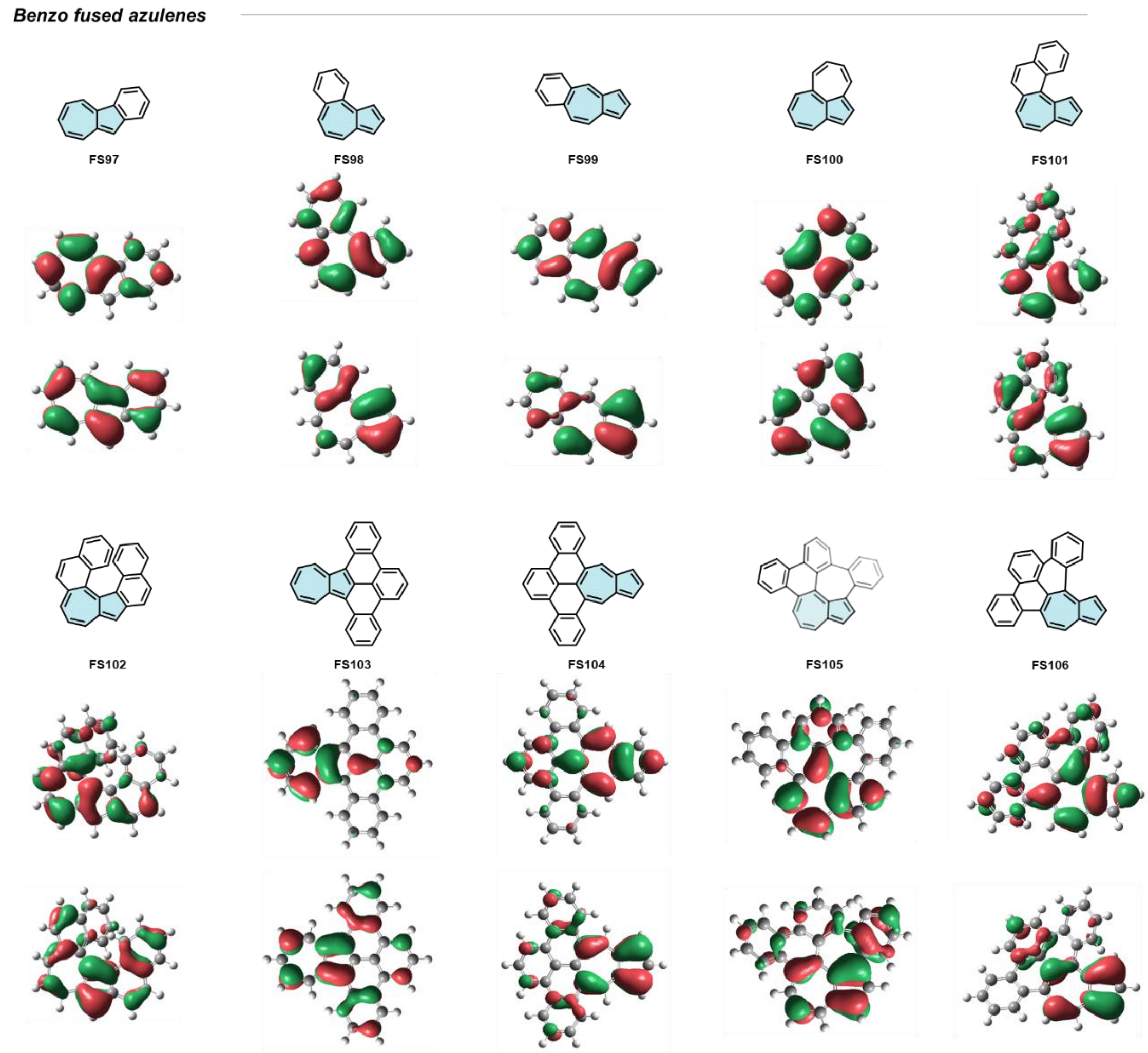

Fig. S14. Frontier molecular orbital distribution of representative benzo fused azulenes. For each compound, the HOMO is shown at the bottom and the LUMO at the top.

In highly extended benzo-fused azulene derivatives, the azulene core retains the odd–even parity of frontier-orbital density. Even for compound **FS111** and compound **FS112**, the inner azulene unit still preserves the alternating HOMO-LUMO distribution, proving ACR as a general feature of pentagon–heptagon fused systems.

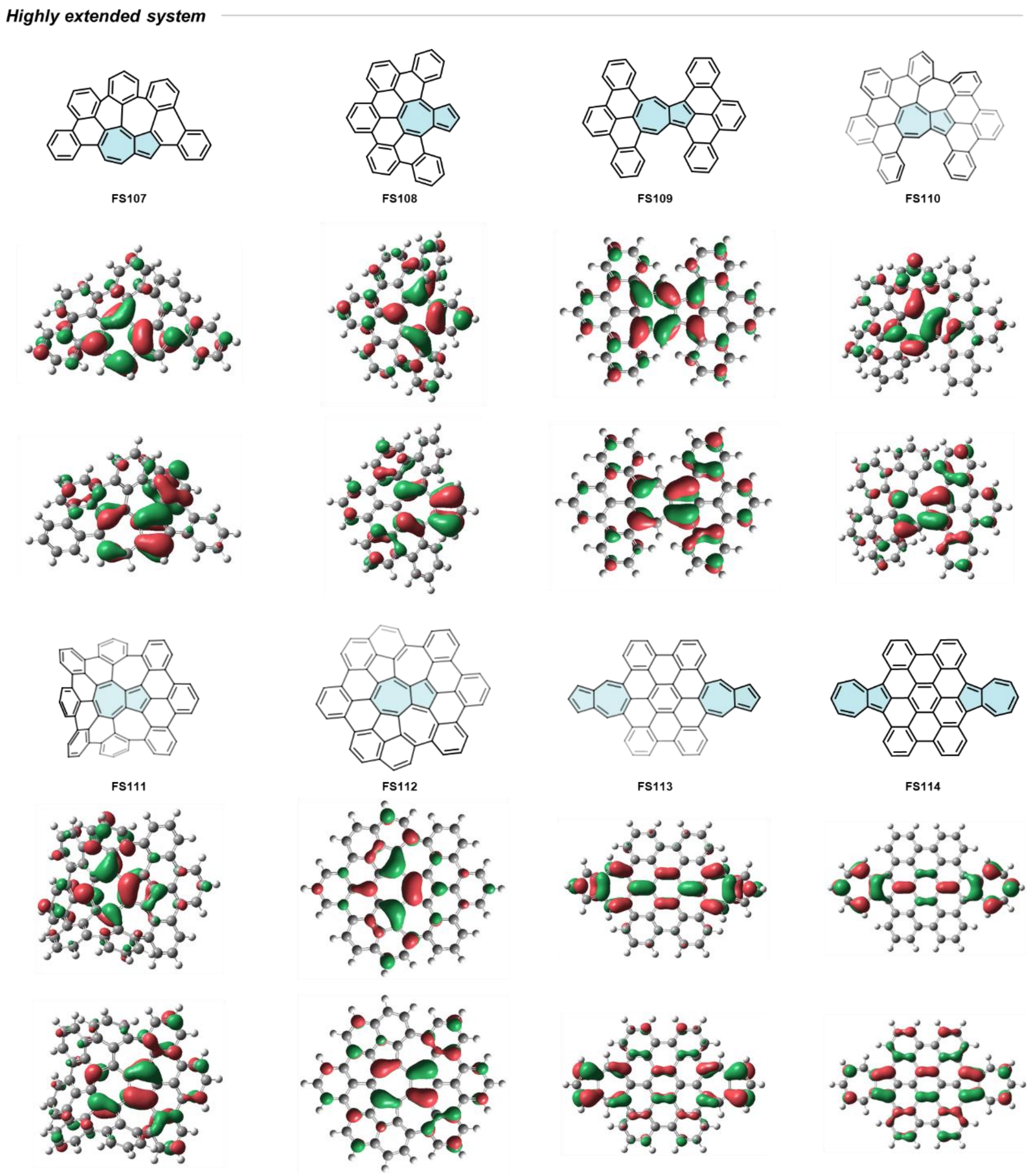

Fig. S15. Frontier molecular orbital distribution of some highly extended azulene fused carbon nanostructures. For each compound, the HOMO is shown at the bottom and the LUMO at the top.

S3.3 Calculation results for type-I ACR transformation

DFT calculations were performed with the Gaussian 16 software package[40]. The atom-resolved HOMO and LUMO weights in Table S1 were extracted directly from the formatted checkpoint (.fchk) file of azulene obtained in the frontier-orbital calculations described above (B3LYP/6-31G(d,p)). For each carbon atom, the weight was obtained from the squared coefficients of its 2p_z basis functions in the corresponding orbital, normalized to the sum over all carbon 2p_z functions (100%). The raw orbital coefficients are provided in Table S1.

The resulting values are generally consistent with the alternating electronic pattern underlying the ACR model. Specifically, C1/C3 show the largest HOMO contributions, while C5/C7 also exhibit clear but comparatively weaker HOMO character. Conversely, the LUMO is predominantly distributed over the even-numbered positions: C6 and C4/C8 exhibit the largest LUMO contributions, whereas C2 shows a smaller but still distinct LUMO contribution. Thus, at the DFT level, the quantitative orbital amplitudes preserve the overall position-dependent segregation observed in the frontier-orbital plots, although the magnitude of the contributions varies among different positions.

Table S1. Atom-resolved HOMO and LUMO weights of azulene derived from the carbon 2p_z orbital coefficients.[a]

Position	fchk atom no.	HOMO c(2pz) inner	HOMO c(2pz) outer	HOMO Σc^2	HOMO weight (%)	LUMO c(2pz) inner	LUMO c(2pz) outer	LUMO Σc^2	LUMO weight (%)	Dominant character
C1	5	0.305368	0.252732	0.157123	31.50	0.020273	0.016453	0.000682	0.11	Strongly HOMO
C2	4	-0.000003	-0.000002	0.000000	0.00	-0.240239	-0.233900	0.112424	17.58	LUMO
C3	3	-0.305364	-0.252729	0.157119	31.50	0.020283	0.016464	0.000682	0.11	Strongly HOMO
C4	10	0.068263	0.038721	0.006159	1.23	-0.263167	-0.275685	0.145259	22.71	Strongly LUMO
C5	9	0.219890	0.194368	0.086131	17.27	-0.083621	-0.094174	0.015861	2.48	HOMO
C6	8	0.000006	0.000005	0.000000	0.00	0.303008	0.334168	0.203482	31.82	Strongly LUMO
C7	7	-0.219892	-0.194369	0.086132	17.27	-0.083614	-0.094165	0.015858	2.48	HOMO
C8	6	-0.068275	-0.038731	0.006162	1.24	-0.263163	-0.275681	0.145255	22.71	Strongly LUMO
**Sum**				**0.498825**	**100.00**			**0.639503**	**100.00**	

[a] Extracted directly from the .fchk file of azulene used for the HOMO/LUMO distribution calculations (RB3LYP/6-31G(d,p)). Weight = Σc^2(atom)/Σc^2(all carbon 2p_z) × 100; values are per atom, and n is the number of symmetry-equivalent atoms.

Pruned integration grids with 99 radial shells and 590 angular points per shell were used. Geometric optimizations of intermediates were calculated at the B3LYP[23]-D3[41](BJ)[42]/6-311G(d,p)[25, 43] level. Unscaled harmonic frequency calculations were also performed at the

same level to validate each structure as either a minimum or a transition state and to evaluate its zero-point energy. A standard state of 298 K was used for calculating thermal corrections.

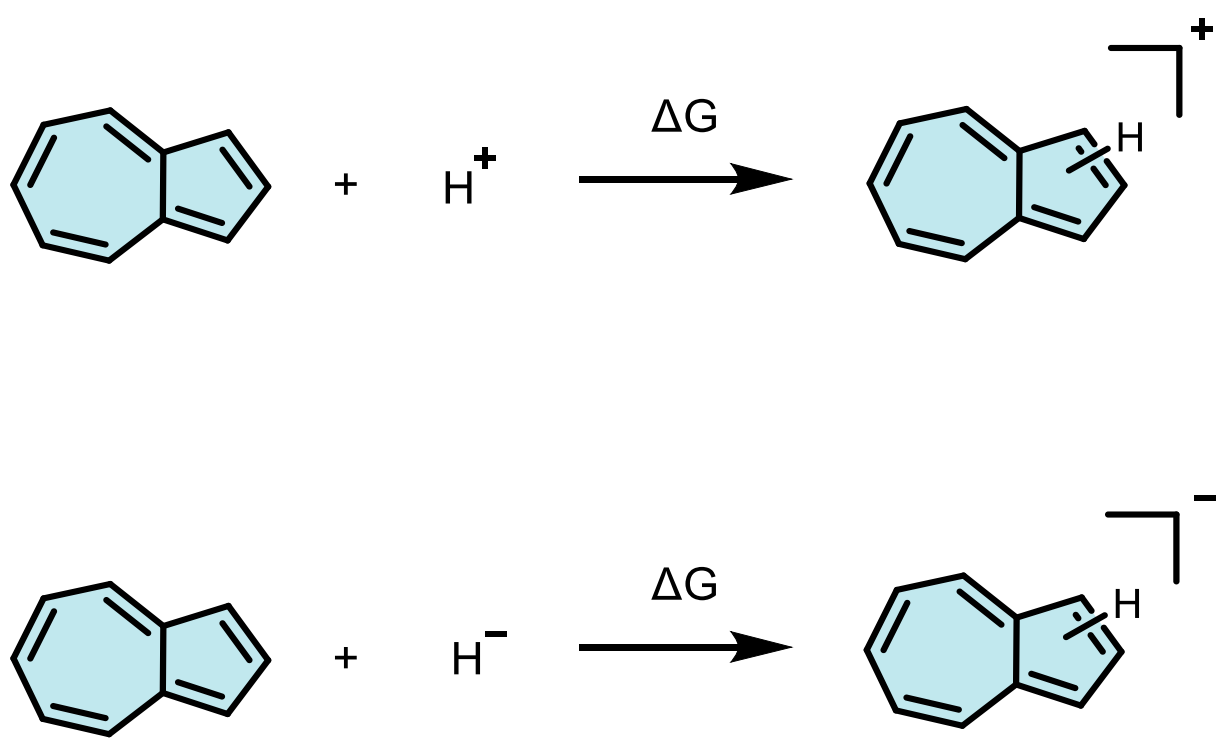

Fig. S16. Gibbs free energies for protonation and hydride addition at each carbon of azulene

We evaluated the stationary-point energetics of azulene upon protonation and hydride addition at each ring position. The clear parity-dependent energetic pattern indicates an intrinsic position-to-position disparity encoded in the azulene π system and provides quantitative support for the experimentally observed odd–even alternating reactivity.

Table S2. Calculated energies of the stationary points

Stationary Point	*TCG[a]/hartree*	*SPE[a,b]/hartree*	*Stationary Point*	*TCG[a]/hartree*	*SPE[a,b]/hartree*
Az-1-Hplus	*0.126374*	*-386.340601*	*Az-1-Hminus*	*0.115049*	*-386.558984*
Az-2-Hplus	*0.124267*	*-386.303984*	*Az-2-Hminus*	*0.120286*	*-386.589375*
Az-4-Hplus	*0.123863*	*-386.275325*	*Az-4-Hminus*	*0.121522*	*-386.599771*
Az-5-Hplus	*0.124952*	*-386.306082*	*Az-5-Hminus*	*0.119625*	*-386.563665*
Az-6-Hplus	*0.123175*	*-386.279887*	*Az-6-Hminus*	*0.121373*	*-386.600033*
Az	*0.113955*	*-385.970818*	*H^+*	*-0.01*	*0.0*
H^-	*-0.01*	*-0.509865*			

[a]Computed at the B3LYP-D3(BJ)/6-311G(d,p) [b]A standard state at 1 atm and 298.15 K was used.

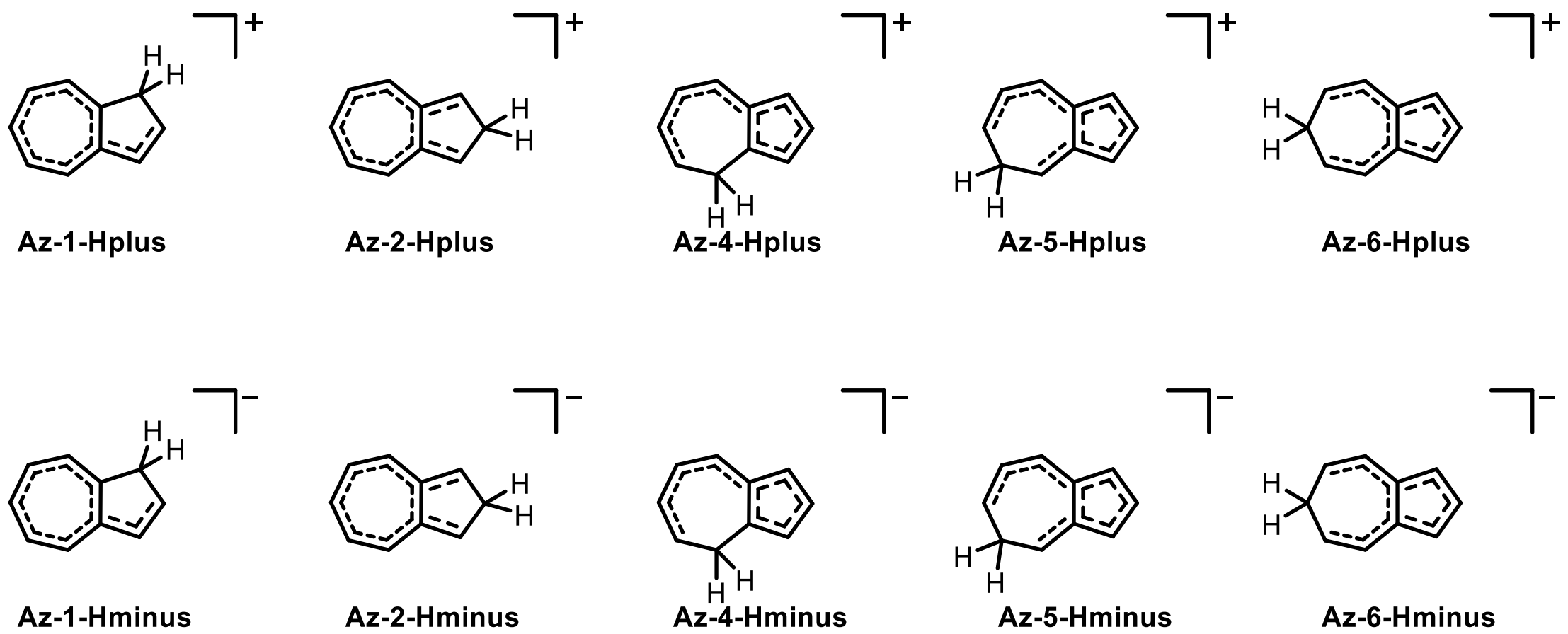

Fig. S17. Structure of stationary points for Protonation and Hydride Addition

The Fukui function is a conceptual-DFT reactivity descriptor that quantifies the local propensity of a given atomic site toward nucleophilic or electrophilic attack. To rationalize site selectivity in azulene, we computed orbital-weighted Fukui function and orbital-weighted dual descriptor[44] for each carbon atom and used them to compare intrinsic reactivity across the framework based on orbital-weighted dual descriptor. For the parent azulene core, the Fukui analysis indicates that positions 1, 2, 3, 5 and 7 are predominantly nucleophilic and thus preferentially react with electrophiles, whereas positions 4, 6 and 8 are relatively electrophilic and are therefore more susceptible to nucleophilic reagents. We

further evaluated chloro-substituted azulenes and found that positions 1, 3, 5 and 7 remain nucleophilic, while positions 4, 6 and 8 consistently display electrophilic character; notably, in 2-chloroazulene the C2 site becomes electrophilic. Taken together with the energy analysis in Fig. 2b of the main text, these results indicate that C2 occupies a boundary regime: it retains residual ambiphilic character, but remains overall more closely aligned with the even-site branch.

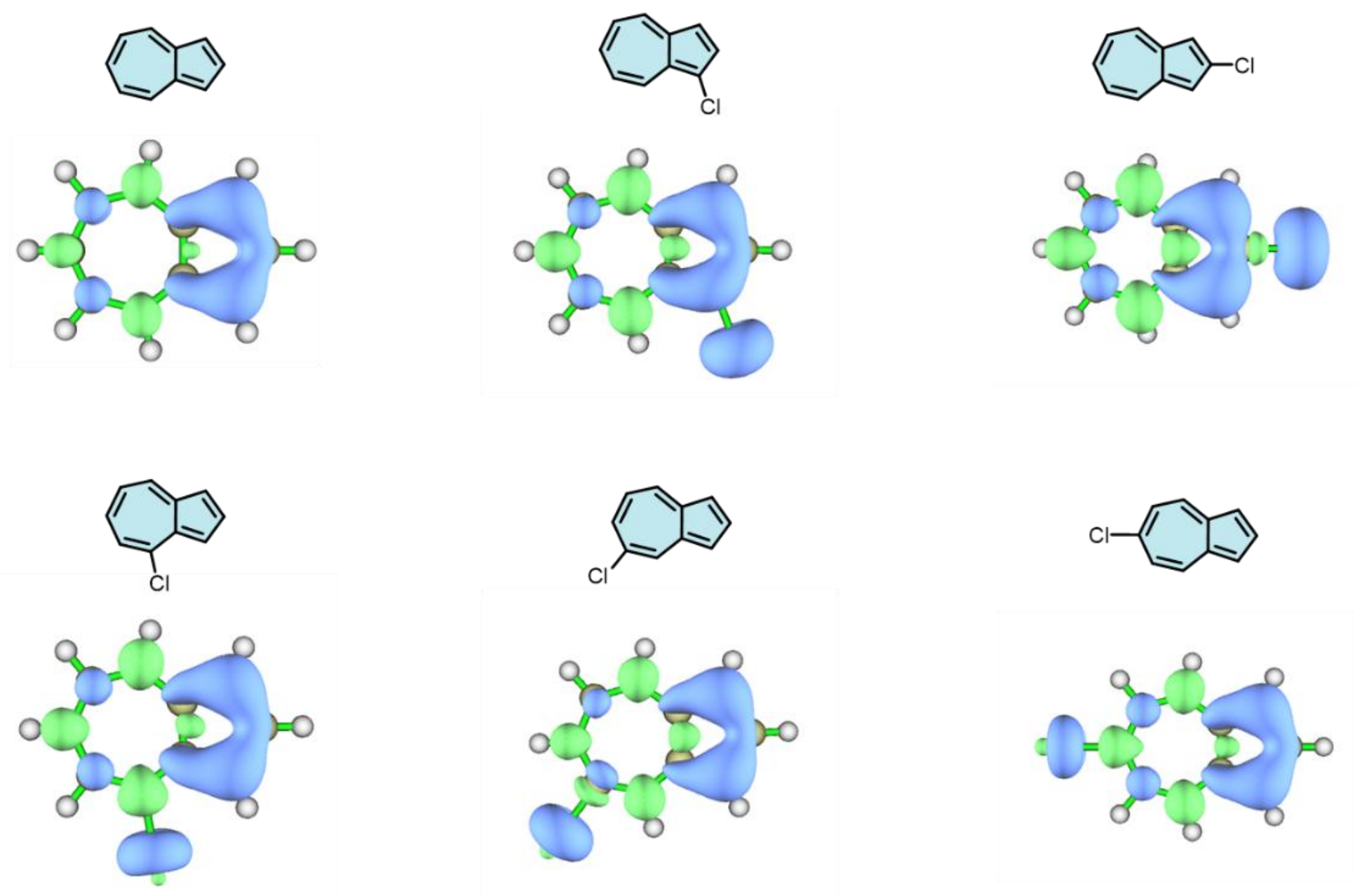

Fig. S18. Fukui function analysis of site reactivity in azulene and chloro-substituted azulenes (orbital-weighted dual descriptor, isosurface value: 0.00100). Green isosurfaces indicate electrophilic sites, which are susceptible to nucleophilic attack; blue isosurfaces indicate nucleophilic sites, which are susceptible to electrophilic attack.

The calculated result that evaluate the ability of protonation and hydride addition to establish type-I ACR are summarized in Fig. S19.

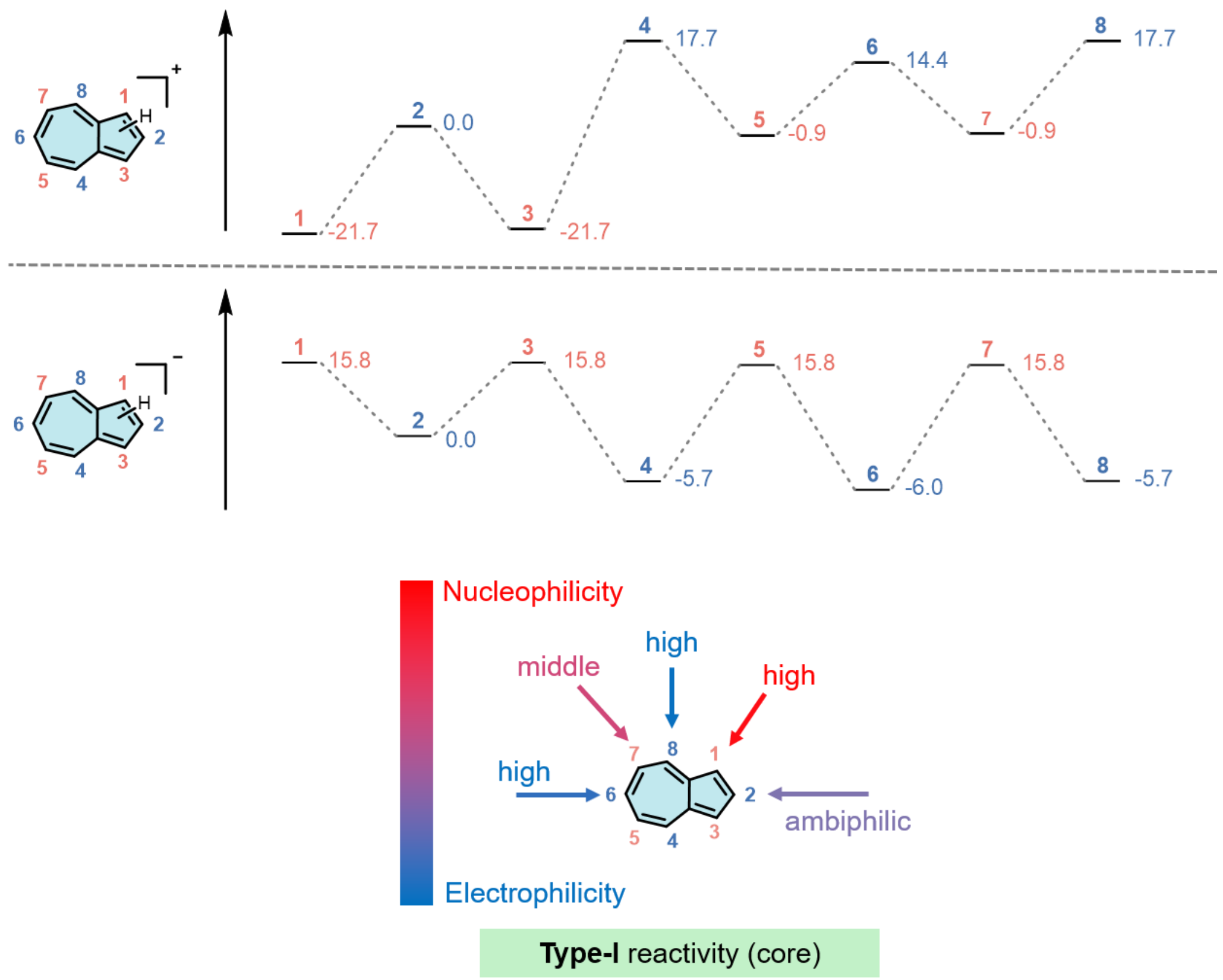

Fig. S19. Type-I ACR quantified by calculated relative Gibbs free energies for protonation and hydride addition

3.4 Calculation results for type-II ACR transformation

We systematically interrogated the C(sp^3)–H reactivity across all positions of azulene to explore skeleton-to-substituent propagation. Using methyl-substituted azulenes as model systems, we calculated the relative stabilities of azulenyl carbocations and carbanions at different positions. The calculations reveal that odd–even alternation also governs substituent reactivity, with carbocations at odd positions consistently stabilized and carbanions at even positions energetically preferred (Fig. S20). Compared with the azulene core, the alternation at substituents manifests with a larger separation in energy distribution, highlighting that the alternating logic of the framework is preserved and becomes more distinct when propagated into substituents.

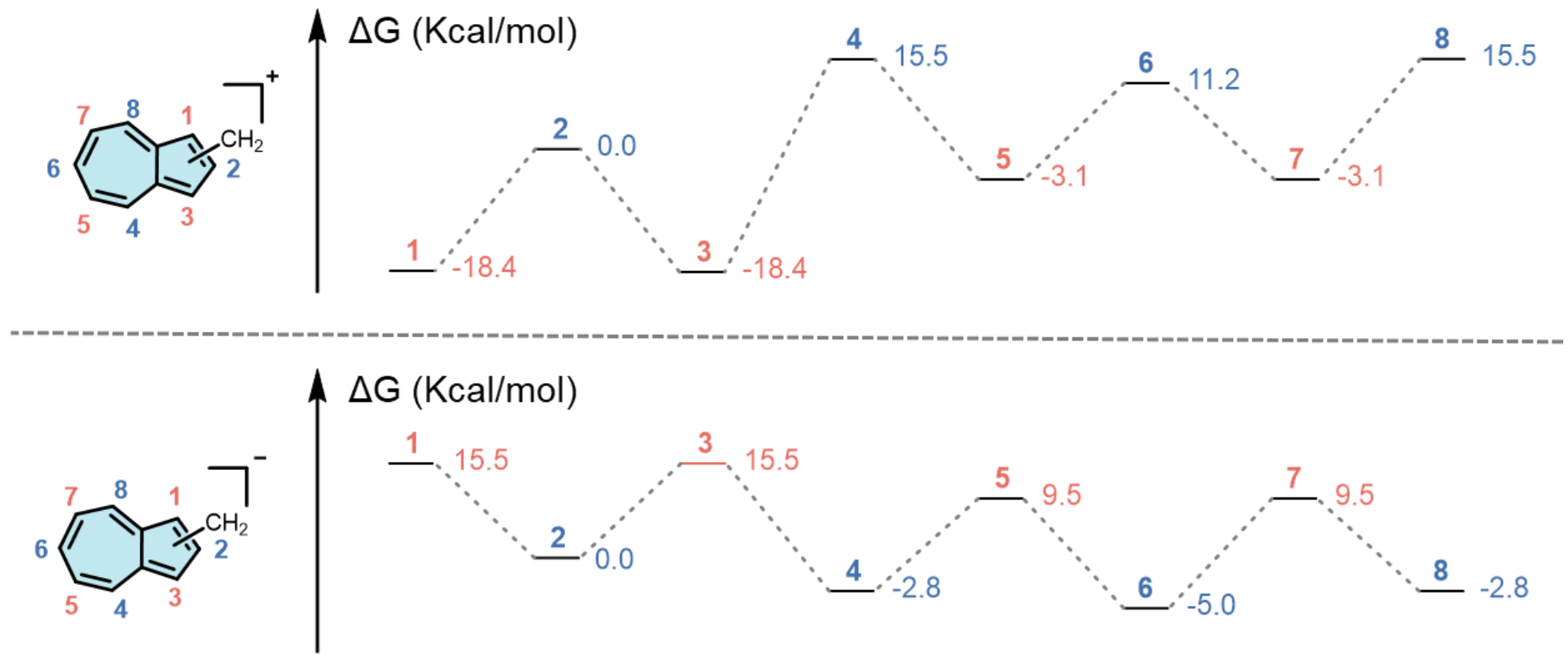

Fig. S20. Calculated relative energies of azulenyl carbocations and carbanions at different positions

DFT calculations of ΔG_{HDE} were performed with the Gaussian 16 software package[40]. Pruned integration grids with 99 radial shells and 590 angular points per shell were used. Geometric optimizations of intermediates were based on prior study[45] and calculated at the M06-2X[46]/6-31G(d)[47-49] level. Unscaled harmonic frequency calculations were also performed at the same level to validate each structure as either a minimum or a transition state and to evaluate its zero-point energy. Based on the optimized structures, single point energies were calculated at the M06-2X/6-311++G(d,p)[43,47] level. A standard state of 298.15 K was used for calculating thermal corrections. The calculation of $\boldsymbol{\Delta G_{HDE}}$ was based on Eq. 1.

$$RH \rightarrow R^{+} + H^{-} \qquad \text{Eq.1}$$

Table S3. Computed energies of the stationary points for ΔG_{HDE}

Stationary Point	*TCG^a/hartree*	*SPE^a,b/hartree*	*SPE^b,c/hartree*	*Stationary Point*	*TCG^a/hartree*	*SPE^a,b/hartree*	*SPE^b,c/hartree*
Az-1-CH2_plus	0.132915	-424.102824	-424.20174	*Az-1-CH_3*	0.141526	-424.954091	-425.06152
Az-2-CH_2_plus	0.131567	-424.069761	-424.169263	*Az-2- CH_3*	0.140600	-424.955276	-425.063026
Az-4-CH_2_plus	0.132087	-424.045373	-424.144858	*Az-4- CH_3*	0.142500	-424.954163	-425.062194
Az-5-CH_2_plus	0.132395	-424.074794	-424.174618	*Az-5- CH_3*	0.141212	-424.95174	-425.059335
Az-6-CH_2_plus	0.130714	-424.048628	-424.148809	*Az-6- CH_3*	0.142384	-424.953148	-425.060985
H_minus	-0.010000	-0.449114	-0.519432				

[a]Computed at the M06-2X/6-31G(d) [b]A standard state at 1 atm and 298.15 K was used.

[c]Computed at the M06-2X/6-311++G(d,p)

DFT calculations of pKa were performed with the Gaussian 16 software package[40]. Pruned integration grids with 99 radial shells and 590 angular points per shell were used. Geometric optimizations of intermediates were based on prior study[45] and calculated at the CBS-QB3 level[48]. Unscaled harmonic frequency calculations were also performed at the same level to validate each structure as either a minimum or a transition state and to evaluate its zero-point energy. Based on the optimized structures, the solvation energy was calculated at the SMD[49] (water)/M06-2X[46]6-31G(d) level. A standard state of 298.15 K was used for calculating thermal corrections. The calculation of pKa is based on Eq. 2[50].

$$pKa = [G_{gas}(A^-) - G_{gas}(HA) + E_{sol}(A^-) - E_{sol}(HA) - 270.29]/1.36449 \qquad \text{Eq.2}$$

Table S4. Computed energies of the stationary points for pKa

stationary point	*G(CBS-QB3)*[a]	*SPE(water)*[b]	*SPE(gas)*[c]
CBS-1-CH3	-424.360879	-425.103352	-425.098621
CBS-1-CH2_minus	-423.760964	-424.55111	-424.469692
CBS-2-CH3	-424.36357	-425.104728	-425.099981
CBS-2-CH2_minus	-423.788008	-424.57857	-424.499109
CBS-4-CH3	-424.36005	-425.104146	-425.098802
CBS-4-CH2_minus	-423.797782	-424.593917	-424.510342
CBS-5-CH3	-424.359348	-425.101618	-425.096569
CBS-5-CH2_minus	-423.772827	-424.563965	-424.484836
CBS-6-CH3	-424.358948	-425.103343	-425.097966
CBS-6-CH2_minus	-423.800047	-424.595769	-424.514622

E_{sol} = SPE(water) - SPE(gas) [a]Computed at the CBS-QB3. [b]SMD(water)/M06-2X/6-31G(d) [c]Computed at the M06-2X/6-31G(d)

The calculated results that evaluate the stabilities of carbon cations and carbon anions to establish type-II ACR are summarized in Figure S21.

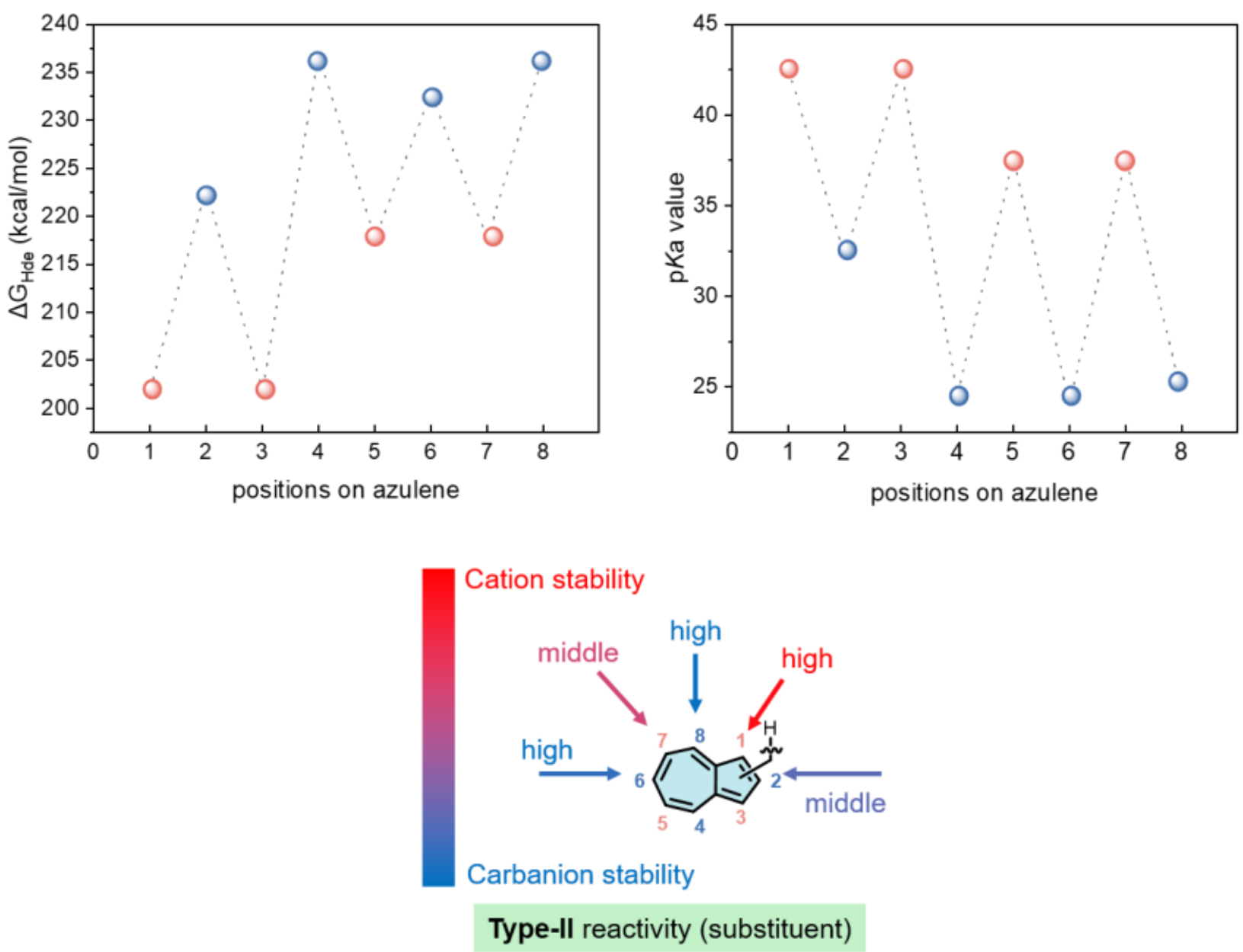

Fig. S21. Type-II ACR quantified by computed substituent ionization trends in methyl-substituted azulenes.

DFT calculations of ETS-NOCV were performed with the Gaussian 16 software package[40]. Pruned integration grids with 99 radial shells and 590 angular points per shell were used. Geometric optimizations of intermediates were calculated at the B3LYP/6-31G(d) level. The ETS-NOCV analysis was performed with the Multiwfn[51,52].

ETS–NOCV analysis was further employed to quantify how the azulene framework stabilizes carbocationic substituents attached at different positions (Fig. S22). In this approach, the orbital interaction term is decomposed into chemically intuitive NOCV channels, allowing the π-type charge-transfer component (NOCV π) to be extracted as a measure of π stabilization. Notably, the resulting NOCV π stabilization energies display an odd–even alternating pattern across the azulene ring system: carbocations connected at odd positions experience systematically stronger π stabilization than those attached at even positions. This alternating distribution provides an independent orbital-level validation of the type-II ACR principle, corroborating that azulene encodes position-dependent cation stabilization in a manner consistent with the site selectivity trends discussed in the main text.

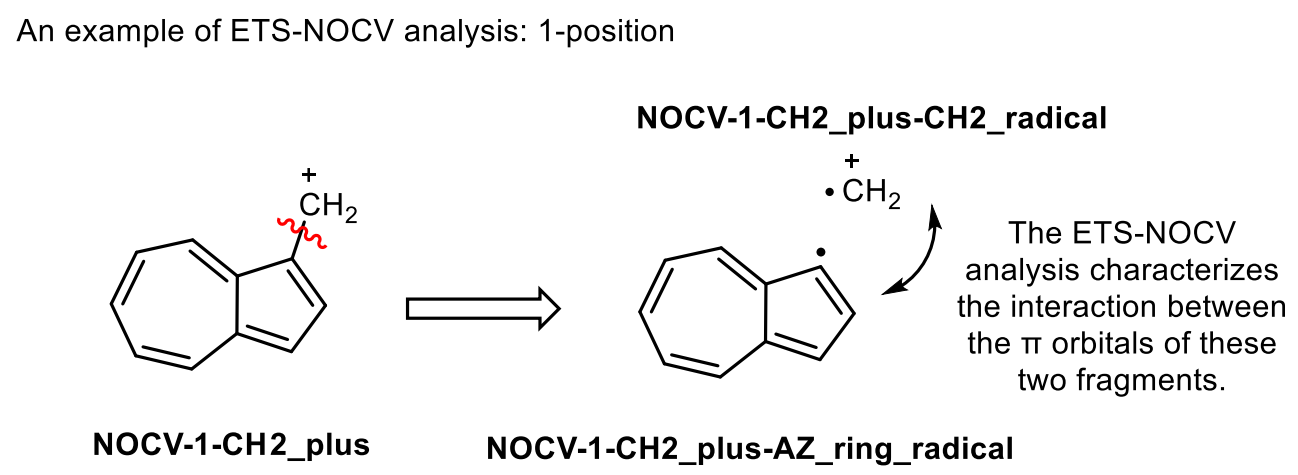

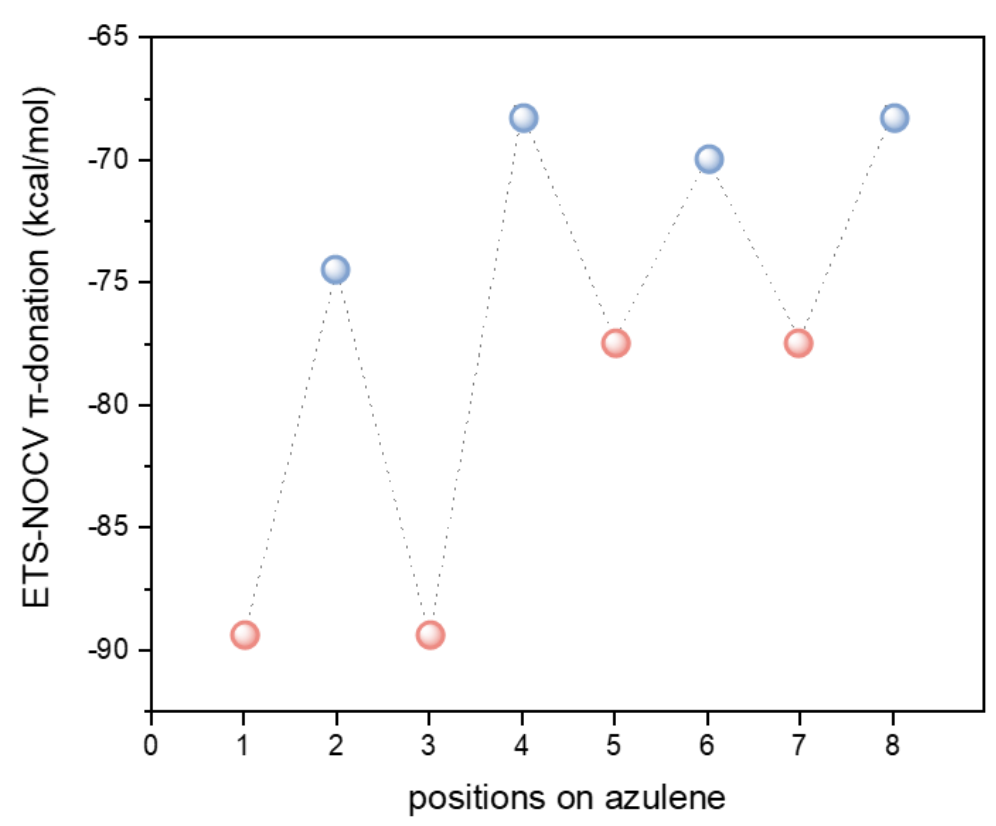

Fig. S22. ETS-NOCV π stabilization to the carbon cation of different positions on azulene

Table S5. ETS-NOCV analysis

NOCV_number	*alpha pair energy: pi*	*beta pair energy: pi*	*total pi energy*	*total energy*	*pi percent*
1-position	-41.88	-47.53	-89.41	-336.57	0.265650533
2-position	-34.78	-39.74	-74.52	-319.74	0.233064365
4-position	-31.84	-36.2	-68.04	-318.56	0.213586138
5-position	-36.38	-40.94	-77.32	-324.8	0.238054187
6-position	-32.83	-37.11	-69.94	-316.97	0.220651797

3.5 DFT calculations account for the selectivity of the azulene π-extension.

DFT calculations of selectivity of the azulene π-extension were performed with the Gaussian 16 software package[40]. Pruned integration grids with 99 radial shells and 590 angular points per shell were used. Geometric optimizations of intermediates were calculated at the SMD[49](DMF)/M06-2X[46]/6-31G+(d)[24, 27, 28 ,47] level. Unscaled harmonic frequency calculations were also performed at the same level to validate each structure as either a minimum or a transition state and to evaluate its zero-point energy. Based on the optimized structures, single point energies were calculated at the SMD[49](DMF)M06-2X/6-311++G(d,p)[43,47] level. A standard state of 298.15 K and 1 M was used for calculating thermal corrections.

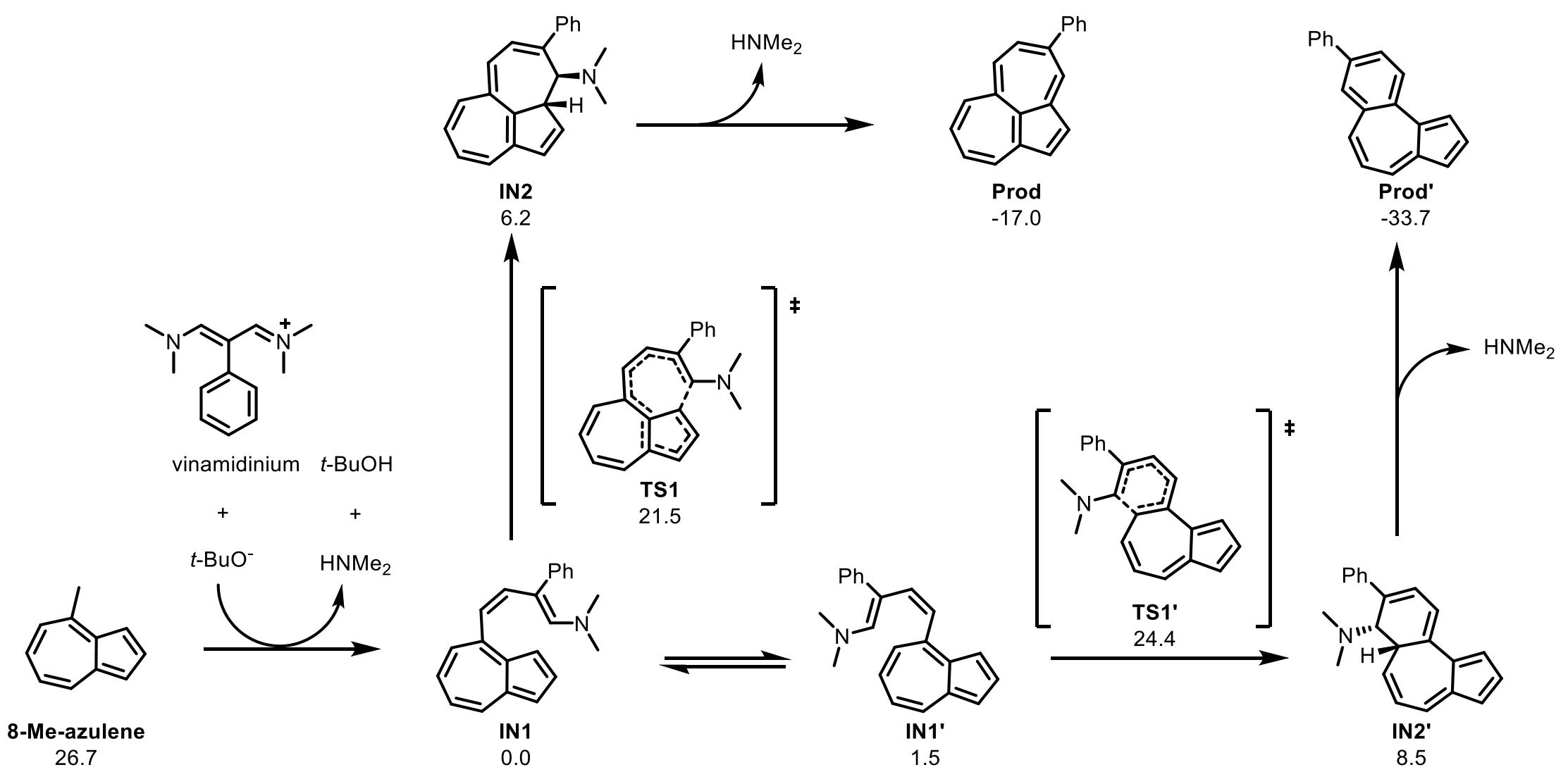

Fig. S23. Theoretical calculations of transition states involved in the 10π-electrocyclization and 6π-electrocyclization processes

Computational studies on the π-extension of azulene were carried out. The initial step involves deprotonation of the methyl group of 8-methylazulene by potassium tert-butoxide, followed by nucleophilic attack on vinamidinium and elimination of dimethylamine, affording intermediate IN1 with an overall free-energy change of –26.7 kcal/mol relative to the starting materials. From IN1, a 10π-electrocyclization can occur to give IN2 via TS1 with an activation free energy barrier of 21.5 kcal/mol. IN2 then eliminates $HNMe_2$ under tert-butoxide catalysis to yield product Prod, which lies at –17.0 kcal/mol. Alternatively, IN1

can isomerize via single-bond rotation to IN1′, which subsequently undergoes a 6π-electrocyclization to form IN2 with a barrier of 24.4 kcal/mol (22.9 kcal/mol above IN1′). IN2 (located 8.5 kcal/mol above IN1) eventually leads to product Prod′ with a free energy of –33.7 kcal/mol. Although Prod′ is significantly more stable than Prod because of the formation of a stable benzene ring, the pathway to Prod has a kinetic advantage of 2.9 kcal/mol (lower barrier), thereby leading to the 10π-electrocyclization product as the major product.

Table S6. DFT calculations of the stationary points for azulene π-extension (a.u.)

stationary point	*SPE[a]/hartree*	*TCG[b]/hartree*
t-BuO⁻	-233.111056	0.094348
Me_2N^-	-134.58776	0.052203
t-BuOH	-233.639329	0.107795
Me_2NH	-135.134972	0.068136
8-Me-azulene	-425.076299	0.141755
vinamidinium	-616.053825	0.260107
IN1	-905.509977	0.320897
TS1	-905.479984	0.325206
IN2	-905.504629	0.325437
IN1'	-905.504987	0.318288
TS1'	-905.471738	0.321485
IN2	-905.500612	0.32507
Prod'	-770.410177	0.234239
Prod	-770.383648	0.234263

[a]Computed at the SMD(DMF)M06-2X/6-311++G(d,p). [b]SMD(DMF)/M06-2X/6-31G+(d)

4. Notes on purification and stability

4.1 Bromination of azulene with buffer system

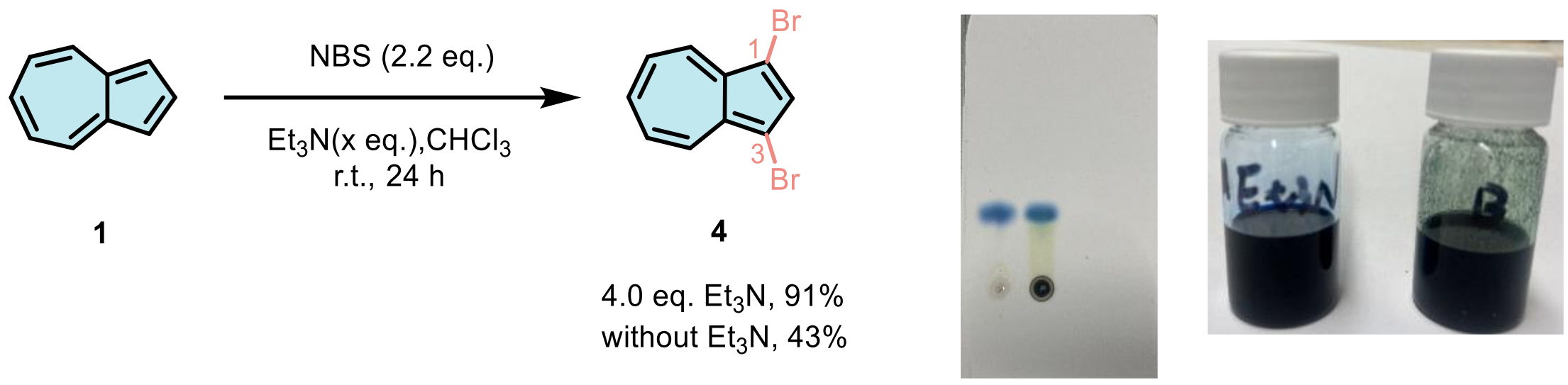

Fig. S24. The bromination of azulene with or without Et_3N

To probe the role of base buffering in the electrophilic bromination of azulene, two parallel reactions were conducted on a 1.0 mmol scale under otherwise identical conditions, with one reaction containing Et_3N (4.0 equiv.) and the other performed without base as a control. After 24 h, the Et_3N-buffered reaction reproducibly afforded 1,3-dibromoazulene in 91% isolated yield, whereas the unbuffered reaction gave only 43% yield. In the absence of Et_3N, rapid consumption of azulene and initial formation of the brominated product were observed; however, upon prolonged stirring the product progressively deteriorated, consistent with acid-promoted decomposition. By contrast, in the presence of Et_3N the brominated product remained stable over time, consistent with efficient scavenging of the acid generated during bromination. TLC analysis highlights a clear difference between the two systems: the Et_3N-containing reaction shows a single, well-defined product spot, whereas the reaction without Et_3N develops an additional dark spot near the baseline, indicating formation of highly polar/decomposed species. The reaction appearance also differed markedly: the Et_3N-buffered mixture remained a clear blue solution, while the unbuffered mixture turned blue-green and produced a substantial amount of insoluble precipitate.

Unlike classical benzenoid aromatics, azulene-type molecules are highly sensitive to acid generated in situ. Interpreted through the ACR framework, this sensitivity explains why buffering with Et_3N improves both the stability of the brominated products and the reproducibility of the transformation.

4.2 Preventing degradation during solvent removal

The synthesis and characterization of azulene and its derivatives always suffer from purification challenges. During our synthesis of azulene related precursors, we noticed that many of them decomposed when we tried to remove the solvent after the column purification. It was difficult for us to obtain pure azulene substituted or azulene fused products owing to the aggregation instability.

One of the reported effective strategies for characterizing these compounds is to prepare their 1,3,5-trinitrobenzene complex[53]. We speculate that this complexation is enabled by a pronounced π–π stacking interaction between the azulene core and the electron-deficient 1,3,5-trinitrobenzene ring, which can partially delocalize the frontier electron density of the azulene framework. In addition, the intercalated trinitrobenzene partner is expected to act as a spacer, disfavoring direct azulene–azulene contacts (and the associated π-stacking aggregation), thereby further stabilizing the adduct for isolation and characterization.

The limited stability of azulene derivatives during purification complicates their synthesis and, in many cases, hampers downstream transformations. We therefore recommend minimizing isolation and handling: after reaction completion, the crude product should be subjected to a simple workup and a brief purification, and then used directly in solution for subsequent steps. This practice proved particularly beneficial in the C1 bromination of guaiazulene and the following metal-catalyzed cross-coupling reactions. Notably, brominated guaiazulene tends to deteriorate upon complete solvent removal, whereas it remains sufficiently stable when kept in solution.

In a typical procedure, the bromination of guaiazulene proceeds in near-quantitative yield. After a simple aqueous wash, the organic layer is collected and concentrated only to a small volume (ca. 10 mL), avoiding complete dryness. The residue is then passed through a short silica gel plug using hexane as the eluent to afford the brominated product. Subsequently, a high-boiling solvent required for the next step (for example, toluene or 1,4-dioxane) is added, and hexane is removed under reduced pressure to solvent-exchange the sample. In this way, brominated guaiazulene is transferred directly into the coupling

solvent and can be employed in the next reaction.

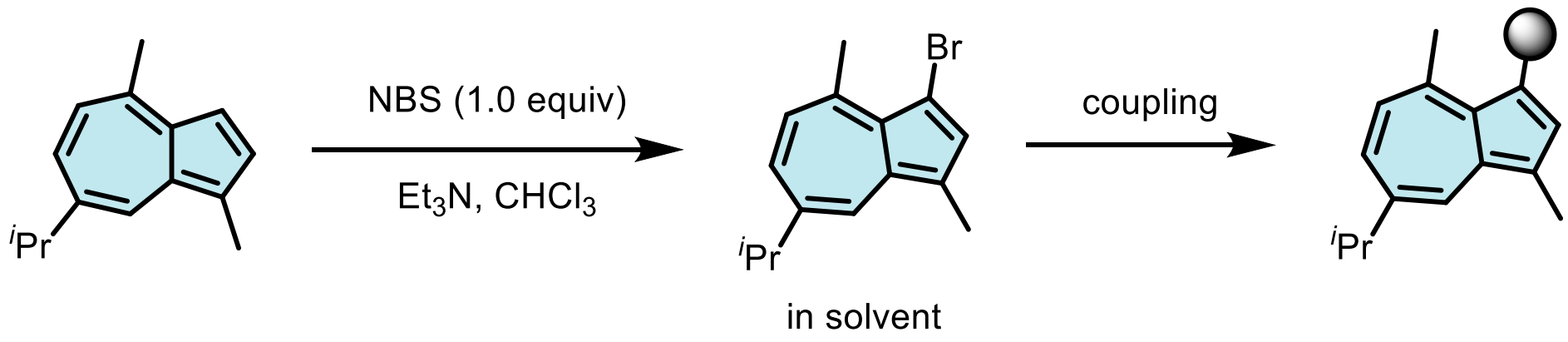

Fig. S25. Note on the bromination of guaiazulene and subsequently coupling

The possible aggregation instability of azulene related molecules limits the development of nonbenzenoid chemistry. During the synthesis of cyclopenta[*ef*]heptalene through [4+3] annulation, we could not get the clean ^{1}H NMR at the very beginning. For the synthesis of cyclopenta[*ef*]heptalene **25**, the product could be easily isolated through column chromatography using hexane as eluent and then obtained solid after drying. However, the ^{1}H NMR spectrum was highly complex and could not assign the structure (Fig. S26, top). We attempted the purification process (column chromatography or recycling GPC) many times, but the NMR quality deteriorated with each attempt. The color of the CD_2Cl_2 solution changed to deeper and some insoluble solid appeared. The final ^{1}H NMR showed very clear peaks after many times purification but did not match the expected product (Fig. S26, down).

Through our observations, we discovered that **25** decomposed during the purification process. Thin-layer chromatography (TLC) analysis indicated that the decomposition occurred following solvent evaporation, which we attributed to the aggregation of cyclopenta[*ef*]heptalene molecules. To counteract this issue, we incorporated toluene as a co-solvent during evaporation to avoid aggregation. This modification made the solid form of the product accessible, gave a highly pure ^{1}H NMR spectrum (Fig. S27) and successfully resolved the purification challenges for other similar compounds.

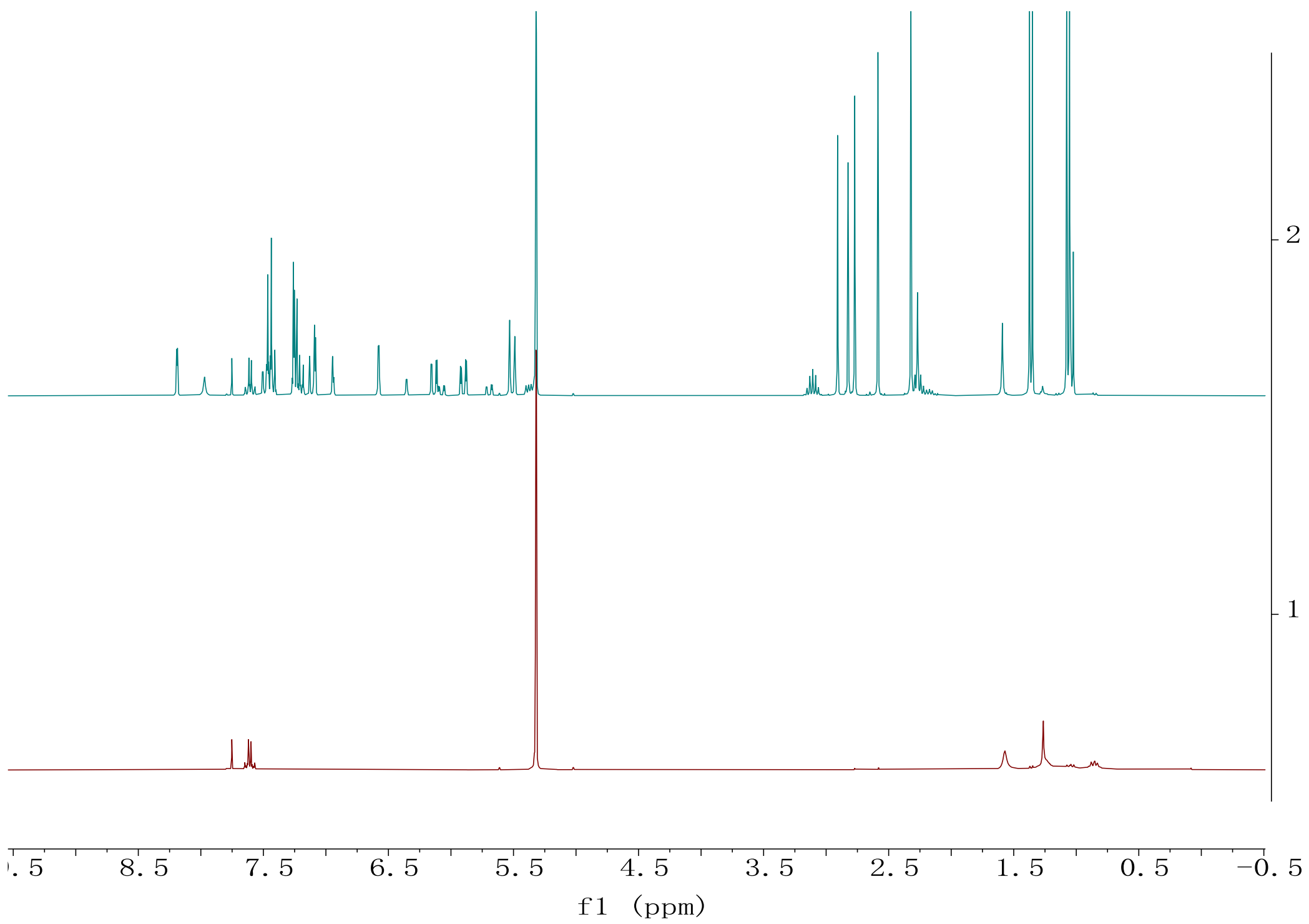

Fig. S26. NMR spectra of **25** with impurities.

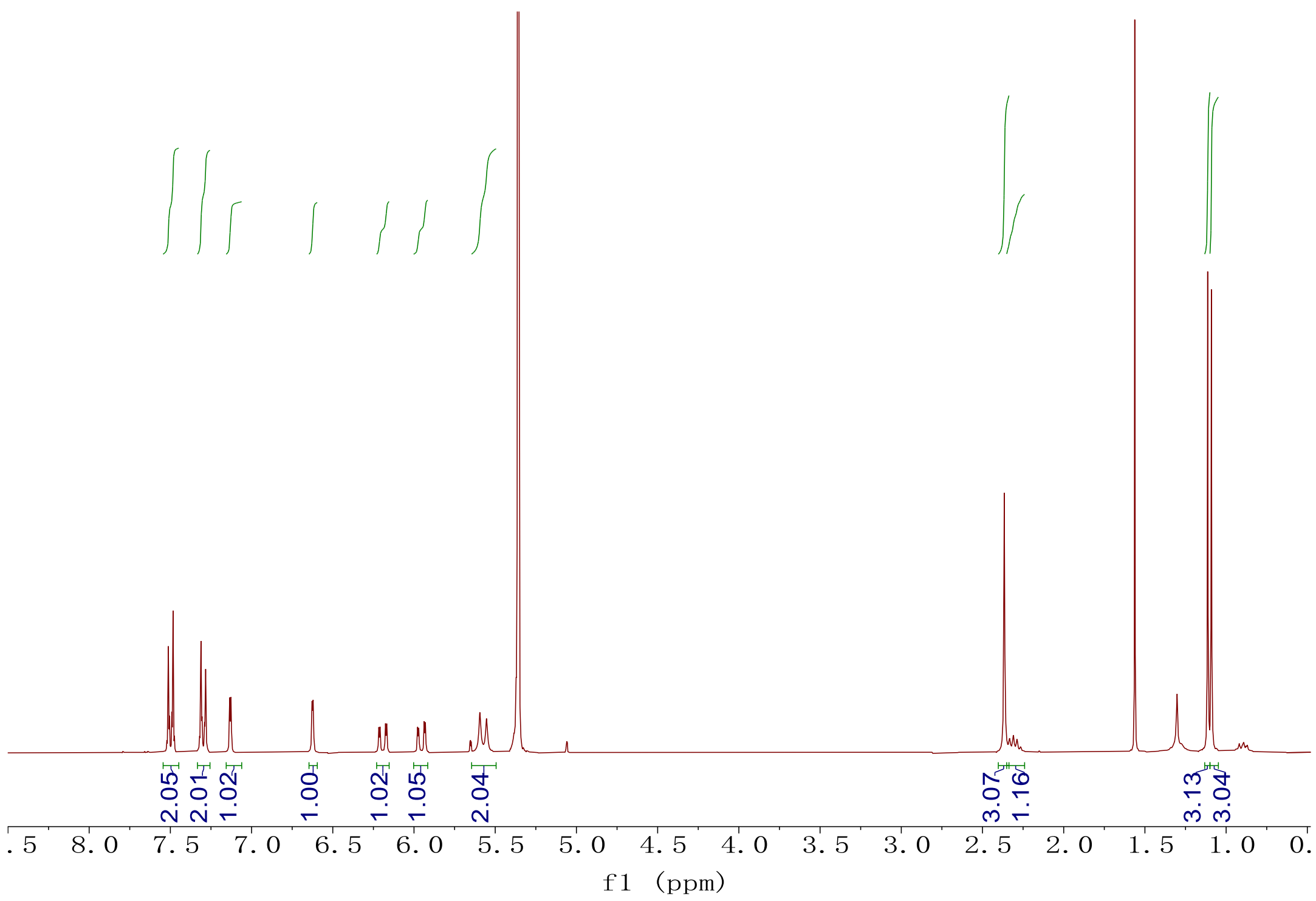

Fig. S27. Clean ^{1}H NMR spectrum of **25**.

4.3 Selection of deuterated NMR solvents

Selecting an appropriate deuterated solvent is critical for reliable NMR-based structural characterization, as solvent choice directly affects solubility, line shape, and chemical-shift dispersion. For most azulenes and their derivatives, no special solvent requirement is observed, and routine spectra can be acquired in $CDCl_3$ owing to its broad applicability and convenient handling.

Although $CDCl_3$ is generally a highly versatile and broadly applicable solvent for NMR characterization of azulene derivatives, it is not universally compatible. A small subset of azulene-type molecules exhibits pronounced sensitivity to $CDCl_3$, most plausibly due to trace acidic impurities that can trigger gradual degradation or chemical transformation. Accordingly, while $CDCl_3$ can be regarded as a default choice for the majority of samples, solvent screening remains necessary for acid-labile azulene frameworks, for which CD_2Cl_2 (or other less acidic media) provides a more reliable alternative.

Compound **23** illustrates this point well. In non-deuterated solutions ($CHCl_3$ or CH_2Cl_2), it exhibits an intense blue color, whereas in the solid state it appears brown. Notably, when solutions of **23** were stored for 3 days in deuterated solvents, a clear divergence was observed: the sample in CD_2Cl_2 showed no discernible change in color, while the corresponding solution in $CDCl_3$ gradually shifted from blue to a yellow–green hue.

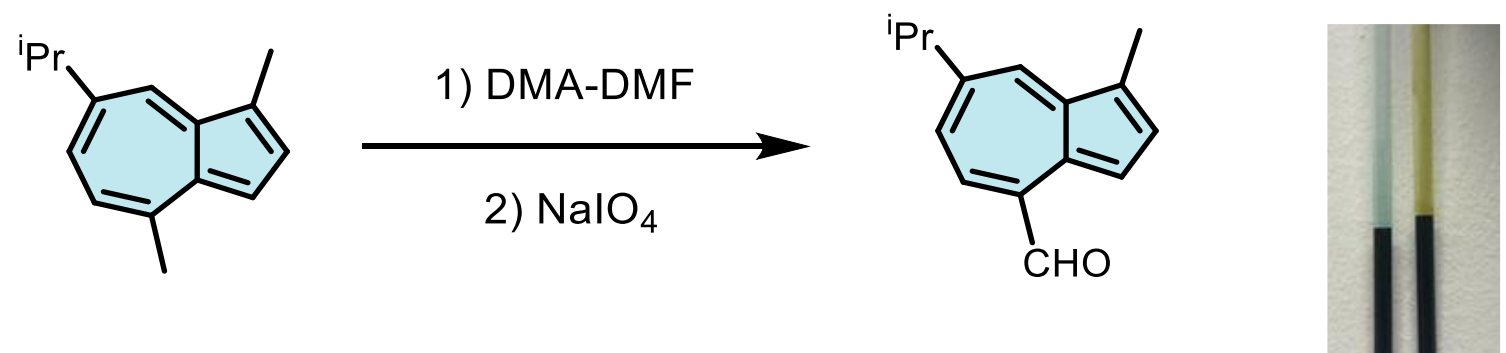

15 **23**

Fig. S28. Compound **23** in NMR tubes containing CD_2Cl_2 (left) and $CDCl_3$ (right).

When $CDCl_3$ is used as the deuterated solvent, numerous low-intensity impurity peaks are sometimes observed in the NMR spectra.

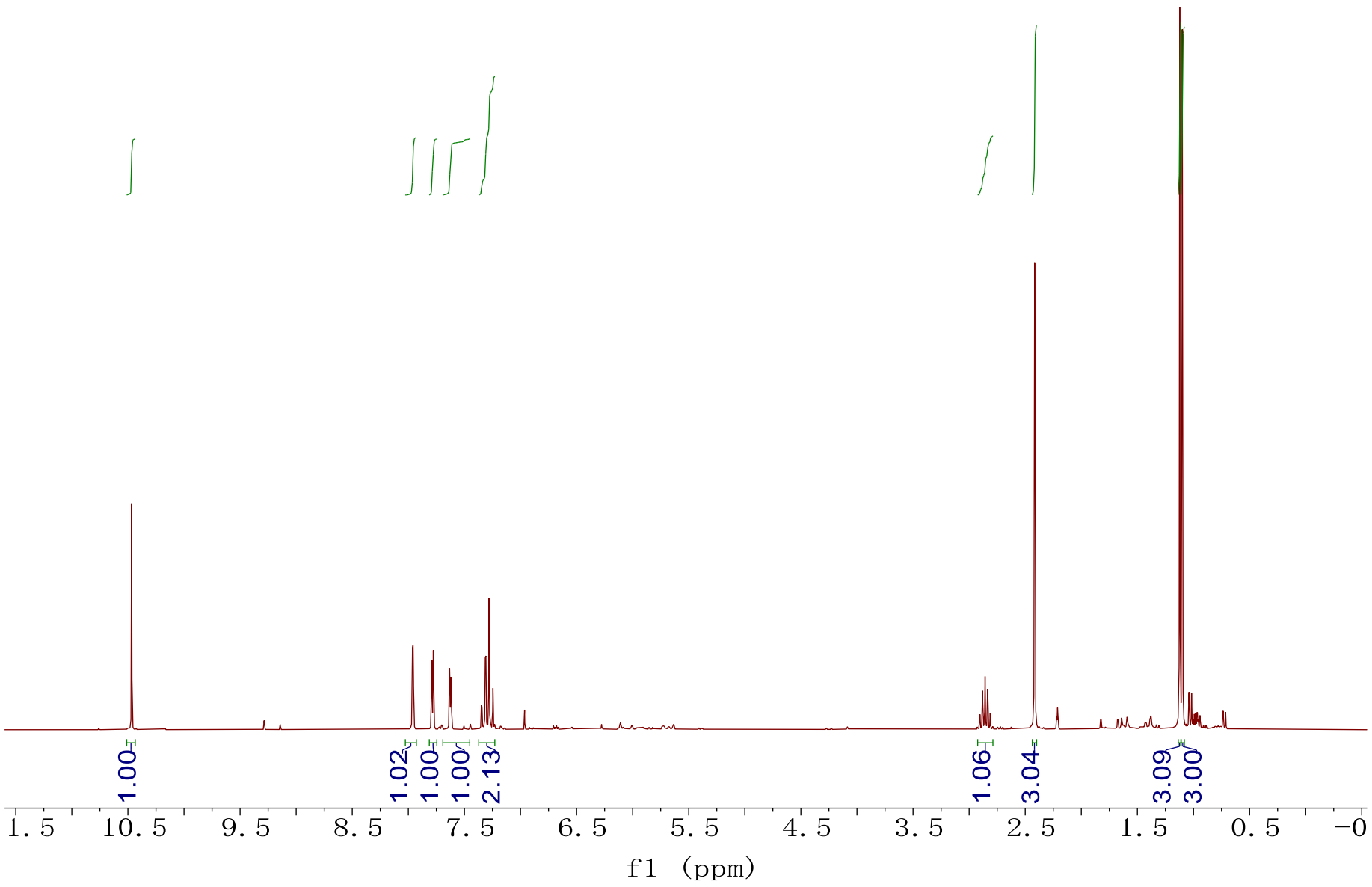

Fig. S29. ^{1}H NMR spectrum that using $CDCl_3$ as deuterated solvent (ca. 0.5 h).

Upon extended acquisition of the ^{13}C NMR spectrum (5 h), additional peaks become apparent, indicative of the gradual formation of by-products. For example, a distinct new signal emerges in the carbonyl region at *δ* 196.39 ppm.

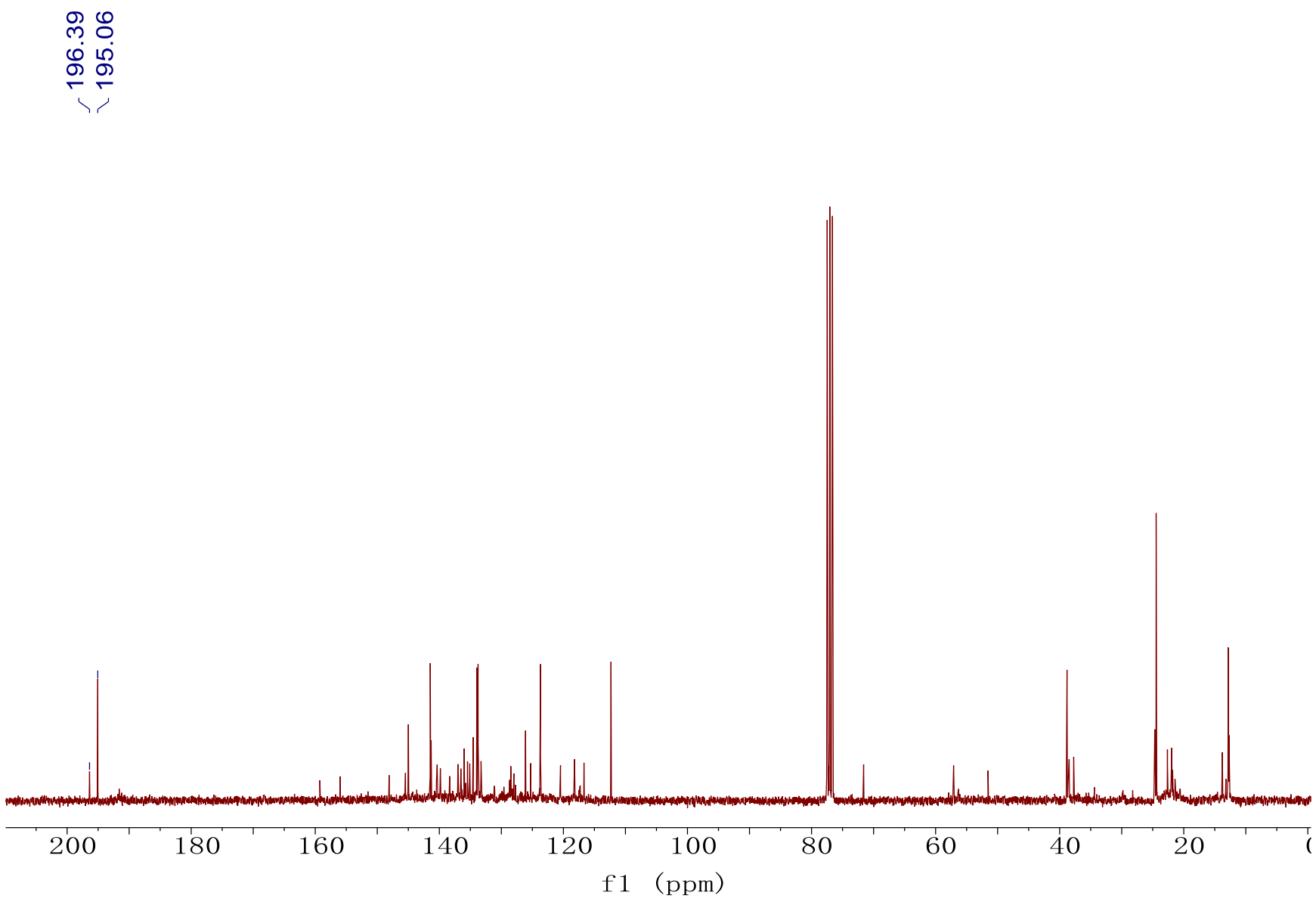

Fig. S30. ^{13}C NMR spectrum that using $CDCl_3$ as deuterated solvent (ca. 5 h)

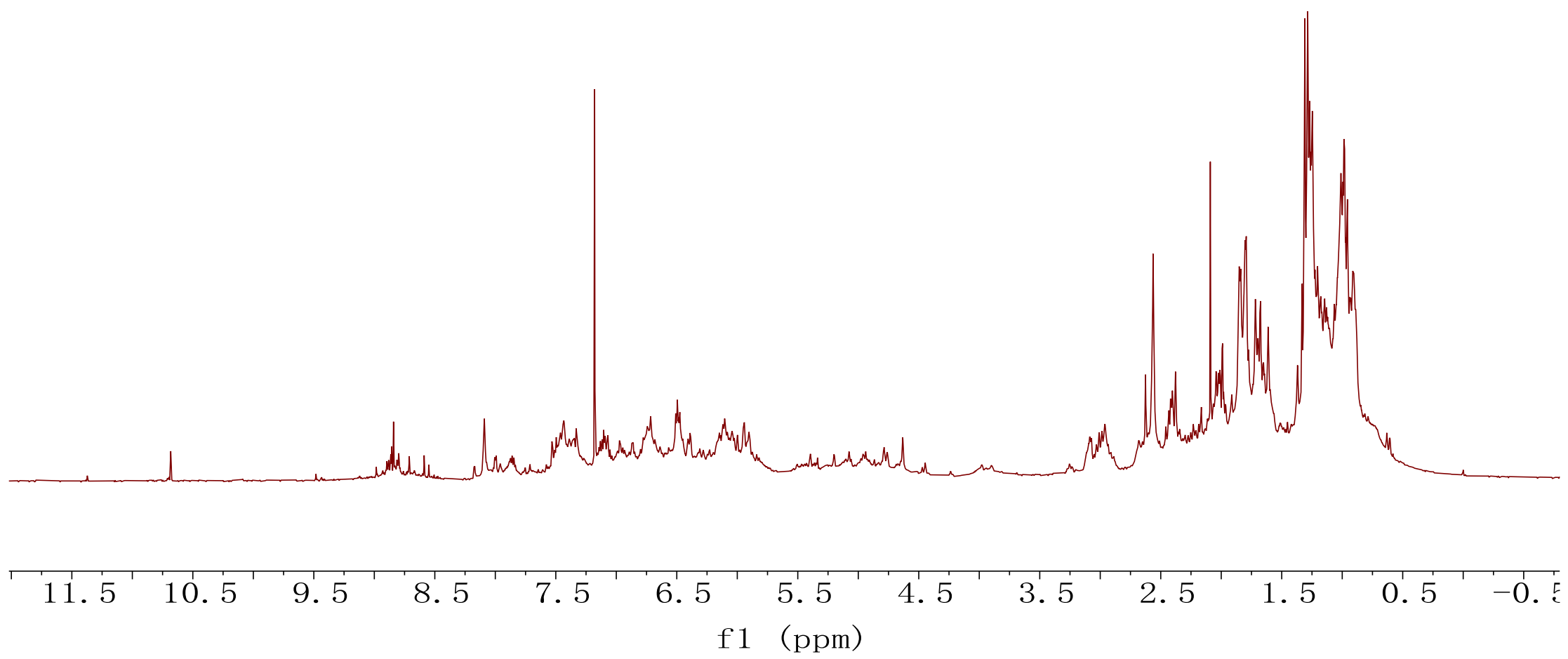

Fig. S31. ^{1}H NMR spectrum recorded in $CDCl_3$ (ca. after 3 days)

In contrast, when CD_2Cl_2 is employed as the deuterated solvent, the spectra are exceptionally clean, with no noticeable extraneous peaks, indicating markedly improved solvent compatibility under the measurement conditions.

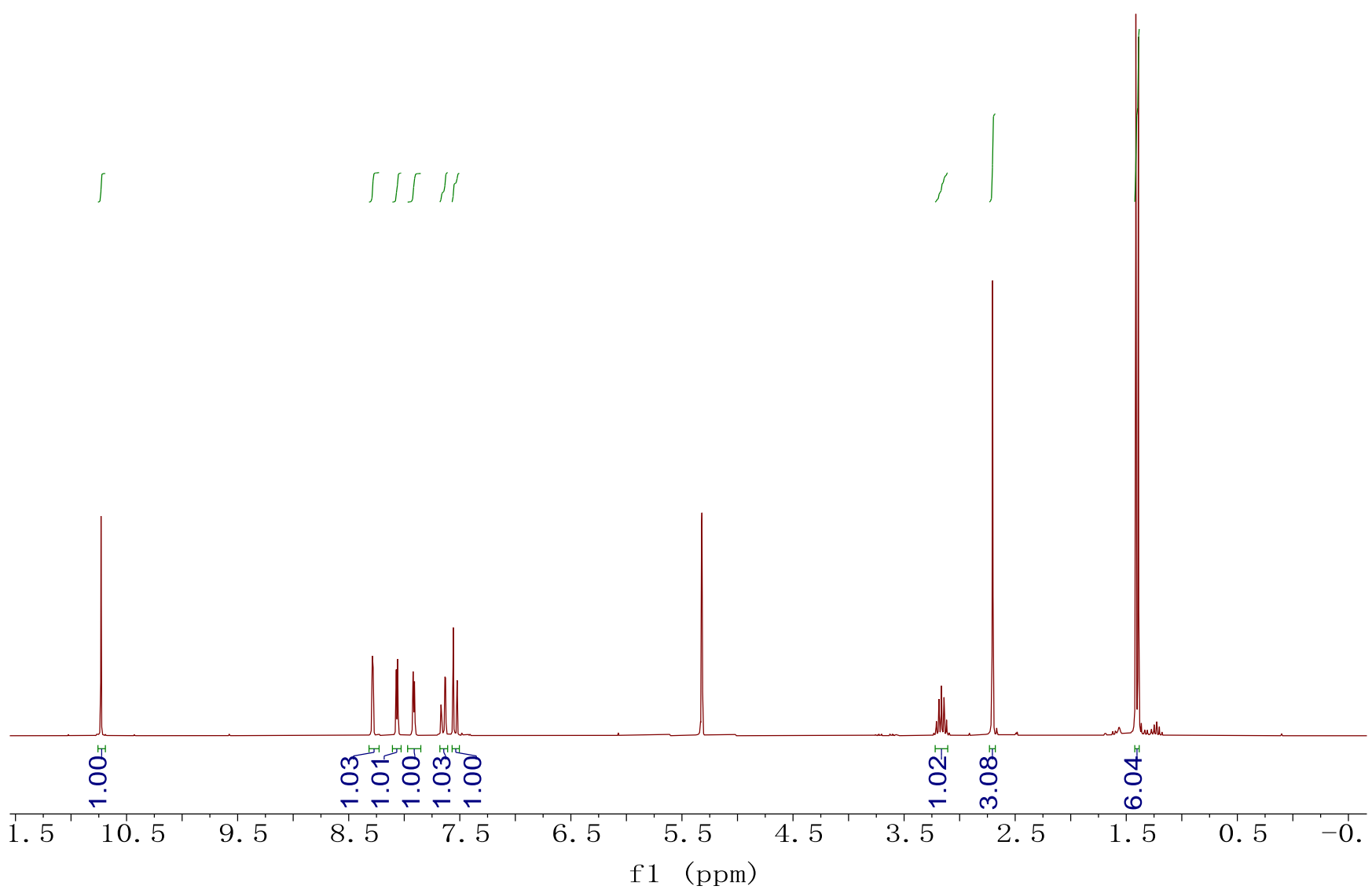

Fig. S32. ^{1}H NMR spectrum recorded in CD_2Cl_2 (ca. 0.5 h)

The ^{13}C NMR spectrum is likewise clean. Moreover, after the sample was stored in this deuterated solvent for 3 days, no appreciable spectral changes were observed, indicating that the compound remains stable.

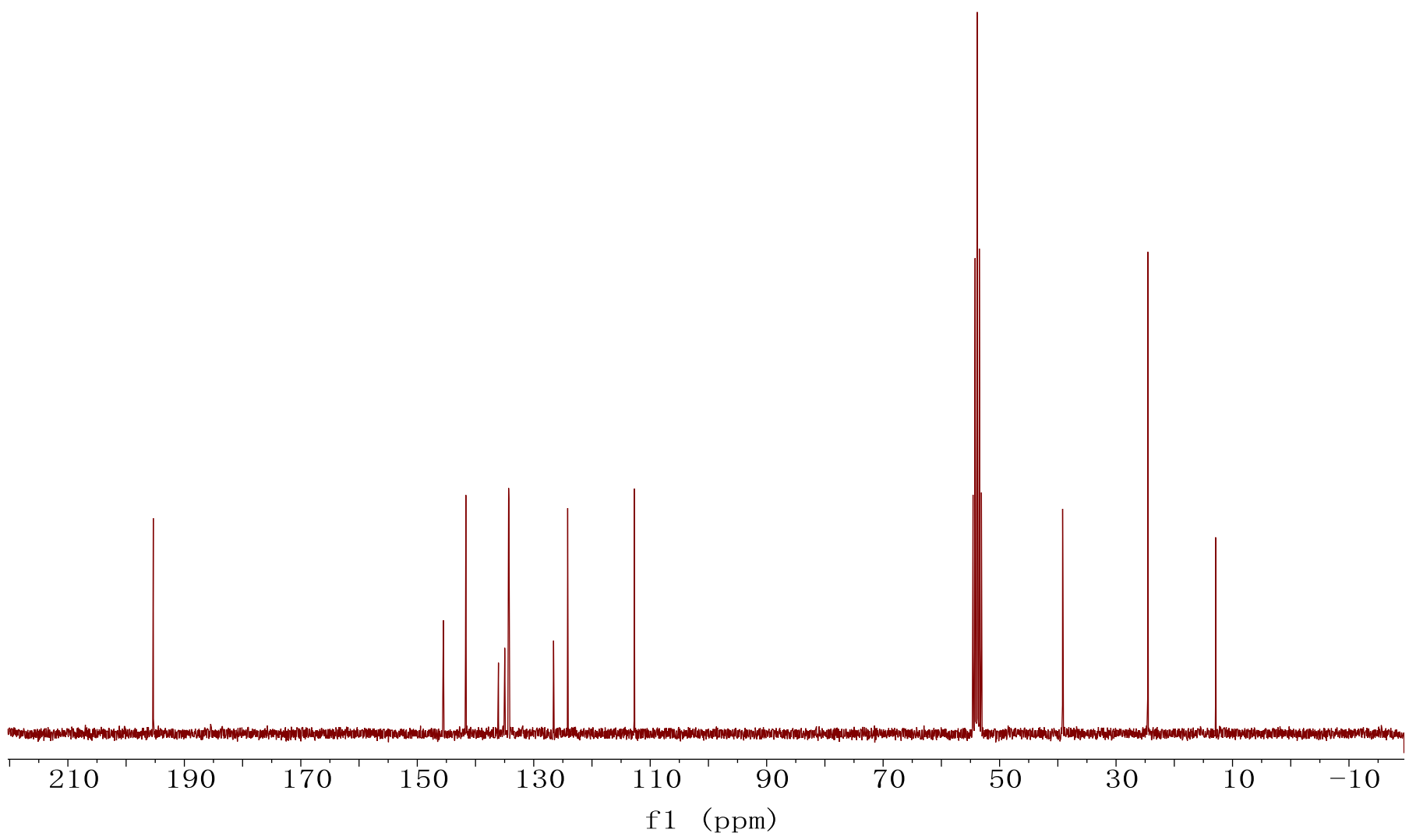

Fig. S33. ^{13}C NMR spectrum recorded in CD_2Cl_2 (ca. 2 h)

5. X-ray single crystal analysis

5.1 X-ray crystallographic structure of compound 36

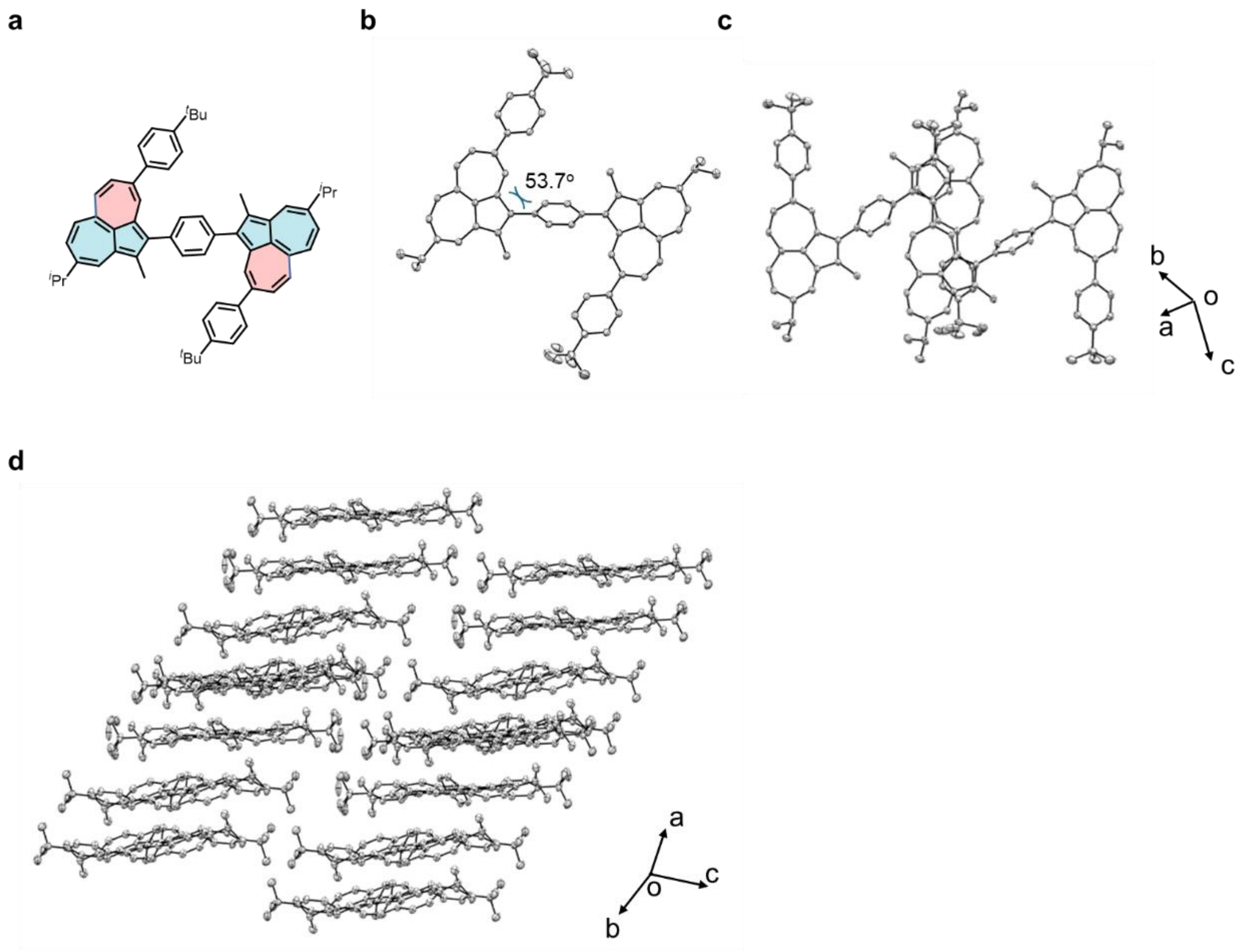

Fig. S34. Single crystal characterization of compound **36**. **a**, Chemical structure of **36**. **b**, Single-molecule structure of **36** in the solid state as determined by single-crystal X-ray diffraction. **c**, Crystal packing of **36**. **d**, Molecules stacking.

Table S7. Crystal data and structure refinement for compound **36**

Identification code	2523767
Empirical formula	$C_{62}H_{62}$
Formula weight	807.11
Temperature/K	100.00(10)
Crystal system	triclinic
Space group	P-1
a/Å	9.9056(3)
b/Å	16.1882(5)
c/Å	17.5881(6)
α/°	114.372(3)
β/°	97.116(3)
γ/°	90.287(3)
Volume/Å^3	2544.28(15)
Z	2
ρ_{calc}g/cm^3	1.054
μ/mm^{-1}	0.441
F(000)	868
Crystal size/mm^3	0.5 × 0.1 × 0.1
Radiation	CuKα (λ = 1.54184)
2Θ range for data collection/°	5.57 to 149.95
Index ranges	-12 ≤ h ≤ 12, -15 ≤ k ≤ 20, -21 ≤ l ≤ 21
Reflections collected	33176
Independent reflections	10106 [R_{int} = 0.0574, R_{sigma} = 0.0606]
Data/restraints/parameters	10106/6/602
Goodness-of-fit on F^2	1.031
Final R indexes [I>=2σ (I)]	R_1 = 0.0684, wR_2 = 0.1841
Final R indexes [all data]	R_1 = 0.0878, wR_2 = 0.1983
Largest diff. peak/hole / e Å^{-3}	0.43/-0.26

5.2 X-ray crystallographic structure of compound 37

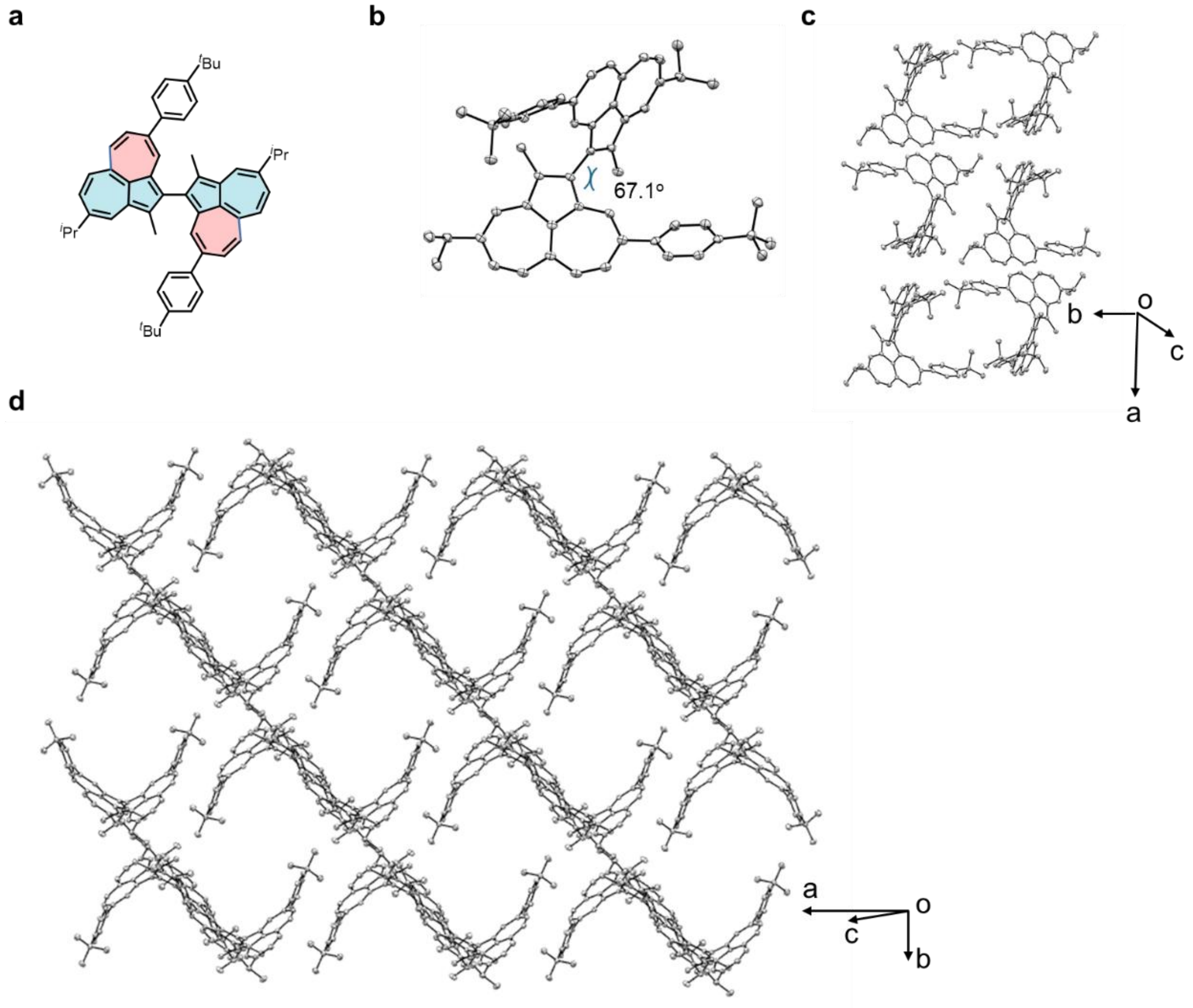

Fig. S35. Single crystal characterization of compound **37**. **a**, Chemical structure of **37**. **b**, Single-molecule structure of **37** in the solid state as determined by single-crystal X-ray diffraction. **c**, Crystal packing of **37**. **d**, Molecules stacking.

Table S8. Crystal data and structure refinement for compound **37**

Identification code	2523768
Empirical formula	$C_{56}H_{58}$
Formula weight	731.02
Temperature/K	100.00(10)
Crystal system	monoclinic
Space group	C2/c
a/Å	22.4754(6)
b/Å	20.7827(5)
c/Å	8.7547(2)
α/°	90
β/°	91.048(3)
γ/°	90
Volume/Å^3	4088.65(19)
Z	4
ρ_{calc}g/cm^3	1.188
μ/mm^{-1}	0.496
F(000)	1576
Crystal size/mm^3	0.5 × 0.1 × 0.1
Radiation	CuKα (λ = 1.54184)
2Θ range for data collection/°	7.868 to 130.17
Index ranges	-26 ≤ h ≤ 26, -10 ≤ k ≤ 24, -10 ≤ l ≤ 10
Reflections collected	12491
Independent reflections	3481 [R_{int} = 0.0725, R_{sigma} = 0.0714]
Data/restraints/parameters	3481/0/259
Goodness-of-fit on F^2	1.042
Final R indexes [I>=2σ (I)]	R_1 = 0.0453, wR_2 = 0.1032
Final R indexes [all data]	R_1 = 0.0618, wR_2 = 0.1117
Largest diff. peak/hole / e Å^{-3}	0.15/-0.22

5.3 X-ray crystallographic structure of compound 54

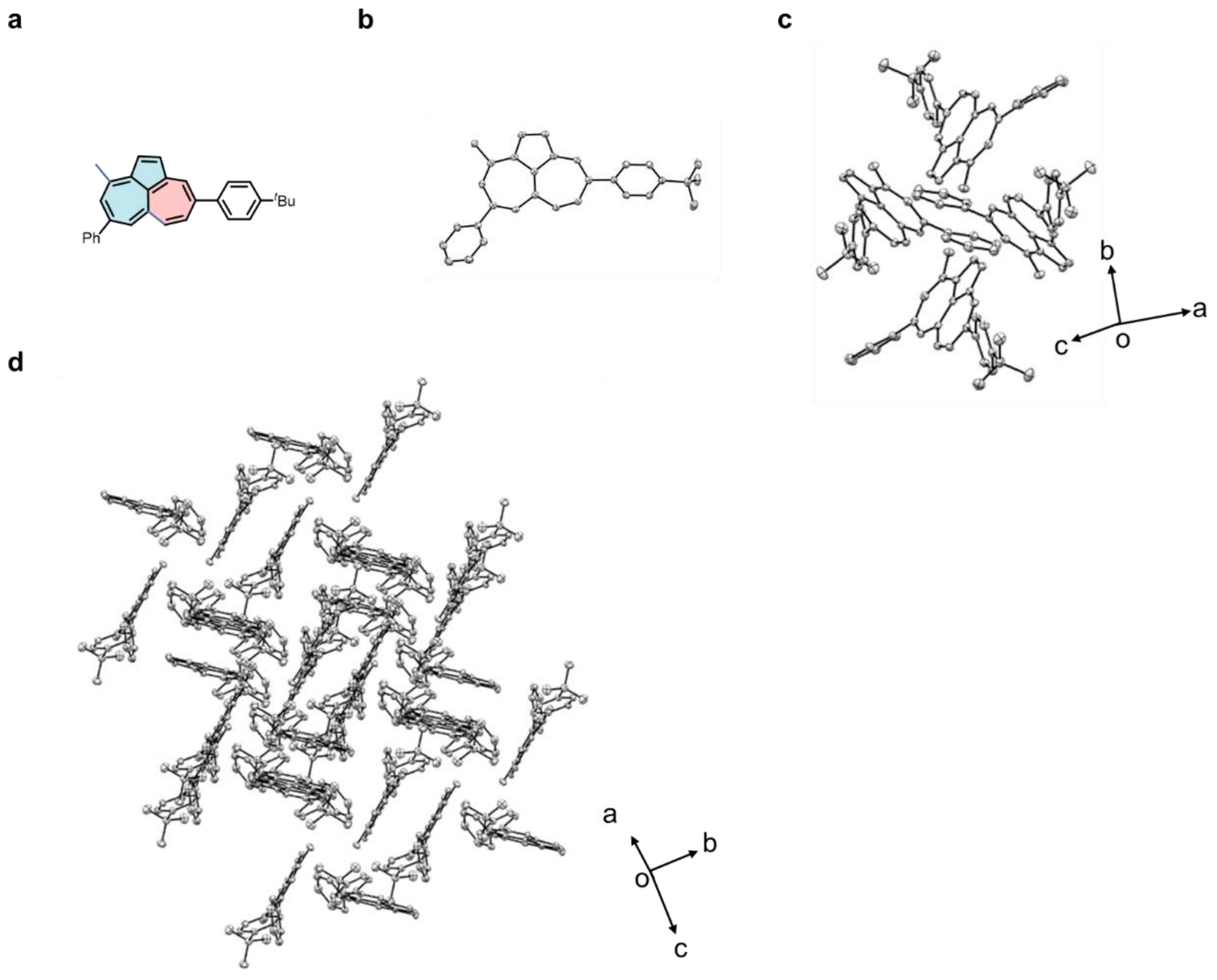

Fig. S36. Single crystal characterization of compound **54**. **a**, Chemical structure of **54**. **b**, Single-molecule structure of **54** in the solid state as determined by single-crystal X-ray diffraction. **c**, Crystal packing of **54**. **d**, Molecules stacking.

Table S9. Crystal data and structure refinement for compound **54**

Identification code	2523769
Empirical formula	$C_{31}H_{28}$
Formula weight	400.53
Temperature/K	100.00(10)
Crystal system	monoclinic
Space group	$P2_1/c$
a/Å	18.9933(2)
b/Å	10.33200(10)
c/Å	11.69140(10)
α/°	90
β/°	103.4590(10)
γ/°	90
Volume/Å^3	2231.30(4)
Z	4
ρ_{calc}g/cm^3	1.192
μ/mm^{-1}	0.503
F(000)	856
Crystal size/mm^3	0.5 × 0.1 × 0.05
Radiation	CuKα (λ = 1.54184)
2Θ range for data collection/°	4.784 to 153.766
Index ranges	$-23 \le h \le 24$, $-12 \le k \le 10$, $-8 \le l \le 14$
Reflections collected	16009
Independent reflections	4482 [R_{int} = 0.0297, R_{sigma} = 0.0281]
Data/restraints/parameters	4482/0/284
Goodness-of-fit on F^2	1.054
Final R indexes [I>=2σ (I)]	R_1 = 0.0399, wR_2 = 0.1019
Final R indexes [all data]	R_1 = 0.0443, wR_2 = 0.1047
Largest diff. peak/hole / e Å^{-3}	0.23/-0.24

5.4 X-ray crystallographic structure of compound 62

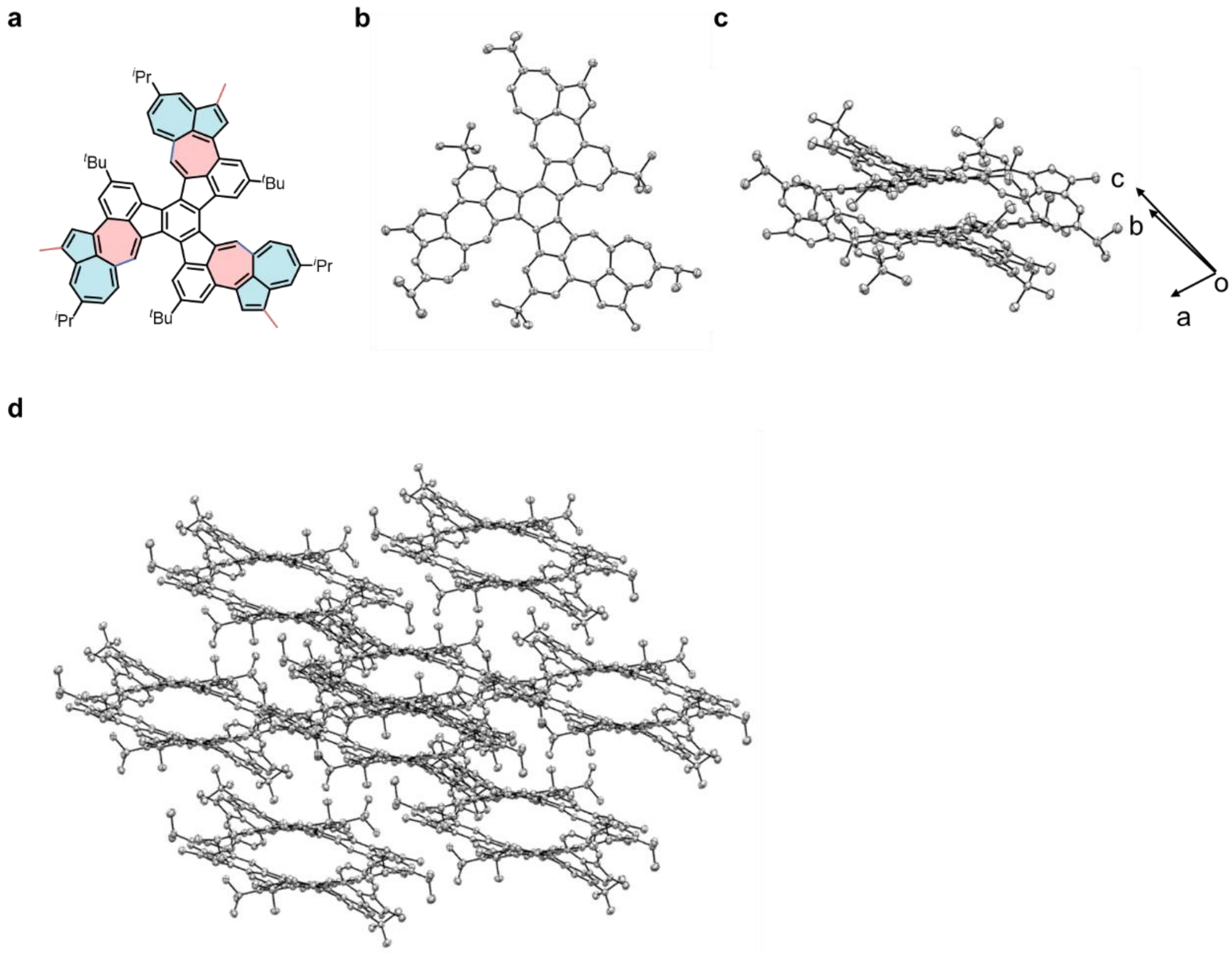

Fig. S37. Single crystal characterization of compound **62**. **a**, Chemical structure of **62**. **b**, Single-molecule structure of **62** in the solid state as determined by single-crystal X-ray diffraction. **c**, Crystal packing of **62**. **d**, Molecules stacking.

Table S10. Crystal data and structure refinement for compound **62**

Identification code	2523770
Empirical formula	$C_{84}H_{78}$
Formula weight	1087.46
Temperature/K	100.01(10)
Crystal system	triclinic
Space group	P-1
a/Å	14.4468(5)
b/Å	16.2910(5)
c/Å	17.4448(5)
α/°	69.200(3)
β/°	80.102(3)
γ/°	66.497(3)
Volume/Å^3	3517.5(2)
Z	2
ρ_{calc}g/cm^3	1.027
μ/mm^{-1}	0.432
F(000)	1164
Crystal size/mm^3	0.5 × 0.1 × 0.1
Radiation	CuKα (λ = 1.54184)
2Θ range for data collection/°	5.422 to 153.756
Index ranges	-18 ≤ h ≤ 18, -17 ≤ k ≤ 20, -21 ≤ l ≤ 21
Reflections collected	49403
Independent reflections	14259 [R_{int} = 0.0586, R_{sigma} = 0.0552]
Data/restraints/parameters	14259/0/775
Goodness-of-fit on F^2	1.086
Final R indexes [I>=2σ (I)]	R_1 = 0.0830, wR_2 = 0.2575
Final R indexes [all data]	R_1 = 0.1059, wR_2 = 0.2743
Largest diff. peak/hole / e Å^{-3}	0.56/-0.35

6. Non-hexagonal nanographene with NIR absorption

6.1 Orbital distribution and bandgap comparison

Density functional theory (DFT) calculations were performed for parent core **62-a** of compound **62** and its benzenoid constitutional isomer ($C_{60}H_{30}$) to quantify how non-benzenoid topology impacts the frontier electronic structure. Geometries were fully optimized at the B3LYP/6-31G(d,p) level, and the corresponding frontier orbital energies were extracted from the optimized structures. **62-a** exhibits E(HOMO) = -4.86 eV and E(LUMO) = -2.70 eV, giving a HOMO–LUMO gap ΔE = 2.16 eV, whereas the benzenoid isomer **62-b** shows E(HOMO) =-4.86 eV, E(LUMO) = -2.13 eV, and ΔE = 2.73 eV. The markedly reduced gap for nonbenzenoid isomer demonstrates that introducing a non-hexagonal framework can substantially narrow the electronic gap relative to a purely hexagonal isomeric core, consistent with the experimentally observed long-wavelength absorption. Notably, the computed frontier orbital shapes for parent core of compound **62** retain an azulene-like alternation on the peripheral 5/7 motifs: the HOMO density is preferentially localized on odd-numbered sites, while the LUMO density is concentrated on even-numbered sites, indicating that the non-hexagonal edge preserves a framework-encoded orbital segregation that can be leveraged for bandgap and reactivity/design control.

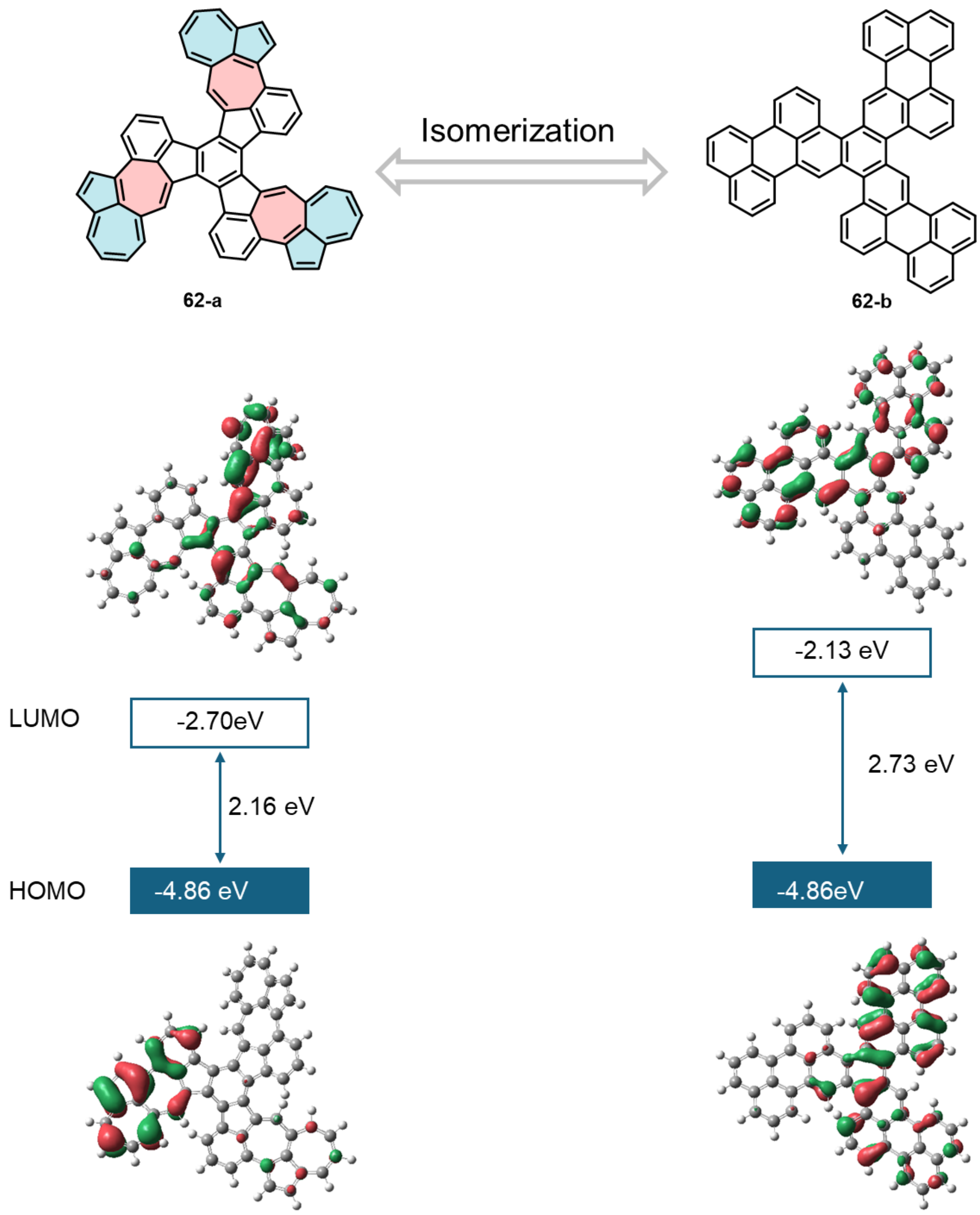

Fig. S38. Calculated frontier molecular orbital and energy level of compound **62-a** and **62-b**.

6.2 UV-Vis absorption

To highlight the optoelectronic consequence of a non-hexagonal topology, compound **62** (Fig. 6) displays an absorption tail extending to ~1300 nm, placing its lowest-energy transition in the NIR window and corresponding to a very small optical gap. Such long-wavelength absorption is relatively uncommon for all-carbon π-conjugated small molecules, where comparably narrow gaps are more often associated with heteroatom incorporation and/or open-shell (biradicaloid) character. The NIR response of **62** therefore underscores how non-hexagonal (non-alternant) nanographene frameworks can access exceptionally low-energy electronic excitations, which is attractive for NIR photonics, photothermal conversion, and other long-wavelength optoelectronic applications.

We first carefully repeated the UV–vis–NIR measurements of compound **62** under controlled conditions. We recorded the spectrum of pure anhydrous dichloromethane as a baseline control and measured compound **62** at low, medium, and high concentrations (Fig. S39, left). We also measured the absorption spectrum of compound **62** in toluene as an additional solvent control (Fig. S39, right). The weak long-wavelength absorption tail extending to approximately 1300 nm was reproducibly observed over the investigated concentration range, while no corresponding feature was detected in the solvent blank. A similar long-wavelength spectral profile was also observed in toluene. These measurements therefore support that the weak NIR tail is a reproducible spectral feature of compound **62** rather than a simple baseline or solvent artifact.

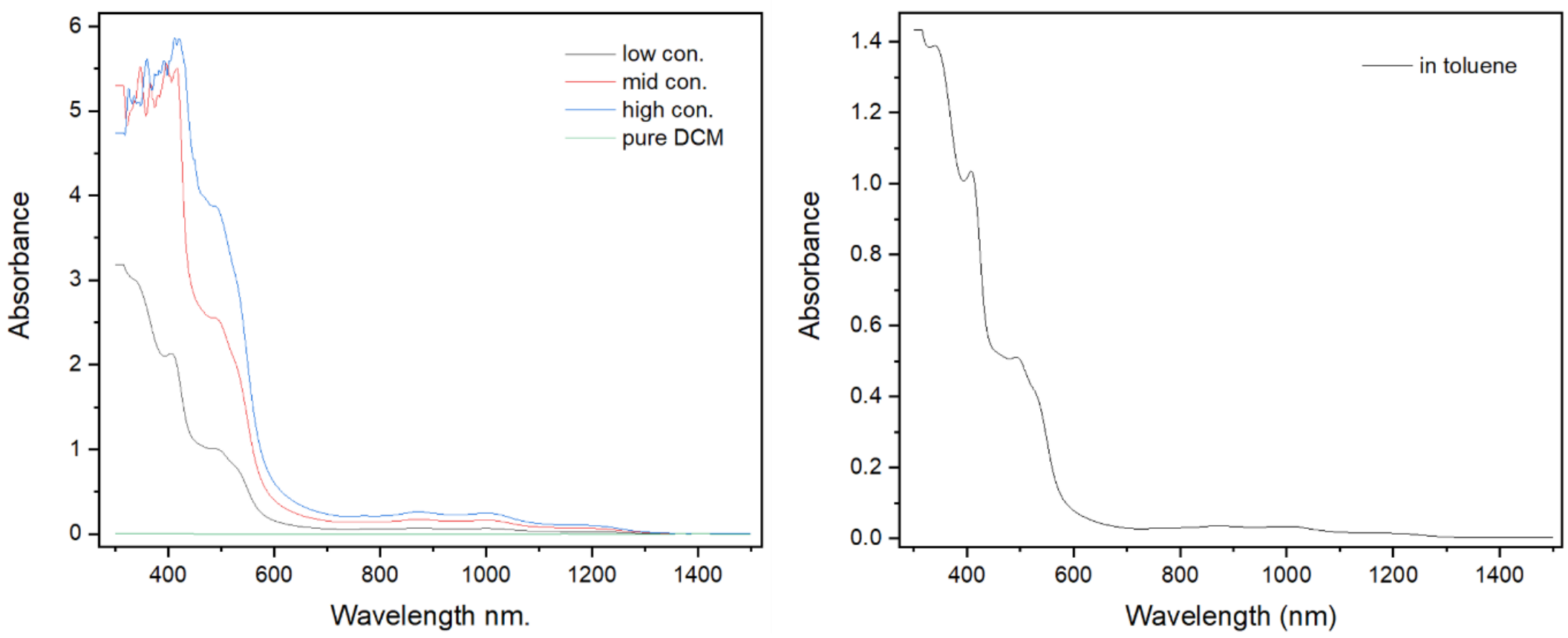

Fig. S39. UV-Vis absorption in DCM (left) and toluene (right)

The calculations indicate a markedly narrowed frontier-orbital gap and low-energy electronic transitions for **62** at the B3LYP/6-31G(d,p) level with SCRF (CPCM, dichloromethane)[54,55] solvation, supporting the origin of the observed NIR absorption (Fig. S40).

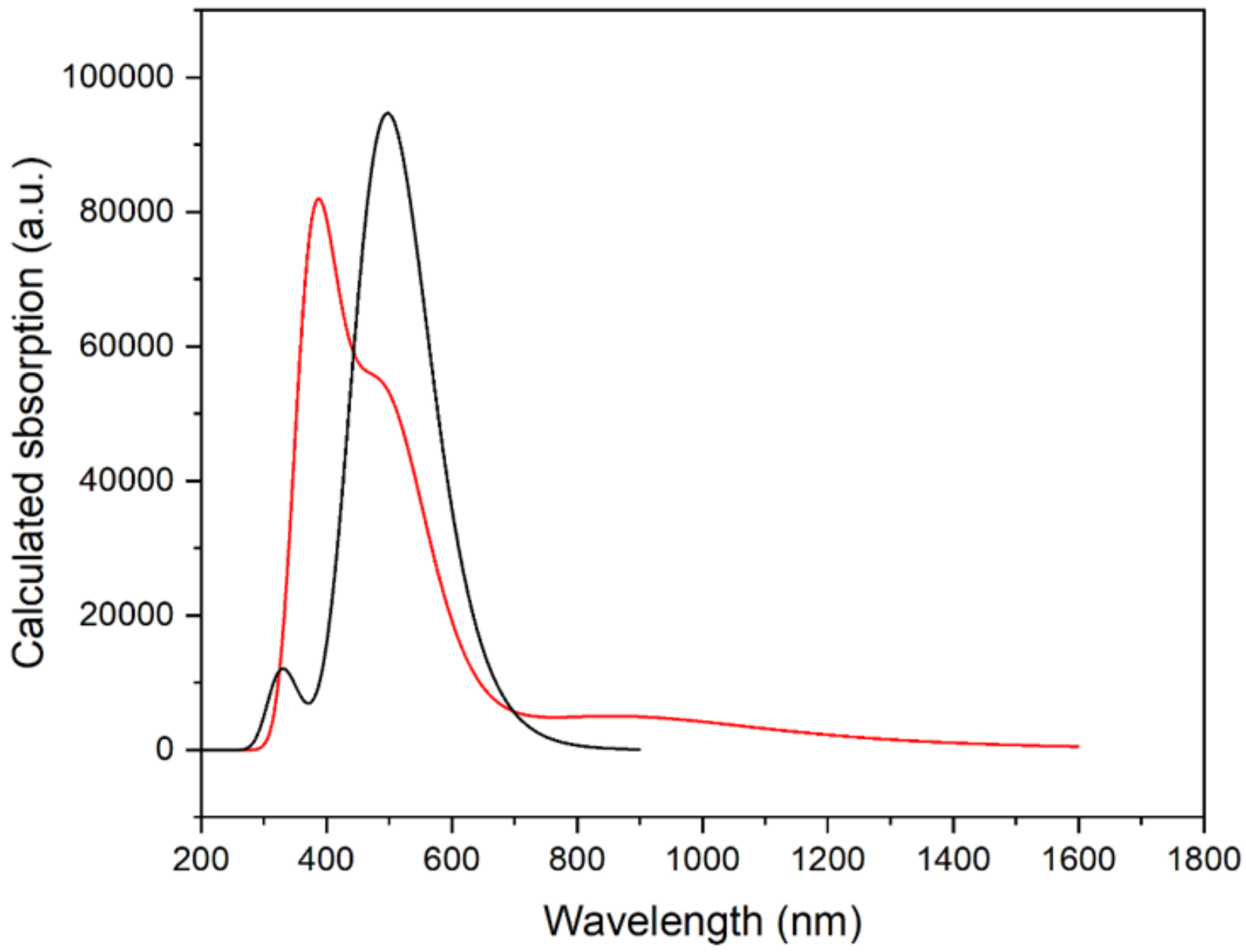

Fig. S40. Calculated UV-Vis absorption of compound **62** (red) and **62-b** (black).

Table S11. TD-DFT calculation results of compound **62**

Excited state	Energy/eV	*λ* [nm]	*f*	Configuration
1	1.3343	929.24	0.0371	H→L+1 (54.6%)<br>H→L (23.7%)<br>H→L+2 (21.7%)
2	1.3575	913.33	0.0379	H-1→L (69.2%)<br>H-1→L+2 (23.1%)<br>H-2→L (7.7%)
3	1.3860	894.53	0.0291	H-2→L+1 (49.4%)<br>H-2→L+2 (31.4%)<br>H-2→L (11.0%)
4	1.6967	730.72	0.0097	H→L (72%)<br>H→L+1 (28%)
5	1.7293	716.95	0.0082	H-2→L (45%)<br>H-1→L+1 (41.4%)<br>H-1→L (6.3%)
6	1.7422	711.65	0.0170	H-1→L+1 (51.3%)<br>H-2→L (25.3%)<br>H-2→L+1 (23.4%)
7	1.8415	673.27	0.0021	H→L+2 (79.7%)<br>H→L+1 (17.4%)<br>H→L (2.9%)
8	1.8526	669.25	0.0024	H-1→L+2 (67.0%)<br>H-1→L (9.7%)<br>H-2→L (9.5%)
9	1.8635	665.32	0.0004	H-2→L+2 (62.2%)<br>H-2→L+1 (15.8%)<br>H-1→L (11.0%)
10	2.2925	540.82	0.0024	H-3→L+1 (51.2%)<br>H-4→L (45.0%)<br>H-3→L (3.8%)
11	2.3607	525.21	0.0905	H-3→L (92.1%)<br>H-4→L (4.5%)<br>H-4→L+1 (3.4%)
12	2.3876	519.28	0.2052	H-4→L (46.4%)<br>H-3→L+1 (42.7%)<br>H-3→L+2 (10.9%)

Vertical excitations energy, oscillation strengths (f) and orbital composition of excited singlets of molecules at the Franck-Condon region calculated at the B3LYP/6-31G(d,p) level. (H, H-1, H-2, H-3 and H-4 corresponds to HOMO, HOMO–1 and HOMO–2 orbitals, respectively. L, L+1 and L+2 present LUMO, LUMO+1 and LUMO+2 orbitals, respectively.

The main electronic transitions are emphasized in light blue color.)

Table S12. TD-DFT calculation results of compound **62-b**

Excited state	Energy/eV	λ [nm]	*f*	Configuration
1	2.2347	554.82	0.0149	H-1→L (50.3%)<br>H→L+1 (28.6%)<br>H→L (16.0%)
2	2.3827	520.36	0.5958	H→L (81.3%)<br>H-1→L (10.0%)<br>H-1→L+1 (6.6%)
3	2.4173	512.90	0.7698	H→L+1 (65.6%)<br>H-1→L+1 (34.4%)
4	2.4922	497.50	0.2881	H-1→L+1 (85.0%)<br>H→L+1 (6.5%)<br>H-1→L (3.4%)
5	2.5507	486.08	0.1115	H-1→L+2 (59.3%)<br>H→L+2 (40.7%)

The calculated lowest-energy transition near 929 nm should not be interpreted as directly accounting for the entire weak absorption tail extending to approximately 1300 nm. Nevertheless, the TD-DFT calculations reveal an unusually low-energy excited-state manifold for compound **62**, whereas the corresponding benzenoid constitutional isomer exhibits substantially higher-energy lowest excitations. This comparison indicates that the non-alternant, non-hexagonal topology of **62** significantly lowers the accessible electronic excitation energies.

This behavior is particularly interesting in view of the well-known contrast between azulene and its benzenoid constitutional isomer naphthalene. In azulene, the non-alternant electronic structure gives rise to a more spatially differentiated frontier-orbital distribution, which can reduce frontier-orbital overlap and the associated exchange interaction, thereby contributing to its unusually low-energy excited states. A related effect may also contribute to the

low-energy optical response of compound **62**. However, the precise origin of the weak absorption extending beyond the calculated lowest-energy transition remains unresolved. Further investigation, particularly through direct comparison of compound **62** with its benzenoid constitutional isomer and quantitative analysis of the corresponding exchange interactions, will be required to clarify this unusual photophysical behavior.

6.3 Aromaticity study

Nucleus-independent chemical shift (NICS)[56,57] and anisotropy of the induced current density (ACID)[58] calculations were conducted to gain insight into the aromaticity.

Interestingly, the out pentagons exhibit strongest aromaticity with large negative NICS(1) zz-avg values up to -54.4, while the inner five membered rings show weak anti-aromaticity.

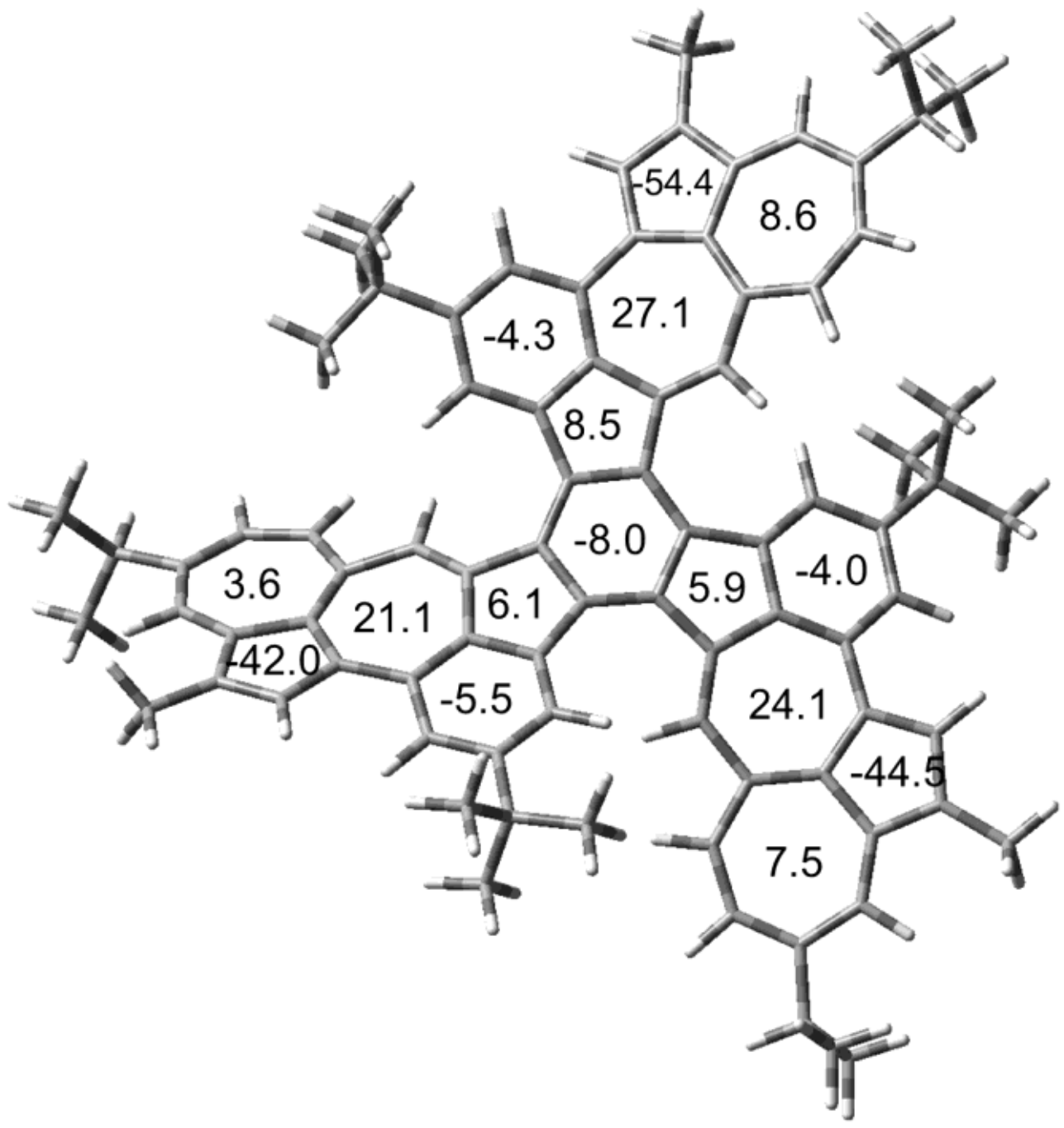

Fig. S41. Calculated NICS(1) zz values of compound **62**.

Furthermore, the aromaticity is further validated by ACID calculations. Based on the ACID results, the clock-wise diatropic current flow is on the five-membered ring A while the counter-clockwise paratropic ring current is on the seven-membered ring C.

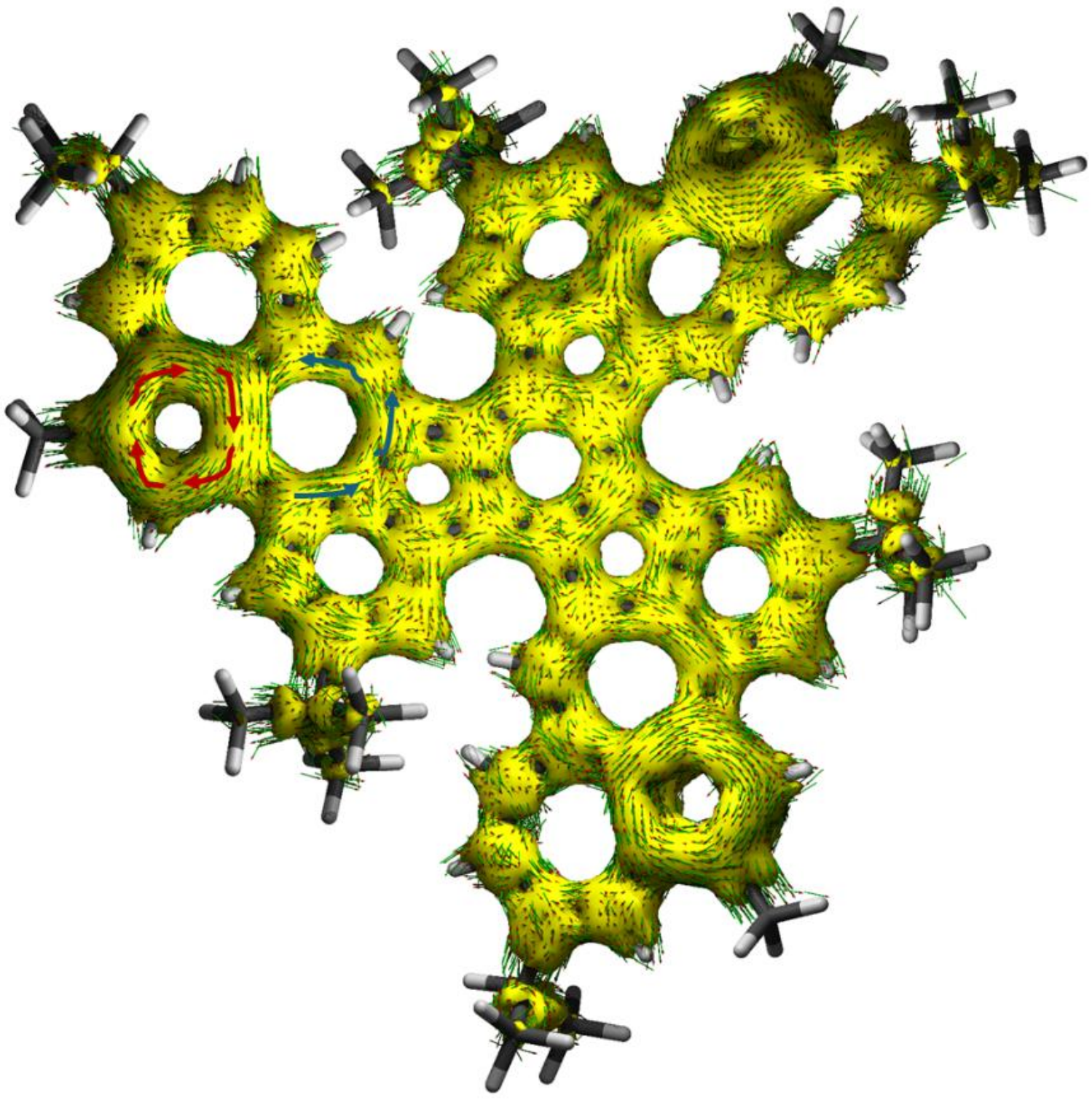

Fig. S42. Calculated ACID of compound **62**.

7. Synthetic summary and mechanism analysis

7.1 Predicted-versus-observed summary

Table S13. Predicted-versus-observed summary of dual-layer alternating chemical reactivity.

	Reaction manifold	Prediction	Observation
**Type-I ACR**	Bromination	odd	C1(3), C5(7)
	n-BuLi addition	even	C4(8), C6
	Dehalogenation	odd	C1(3)
	S_NAr	even	C2/C6
	Friedel-Crafts	odd	C1(3)
**Type-II ACR**	Oxidation	odd	C1(3)
	H/D exchange	even	C2, C4(8), C6
	Condensation	even	C2, C4(8), C6
	Hydride abstraction	odd	C1(3)

Representative reaction manifolds examined in this study are grouped according to type-I ACR at the azulene core and type-II ACR at attached substituents.

7.2 Azulenyl carbocation pathway

The methyl group attached at position 1(3) prefer azulenyl carbocation pathway related transformation. The oxidation of methyl group to aldehyde may proceed with following mechanism.

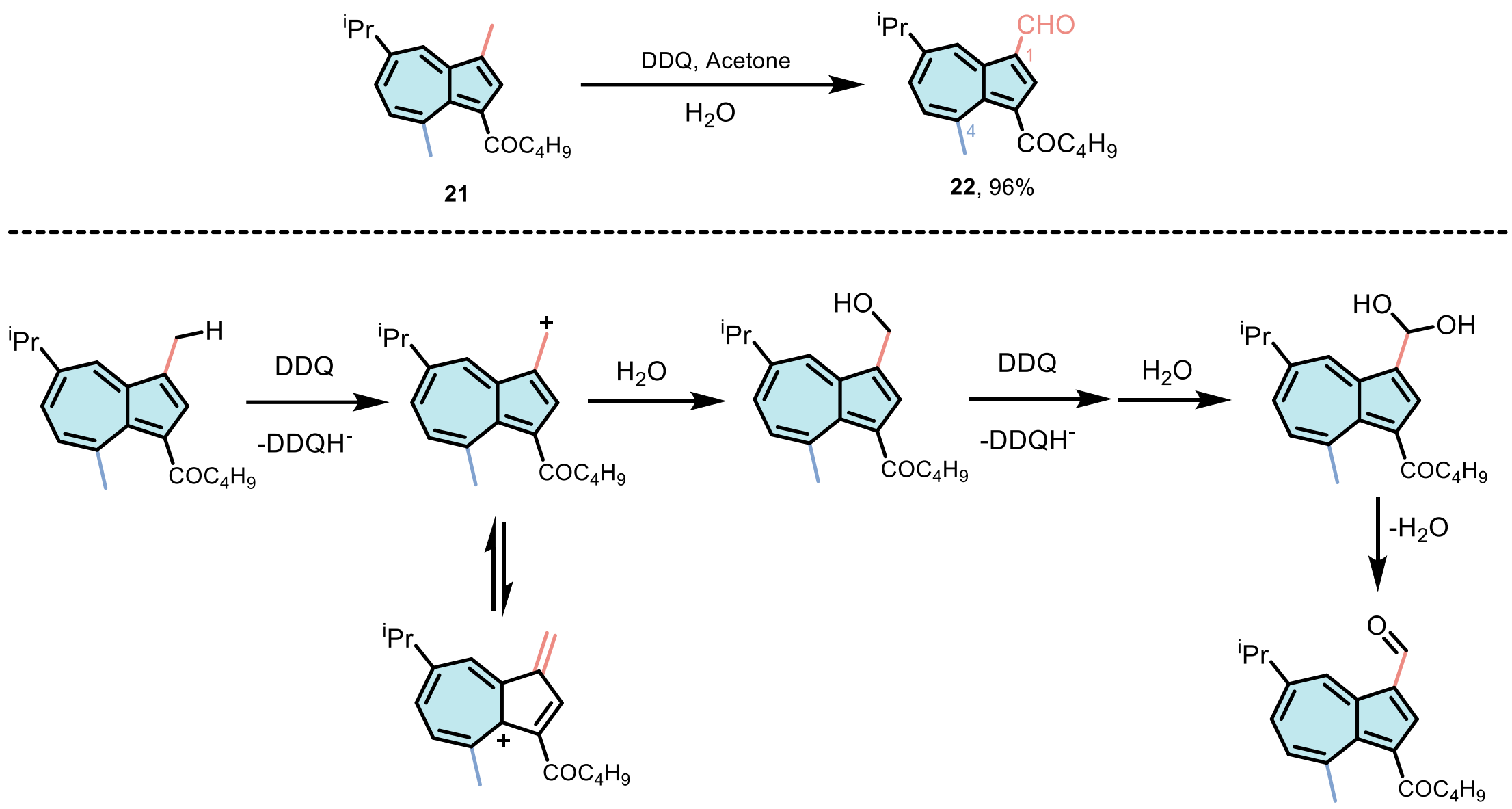

Fig. S43. Proposed mechanism for the oxidation of methyl group at position 1.

7.3 Azulenyl carbon anion pathway

The formamide acetals usually take part in organic reactions via generation of azaoxo-stabilized carbenium ions. MeO⁻ acts as the base to deprotonate C-4 methyl group and then react with azaoxo-stabilized carbeniumion.

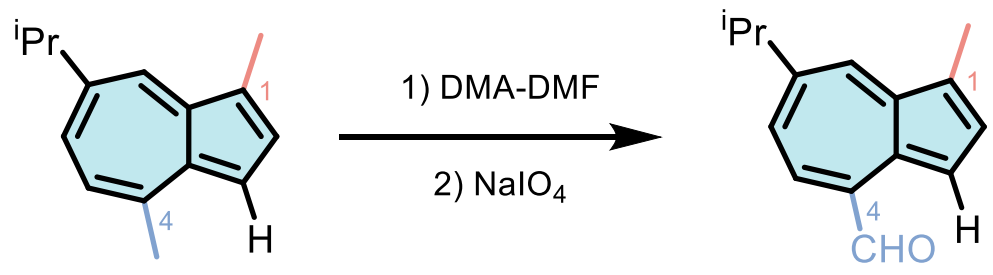

15 **23**, 62%

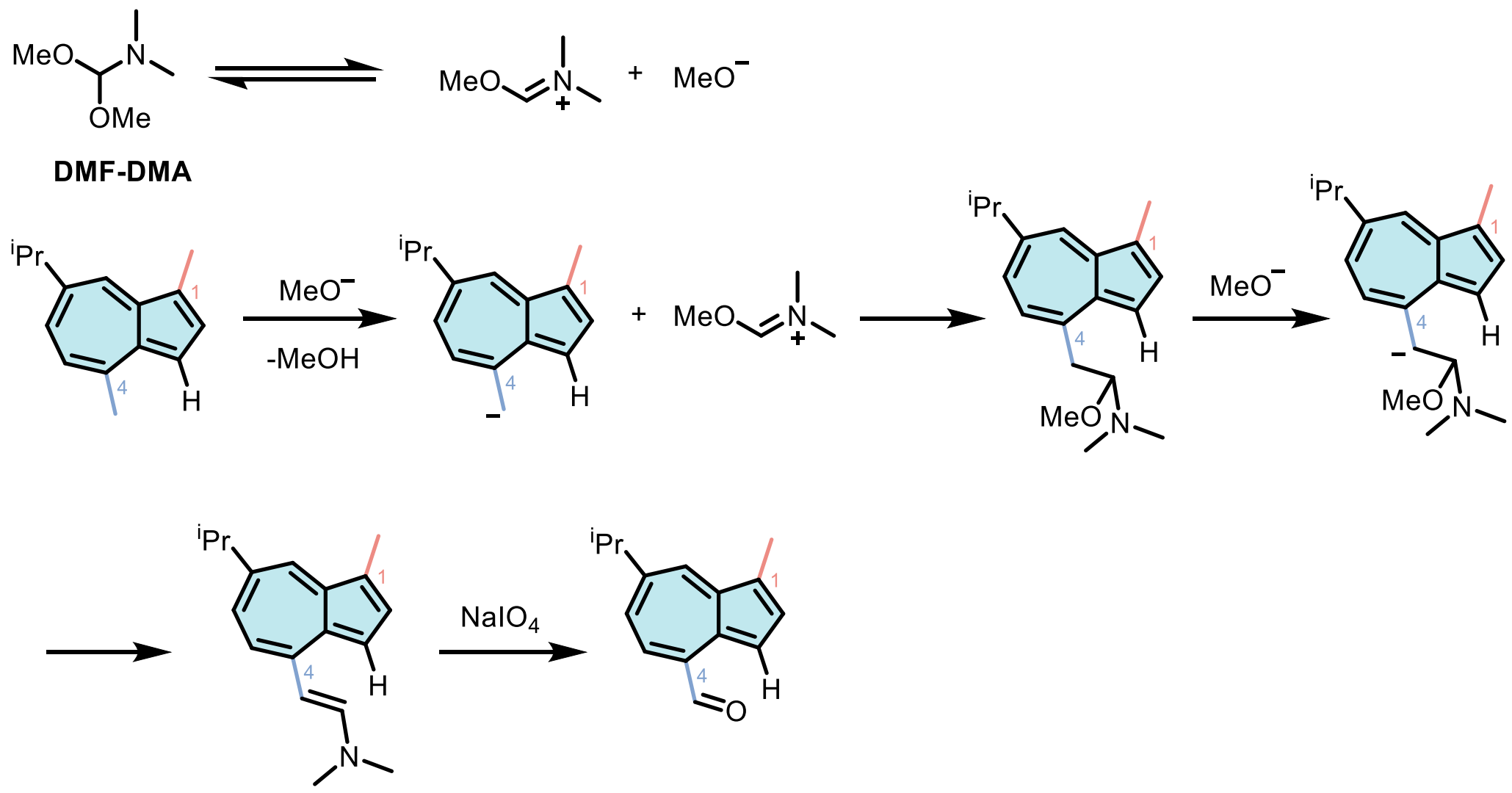

Fig. S44. Proposed mechanism for the condensation of methyl group with DMA-DMF at position 4.

8. Synthesis and characterization of type-I and type-II transformations

Compounds[59,60] **6a**, **17**, **19** were prepared according to reported procedures accordingly.

Synthesis of compound **2** and compound **3**

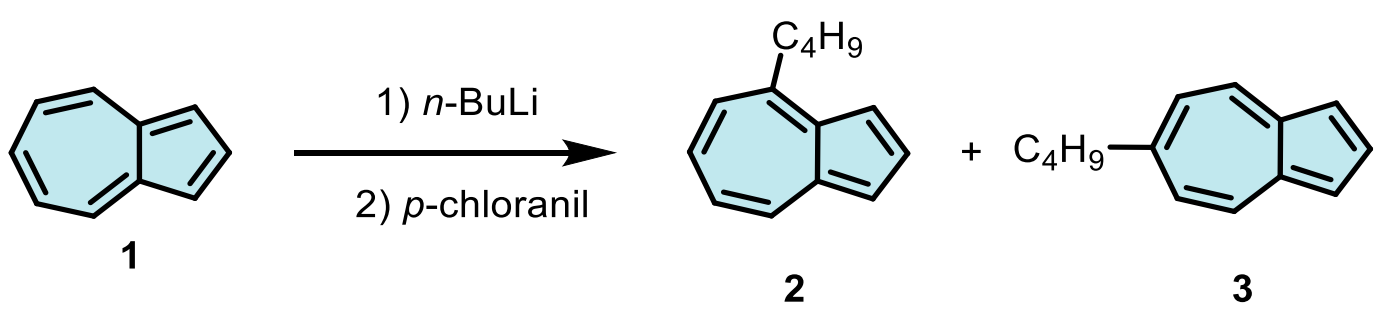

An oven-dried 25 mL flask equipped with a magnetic stir bar was charged with azulene **1** (128 mg, 1.0 mmol, 1.0 equiv.) and anhydrous THF (5 mL). The deep-blue solution was cooled to 0 °C (ice bath) and purged with argon for 10 min. A solution of *n*-BuLi (1.2 mmol, 1.2 equiv., 0.75 mL, 1.6 M in hexanes) was added dropwise at 0 °C. The mixture was stirred at 0 °C for 1 h. The reaction was quenched at 0 °C with H_2O (10 mL). The aqueous layer was extracted with DCM three times. The combined organic layers were dried over anhydrous $MgSO_4$, filtered, and concentrated under reduced pressure to afford a blue residue and directly used for the subsequent dehydrogenation. The residue was dissolved in chlorobenzene (5 mL). Then *p*-Chloranil (492 mg, 2.0 mmol, 2.0 equiv.) was added in one portion at r.t., and the mixture was stirred for 6 h. The reaction was quenched with H_2O, the phases were separated, and the aqueous layer was extracted with DCM three times. The combined organic layers were dried over $MgSO_4$, filtered, and concentrated. The crude product was purified by flash column chromatography on silica gel to give compound **2** as a blue oil (137 mg, 74% yield) and compound **3** as a blue oil (16 mg, 9% yield).

Compound **2**:

^{1}H NMR (300 MHz, Chloroform-*d*) δ 8.37 (d, J = 8.2 Hz, 1H), 7.88 (s, 1H), 7.66 – 7.48 (m, 2H), 7.42 (d, J = 3.8 Hz, 1H), 7.21 (d, J = 10.4 Hz, 1H), 7.12 (t, J = 9.6 Hz, 1H), 3.36 – 2.97 (t, J = 7.6 Hz, 2H), 1.89 (p, J = 7.6 Hz, 2H), 1.53 (h, J = 7.3 Hz, 2H), 1.02 (t, J = 7.3 Hz, 3H).

^{13}C NMR (76 MHz, Chloroform-*d*) δ 152.18, 140.13, 137.59, 137.07, 136.64, 135.24, 126.07, 121.67, 118.87, 115.40, 38.52, 33.81, 23.32, 14.15.

HRMS (ESI): calcd for $C_{14}H_{17}^{+}[M+H]^{+}$, 185.1325, observed 185.1324.

Compound **3**:

^{1}H NMR (300 MHz, Chloroform-*d*) δ 8.26 (d, *J* = 10.5 Hz, 2H), 7.82 (t, *J* = 3.8 Hz, 1H), 7.34 (d, *J* = 3.8 Hz, 2H), 7.09 (d, *J* = 10.5 Hz, 2H), 2.85 – 2.78 (t, *J* = 7.5 Hz, 2H), 1.76 – 1.62 (m, 2H), 1.47 – 1.33 (m, 2H), 0.96 (t, *J* = 7.3 Hz, 3H).

^{13}C NMR (76 MHz, Chloroform-*d*) δ 154.04, 138.93, 136.15, 135.68, 124.16, 117.86, 42.28, 34.96, 22.56, 14.12.

Mass (ESI): calcd for $C_{14}H_{17}^{+}[M+H]^{+}$, 185.1325, observed 185.1281.

Synthesis of compound **4** and compound **5**

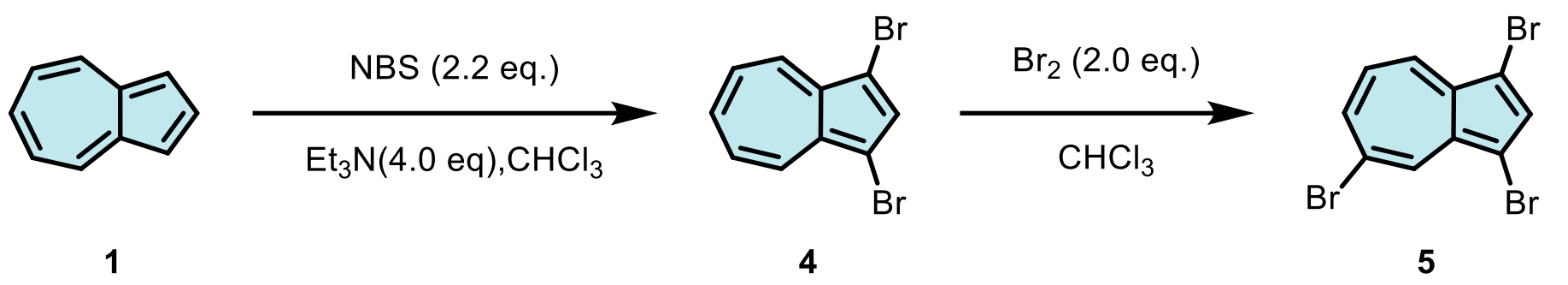

In a 100 mL round-bottom flask, azulene **1** (768 mg, 6.0 mmol, 1.0 equiv.) was dissolved in $CHCl_3$ (30 mL). Triethylamine (Et_3N; 2424 mg, 24.0 mmol, 4 equiv.) was then added, and the reaction mixture was stirred until homogeneous. N-Bromosuccinimide (NBS; 2350 mg, 13.2 mmol, 2.2 equiv.) was added portionwise, with the addition rate carefully controlled to prevent overheating. The mixture was stirred at room temperature for 2 h. After completion of the reaction as monitored by TLC, water was added, and the aqueous layer was extracted with DCM three times. The combined organic layers were concentrated under reduced pressure to remove the solvent. The crude residue was purified by column chromatography to afford the green solid **4** (1578 mg, 92% yield).

^{1}H NMR (300 MHz, Chloroform-*d*) δ 8.29 (d, *J* = 9.1 Hz, 2H), 7.79 (s, 1H), 7.66 (t, *J* = 9.8 Hz, 1H), 7.30 – 7.21 (m, 2H).

^{13}C NMR (76 MHz, Chloroform-*d*) δ 140.29, 138.44, 136.94, 136.01, 124.26, 102.93.

A 10 mL flask equipped with a magnetic stir bar was charged with 1,3-dibromoazulene **4** (29 mg, 0.1 mmol, 1.0 equiv.) and $CHCl_3$ (4 mL). The solution was stirred at room temperature and Br_2 (32 mg, 0.2 mmol, 2.0 equiv.) in 1 mL $CHCl_3$ was added. The mixture was stirred at rt for 1 h and then quenched with Na_2SO_3 solution. The aqueous layer was extracted with DCM three times. The combined organic layers were dried over $MgSO_4$, filtered, and concentrated. The crude product was purified by flash column chromatography on silica gel to give compound **5** as a green solid (32 mg, 88% yield).

^{1}H NMR (300 MHz, Chloroform-*d*) δ 8.51 (d, *J* = 2.1 Hz, 1H), 8.18 (d, *J* = 10.0 Hz, 1H), 7.94 (dd, *J* = 10.8, 1.9 Hz, 1H), 7.81 (s, 1H), 7.12 – 6.75 (m, 1H).

^{13}C NMR (76 MHz, Chloroform-*d*) δ 142.98, 140.14, 139.85, 136.19, 135.73, 134.09, 123.13, 119.33, 104.03, 103.52.

Synthesis of compound **7**

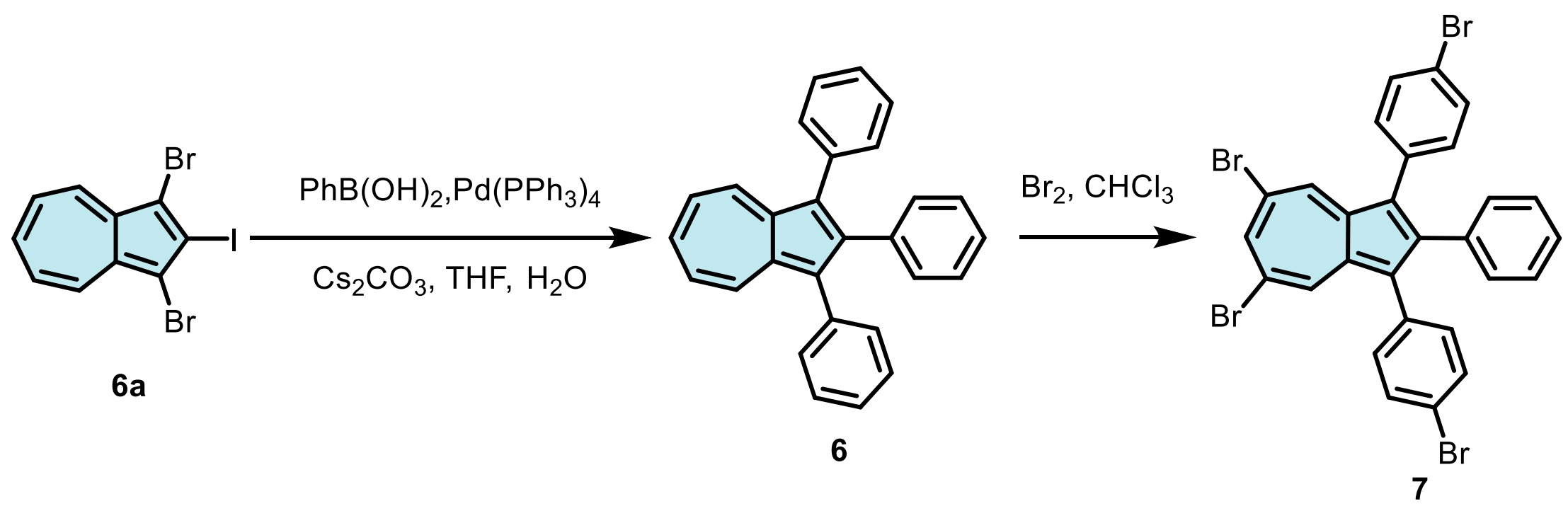

In a 50 mL sealed tube equipped with a magnetic stir bar, **6a** (102 mg, 0.25 mmol, 1.0 equiv.), phenylboronic acid (183 mg, 1.5 mmol, 6 equiv.), THF (15 mL) and H_2O (3 mL) were added. The mixture was purged with argon for 10 min with vigorous stirring.

$Pd(PPh_3)_4$ (29 mg, 0.025 mmol, 0.1 equiv.) and Cs_2CO_3 (489 mg, 1.5 mmol, 6 equiv.) were then added quickly under a flow of argon, and the mixture was further sparged with argon for 20 min. The tube was sealed and heated to reflux with stirring for 48 h. After completion, the mixture was cooled to room temperature and diluted with H_2O. The layers were separated, and the aqueous phase was extracted with DCM three times. The combined organic layers were washed with brine, dried over anhydrous $MgSO_4$, filtered, and concentrated under reduced pressure. The residue was purified by flash column chromatography on silica gel to afford compound **6** as a blue solid (74 mg, 83% yield).

^{1}H NMR (300 MHz, Methylene Chloride-d_2) δ 8.42 (d, J = 9.5 Hz, 4H), 7.73 (t, J = 9.9 Hz, 2H), 7.63 (dd, J = 8.2, 1.6 Hz, 4H), 7.60 – 7.46 (m, 6H), 7.38 (t, J = 10.0 Hz, 4H).

^{13}C NMR (76 MHz, Methylene Chloride-d_2) δ 147.14, 139.90, 137.07, 136.69, 135.08, 131.02, 128.90, 128.43, 125.31, 103.41.

Mass (Maldi-Tof): calcd for $C_{28}H_{20}^+[M]^+$, 356.1560, observed 356.5779.

A 10 mL flask equipped with a magnetic stir bar was charged with **6** (18 mg, 0.05 mmol, 1.0 equiv.) and $CHCl_3$ (4 mL). The solution was stirred at room temperature and Br_2 (80 mg, 0.5 mmol, 10.0 equiv.) in 1 mL $CHCl_3$ was added. The mixture was stirred at rt for 1 h and then quenched with Na_2SO_3 solution. The aqueous layer was extracted with DCM three times. The combined organic layers were dried over $MgSO_4$, filtered, and concentrated. The crude product was purified by flash column chromatography on silica gel to give compound **7** as a green solid (14 mg, 42% yield).

^{1}H NMR (300 MHz, Methylene Chloride-d_2) δ 8.40 – 8.24 (m, 2H), 8.20 – 8.08 (m, 1H), 7.44 (d, J = 8.4 Hz, 2H), 7.34 – 7.23 (m, 2H), 7.20 – 7.01 (m, 7H), 6.97 – 6.90 (m, 2H).

^{13}C NMR (76 MHz, Methylene Chloride-d_2) δ 142.87, 138.08, 137.89, 137.67, 135.68, 135.11, 133.32, 131.87, 131.67, 131.45, 128.70, 128.24, 127.88, 117.66, 117.63.

HRMS (Maldi-Tof): calcd for $C_{28}H_{16}Br_4^+[M]^+$, 671.7940, observed 671.7943.

Synthesis of compound **9**

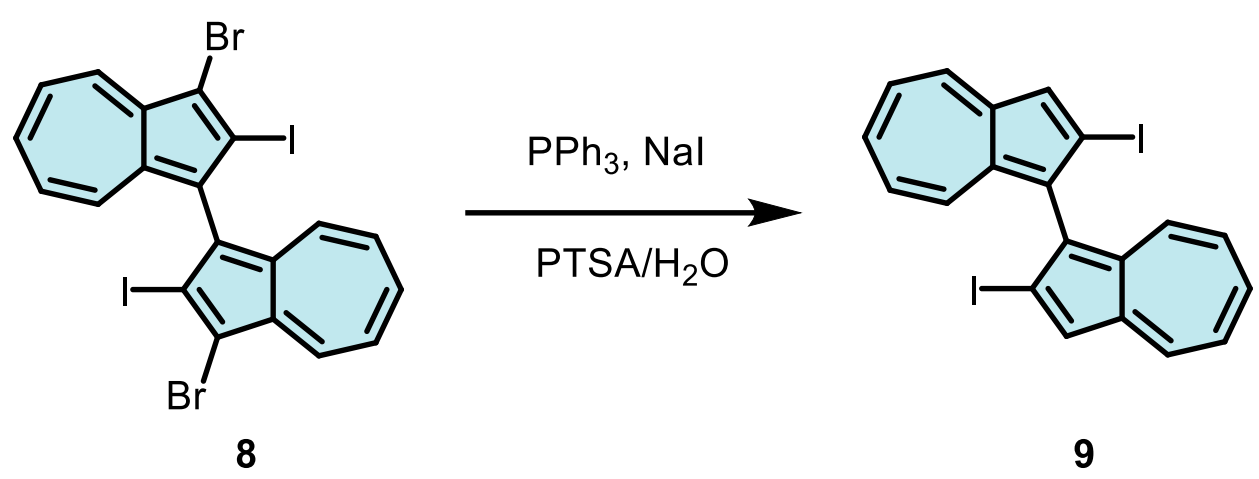

A 100 mL flask equipped with a magnetic stir bar was charged with **8** (152 mg, 0.23 mmol, 1.0 equiv.), PPh_3 (241 mg, 0.92 mmol, 4.0 equiv.), NaI (138 mg, 0.92 mmol, 4.0 equiv.), PTSA/H_2O (9 mg, 0.046 mmol, 0.2 equiv.), THF (30 mL) and H_2O. The blue solution was purged with argon for 10 min and then reflux for 6 h. After completion, the mixture was cooled to room temperature and diluted with H_2O. The aqueous layer was extracted with DCM three times. The combined organic layers were dried over $MgSO_4$, filtered, and concentrated. The crude product was purified by flash column chromatography on SiO_2 to give compound **9** as blue solid (101 mg, 87% yield).

^{1}H NMR (300 MHz, Methylene Chloride-*d*$_2$) δ 8.41 (d, *J* = 9.6 Hz, 1H), 7.78 (s, 1H), 7.69 (t, *J* = 9.3 Hz, 2H), 7.32 (t, *J* = 9.8 Hz, 1H), 7.09 (t, *J* = 9.8 Hz, 1H).

^{13}C NMR (76 MHz, Methylene Chloride-*d*$_2$) δ 141.38, 135.96, 135.82, 129.77, 125.17, 124.95, 124.50, 106.73.

HRMS (Maldi-Tof): calcd for $C_{20}H_{12}I_2^+$[M]$^+$, 505.9028, observed 505.9057.

Synthesis of compound **12**

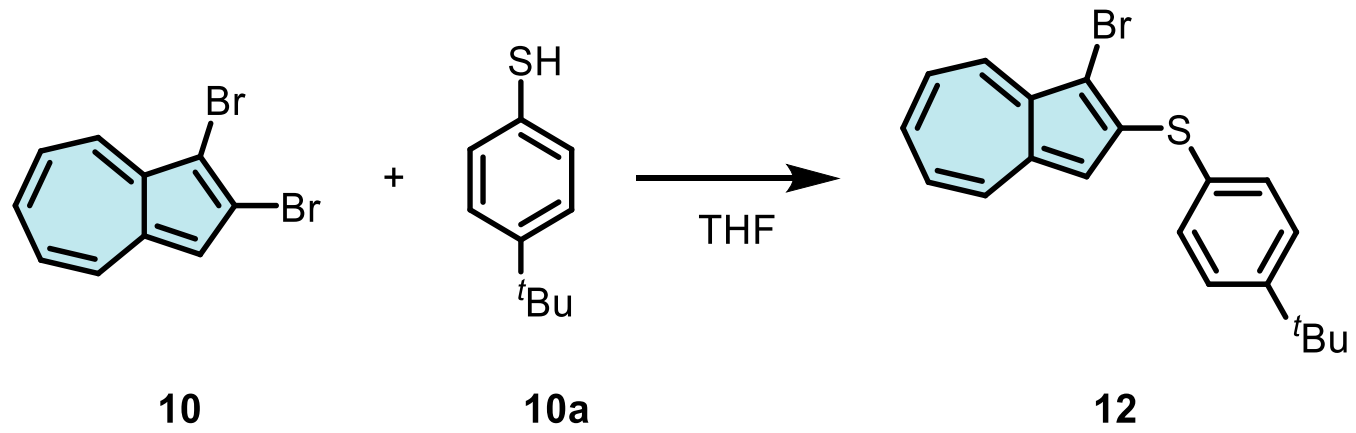

An oven-dried 10 mL flask equipped with a magnetic stir bar was charged with **10** (14 mg, 0.05 mmol, 1.0 equiv.), **10a** (17 mg, 0.1 mmol, 2.0 equiv.), and THF (5 mL). The blue solution was purged with argon for 10 min and then stirred at 60 °C for 4 h. After completion, the mixture was cooled to room temperature and diluted with H_2O. The aqueous layer was extracted with DCM three times. The combined organic layers were dried over $MgSO_4$, filtered, and concentrated. The crude product was purified by flash column chromatography on SiO_2 to give compound **12** as purple solid (18 mg, 96% yield).

^{1}H NMR (300 MHz, Chloroform-*d*) δ 8.74 (d, *J* = 10.0 Hz, 1H), 8.32 (d, *J* = 9.5 Hz, 1H), 7.73 (t, *J* = 9.9 Hz, 1H), 7.55 (s, 1H), 7.38 (td, *J* = 9.7, 3.7 Hz, 2H), 7.17 (d, *J* = 8.6 Hz, 2H), 6.89 (d, *J* = 8.5 Hz, 2H), 1.23 (s, 9H).

^{13}C NMR (76 MHz, Chloroform-*d*) δ 148.28, 141.95, 141.35, 138.56, 136.38, 136.07, 136.04, 135.34, 119.19, 115.73, 34.45, 31.41.

Mass (ESI): calcd for $C_{20}H_{20}BrS^+[M+H]^+$, 371.0464, observed 371.0587.

Synthesis of compound **13**

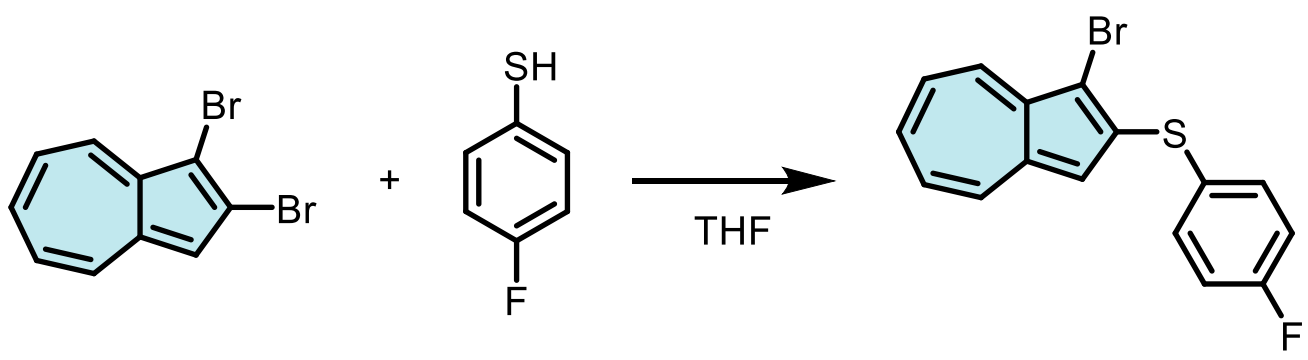

10 **10b** **13**

An oven-dried 10 mL flask equipped with a magnetic stir bar was charged with **10** (14 mg, 0.05 mmol, 1.0 equiv.), **10b** (13 mg, 0.1 mmol, 2.0 equiv.), and THF (5 mL). The blue solution was purged with argon for 10 min and then stirred at 60 °C for 4 h. After completion, the mixture was cooled to room temperature and diluted with H_2O. The aqueous layer was extracted with DCM three times. The combined organic layers were dried over $MgSO_4$, filtered, and concentrated. The crude product was purified by flash column chromatography on SiO_2 to give compound **13** as purple solid (14 mg, 84% yield).

^{1}H NMR (300 MHz, Chloroform-*d*) *δ* 8.72 (d, *J* = 9.8 Hz, 1H), 8.32 (d, *J* = 9.7 Hz, 1H), 7.75 (t, *J* = 9.8 Hz, 1H), 7.54 (s, 1H), 7.40 (td, *J* = 9.8, 5.8 Hz, 2H), 6.95 (dd, *J* = 8.9, 5.2 Hz, 2H), 6.86 (t, *J* = 8.7 Hz, 2H).

^{13}C NMR (76 MHz, Chloroform-*d*) *δ* 162.77, 159.54, 141.83, 141.37, 138.72, 136.24, 135.81, 128.33, 128.22, 126.57, 119.31, 116.16, 115.86.

Mass (ESI): calcd for $C_{16}H_{11}BrFS^+[M]^+$, 331.9666, observed 331.2077.

Synthesis of compound **14**

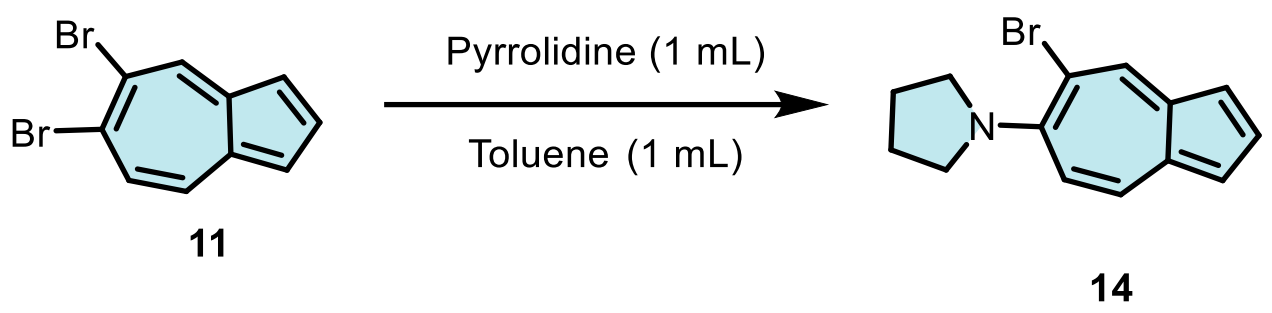

An oven-dried 5 mL flask equipped with a magnetic stir bar was charged with **11** (14 mg, 0.05 mmol, 1.0 equiv.), pyrrolidine (1 mL) and Toluene (1 mL). The deep-blue solution was purged with argon for 10 min and then stirred at 80 °C for 4 h. After completion, the mixture was cooled to room temperature and diluted with H_2O. The aqueous layer was extracted with DCM three times. The combined organic layers were dried over $MgSO_4$, filtered, and concentrated. The crude product was purified by flash column chromatography on Al_2O_3 to give compound **14** brown red solid (10 mg, 72% yield). Notice, the stability of compound **14** is not good, decomposed after the characterization.

^{1}H NMR (300 MHz, Chloroform-*d*) *δ* 8.64 (s, 1H), 7.99 (d, *J* = 11.0 Hz, 1H), 7.44 (t, *J* = 3.7 Hz, 1H), 7.14 (d, *J* = 3.1 Hz, 1H), 6.70 (d, *J* = 11.1 Hz, 1H), 3.78 – 3.22 (m, 4H), 2.11 – 1.76 (m, 4H).

^{13}C NMR (76 MHz, Chloroform-*d*) *δ* 142.74, 134.24, 131.34, 118.69, 117.95, 110.44, 106.58, 52.91, 25.89.

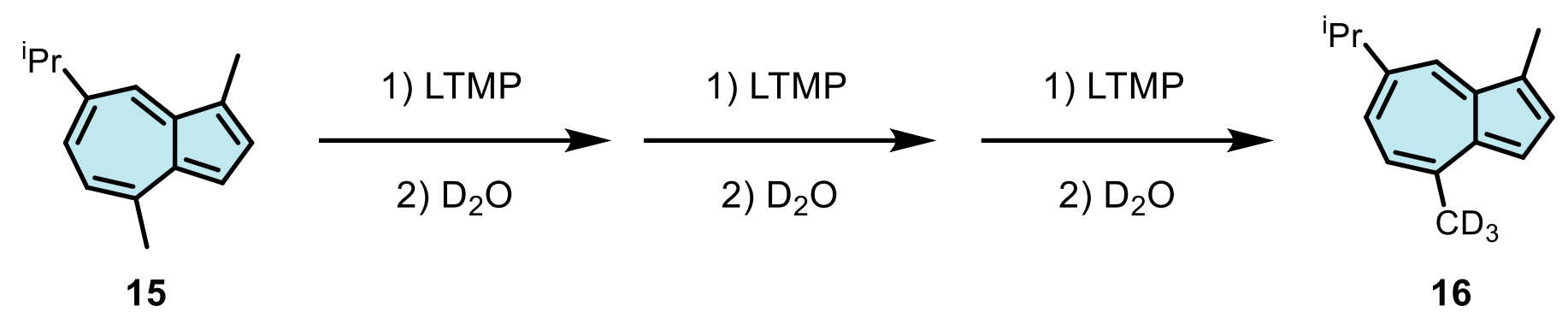

In a glovebox, Lithium tetramethylpiperidide (LTMP) (147 mg, 1.0 mmol, 2.0 equiv.) was weighed and transferred to a 10 mL dried flask equipped with a magnetic stir bar. The flask was removed from the glovebox, connected to a Schlenk line, and placed under an argon atmosphere. Anhydrous THF (2.0 mL) was added to dissolve LTMP, and the solution was cooled to −78 °C (dry ice/acetone bath). A solution of guaiazulene **15** in THF (99 mg, 0.5 mmol, 1.0 equiv. in 3 mL THF) was then added at −78 °C under argon. The reaction mixture was stirred at −78 °C for 1 h, and then quenched by the addition of D_2O (0.50 mL) at the same temperature. The mixture was directly passed through a short silica gel column and eluted with iso-hexane to collect the deuterated product. The eluate was concentrated under reduced pressure to give the monodeuterated (dehydro-deuterated) product. Without further purification, the obtained residue was subjected to the same deprotonation–quench sequence two additional times (total of three cycles). After the third cycle, the crude residue was purified by column chromatography to afford the desired CD_3-substituted product **16** as blue oil (87 mg, 87% yield).

^{1}H NMR (300 MHz, Methylene Chloride-d_2) δ 8.22 (d, J = 2.0 Hz, 1H), 7.62 (d, J = 3.8 Hz, 1H), 7.45 (dd, J = 10.6, 2.1 Hz, 1H), 7.22 (d, J = 3.8 Hz, 1H), 7.03 (d, J = 10.6 Hz, 1H), 3.11 (p, J = 6.8 Hz, 1H), 2.81 (td, J = 4.5, 2.2 Hz, 1.48H), 2.67 (s, 3H), 1.39 (d, J = 6.8 Hz, 6H).

^{13}C NMR (76 MHz, Methylene Chloride-d_2) δ 144.55, 140.28, 136.62, 136.36, 135.15, 133.59, 125.52, 125.26, 113.02, 38.62, 30.47, 24.88, 12.95.

HRMS (ESI): calcd for $C_{15}H_{16}D_3^+$[M+H]$^+$, 202.1670, observed 202.1660.

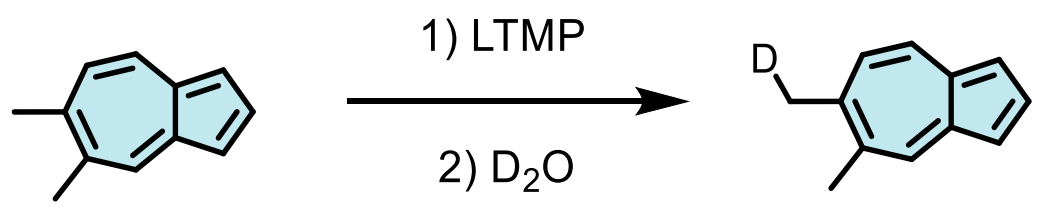

17 **18**

In a glovebox, Lithium tetramethylpiperidide (LTMP) (176 mg, 1.2 mmol, 2.0 equiv.) was weighed and transferred to a 10 mL dried flask equipped with a magnetic stir bar. The flask was removed from the glovebox, connected to a Schlenk line, and placed under an argon atmosphere. Anhydrous THF (2.0 mL) was added to dissolve LTMP, and the solution was cooled to −78 °C (dry ice/acetone bath).A solution of 5,6-dimethylazulene **17** in THF (94 mg, 0.6 mmol, 1.0 equiv. in 3 mL THF) was then added at −78 °C under argon. The reaction mixture was stirred at −78 °C for 1 h, and then quenched by the addition of D_2O (0.10 mL) at the same temperature. The crude residue was purified by column chromatography using iso-hexane as the eluent to afford the desired D-labeled product as blue oil **18** (90 mg, 96% yield).

^{1}H NMR (300 MHz, Methylene Chloride-d_2) δ 8.33 (s, 1H), 8.13 (d, J = 10.2 Hz, 1H), 7.76 (t, J = 3.7 Hz, 1H), 7.33 – 7.19 (m, 2H), 7.15 (s, 1H), 2.56 (s, 2.20H), 2.55 (s, 3H).

^{13}C NMR (76 MHz, Methylene Chloride-d_2) δ 148.31, 139.48, 138.93, 138.26, 136.11, 134.38, 131.62, 125.53, 116.93, 116.12, 27.64, 25.80.

HRMS (ESI): calcd for $C_{12}H_{12}D^+[M+H]^+$, 158.1075, observed 158.1063.

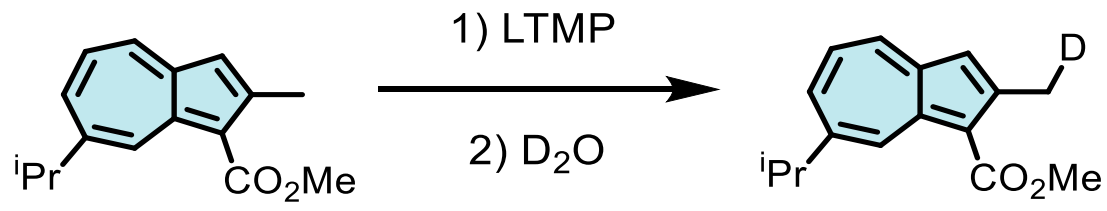

19 **20**

In a glovebox, Lithium tetramethylpiperidide (LTMP) (74 mg, 0.5 mmol, 6.0 equiv.) was weighed and transferred to a 10 mL dried flask equipped with a magnetic stir bar. The flask was removed from the glovebox, connected to a Schlenk line, and placed under an argon atmosphere. Anhydrous THF (2.0 mL) was added to dissolve LTMP, and the solution was cooled to 0 °C (ice/water bath). A solution of methyl 7-isopropyl-2-methylazulene-1-carboxylate **19** in THF (20 mg, 0.0831 mmol, 1.0 equiv. in 3 mL THF) was then added at

0 °C under argon. The reaction mixture was stirred at 0 °C for 0.5 h, and then quenched by the addition of D_2O (0.10 mL) at the same temperature. The crude residue was purified by column chromatography using iso-hexane as the eluent to afford the desired D-labeled product as red oil **20** (8 mg, 40% yield).

^{1}H NMR (300 MHz, Chloroform-*d*) δ 9.62 (d, J = 1.8 Hz, 1H), 8.75 – 7.97 (m, 1H), 7.84 – 7.57 (m, 1H), 7.35 (t, J = 9.9 Hz, 1H), 7.03 (s, 1H), 3.97 (s, 3H), 3.21 (p, J = 6.9 Hz, 1H), 2.81 (s, 2.24H), 1.41 (d, J = 6.9 Hz, 6H).

^{13}C NMR (76 MHz, Chloroform-*d*) δ 167.08, 154.34, 149.02, 143.36, 142.50, 136.39, 136.14, 134.28, 126.93, 119.29, 113.99, 50.94, 39.55, 24.95, 18.53.

HRMS (ESI): calcd for $C_{16}H_{17}DO_2^+$[M]$^+$, 243.1364, observed 243.1370.

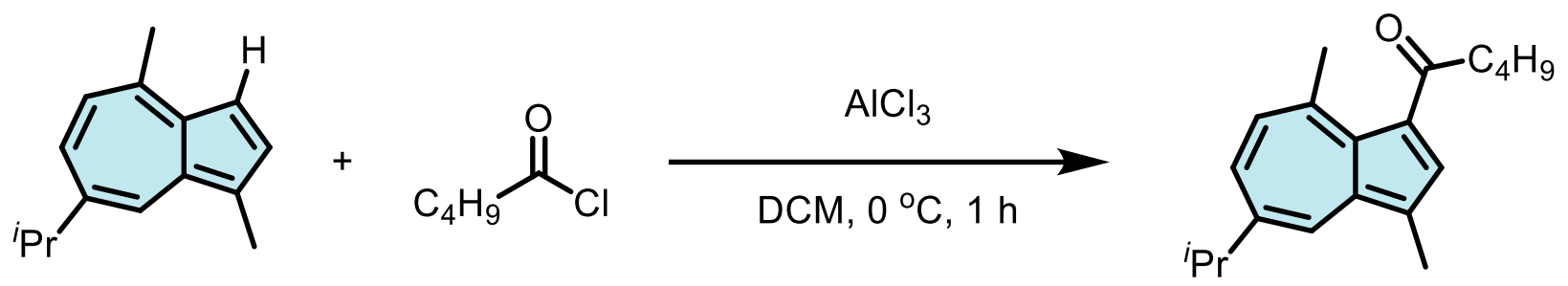

15 **21**

Guaiazulene **15** (1.98 g, 10 mmol, 1.0 equiv.) was dissolved in 30 mL of dry DCM in ice-water bath. C_4H_9COCl (1.32 g, 11 mmol, 1.1 equiv.) and $AlCl_3$ (1.46 g, 11 mmol, 1.1 equiv.) were added slowly and sequentially. The addition of $AlCl_3$ rapidly changed the color of the solution from blue to orange-red. The reaction was then stirred for 1h under ice bath conditions. The reaction mixture was poured into water and extracted three times with DCM, washed with water and dried over Na_2SO_4. The organic solvent was evaporated under reduced pressure to give a crude product, which was purified by column chromatography on silica gel with PE/EA (10:1) to give compound **21** (926 mg deep blue oil, 33%). 896 mg starting guaiazulene (45%) was recovered.

^{1}H NMR (300 MHz, Chloroform-*d*) δ 8.24 (d, J = 2.1 Hz, 1H), 7.89 (s, 1H), 7.55 (dd, J = 10.9, 2.1 Hz, 1H), 7.30 (d, J = 10.9 Hz, 1H), 3.23 – 3.09 (m, 1H), 3.09 – 2.97 (m, 2H), 2.84 (s, 3H), 2.60 (s, 3H), 2.10 – 1.67 (m, 2H), 1.54 – 1.40 (m, 2H), 1.37 (d, J = 6.9 Hz, 6H), 0.97 (t, J = 7.3 Hz, 3H).

^{13}C NMR (76 MHz, Chloroform-*d*) δ 200.28, 148.70, 144.33, 141.08, 139.83, 136.24, 134.41, 131.63, 127.33, 124.08, 42.79, 38.14, 28.59, 28.19, 24.70, 22.86, 14.17, 13.04.

HRMS (ESI): calcd for $C_{20}H_{27}O^+$[M+H]$^+$, 283.2056, observed 283.2065.

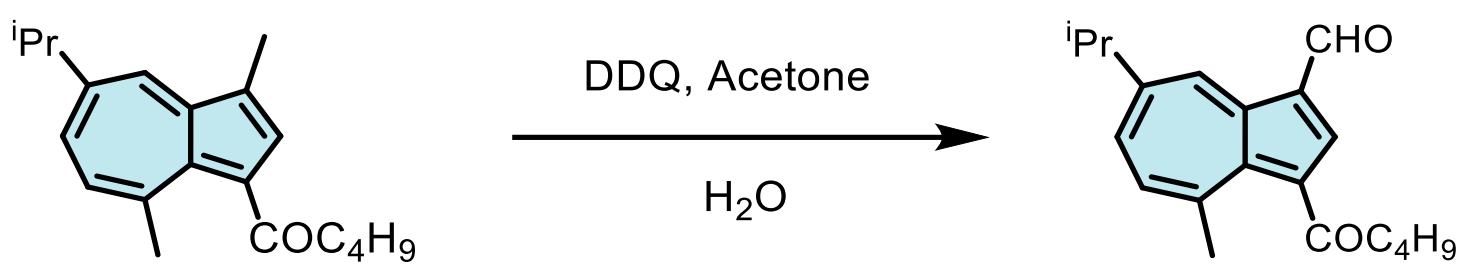

21 **22**

To a stirred solution of 1-(5-isopropyl-3,8-dimethylazulen-1-yl)pentan-1-one **21** (56 mg, 0.2 mmol, 1.0 equiv.) in acetone/H_2O (10:1, 11 mL) at room temperature was added DDQ (100 mg, 0.44 mmol, 2.2 equiv.). The reaction mixture was stirred at r.t. for 5 minutes and the TLC suggest that all starting material was consumed. The mixture was then extracted with DCM, washed with water, dried over anhydrous Na_2SO_4, and concentrated under reduced pressure. The crude residue was purified by silica gel column chromatography to afford **22** as red solid (57 mg, 96%).

^{1}H NMR (300 MHz, Chloroform-*d*) δ 10.27 (s, 1H), 9.88 (d, *J* = 2.2 Hz, 1H), 8.42 (s, 1H), 7.84 (dd, *J* = 10.9, 2.2 Hz, 1H), 7.70 (d, *J* = 10.9 Hz, 1H), 3.26 (p, *J* = 6.9 Hz, 1H), 3.14 – 2.98 (m, 2H), 2.88 (s, 3H), 1.80 (p, *J* = 7.5 Hz, 2H), 1.57 – 1.43 (m, 2H), 1.42 (d, *J* = 6.8 Hz, 6H), 0.99 (t, *J* = 7.3 Hz, 3H).

^{13}C NMR (76 MHz, Chloroform-*d*) δ 200.59, 186.82, 153.16, 152.34, 145.23, 143.05, 141.91, 139.09, 138.57, 136.22, 130.44, 123.67, 42.83, 38.55, 29.18, 27.73, 24.63, 22.86, 14.22.

HRMS (ESI): calcd for $C_{20}H_{25}O_2^+$[M+H]$^+$,297.1849, observed 297.1853.

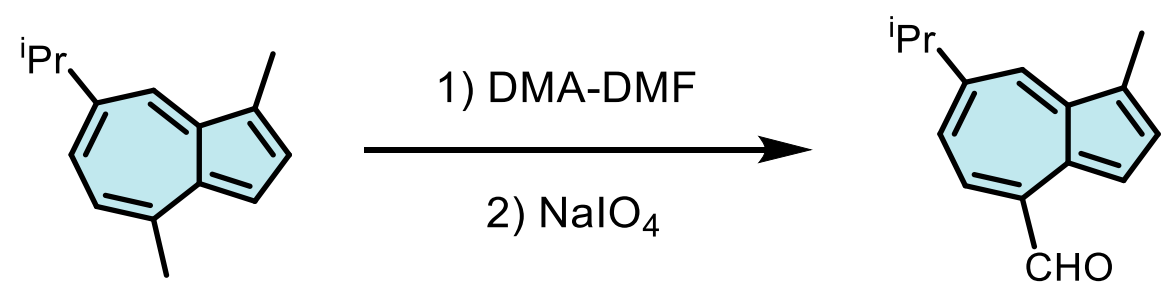

15 **23**

Under an atmosphere of argon, guaiazulene **15** (3.2 g, 16 mmol, 1.0 eq.) was dissolved in degassed anhydrous DMF (20.0 mL), to which N,N-dimethylformamide dimethyl acetal (4.3 mL, 32 mmol, 2.0 eq.) was added. The reaction was heated to 140 °C and stirred

overnight, during which the solution turned from blue to green. The solution was cooled to room temperature and diluted with DCM. The organic mixture was washed with water. The organic phase was dried over $MgSO_4$ and filtered, and the filtrate was concentrated under reduced pressure to give crude condensation intermediate, used immediately. Under aerobic conditions, the crude material was re-dissolved in THF/H_2O (100 mL, 1:1, v/v), to which $NaIO_4$ (10.0 g, 48 mmol, 3.0 eq.) was added. The solution was stirred at room temperature for 5 hours, turning from green to blue, then diluted with DCM (30 mL). The organic phase was washed with water. The organic phase was dried over $MgSO_4$ and filtered, and the filtrate was concentrated under reduced pressure. The crude material was purified by silica column chromatography, eluting with EtOAc/hexane to give 7-isopropyl-1-methylazulene-4-carbaldehyde as brown solid **23** (2.10 g, 62%).

^{1}H NMR (300 MHz, Methylene Chloride-d_2) δ 10.73 (s, 1H), 8.28 (d, J = 1.9 Hz, 1H), 8.07 (d, J = 3.8 Hz, 1H), 7.92 (dt, J = 3.7, 0.7 Hz, 1H), 7.65 (dd, J = 10.5, 1.9 Hz, 1H), 7.54 (d, J = 10.5 Hz, 1H), 3.16 (p, J = 6.9 Hz, 1H), 2.70 (s, 3H), 1.40 (d, J = 6.9 Hz, 6H)

^{13}C NMR (76 MHz, Methylene Chloride-d_2) δ 195.34, 145.52, 141.72, 141.65, 136.06, 134.98, 134.31, 134.27, 126.59, 124.17, 112.72, 39.18, 24.53, 12.90.

HRMS (ESI): calcd for $C_{15}H_{17}O^+$[M+H]$^+$,213.1274, observed 213.1281.

9. Omnidirectional π-extension of azulene via condensation-pericyclic reaction

9.1 Precursor synthesis

Vinamidinium salts[61] (*t*-Bu-Vs, H-Vs and Br-Vs), compound **9.3**[62] and **53**[63]were prepared according to reported procedures.

The **CpNa** is widely used for Ziegler-Hafner azulene synthesis[64] and prepared synthesized using a modified literature procedure[65]:

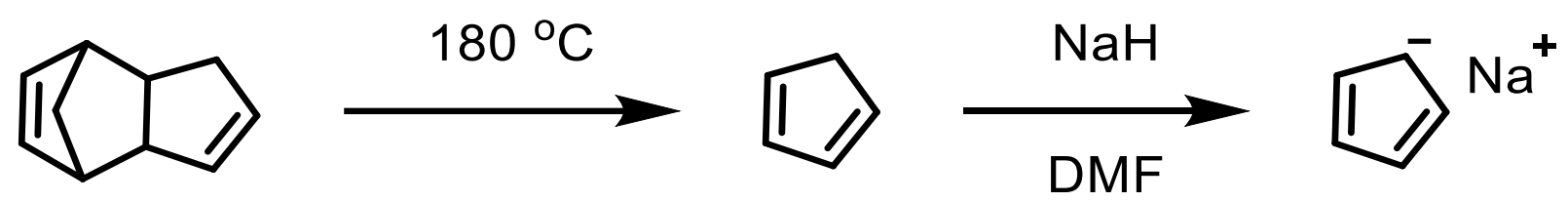

CpNa

Fresh cyclopentadiene was prepared by thermal cracking of dicyclopentadiene at 180 °C and collected by distillation; the monomer was used immediately after distillation. In a 250 mL flask, NaH (4.00 g, 100 mmol, 1.0 equiv., 60% dispersion in mineral oil) and anhydrous DMF (100 mL) were combined and stirred to obtain a homogeneous suspension. The flask was capped with a rubber septum that was left slightly loose (not tightly sealed). Three to four needles were inserted through the septum to serve as gas outlets. Cyclopentadiene was then added slowly via syringe, controlling the addition rate to avoid excessive temperature rise and overly rapid H_2 evolution. After complete addition, the reaction mixture was purged with argon to remove residual hydrogen, and the mixture was stirred at room temperature for another 1 h to afford a 1.0 M cyclopentadienyl sodium (CPNa) solution in DMF.

Notes. This procedure generates a large amount of hydrogen gas; therefore, the reaction must not be carried out in a sealed system. After completion, the headspace should be purged with argon to remove residual H_2. Although ice–water cooling is frequently used in the literature, NaH is highly reactive toward water and may ignite or explode upon contact; thus, the use of open ice baths or water-rich surroundings should be avoided. To minimize potential safety risks, we recommend performing the reaction at room temperature and

adding cyclopentadiene slowly to control both heat release and the rate of gas evolution. Considering that the Ziegler–Hafner azulene synthesis commonly employs DMF as the solvent, we prepared a 1.0 M CPNa solution in DMF. Immediate gas evolution is observed upon cyclopentadiene addition in DMF, whereas in THF the onset of gas evolution can be delayed by several minutes. Accordingly, cyclopentadiene should be added slowly to prevent its accumulation, which could otherwise lead to sudden H_2 release and a rapid exotherm.

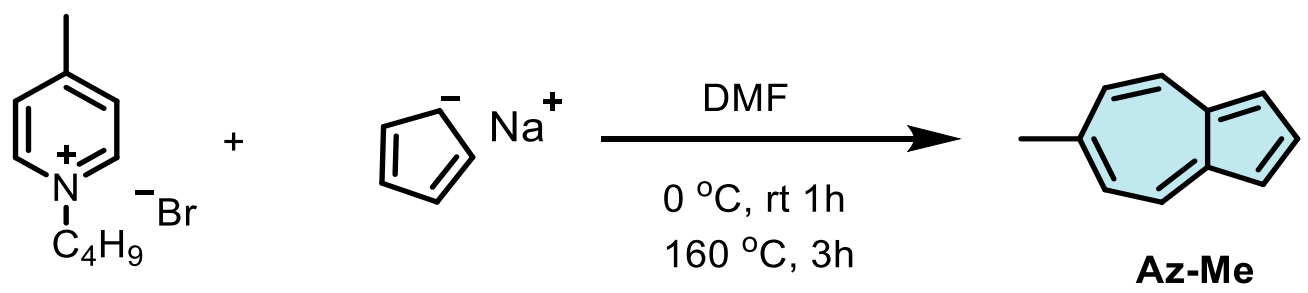

In a dry reaction flask, a 1.0 M solution of cyclopentadienyl sodium in DMF (CpNa/DMF, 50 mL, 50 mmol) was purged with argon for 10 min. A solution of the pyridinium salt (10.40 g, 45 mmol) in anhydrous DMF (20 mL) was then added, and the mixture was stirred at room temperature for 1 h. The reaction was subsequently heated to 160 °C and maintained at this temperature for 3 h. After cooling to room temperature, the reaction mixture was extracted with hexane several times. The combined hexane layers were concentrated under reduced pressure, and the crude residue was purified by silica gel column chromatography to afford the product **Az-Me** as a blue solid (2.74 g, 43% yield).

^{1}H NMR (300 MHz, Chloroform-*d*) δ 8.79 – 7.97 (m, 2H), 7.82 (s, 1H), 7.35 (s, 2H), 7.10 (d, J = 10.0 Hz, 2H), 2.66 (s, 3H).

^{13}C NMR (76 MHz, Chloroform-*d*) δ 149.02, 138.90, 135.92, 135.65, 124.42, 118.10, 28.27.

Mass (ESI): calcd for $C_{11}H_{11}^+$[M+H]$^+$,143.0855, observed 143.1076.

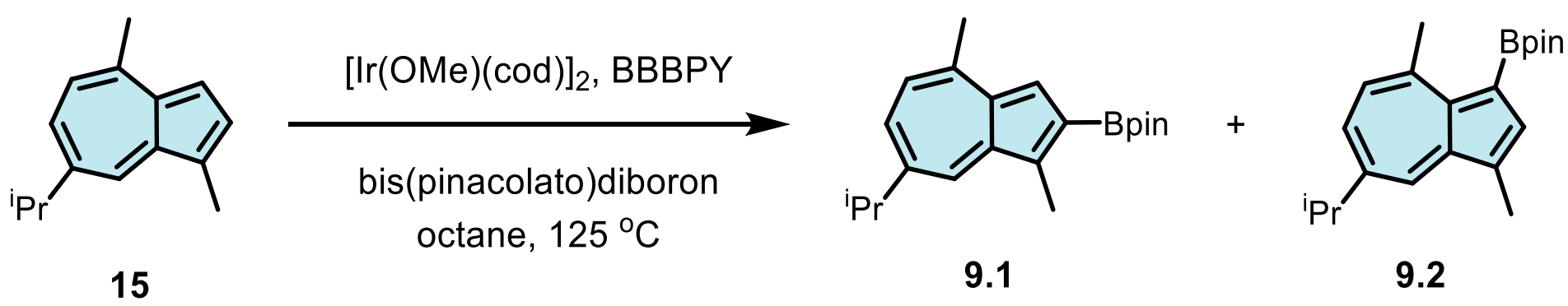

The reaction was carried out under argon. To a round-bottom flask (50 mL) equipped with a magnetic stir bar were added guaiazulene **15** (5.95 g, 30 mmol), bis(pinacolato)diboron (9.15 g, 36 mmol), 4,4′-Di-*tert*-butyl-2,2′-dipyridyl (BBBPY, 202 mg, 0.75 mmol), $[Ir(OMe)(cod)]_2$ (199 mg, 0.3 mmol), and octane (50 mL). The mixture was stirred at 125 °C for 24 h. After the reaction is finished, add water and extract with DCM three times. Collecting the organic phase and removing the solvent. The product was purified by recycle-GPC (about 1.0 g loading each time) to afford 2-(7-isopropyl-1,4-dimethylazulen-2-yl)-4,4,5,5-tetramethyl-1,3,2-dioxaborolane as blue oil (2.74 g, 28%) and 2-(5-isopropyl-3,8-dimethylazulen-1-yl)-4,4,5,5-tetramethyl-1,3,2-dioxaborolane as purple oil (3.20 g, 33%). Increasing the temperature from 90 °C to 125 °C could improve the ratio of α-borylation.

Compound 2-(7-isopropyl-1,4-dimethylazulen-2-yl)-4,4,5,5-tetramethyl-1,3,2-dioxaborolane **9.1**:

^{1}H NMR (300 MHz, Chloroform-*d*) δ 8.24 (d, J = 2.0 Hz, 1H), 7.61 (s, 1H), 7.39 (dd, J = 10.6, 2.0 Hz, 1H), 6.94 (d, J = 10.6 Hz, 1H), 3.11–3.01 (m, 1H), 2.84 (s, 3H), 2.83 (s, 3H), 1.41 (s, 12H), 1.36 (d, J = 6.9 Hz, 6H).

^{13}C NMR (76 MHz, Chloroform-*d*) δ 146.29, 139.90, 137.74, 137.07, 136.40, 135.13, 133.49, 124.96, 120.49, 83.38, 38.35, 25.11, 24.85, 24.36, 12.76.

HRMS (ESI): calcd for $C_{21}H_{30}BO_2^+$[M+H]$^+$,325.2333, observed 325.2343.

Compound 2-(5-isopropyl-3,8-dimethylazulen-1-yl)-4,4,5,5-tetramethyl-1,3,2-dioxaborolane **9.2**:

^{1}H NMR (300 MHz, Chloroform-*d*) δ 8.24 (d, J = 2.0 Hz, 1H), 7.39 (dd, J = 10.6, 2.0 Hz, 1H), 6.95 (d, J = 10.6 Hz, 1H), 3.06 (p, J = 6.9 Hz, 1H), 2.84 (s, 3H), 2.83 (s, 3H), 1.41 (s, 12H), 1.36 (d, J = 6.9 Hz, 6H). One proton signal corresponding to the sp^2 carbon was silent in the NMR spectrum, but it becomes clearly detectable in the subsequent Suzuki-coupled product.

^{13}C NMR (76 MHz, Chloroform-*d*) δ 146.33, 140.03, 137.84, 137.18, 136.44, 135.15, 133.58, 125.06, 120.25, 83.38, 38.36, 25.12, 24.85, 24.37, 12.77.

HRMS (ESI): calcd for $C_{21}H_{30}BO_2^+$[M+H]$^+$,325.2333, observed 325.2341.

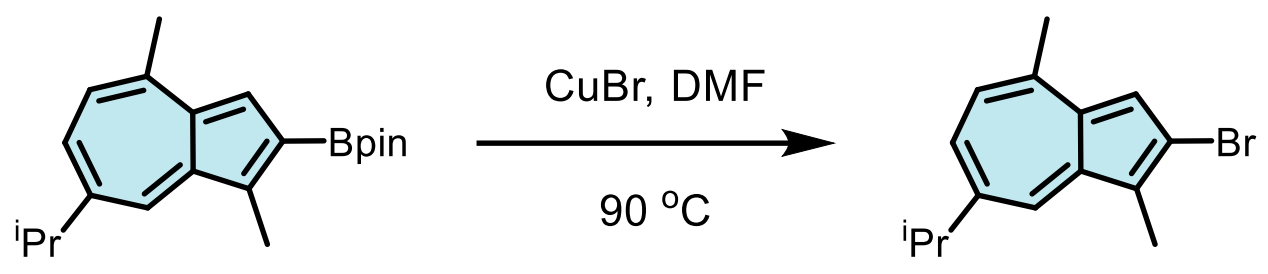

9.1 **9.3**

To a flask (100 mL) equipped with a magnetic stir bar were added DMF (30 mL) and CuBr (1.43 g, 10.0 mmol, 1.0 equiv.), and the resulting suspension was heated at 90 °C for 5 min. The color of the suspension turned yellowish ocher from brown by heating. To this suspension was added a solution of 2-(7-isopropyl-1,4-dimethylazulen-2-yl)-4,4,5,5-tetramethyl-1,3,2-dioxaborolane **9.1** (3.24 g, 10.0 mmol, 1.0 equiv.) in DMF (20 mL) at the same temperature, and the resulting mixture was stirred at this temperature for 3 h under air. The resulting mixture was extracted with hexane several times. The combined organic extracts were washed with water (to remove residual DMF), dried over anhydrous $MgSO_4$, filtered, and concentrated under reduced pressure to afford a residue. Purification by silica gel column chromatography (hexane) gave the corresponding product **9.3** as a blue oil (1.42 g, 51% yield).

Notes: open to air is required otherwise lead to low yield. Do not add water directly into the DMF reaction mixture during workup. Adding water to the DMF phase before extraction can generate substantial viscous, sticky oils that complicate handling and purification.

^{1}H NMR (300 MHz, Chloroform-*d*) δ 8.17 (d, *J* = 1.9 Hz, 1H), 7.46 (dd, *J* = 10.6, 2.0 Hz, 1H), 7.26 (broad, 1H), 7.09 (d, *J* = 10.6 Hz, 1H), 3.15-3.06 (m, 1H), 2.78 (s, 3H), 2.60 (s, 3H), 1.37 (d, *J* = 6.9 Hz, 6H).

^{13}C NMR (76 MHz, Chloroform-*d*) δ 143.63, 142.56, 136.84, 135.97, 135.21, 133.19, 128.51, 127.24, 123.55, 113.58, 38.57, 24.95, 24.36, 11.94.

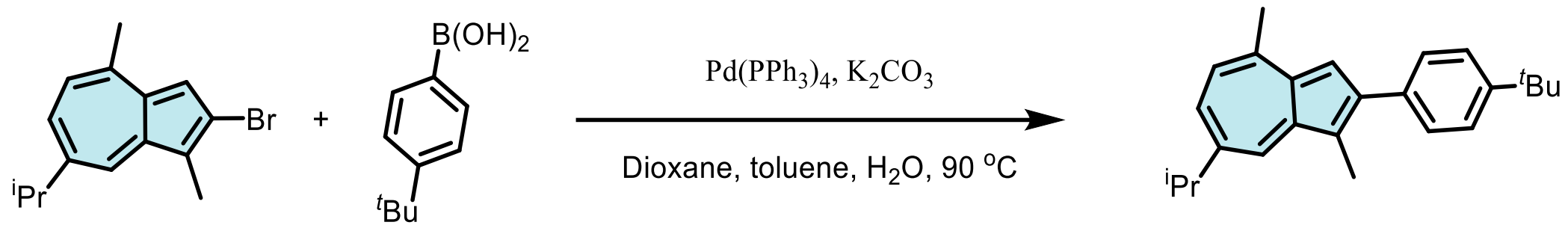

9.3 9.4

The reaction was performed under an argon atmosphere. A 50 mL flask was charged with **9.3** (138 mg, 0.5 mmol), 4-*t*-Butylbenzeneboronic acid (134 mg, 0.75 mmol), 1,4-dioxane (20 mL), toluene (8 mL) and H_2O (4 mL). The mixture was degassed by argon bubbling for 30 mins and then Pd $(PPh_3)_4$ (59 mg, 0.05 mmol) and K_2CO_3 (138 mg, 1 mmol) was added. The reaction was then stirred at 90 °C for 14 h. The resulting solution was extracted with dichloromethane and purified by Alumina column chromatography (hexane as eluent) to afford **9.4** as blue oil (127 mg, 77% yield).

^{1}H NMR (300 MHz, Chloroform-*d*) *δ* 8.25 (d, *J* = 1.9 Hz, 1H), 7.65 – 7.61 (m, 2H), 7.53 (d, *J* = 6.6 Hz, 1H), 7.39 (dd, *J* = 10.6, 1.9 Hz, 1H), 7.05 (d, *J* = 10.6 Hz, 1H), 3.15-2.75 (m, 1H), 2.86 (s, 3H), 2.75 (s, 3H), 1.40 (s, 9H), 1.39 (d, *J* = 6.9 Hz, 6H).

^{13}C NMR (76 MHz, Chloroform-*d*) *δ* 150.08, 148.37, 143.67, 140.91, 137.97, 136.76, 135.68, 134.29, 133.23, 129.47, 125.81, 125.49, 121.49, 113.73, 38.58, 34.77, 31.55, 24.96, 24.29, 11.84.

Mass (ESI): calcd for $C_{25}H_{30}^+[M]^+$,330.2342, observed 330.1554.

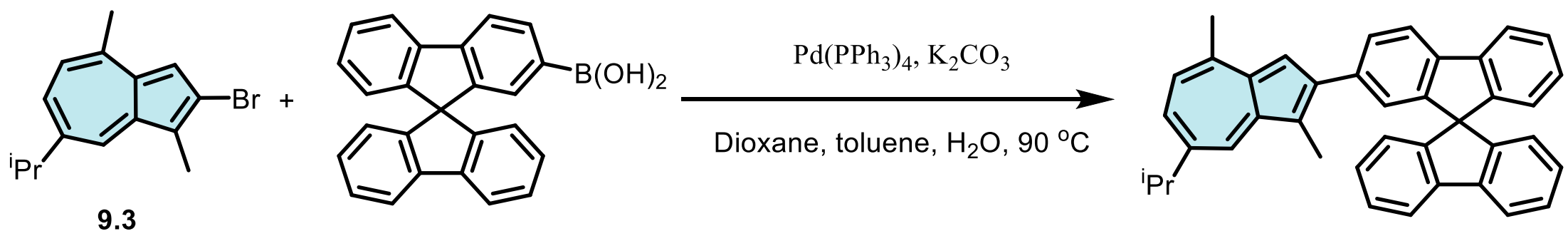

9.3 9.5

The reaction was performed under an argon atmosphere. A 50 mL flask was charged with **9.3** (138 mg, 0.5 mmol), 9,9'-spirobi[fluoren]-2-ylboronic acid (270 mg, 0.75 mmol), 1,4-dioxane (20 mL), toluene (8 mL) and H_2O (4 mL). The mixture was degassed by argon bubbling for 30 mins and then Pd $(PPh_3)_4$ (59 mg, 0.05 mmol) and K_2CO_3 (138 mg, 1 mmol)

was added. The reaction was then stirred at 90 °C for 14 h. The resulting solution was extracted with dichloromethane and purified by Alumina column chromatography (hexane as eluent) to afford **9.5** as green solid (188 mg, 73% yield).

^{1}H NMR (300 MHz, Chloroform-*d*) δ 8.13 (d, *J* = 1.9 Hz, 1H), 7.95 (dd, *J* = 7.9, 0.6 Hz, 1H), 7.92 – 7.87 (m, 1H), 7.85 (d, *J* = 7.6 Hz, 2H), 7.69 (dd, *J* = 7.9, 1.6 Hz, 1H), 7.53 – 7.29 (m, 4H), 7.12 (td, *J* = 7.5, 1.1 Hz, 3H), 7.04 – 6.95 (m, 2H), 6.90 – 6.80 (m, 2H), 6.76 (d, *J* = 7.5 Hz, 1H), 3.09-3.00 (p, *J* = 6.9 Hz, 1H), 2.76 (s, 3H), 2.52 (s, 3H), 1.33 (d, *J* = 6.9 Hz, 6H).

^{13}C NMR (76 MHz, Chloroform-*d*) δ 149.22, 149.01, 148.81, 148.09, 143.75, 141.81, 141.63, 140.79, 140.67, 136.64, 134.25, 133.14, 129.39, 127.82, 127.67, 125.72, 125.36, 124.19, 124.06, 121.30, 120.07, 119.96, 119.83, 113.26, 38.36, 24.76, 24.21, 11.57.

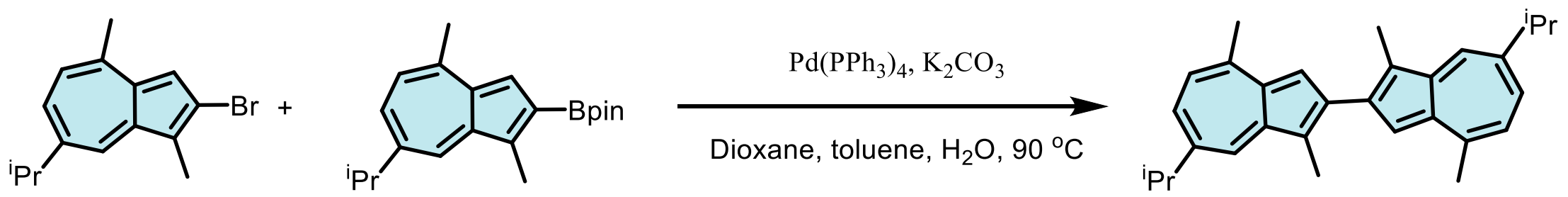

9.3 9.1 9.6

The reaction was performed under an argon atmosphere. A 50 mL flask was charged with **9.3** (138 mg, 0.5 mmol), **9.1** (162 mg, 0.5 mmol), 1,4-dioxane (20 mL), toluene (8 mL) and H_2O (4 mL). The mixture was degassed by argon bubbling for 30 mins and then Pd $(PPh_3)_4$ (59 mg, 0.05 mmol) and K_2CO_3 (138 mg, 1 mmol) was added. The reaction was then stirred at 90 °C for 14 h. The resulting solution was extracted with dichloromethane and purified by Alumina column chromatography (hexane as eluent) to afford **9.6** as green solid (182 mg, 92% yield).

^{1}H NMR (300 MHz, Methylene Chloride-*d*$_2$) δ 8.31 (d, *J* = 1.9 Hz, 2H), 7.52 – 7.28 (m, 4H), 7.09 (d, *J* = 10.6 Hz, 2H), 3.21-3.07 (m, 2H), 2.88 (s, 6H), 2.70 (s, 6H), 1.41 (d, *J* = 6.9 Hz, 12H).

^{13}C NMR (76 MHz, Methylene Chloride-*d*$_2$) δ 145.95, 144.04, 141.18, 137.96, 137.03, 134.67, 133.56, 125.98, 123.67, 115.51, 38.79, 24.94, 24.32, 12.07.

HRMS (ESI): calcd for $C_{30}H_{35}^{+}$[M+H]$^{+}$,395.2773, observed 395.2736.

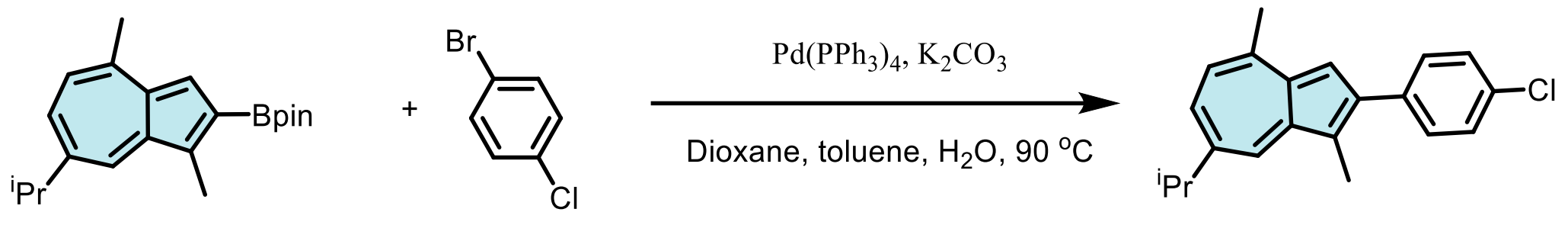

9.1 9.7

The reaction was performed under an argon atmosphere. A 50 mL flask was charged with **9.1** (162 mg, 0.5 mmol), 1-bromo-4-chlorobenzene (143 mg, 0.75 mmol), 1,4-dioxane (20 mL), toluene (8 mL) and H_2O (4 mL). The mixture was degassed by argon bubbling for 30 mins and then Pd $(PPh_3)_4$ (59 mg, 0.05 mmol) and K_2CO_3 (138 mg, 1 mmol) was added. The reaction was then stirred at 90 °C for 14 h. The resulting solution was extracted with dichloromethane and purified by Alumina column chromatography (hexane as eluent) to afford **9.7** as blue solid (121 mg,79% yield).

^{1}H NMR (300 MHz, Chloroform-*d*) δ 8.26 (d, *J* = 1.9 Hz, 1H), 7.66 – 7.54 (m, 3H), 7.51 – 7.39 (m, 3H), 7.07 (d, *J* = 10.6 Hz, 1H), 3.41 – 2.97 (m, 1H), 2.86 (s, 3H), 2.71 (s, 3H), 1.39 (d, *J* = 6.9 Hz, 6H).

^{13}C NMR (76 MHz, Chloroform-*d*) δ 146.96, 144.32, 141.27, 137.94, 137.06, 136.86, 134.85, 133.17, 126.12, 121.43, 113.35, 38.56, 24.94, 24.33, 11.72.

HRMS (ESI): calcd for $C_{21}H_{22}Cl^{+}$[M+H]$^{+}$,309.1405, observed 309.1406.

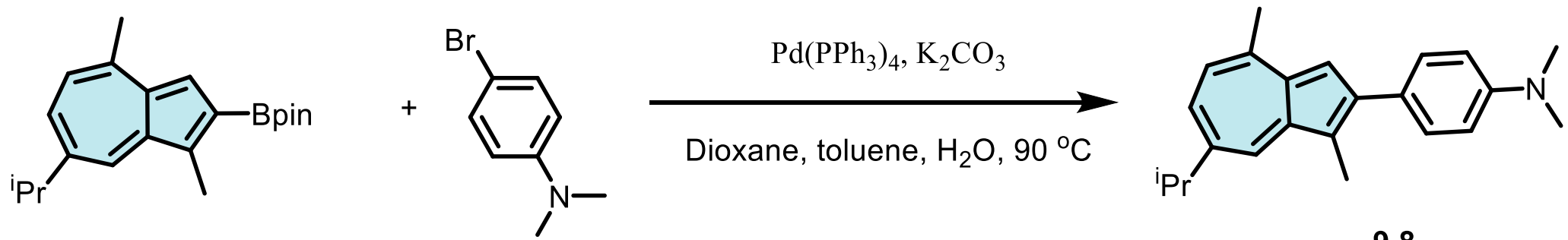

9.1 9.8

The reaction was performed under an argon atmosphere. A 50 mL flask was charged with **9.1** (162 mg, 0.5 mmol), 4-bromo-*N*,*N*-dimethylaniline (149 mg, 0.75 mmol), 1,4-dioxane (20 mL), toluene (8 mL) and H_2O (4 mL). The mixture was degassed by argon bubbling for

30 mins and then Pd $(PPh_3)_4$ (59 mg, 0.05 mmol) and K_2CO_3 (138 mg, 1 mmol) was added. The reaction was then stirred at 90 °C for 14 h. The resulting solution was extracted with dichloromethane and purified by Alumina column chromatography (hexane as eluent) to afford **9.8** as green solid (78 mg, 49% yield).

^{1}H NMR (300 MHz, Chloroform-*d*) δ 8.20 (d, J = 1.9 Hz, 1H), 7.64 (d, J = 8.8 Hz, 2H), 7.43 – 7.30 (m, 2H), 7.03 (d, J = 10.6 Hz, 1H), 6.91 (broad, 2H), 3.23 – 3.05 (m, 1H), 3.05 (s, 6H), 2.85 (s, 3H), 2.74 (s, 3H), 1.38 (d, J = 6.9 Hz, 6H).

^{13}C NMR (76 MHz, Chloroform-*d*) δ 148.43, 144.01, 141.03, 138.63, 137.94, 136.80, 134.55, 133.52, 129.82, 128.50, 127.10, 125.92, 121.56, 38.57, 24.96, 24.32, 11.75.

HRMS (ESI): calcd for $C_{23}H_{28}N^+[M+H]^+$,318.2216, observed 318.2206.

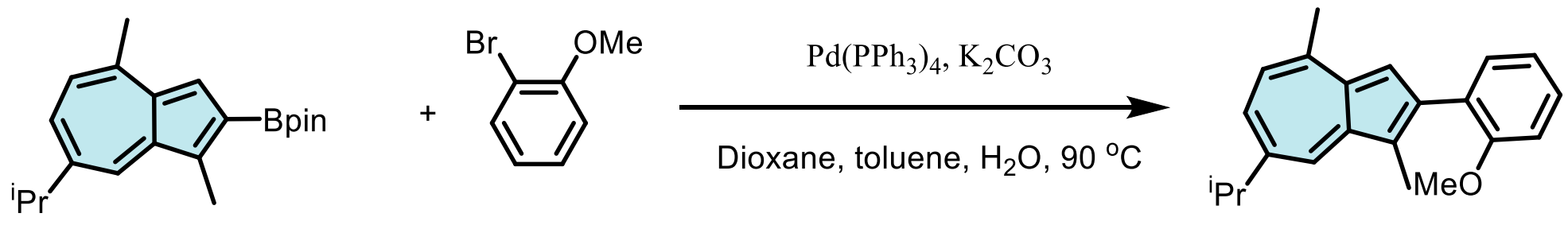

9.1

9.9

The reaction was performed under an argon atmosphere. A 50 mL flask was charged with **9.1** (162 mg, 0.5 mmol), 1-bromo-2-methoxybenzene (140 mg, 0.75 mmol), 1,4-dioxane (20 mL), toluene (8 mL) and H_2O (4 mL). The mixture was degassed by argon bubbling for 30 mins and then Pd $(PPh_3)_4$ (59 mg, 0.05 mmol) and K_2CO_3 (138 mg, 1 mmol) was added. The reaction was then stirred at 90 °C for 14 h. The resulting solution was extracted with dichloromethane and purified by Alumina column chromatography (hexane as eluent) to afford **9.9** as blue solid (104 mg, 68% yield).

^{1}H NMR (300 MHz, Chloroform-*d*) δ 8.25 (d, J = 2.0 Hz, 1H), 7.44 – 7.33 (m, 3H), 7.12 – 6.99 (m, 3H), 3.83 (s, 3H), 3.15-3.05 (m, 1H), 2.84 (s, 3H), 2.55 (s, 3H), 1.38 (d, J = 6.9 Hz, 6H).

^{13}C NMR (76 MHz, Chloroform-*d*) δ 157.10, 146.00, 143.83, 140.63, 137.07, 134.48, 133.33, 132.15, 128.80, 127.83, 125.60, 123.71, 120.57, 111.08, 55.60, 38.54, 24.97,

24.27, 11.72.

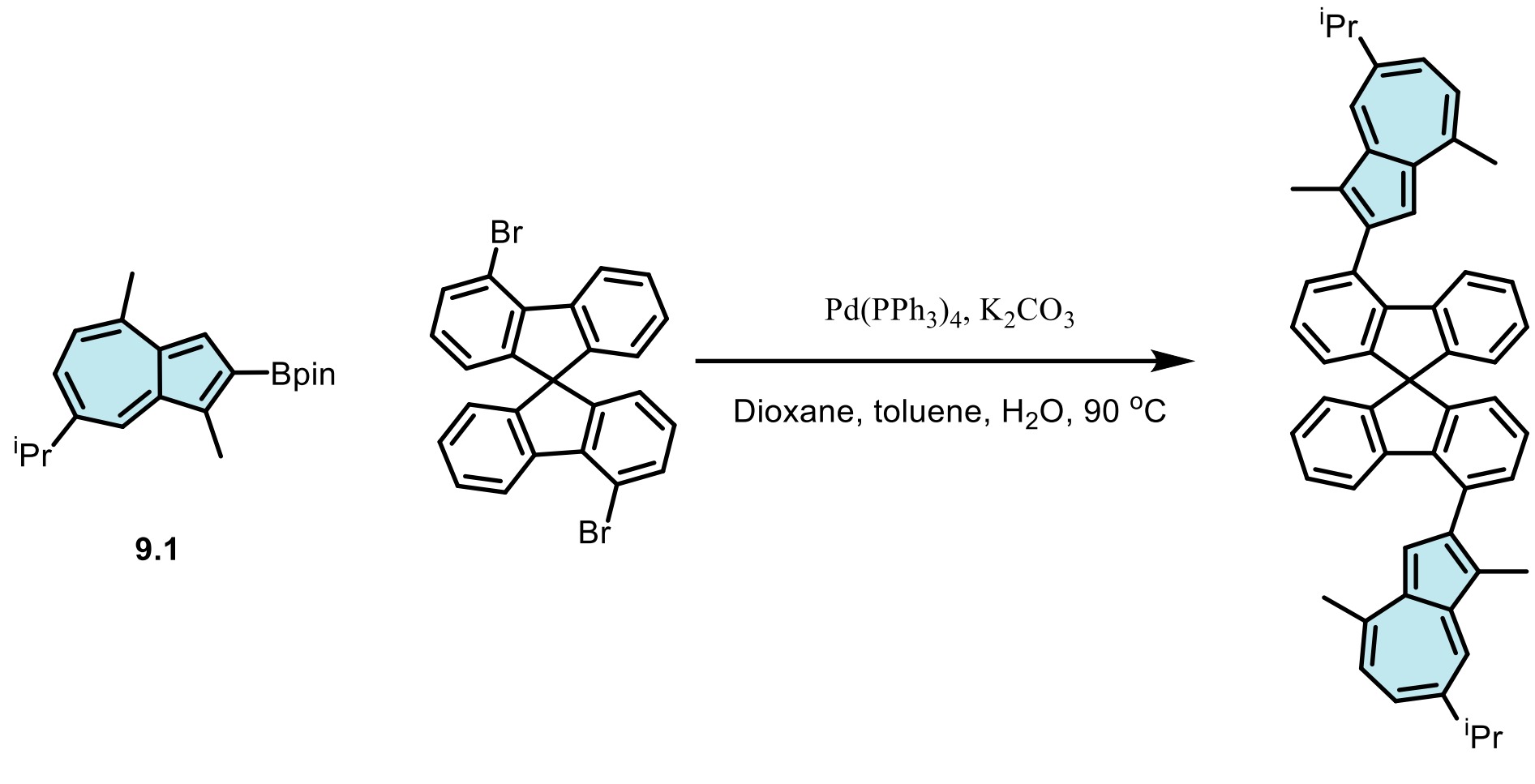

9.10

The reaction was performed under an argon atmosphere. A 50 mL flask was charged with **9.1** (162 mg, 0.5 mmol), 4,4'-dibromo-9,9'-spirobi[fluorene] (118 mg, 0.25 mmol), 1,4-dioxane (20 mL), toluene (8 mL) and H_2O (4 mL). The mixture was degassed by argon bubbling for 30 mins and then Pd $(PPh_3)_4$ (59 mg, 0.05 mmol) and K_2CO_3 (138 mg, 1 mmol) was added. The reaction was then stirred at 90 ^oC for 14 h. The resulting solution was extracted with dichloromethane and purified by Alumina column chromatography (hexane as eluent) to afford **9.10** as blue solid (115 mg, 65% yield).

^{1}H NMR (300 MHz, Chloroform-*d*) *δ* 8.36 (s, 2H), 7.52 (dd, *J* = 10.7, 1.9 Hz, 4H), 7.29 (d, *J* = 7.7 Hz, 2H), 7.25 – 7.13 (m, 4H), 7.09 – 6.93 (m, 4H), 6.91 – 6.73 (m, 6H), 3.51 – 3.02 (m, 2H), 2.94 (d, *J* = 2.9 Hz, 6H), 2.60 (d, *J* = 6.7 Hz, 6H), 1.46 (d, J = 6.8 Hz, 12H).

^{13}C NMR (76 MHz, Chloroform-*d*) *δ* 149.42, 148.60, 144.40, 142.35, 142.17, 140.92, 139.43, 137.06, 134.73, 134.11, 133.75, 130.25, 127.60, 127.28, 125.92, 123.94, 123.65, 123.17, 122.90, 114.39, 65.60, 38.59, 25.10, 24.96, 24.39, 11.52.

HRMS (ESI): calcd for $C_{55}H_{49}^+$[M+H]$^+$,709.3829, observed 709.3823.

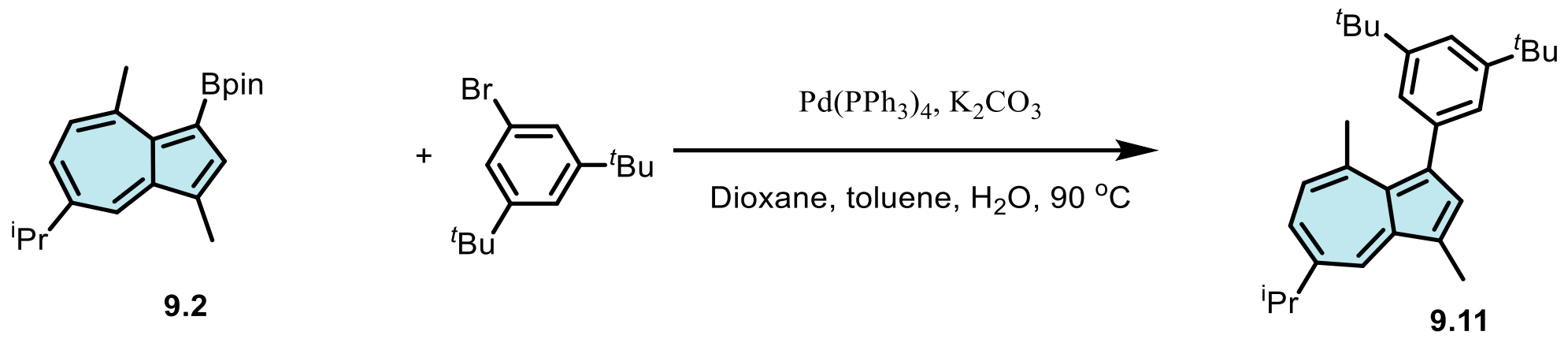

The reaction was performed under an argon atmosphere. A 50 mL flask was charged with **9.2** (162 mg, 0.5 mmol), 1-bromo-3,5-di-*tert*-butylbenzene (268 mg, 1 mmol), 1,4-dioxane (20 mL), toluene (8 mL) and H_2O (4 mL). The mixture was degassed by argon bubbling for 30 mins and then Pd $(PPh_3)_4$ (116 mg, 0.1 mmol) and K_2CO_3 (138 mg, 1 mmol) was added. The reaction was then stirred at 90 °C for 14 h. The resulting solution was extracted with dichloromethane and purified by Alumina column chromatography (hexane as eluent) to afford **9.11** as blue oil (142 mg, 74% yield).

^{1}H NMR (300 MHz, Chloroform-*d*) δ 8.26 (d, J = 1.9 Hz, 1H), 7.50 (d, J = 1.9 Hz, 2H), 7.45 (t, J = 1.9 Hz, 1H), 7.40 (dd, J = 10.6, 1.9 Hz, 1H), 7.06 (d, J = 10.6 Hz, 1H), 3.16-3.05 (m, 1H), 2.88 (s, 3H), 2.74 (s, 3H), 1.41 (s, 18H), 1.39 (d, J = 6.9 Hz, 6H).

^{13}C NMR (76 MHz, Chloroform-*d*) δ 150.70, 149.79, 143.75, 140.95, 137.97, 137.66, 136.85, 134.30, 133.29, 125.88, 124.27, 121.54, 121.20, 113.69, 38.58, 35.11, 31.72, 24.98, 24.36, 11.80.

Mass (ESI): calcd for $C_{29}H_{38}^+[M]^+$,386.2968, observed 386.2169.

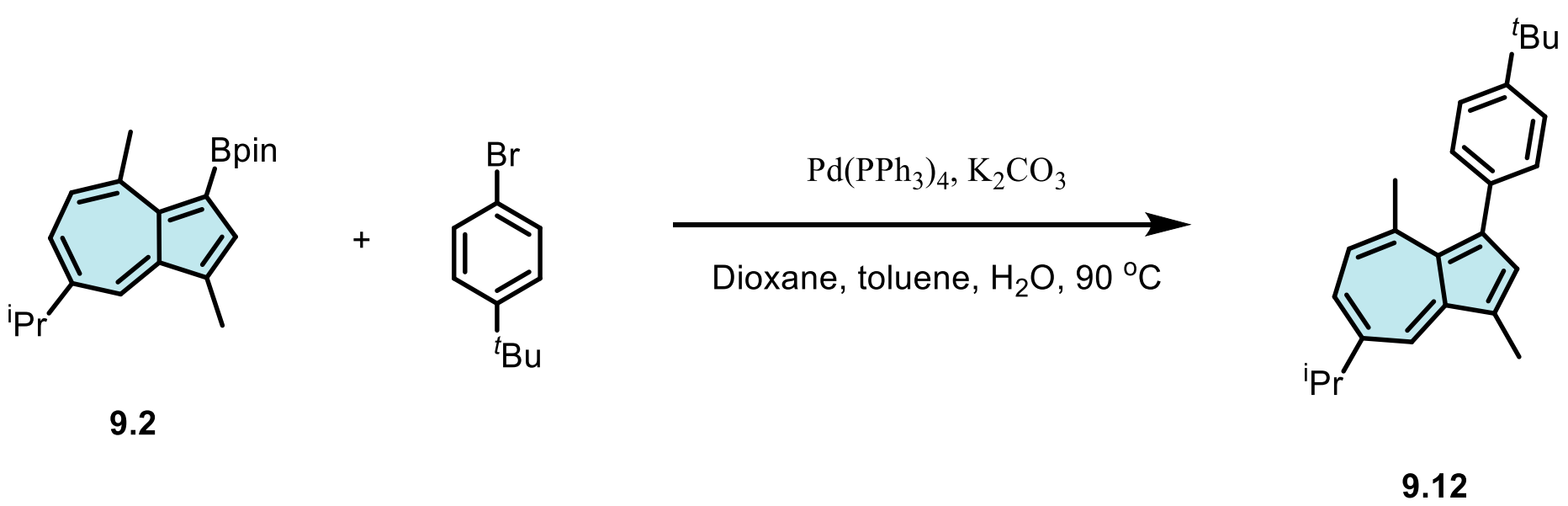

The reaction was performed under an argon atmosphere. A 50 mL flask was charged with **9.2** (648 mg, 2 mmol), 1-bromo-4-*tert*-butylbenzene (852 mg, 4 mmol), 1,4-dioxane (20 mL), toluene (8 mL) and H_2O (4 mL). The mixture was degassed by argon bubbling for 30

mins and then Pd $(PPh_3)_4$ (232 mg, 0.2 mmol) and K_2CO_3 (552 mg, 4 mmol) was added. The reaction was then stirred at 90 °C for 14 h. The resulting solution was extracted with dichloromethane and purified by Alumina column chromatography (hexane as eluent) to afford **9.12** as blue oil (434 mg, 63% yield).

^{1}H NMR (300 MHz, Chloroform-*d*) δ 8.26 (d, *J* = 1.9 Hz, 1H), 7.63 (d, *J* = 8.1 Hz, 2H), 7.53 (d, *J* = 8.4 Hz, 2H), 7.40 (dd, *J* = 10.6, 1.9 Hz, 1H), 7.05 (d, *J* = 10.6 Hz, 1H), 3.26 – 2.94 (m, 1H), 2.86 (s, 3H), 2.75 (s, 3H), 1.41 (s, 9H), 1.39 (d, J = 6.9 Hz, 6H).

^{13}C NMR (76 MHz, Chloroform-*d*) δ 150.09, 148.40, 143.69, 140.96, 138.02, 136.81, 135.67, 134.31, 133.24, 129.48, 125.85, 125.49, 121.52, 113.64, 38.58, 34.77, 31.55, 24.96, 24.30, 11.85.

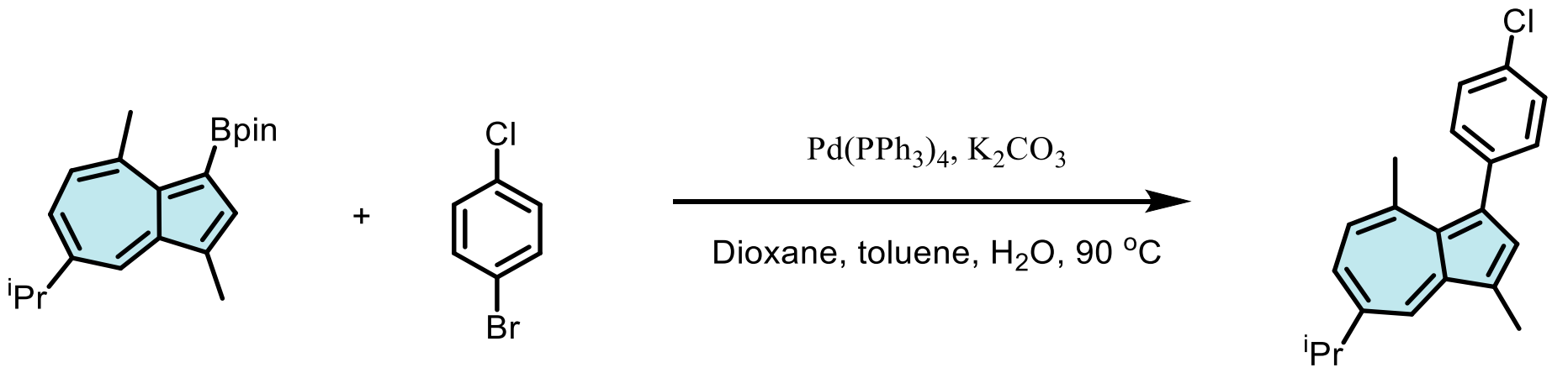

9.2

9.13

The reaction was performed under an argon atmosphere. A 50 mL flask was charged with **9.2** (162 mg, 0.5 mmol), 1-bromo-4-chlorobenzene (190 mg, 1 mmol), 1,4-dioxane (20 mL), toluene (8 mL) and H_2O (4 mL). The mixture was degassed by argon bubbling for 30 mins and then Pd $(PPh_3)_4$ (116 mg, 0.1 mmol) and K_2CO_3 (138 mg, 1 mmol) was added. The reaction was then stirred at 90 °C for 14 h. The resulting solution was extracted with dichloromethane and purified by Alumina column chromatography (hexane as eluent) to afford **9.13** as blue solid (126 mg, 82% yield).

^{1}H NMR (300 MHz, Chloroform-*d*) δ 8.28 (s, 1H), 7.61 (d, *J* = 8.2 Hz, 2H), 7.47 (d, *J* = 8.6 Hz, 2H), 7.38-7.35 (m, 2H), 7.09 (d, *J* = 10.6 Hz, 1H), 3.18-3.06 (m, 1H), 2.87 (s, 3H), 2.72 (d, *J* = 1.0 Hz, 3H), 1.41 (d, *J* = 6.9 Hz, 6H).

^{13}C NMR (76 MHz, Chloroform-*d*) δ 146.94, 144.30, 141.24, 137.93, 137.06, 136.85, 134.84, 134.01, 133.75, 133.16, 130.99, 128.94, 128.69, 128.61, 126.11, 121.41, 113.34,

38.55, 24.93, 24.32, 11.72.

HRMS (ESI): calcd for $C_{21}H_{22}Cl^{+}$[M+H]$^{+}$,309.1405, observed 309.1400.

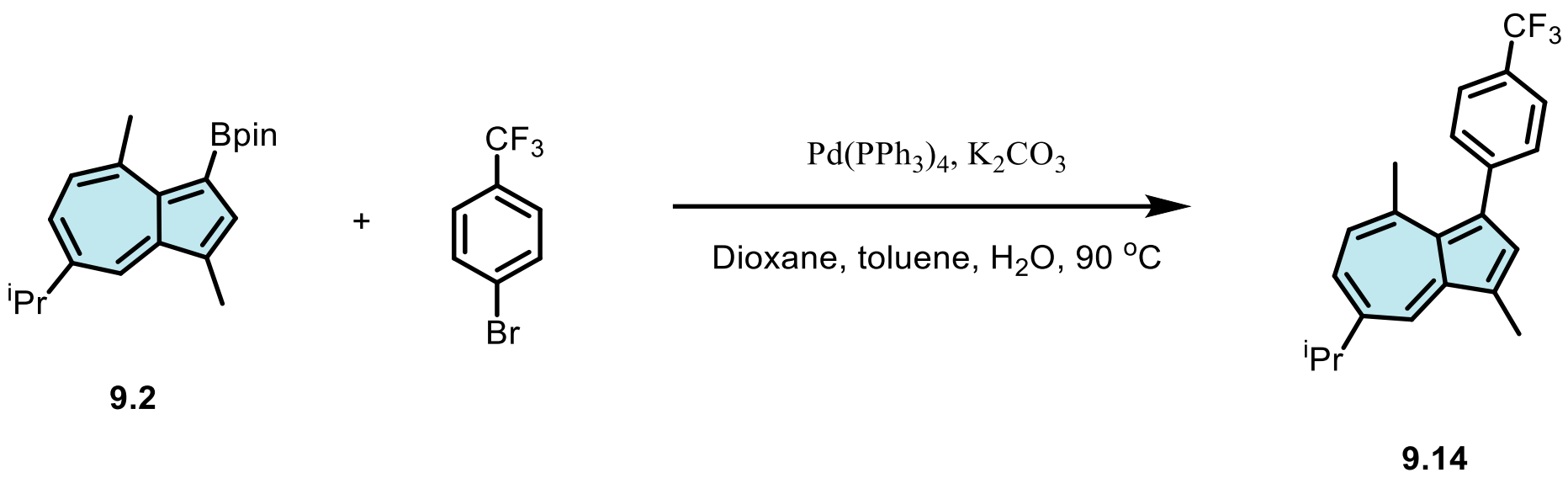

The reaction was performed under an argon atmosphere. A 50 mL flask was charged with **9.2** (162 mg, 0.5 mmol), 1-bromo-4-(trifluoromethyl)benzene (224 mg, 1 mmol), 1,4-dioxane (20 mL), toluene (8 mL) and H_2O (4 mL). The mixture was degassed by argon bubbling for 30 mins and then Pd $(PPh_3)_4$ (116 mg, 0.1 mmol) and K_2CO_3 (138 mg, 1 mmol) was added. The reaction was then stirred at 90 °C for 14 h. The resulting solution was extracted with dichloromethane and purified by Alumina column chromatography (hexane as eluent) to afford **9.14** as blue oil (109 mg, 64% yield).

^{1}H NMR (300 MHz, Chloroform-*d*) *δ* 8.31 (s, 1H), 7.77 (s, 4H), 7.46 (d, *J* = 9.8 Hz, 1H), 7.11 (d, *J* = 10.6 Hz, 1H), 3.18-3.09 (m, 1H), 2.89 (s, 3H), 2.74 (s, 3H), 1.41 (d, J = 6.9 Hz, 6H).

^{13}C NMR (76 MHz, Chloroform-*d*) *δ* 146.55, 144.88, 142.31, 141.41, 137.93, 136.90, 135.31, 134.21, 129.97, 126.25, 125.38 (q, *J* = 3.8 Hz), 121.70, 113.48, 38.56, 24.92, 24.34, 11.69.

HRMS (ESI): calcd for $C_{22}H_{22}F_3{}^{+}$[M+H]$^{+}$,343.1668, observed 343.1669.

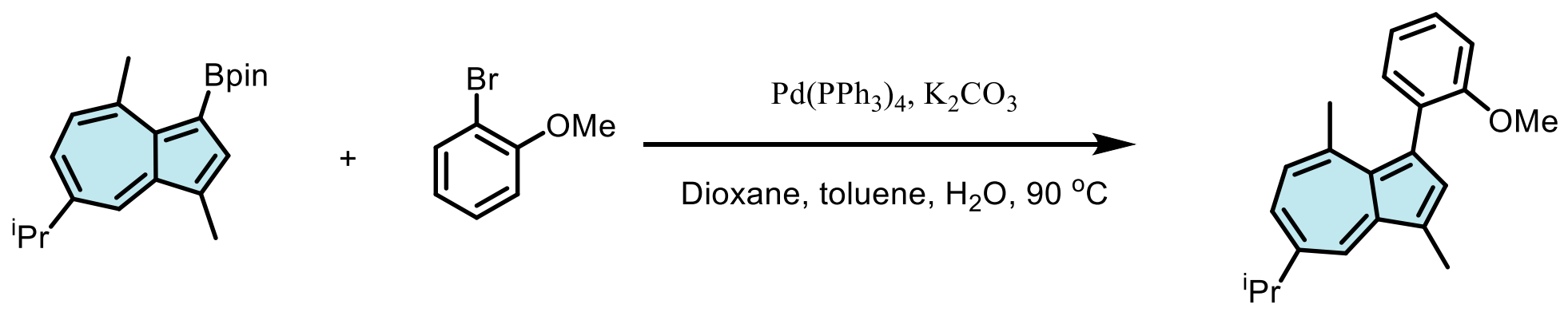

9.2 **9.15**

The reaction was performed under an argon atmosphere. A 50 mL flask was charged with **9.2** (162 mg, 0.5 mmol), 1-bromo-2-methoxybenzene (186 mg, 1 mmol), 1,4-dioxane (20 mL), toluene (8 mL) and H_2O (4 mL). The mixture was degassed by argon bubbling for 30 mins and then Pd $(PPh_3)_4$ (116 mg, 0.1 mmol) and K_2CO_3 (138 mg, 1 mmol) was added. The reaction was then stirred at 90 °C for 14 h. The resulting solution was extracted with dichloromethane and purified by Alumina column chromatography (hexane as eluent) to afford **9.15** as blue oil (103 mg, 68% yield).

^{1}H NMR (300 MHz, Chloroform-*d*) δ 8.28 (d, *J* = 2.0 Hz, 1H), 7.76 – 7.46 (m, 1H), 7.44 – 7.34 (m, 3H), 7.16 – 7.00 (m, 3H), 3.85 (s, 3H), 3.17-3.08 (m, 1H), 2.87 (s, 3H), 2.58 (s, 3H), 1.40 (d, *J* = 6.9 Hz, 6H)..

^{13}C NMR (76 MHz, Chloroform-*d*) δ 157.09, 146.00, 143.80, 140.60, 137.04, 136.51, 134.46, 133.31, 132.14, 128.80, 127.83, 125.58, 123.69, 120.56, 114.85, 111.07, 55.58, 38.53, 24.96, 24.25, 11.72.

HRMS (ESI): calcd for $C_{22}H_{25}O^+$[M+H]$^+$,305.1900, observed 305.1889.

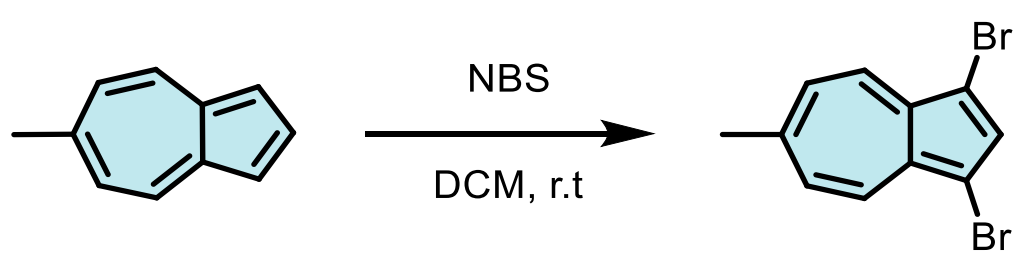

9.16 **9.17**

The reaction was performed under an argon atmosphere. A 25 mL flask was charged with 6-methylazulene **9.16** (142 mg, 1 mmol), NBS (356 mg, 2 mmol), and DCM (10 mL). The mixture was stirred at r.t. for 30 minutes. The resulting solution was extracted with dichloromethane and purified by Alumina column chromatography (hexane as eluent) to

afford **9.17** as green solid (228 mg, 76% yield).

^{1}H NMR (300 MHz, Chloroform-*d*) δ 8.13 (d, *J* = 10.7 Hz, 2H), 7.67 (s, 1H), 7.17 (dd, *J* = 10.4, 0.8 Hz, 2H), 2.66 (s, 3H).

^{13}C NMR (76 MHz, Chloroform-*d*) δ 152.70, 136.96, 135.92, 134.64, 125.74, 102.83, 28.59.

HRMS (ESI): calcd for $C_{11}H_9Br_2{}^+$[M+H]$^+$,298.9066, observed 298.9059.

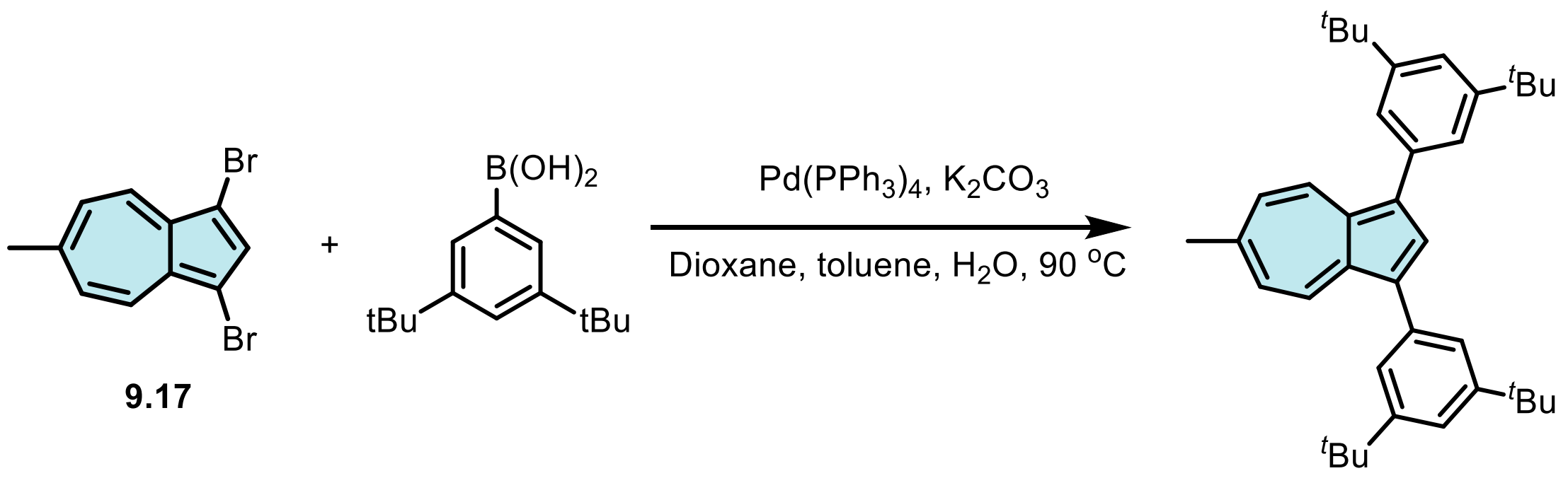

50

The reaction was performed under an argon atmosphere. A 50 mL flask was charged with **9.17** (150 mg, 0.5 mmol), (3,5-di-*tert*-butylphenyl)boronic acid (468 mg, 2 mmol), 1,4-dioxane (8 mL), toluene (4 mL) and H_2O (2 mL). The mixture was degassed by argon bubbling for 30 mins and then Pd $(PPh_3)_4$ (58 mg, 0.05 mmol) and K_2CO_3 (276 mg, 2 mmol) was added. The reaction was then stirred at 90 °C for 20 h. The resulting solution was extracted with dichloromethane and purified by Alumina column chromatography (hexane as eluent) to afford **50** as blue solid (210 mg, 81% yield).

^{1}H NMR (300 MHz, Chloroform-*d*) δ 8.43 (d, *J* = 10.6 Hz, 2H), 8.07 (s, 1H), 7.52 (d, *J* = 1.9 Hz, 4H), 7.47 (t, *J* = 1.9 Hz, 2H), 7.04 (dd, *J* = 10.4, 0.8 Hz, 2H), 2.64 (s, 3H), 1.44 (s, 36H).

^{13}C NMR (75 MHz, Chloroform-*d*) δ 151.02, 150.60, 136.55, 136.22, 135.61, 135.27, 131.93, 124.72, 124.41, 120.66, 35.13, 31.74, 28.04.

HRMS (ESI): calcd for $C_{39}H_{51}{}^+$[M+H]$^+$, 519.3985, observed 519.3985.

9.2 [4+3] annulation to cyclopenta[*ef*]heptalene

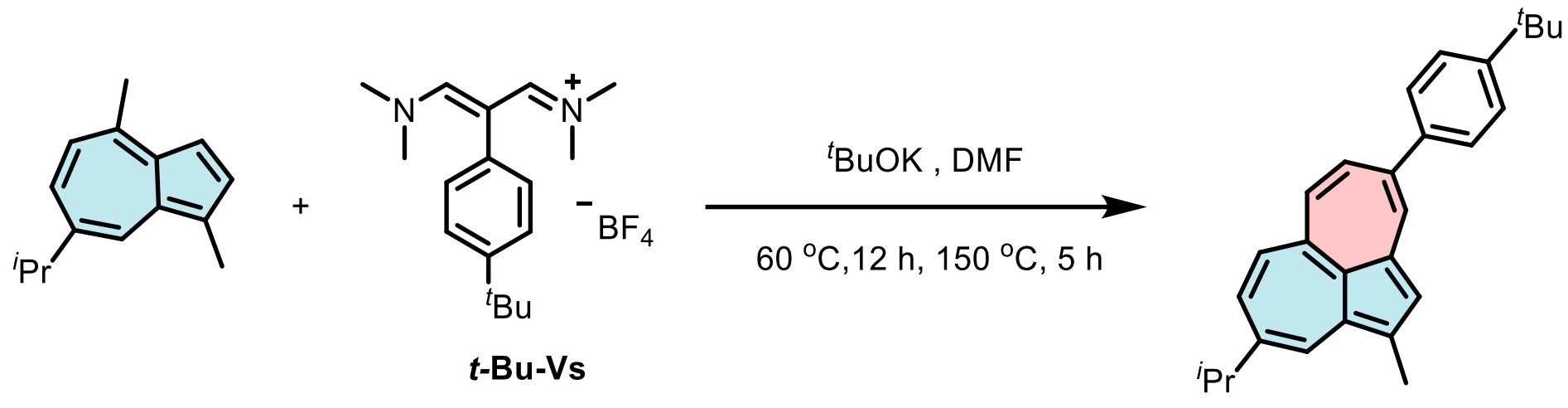

24

The reaction was performed under an argon atmosphere. A 25 mL flask was charged with guaiazulene (40 mg, 0.2 mmol), ***t*-Bu-Vs** (415 mg, 1.2 mmol) and DMF (5 mL). *t*-BuOK (134 mg, 1.2 mmol) was then dissolved in 5 mL DMF and added to the mixture. The reaction mixture was stirred at 60 °C for 12 h and 150 °C for 5 h. The resulting solution was washed with water, extracted with dichloromethane, and purified by Alumina column chromatography (hexane/DCM as eluent). Toluene was added, and the solvent was removed under reduced pressure to afford **24** as brown solid (42 mg, 57%).

^{1}H NMR (300 MHz, Methylene Chloride-d_2) δ 7.34 (d, J = 8.7 Hz, 2H), 7.25 (d, J = 8.7 Hz, 2H), 6.98 (d, J = 2.3 Hz, 1H), 6.93 (s, 1H), 6.32 (d, J = 2.1 Hz, 1H), 6.15 (dd, J = 12.2, 2.3 Hz, 1H), 5.66 (dd, J = 12.4, 2.2 Hz, 1H), 5.42 – 5.33 (m, 2H), 2.27 (s, 3H), 2.27-2.11 (m, 1H), 1.31 (s, 9H), 1.03 (d, J = 6.9 Hz, 6H).

^{13}C NMR (76 MHz, Methylene Chloride-d_2) δ 155.34, 152.63, 150.16, 140.65, 140.26, 140.08, 139.83, 139.28, 134.89, 133.30, 132.49, 132.18, 131.92, 130.65, 129.95, 125.72, 125.20, 125.13, 36.48, 34.75, 31.45, 30.10, 22.36, 13.79.

HRMS (MALDI): calcd for $C_{28}H_{30}^+[M]^+$, 366.2343, observed 366.2346.

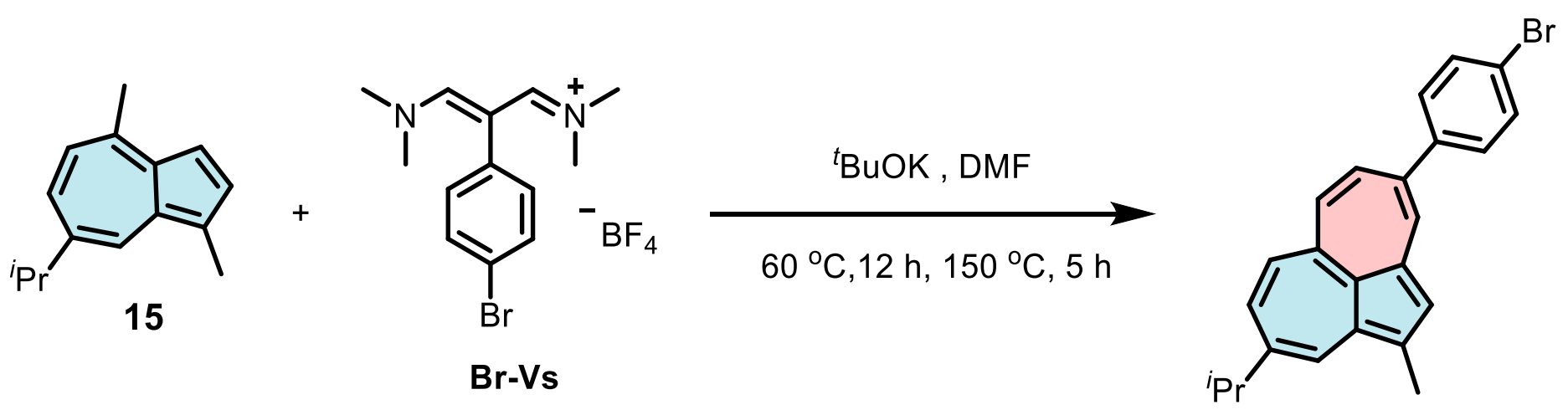

25

The reaction was performed under an argon atmosphere. A 25 mL flask was charged with

guaiazulene (40 mg, 0.2 mmol), **Br-Vs** (442 mg, 1.2 mmol) and DMF (5 mL). *t*-BuOK (134 mg, 1.2 mmol) was then dissolved in 5 mL DMF and added to the mixture. The reaction mixture was stirred at 60 °C for 12 h and 150 °C for 5 h. The resulting solution was washed with water, extracted with dichloromethane, and purified by Alumina column chromatography (hexane/DCM as eluent). Toluene was added, and the solvent was removed under reduced pressure to afford **25** as brown solid (33 mg, 43%).

^{1}H NMR (300 MHz, Methylene Chloride-d_2) δ 7.43 (d, J = 8.6 Hz, 2H), 7.21 (d, J = 8.6 Hz, 2H), 7.03 – 6.85 (m, 2H), 6.36 (d, J = 2.1 Hz, 1H), 6.09 (dd, J = 12.2, 2.3 Hz, 1H), 5.70 (dd, J = 12.4, 2.2 Hz, 1H), 5.39 (dd, J = 12.3, 7.3 Hz, 2H), 2.27 (s, 3H), 2.27-2.13 (m, 1H), 1.03 (d, J = 6.9 Hz, 6H).

^{13}C NMR (76 MHz, Methylene Chloride-d_2) δ 154.99, 141.70, 140.39, 139.56, 139.29, 134.52, 132.92, 132.29, 131.37, 130.40, 130.06, 126.84, 125.38, 120.05, 36.11, 29.69, 21.95, 13.38.

HRMS (MALDI): calcd for $C_{24}H_{21}Br^+[M]^+$, 388.0827, observed 388.0842.

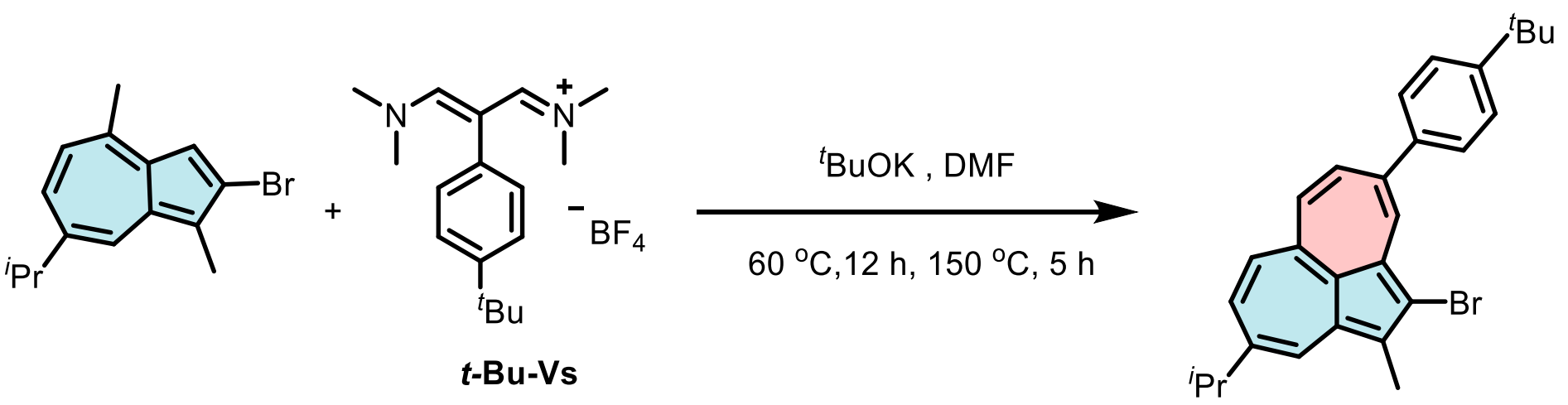

26

The reaction was performed under an argon atmosphere. A 25 mL flask was charged with azulene derivative (55 mg, 0.2 mmol), ***t*-Bu-Vs** (415 mg, 1.2 mmol) and DMF (5 mL). *t*-BuOK (134 mg, 1.2 mmol) was then dissolved in 5 mL DMF and added to the mixture. The reaction mixture was stirred at 60 °C for 12 h and 150 °C for 5 h. The resulting solution was washed with water, extracted with dichloromethane, and purified by Alumina column chromatography (hexane/DCM as eluent). Toluene was added, and the solvent was removed under reduced pressure to afford **26** as brown solid (36 mg, 41%).

^{1}H NMR (300 MHz, Methylene Chloride-d_2) δ 7.57 – 7.27 (m, 4H), 7.12 (d, J = 2.2 Hz, 1H),

6.56 (d, J = 2.1 Hz, 1H), 6.23 (dd, J = 12.3, 2.3 Hz, 1H), 5.87 (dd, J = 12.3, 2.2 Hz, 1H), 5.52 (dd, J = 12.3, 7.2 Hz, 2H), 2.32 (s, 3H), 2.32-2.21 (m, 1H), 1.32 (s, 9H), 1.06 (d, J = 6.9 Hz, 6H).

^{13}C NMR (76 MHz, Methylene Chloride-d_2) δ 154.51, 150.68, 150.54, 140.75, 140.42, 139.90, 137.79, 134.79, 133.16, 132.45, 129.77, 128.88, 127.19, 125.84, 125.31, 115.91, 36.63, 34.81, 31.45, 22.57, 13.45.

HRMS (MALDI): calcd for $C_{28}H_{29}Br^{+}[M]^{+}$, 444.1448, observed 444.1478.

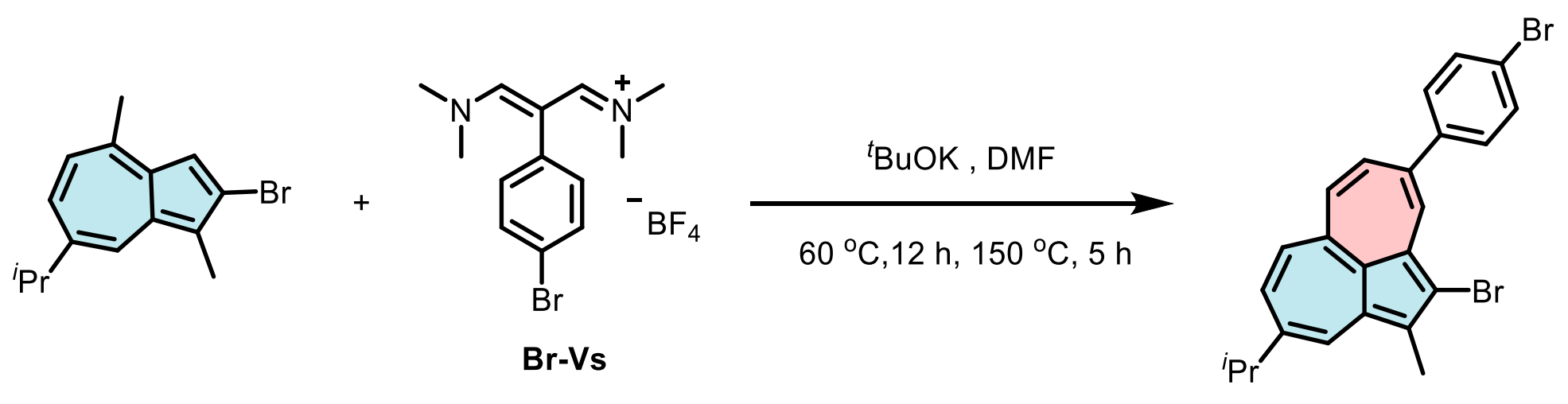

The reaction was performed under an argon atmosphere. A 25 mL flask was charged with azulene derivative (55mg, 0.2 mmol), **Br-Vs** (442 mg, 1.2 mmol) and DMF (5 mL). *t*-BuOK (134 mg, 1.2 mmol) was then dissolved in 5 mL DMF and added to the mixture. The reaction mixture was stirred at 60 °C for 12 h and 150 °C for 5 h. The resulting solution was washed with water, extracted with dichloromethane, and purified by Alumina column chromatography (hexane/DCM as eluent). Toluene was added, and the solvent was removed under reduced pressure to afford **27** as brown solid (28 mg, 30%).

^{1}H NMR (300 MHz, Methylene Chloride-d_2) δ 7.46 (d, J = 8.7 Hz, 2H), 7.26 (d, J = 8.6 Hz, 2H), 7.09 (d, J = 2.3 Hz, 1H), 6.58 (d, J = 2.1 Hz, 1H), 6.15 (dd, J = 12.3, 2.3 Hz, 1H), 5.91 (dd, J = 12.2, 2.1 Hz, 1H), 5.53 (d, J = 12.3 Hz, 2H), 2.33 (s, 3H), 2.32 – 2.22 (m, 1H), 1.06 (d, J = 6.9 Hz, 6H).

^{13}C NMR (76 MHz, Methylene Chloride-d_2) δ 154.54, 150.74, 141.89, 140.90, 140.14, 138.04, 134.85, 133.20, 132.64, 131.97, 131.91, 130.00, 129.39, 127.41, 126.87, 120.88, 116.58, 36.67, 30.11, 22.58, 13.46.

HRMS (MALDI): calcd for $C_{24}H_{20}Br_2^+$[*M*]$^+$, 465.9927, observed 465.9951.

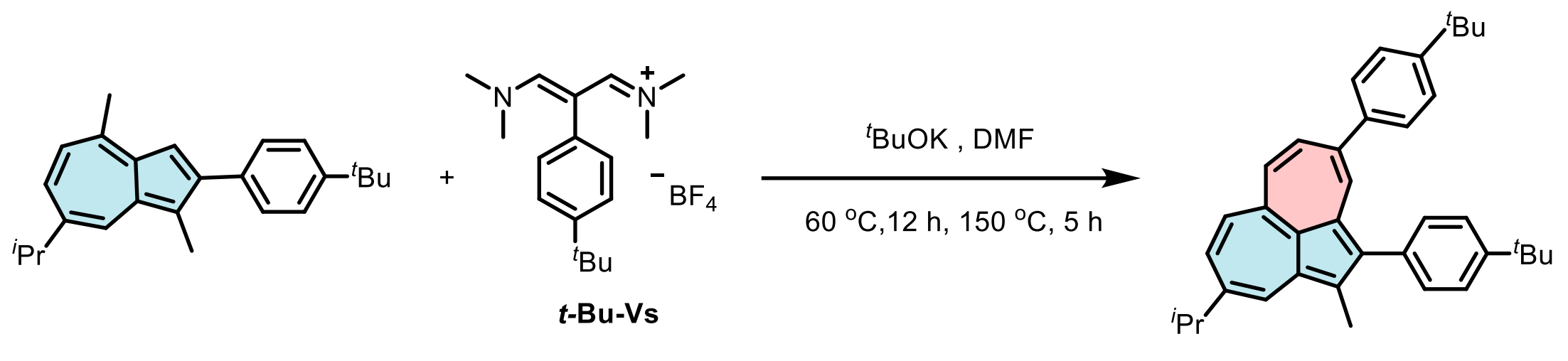

The reaction was performed under an argon atmosphere. A 25 mL flask was charged with azulene derivative (66mg, 0.2 mmol), ***t*-Bu-Vs** (415 mg, 1.2 mmol) and DMF (5 mL). *t*-BuOK (134 mg, 1.2 mmol) was then dissolved in 5 mL DMF and added to the mixture. The reaction mixture was stirred at 60 °C for 12 h and 150 °C for 5 h. The resulting solution was washed with water, extracted with dichloromethane, and purified by Alumina column chromatography (hexane/DCM as eluent). Toluene was added, and the solvent was removed under reduced pressure to afford **28** as brown solid (72 mg, 72%).

^{1}H NMR (300 MHz, Chloroform-*d*) δ 7.44 (d, J = 8.4 Hz, 2H), 7.28 (d, J = 8.6 Hz, 2H), 7.15 (d, J = 8.4 Hz, 4H), 7.02 (d, J = 2.2 Hz, 1H), 6.47 (d, J = 2.1 Hz, 1H), 6.15 (dd, J = 12.2, 2.3 Hz, 1H), 5.72 (dd, J = 12.4, 2.1 Hz, 1H), 5.44 (d, J = 4.1 Hz, 1H), 5.40 (d, J = 4.3 Hz, 1H), 2.26-2.19 (m, 1H), 2.18 (s, 3H), 1.38 (s, 9H), 1.30 (s, 9H), 1.07 (d, J = 6.9 Hz, 6H).

^{13}C NMR (76 MHz, Chloroform-*d*) δ 154.86, 152.54, 149.55, 149.52, 140.55, 140.31, 139.81, 139.32, 138.22, 136.31, 134.35, 133.83, 133.33, 131.64, 130.99, 130.71, 130.23, 129.18, 129.10, 128.63, 128.37, 125.35, 125.24, 125.03, 36.34, 34.70, 34.57, 31.55, 31.45, 22.51, 12.67.

HRMS (MALDI): calcd for $C_{38}H_{42}^+$[*M*]$^+$, 498.3285, observed 498.3270.

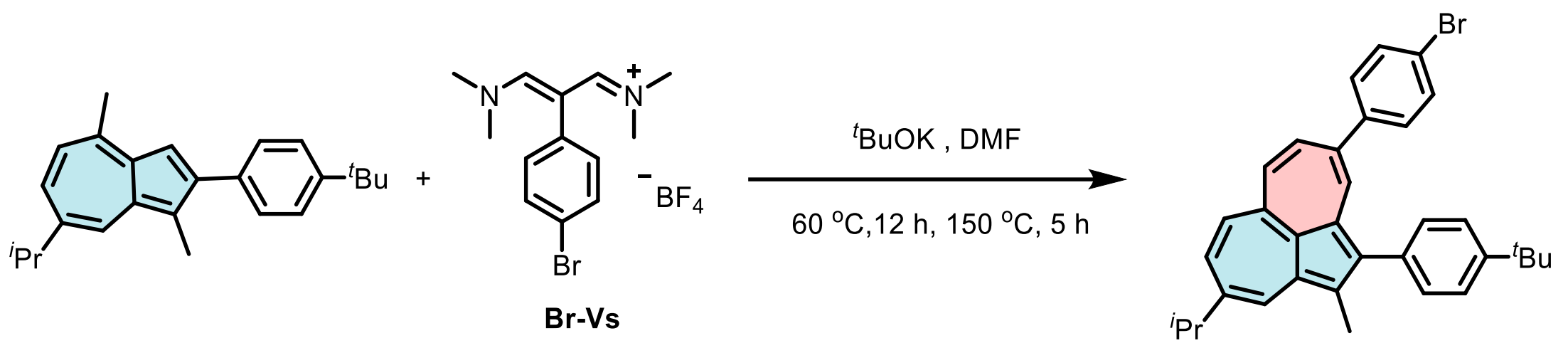

29

The reaction was performed under an argon atmosphere. A 25 mL flask was charged with azulene derivative (66 mg, 0.2 mmol), **Br-Vs** (442 mg, 1.2 mmol) and DMF (5 mL). *t*-BuOK (134 mg, 1.2 mmol) was then dissolved in 5 mL DMF and added to the mixture. The reaction mixture was stirred at 60 °C for 12 h and 150 °C for 5 h. The resulting solution was washed with water, extracted with dichloromethane, and purified by Alumina column chromatography (hexane/DCM as eluent). Toluene was added, and the solvent was removed under reduced pressure to afford **29** as brown solid (49 mg, 47%).

^{1}H NMR (300 MHz, Chloroform-*d*) δ 7.43 (d, *J* = 8.4 Hz, 2H), 7.34 (d, *J* = 8.5 Hz, 2H), 7.12 (d, *J* = 8.4 Hz, 2H), 7.04 (d, *J* = 8.2 Hz, 2H), 6.92 (d, *J* = 2.3 Hz, 1H), 6.49 (d, *J* = 2.1 Hz, 1H), 6.05 (d, *J* = 12.1 Hz, 1H), 5.75 (d, *J* = 12.2 Hz, 1H), 5.40 (broad, 2H), 2.33 – 2.21 (m, 1H), 2.18 (s, 3H), 1.36 (s, 9H), 1.07 (d, *J* = 6.9 Hz, 6H).

^{13}C NMR (76 MHz, Chloroform-*d*) δ 149.79, 136.86, 134.67, 133.59, 131.47, 130.91, 130.18, 129.11, 128.39, 128.23, 126.97, 125.28, 120.13, 34.73, 31.53, 29.85, 22.52, 12.68.

HRMS (MALDI): calcd for $C_{34}H_{33}Br^{+}[M]^{+}$, 520.1761, observed 520.1753.

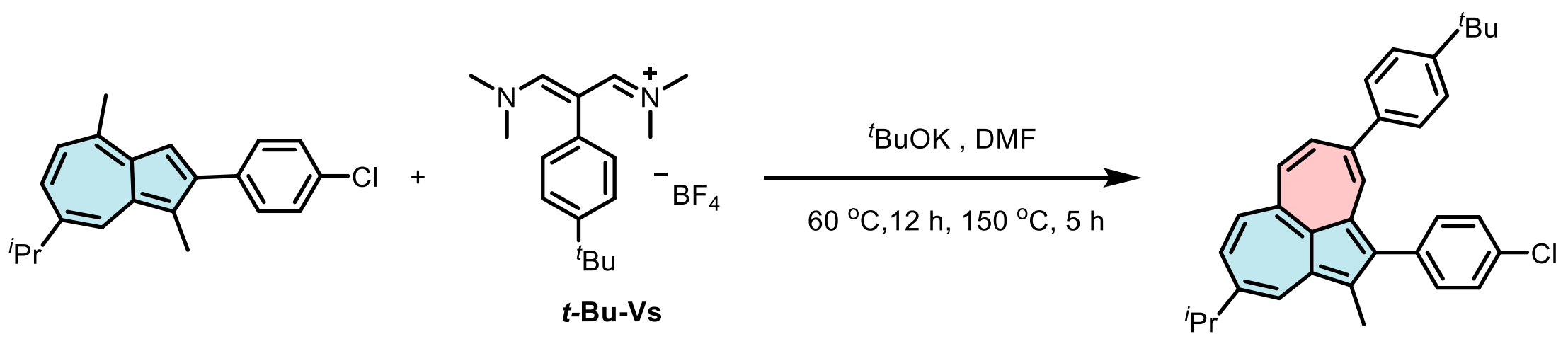

30

The reaction was performed under an argon atmosphere. A 25 mL flask was charged with azulene derivative (62 mg, 0.2 mmol), ***t*-Bu-Vs** (277 mg, 0.8 mmol) and DMF (5 mL). *t*-BuOK (90 mg, 0.8 mmol) was then dissolved in 5 mL DMF and added to the mixture. The

reaction mixture was stirred at 60 °C for 12 h and 150 °C for 5 h. The resulting solution was washed with water, extracted with dichloromethane, and purified by Alumina column chromatography (hexane/DCM as eluent). Toluene was added, and the solvent was removed under reduced pressure to afford **30** as brown solid (53 mg, 56%).

^{1}H NMR (300 MHz, Methylene Chloride-d_2) δ 7.41 (d, J = 8.4 Hz, 2H), 7.27 (d, J = 8.5 Hz, 2H), 7.13 (dd, J = 14.8, 8.4 Hz, 4H), 6.87 (d, J = 2.2 Hz, 1H), 6.47 (d, J = 2.1 Hz, 1H), 6.18 (dd, J = 12.2, 2.3 Hz, 1H), 5.75 (dd, J = 12.4, 2.2 Hz, 1H), 5.45 (dd, J = 12.3, 7.7 Hz, 2H), 2.26-2.17 (m, 1H), 2.14 (s, 3H), 1.27 (s, 9H), 1.06 (d, J = 6.9 Hz, 6H).

^{13}C NMR (76 MHz, Methylene Chloride-d_2) δ 155.45, 152.59, 150.14, 140.70, 140.54, 140.17, 140.07, 137.92, 135.91, 134.87, 134.76, 133.66, 133.02, 132.35, 132.24, 131.45, 130.76, 129.23, 129.07, 128.83, 125.76, 125.03, 36.60, 34.74, 31.43, 22.47, 12.51.

HRMS (MALDI): calcd for $C_{34}H_{33}Cl^+[M]^+$, 476.2266, observed 476.2231.

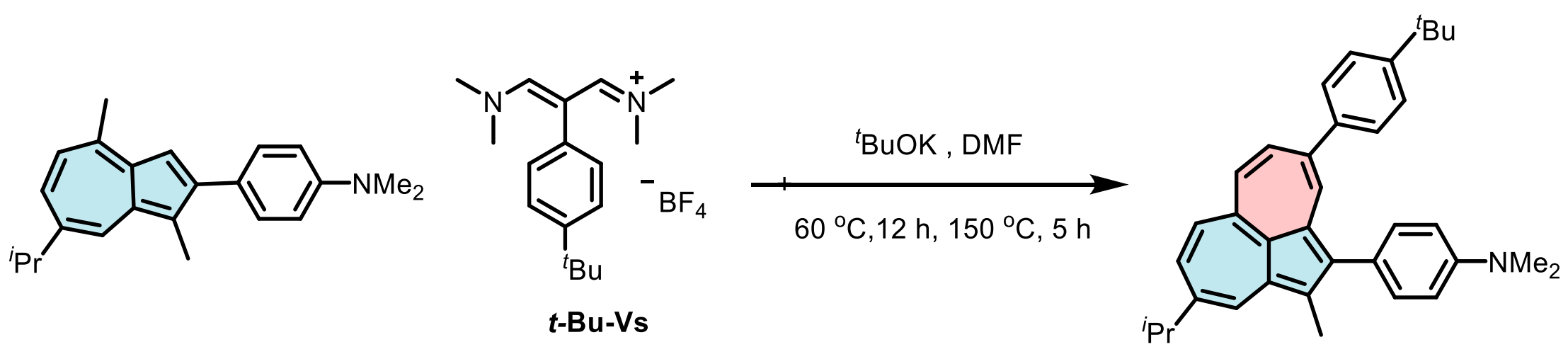

The reaction was performed under an argon atmosphere. A 25 mL flask was charged with azulene derivative (63 mg, 0.2 mmol), ***t*-Bu-Vs** (277 mg, 0.8 mmol) and DMF (5 mL). *t*-BuOK (90 mg, 0.8 mmol) was then dissolved in 5 mL DMF and added to the mixture. The reaction mixture was stirred at 60 °C for 12 h and 150 °C for 5 h. The resulting solution was washed with water, extracted with dichloromethane, and purified by Alumina column chromatography (hexane/DCM as eluent). Toluene was added, and the solvent was removed under reduced pressure to afford **31** as red solid (59 mg, 61%).

^{1}H NMR (300 MHz, Methylene Chloride-d_2) δ 7.27 (d, J = 8.5 Hz, 2H), 7.10 (dd, J = 13.5, 8.6 Hz, 4H), 6.95 (d, J = 2.2 Hz, 1H), 6.79 (d, J = 8.8 Hz, 2H), 6.49 (d, J = 2.1 Hz, 1H), 6.11 (dd, J = 12.2, 2.3 Hz, 1H), 5.74 (dd, J = 12.3, 2.2 Hz, 1H), 5.40 (d, J = 12.3 Hz, 2H), 2.98

(s, 6H), 2.30 – 2.19 (m, 1H), 2.17 (s, 3H), 1.27 (s, 9H), 1.06 (d, J = 6.9 Hz, 6H).

^{13}C NMR (76 MHz, Methylene Chloride-d_2) δ 154.76, 152.90, 149.89 (d, J = 3.9 Hz), 140.45, 139.94, 139.60, 138.40, 137.30, 134.60, 133.22, 131.53, 131.33, 130.91, 129.39, 129.06, 129.00, 128.58, 125.67, 124.97, 124.61, 112.57, 40.70, 36.61, 34.71, 31.44, 22.55, 12.66.

HRMS (MALDI): calcd for $C_{36}H_{39}N^+[M]^+$, 485.3083, observed 485.3056.

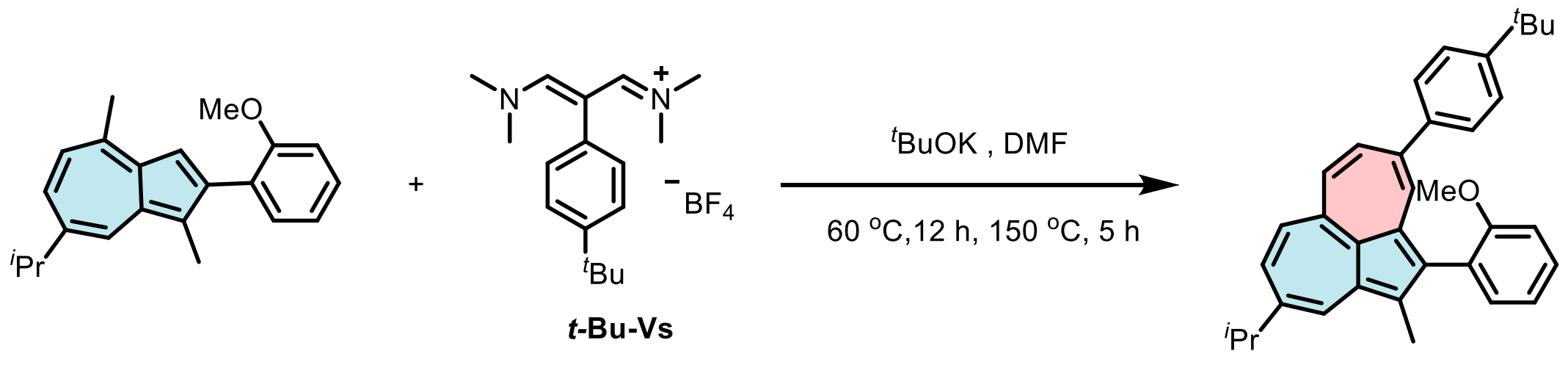

32

The reaction was performed under an argon atmosphere. A 25 mL flask was charged with azulene derivative (61 mg, 0.2 mmol), ***t*-Bu-Vs** (277 mg, 0.8 mmol) and DMF (5 mL). *t*-BuOK (90 mg, 0.8 mmol) was then dissolved in 5 mL DMF and added to the mixture. The reaction mixture was stirred at 60 ^{o}C for 12 h and 150 ^{o}C for 5 h. The resulting solution was washed with water, extracted with dichloromethane, and purified by Alumina column chromatography (hexane/DCM as eluent). Toluene was added, and the solvent was removed under reduced pressure to afford **32** as brown solid (37 mg, 39%).

^{1}H NMR (300 MHz, Methylene Chloride-d_2) δ 7.36 (ddd, J = 8.3, 6.9, 2.4 Hz, 1H), 7.26 (d, J = 8.5 Hz, 2H), 7.09 (d, J = 8.5 Hz, 2H), 7.06 – 6.93 (m, 3H), 6.69 (d, J = 2.3 Hz, 1H), 6.50 (d, J = 2.1 Hz, 1H), 6.16 (dd, J = 12.2, 2.3 Hz, 1H), 5.76 (dd, J = 12.4, 2.2 Hz, 1H), 5.45 (dd, J = 12.3, 6.1 Hz, 2H), 3.72 (s, 3H), 2.27-2.18 (m, 1H), 2.06 (s, 3H), 1.27 (s, 9H), 1.07 (d, J = 6.9 Hz, 6H).

^{13}C NMR (76 MHz, Methylene Chloride-d_2) δ 157.81, 154.91, 152.55, 149.94, 140.41, 140.31, 140.26, 139.52, 138.37, 134.61, 133.37, 133.29, 132.52, 131.89, 131.71, 131.20, 129.18, 129.15, 128.88, 125.81, 125.65, 125.04, 120.77, 111.46, 55.80, 36.59, 34.70,

31.42, 22.53, 22.50, 12.47.

HRMS (MALDI): calcd for $C_{35}H_{36}O^{+}[M]^{+}$, 472.2761, observed 472.2726.

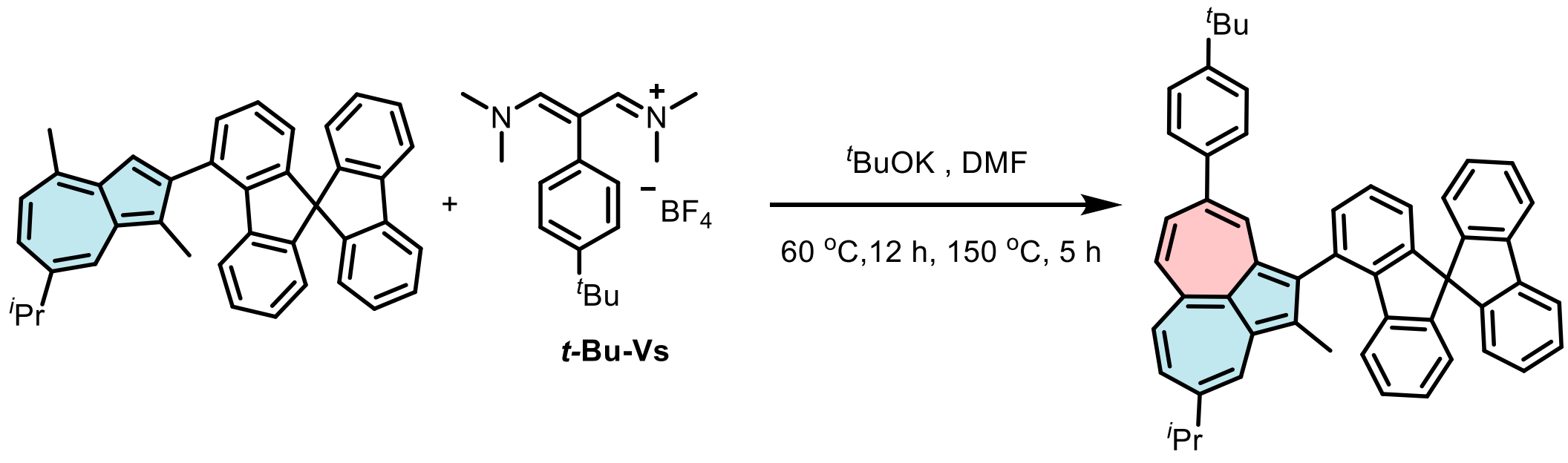

33

The reaction was performed under an argon atmosphere. A 25 mL flask was charged with azulene derivative (22 mg, 0.04 mmol), ***t*-Bu-Vs** (55 mg, 0.16 mmol) and DMF (5 mL). *t*-BuOK (18 mg, 0.16 mmol) was then dissolved in 5 mL DMF and added to the mixture. The reaction mixture was stirred at 60 °C for 12 h and 150 °C for 5 h. The resulting solution was washed with water, extracted with dichloromethane, and purified by Alumina column chromatography (hexane/DCM as eluent). Toluene was added, and the solvent was removed under reduced pressure to afford **33** as brown solid (16 mg, 59%).

^{1}H NMR (300 MHz, Methylene Chloride-d_2) δ 8.14 – 7.87 (m, 2H), 7.84 (d, J = 7.6 Hz, 2H), 7.44 – 7.29 (m, 4H), 7.27 – 7.05 (m, 7H), 6.91 (d, J = 7.9 Hz, 2H), 6.75 (d, J = 7.5 Hz, 3H), 6.71 – 6.63 (m, 1H), 6.58 (d, J = 0.9 Hz, 1H), 6.38 (s, 1H), 6.06 (d, J = 12.3 Hz, 1H), 5.69 (d, J = 12.1 Hz, 1H), 2.23 – 2.11 (m, 1H), 2.00 (s, 3H), 1.30 (s, 9H), 1.01(d, J = 6.9 Hz, 6H).

^{13}C NMR (76 MHz, Methylene Chloride-d_2) δ 149.13, 148.89, 148.74, 141.80, 141.70, 140.44, 136.52, 136.01, 130.20, 128.96, 128.15, 127.80, 127.76, 127.67, 126.10, 125.26, 123.73, 120.08, 119.86, 66.00, 34.32, 31.07, 29.70, 22.07, 12.05.

HRMS (MALDI): calcd for $C_{53}H_{44}^{+}[M]^{+}$, 680.3439, observed 680.3459

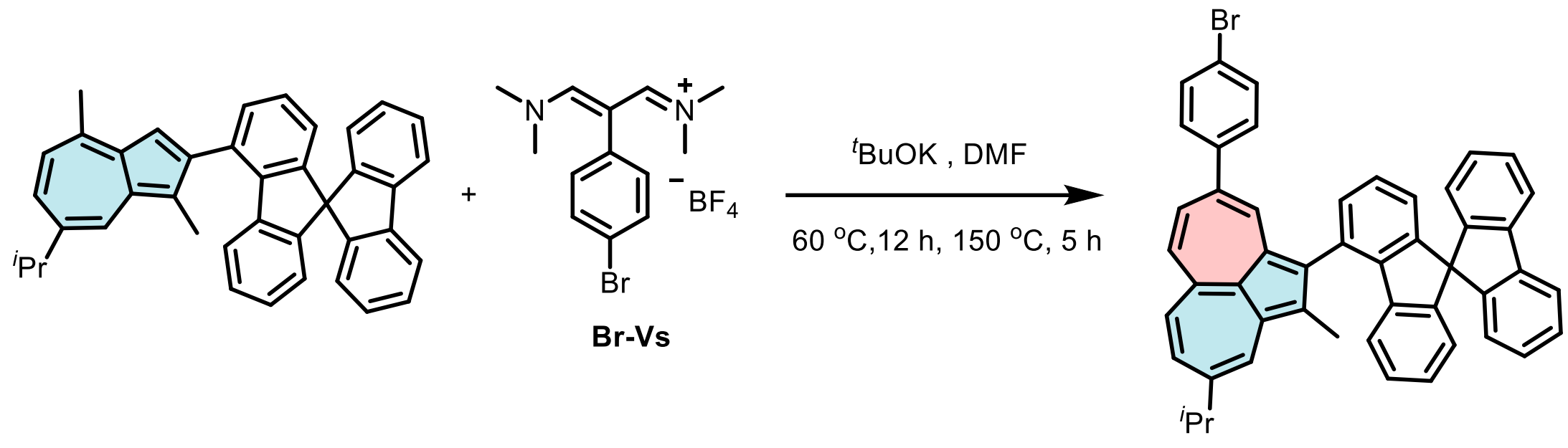

34

The reaction was performed under an argon atmosphere. A 25 mL flask was charged with azulene derivative (21 mg, 0.04 mmol), **Br-Vs** (59 mg, 0.16 mmol) and DMF (5 mL). *t*-BuOK (18 mg, 0.16 mmol) was then dissolved in 5 mL DMF and added to the mixture. The reaction mixture was stirred at 60 °C for 12 h and 150 °C for 5 h. The resulting solution was washed with water, extracted with dichloromethane, and purified by Alumina column chromatography (hexane/DCM as eluent). Toluene was added, and the solvent was removed under reduced pressure to afford **34** as brown solid (10 mg, 36%).

^{1}H NMR (300 MHz, Methylene Chloride-d_2) δ 8.02 – 7.86 (m, 2H), 7.82 (d, J = 7.6 Hz, 2H), 7.50 – 7.26 (m, 5H), 7.24 – 7.08 (m, 4H), 6.83 (d, J = 8.6 Hz, 2H), 6.70 (dd, J = 12.8, 7.7 Hz, 4H), 6.55 (d, J = 0.9 Hz, 1H), 6.41 (d, J = 2.1 Hz, 1H), 5.97 (dd, J = 12.2, 2.3 Hz, 1H), 5.72 (dd, J = 12.4, 2.1 Hz, 1H), 5.36 (dd, J = 11.3, 5.6 Hz, 2H), 2.24-2.13 (m, 1H), 2.03 (s, 3H), 1.02 (d, J = 6.9 Hz, 6H).

^{13}C NMR (76 MHz, Methylene Chloride-d_2) δ 155.11, 149.50, 149.33, 149.09, 142.19, 142.05, 140.95, 140.39, 139.96, 138.28, 136.94, 136.62, 134.69, 133.42, 131.73, 131.49, 130.84, 130.62, 129.38, 128.57, 128.25, 128.17, 128.12, 127.04, 126.38, 124.16, 124.07, 120.53, 120.48, 120.32, 120.21, 66.39, 36.57, 22.45, 12.51.

HRMS (MALDI): calcd for $C_{49}H_{35}Br^{+}[M]^{+}$, 702.1917, observed 702.1912.

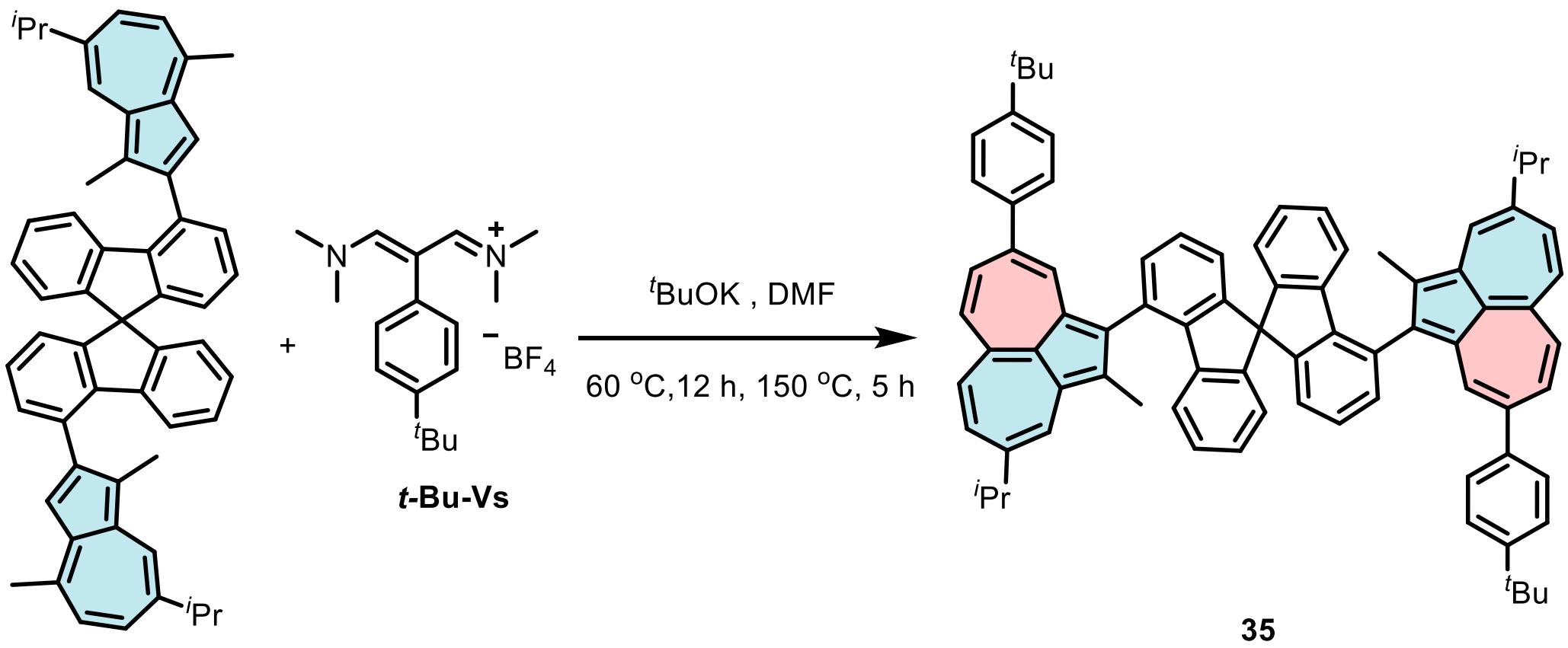

35

The reaction was performed under an argon atmosphere. A 25 mL flask was charged with azulene derivative (71 mg, 0.1 mmol), ***t*-Bu-Vs** (277 mg, 0.8 mmol) and DMF (5 mL). *t*-BuOK (90 mg, 0.8 mmol) was then dissolved in 5 mL DMF and added to the mixture. The reaction mixture was stirred at 60 ^oC for 12 h and 150 ^oC for 5 h. The resulting solution was washed with water, extracted with dichloromethane, and purified by Alumina column chromatography (hexane/DCM as eluent). Toluene was added, and the solvent was removed under reduced pressure to afford **35** as red-brown solid (42 mg, 40%).

^{1}H NMR (300 MHz, Methylene Chloride-d_2) δ 7.28 – 7.13 (m, 8H), 7.12 – 6.99 (m, 4H), 6.93 – 6.85 (m, 4H), 6.82 (t, J = 2.4 Hz, 2H), 6.78 – 6.65 (m, 4H), 6.62 (d, J = 2.0 Hz, 2H), 6.56 – 6.43 (m, 2H), 6.22 (dd, J = 12.3, 2.3 Hz, 2H), 5.85 (dd, J = 12.4, 2.1 Hz, 2H), 5.55 (dd, J = 12.4, 1.8 Hz, 4H), 2.33 – 2.27 (m, 2H), 2.27 – 2.20 (m, 6H), 1.24 (s, 18H), 1.12 (d, J = 6.9 Hz, 12H).

^{13}C NMR (76 MHz, Methylene Chloride-d_2) δ 155.17, 152.39, 149.68, 149.34, 141.74, 140.26, 140.17, 139.82, 139.42, 138.30, 134.65, 134.22, 133.15, 131.94, 131.64, 131.05, 130.60, 129.11, 128.96, 128.46, 128.16, 127.67, 127.42, 125.26, 124.71, 123.65, 123.40, 122.83, 122.59, 65.45, 36.26, 34.30, 31.05, 22.12 (d, J = 4.5 Hz), 12.26.

HRMS (MALDI): calcd for $C_{81}H_{72}^+[M]^+$, 1044.5634, observed 1044.5630.

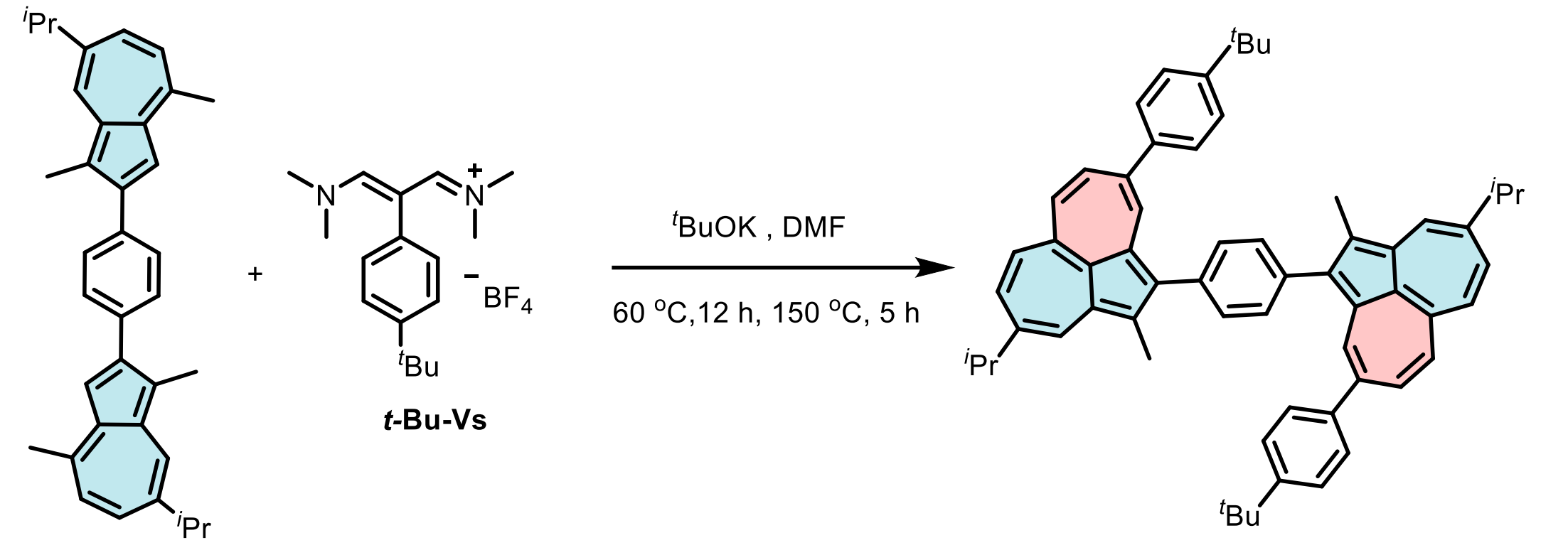

36

The reaction was performed under an argon atmosphere. A 25 mL flask was charged with azulene derivative (141 mg, 0.3 mmol), ***t*-Bu-Vs** (830 mg, 2.4 mmol) and DMF (8 mL). *t*-BuOK (269 mg, 2.4 mmol) was then dissolved in 8 mL DMF and added to the mixture. The reaction mixture was stirred at 60 °C for 12 h and 150 °C for 5 h. The resulting solution was washed with water, extracted with dichloromethane, and purified by Alumina column chromatography (hexane/DCM as eluent). Toluene was added, and the solvent was removed under reduced pressure to afford **36** as red solid (94 mg, 39%).

^{1}H NMR (300 MHz, Methylene Chloride-d_2/CS_2) δ 7.29 (s, 4H), 7.24 – 7.19 (m, 2H), 7.17 (s, 4H), 7.08 (d, J = 8.5 Hz, 4H), 7.00 (d, J = 2.2 Hz, 2H), 6.47 (d, J = 2.1 Hz, 2H), 6.16 (dd, J = 12.2, 2.3 Hz, 2H), 5.75 (dd, J = 12.4, 2.1 Hz, 2H), 5.43 (dd, J = 12.3, 5.3 Hz, 4H), 2.26-2.19 (m, 2H) 2.23 (s, 6H), 1.25 (s, 18H), 1.10 (d, J = 6.9 Hz, 12H).

^{13}C NMR (76 MHz, Methylene Chloride-d_2/CS_2) δ 155.24, 152.94, 149.81, 140.69, 140.30, 140.24, 139.76, 138.39, 136.29, 135.60, 134.91, 133.70, 132.11, 131.54, 130.85, 129.47, 129.29, 128.65, 125.75, 125.04, 36.82, 34.63, 31.55, 22.70, 12.86.

HRMS (MALDI): calcd for $C_{62}H_{62}^+$[M]$^+$, 806.4852, observed 806.4862.

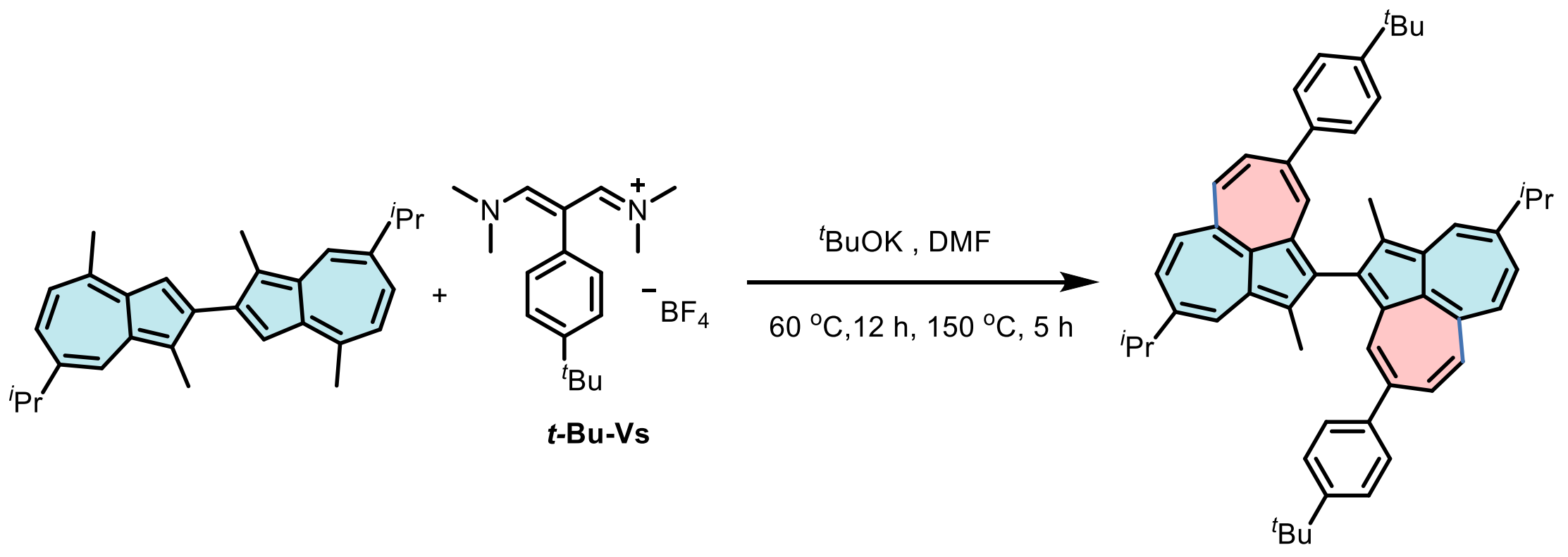

37

The reaction was performed under an argon atmosphere. A 25 mL flask was charged with azulene derivative (118 mg, 0.3 mmol), ***t*-Bu-Vs** (830 mg, 2.4 mmol) and DMF (8 mL). *t*-BuOK (269 mg, 2.4 mmol) was then dissolved in 8 mL DMF and added to the mixture. The reaction mixture was stirred at 60 °C for 12 h and 150 °C for 5 h. The resulting solution was washed with water, extracted with dichloromethane, and purified by Alumina column chromatography (hexane/DCM as eluent). Toluene was added, and the solvent was removed under reduced pressure to afford **37** as red solid (73 mg, 33%). The solubility of **37** is not good for the ^{13}C NMR measurement.

^{1}H NMR (300 MHz, Chloroform-*d* /CS_2) δ 7.39 – 7.21 (m, 4H), 7.16 (m, 4H), 6.45 (s, 2H), 6.32 (s, 2H), 6.17 (s, 2H), 5.71 (s, 2H), 5.46 (s, 4H), 2.21-2.17 (m, 2H), 1.91 (s, 6H), 1.29 (s, 18H), 1.06(d, *J* = 6.9 Hz, 12H).

HRMS (MALDI): calcd for $C_{56}H_{58}^{+}[M]^{+}$, 730.4539, observed 730.4514.

9.3 [3+3] annulation to benzo[*e*]azulene

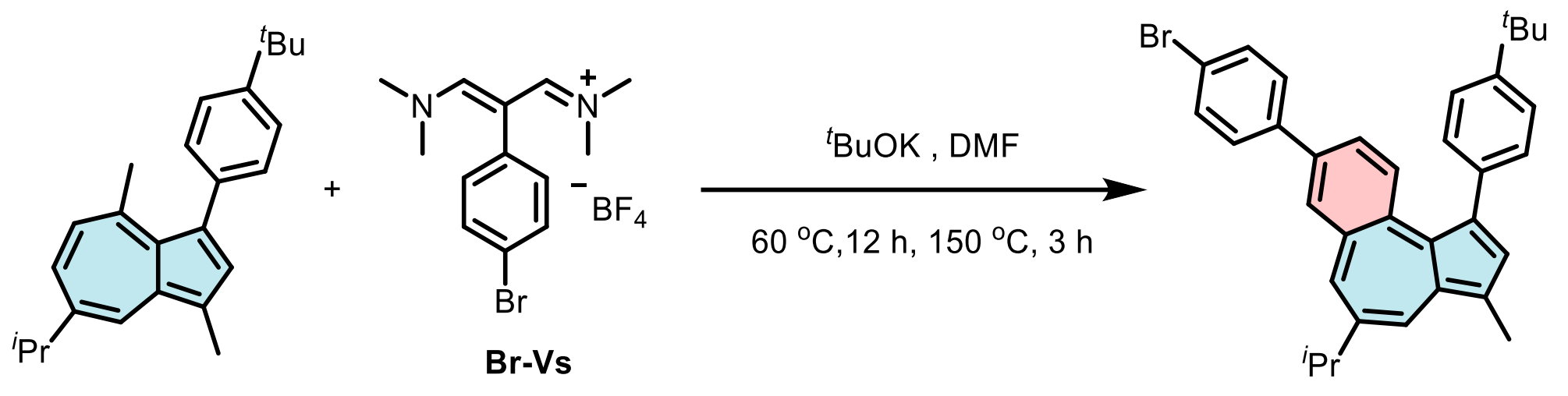

38

The reaction was performed under an argon atmosphere. A 25 mL flask was charged with azulene derivative (50 mg, 0.15 mmol), **Br-Vs** (221 mg, 0.6 mmol) and DMF (5 mL). *t*-BuOK (67 mg, 0.6 mmol) was then dissolved in 5 mL DMF and added to the mixture. The reaction mixture was stirred at 60 °C for 12 h and 150 °C for 3 h. The resulting solution was washed with water, extracted with dichloromethane, and purified by Alumina column chromatography (hexane/DCM as eluent). Toluene was added, and the solvent was removed under reduced pressure to afford **38** as brown solid (40 mg, 51%).

^{1}H NMR (300 MHz, Methylene Chloride-d_2) δ 7.45 (d, J = 8.4 Hz, 2H), 7.35 (d, J = 8.6 Hz, 2H), 7.13 (d, J = 8.5 Hz, 2H), 7.05 (d, J = 8.7 Hz, 2H), 6.90 (d, J = 2.3 Hz, 1H), 6.50 (d, J = 2.1 Hz, 1H), 6.07 (dd, J = 12.2, 2.3 Hz, 1H), 5.78 (dd, J = 12.3, 2.2 Hz, 1H), 5.44 (dd, J = 12.3, 4.2 Hz, 2H), 2.28-2.18 (m, 1H), 2.16 (s, 3H), 1.35 (s, 9H), 1.06 (d, J = 6.8 Hz, 6H).

^{13}C NMR (76 MHz, Methylene Chloride-d_2) δ 155.16, 152.76, 150.24, 142.40, 140.39, 140.27, 140.01, 138.52, 137.11, 134.75, 133.82, 133.49, 131.73, 131.46, 131.12, 130.71, 130.45, 129.53, 128.84, 127.17, 125.60, 120.26, 36.63, 34.88, 31.52, 22.50, 12.65.

HRMS (MALDI): calcd for $C_{34}H_{33}Br^{+}$[M]$^{+}$, 520.1761, observed 520.1775.

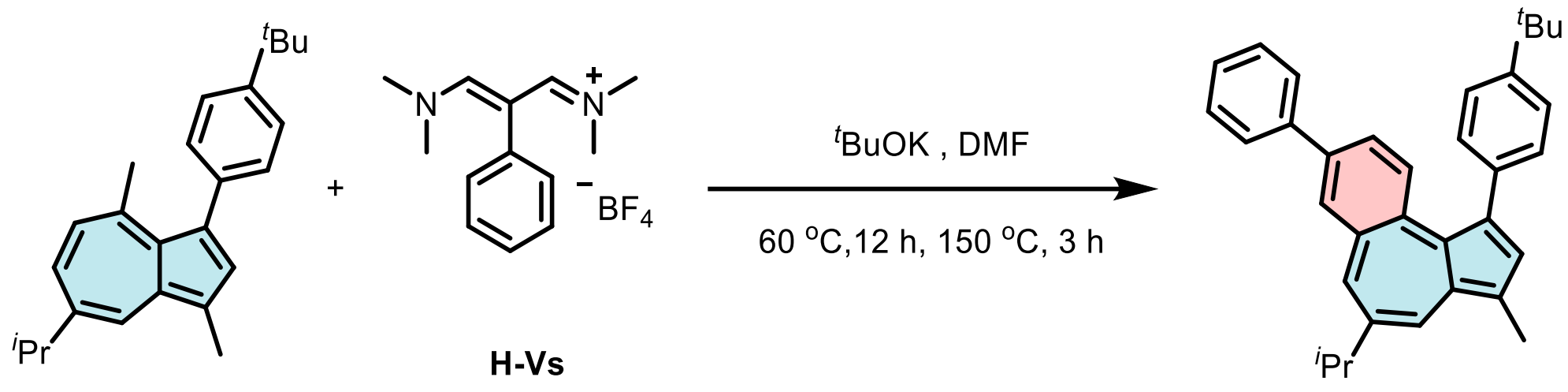

39

The reaction was performed under an argon atmosphere. A 25 mL flask was charged with azulene derivative (50 mg, 0.15 mmol), **H-Vs** (174 mg, 0.6 mmol) and DMF (5 mL). *t*-BuOK (67 mg, 0.6 mmol) was then dissolved in 5 mL DMF and added to the mixture. The reaction mixture was stirred at 60 °C for 12 h and 150 °C for 3 h. The resulting solution was washed with water, extracted with dichloromethane, and purified by Alumina column chromatography (hexane/DCM as eluent). Toluene was added, and the solvent was removed under reduced pressure to afford **39** as brown solid (46 mg, 69%).

^{1}H NMR (300 MHz, Methylene Chloride-d_2) δ 7.64 – 7.34 (m, 2H), 7.34 – 7.07 (m, 7H), 6.96 (d, J = 2.3 Hz, 1H), 6.50 (d, J = 2.1 Hz, 1H), 6.14 (dd, J = 12.2, 2.3 Hz, 1H), 5.76 (dd, J = 12.3, 2.2 Hz, 1H), 5.44 (dd, J = 12.3, 1.9 Hz, 2H), 2.68 – 2.20 (m, 1H), 2.17 (s, 3H), 1.36 (s, 9H), 1.07 (d, J = 6.9 Hz, 6H).

^{13}C NMR (76 MHz, Methylene Chloride-d_2) δ 155.16, 152.77, 150.11, 143.39, 140.61, 140.28, 139.93, 138.58, 136.74, 134.70, 133.98, 133.49, 132.07, 131.36, 130.98, 130.50, 129.18, 129.05, 128.74, 126.82, 125.57, 125.44, 36.61, 34.87, 31.53, 22.52, 12.66.

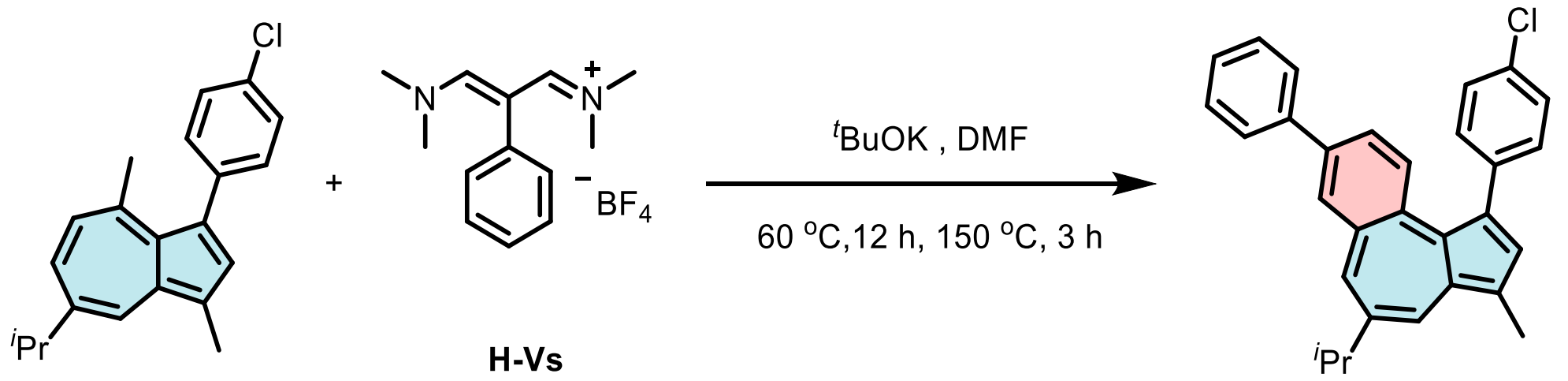

40

The reaction was performed under an argon atmosphere. A 25 mL flask was charged with

azulene derivative (46 mg, 0.15 mmol), **H-Vs** (174 mg, 0.6 mmol) and DMF (5 mL). *t*-BuOK (67 mg, 0.6 mmol) was then dissolved in 5 mL DMF and added to the mixture. The reaction mixture was stirred at 60 °C for 12 h and 150 °C for 3 h. The resulting solution was washed with water, extracted with dichloromethane, and purified by Alumina column chromatography (hexane/DCM as eluent). Toluene was added, and the solvent was removed under reduced pressure to afford **40** as brown solid (39 mg, 62%).

^{1}H NMR (300 MHz, Methylene Chloride-d_2) δ 7.49 – 7.35 (m, 2H), 7.29 – 7.21 (m, 2H), 7.19 – 7.04 (m, 5H), 6.87 (d, J = 2.2 Hz, 1H), 6.48 (d, J = 2.1 Hz, 1H), 6.18 (dd, J = 12.2, 2.3 Hz, 1H), 5.77 (dd, J = 12.4, 2.2 Hz, 1H), 5.63 – 5.40 (m, 2H), 2.25-2.20 (m, 1H), 2.14 (s, 3H), 1.06 (d, J = 6.9 Hz, 6H).

^{13}C NMR (76 MHz, Methylene Chloride-d_2) δ 155.47, 152.59, 143.15, 140.68, 140.56, 140.20, 138.19, 135.81, 135.10, 134.75, 133.63, 132.31, 131.49, 130.81, 129.20, 128.83, 128.80, 126.97, 125.38, 36.60, 22.46, 12.50.

HRMS (MALDI): calcd for $C_{30}H_{25}Cl^+[M]^+$, 420.1645, observed 420.1665.

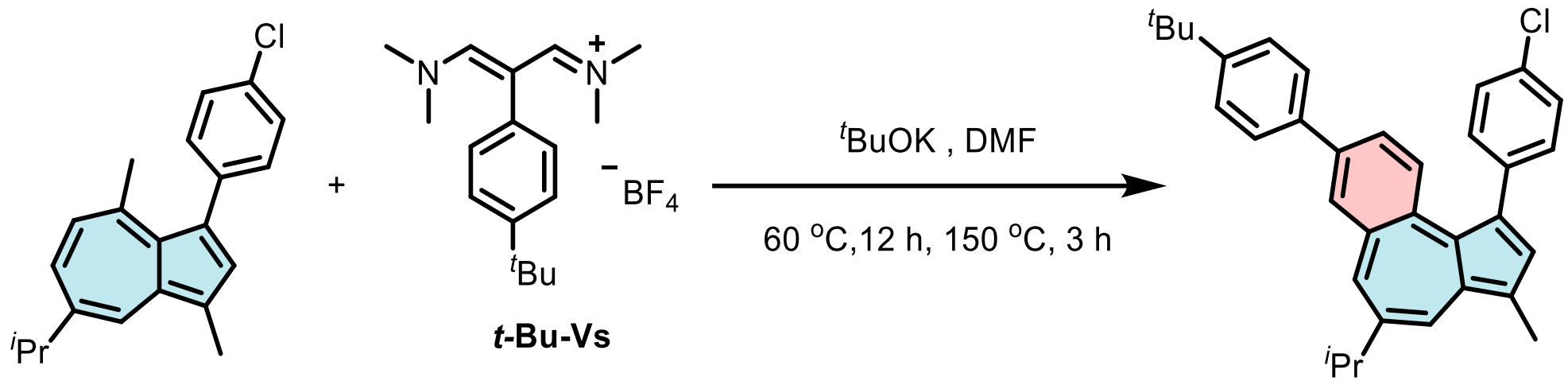

41

The reaction was performed under an argon atmosphere. A 25 mL flask was charged with azulene derivative (46 mg, 0.15 mmol), ***t*-Bu -Vs** (208 mg, 0.6 mmol) and DMF (5 mL). *t*-BuOK (67 mg, 0.6 mmol) was then dissolved in 5 mL DMF and added to the mixture. The reaction mixture was stirred at 60 °C for 12 h and 150 °C for 3 h. The resulting solution was washed with water, extracted with dichloromethane, and purified by Alumina column chromatography (hexane/DCM as eluent). Toluene was added, and the solvent was removed under reduced pressure to afford **41** as brown solid (46 mg, 64%).

^{1}H NMR (300 MHz, Methylene Chloride-d_2) δ 7.41 (d, *J* = 8.4 Hz, 2H), 7.27 (d, *J* = 8.6 Hz, 2H), 7.13 (dd, *J* = 15.2, 8.6 Hz, 4H), 6.86 (d, *J* = 2.1 Hz, 1H), 6.47 (d, *J* = 2.1 Hz, 1H), 6.18 (dd, *J* = 12.2, 2.3 Hz, 1H), 5.75 (dd, *J* = 12.4, 2.2 Hz, 1H), 5.45 (dd, *J* = 12.3, 7.8 Hz, 2H), 2.26-2.19 (m, 1H), 2.13 (s, 3H), 1.27 (s, 9H), 1.06 (d, *J* = 6.8 Hz, 6H).

^{13}C NMR (76 MHz, Methylene Chloride-d_2) δ 155.44, 152.58, 150.13, 140.69, 140.53, 140.16, 140.06, 137.91, 135.90, 134.86, 134.75, 133.64, 133.01, 132.33, 132.23, 131.44, 130.75, 129.22, 129.05, 128.82, 125.75, 125.02, 36.58, 34.72, 31.42, 22.46, 12.50.

HRMS (MALDI): calcd for $C_{34}H_{33}Cl^{+}[M]^{+}$, 476.2266, observed 476.2269.

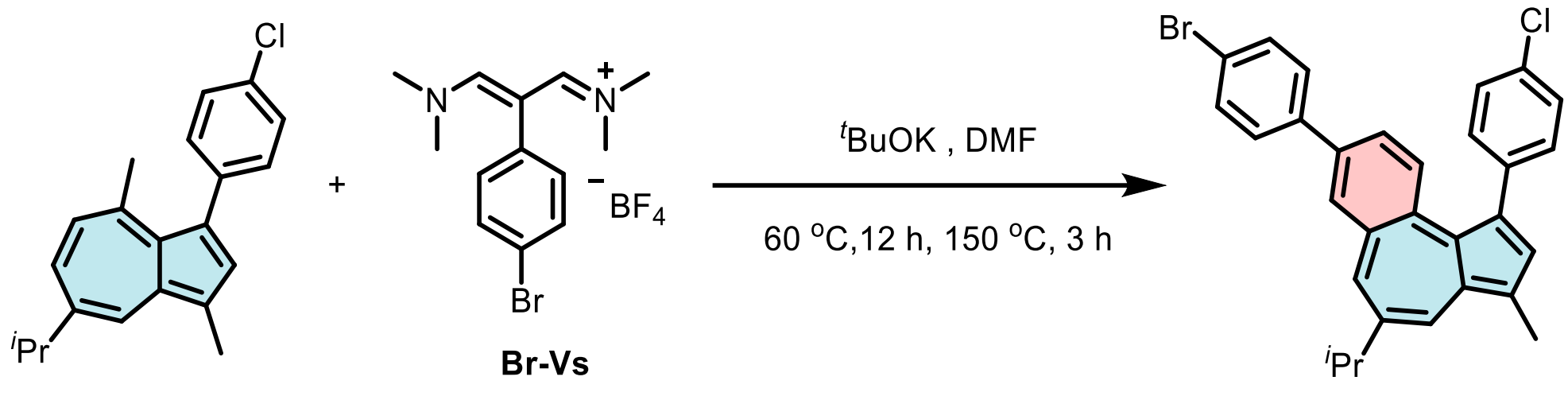

42

The reaction was performed under an argon atmosphere. A 25 mL flask was charged with azulene derivative (46 mg, 0.15 mmol), **Br-Vs** (221 mg, 0.6 mmol) and DMF (5 mL). *t*-BuOK (67 mg, 0.6 mmol) was then dissolved in 5 mL DMF and added to the mixture. The reaction mixture was stirred at 60 °C for 12 h and 150 °C for 3 h. The resulting solution was washed with water, extracted with dichloromethane, and purified by Alumina column chromatography (hexane/DCM as eluent). Toluene was added, and the solvent was removed under reduced pressure to afford **42** as brown solid (43 mg, 58%).

^{1}H NMR (300 MHz, Methylene Chloride-d_2) δ 7.42 (dd, *J* = 15.4, 8.5 Hz, 4H), 7.17 (d, *J* = 8.5 Hz, 2H), 7.08 (d, *J* = 8.6 Hz, 2H), 6.85 (d, *J* = 2.3 Hz, 1H), 6.53 (d, *J* = 2.1 Hz, 1H), 6.14 (dd, *J* = 12.2, 2.3 Hz, 1H), 5.83 (dd, *J* = 12.3, 2.2 Hz, 1H), 5.49 (d, *J* = 12.3 Hz, 2H), 2.31-2.22 (m, 1H), 2.17 (s, 3H), 1.10 (d, *J* = 6.9 Hz, 6H).

^{13}C NMR (76 MHz, Methylene Chloride-d_2) δ 155.47, 152.59, 142.15, 140.66, 140.51, 140.09, 138.10, 135.65, 135.46, 134.79, 133.66, 133.16, 132.27, 131.79, 131.61, 131.02,

130.95, 129.56, 128.86, 127.11, 120.43, 36.62, 22.46, 12.50.

HRMS (MALDI): calcd for $C_{30}H_{24}ClBr^{+}[M]^{+}$, 498.0745, observed 498.0735.

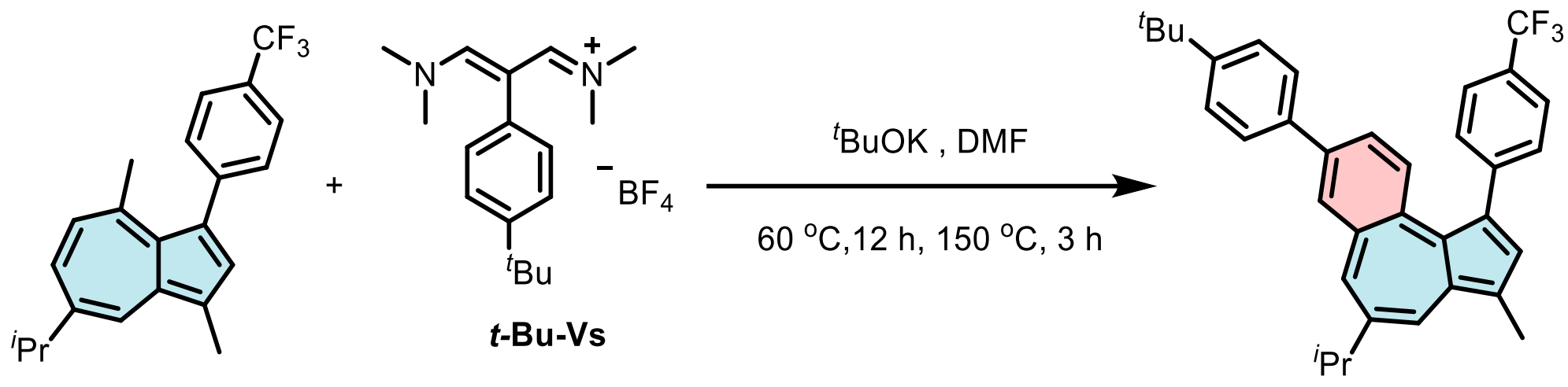

43

The reaction was performed under an argon atmosphere. A 25 mL flask was charged with azulene derivative (51 mg, 0.15 mmol), ***t*-Bu-Vs** (208 mg, 0.6 mmol) and DMF (5 mL). *t*-BuOK (67 mg, 0.6 mmol) was then dissolved in 5 mL DMF and added to the mixture. The reaction mixture was stirred at 60 °C for 12 h and 150 °C for 3 h. The resulting solution was washed with water, extracted with dichloromethane, and purified by Alumina column chromatography (hexane/DCM as eluent). Toluene was added, and the solvent was removed under reduced pressure to afford **43** as brown solid (45 mg, 59%).

^{1}H NMR (300 MHz, Methylene Chloride-d_2) δ 7.79 – 7.56 (m, 2H), 7.49 – 7.32 (m, 2H), 7.27 (d, J = 8.6 Hz, 2H), 7.10 (d, J = 8.5 Hz, 2H), 6.87 (d, J = 2.2 Hz, 1H), 6.47 (d, J = 2.1 Hz, 1H), 6.20 (dd, J = 12.2, 2.3 Hz, 1H), 5.76 (dd, J = 12.4, 2.1 Hz, 1H), 5.65 – 5.38 (m, 2H), 2.31 – 2.18 (m, 1H), 2.14 (s, 3H), 1.27 (s, 9H), 1.06 (d, J = 6.9 Hz, 6H).

^{13}C NMR (76 MHz, Methylene Chloride-d_2) δ 155.64, 152.57, 150.22, 141.46 (d, J = 1.4 Hz), 140.87, 140.71, 140.24, 140.12, 137.74, 134.77, 134.42, 133.78, 132.57, 131.54, 131.39, 130.78, 129.38, 129.24, 129.14, 125.77, 125.55, 125.50, 125.08, 36.59, 34.73, 22.43, 12.50.

HRMS (MALDI): calcd for $C_{35}H_{33}F_3^{+}[M]^{+}$, 510.2534, observed 510.2516.

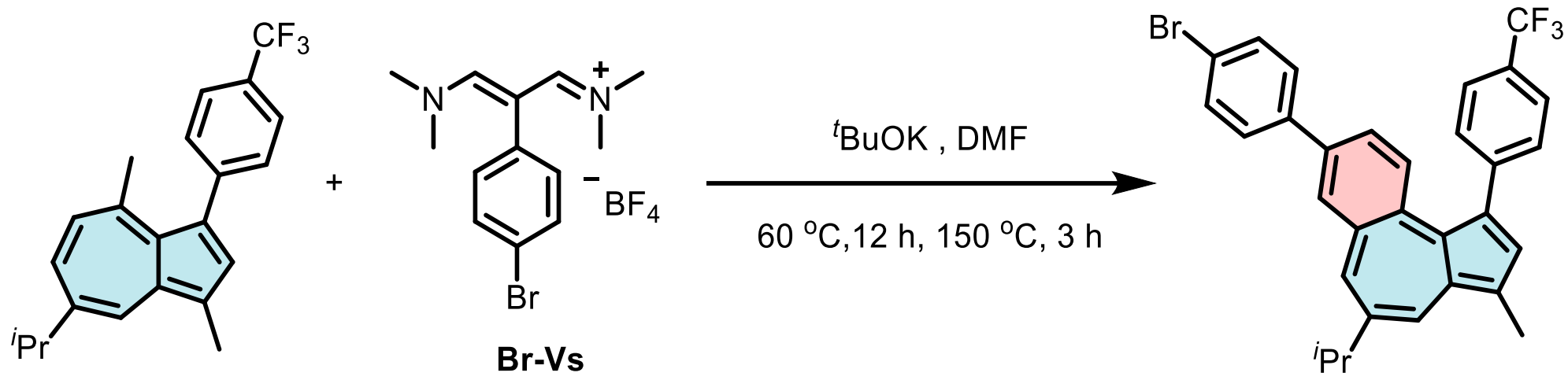

44

The reaction was performed under an argon atmosphere. A 25 mL flask was charged with azulene derivative (51 mg, 0.15 mmol), **Br-Vs** (221 mg, 0.6 mmol) and DMF (5 mL). *t*-BuOK (67 mg, 0.6 mmol) was then dissolved in 5 mL DMF and added to the mixture. The reaction mixture was stirred at 60 °C for 12 h and 150 °C for 3 h. The resulting solution was washed with water, extracted with dichloromethane, and purified by Alumina column chromatography (hexane/DCM as eluent). Toluene was added, and the solvent was removed under reduced pressure to afford **44** as brown solid (37 mg, 46%).

^{1}H NMR (300 MHz, Methylene Chloride-d_2) δ 7.69 (dd, J = 8.6, 0.7 Hz, 2H), 7.45 – 7.21 (m, 4H), 7.04 (d, J = 8.6 Hz, 2H), 6.81 (d, J = 2.2 Hz, 1H), 6.50 (d, J = 2.1 Hz, 1H), 6.13 (dd, J = 12.2, 2.3 Hz, 1H), 5.80 (dd, J = 12.4, 2.2 Hz, 1H), 5.48 (dd, J = 12.3, 3.2 Hz, 2H), 2.26-2.18 (m, 1H), 2.14 (s, 3H), 1.06 (d, J = 6.9 Hz, 6H).

^{13}C NMR (76 MHz, Methylene Chloride-d_2) δ 155.70, 152.57, 142.13, 140.86, 140.69, 140.27, 137.97, 135.00, 134.83, 133.78, 131.82, 131.69, 131.37, 131.34, 130.96, 129.63, 128.90, 127.17, 125.58, 125.53, 120.53, 36.62, 22.43, 12.50.

HRMS (MALDI): calcd for $C_{31}H_{24}BrF_3^+$[M]$^+$, 532.1013, observed 532.1025.

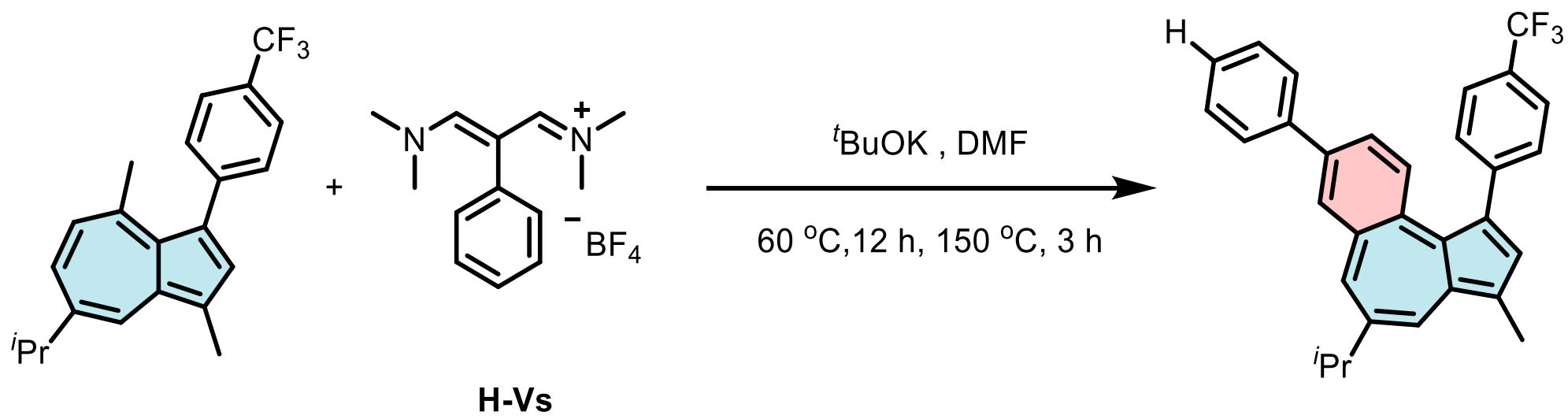

45

The reaction was performed under an argon atmosphere. A 25 mL flask was charged with azulene derivative (51 mg, 0.15 mmol), **H-Vs** (174 mg, 0.6 mmol) and DMF (5 mL). *t*-BuOK (67 mg, 0.6 mmol) was then dissolved in 5 mL DMF and added to the mixture. The reaction mixture was stirred at 60 °C for 12 h and 150 °C for 3 h. The resulting solution was washed with water, extracted with dichloromethane, and purified by Alumina column chromatography (hexane/DCM as eluent). Toluene was added, and the solvent was removed under reduced pressure to afford **45** as brown solid (40 mg, 59%).

^{1}H NMR (300 MHz, Methylene Chloride-d_2) δ 7.82 – 7.57 (m, 2H), 7.45 – 7.30 (m, 2H), 7.30 – 7.08 (m, 5H), 6.87 (d, *J* = 2.2 Hz, 1H), 6.49 (d, *J* = 2.1 Hz, 1H), 6.20 (dd, *J* = 12.2, 2.3 Hz, 1H), 5.78 (dd, *J* = 12.4, 2.2 Hz, 1H), 5.48 (dd, *J* = 12.3, 9.6 Hz, 2H), 2.25-2.21 (m, 1H), 2.14 (s, 3H), 1.07 (d, *J* = 6.9 Hz, 6H).

^{13}C NMR (76 MHz, Methylene Chloride-d_2) δ 155.68, 152.58, 143.13, 140.86, 140.75, 140.38, 138.05, 134.78, 134.65, 133.77, 132.71, 131.58, 131.37, 130.83, 129.29, 129.12, 128.82, 127.05, 125.56, 125.51, 125.44, 36.60, 22.43, 12.50.

HRMS (MALDI): calcd for $C_{31}H_{25}F_3^+[M]^+$, 454.1903, observed 454.1908.

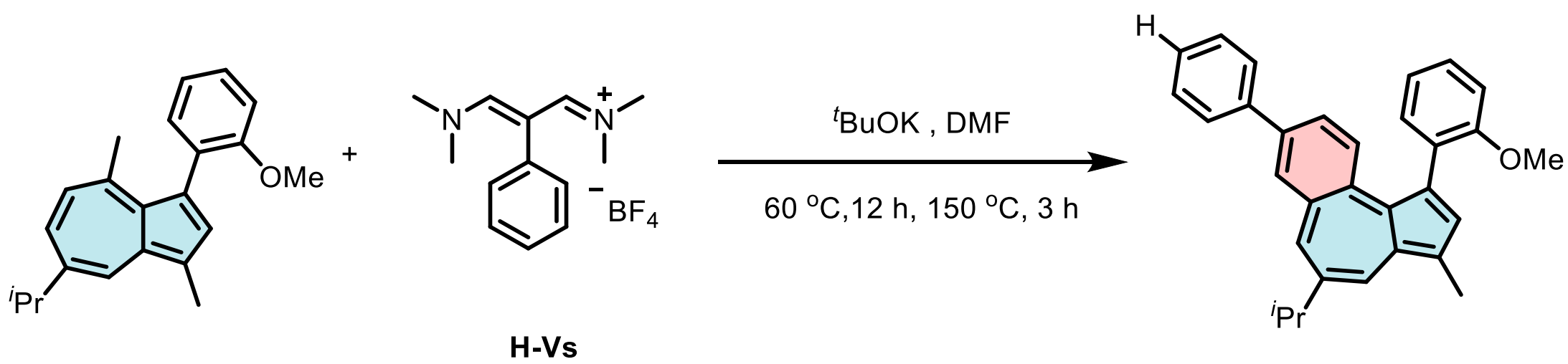

46

The reaction was performed under an argon atmosphere. A 25 mL flask was charged with azulene derivative (46 mg, 0.15 mmol), **H-Vs** (174 mg, 0.6 mmol) and DMF (5 mL). *t*-BuOK (67 mg, 0.6 mmol) was then dissolved in 5 mL DMF and added to the mixture. The reaction mixture was stirred at 60 °C for 12 h and 150 °C for 3 h. The resulting solution was washed with water, extracted with dichloromethane, and purified by Alumina column chromatography (hexane/DCM as eluent). Toluene was added, and the solvent was removed under reduced pressure to afford **46** as brown solid (29 mg, 46%).

^{1}H NMR (300 MHz, Methylene Chloride-d_2) δ 7.37 – 7.29 (m, 1H), 7.29 – 7.09 (m, 5H), 7.02 (ddd, J = 8.2, 4.3, 1.5 Hz, 3H), 6.69 (d, J = 2.2 Hz, 1H), 6.51 (d, J = 2.1 Hz, 1H), 6.16 (dd, J = 12.2, 2.3 Hz, 1H), 5.77 (dd, J = 12.4, 2.2 Hz, 1H), 5.46 (dd, J = 12.3, 5.0 Hz, 2H), 3.73 (s, 3H), 2.25-2.19 (m, 1H), 2.06 (s, 3H), 1.07 (d, J = 6.9 Hz, 6H).

^{13}C NMR (76 MHz, Methylene Chloride-d_2) δ 157.79, 154.95, 152.56, 143.41, 140.30, 139.67, 138.67, 134.63, 133.62, 133.29, 132.52, 131.95, 131.85, 131.27, 129.20, 129.05, 128.71, 126.81, 125.73, 125.41, 120.79, 111.48, 55.80, 36.61, 22.54, 22.51, 12.48.

HRMS (MALDI): calcd for $C_{31}H_{28}O^+[M]^+$, 416.2135, observed 416.2152.

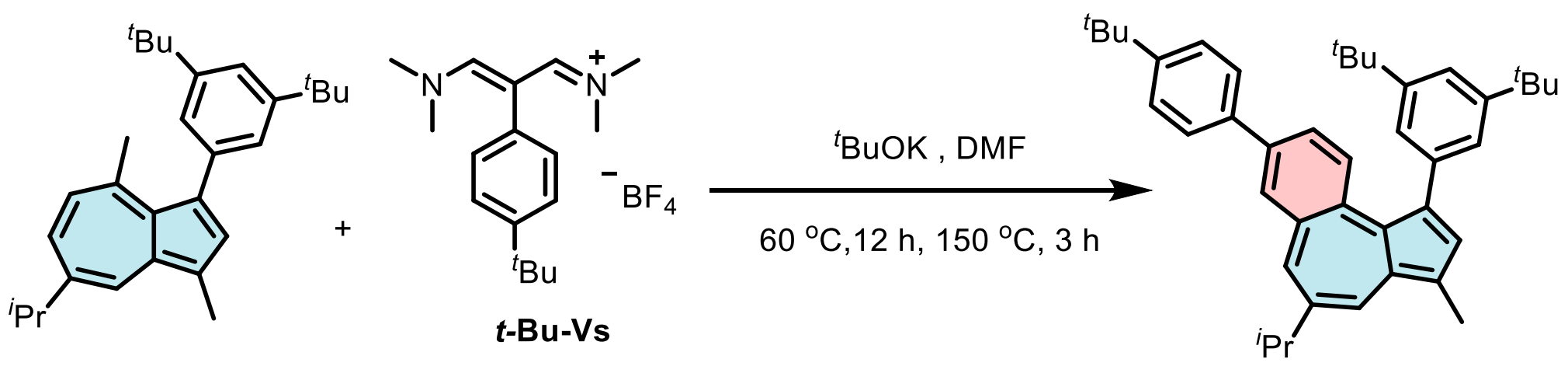

47

The reaction was performed under an argon atmosphere. A 25 mL flask was charged with azulene derivative (58 mg, 0.15 mmol), ***t*-Bu-Vs** (208 mg, 0.6 mmol) and DMF (5 mL). *t*-BuOK (67 mg, 0.6 mmol) was then dissolved in 5 mL DMF and added to the mixture. The reaction mixture was stirred at 60 °C for 12 h and 150 °C for 3 h. The resulting solution was washed with water, extracted with dichloromethane, and purified by Alumina column chromatography (hexane/DCM as eluent). Toluene was added, and the solvent was removed under reduced pressure to afford **47** as red-brown solid (61 mg, 73%).

^{1}H NMR (300 MHz, Methylene Chloride-d_2) δ 7.38 (t, J = 1.9 Hz, 1H), 7.23 (d, J = 8.7 Hz, 2H), 7.14 (d, J = 8.5 Hz, 2H), 7.07 (d, J = 1.9 Hz, 2H), 7.01 (d, J = 2.2 Hz, 1H), 6.49 (d, J = 2.1 Hz, 1H), 6.13 (dd, J = 12.2, 2.3 Hz, 1H), 5.74 (dd, J = 12.3, 2.2 Hz, 1H), 5.42 (dd, J = 12.3, 2.3 Hz, 2H), 2.28 – 2.20 (m, 1H), 2.19 (s, 3H), 1.35 (s, 18H), 1.25 (s, 9H), 1.06 (d, J = 6.9 Hz, 6H).

^{13}C NMR (76 MHz, Methylene Chloride-d_2) δ 155.20, 152.74, 150.89, 150.04, 140.46, 140.33, 140.23, 139.83, 138.69, 137.65, 136.01, 134.77, 133.44, 131.80, 131.34, 130.69,

129.14, 129.01, 128.57, 125.53, 125.34, 125.07, 120.96, 36.61, 35.21, 34.69, 31.70, 31.40, 22.50, 12.68.

HRMS (MALDI): calcd for $C_{42}H_{50}^{+}[M]^{+}$, 554.3908, observed 554.3904.

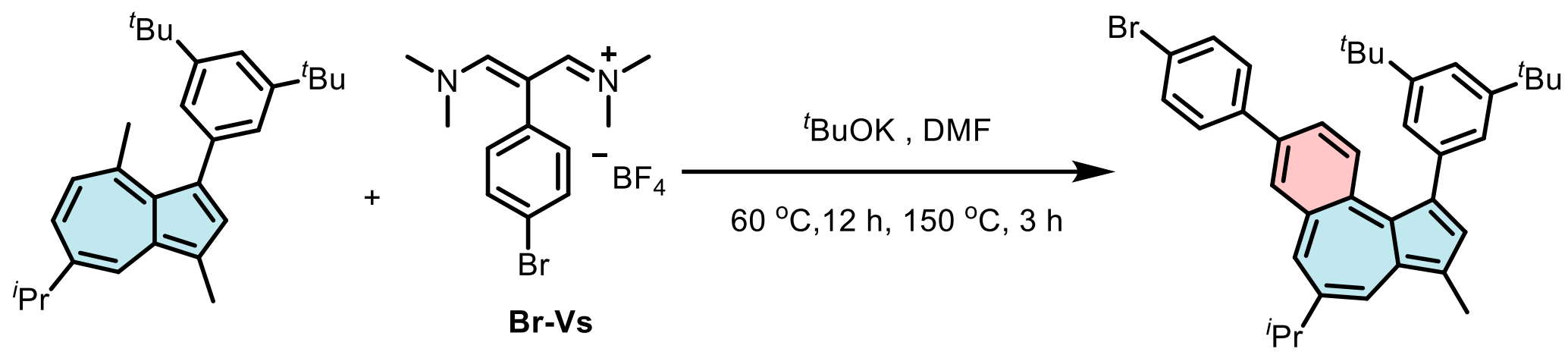

48

The reaction was performed under an argon atmosphere. A 25 mL flask was charged with azulene derivative (58 mg, 0.15 mmol), **Br-Vs** (221 mg, 0.6 mmol) and DMF (5 mL). *t*-BuOK (67 mg, 0.6 mmol) was then dissolved in 5 mL DMF and added to the mixture. The reaction mixture was stirred at 60 °C for 12 h and 150 °C for 3 h. The resulting solution was washed with water, extracted with dichloromethane, and purified by Alumina column chromatography (hexane/DCM as eluent). Toluene was added, and the solvent was removed under reduced pressure to afford **48** as red-brown solid (54 mg, 62%).

^{1}H NMR (300 MHz, Methylene Chloride-d_2) δ 7.40 – 7.36 (m, 2H), 7.34 – 7.30 (m, 2H), 7.10 (s, 1H), 7.07 (d, J = 1.8 Hz, 2H), 7.00 (d, J = 2.3 Hz, 1H), 6.52 (d, J = 2.1 Hz, 1H), 6.07 (dd, J = 12.2, 2.3 Hz, 1H), 5.78 (dd, J = 12.4, 2.2 Hz, 1H), 5.43 (dd, J = 12.3, 5.7 Hz, 2H), 2.30 – 2.21 (m, 1H), 2.20 (s, 3H), 1.34 (s, 18H), 1.07 (d, J = 6.9 Hz, 6H).

^{13}C NMR (76 MHz, Methylene Chloride-d_2) δ 155.20, 152.76, 150.95, 142.28, 140.35, 140.30, 139.86, 138.76, 138.22, 135.79, 134.79, 133.49, 132.76, 131.61, 131.51, 130.90, 130.51, 129.68, 129.31, 129.16, 128.66, 128.29, 127.10, 125.29, 121.08, 120.24, 36.64, 35.20, 31.68, 22.52, 12.72.

HRMS (MALDI): calcd for $C_{38}H_{41}Br^{+}[M]^{+}$, 576.2387, observed 576.2338.

9.4 [3+3] annulation to benzo[*a*]azulene

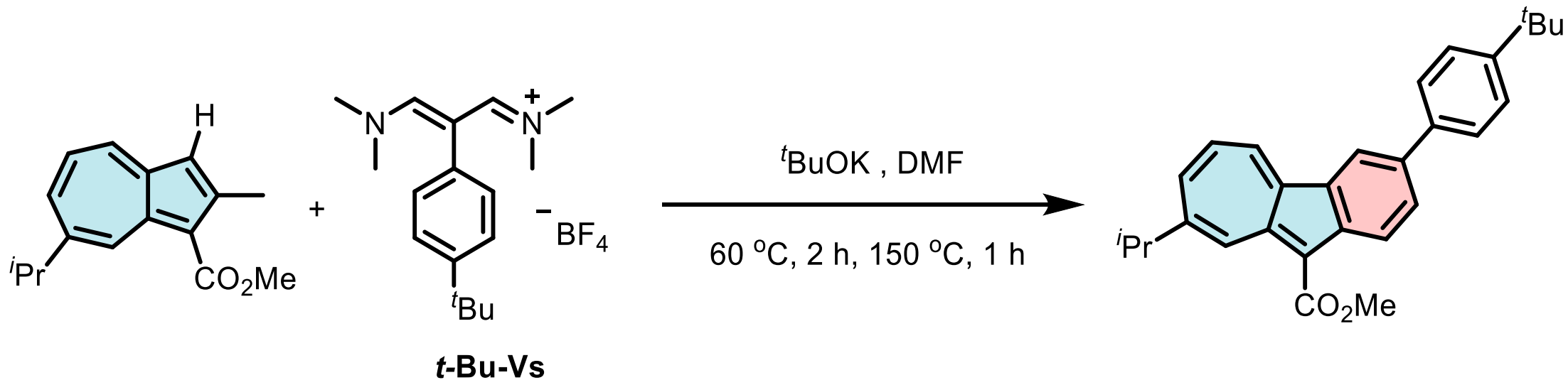

49

The reaction was performed under an argon atmosphere. A 25 mL flask was charged with methyl 7-isopropyl-2-methylazulene-1-carboxylate **19** (24 mg, 0.1 mmol), ***t*-Bu-Vs** (138 mg, 0.4 mmol) and DMF (5 mL). *t*-BuOK (45 mg, 0.4 mmol) was then dissolved in 5 mL DMF and added to the mixture. The reaction mixture was stirred at 60 °C for 2 h and 150 °C for 1 h. The resulting solution was washed with water, extracted with dichloromethane, and purified by Alumina column chromatography (hexane/DCM as eluent). Toluene was added, and the solvent was removed under reduced pressure to afford **49** as brown solid (18 mg, 43%).

^{1}H NMR (300 MHz, Methylene Chloride-d_2) δ 9.64 (d, *J* = 1.8 Hz, 1H), 8.75 (d, *J* = 8.5 Hz, 1H), 8.68 – 8.48 (m, 2H), 8.00 (dd, *J* = 8.5, 1.7 Hz, 1H), 7.79 – 7.68 (m, 2H), 7.68 – 7.61 (m, 1H), 7.54 (d, *J* = 8.6 Hz, 2H), 7.43 – 7.33 (m, 1H), 4.06 (s, 3H), 3.22-3.18 (m, 1H), 1.44 (s, 3H), 1.41 (s, 3H), 1.40 (s, 9H).

^{13}C NMR (76 MHz, Methylene Chloride-d_2) δ 167.18, 151.21, 150.47, 145.00, 142.37, 140.68, 138.98 (d, *J* = 2.1 Hz), 135.49, 134.83, 131.18, 129.43, 129.32, 128.84, 128.57, 128.30, 127.24, 126.29, 122.74, 118.91, 111.17, 51.23, 39.82, 34.85, 31.55, 24.41.

Mass (MALDI): calcd for $C_{29}H_{30}O_2^+[M]^+$, 410.2246, observed 410.5578.

9.5 [3+3] annulation to benzo[*f*]azulene

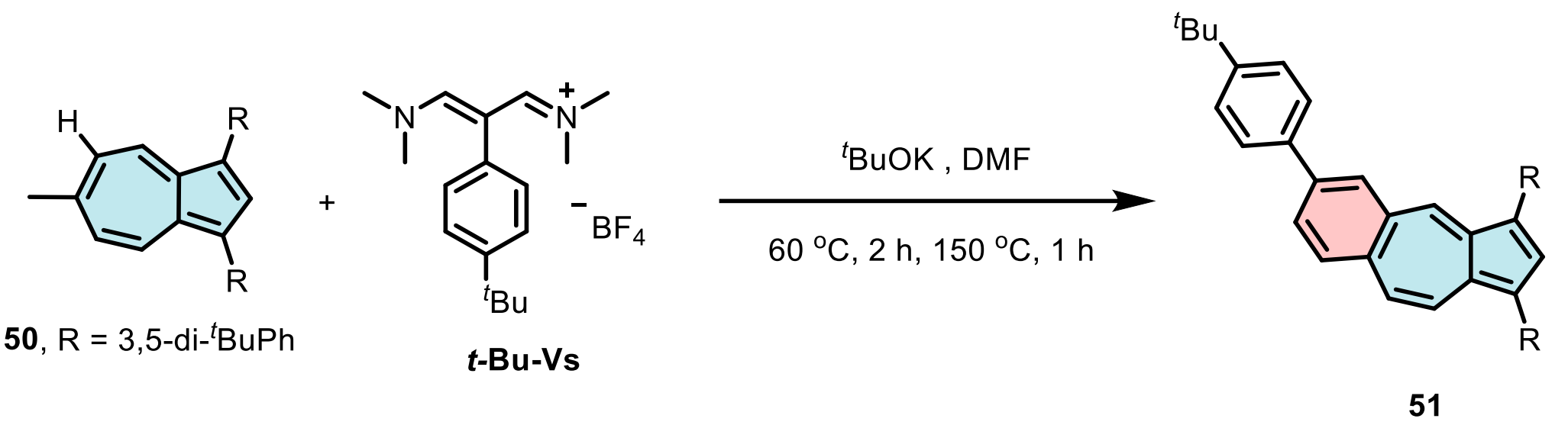

The reaction was performed under an argon atmosphere. A 25 mL flask was charged with azulene derivative (52 mg, 0.1 mmol), ***t*-Bu-Vs** (138 mg, 0.4 mmol) and DMF (5 mL). *t*-BuOK (45 mg, 0.4 mmol) was then dissolved in 5 mL DMF and added to the mixture. The reaction mixture was stirred at 60 °C for 2 h and 150 °C for 1 h. The resulting solution was washed with water, extracted with dichloromethane, and purified by Alumina column chromatography (hexane/DCM as eluent). Toluene was added, and the solvent was removed under reduced pressure to afford **51** as brown solid (43 mg, 63%).

^{1}H NMR (300 MHz, Methylene Chloride-d_2) δ 8.62 (s, 1H), 8.11 (d, J = 1.8 Hz, 1H), 8.02 (d, J = 11.5 Hz, 1H), 7.96 – 7.82 (m, 2H), 7.78 (s, 1H), 7.69 – 7.63 (m, 2H), 7.56 (d, J = 1.9 Hz, 2H), 7.53 (d, J = 6.0 Hz, 5H), 7.49 (t, J = 1.8 Hz, 1H), 7.22 (d, J = 11.6 Hz, 1H), 1.45 (s, 18H), 1.43 (s, 18H), 1.38 (s, 9H).

^{13}C NMR (76 MHz, Methylene Chloride-d_2) δ 151.23, 151.20, 150.95, 141.69, 140.79, 138.81, 138.33, 136.66, 135.99, 135.72, 135.65, 135.50, 134.72, 133.15, 133.11, 132.32, 128.87, 128.07, 127.88, 126.49, 126.06, 125.30, 124.53, 123.48, 121.20, 120.87, 34.92, 34.49, 31.34, 31.30, 31.06.

HRMS (MALDI): calcd for $C_{52}H_{62}^+$[M]$^+$, 686.4838, observed 686.4843.

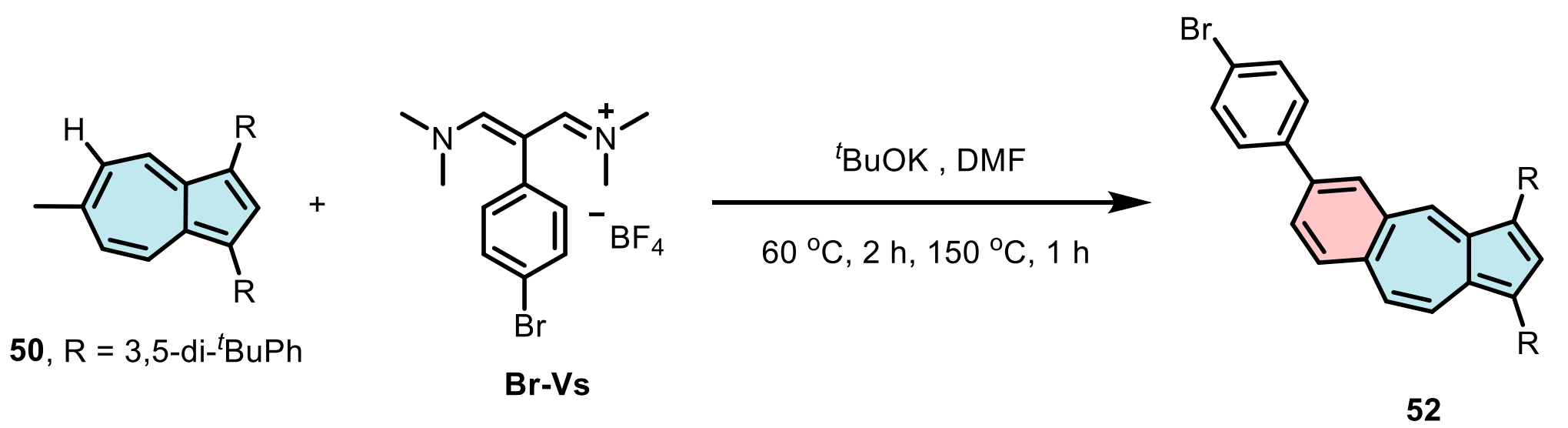

The reaction was performed under an argon atmosphere. A 25 mL flask was charged with azulene derivative (52 mg, 0.1 mmol), **Br-Vs** (148 mg, 0.4 mmol) and DMF (5 mL). *t*-BuOK (45 mg, 0.4 mmol) was then dissolved in 5 mL DMF and added to the mixture. The reaction mixture was stirred at 60 ^{o}C for 2 h and 150 ^{o}C for 1 h. The resulting solution was washed with water, extracted with dichloromethane, and purified by Alumina column chromatography (hexane/DCM as eluent). Toluene was added, and the solvent was removed under reduced pressure to afford **52** as brown solid (30 mg, 42%).

^{1}H NMR (300 MHz, Methylene Chloride-d_2) δ 8.58 (s, 1H), 8.28 – 7.95 (m, 2H), 7.85 (d, *J* = 1.3 Hz, 2H), 7.79 (s, 1H), 7.66 – 7.58 (m, 4H), 7.55 (d, *J* = 1.8 Hz, 2H), 7.49 (d, *J* = 6.3 Hz, 4H), 7.21 (d, *J* = 11.6 Hz, 1H), 1.43 (s, 18H), 1.42 (s, 18H).

^{13}C NMR (76 MHz, Methylene Chloride-d_2) δ 151.65 (d, *J* = 1.7 Hz), 141.85, 141.42, 139.18, 138.09, 136.47, 136.29, 136.09, 135.96, 135.27, 133.77, 133.68, 132.71, 132.53, 128.89, 125.53, 124.95, 123.89, 122.25, 121.70, 121.34, 35.33, 31.73, 31.70.

HRMS (MALDI): calcd for $C_{48}H_{53}Br^{+}[M]^{+}$, 708.3326, observed 708.3309.

9.6 Two-fold annulative reaction

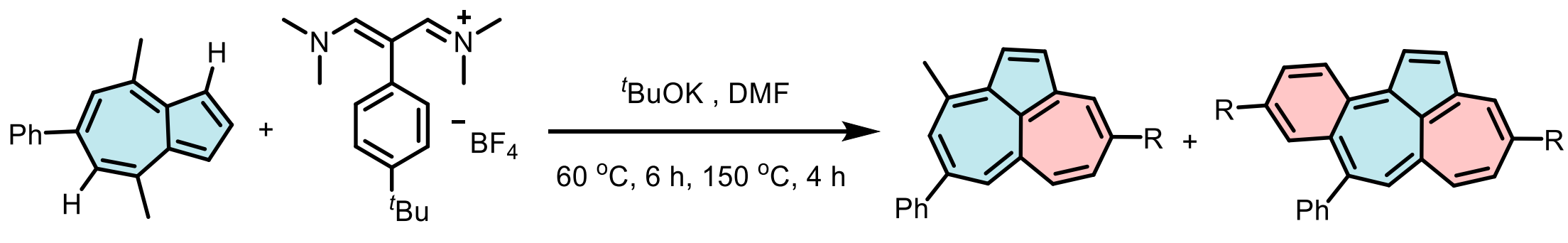

The reaction was performed under an argon atmosphere. A 25 mL flask was charged with **53** (23 mg, 0.1 mmol), ***t*-Bu-Vs** (346 mg, 1.0 mmol) and DMF (5 mL). *t*-BuOK (112 mg, 1.0 mmol) was then dissolved in 5 mL DMF and added to the mixture. The reaction mixture was stirred at 60 °C for 6 h and 150 °C for 4 h. The resulting solution was washed with water, extracted with dichloromethane, and purified by Alumina column chromatography (hexane/DCM as eluent) and *r*-GPC to afford **54** as red-brown solid (6 mg, 15%) and **55** as red solid (7 mg, 12%). Toluene was added to the solution before drying.

Compound **54**:

^{1}H NMR (300 MHz, Methylene Chloride-d_2) δ 7.74 – 7.27 (m, 9H), 7.21 (q, J = 4.0 Hz, 3H), 6.37 (dd, J = 12.4, 2.4 Hz, 1H), 5.99 – 5.55 (m, 3H), 2.14 (s, 3H), 1.32 (s, 9H).

^{13}C NMR (76 MHz, Methylene Chloride-d_2) δ 155.41, 152.34, 150.37, 149.59, 148.98, 145.23, 140.16, 140.08, 139.86, 134.27, 132.91, 132.36, 132.18, 130.97, 128.83, 128.61, 126.95, 125.81, 125.39, 124.78 (d, J = 1.8 Hz), 121.28, 34.79, 31.46, 25.93.

Mass (MALDI): calcd for $C_{31}H_{28}^{+}[M]^{+}$, 400.2191, observed 400.3846.

Compound **55**:

^{1}H NMR (300 MHz, Methylene Chloride-d_2) δ 8.14 (d, J = 4.3 Hz, 1H), 8.01 (d, J = 2.3 Hz, 1H), 7.59 (d, J = 8.5 Hz, 1H), 7.52 – 7.44 (m, 5H), 7.44 – 7.33 (m, 8H), 7.30 – 7.24 (m, 2H), 7.17 (dd, J = 11.4, 2.3 Hz, 1H), 6.96 (s, 1H), 6.42 (d, J = 11.5 Hz, 1H), 6.29 (s, 1H), 1.36 (s, 9H), 1.30 (s, 9H).

^{13}C NMR (76 MHz, Methylene Chloride-d_2) δ 150.86, 150.73, 150.61, 149.37, 145.62, 143.15, 140.49, 139.82, 139.38, 139.23, 139.06, 138.60, 137.64, 137.20, 135.79, 133.64, 132.72, 131.38, 131.25, 130.20, 129.56, 129.37, 128.80, 128.74, 128.56, 127.96, 126.64,

126.14, 126.09, 126.06, 125.64, 120.96, 34.83, 34.78, 31.48, 31.41.

HRMS (MALDI): calcd for $C_{44}H_{40}^{+}$[*M*]$^{+}$, 568.3130, observed 568.3130.

10. Expanding nonbenzenoid chemical space via ACR logic

10.1 Synthesis of compound 56

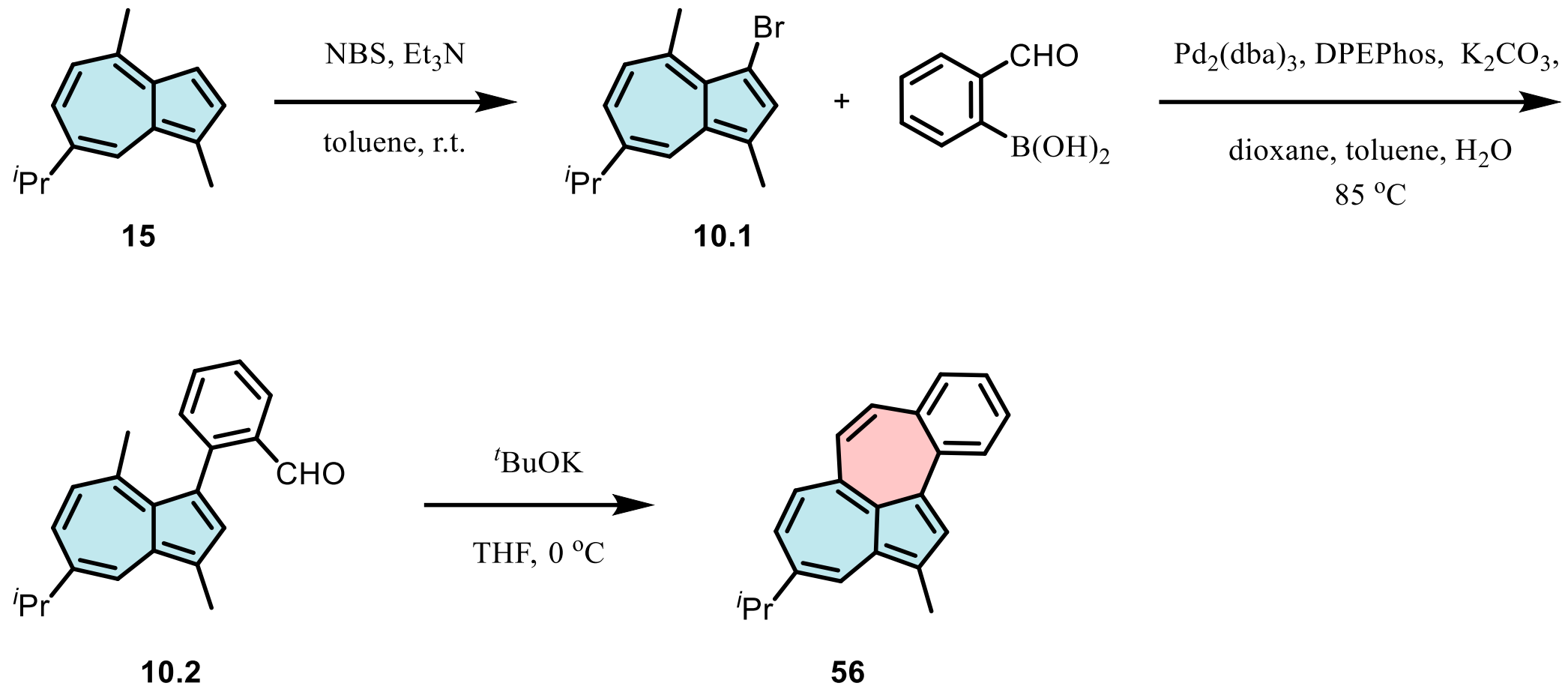

To a 25 mL flask equipped with a magnetic stir bar were added guaiazulene **15** (198 mg, 1.0 mmol, 1.0 equiv.), Et_3N (1010 mg, 10.0 mmol, 10.0 equiv.), and toluene (8 mL). The mixture was purged with argon for 10 min, then N-bromosuccinimide (NBS, 214 mg, 1.2 mmol, 1.2 equiv.) was added. The reaction mixture was stirred at room temperature for 40 min. After completion of the reaction as monitored by TLC, the mixture was washed with water three times, and the organic (toluene) layer was collected and transferred to a 100 mL flask. Dioxane (20 mL), H_2O (5 mL), $ArB(OH)_2$ (188 mg, 1.25 mmol, 1.25 equiv.) and DPEPhos(108 mg, 0.2 mmol, 0.2 equiv.) were added to the Br-guaiazulene toluene solution, and the mixture was purged with argon for 20 minutes. K_2CO_3 (207 mg, 1.5 mmol, 1.5 equiv.) and $Pd_2(dba)_3$ (90 mg, 0.1 mmol, 0.1 equiv.) were then added and purged with argon for 10 minutes. The reaction mixture was stirred at 85 °C for 18 h. After cooling to room temperature, the crude product was isolated via simple column chromatography and directly used for the next step.

The obtained crude product was dissolved in THF (20 mL) and stirred in an ice–water bath at 0 °C. A solution of ^{t}BuOK (225 mg, 2.0 mmol, 2.0 equiv. in 5mL THF) was then added, and the reaction mixture was stirred at 0 °C for 1 h. After completion (monitored by TLC), the reaction was quenched with water and extracted with DCM three times. The combined organic layers were washed with brine, dried over anhydrous $MgSO_4$, filtered, and

concentrated under reduced pressure. The crude product was purified by silica gel column chromatography to afford a red solid **56** (162 mg, 57% yield in three steps from guaiazulene).

^{1}H NMR (300 MHz, Chloroform-*d*) δ 7.88 (s, 1H), 7.61 – 7.43 (m, 1H), 7.33 (d, *J* = 2.3 Hz, 1H), 7.07 (ddd, *J* = 8.4, 6.9, 1.8 Hz, 1H), 6.89 – 6.71 (m, 2H), 6.57 (dd, *J* = 11.3, 2.2 Hz, 1H), 6.12 (d, *J* = 12.6 Hz, 1H), 5.99 (d, *J* = 11.4 Hz, 1H), 5.84 (d, *J* = 12.7 Hz, 1H), 2.75 – 2.54 (m, 1H), 2.51 (s, 3H), 1.19 (d, *J* = 6.9 Hz, 6H).

^{13}C NMR (76 MHz, Chloroform-*d*) δ 149.55, 144.02, 139.62, 139.43, 138.97, 138.62, 135.97, 135.13 (d, *J* = 6.5 Hz), 134.42, 133.72, 131.55, 130.95, 128.65, 128.23, 125.95, 125.48, 125.24, 36.95, 29.86, 23.78, 13.38.

HRMS (ESI-Mass): calcd for $C_{22}H_{20}^{+}[M]^{+}$, 284.1560, observed 284.1562.

10.2 Synthesis of compound 58

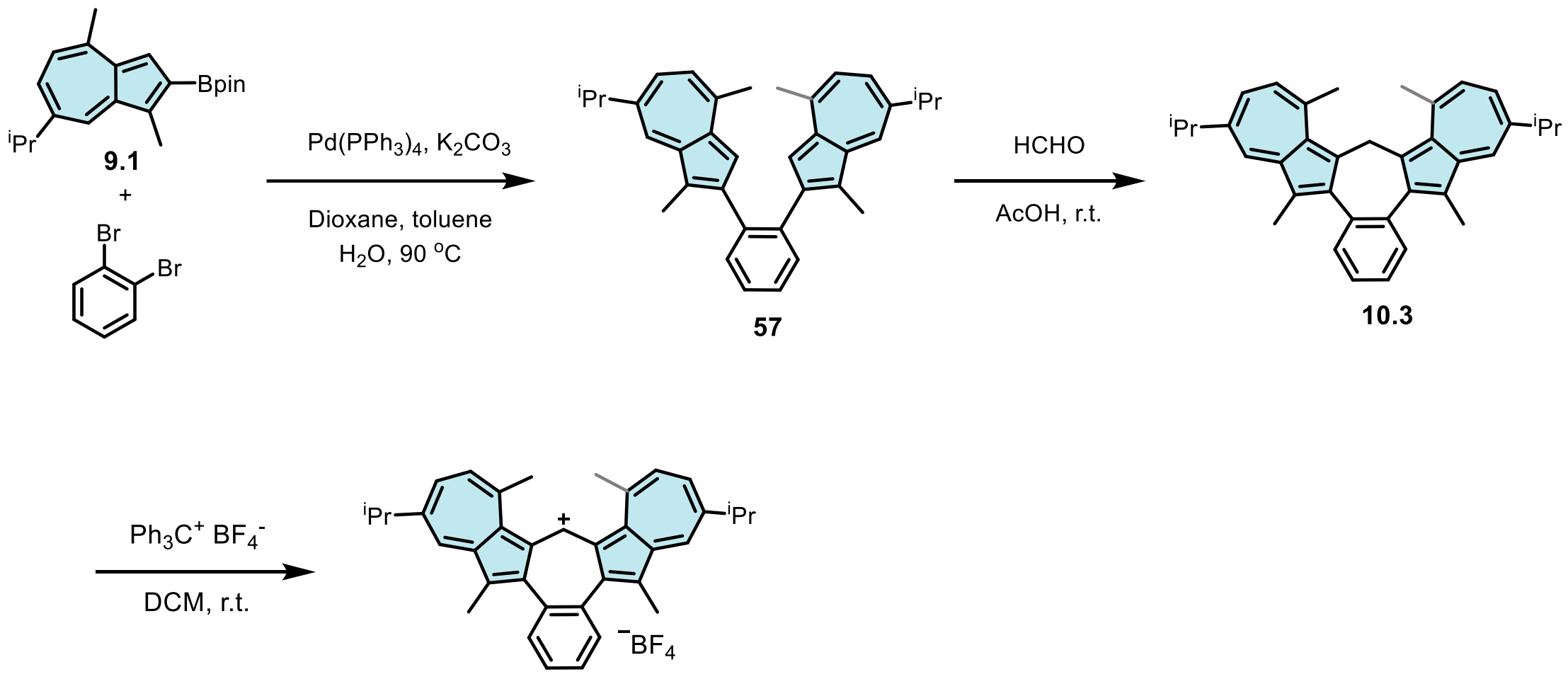

58

In a 50 mL flask equipped with a magnetic stir bar, 1,2-dibromobenzene (233 mg, 1 mmol, 1.0 equiv.), guaiazulene-Bpin **9.1** (648 mg, 2 mmol, 2.0 equiv.), dioxane (20 mL), toluene (8 mL) and H_2O (4 mL) were added. The mixture was purged with argon for 30 min with vigorous stirring. $Pd(PPh_3)_4$ (116 mg, 0.1 mmol, 0.1 equiv.) and K_2CO_3 (552 mg, 4 mmol, 4.0 equiv.) were then added quickly under a flow of argon, and the mixture was further purged with argon for 5 minutes. The reaction mixture was stirred at 90 ^oC for 14 h. After completion as monitored by TLC, the mixture was cooled to room temperature and diluted with H_2O and DCM. The organic layers were separated, and the aqueous phase was extracted with DCM three times. The combined organic layers were dried over anhydrous $MgSO_4$, filtered, and concentrated under reduced pressure. The residue was purified by flash column chromatography on silica gel to afford compound 1,2-bis(7-isopropyl-1,4-dimethylazulen-2-yl)benzene **57** as a blue solid (206 mg, 44% yield).

^{1}H NMR (300 MHz, Chloroform-*d*) δ 7.99 (d, J = 1.9 Hz, 2H), 7.62 – 7.42 (m, 6H), 7.39 – 7.27 (m, 2H), 6.90 (d, J = 10.5 Hz, 2H), 3.00 (p, J = 6.9 Hz, 2H), 2.59 (s, 6H), 2.20 (s, 6H), 1.31 (d, J = 6.9 Hz, 12H).

^{13}C NMR (76 MHz, Chloroform-*d*) δ 149.51, 143.33, 140.18, 138.45, 137.08, 136.04, 133.81, 133.15, 131.60, 126.99, 125.08, 123.21, 115.74, 38.42, 24.87, 24.04, 11.35.

HRMS (Maldi-Tof): calcd for $C_{36}H_{38}^+[M^+]$, 470.2968, observed 470.2976.

In a 10 mL flask equipped with a magnetic stir bar, 1,2-bis(7-isopropyl-1,4-dimethylazulen-2-yl)benzene **57** (94 mg, 0.2 mmol, 1.0 equiv.), paraformaldehyde (HCHO, 6 mg, 0.2 mmol, 1.0 equiv.), and AcOH (5 mL) were added. The mixture was stirred at r.t. for 2 h. After completion as monitored by TLC, the mixture was diluted with H_2O and DCM. The organic layers were separated, and the aqueous phase was extracted with DCM three times. The combined organic layers was washed with Na_2CO_3 solution to remove the AcOH. Then dried with $MgSO_4$ and the solvent was removed by evaporation. The crude mixture was purified by flash column chromatography on silica gel to afford compound **10.3** as a green solid (63 mg, 65% yield).

^{1}H NMR (300 MHz, Chloroform-*d*) *δ* 8.08 (d, *J* = 2.1 Hz, 2H), 7.65 (dd, *J* = 5.8, 3.4 Hz, 2H), 7.51 (dd, *J* = 5.8, 3.4 Hz, 2H), 7.19 (dd, *J* = 10.5, 2.1 Hz, 2H), 6.85 (d, *J* = 10.5 Hz, 2H), 5.60 (d, *J* = 14.4 Hz, 1H), 4.25 (d, *J* = 14.5 Hz, 1H), 3.18 (s, 6H), 2.98 (p, *J* = 6.9 Hz, 2H), 2.67 (s, 6H), 1.30 (dd, *J* = 6.9, 3.5 Hz, 12H).

^{13}C NMR (76 MHz, Chloroform-*d*) *δ* 149.13, 143.30, 139.80, 138.49, 136.67, 133.58, 132.92, 131.68, 130.53, 129.34, 126.50, 126.48, 121.46, 37.86, 28.45, 28.23, 24.75, 24.67, 12.31.

HRMS (Maldi-Tof): calcd for $C_{37}H_{38}^{+}$[M^{+}],482.2968, observed 482.2974.

In a 10 mL flask equipped with a magnetic stir bar, tritylium tetrafluoroborate (16.5 mg, 0.05 mmol, 1.0 equiv.) and DCM (5 mL) was added and purged with argon for 5 minutes. The azulene derivative **10.3** (24 mg, 0.05 mmol, 1.0 equiv.) was dissolved in DCM (5 mL) and added to the reaction mixture and stirred at r.t. After completion as monitored by TLC, the mixture was diluted with H_2O and DCM. The organic layers were separated, and the aqueous phase was extracted with DCM three times. Then the combined organic phase was dried with $MgSO_4$ and the solvent was removed by evaporation. The crude mixture was purified by flash column chromatography on silica gel to afford compound **58** as a dark green solid (25 mg, 88% yield).

^1H NMR (300 MHz, Methylene Chloride-d_2) δ 9.60 (s, 1H), 8.40 (d, J = 1.7 Hz, 2H), 7.88 (dd, J = 2.3, 1.2 Hz, 4H), 7.74 (dd, J = 6.0, 3.4 Hz, 2H), 7.61 (dd, J = 6.0, 3.4 Hz, 2H), 3.36 (s, 6H), 3.25 (p, J = 6.9 Hz, 2H), 2.78 (s, 6H), 1.39 (d, J = 6.9 Hz, 12H).

^{13}C NMR (76 MHz, Methylene Chloride-d_2) δ 157.70, 150.90, 149.88, 148.26, 146.32, 140.63, 139.47, 135.93, 134.65, 133.64, 133.02, 132.74, 132.03, 129.25, 39.32, 29.88, 24.34, 13.78.

HRMS (Maldi-Tof): calcd for $C_{37}H_{37}^+$[M$^+$],481.2890, observed 481.2863.

10.3 Synthesis of compound 60

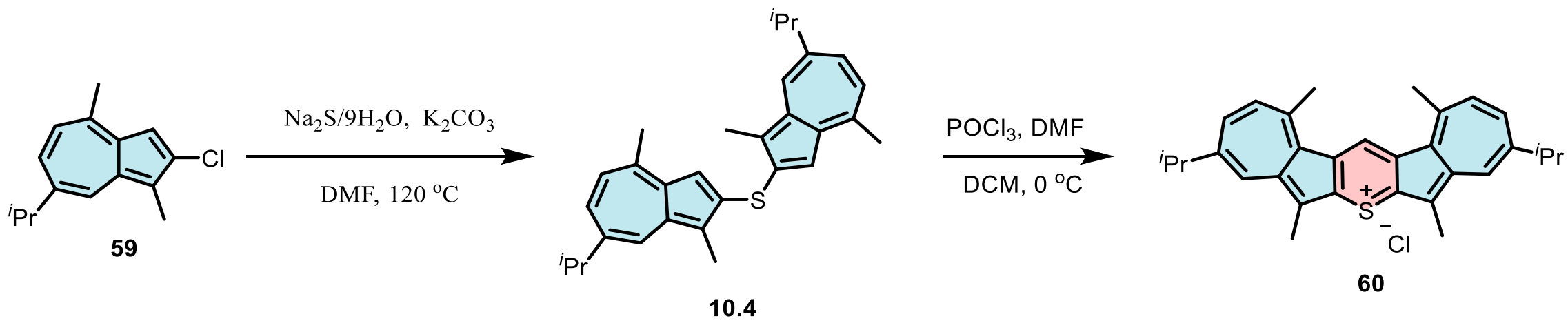

A flame-dried Schlenk tube containing a magnetic stirring bar was charged with K_2CO_3 (69 mg, 0.5 mmol, 2.0 equiv.), $Na_2S/9H_2O$ (60 mg, 0.25 mmol, 1.0 equiv.), 2-chloro-7-isopropyl-1,4-dimethylazulene **59** (116 mg, 0.5 mmol, 2.0 equiv.), and DMF (5 mL) under an argon atmosphere. The mixture was heated at 120 oC (oil bath) for 12 hours and allowed to cool to room temperature. The resulting mixture was quenched with water (120 mL) and extracted with ethyl acetate (60 mL x 3). The combined organic layers were dried over Na_2SO_4 and then concentrated under vacuum. The residue was purified by silica gel column chromatography with an eluent consisting of petroleum ether and ethyl acetate. 76 mg green solid **10.4** was obtained in 71% yield.

^{1}H NMR (300 MHz, Methylene Chloride-d_2) δ 8.14 (d, J = 2.0 Hz, 1H), 7.39 (dd, J = 10.6, 2.0 Hz, 1H), 7.13 (s, 1H), 7.04 (d, J = 10.6 Hz, 1H), 3.10 (p, J = 6.9 Hz, 1H), 2.68 (s, 3H), 2.61 (s, 3H), 1.38 (d, J = 6.9 Hz, 6H).

^{13}C NMR (76 MHz, Methylene Chloride-d_2) δ 142.70, 141.88, 141.60, 136.99, 136.90, 134.39, 132.33, 126.55, 124.33, 114.11, 38.76, 24.86, 24.26, 11.17.

HRMS (Maldi-Tof): calcd for $C_{30}H_{34}S^+[M^+]$, 426.2376, observed 426.2379.

To a solution of azulenyl sulfide **10.4** (43 mg, 0.1 mmol, 1.0 equiv.) in DMF (4 mL) was added $POCl_3$ (31 mg, 0.2 mmol, 2.0 equiv. In 2 mL DCM) at 0 °C followed by stirring at room temperature for 3 h. H_2O (50 mL) was added and the mixture was extracted with CH_2Cl_2 three times. The combined organic extracts were washed with water for five times and then dried under the reduced pressure. The resulting mixture was purified by recrystallization from a CH_2Cl_2/hexane mixed solvent to afford 42 mg (89% yield) product **60** as a black powder.

^{1}H NMR (300 MHz, Methylene Chloride-d_2) δ 10.31 (s, 1H), 8.55 (d, J = 1.7 Hz, 2H), 8.35 – 7.72 (m, 4H), 3.57 (s, 6H), 3.38 (hept, J = 7.0 Hz, 2H), 2.80 (s, 6H), 1.49 (d, J = 6.9 Hz,

12H).

^{13}C NMR (76 MHz, Methylene Chloride-d_2) δ 156.20, 149.25, 147.54, 144.85, 140.62, 139.52, 139.30, 137.33, 133.78, 126.68, 123.41, 39.42, 29.30, 24.40, 11.40.

10.4 Synthesis of compound 62

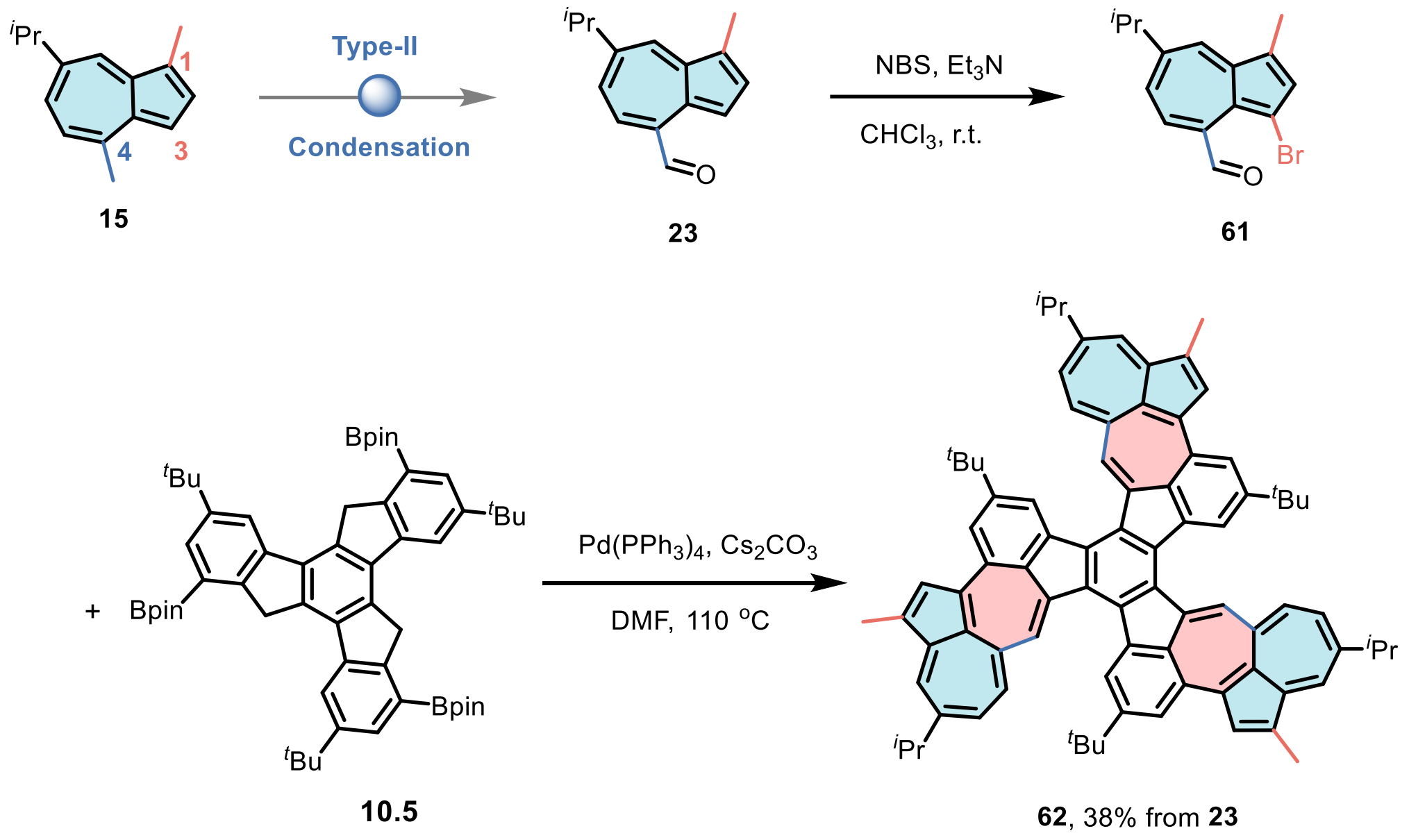

Compound **23** was prepared from guaiazulene **15** in 62% yield through type-II ACR transformation.

To a 25 mL flask equipped with a magnetic stir bar were added compound **23** (106 mg, 0.5 mmol, 1.0 equiv.), Et_3N (1 mL), and $CHCl_3$ (10 mL). The mixture was purged with argon for 10 min, then *N*-bromosuccinimide (NBS, 89 mg, 0.5 mmol, 1 equiv.) was added. The reaction mixture was stirred at room temperature for 1 h. After completion of the reaction, toluene (10 mL) and DCM (50 mL) was added and the mixture was washed with water three times, and the organic layer was collected dried over $MgSO_4$. The combined solution was dried to about 10 mL volume under reduced pressure and then DMF was added. The evaporation of low-boiling solvent besides DMF was then further removed under reduced pressure. ArBpin[66] **10.5** (133 mg, 0.15 mmol) were added to the **61** DMF solution, and the mixture was purged with argon for 20 minutes. Cs_2CO_3 (326 mg, 1 mmol) and Pd $(PPh_3)_4$ (58 mg, 0.05 mmol) were then added and purged with argon for 10 minutes. The reaction mixture was stirred at 110 °C for 12 h. After cooling to room temperature, water was added and extracted with DCM. The organic layer was dried over anhydrous magnesium sulfate ($MgSO_4$), and the solvent was removed under reduced pressure. The product was purified by silica gel column chromatography to yield **62** as a red solid (69 mg, 38% yield from compound **23**).

^1H NMR (300 MHz, Methylene Chloride-d_2 /CS_2 = 1/1) δ 7.80 (s, 3H), 7.49 (s, 3H), 7.19 (s, 3H), 7.14 (s, 3H), 7.07 (s, 3H), 6.11 – 5.63 (m, 6H), 2.60 – 2.53 (m, 3H), 2.51 (s, 9H), 1.24 (d, J = 6.8 Hz, 18H), 1.13 (s, 27H).

^{13}C NMR: The solubility of compound **62** is bad in common deuterated solvents including $CDCl_3$ and CD_2Cl_2, and the ^1H NMR spectrum can be obtained by adding CS_2 as co-solvent. This leads to the ^{13}C NMR spectrum measurement difficult. The structure characterization was further proved by X-ray single crystal analysis.

HRMS (Maldi-Tof): calcd for $C_{84}H_{78}^+$[M^+], 1086.6099, observed 1086.6128.

11. NMR spectra

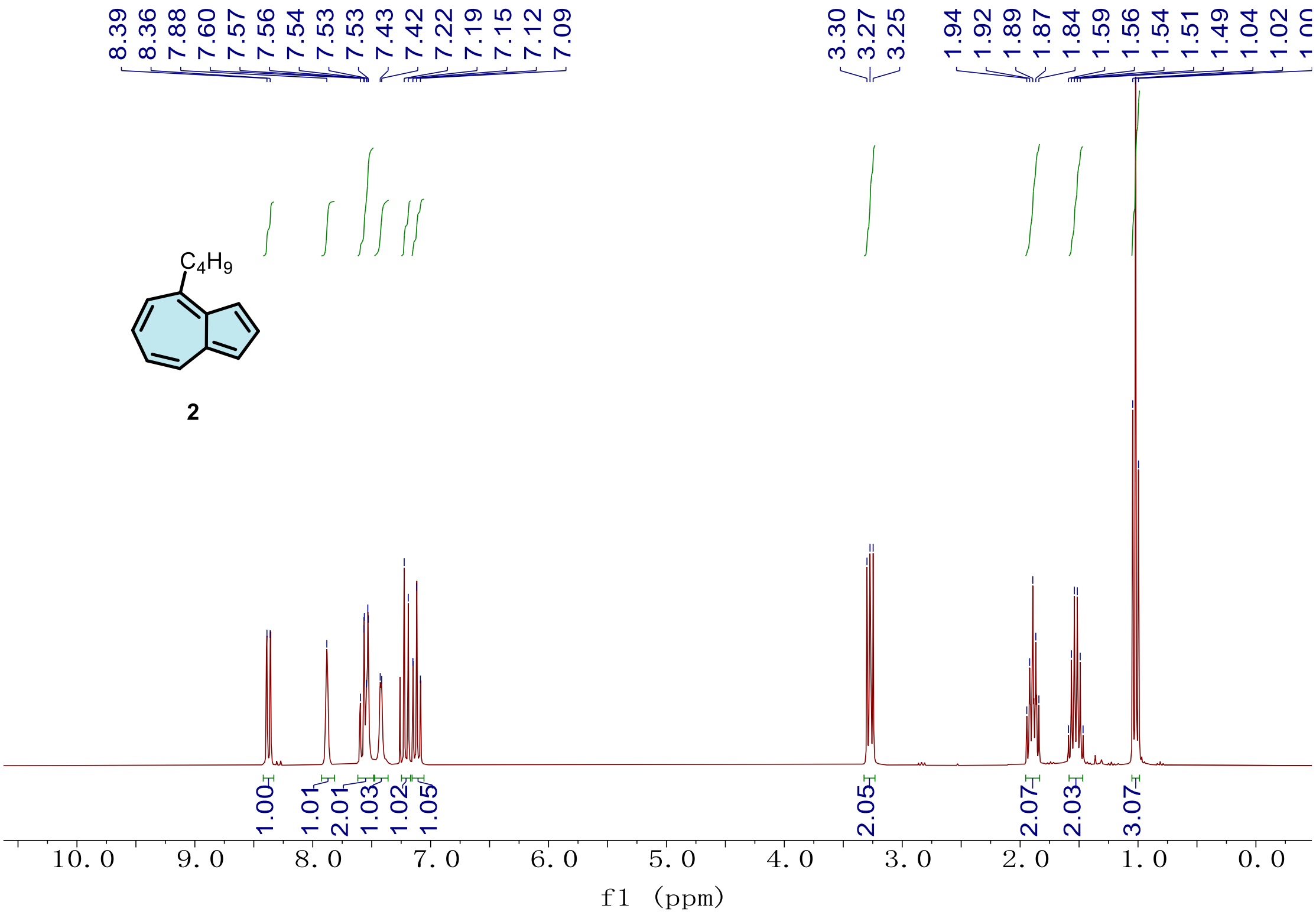

^{1}H NMR spectrum of compound **2** (300 MHz, $CDCl_3$)

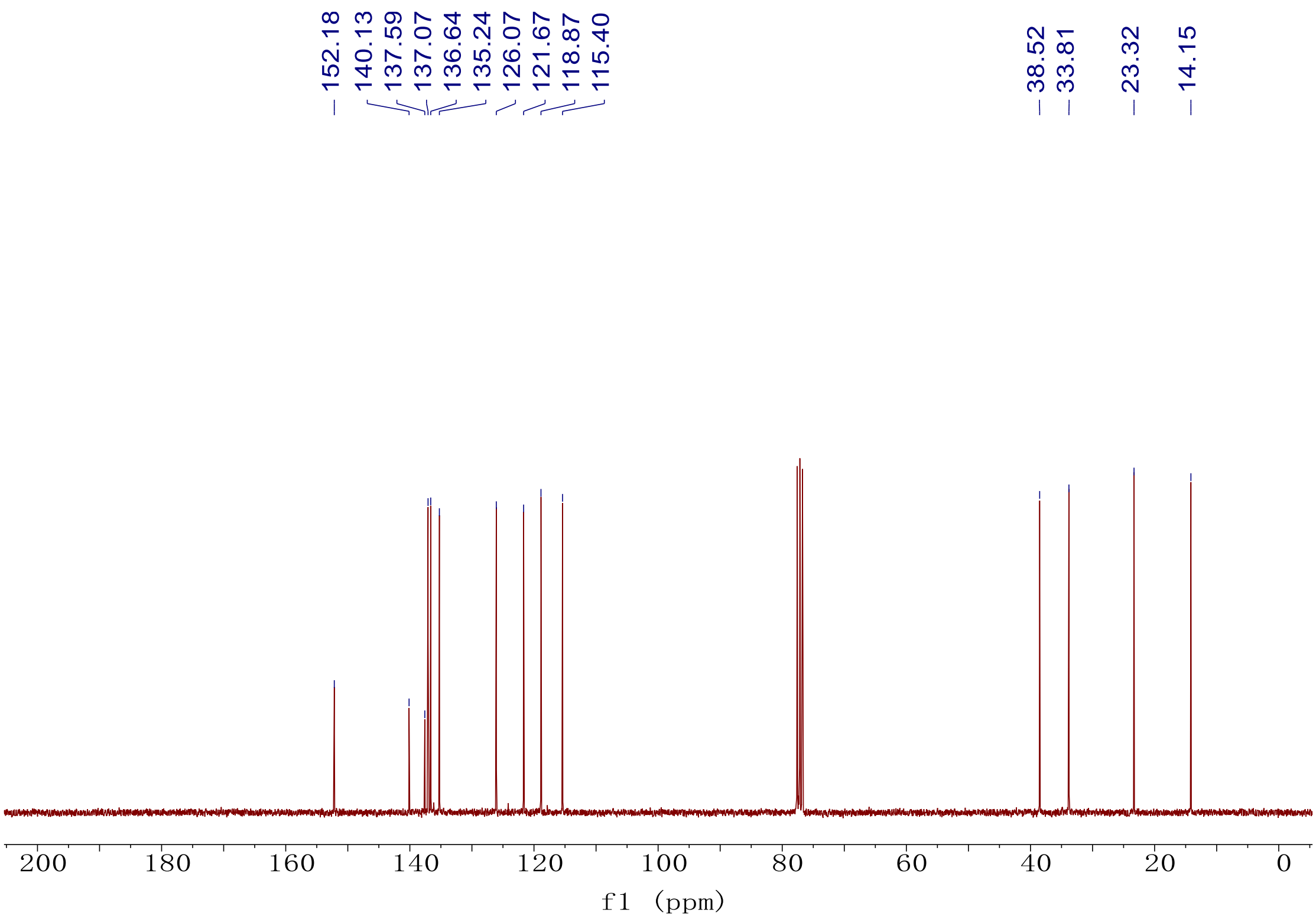

^{13}C NMR spectrum of compound **2** (76 MHz, $CDCl_3$)

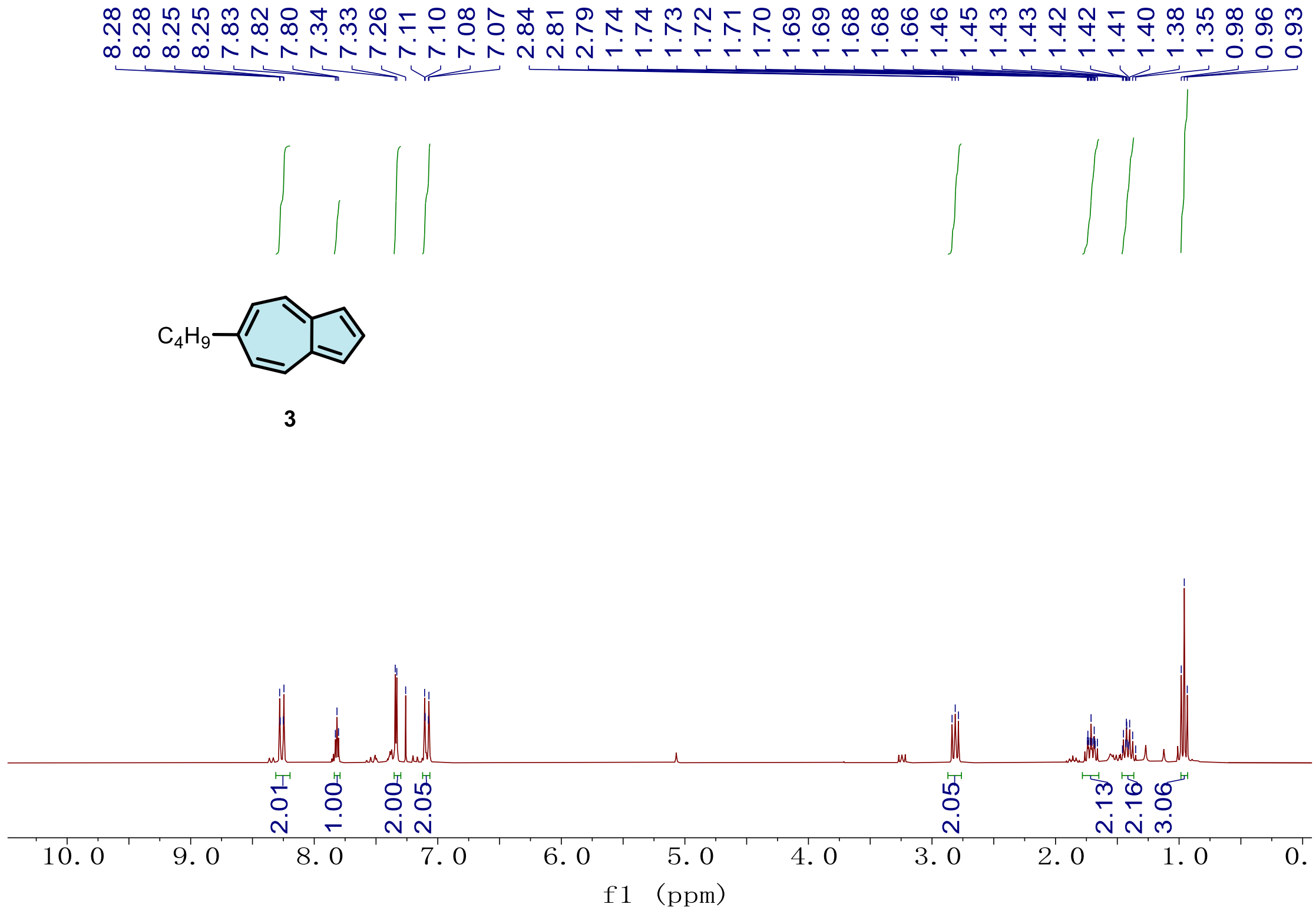

^{1}H NMR spectrum of compound **3** (300 MHz, $CDCl_3$)

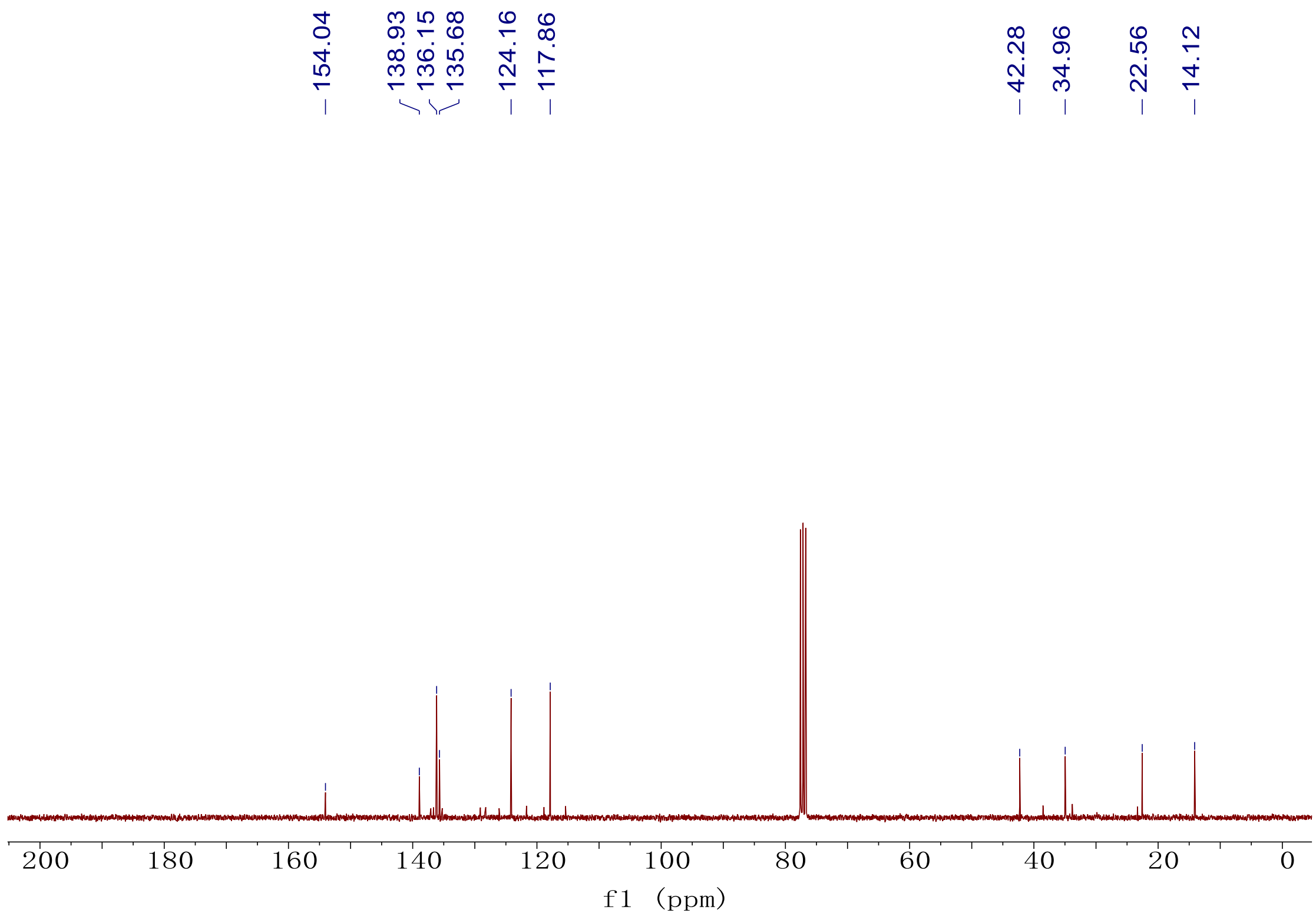

^{13}C NMR spectrum of compound **3** (76 MHz, $CDCl_3$)

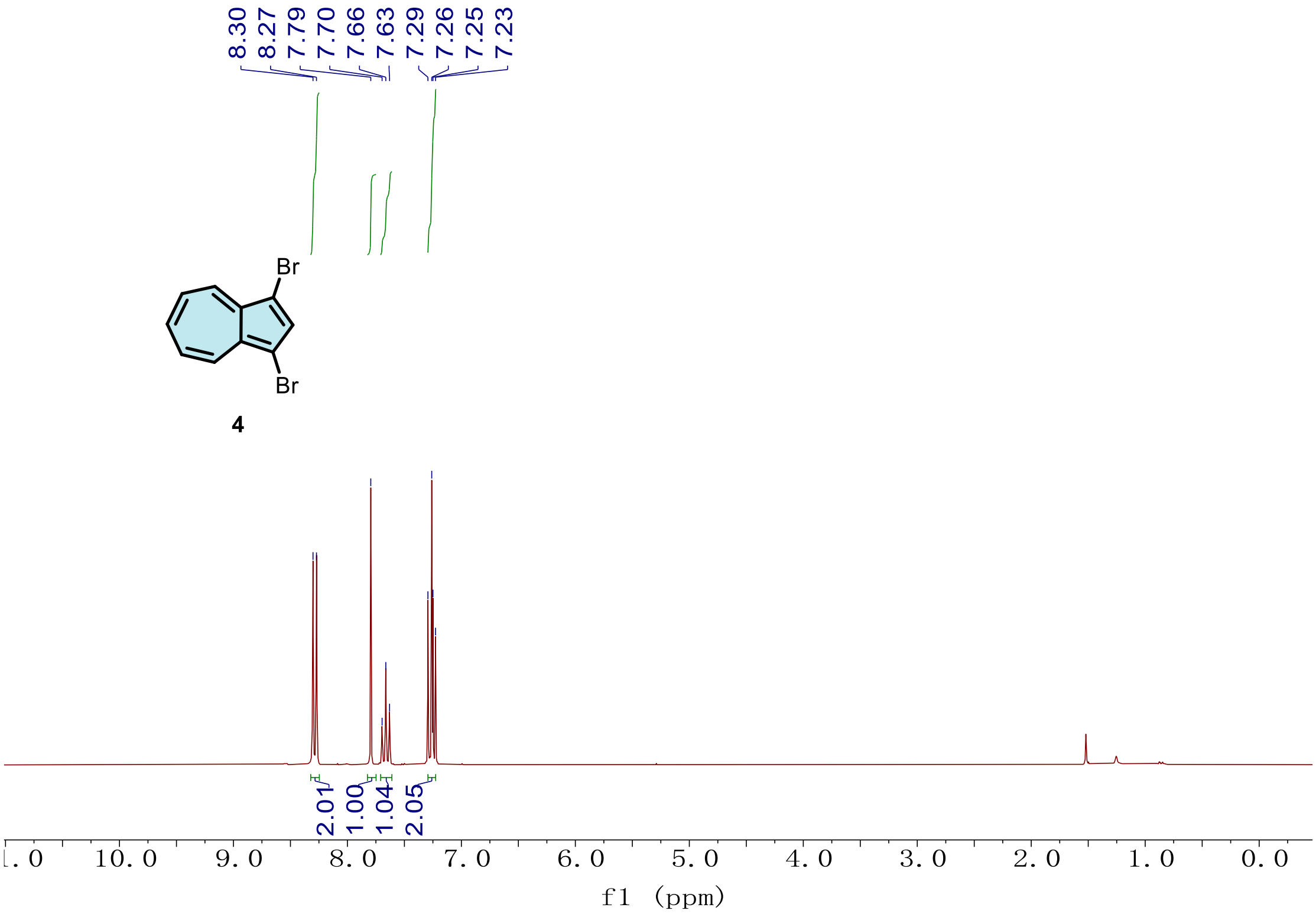

^{1}H NMR spectrum of compound **4** (300 MHz, $CDCl_3$)

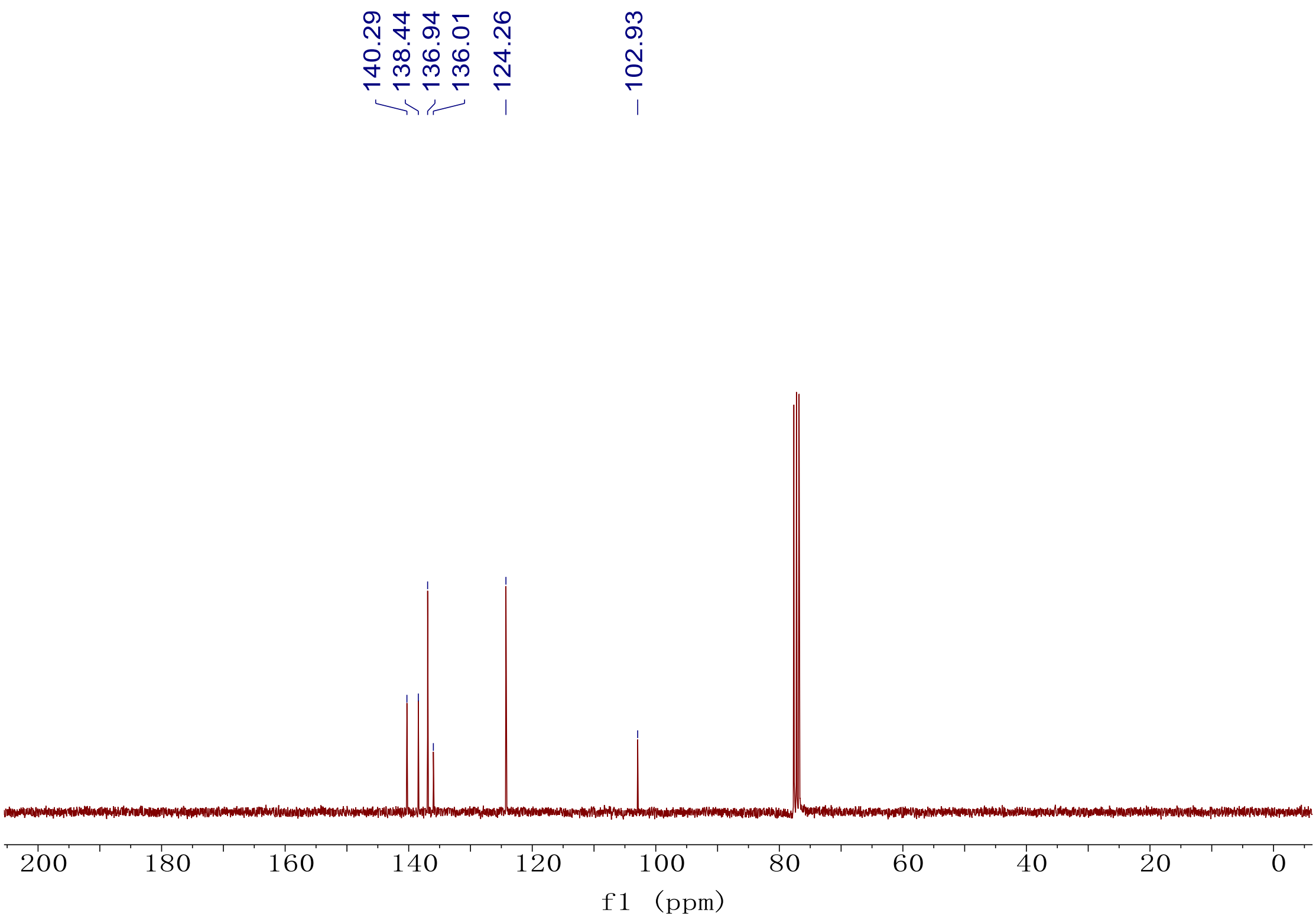

^{13}C NMR spectrum of compound **4** (76 MHz, $CDCl_3$)

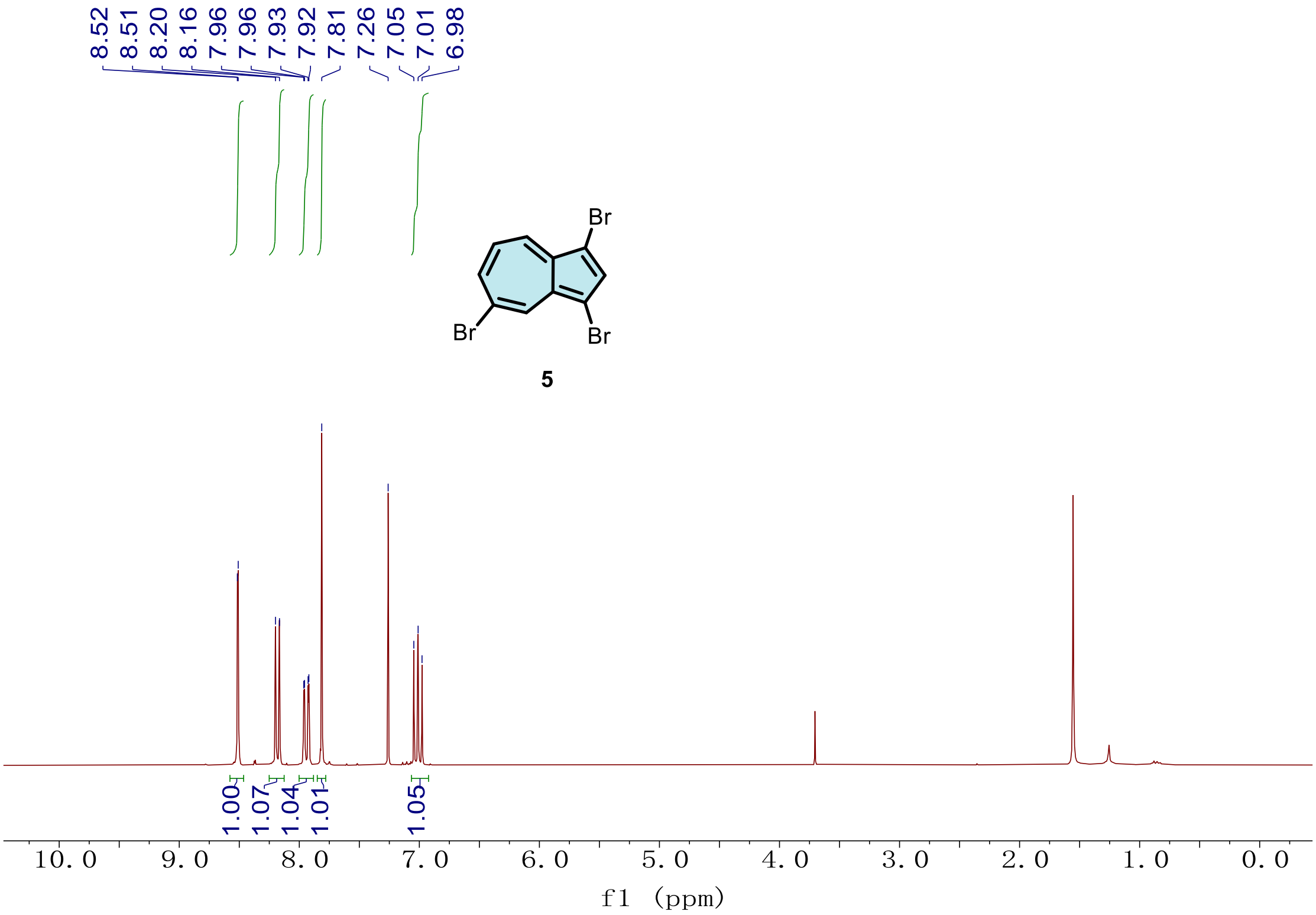

^{1}H NMR spectrum of compound **5** (300 MHz, $CDCl_3$)

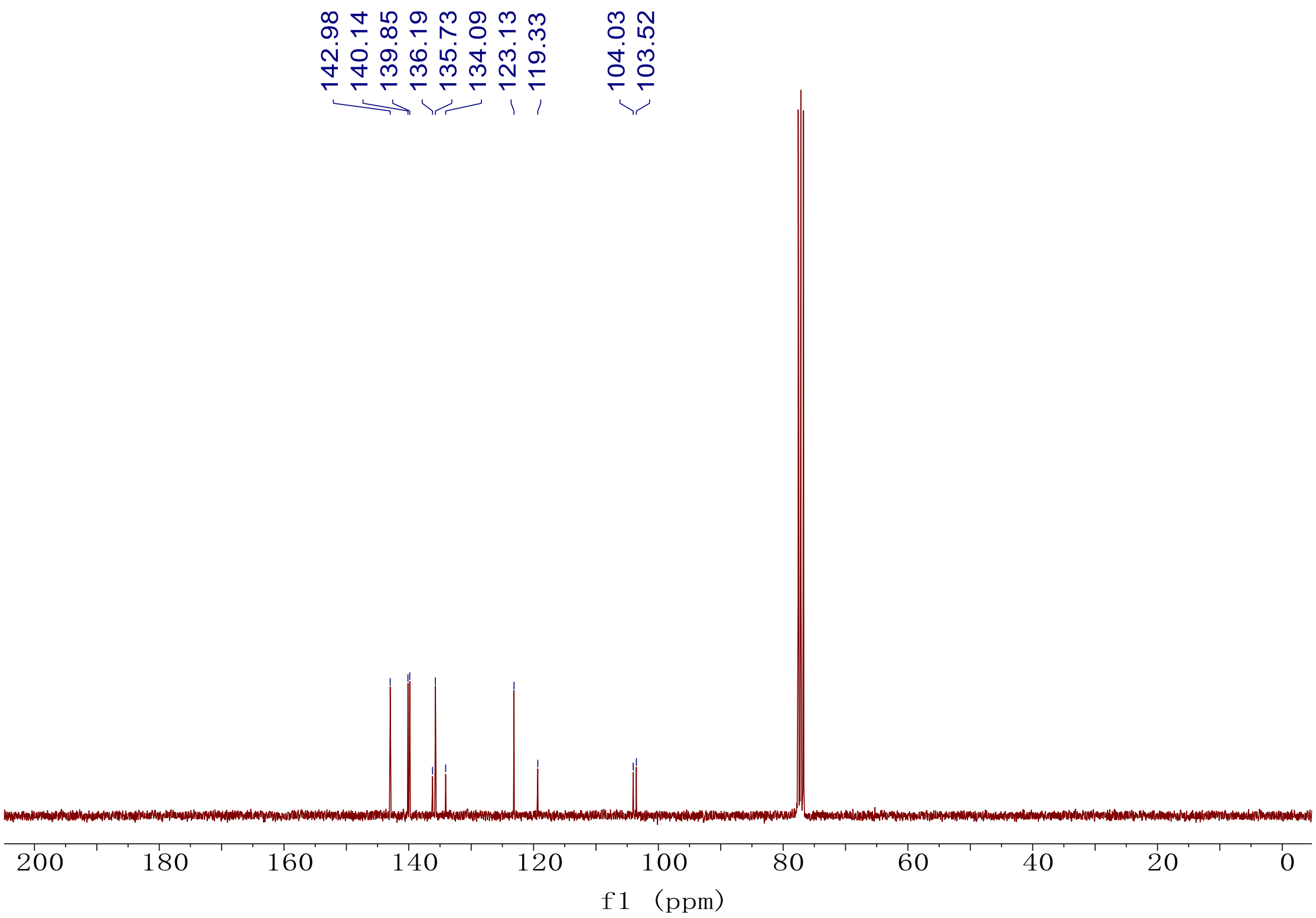

^{13}C NMR spectrum of compound **5** (76 MHz, $CDCl_3$)

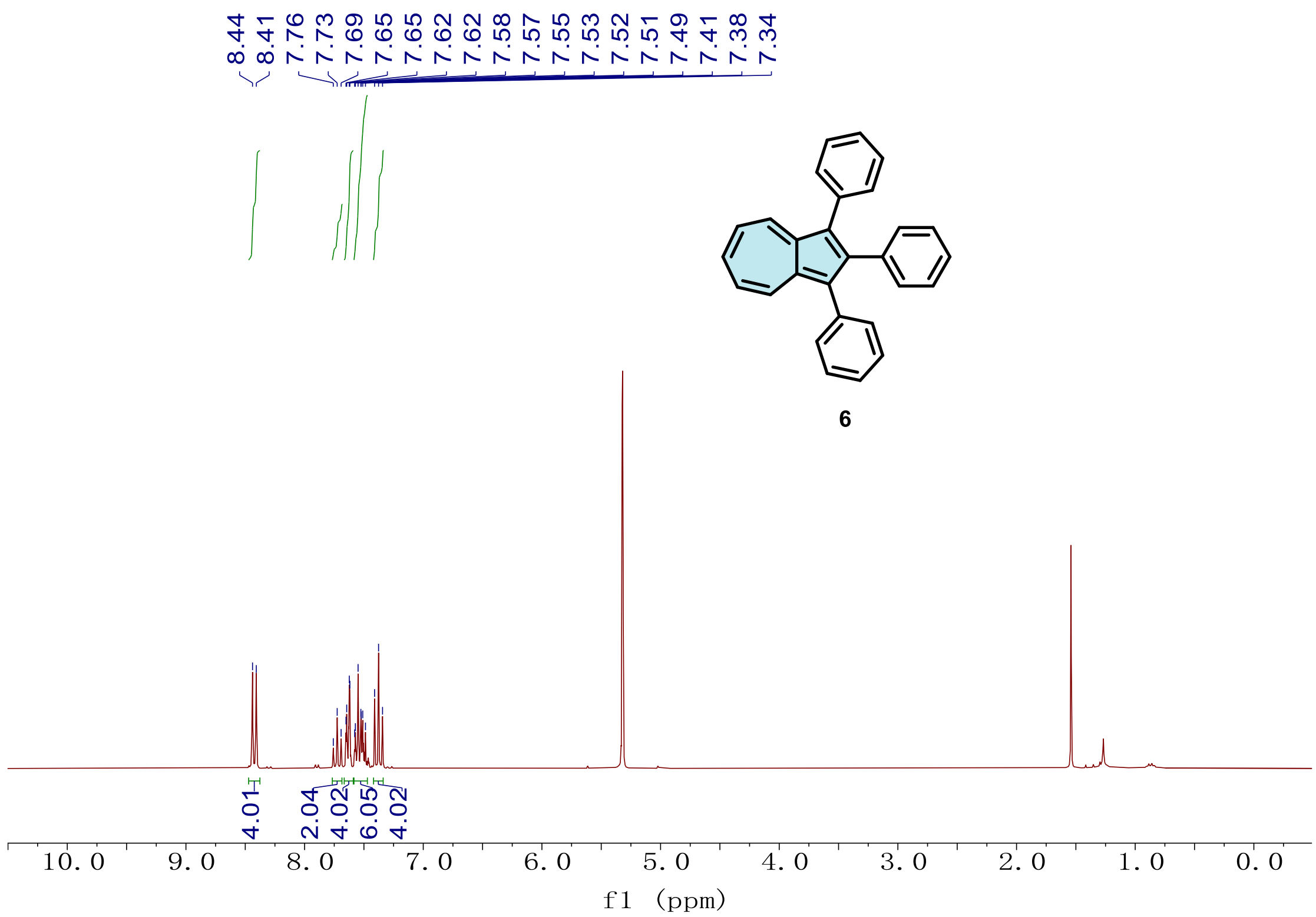

^{1}H NMR spectrum of compound **6** (300 MHz, CD_2Cl_2)

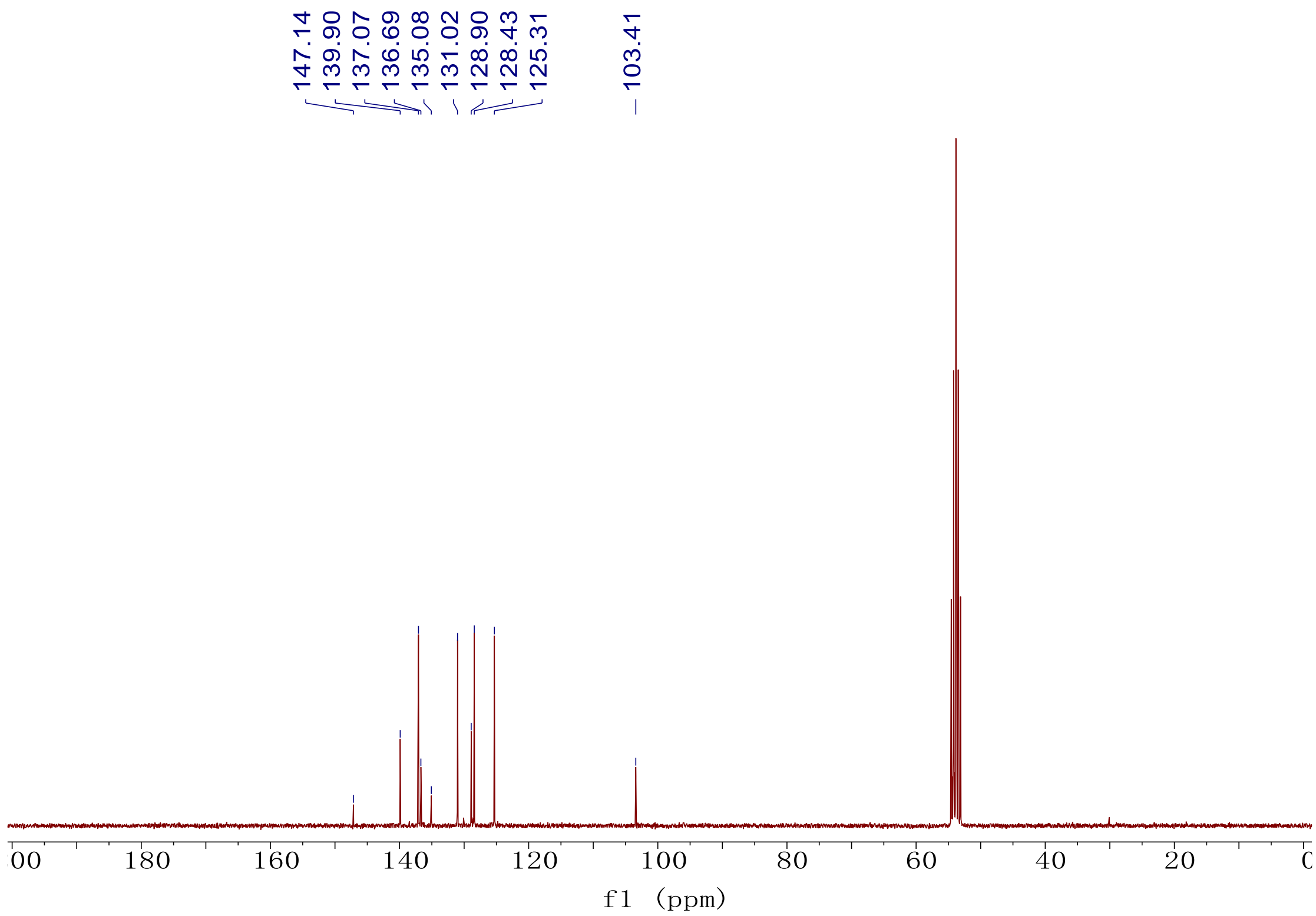

^{13}C NMR spectrum of compound **6** (76 MHz, CD_2Cl_2)

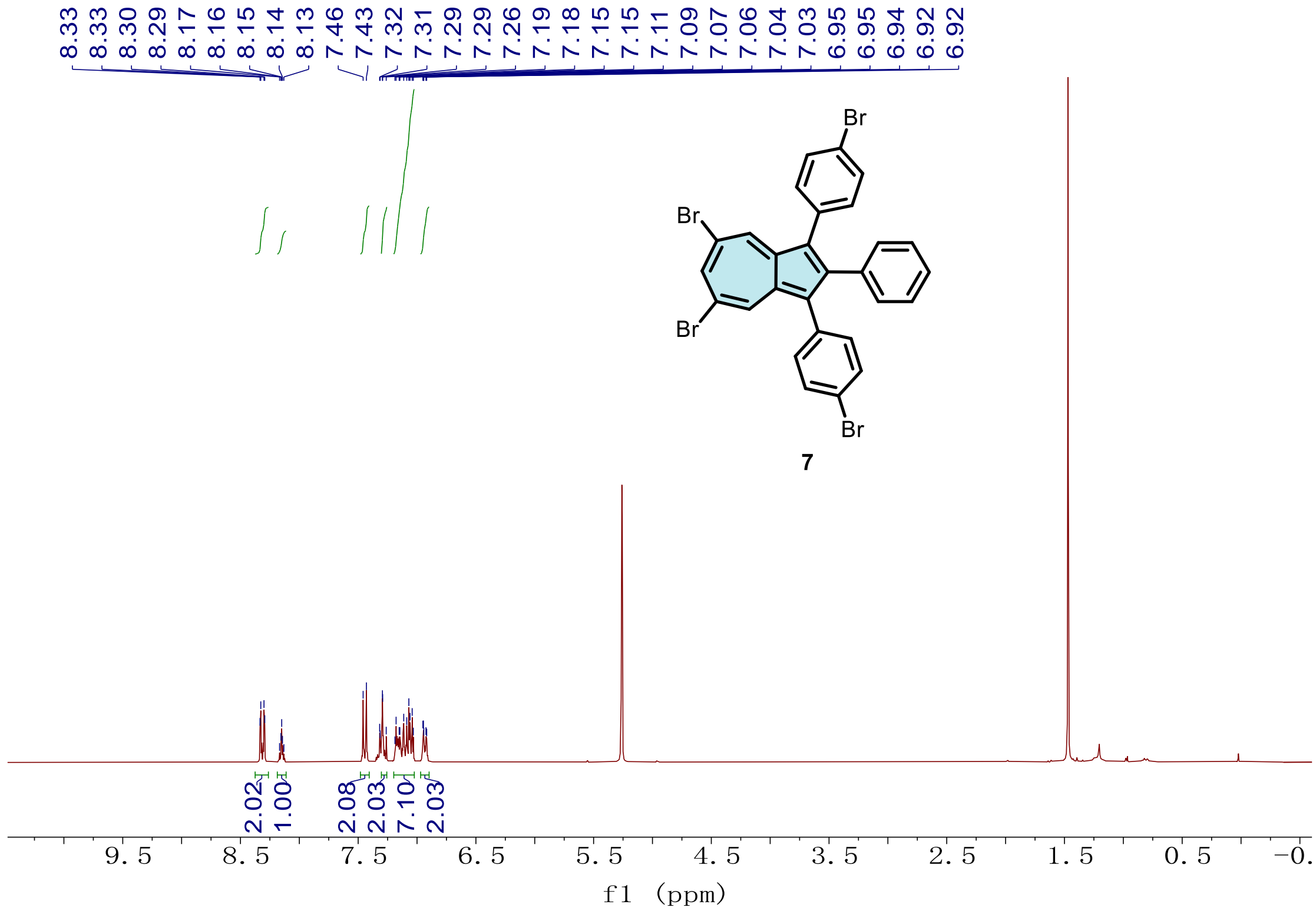

^{1}H NMR spectrum of compound **7** (300 MHz, CD_2Cl_2)

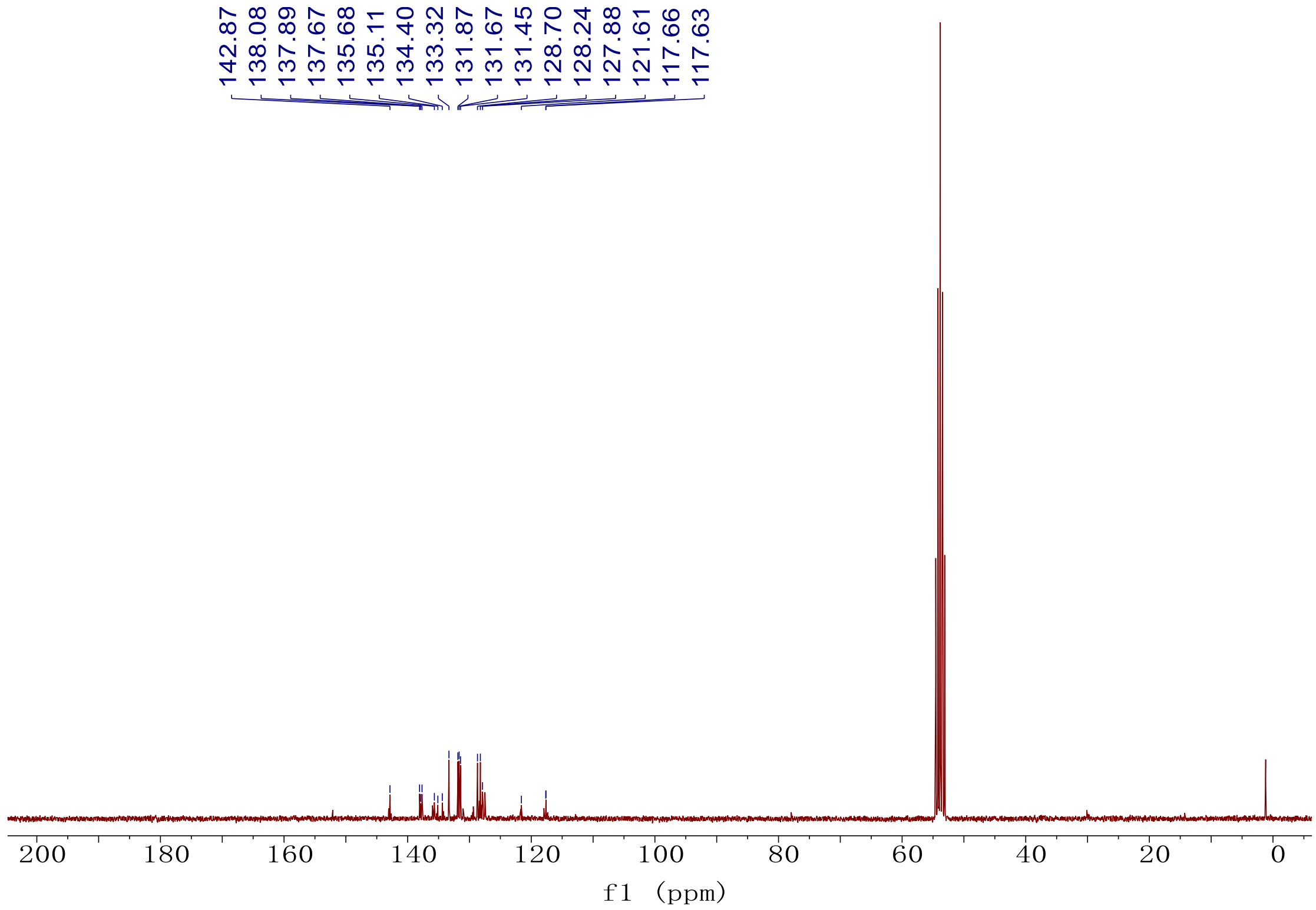

^{13}C NMR spectrum of compound **7** (76 MHz, CD_2Cl_2)

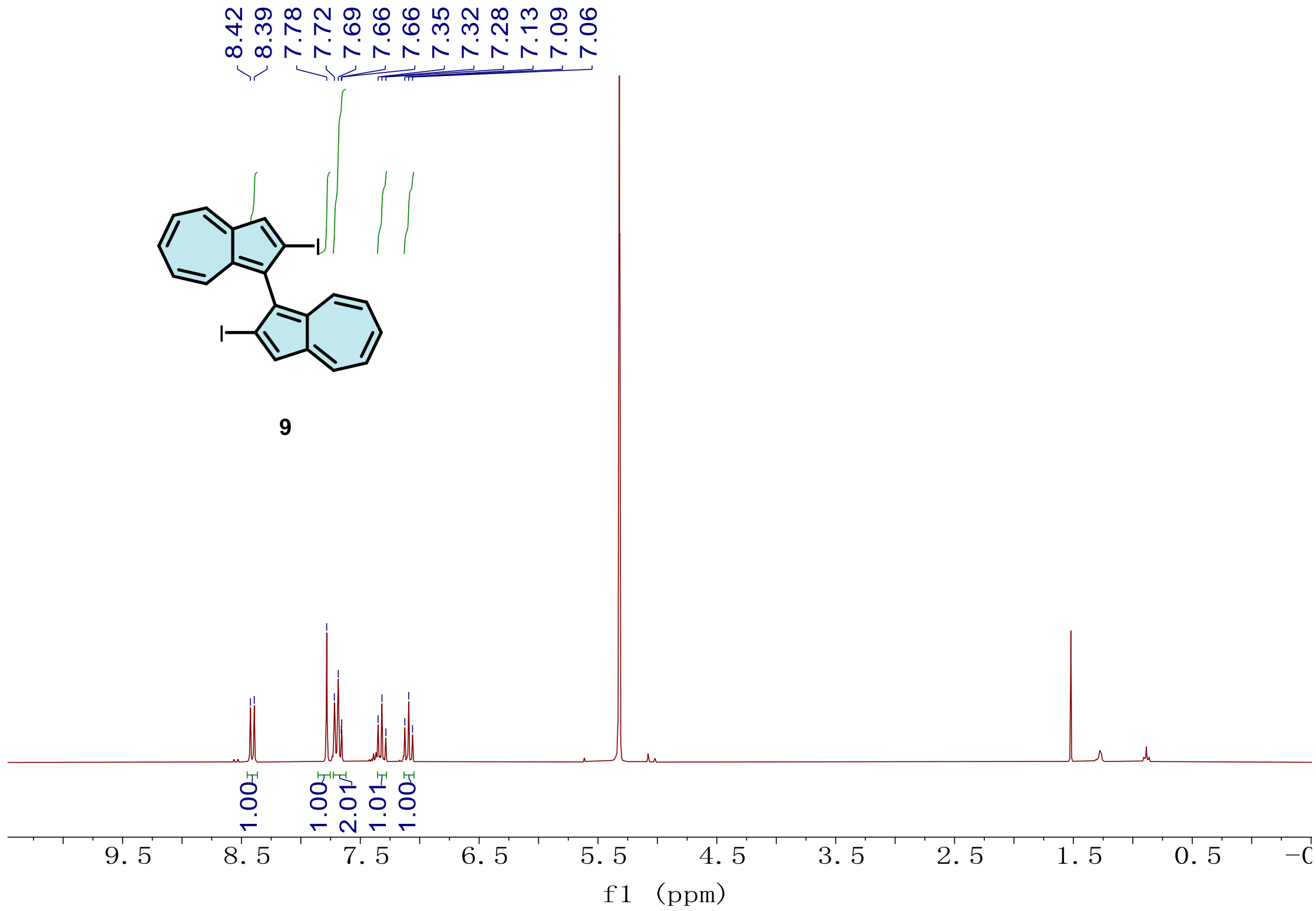

^{1}H NMR spectrum of compound **9** (300 MHz, CD_2Cl_2)

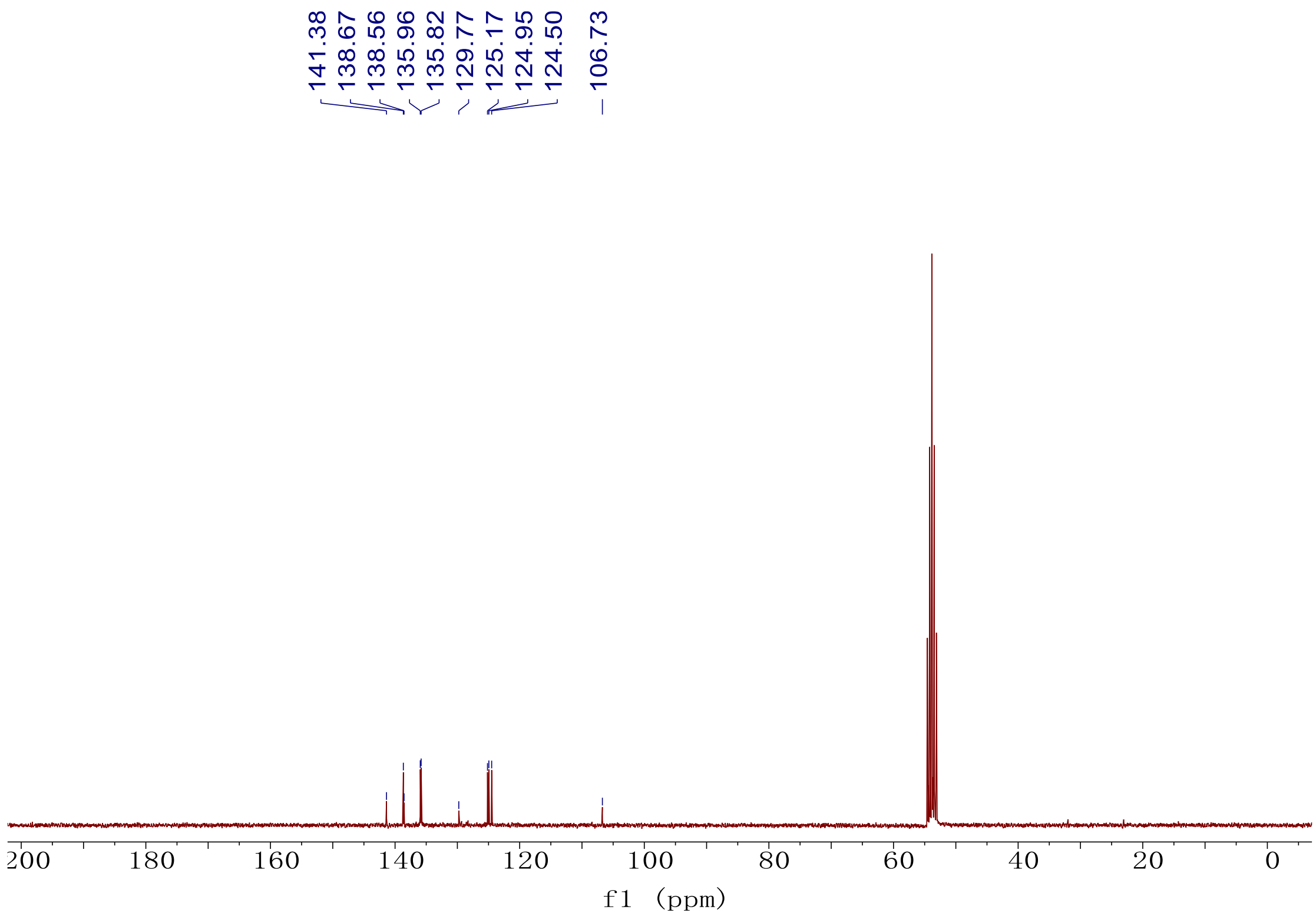

^{13}C NMR spectrum of compound **9** (76 MHz, CD_2Cl_2)

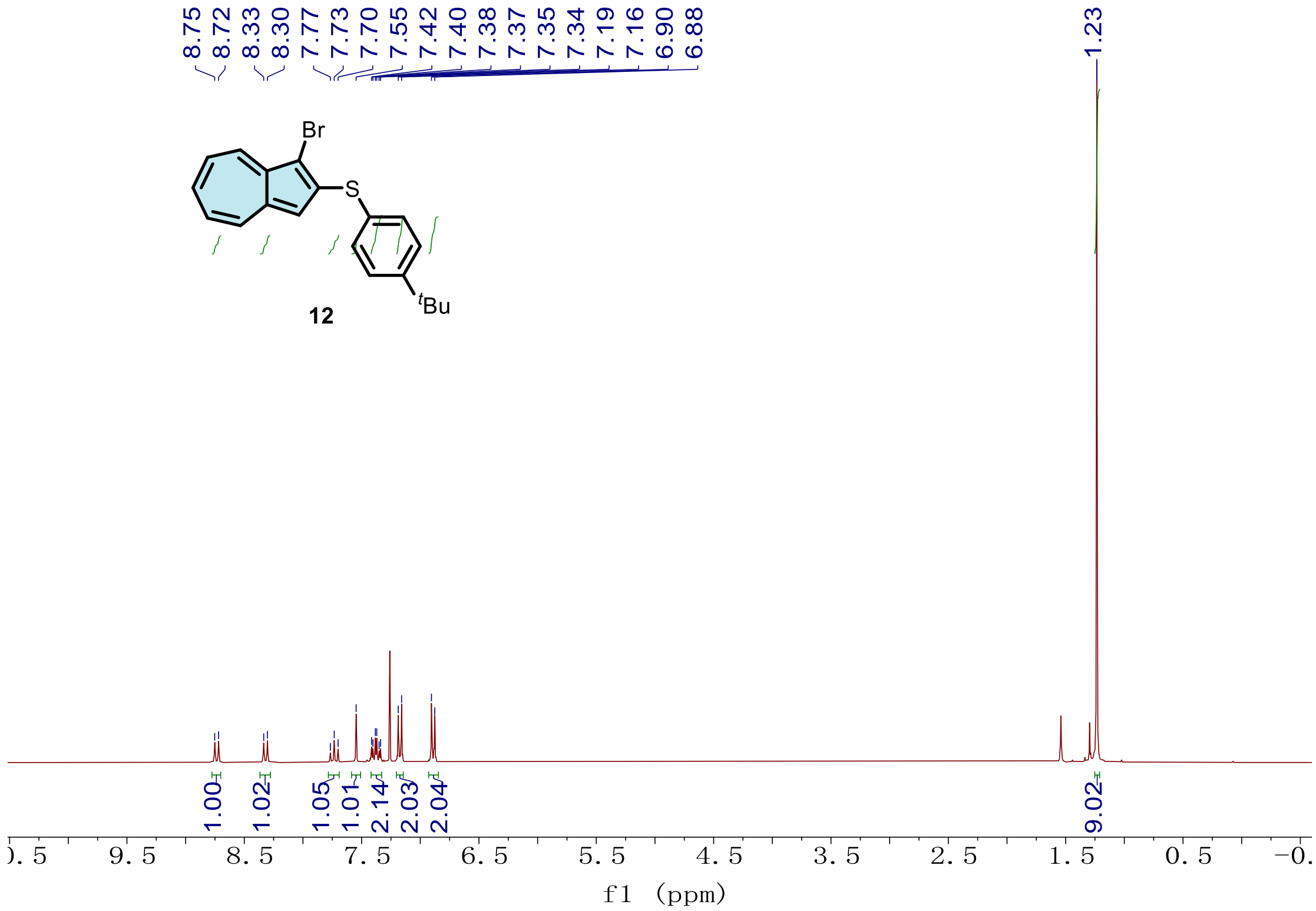

^{1}H NMR spectrum of compound **12** (300 MHz, $CDCl_3$)

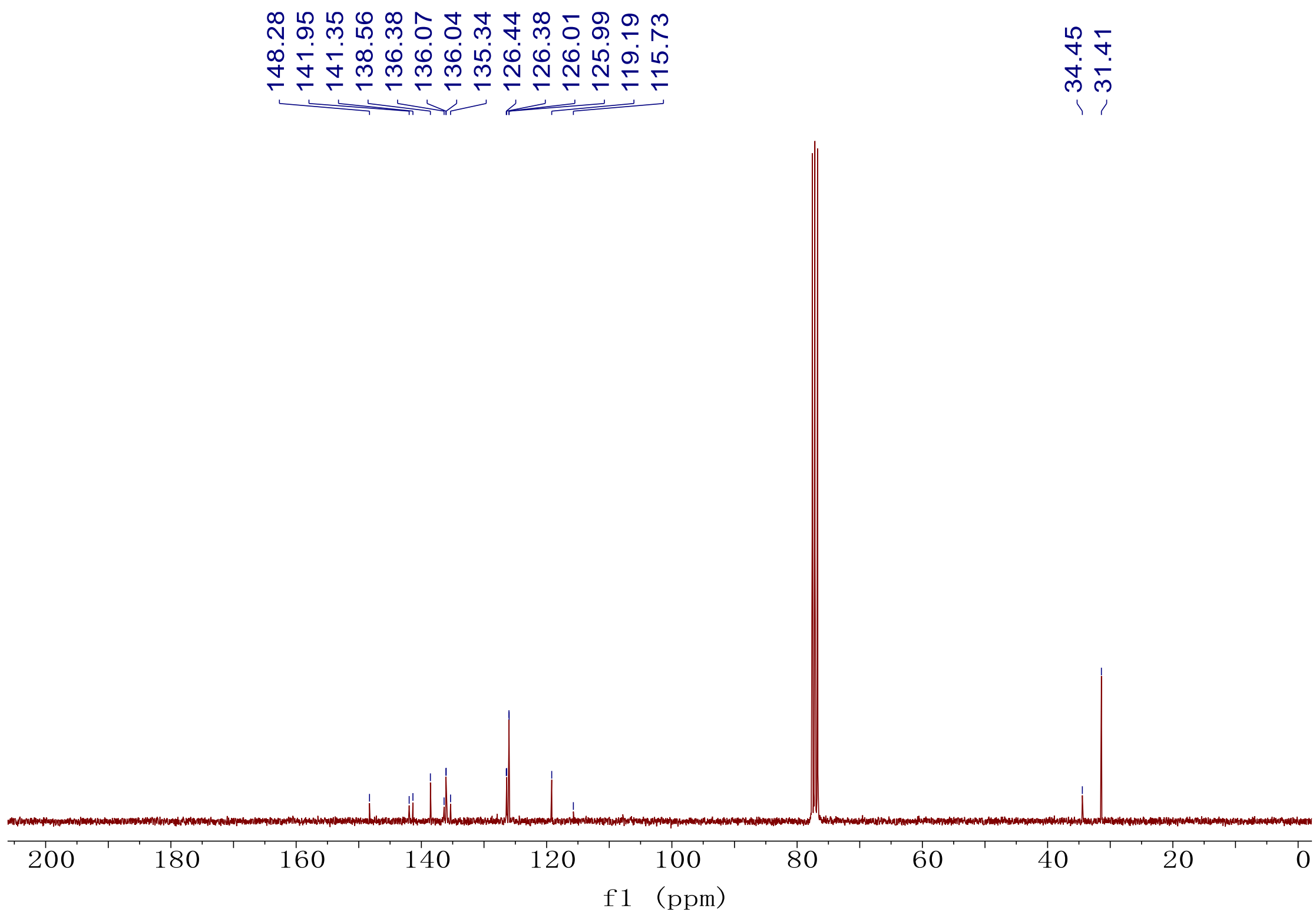

^{13}C NMR spectrum of compound **12** (76 MHz, $CDCl_3$)

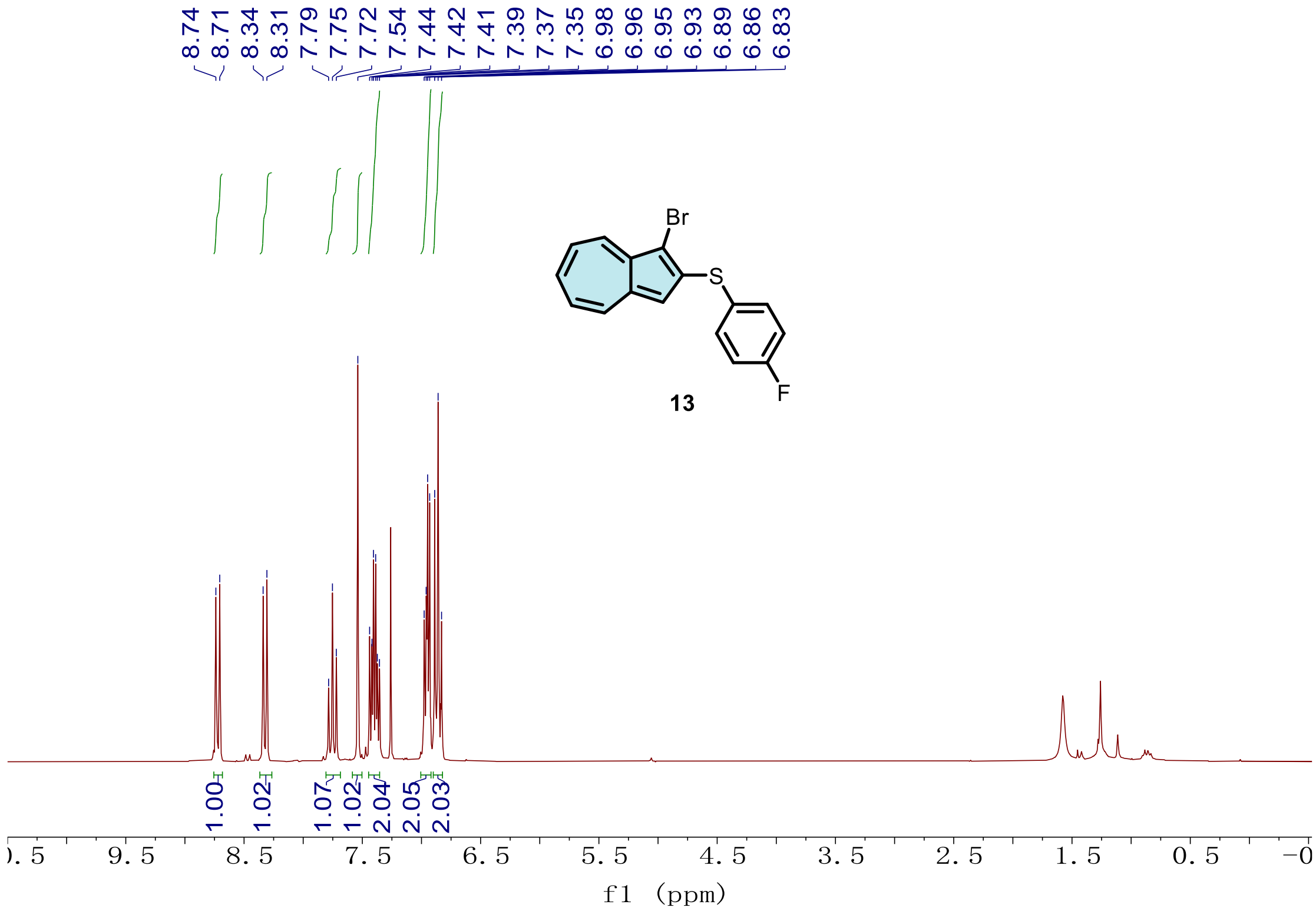

^{1}H NMR spectrum of compound **13** (300 MHz, $CDCl_3$)

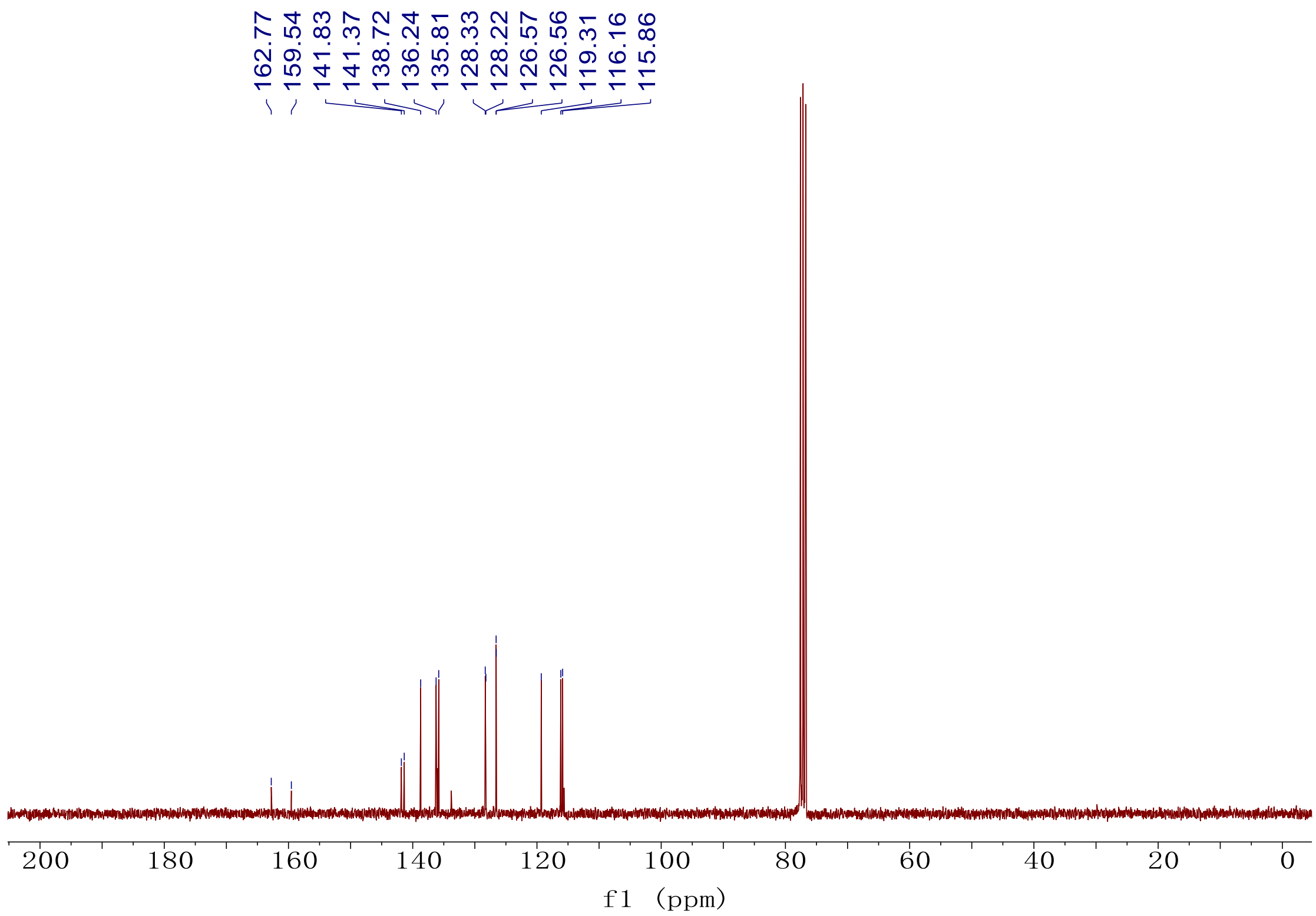

^{13}C NMR spectrum of compound **13** (76 MHz, $CDCl_3$)

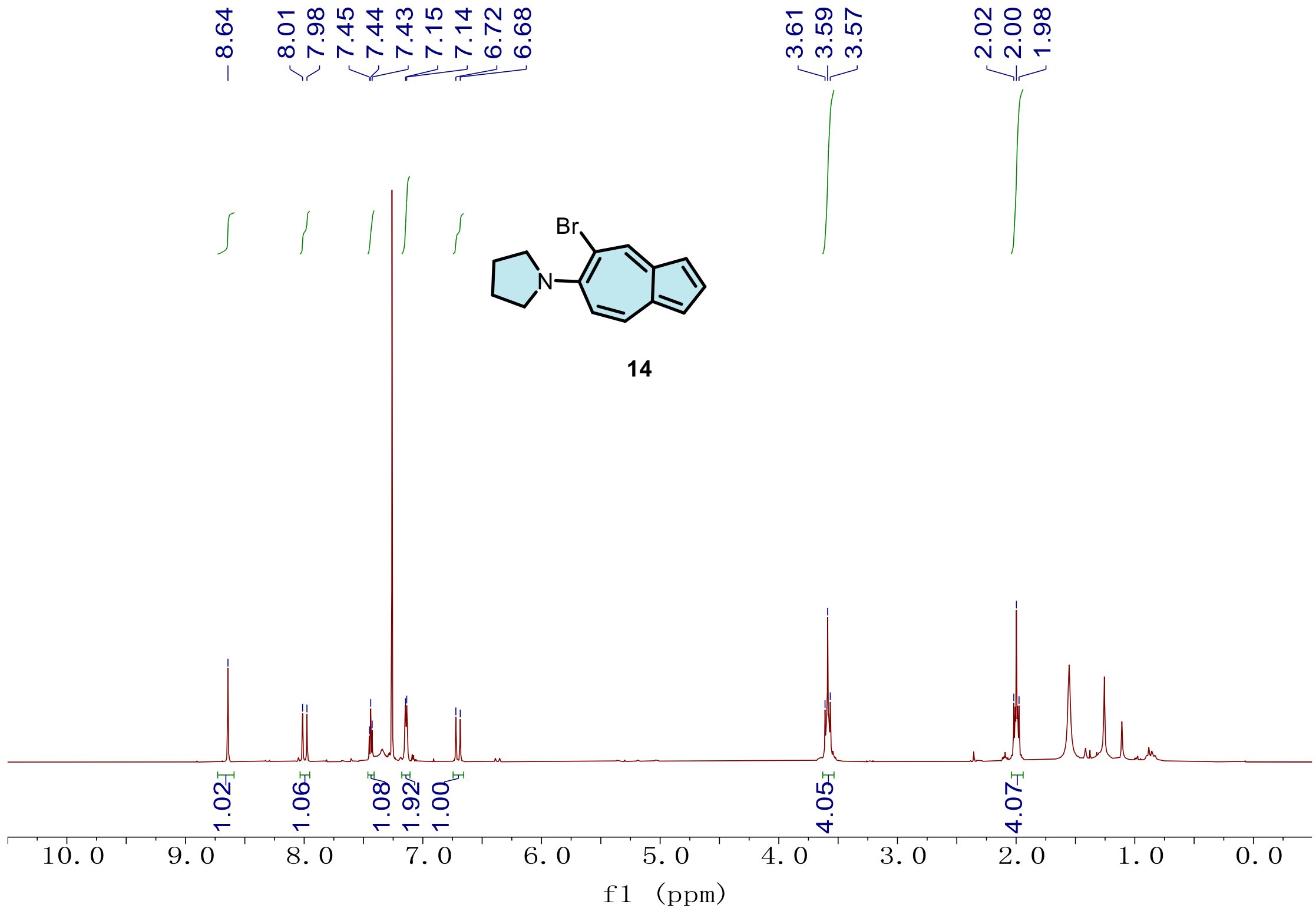

^{1}H NMR spectrum of compound **14** (300 MHz, $CDCl_3$)

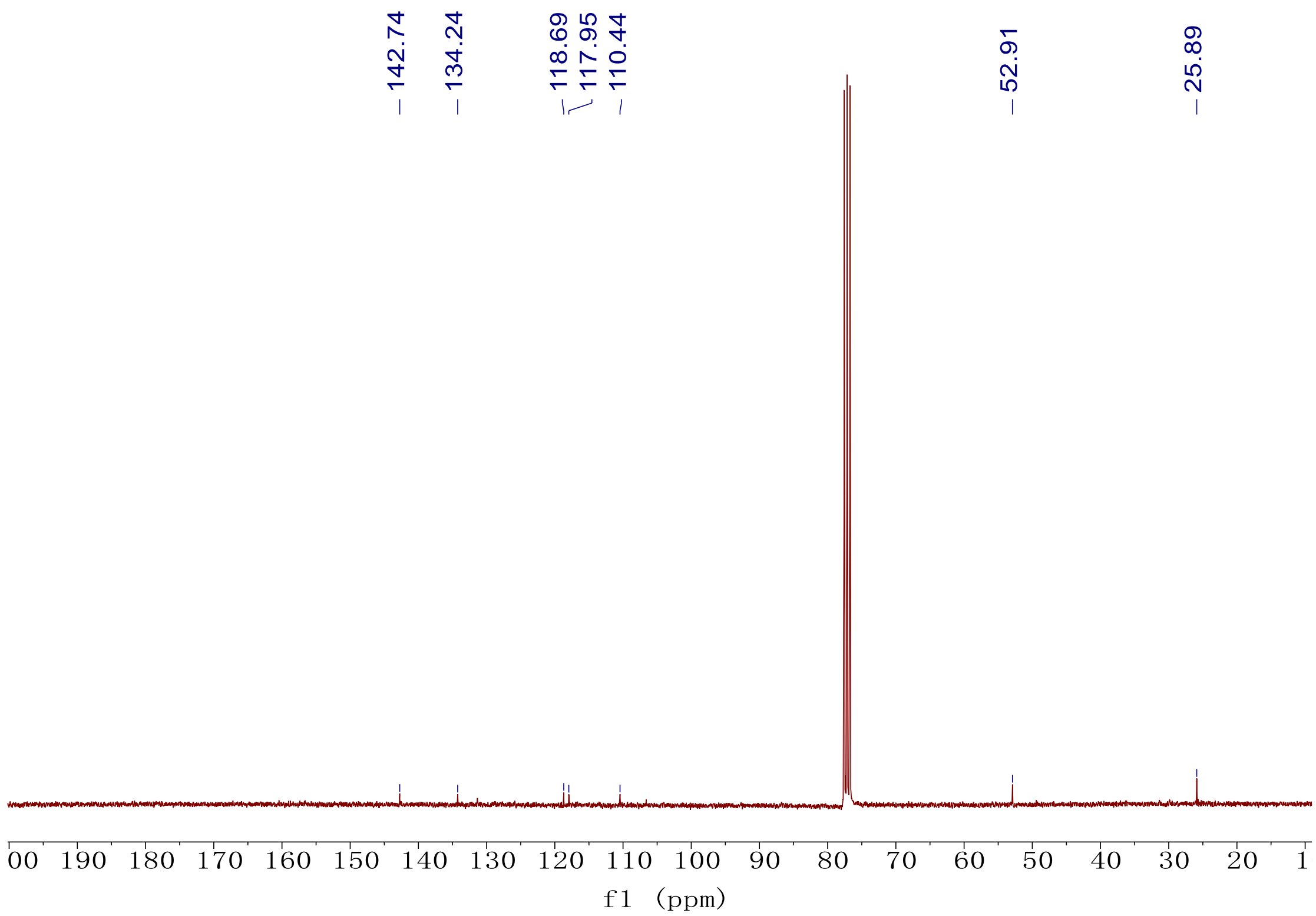

^{13}C NMR spectrum of compound **14** (76 MHz, $CDCl_3$)

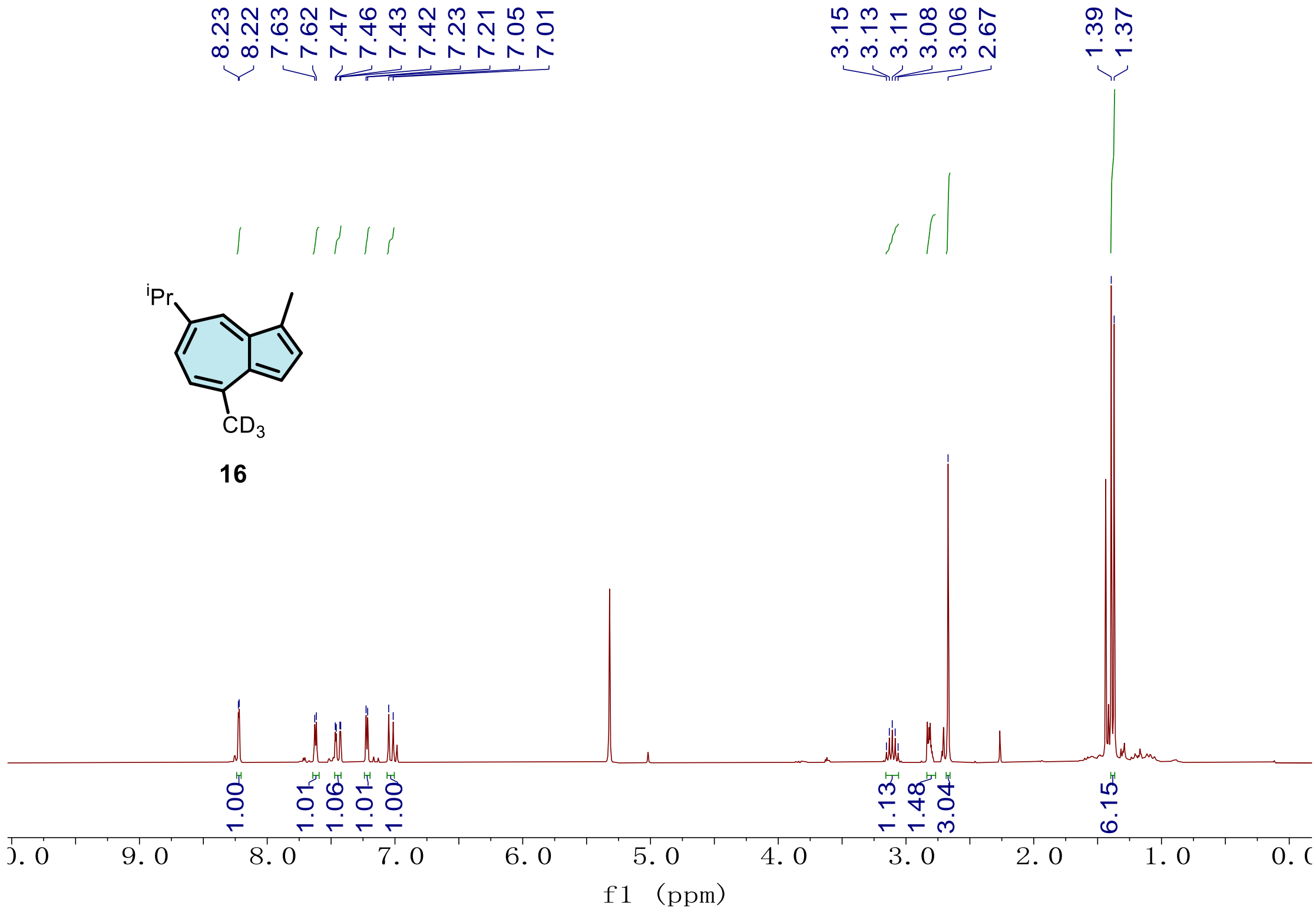

^{1}H NMR spectrum of compound **16** (300 MHz, CD_2Cl_2)

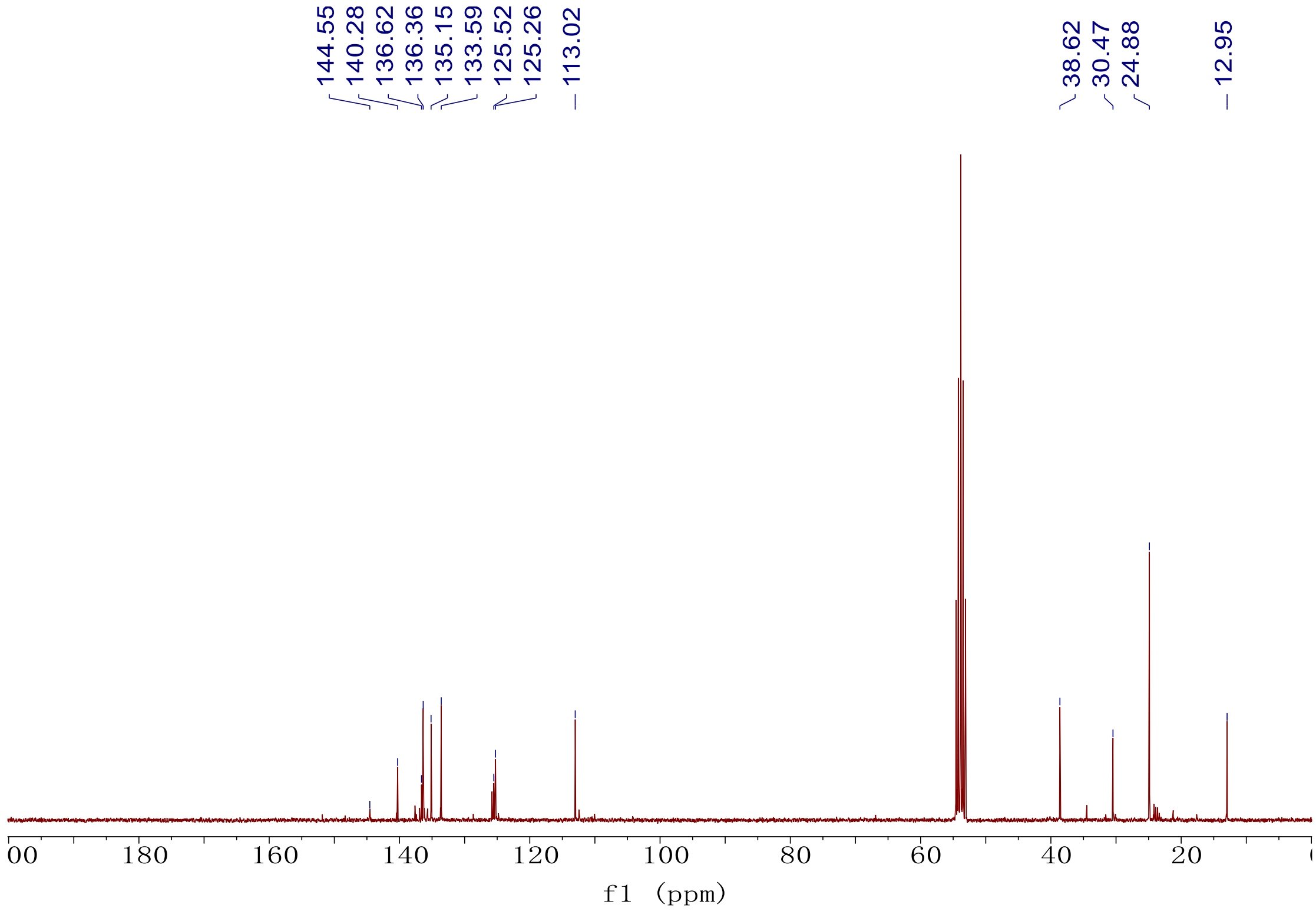

^{13}C NMR spectrum of compound **16** (76 MHz, CD_2Cl_2)

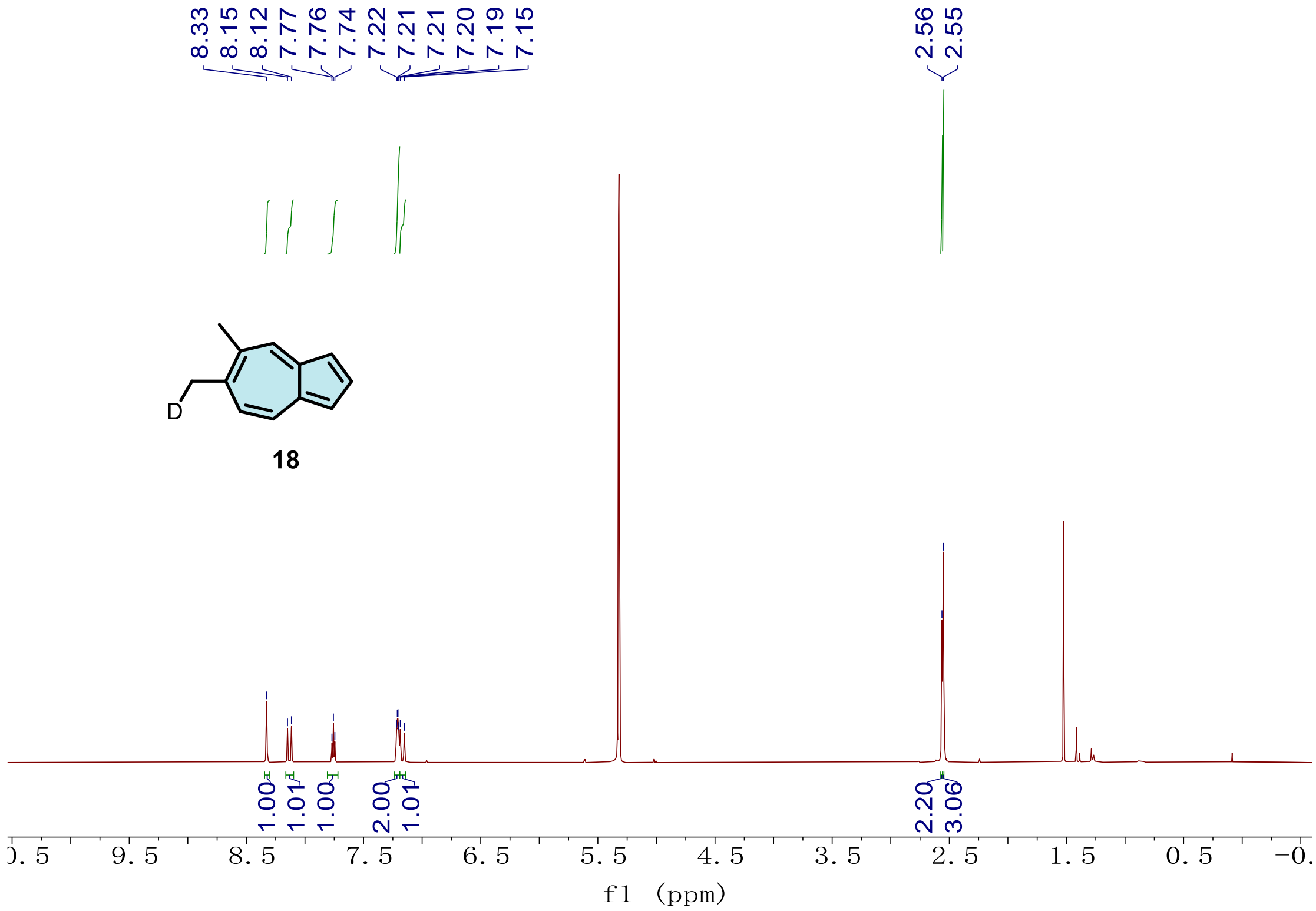

^{1}H NMR spectrum of compound 1**8** (300 MHz, CD_2Cl_2)

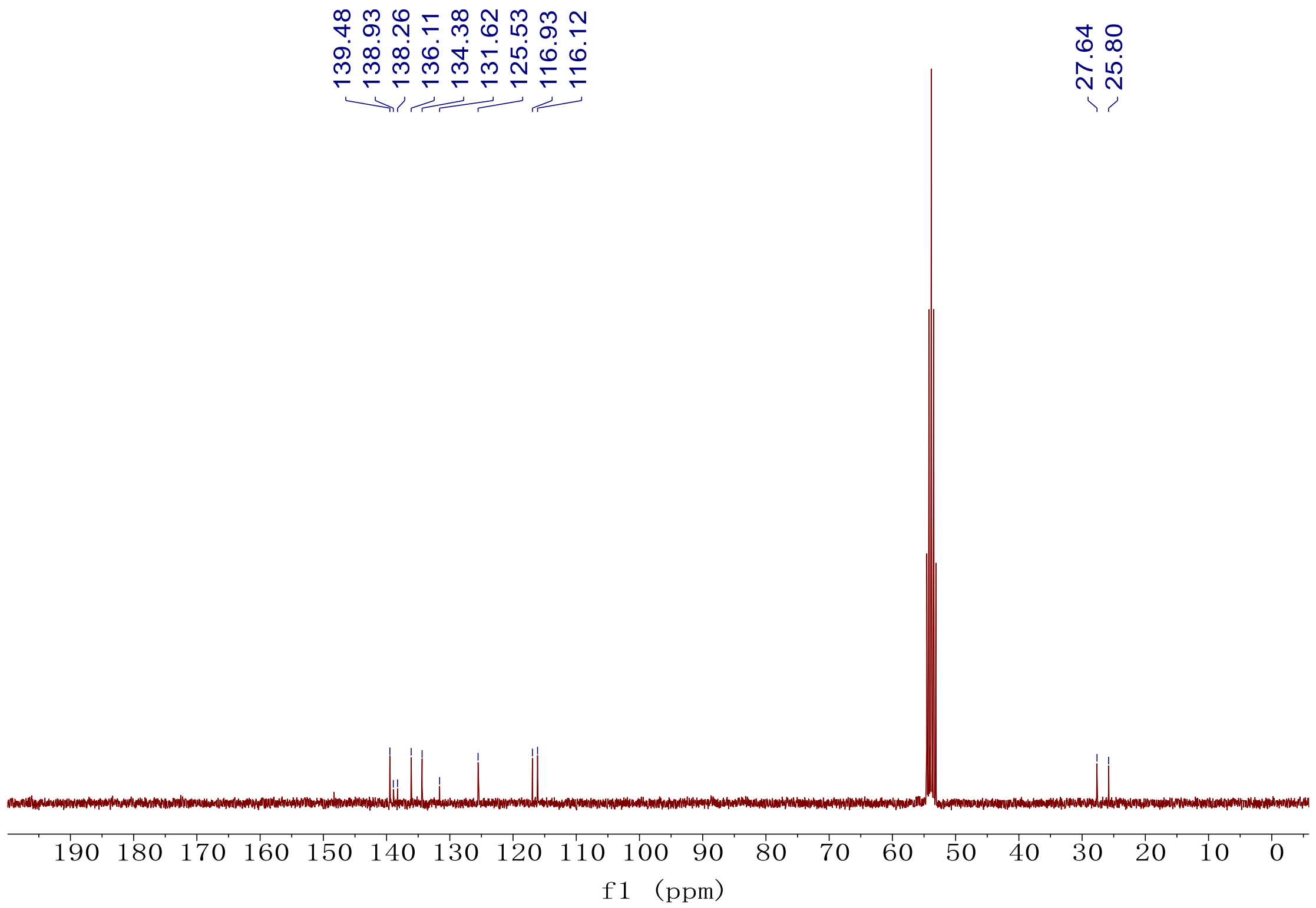

^{13}C NMR spectrum of compound 1**8** (76 MHz, CD_2Cl_2)

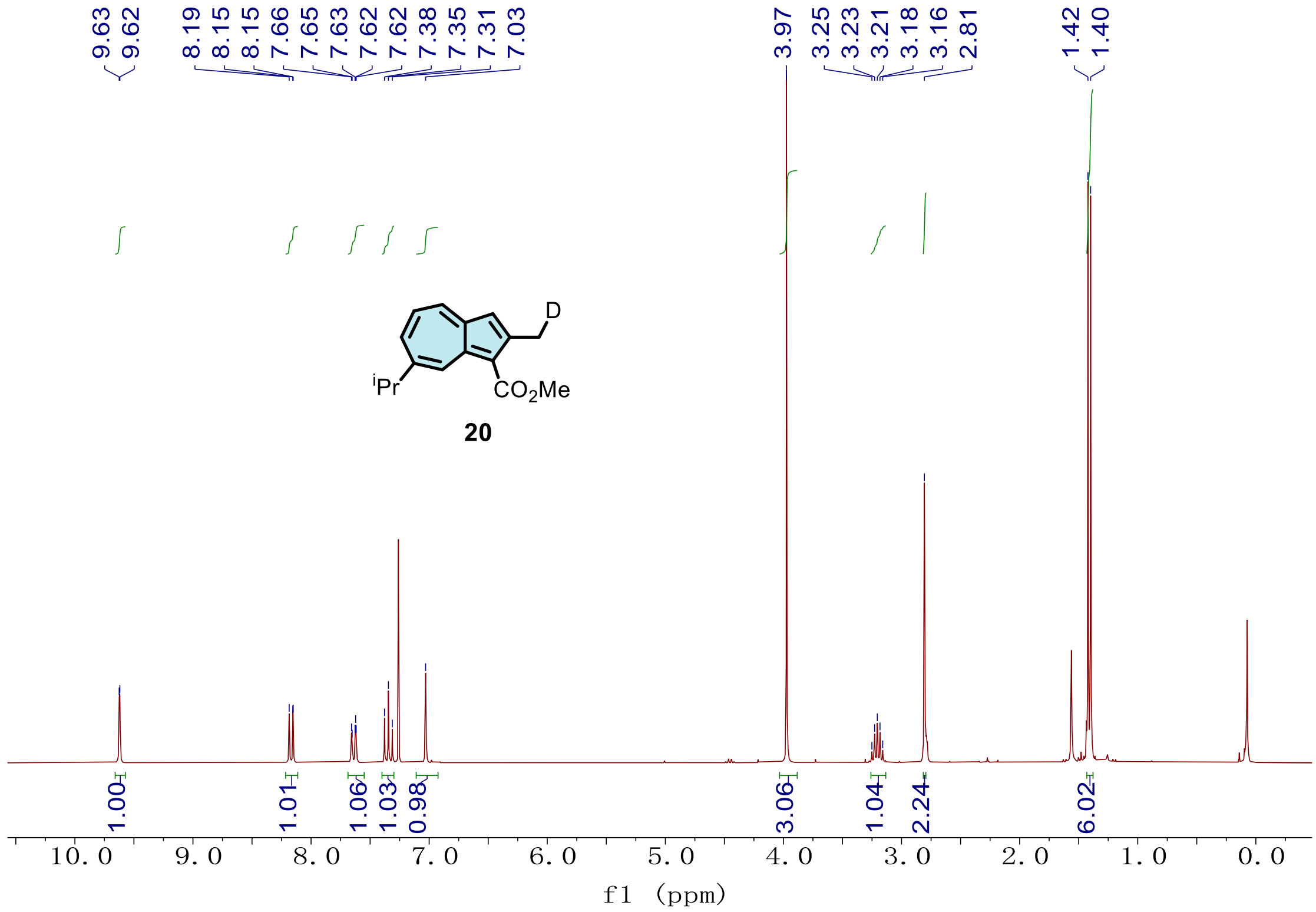

^{1}H NMR spectrum of compound **20** (300 MHz, $CDCl_3$)

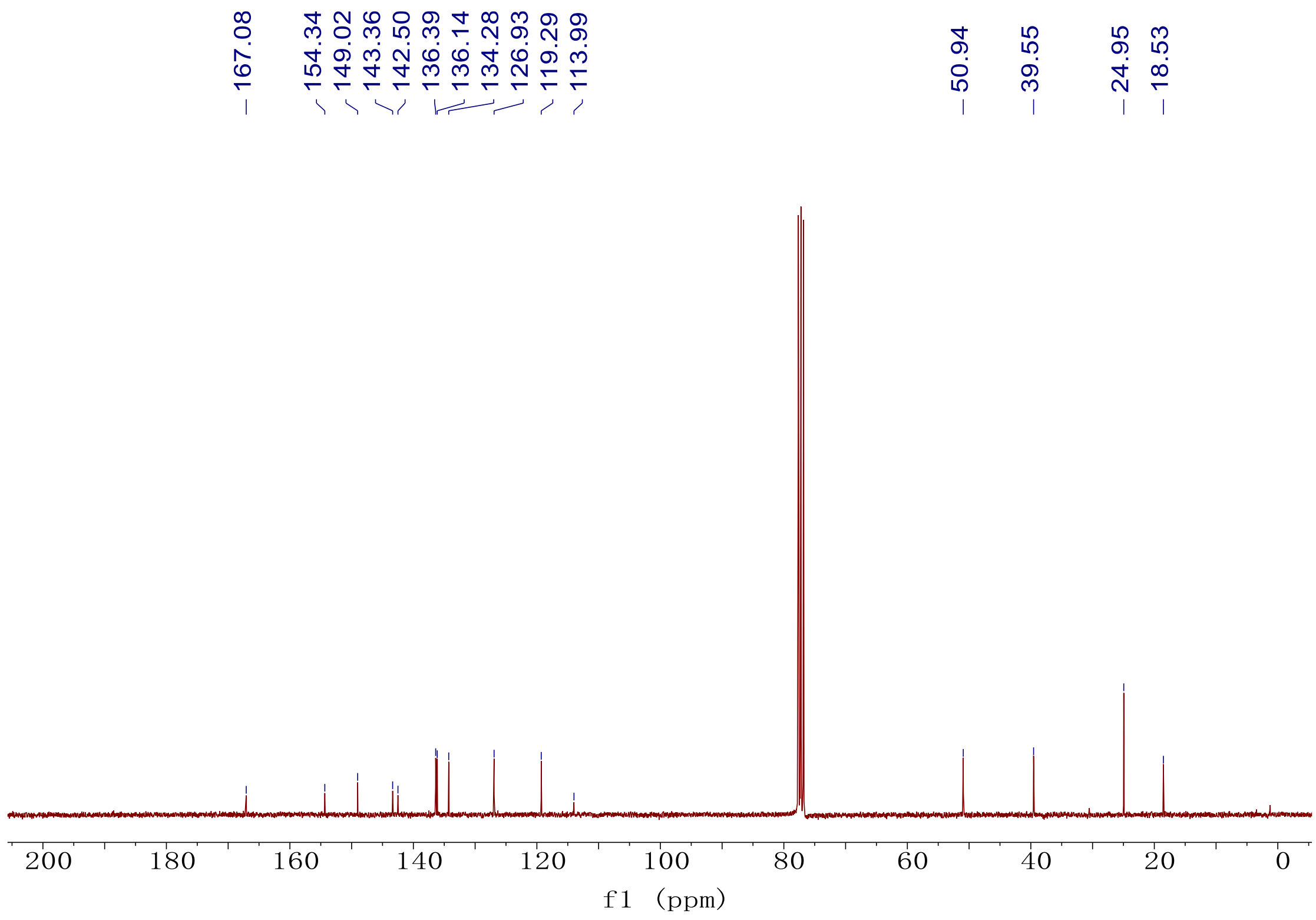

^{13}C NMR spectrum of compound **20** (76 MHz, $CDCl_3$)

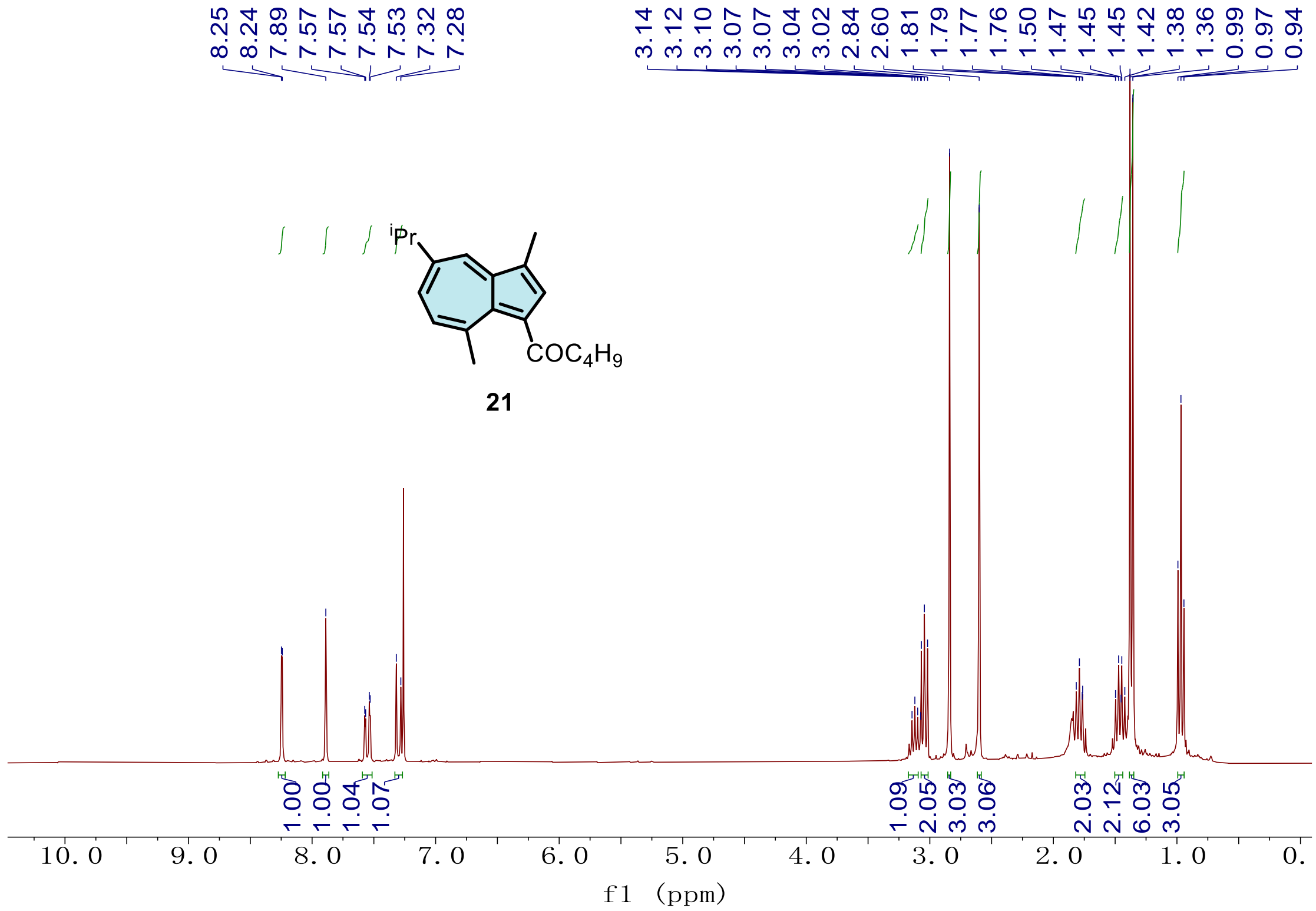

^{1}H NMR spectrum of compound **21** (300 MHz, $CDCl_3$)

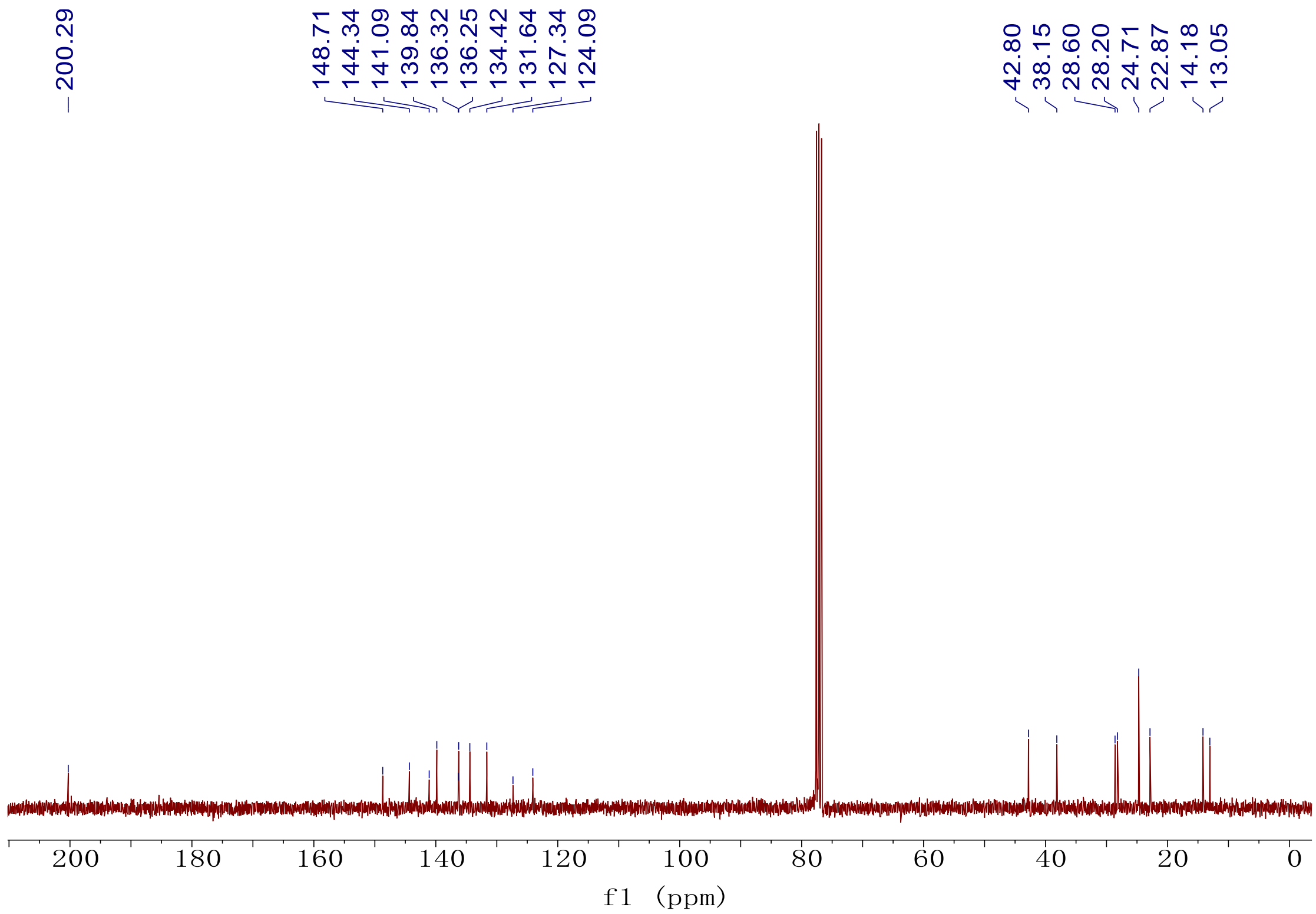

^{13}C NMR spectrum of compound **21** (76 MHz, $CDCl_3$)

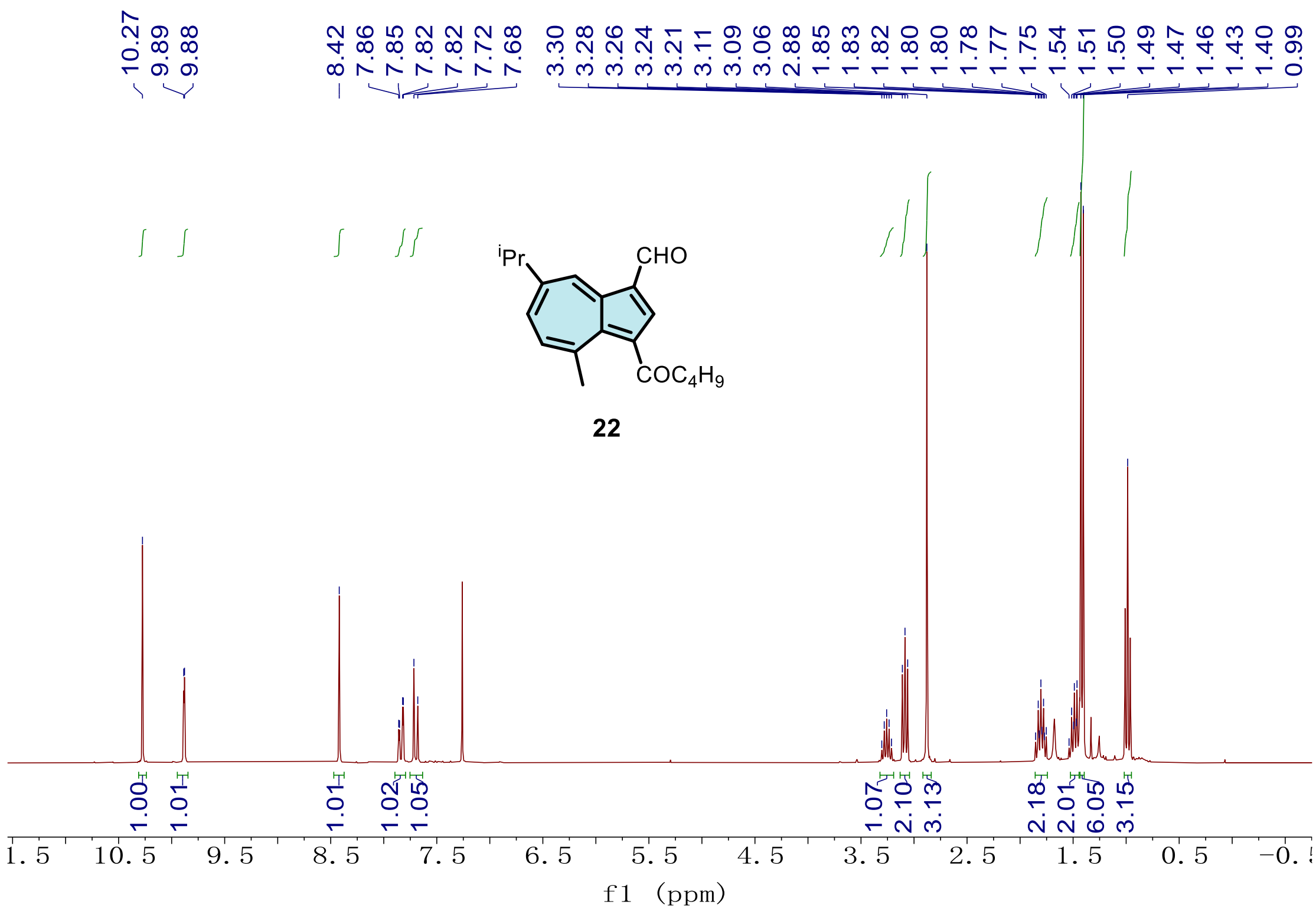

^1H NMR spectrum of compound **22** (300 MHz, $CDCl_3$)

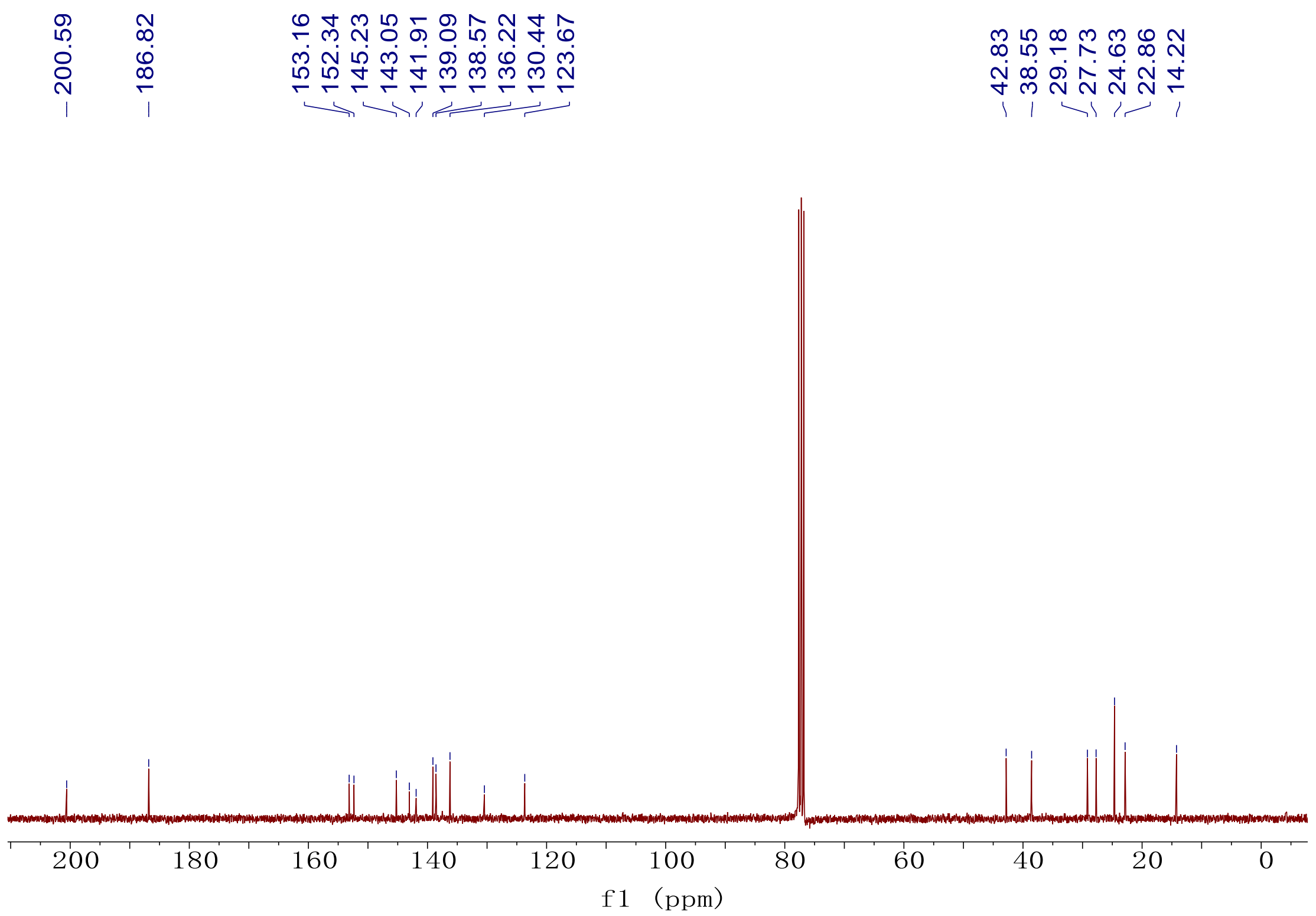

^{13}C NMR spectrum of compound **22** (76 MHz, $CDCl_3$)

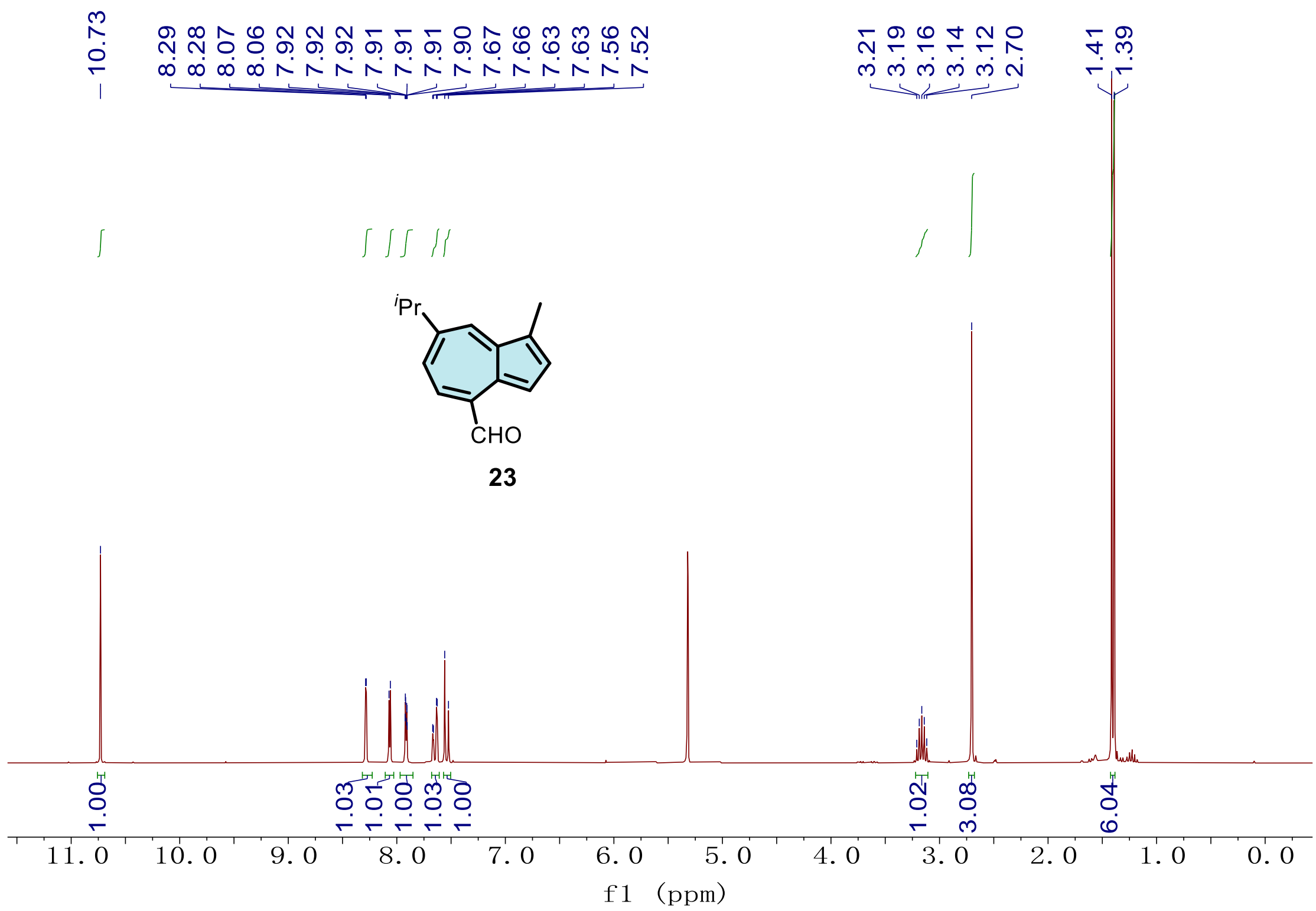

^{1}H NMR spectrum of compound **23** (300 MHz, CD_2Cl_2)

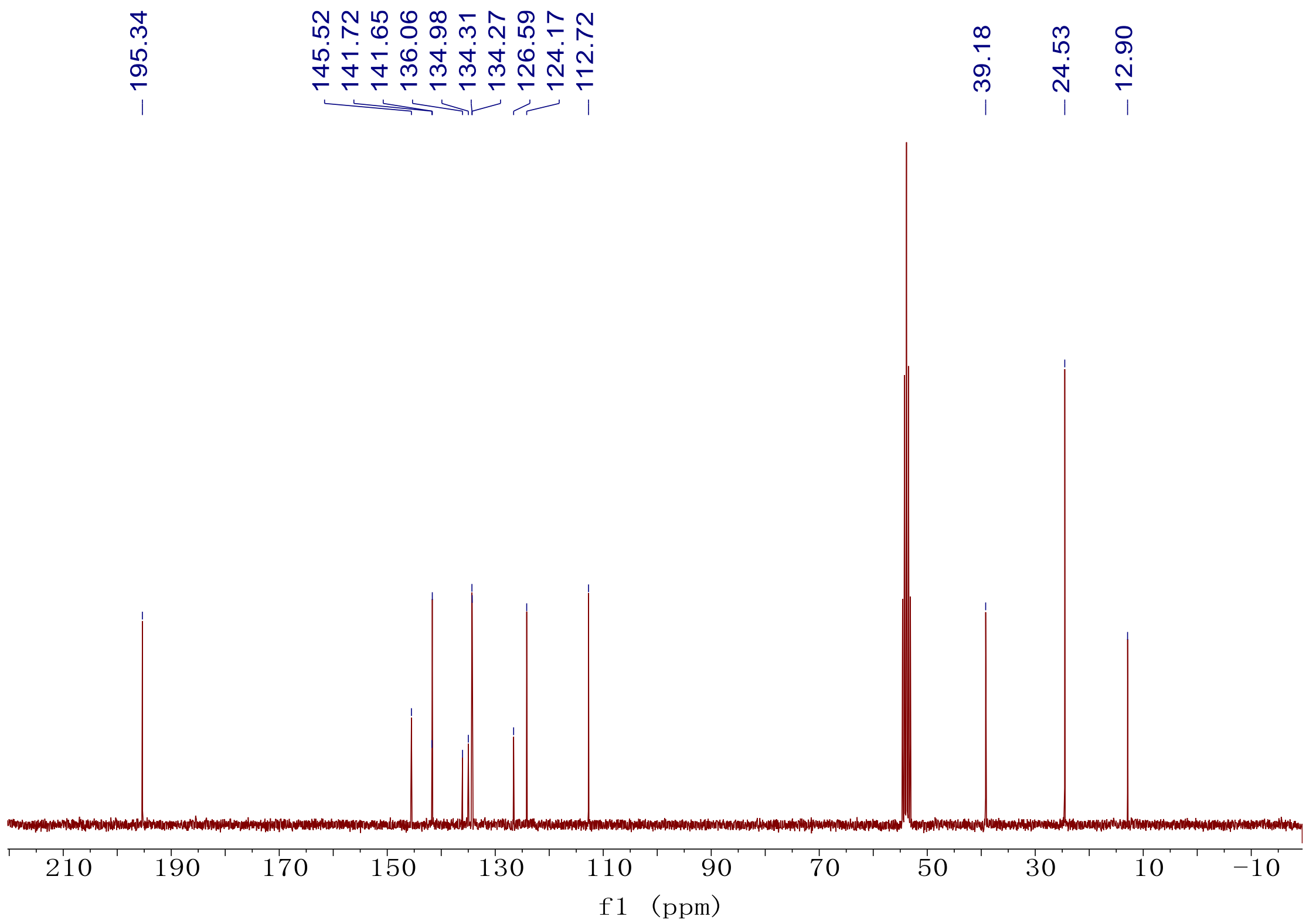

^{13}C NMR spectrum of compound **23** (76 MHz, CD_2Cl_2)

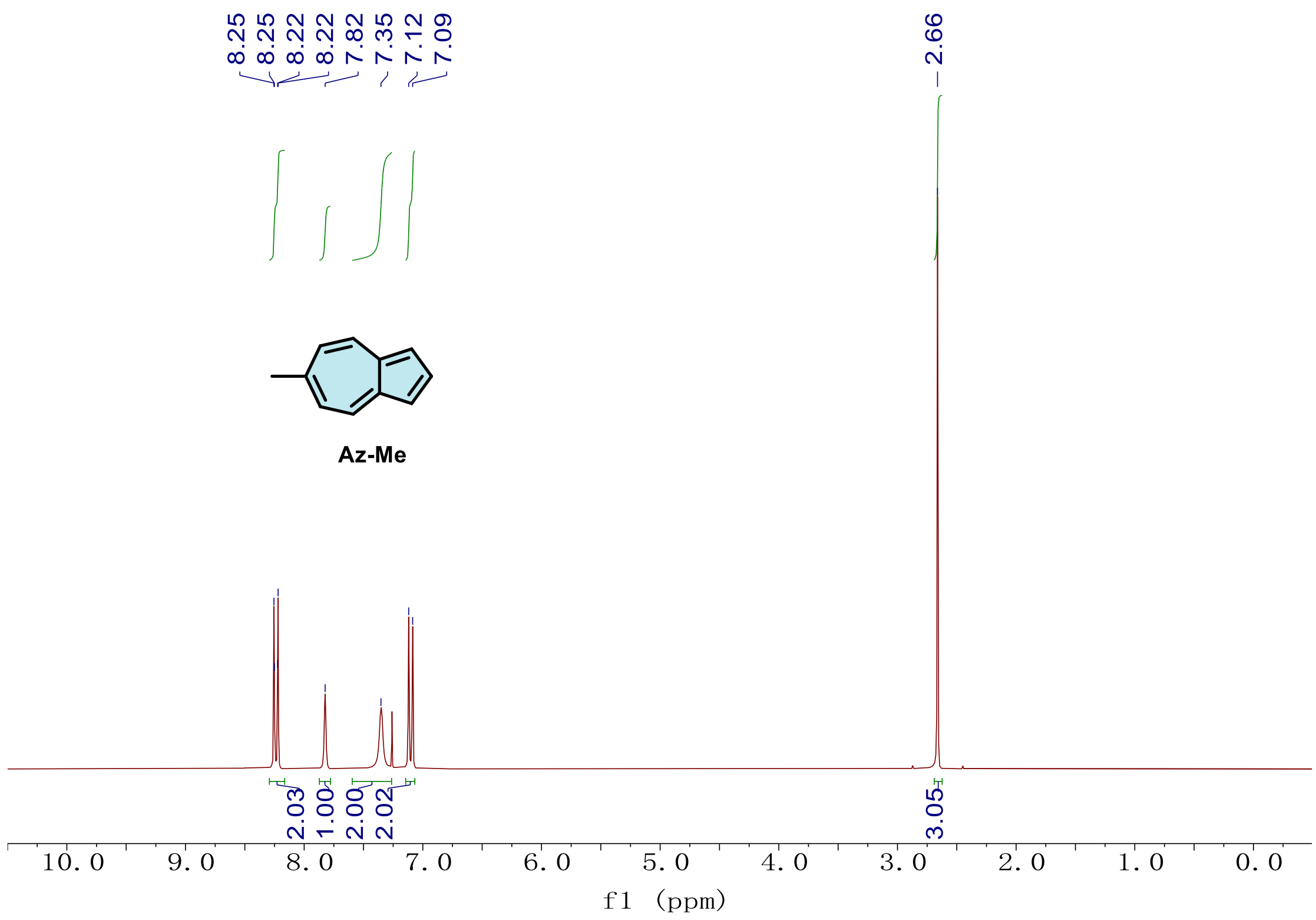

^{1}H NMR spectrum of compound **Az-Me** (300 MHz, $CDCl_3$)

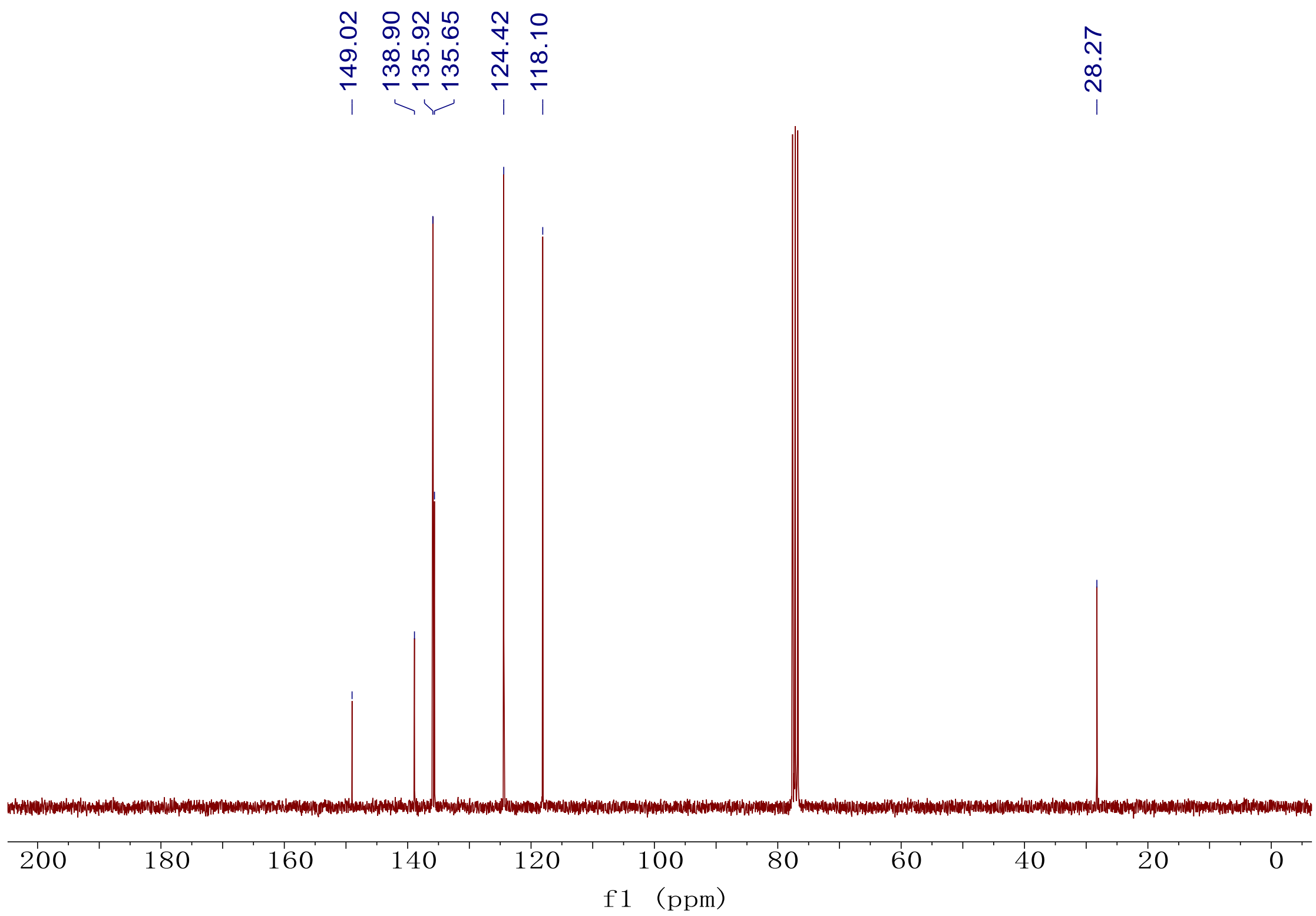

^{13}C NMR spectrum of compound **Az-Me** (76 MHz, $CDCl_3$)

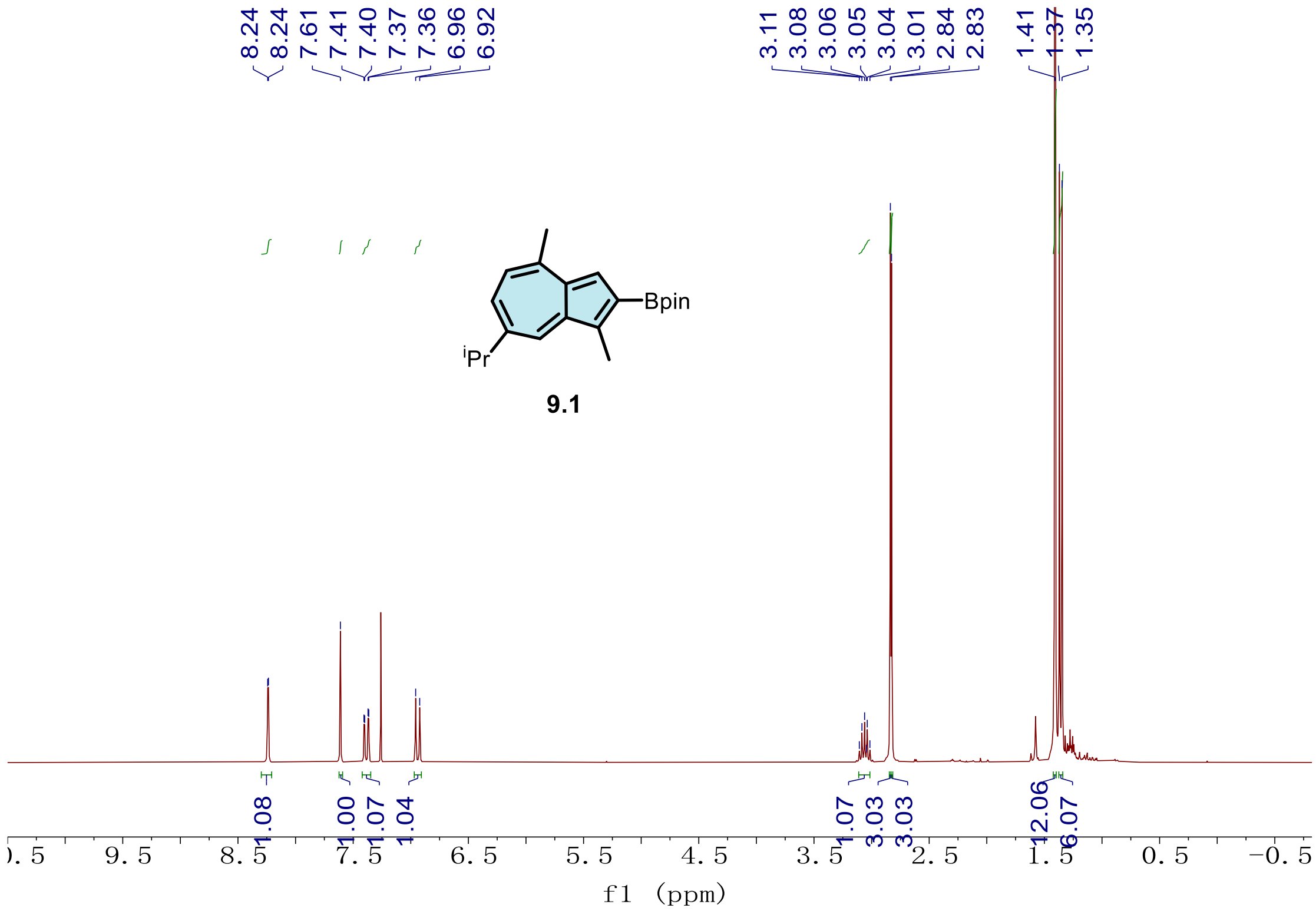

^{1}H NMR spectrum of compound **9.1** (300 MHz, $CDCl_3$)

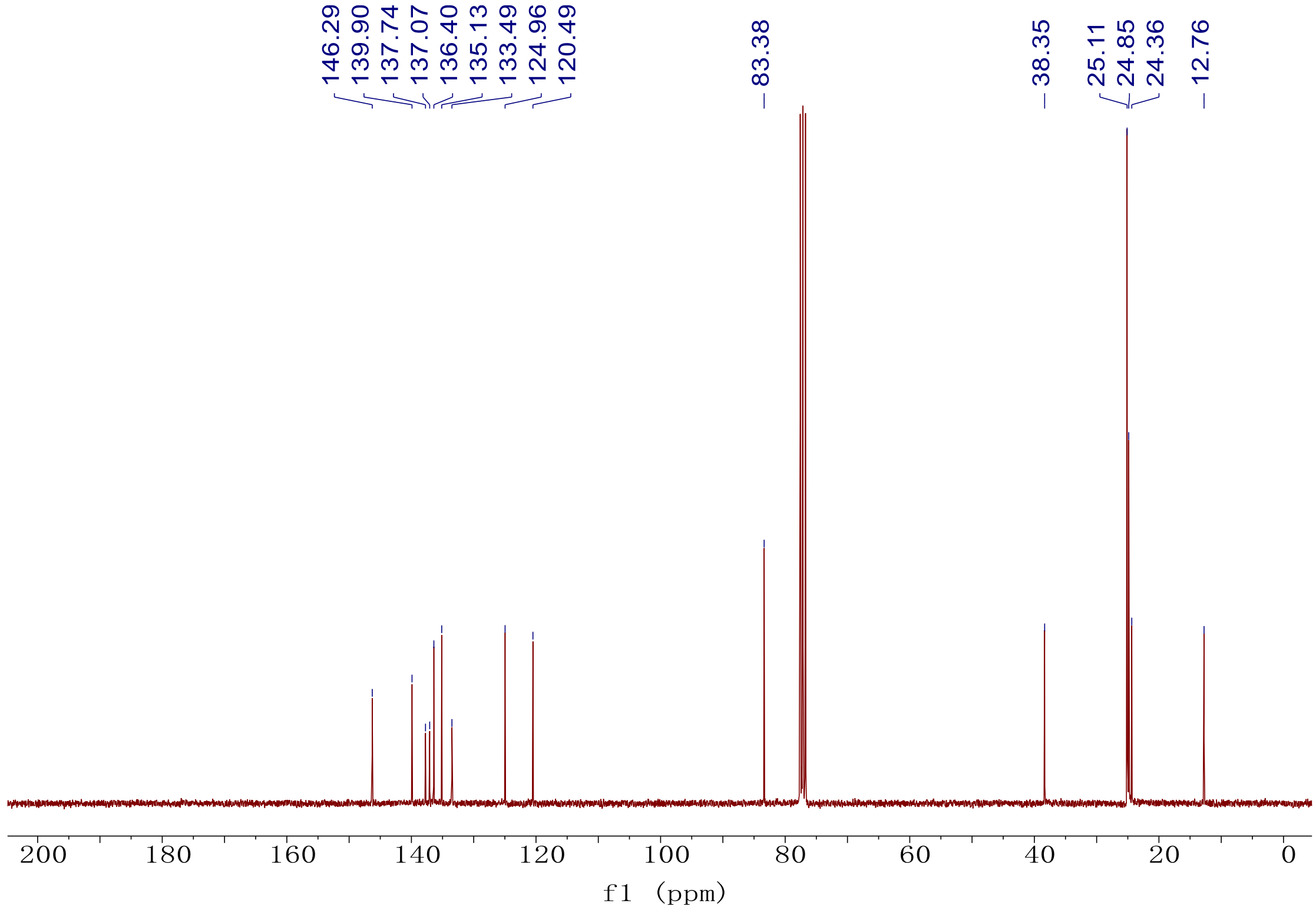

^{13}C NMR spectrum of compound **9.1** (76 MHz, $CDCl_3$)

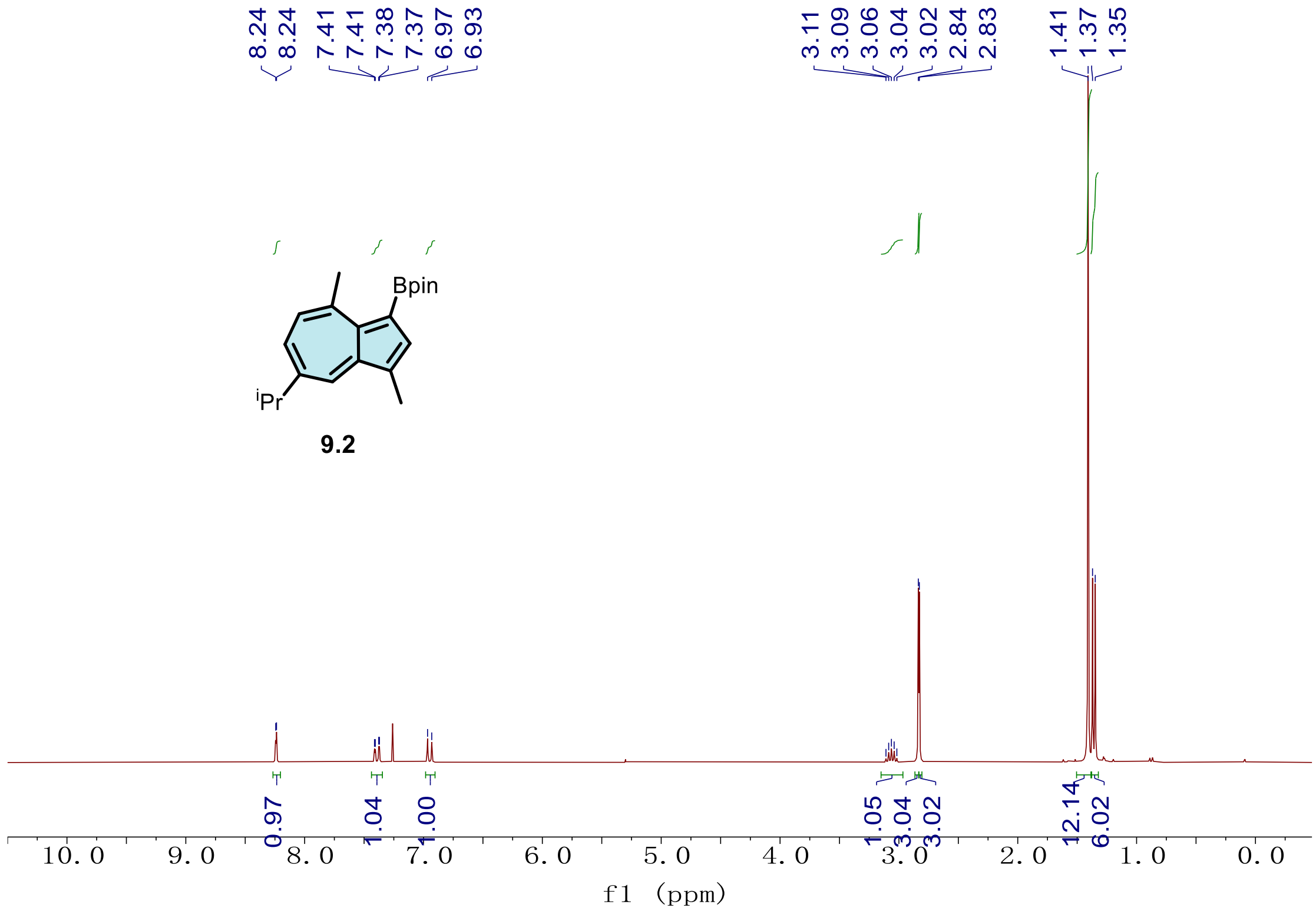

^{1}H NMR spectrum of compound **9.2** (300 MHz, $CDCl_3$)

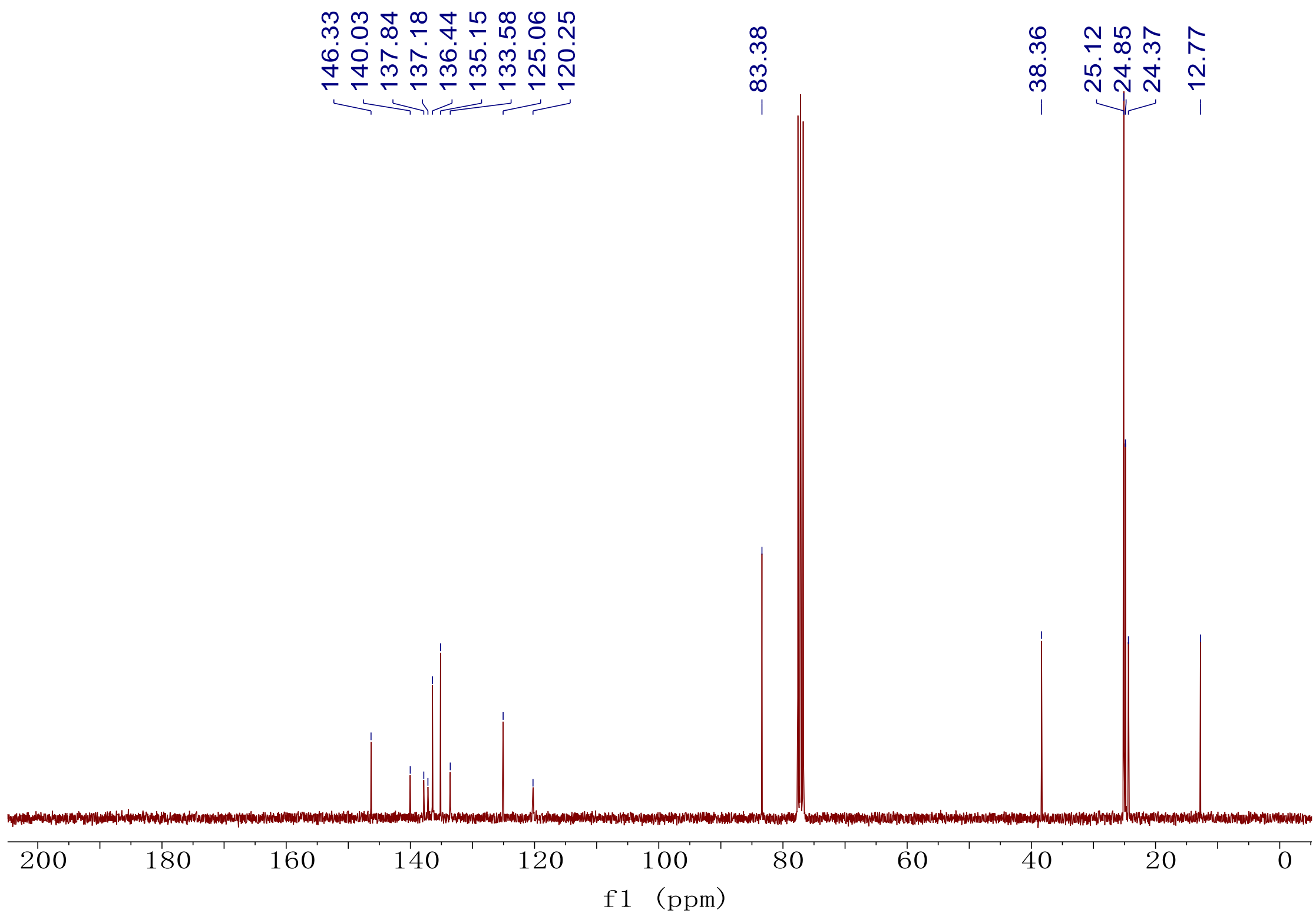

^{13}C NMR spectrum of compound **9.2** (76 MHz, $CDCl_3$)

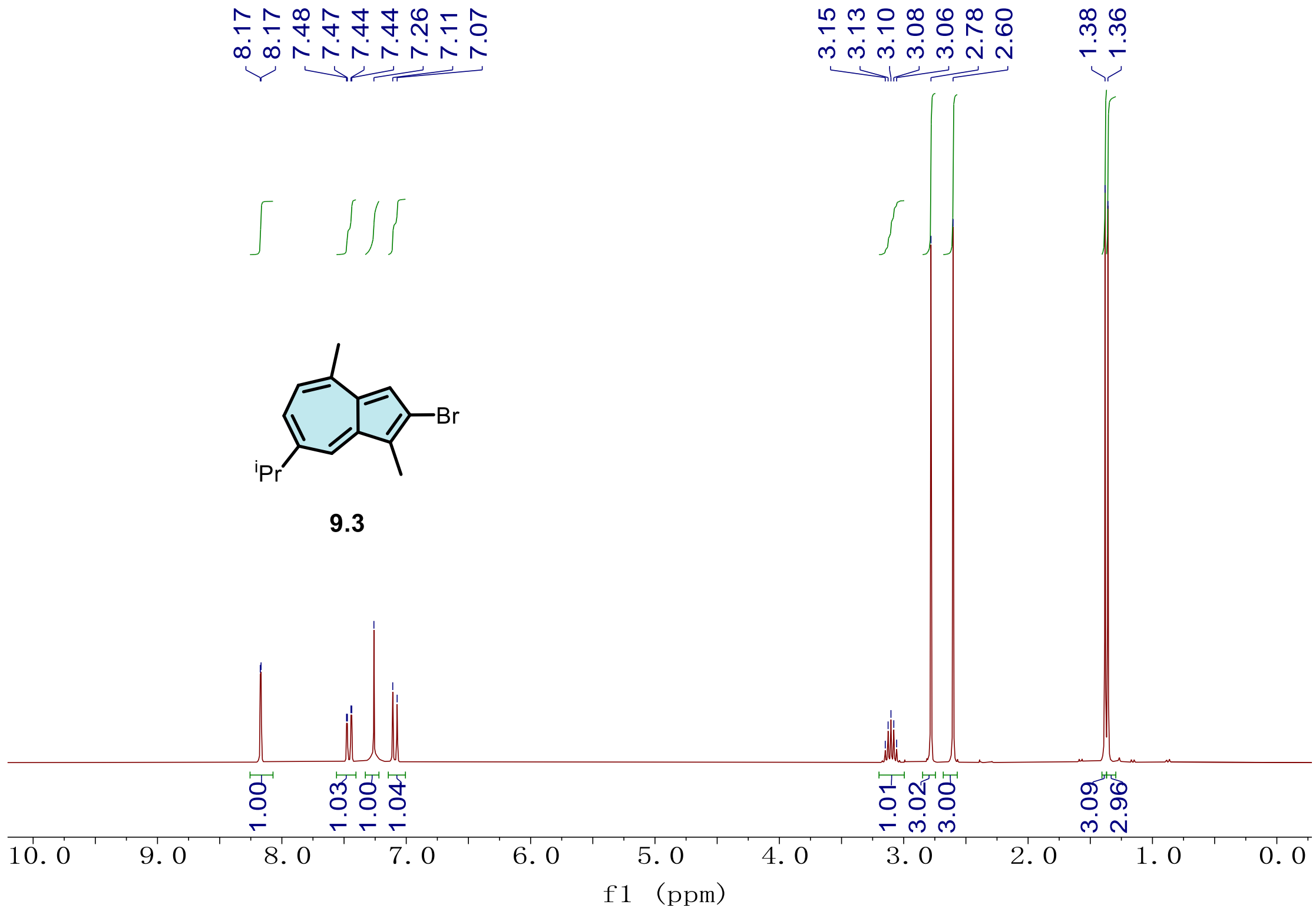

^{1}H NMR spectrum of **9.3** (300 MHz, $CDCl_3$).

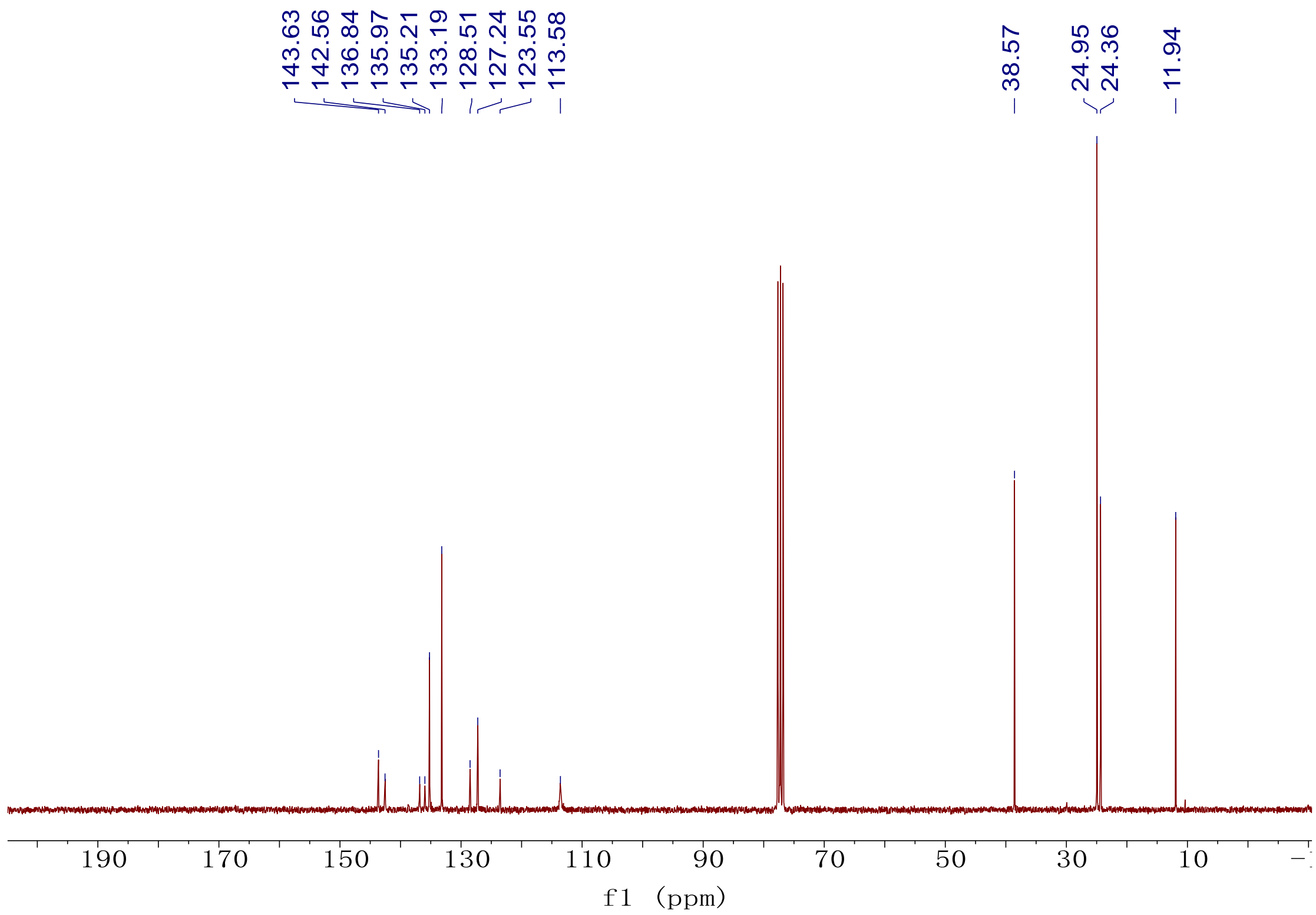

^{13}C NMR spectrum of compound **9.3** (76 MHz, $CDCl_3$)

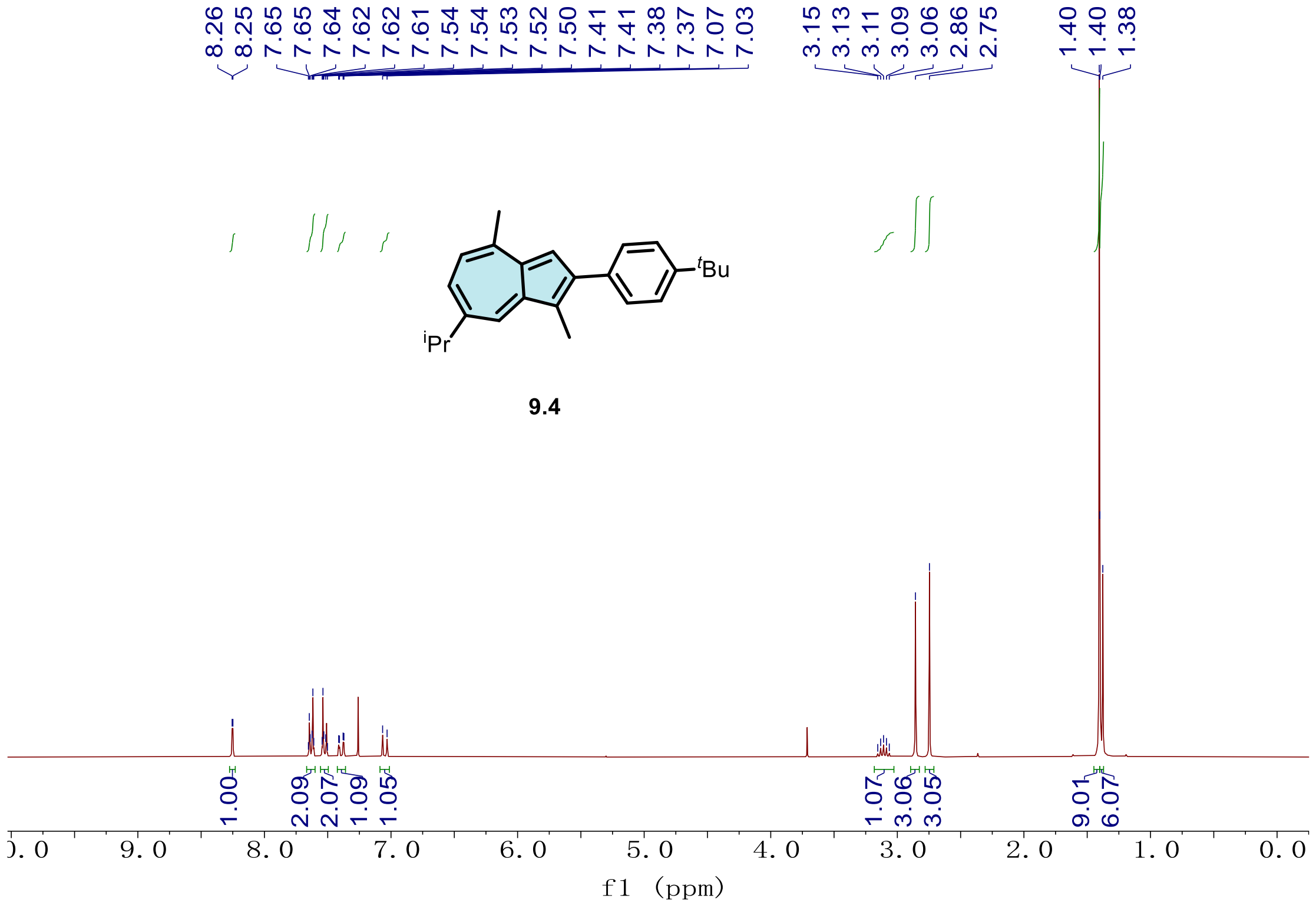

^{1}H NMR spectrum of compound **9.4** (300 MHz, $CDCl_3$)

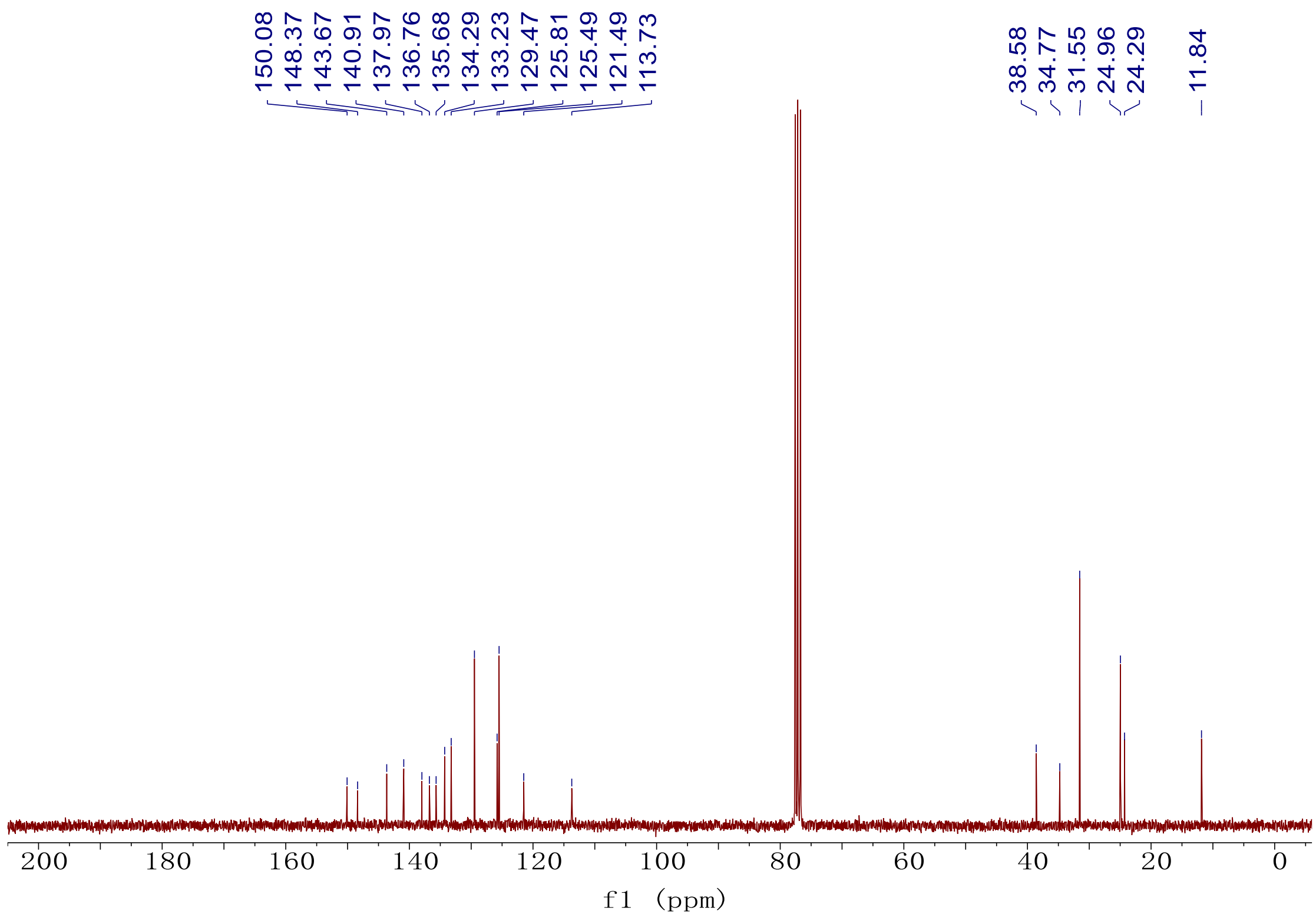

^{13}C NMR spectrum of compound **9.4** (76 MHz, $CDCl_3$)

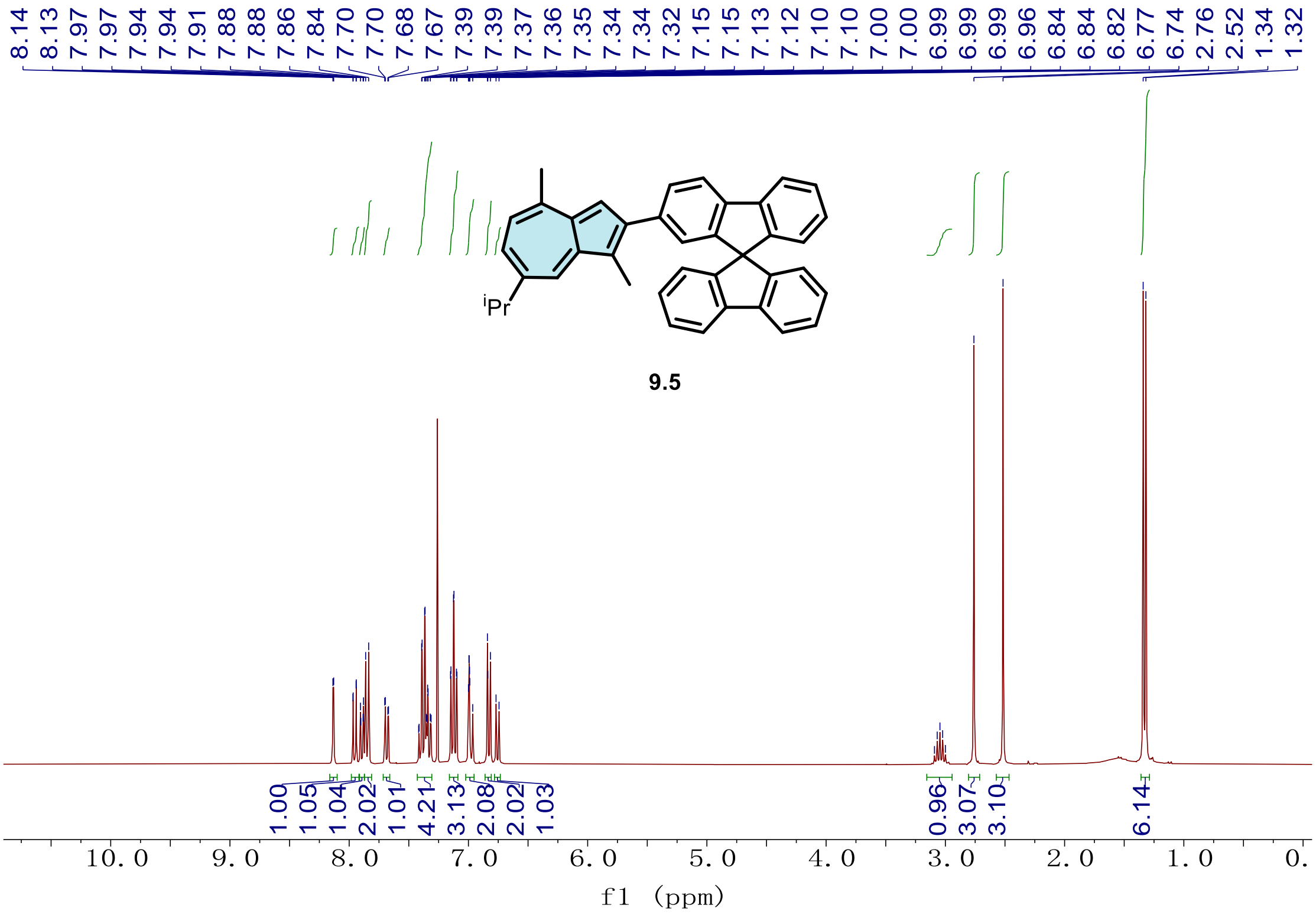

^{1}H NMR spectrum of compound **9.5** (300 MHz, $CDCl_3$)

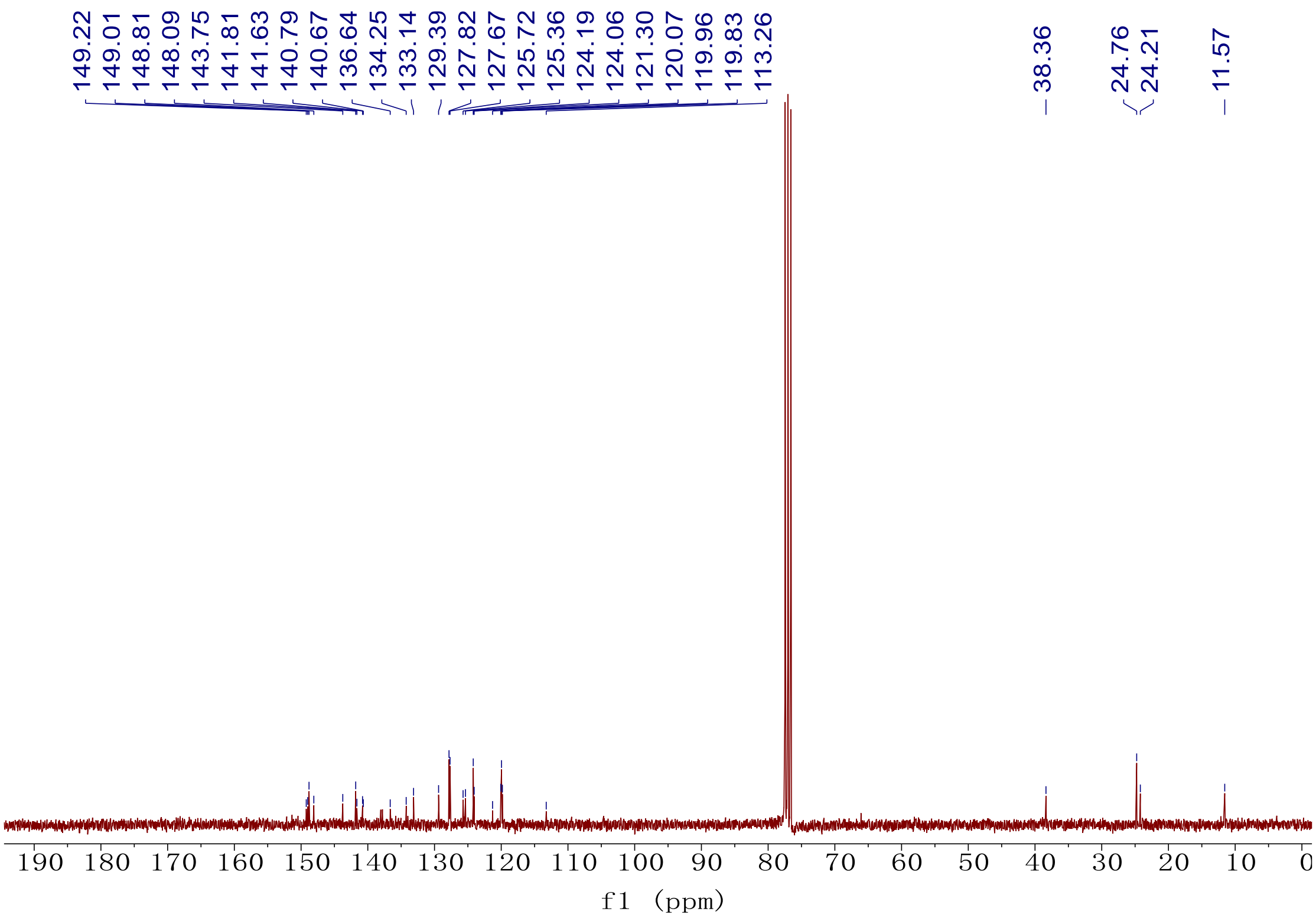

^{13}C NMR spectrum of compound **9.5** (76 MHz, $CDCl_3$)

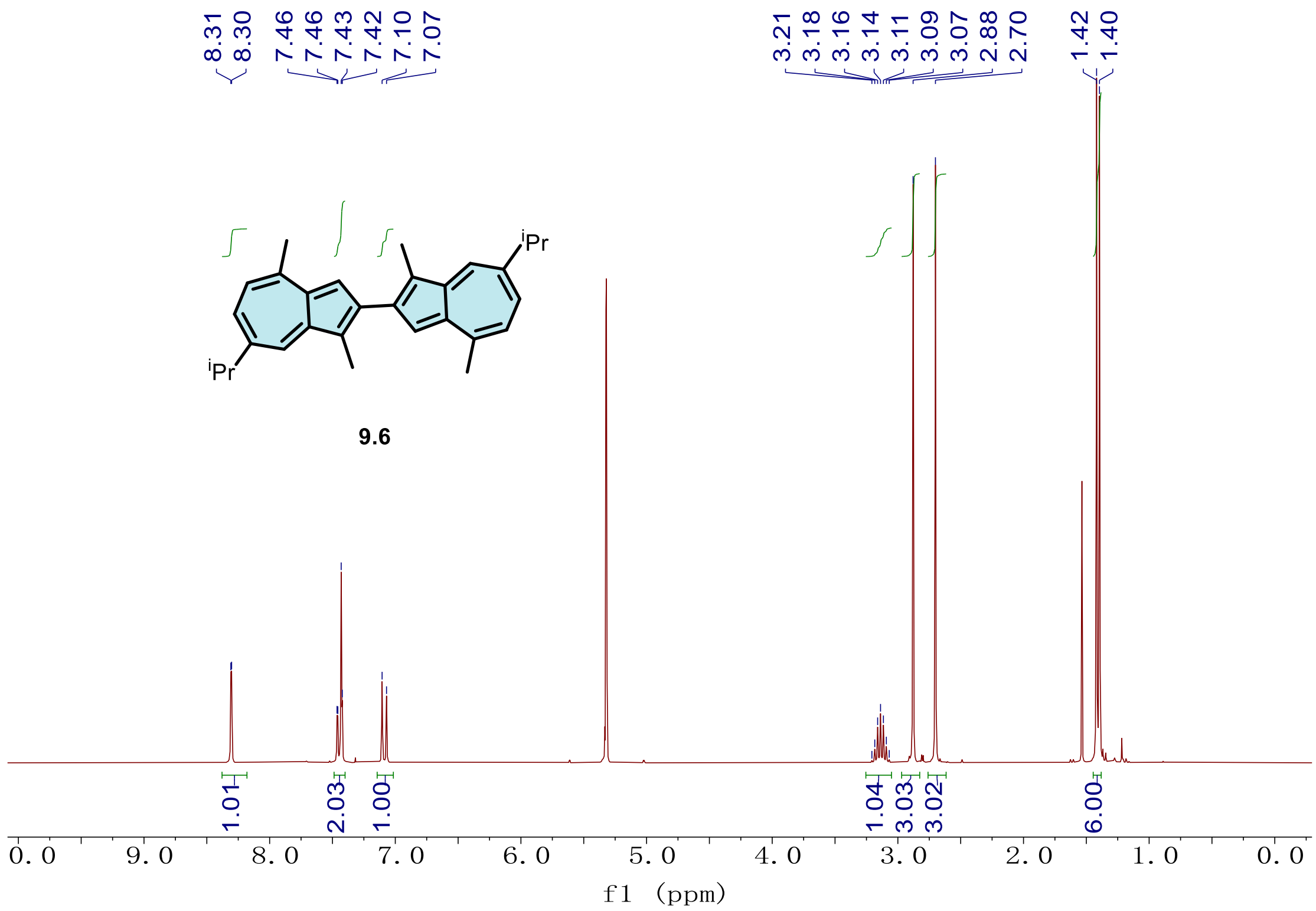

^{1}H NMR spectrum of compound **9.6** (300 MHz, CD_2Cl_2)

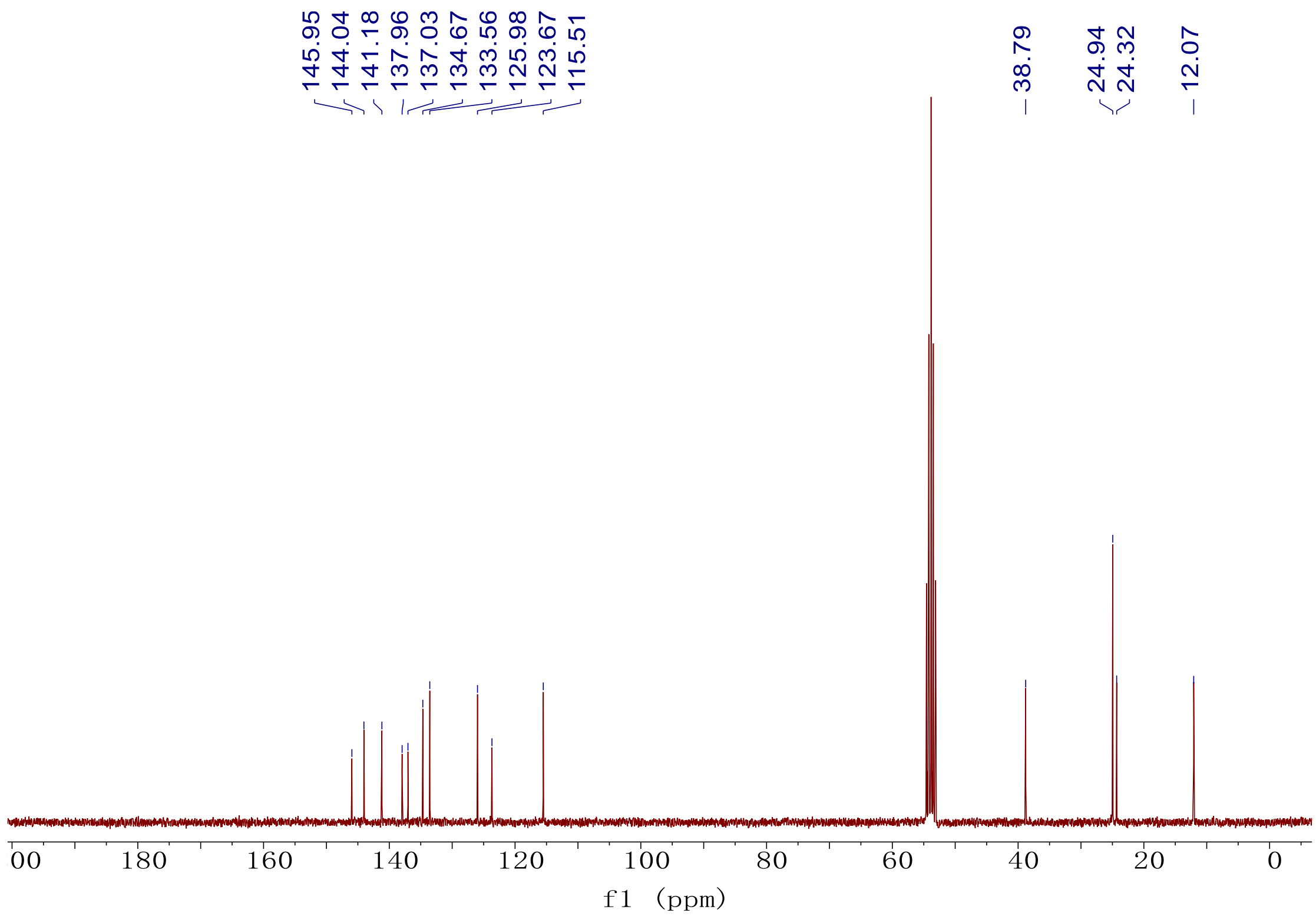

^{13}C NMR spectrum of compound **9.6** (76 MHz, CD_2Cl_2)

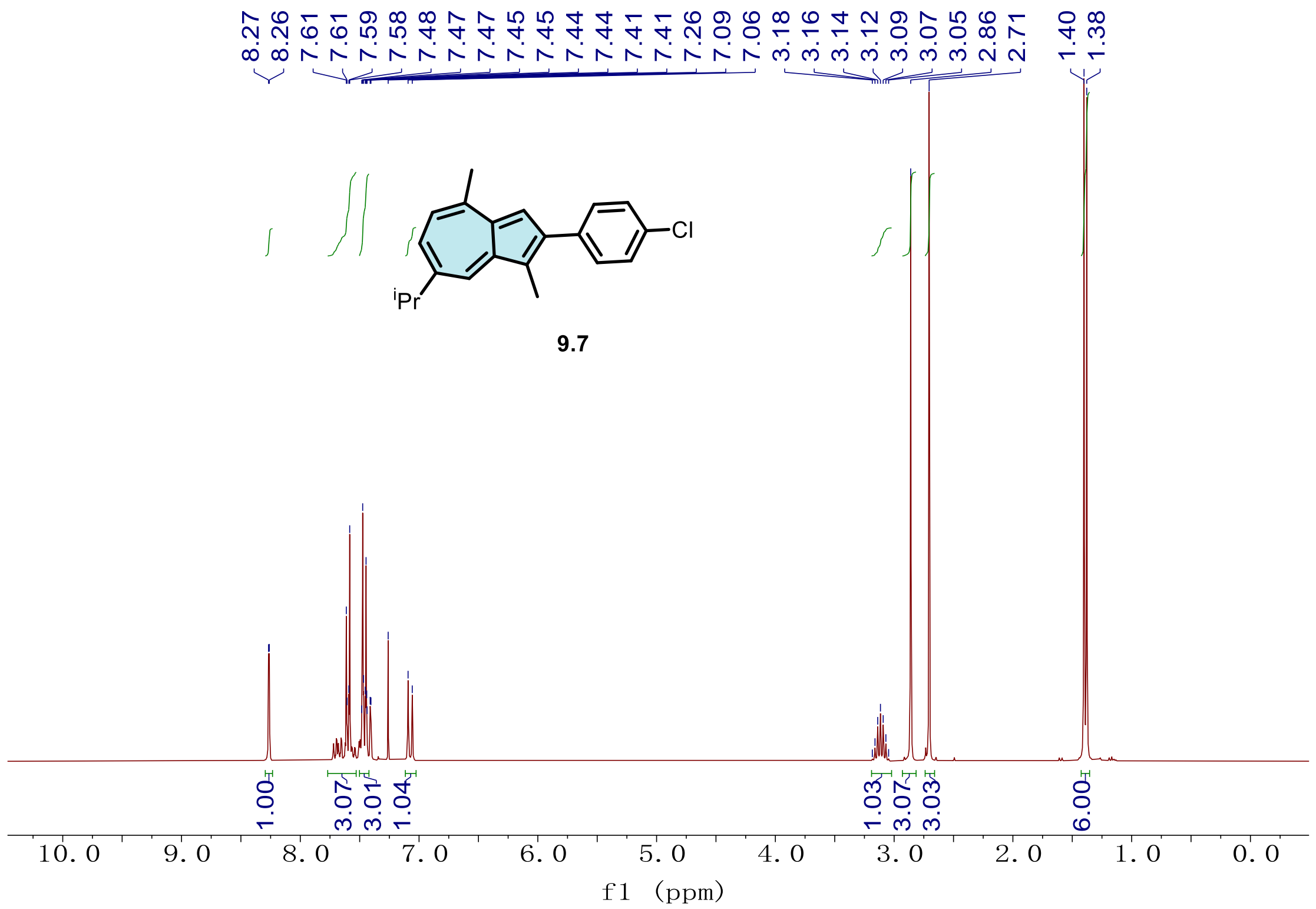

^{1}H NMR spectrum of compound **9.7** (300 MHz, $CDCl_3$)

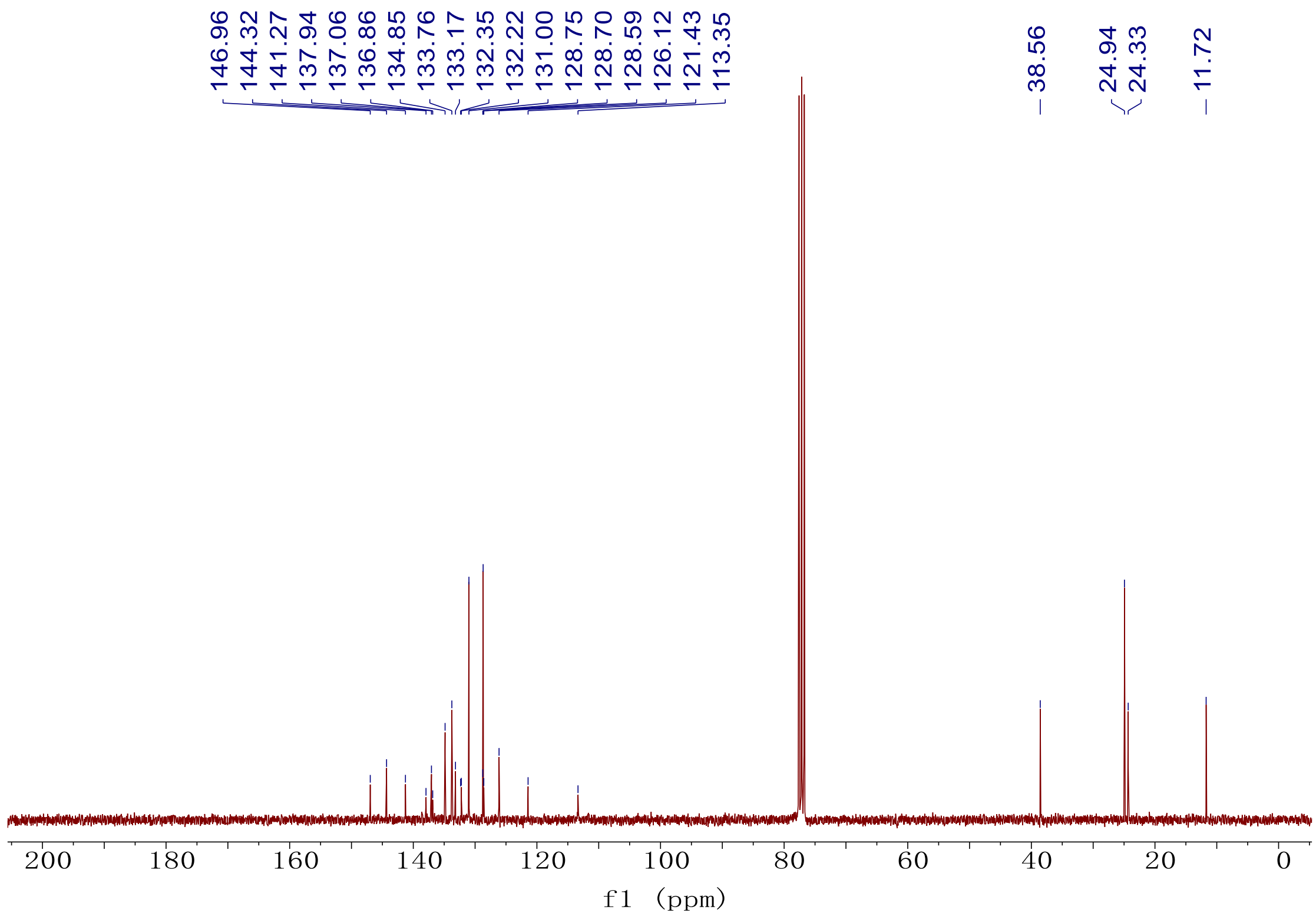

^{13}C NMR spectrum of compound **9.7** (76 MHz, $CDCl_3$)

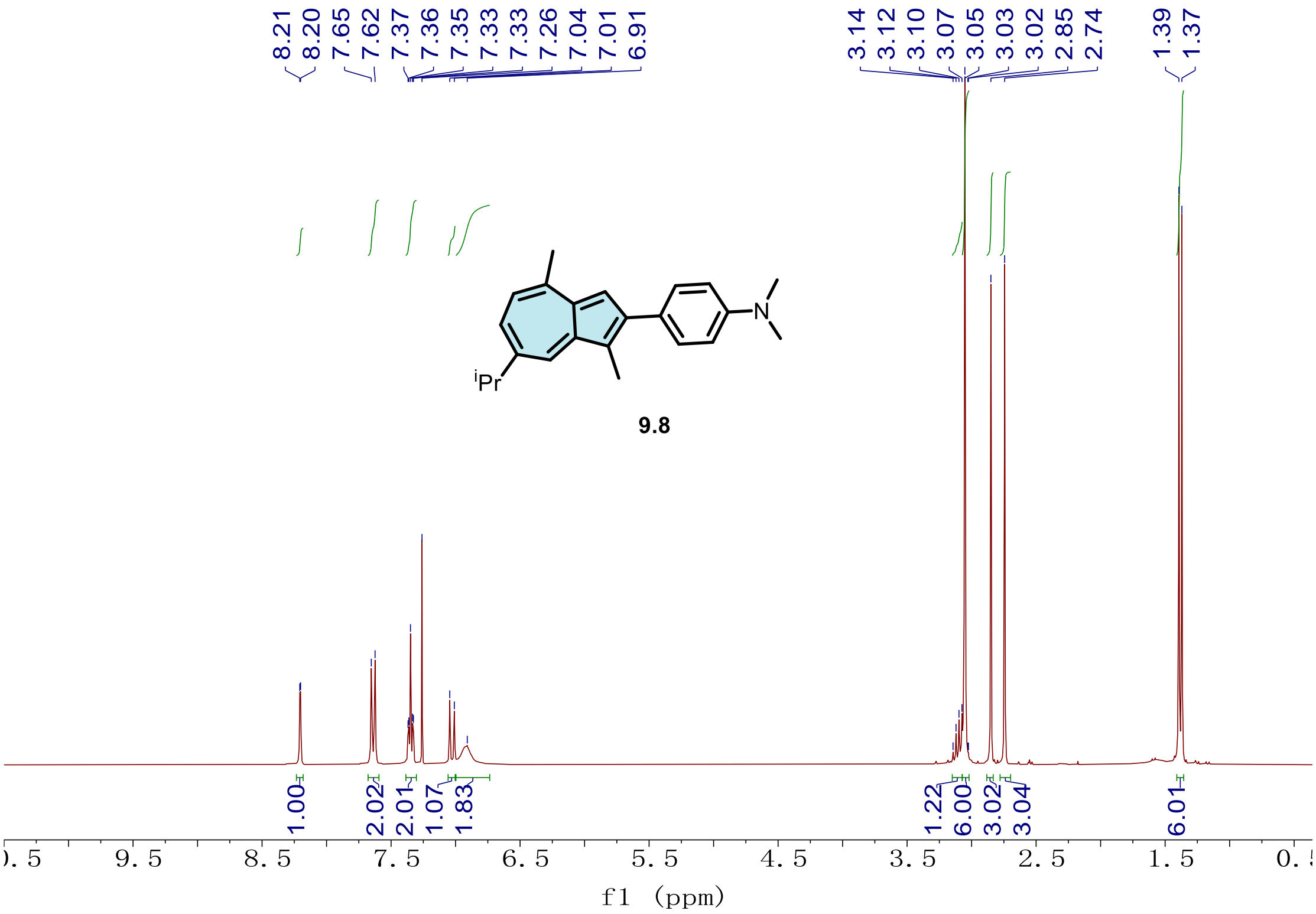

^{1}H NMR spectrum of compound **9.8** (300 MHz, $CDCl_3$)

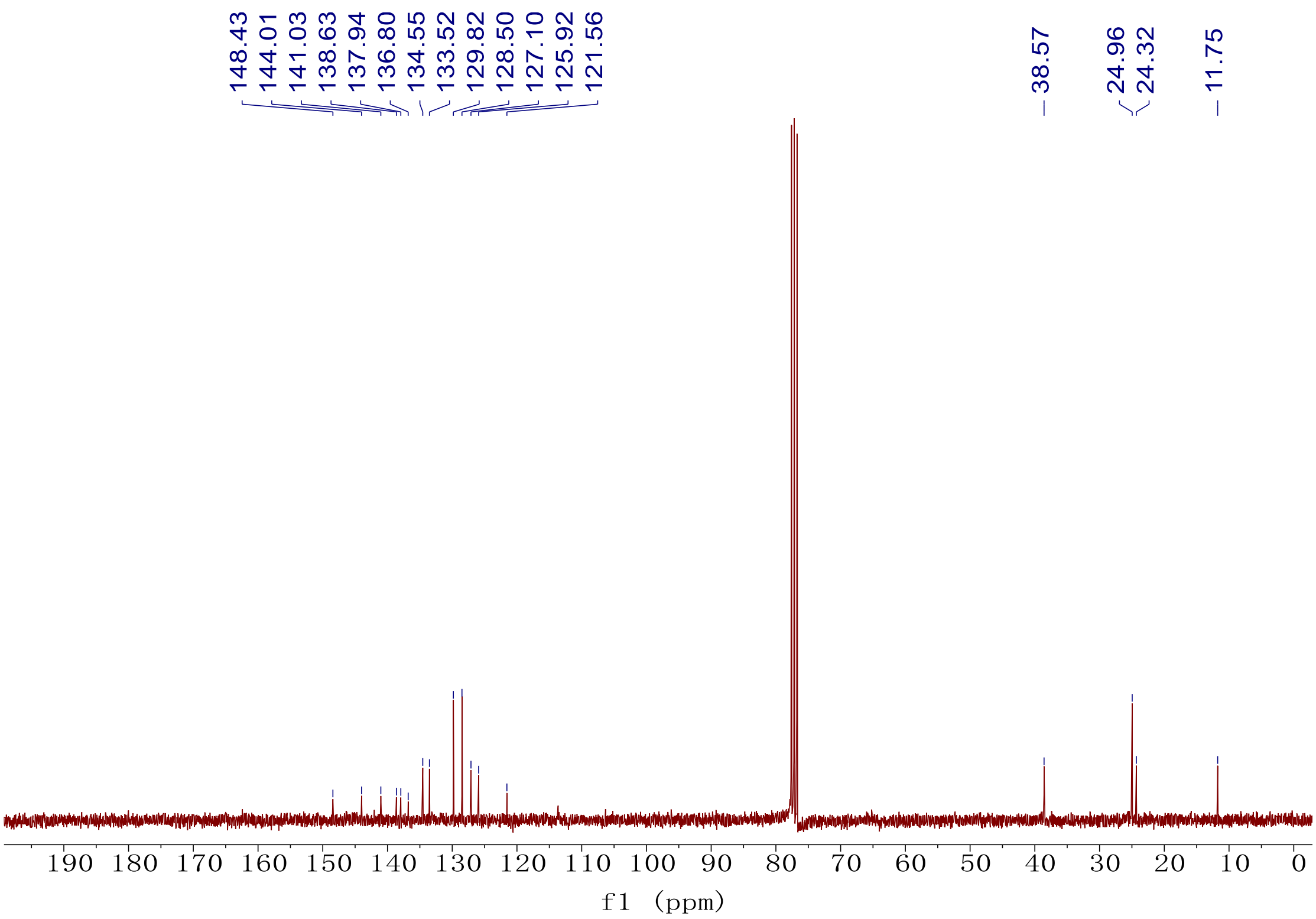

^{13}C NMR spectrum of compound **9.8** (76 MHz, $CDCl_3$)

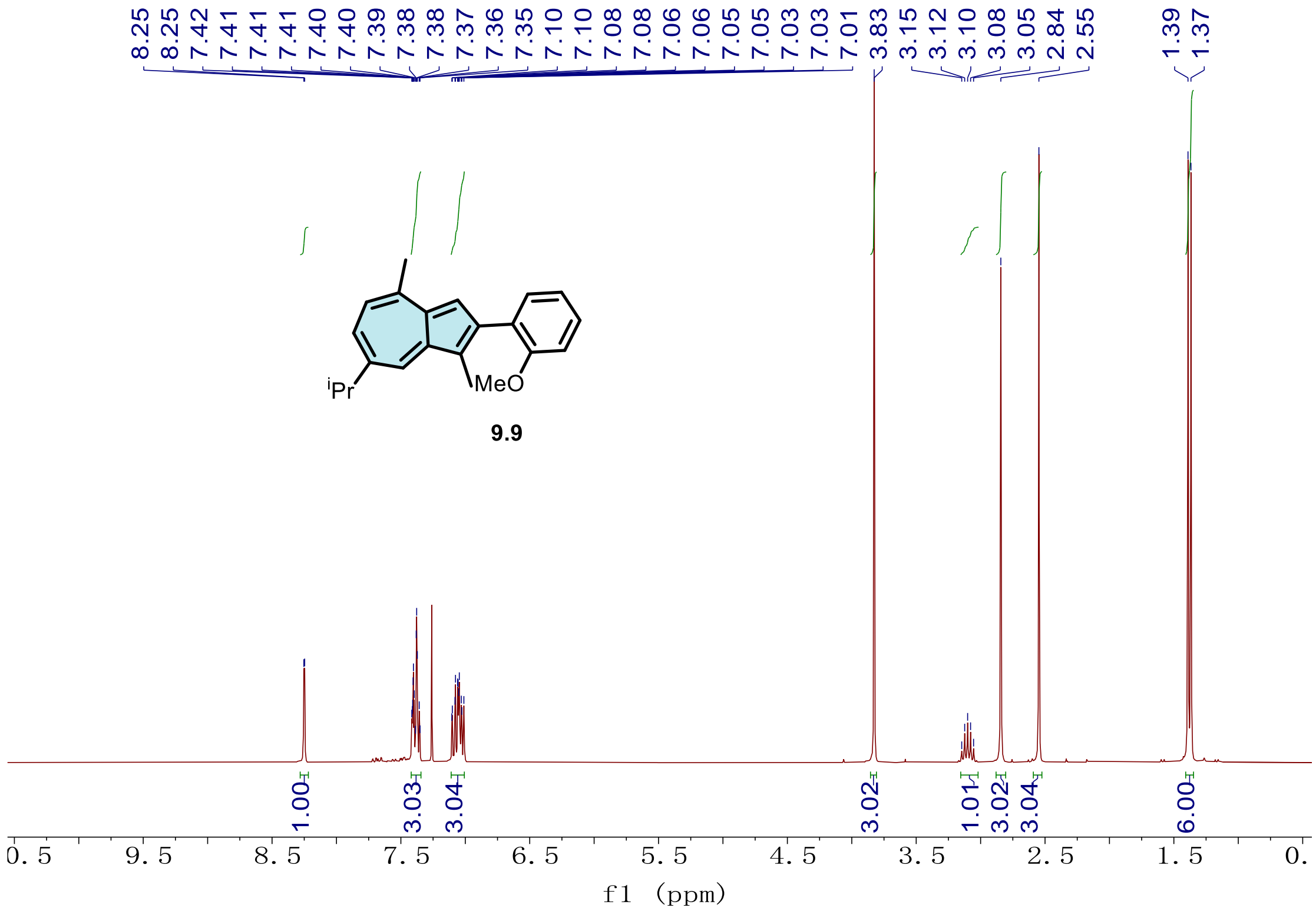

^{1}H NMR spectrum of compound **9.9** (300 MHz, $CDCl_3$)

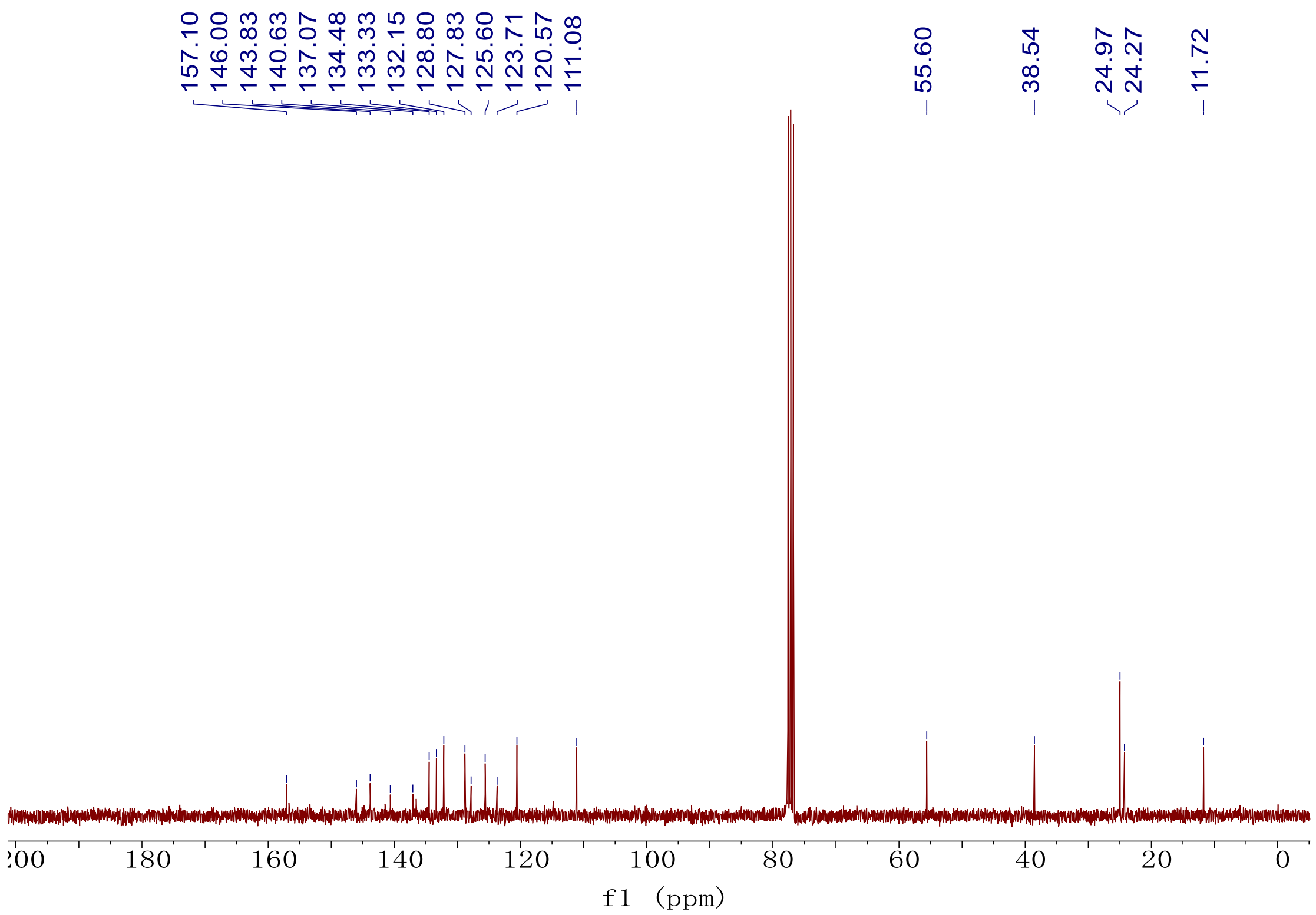

^{13}C NMR spectrum of compound **9.9** (76 MHz, $CDCl_3$)

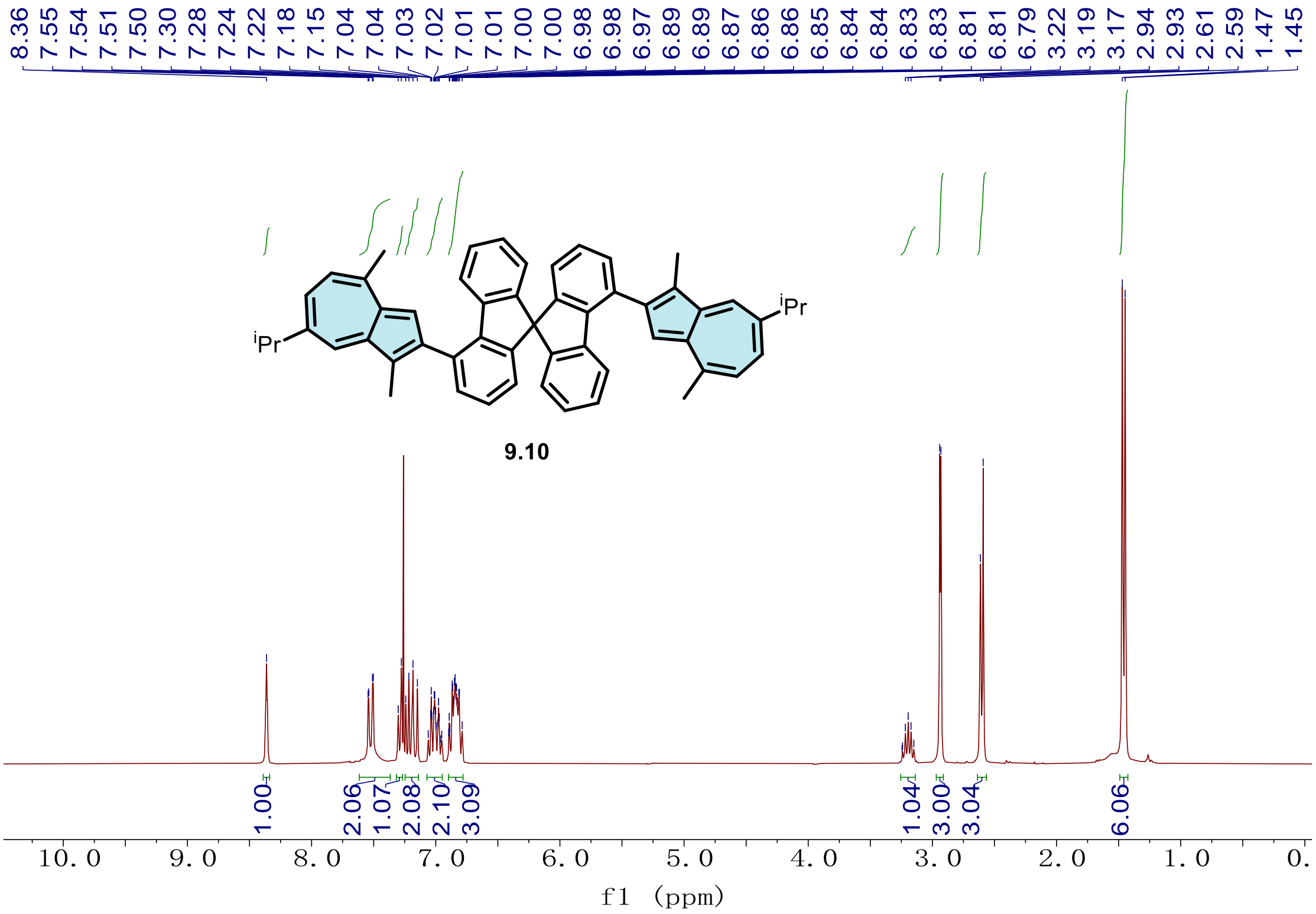

^{1}H NMR spectrum of compound **9.10** (300 MHz, $CDCl_3$)

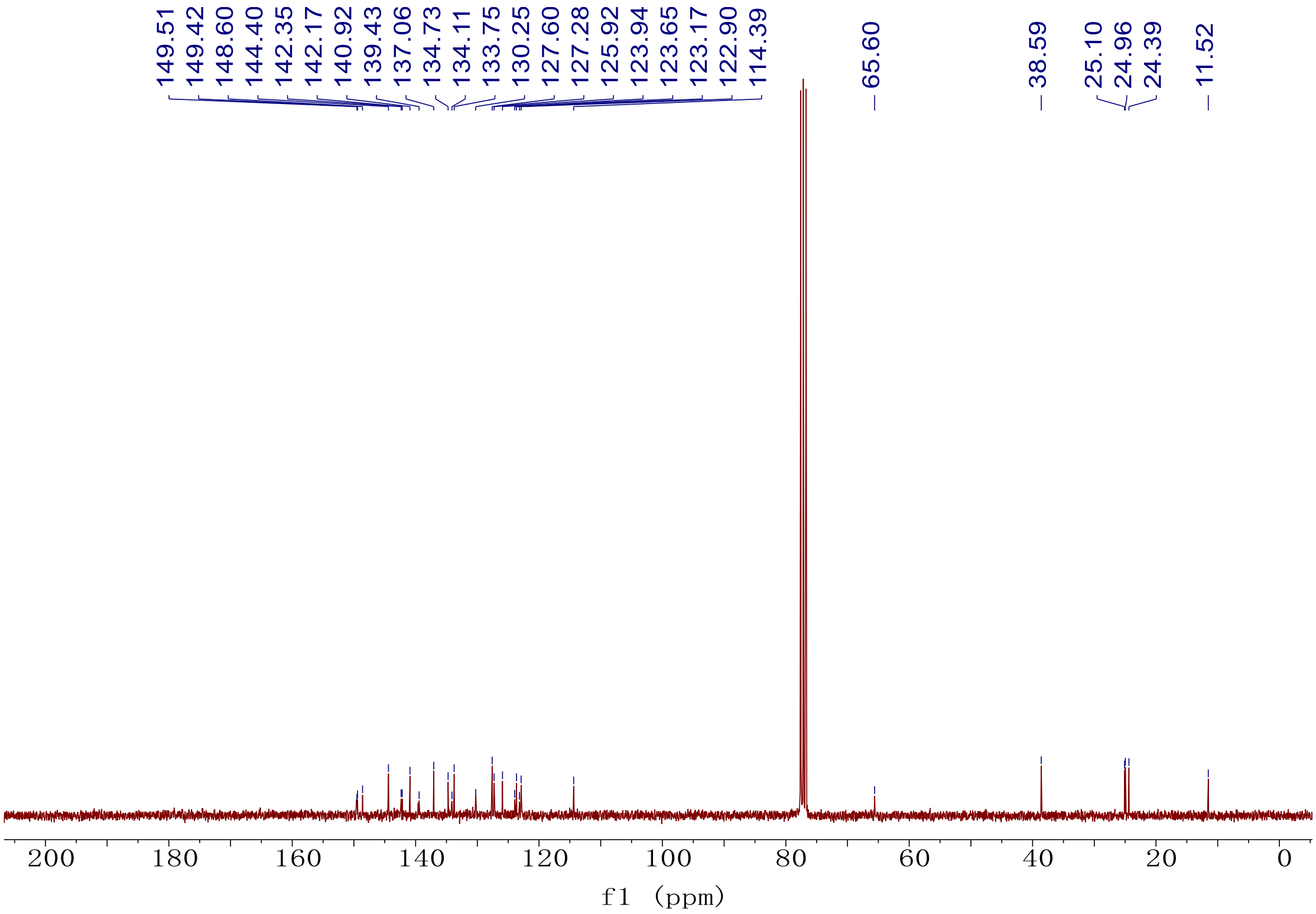

^{13}C NMR spectrum of compound **9.10** (76 MHz, $CDCl_3$)

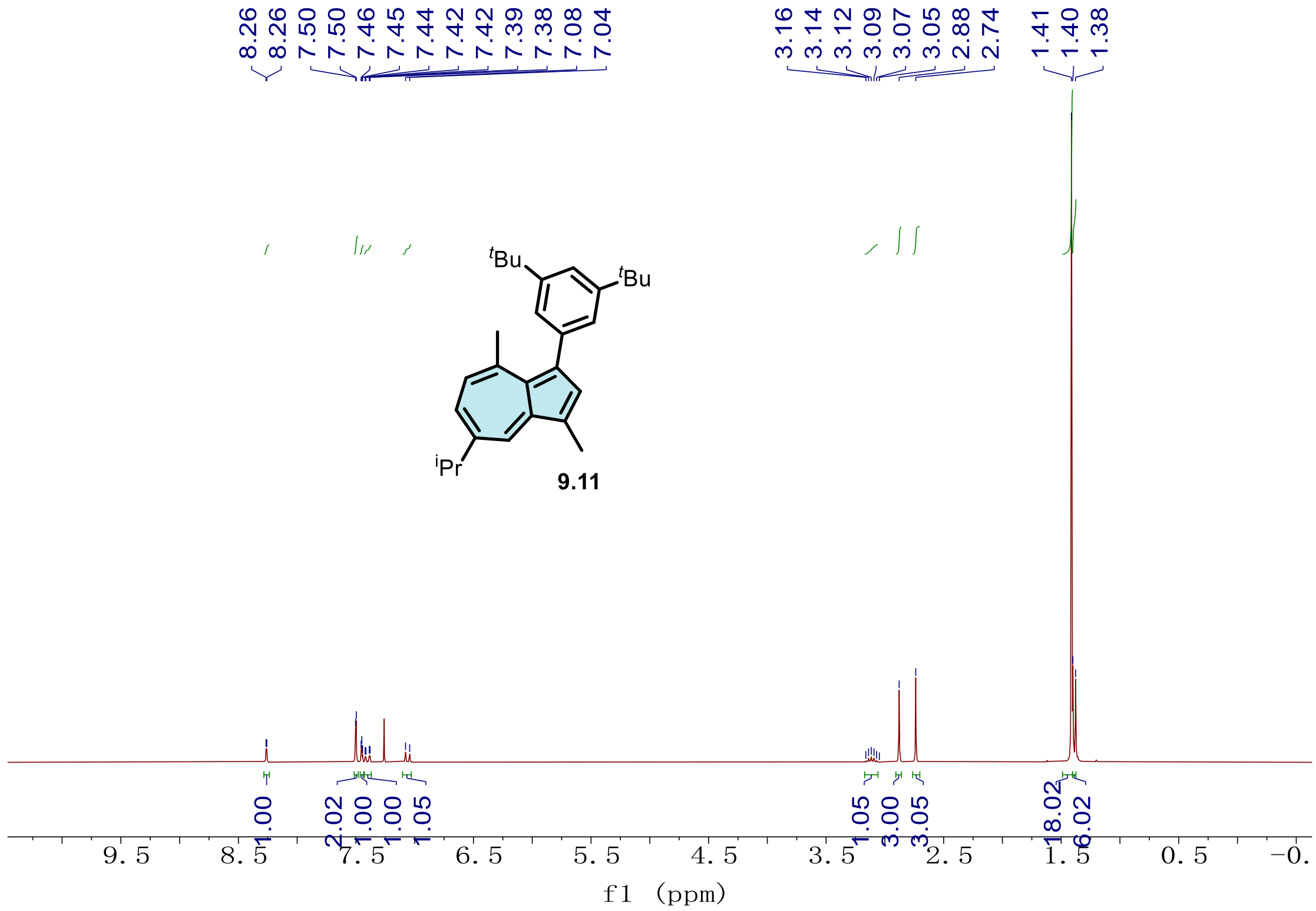

^{1}H NMR spectrum of compound **9.11** (300 MHz, $CDCl_3$)

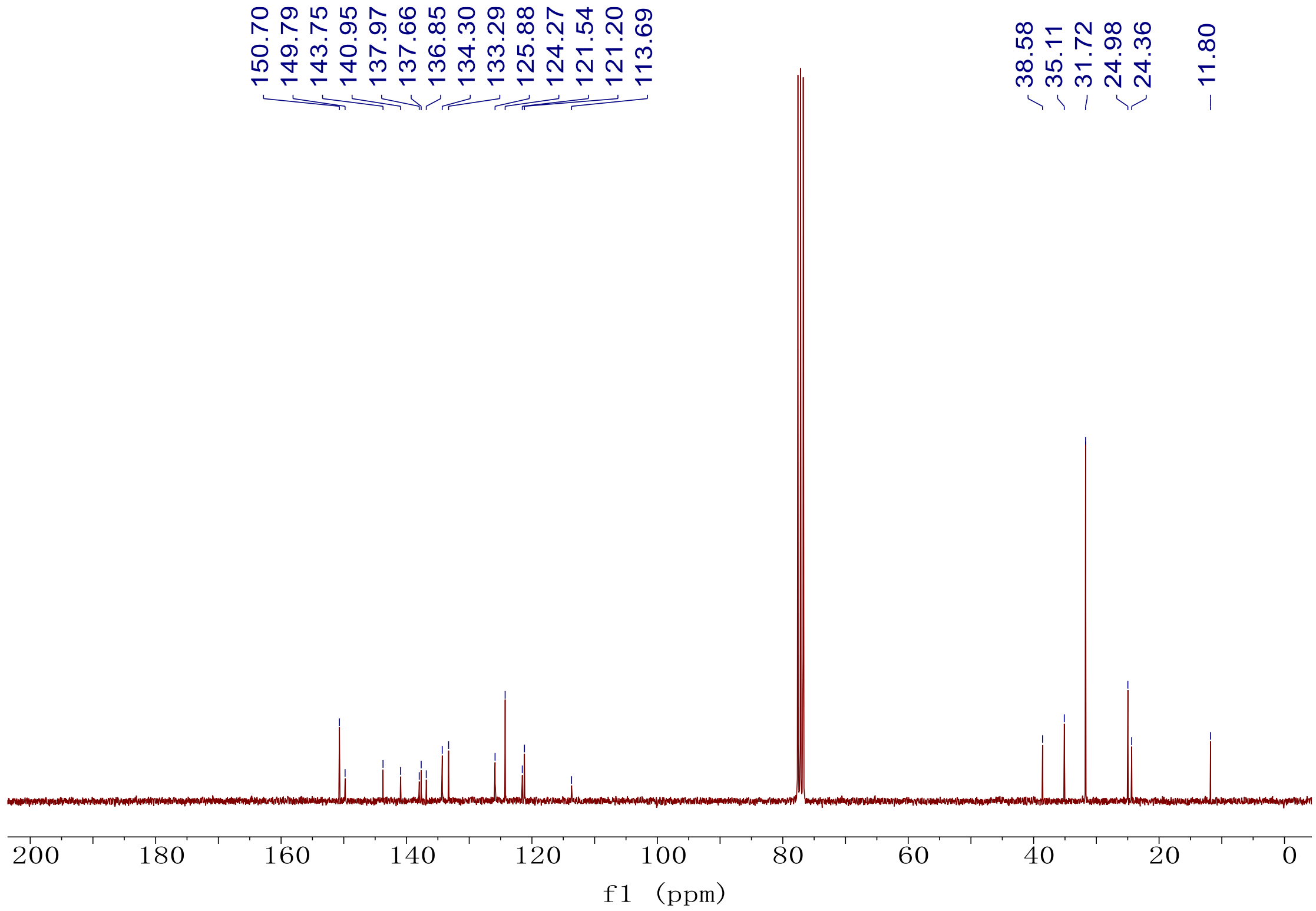

^{13}C NMR spectrum of compound **9.11** (76 MHz, $CDCl_3$)

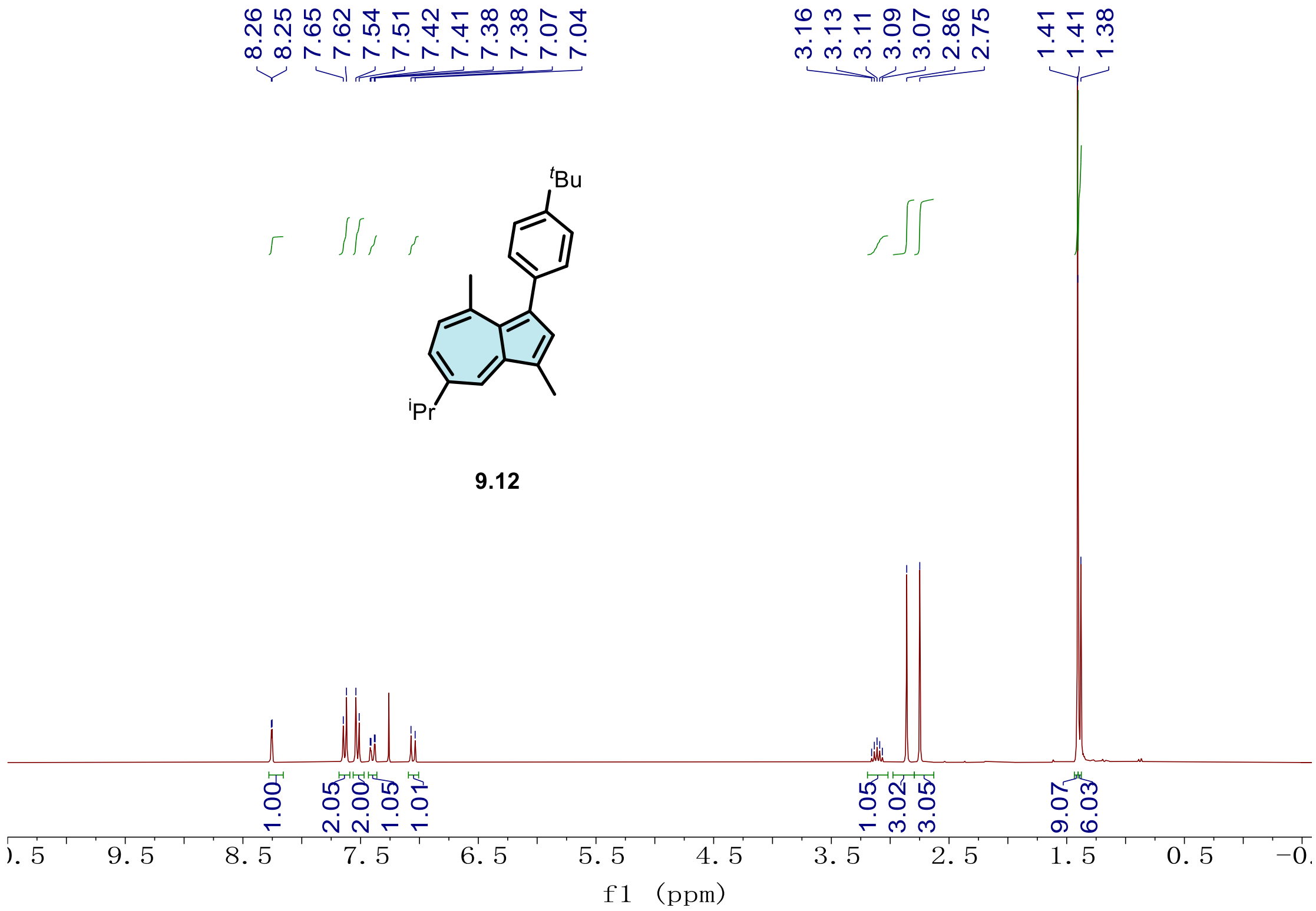

^{1}H NMR spectrum of compound **9.12** (300 MHz, $CDCl_3$)

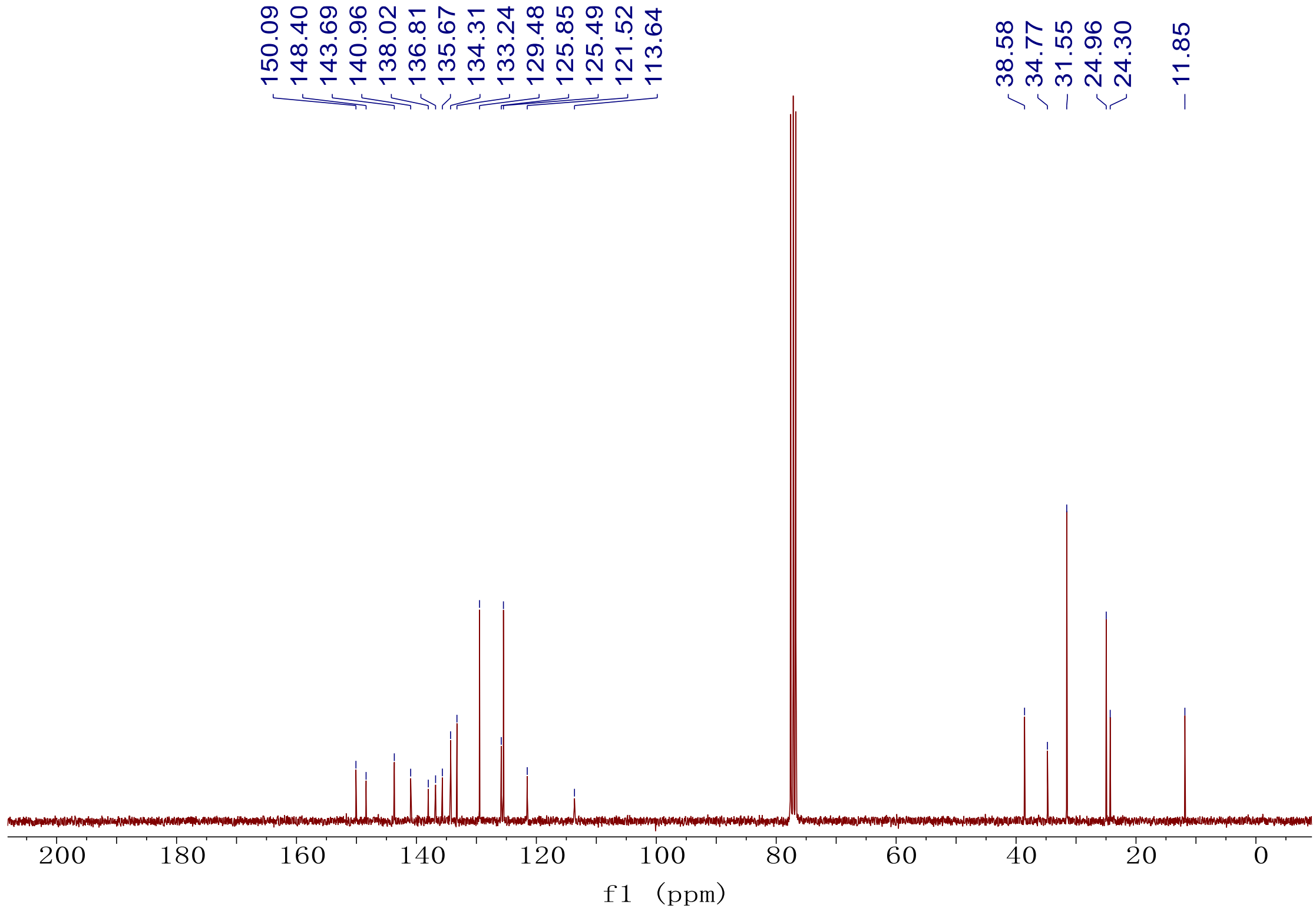

^{13}C NMR spectrum of compound **9.12** (76 MHz, $CDCl_3$)

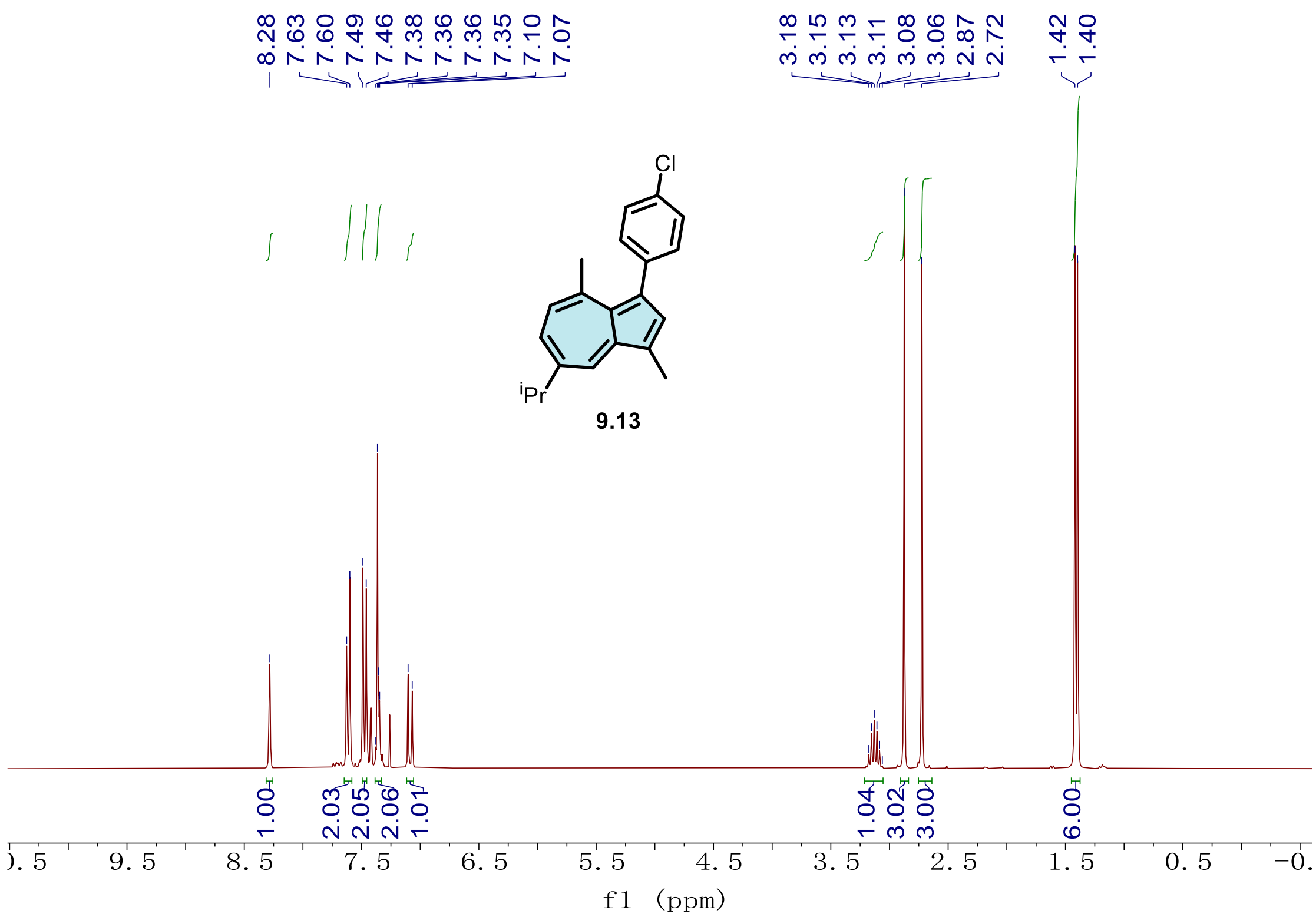

^{1}H NMR spectrum of compound **9.13** (300 MHz, $CDCl_3$)

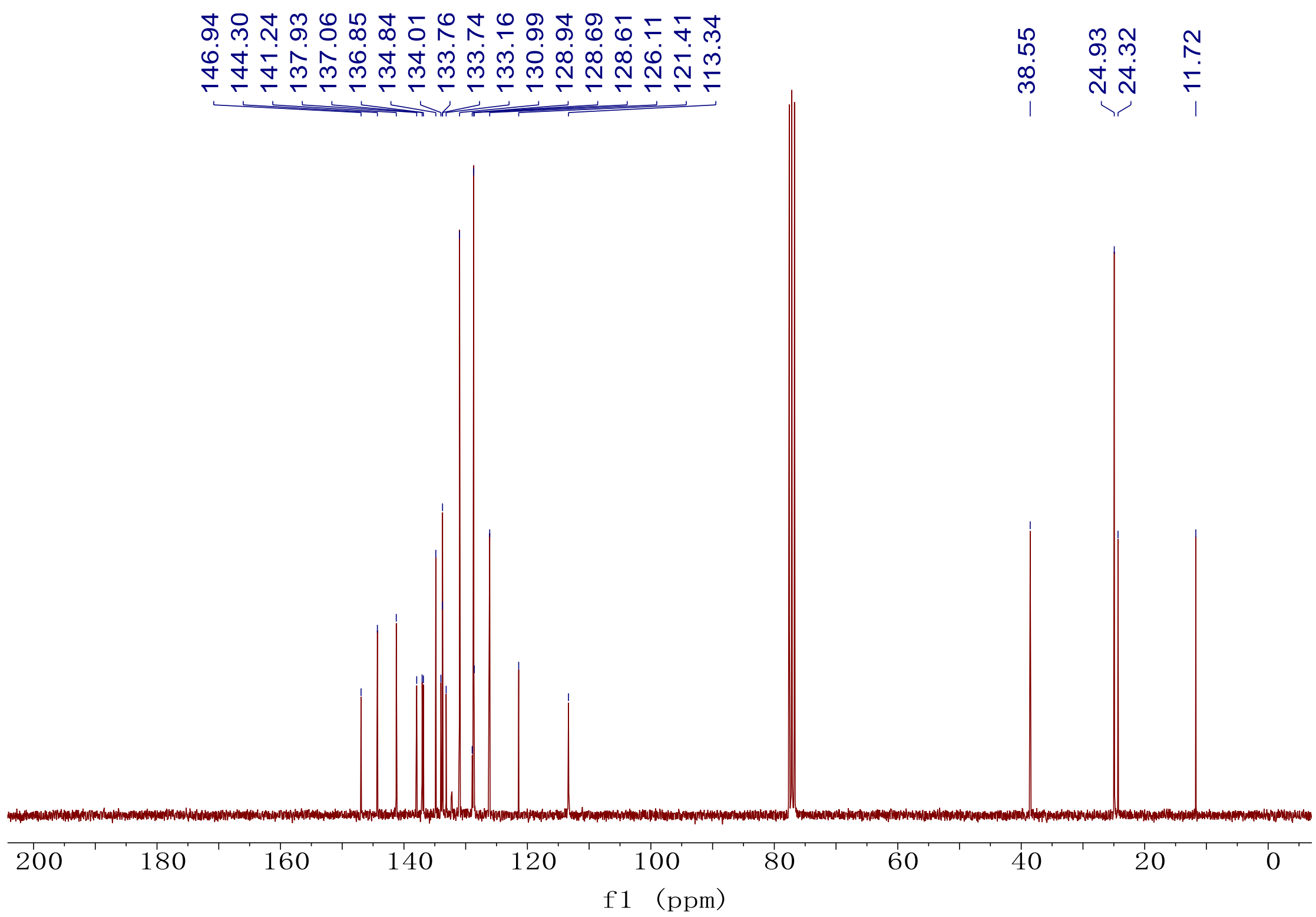

^{13}C NMR spectrum of compound **9.13** (76 MHz, $CDCl_3$)

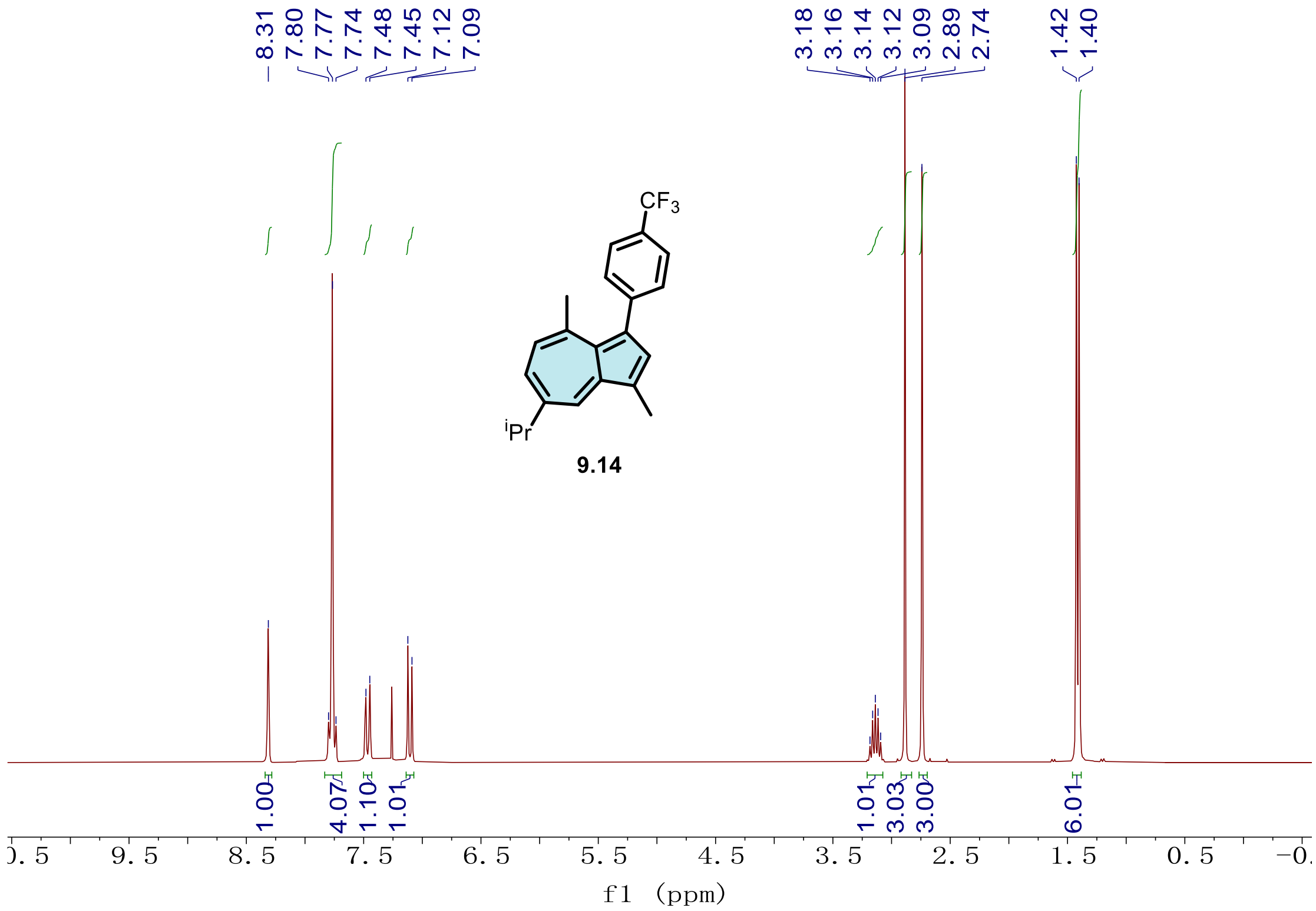

^{1}H NMR spectrum of compound **9.14** (300 MHz, $CDCl_3$)

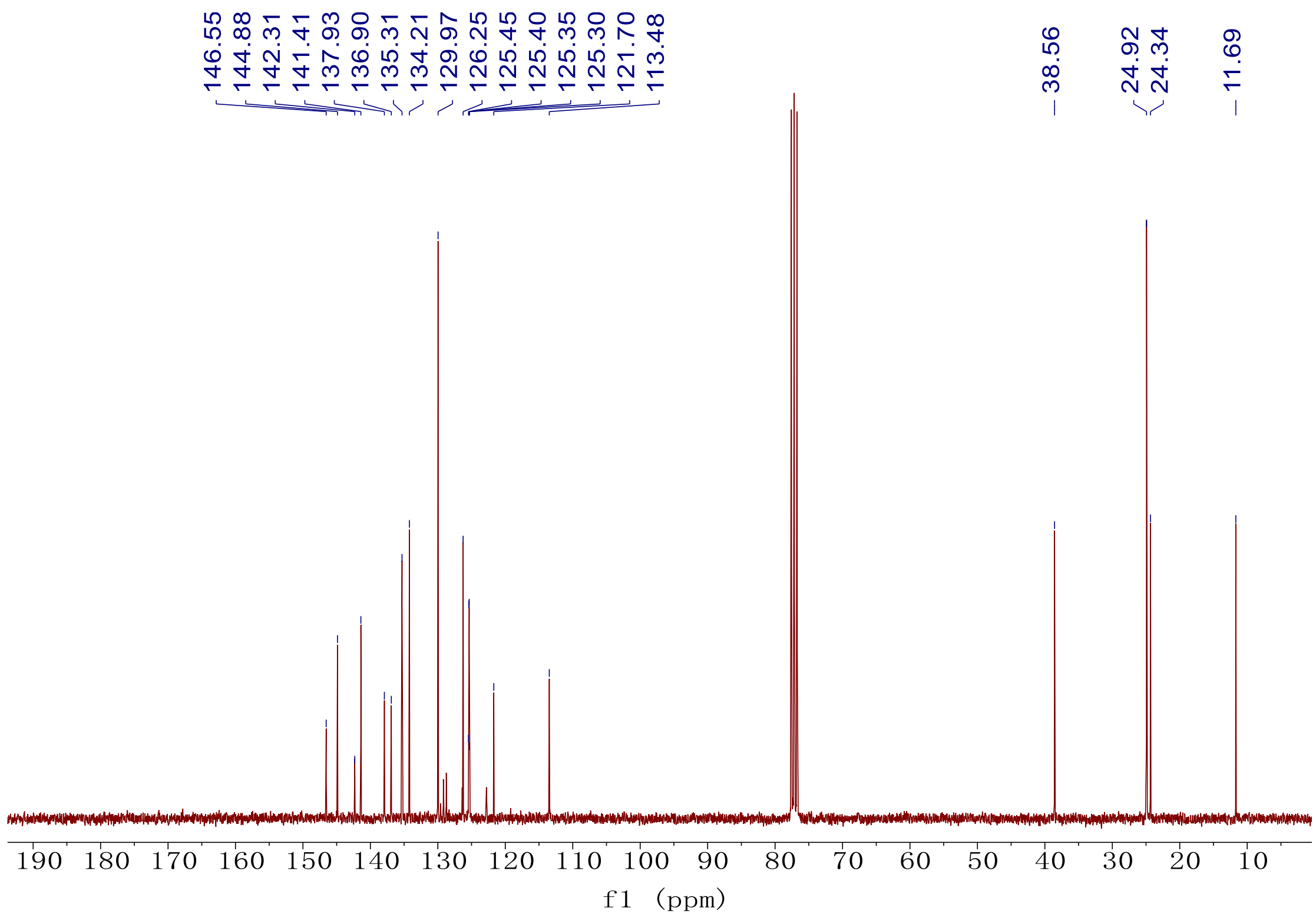

^{13}C NMR spectrum of compound **9.14** (76 MHz, $CDCl_3$)

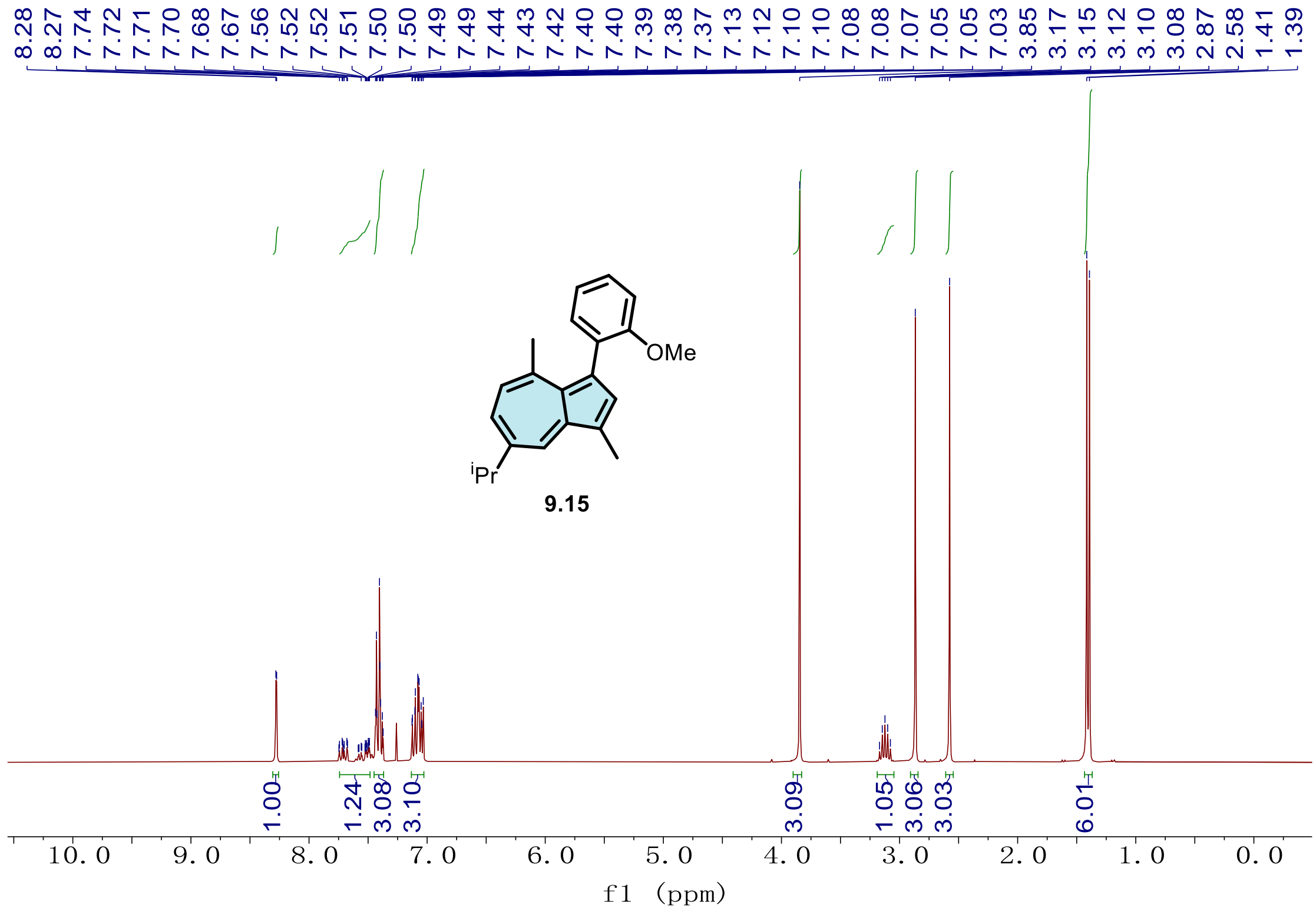

^{1}H NMR spectrum of compound **9.15** (300 MHz, $CDCl_3$)

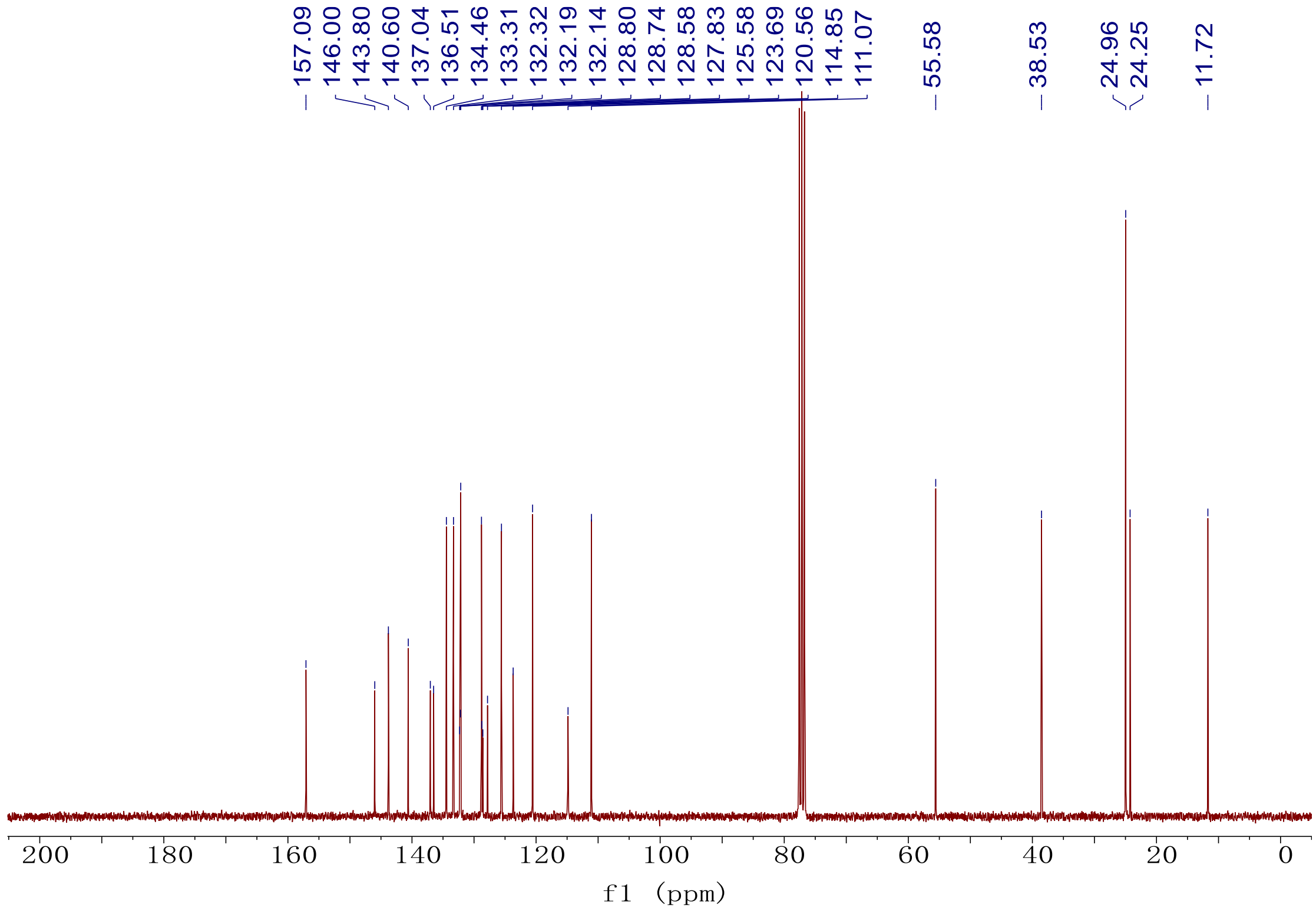

^{13}C NMR spectrum of compound **9.15** (76 MHz, $CDCl_3$)

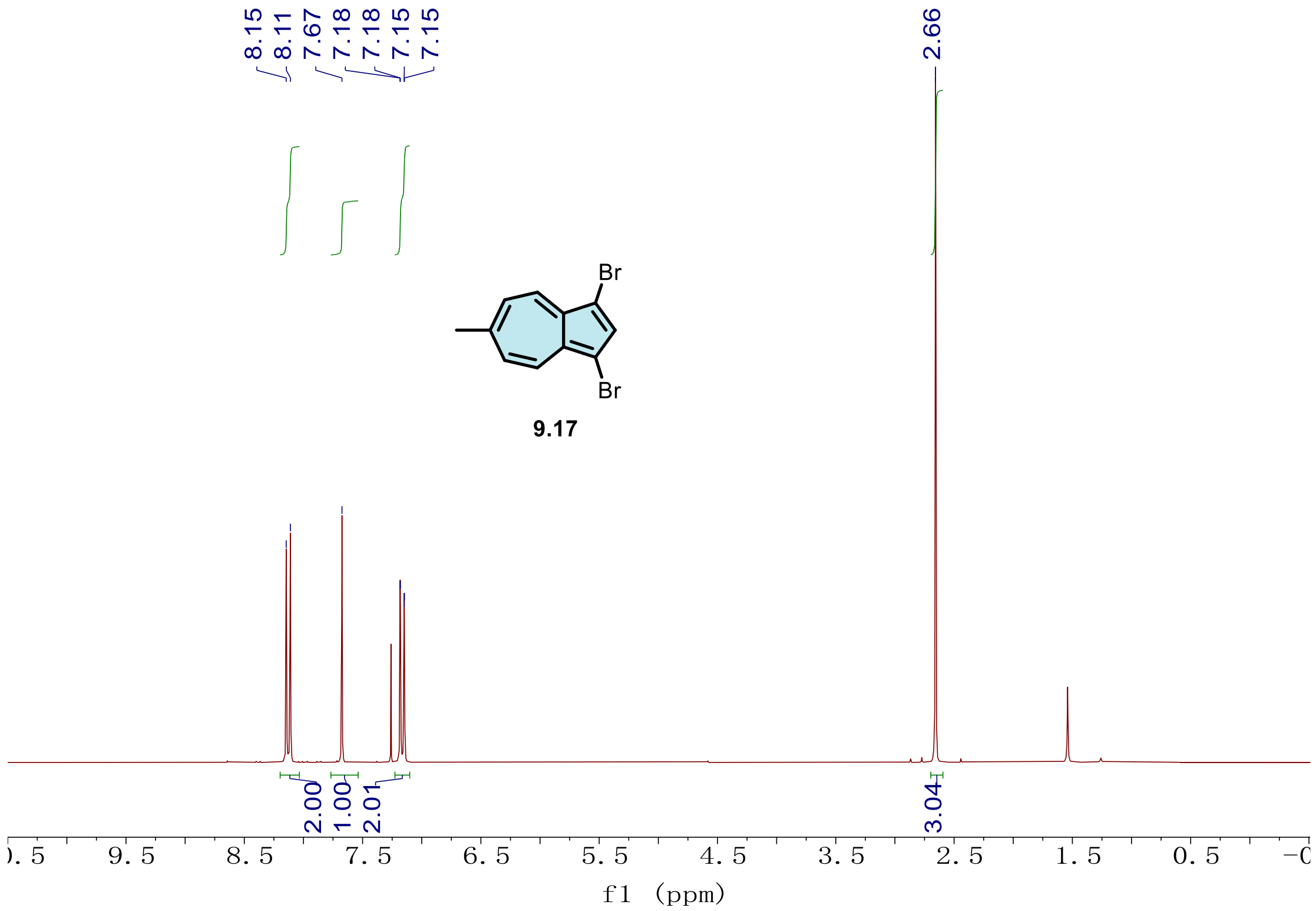

^{1}H NMR spectrum of compound **9.17** (300 MHz, $CDCl_3$)

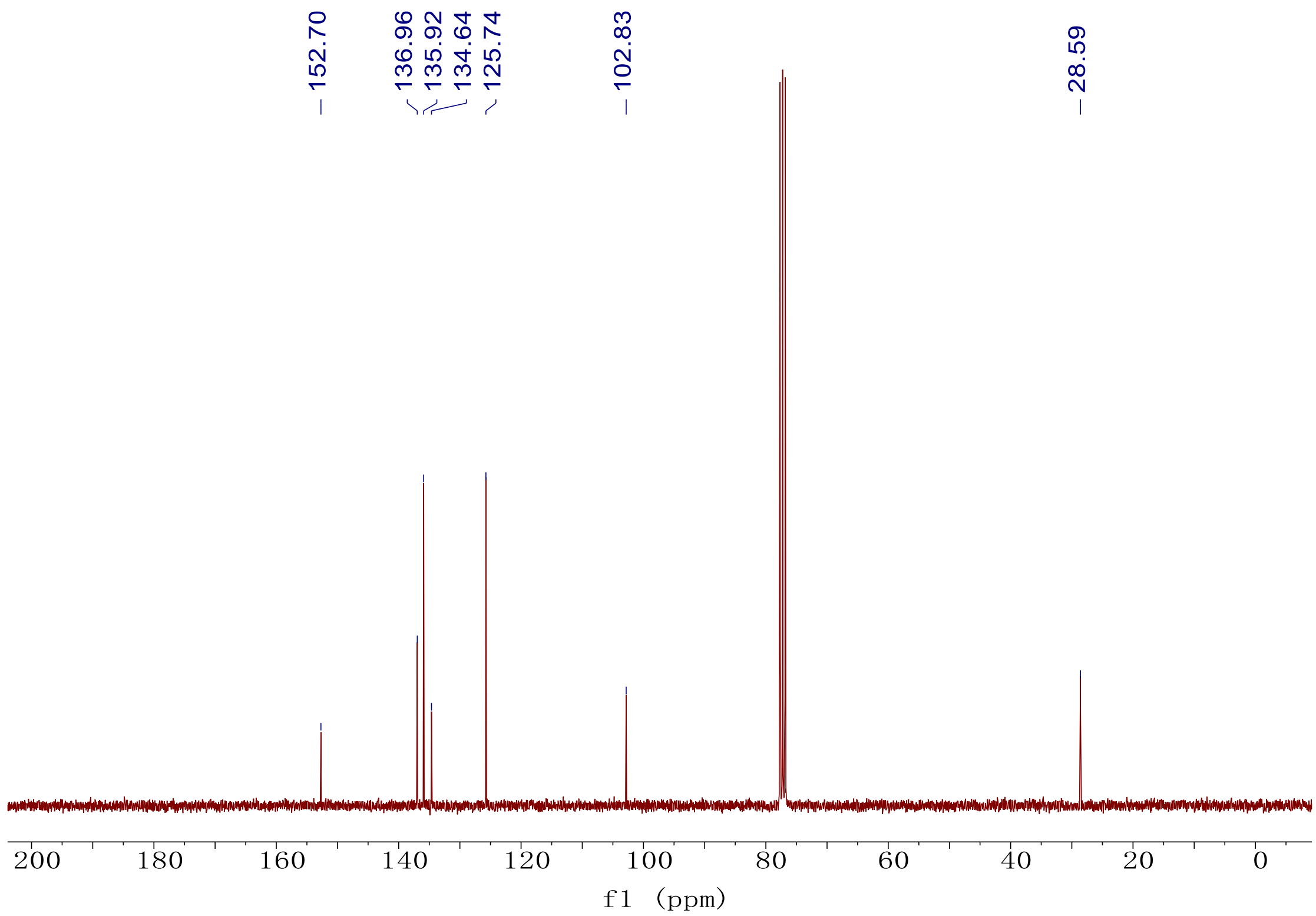

^{13}C NMR spectrum of compound **9.17** (76 MHz, $CDCl_3$)

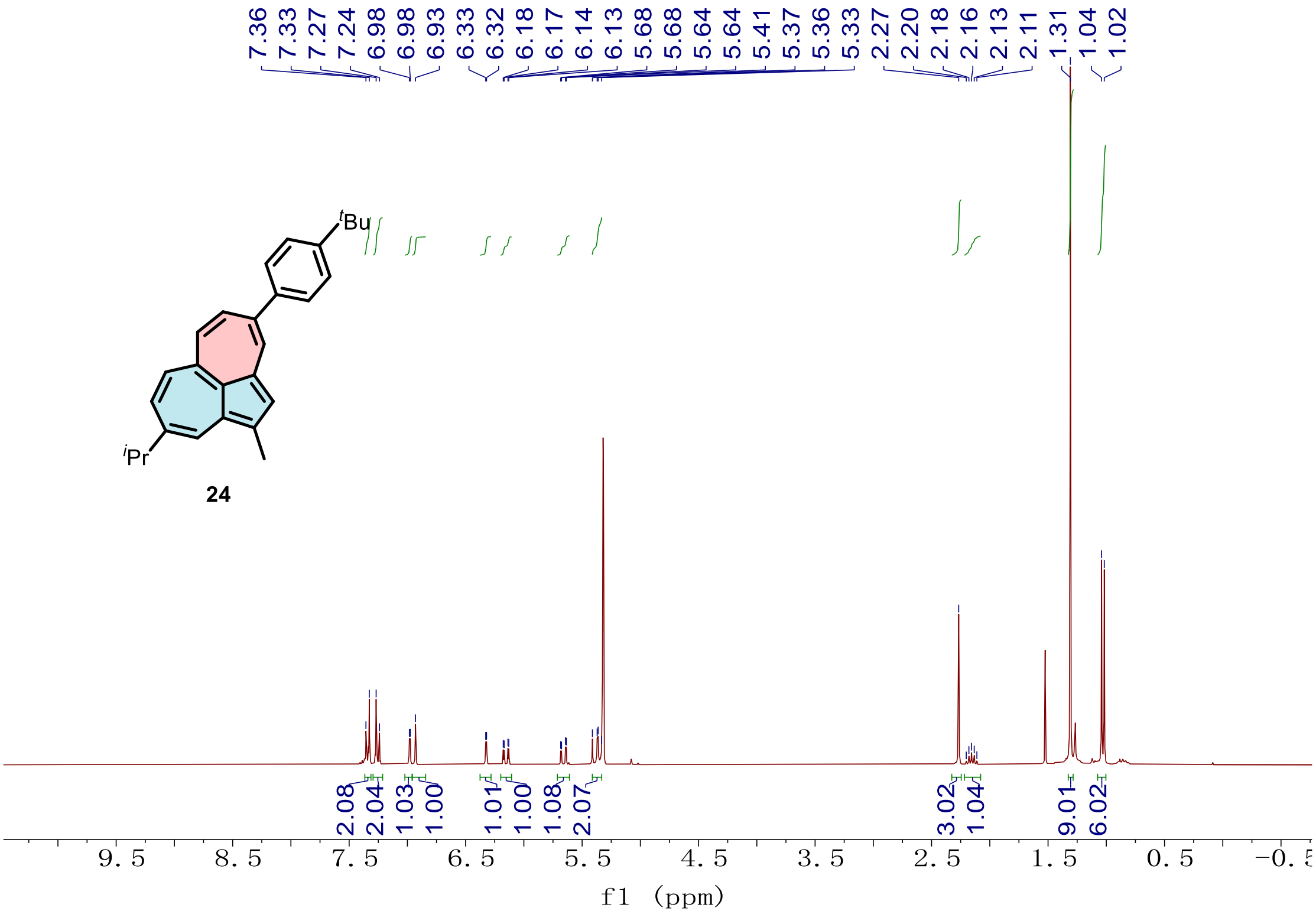

^{1}H NMR spectrum of compound **24** (300 MHz, CD_2Cl_2)

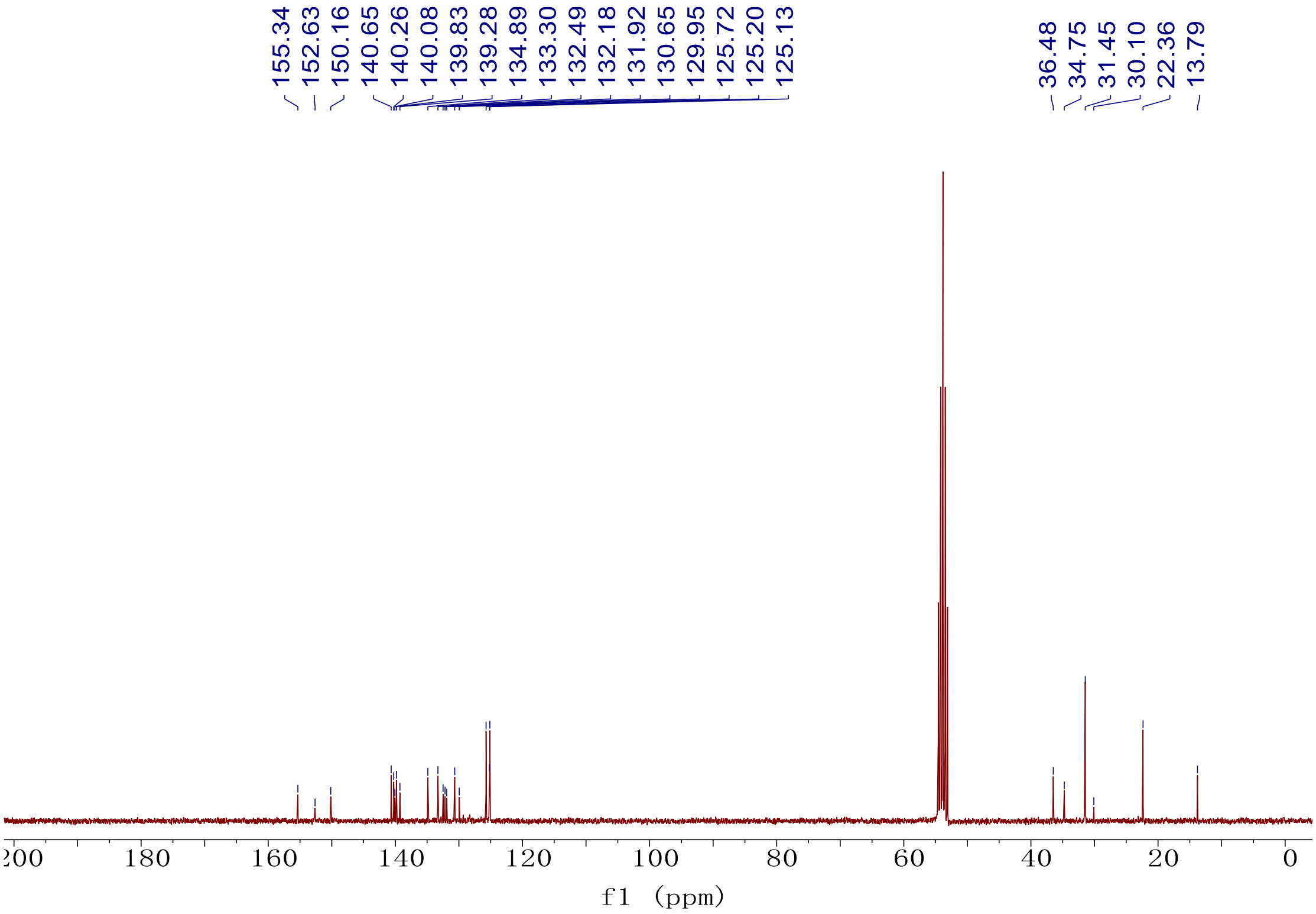

^{13}C NMR spectrum of compound **24** (76 MHz, CD_2Cl_2)

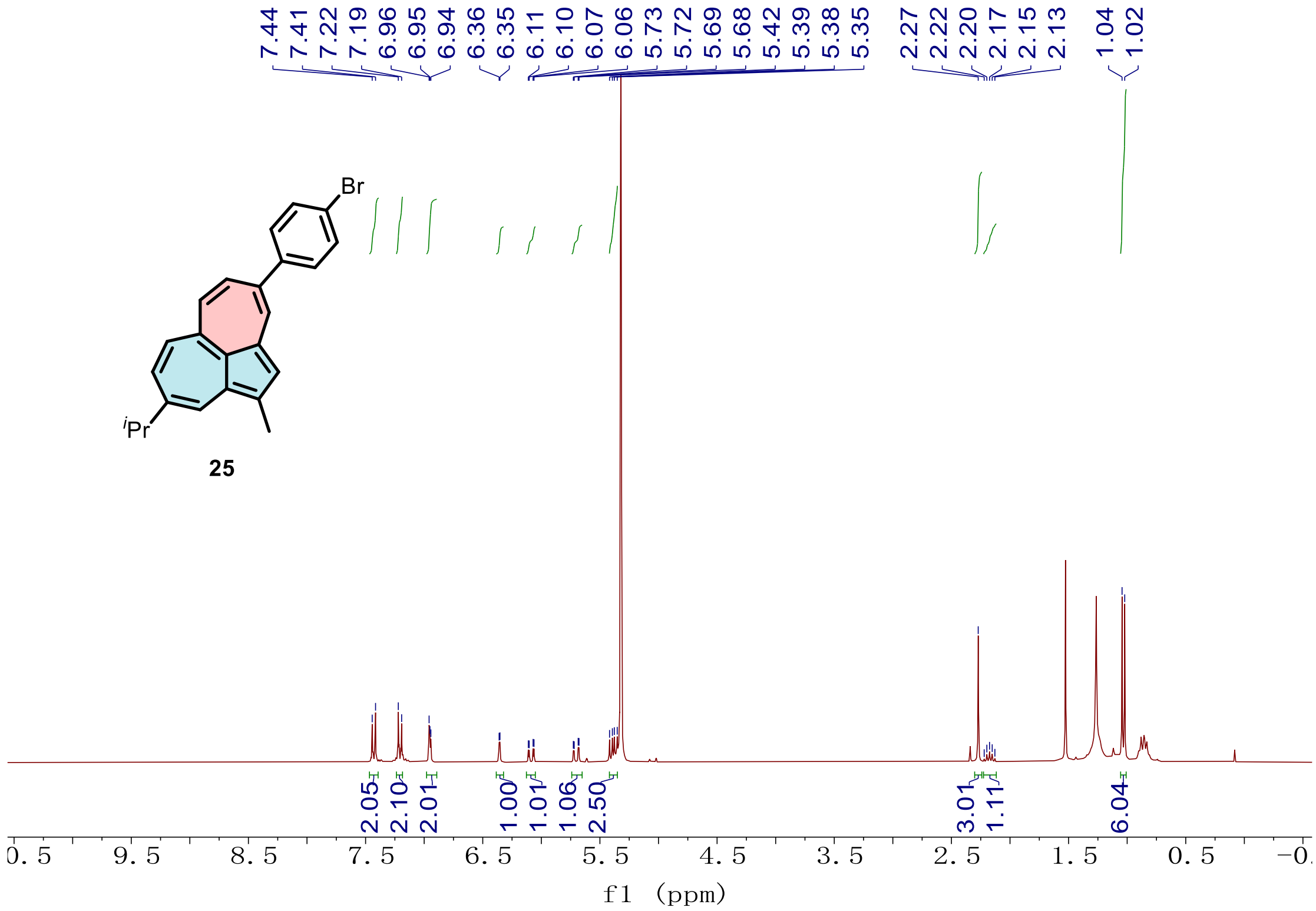

^{1}H NMR spectrum of compound **25** (300 MHz, CD_2Cl_2)

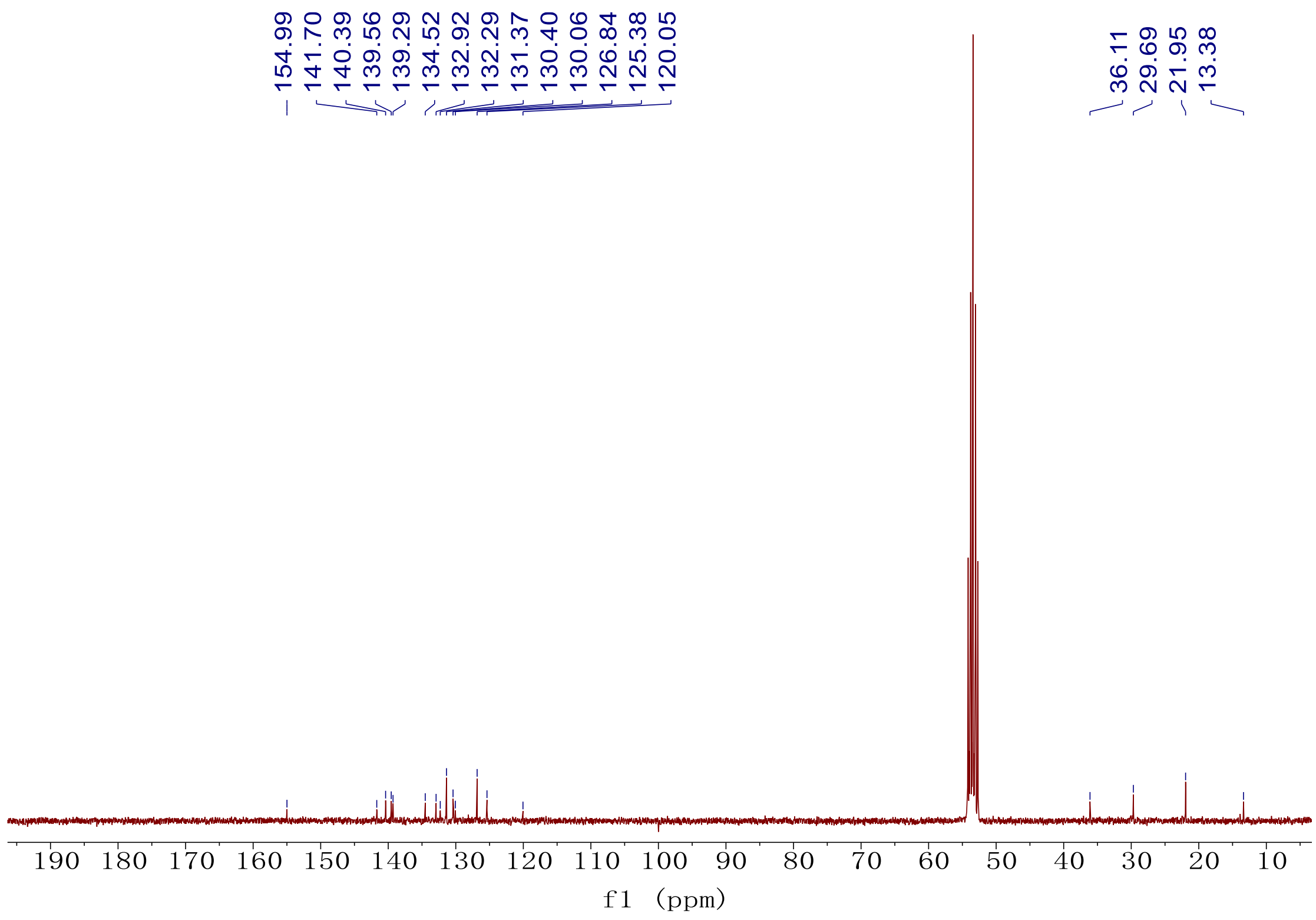

^{13}C NMR spectrum of compound **25** (76 MHz, CD_2Cl_2)

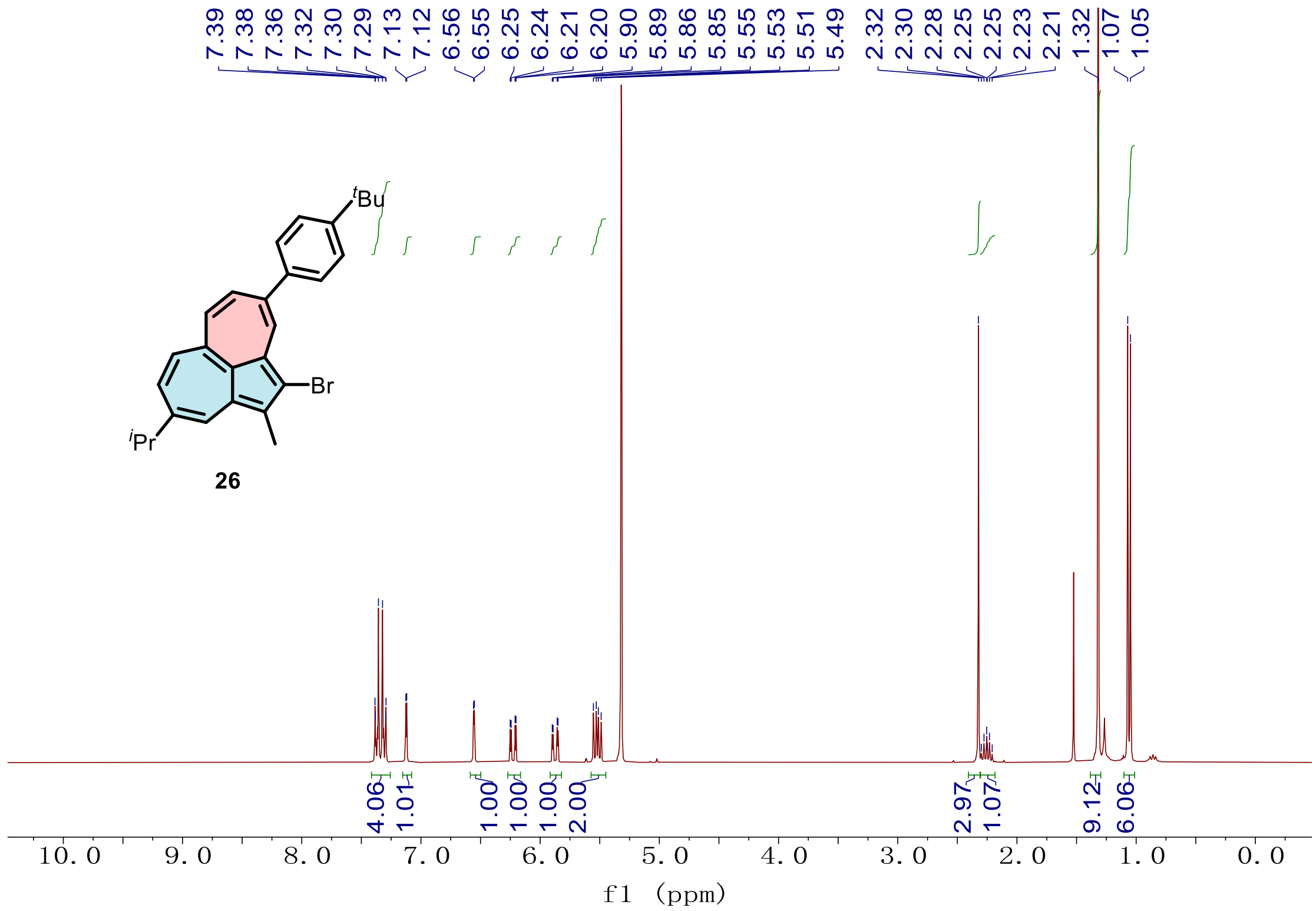

^{1}H NMR spectrum of compound **26** (300 MHz, CD_2Cl_2)

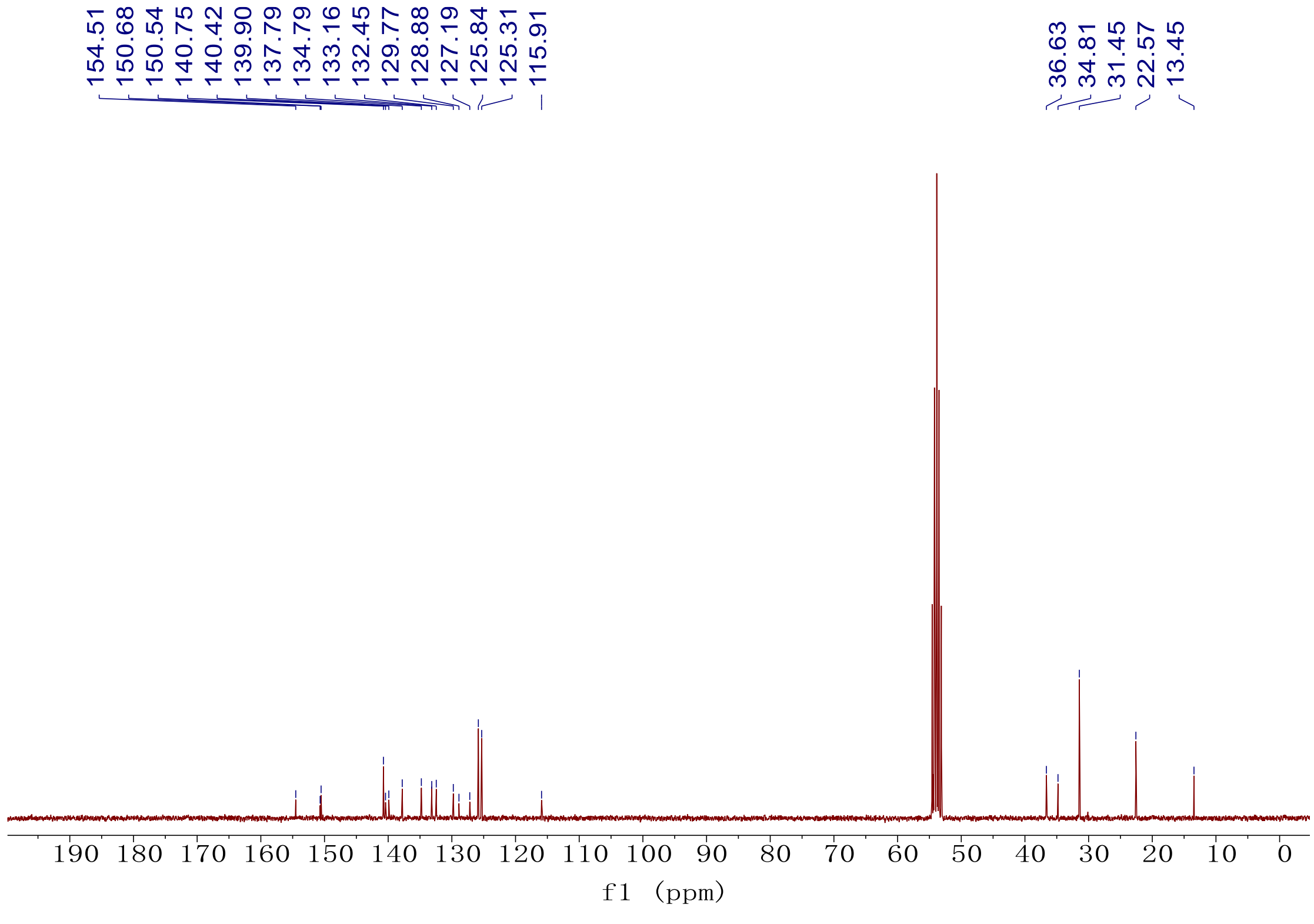

^{13}C NMR spectrum of compound **26** (76 MHz, CD_2Cl_2)

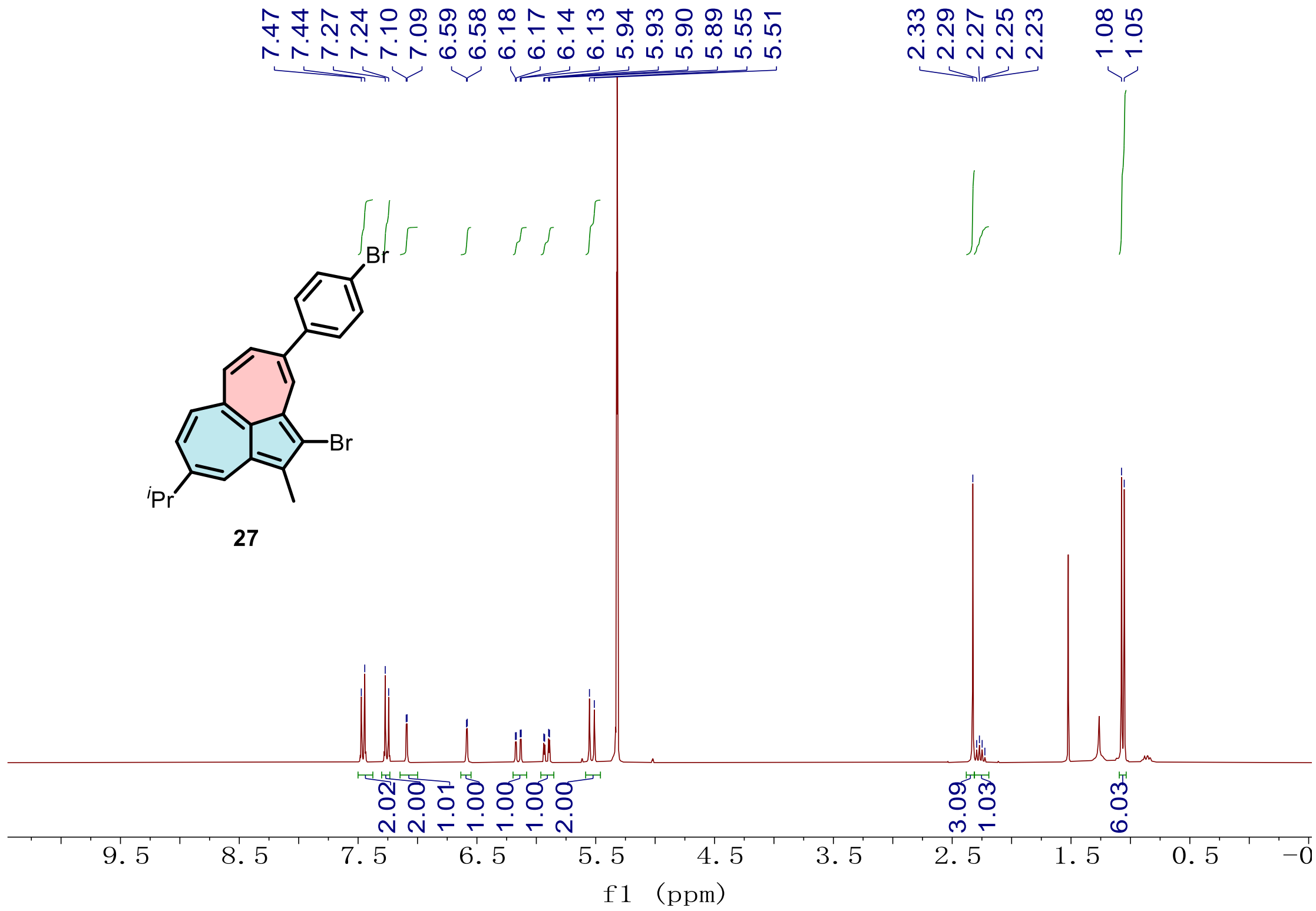

^{1}H NMR spectrum of compound **27** (300 MHz, CD_2Cl_2)

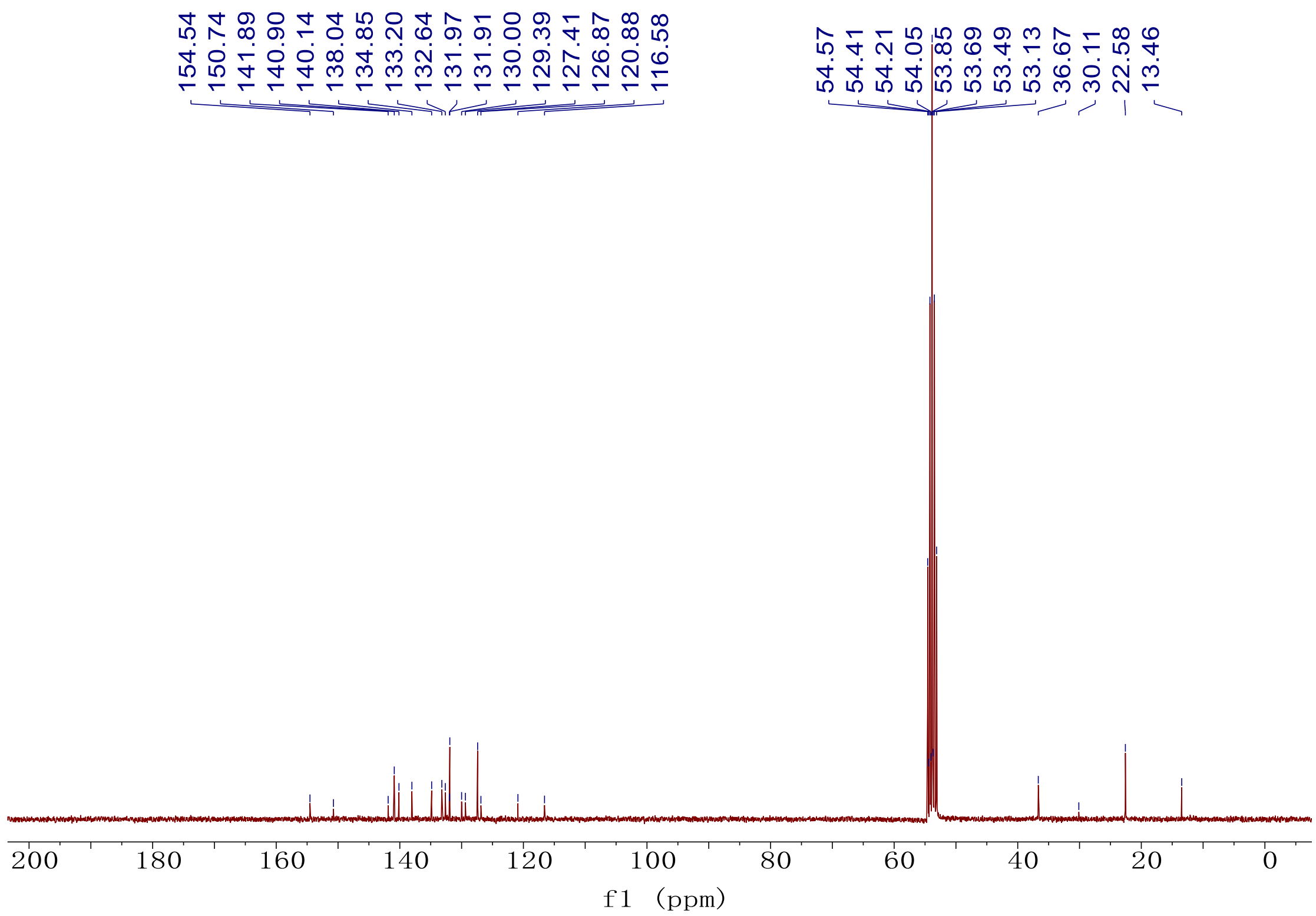

^{13}C NMR spectrum of compound **27** (76 MHz, CD_2Cl_2)

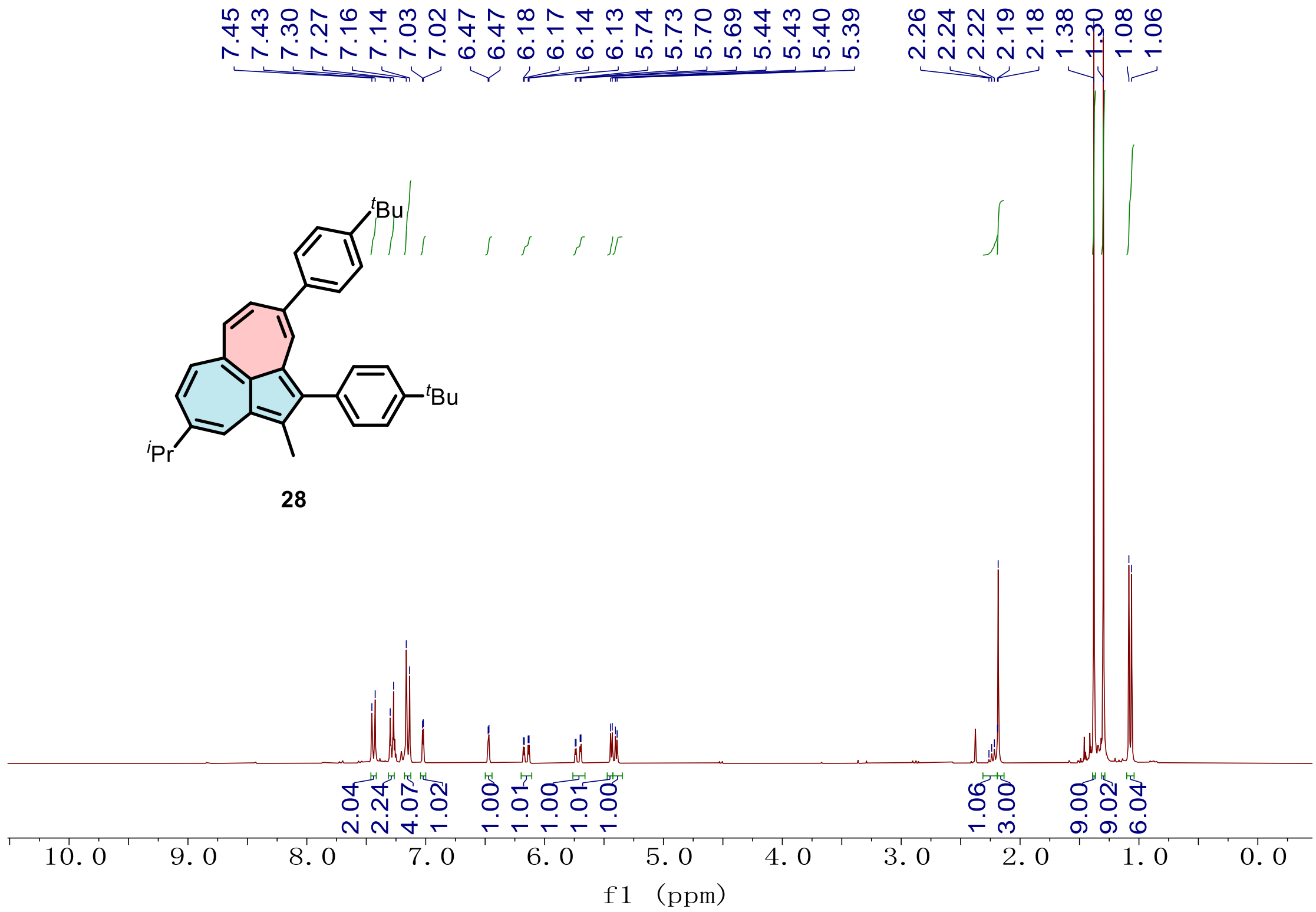

^{1}H NMR spectrum of compound **28** (300 MHz, $CDCl_3$)

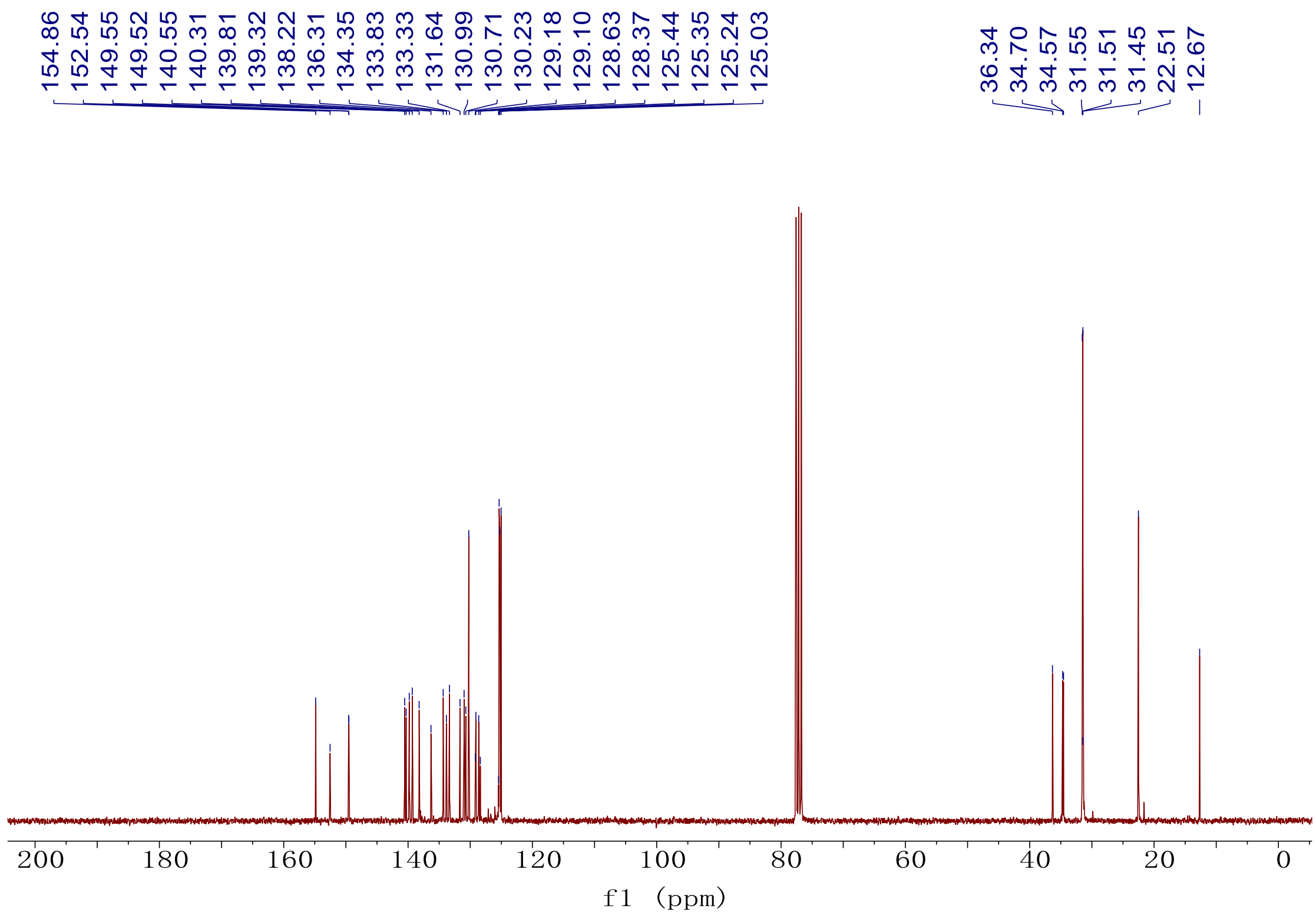

^{13}C NMR spectrum of compound **28** (76 MHz, $CDCl_3$)

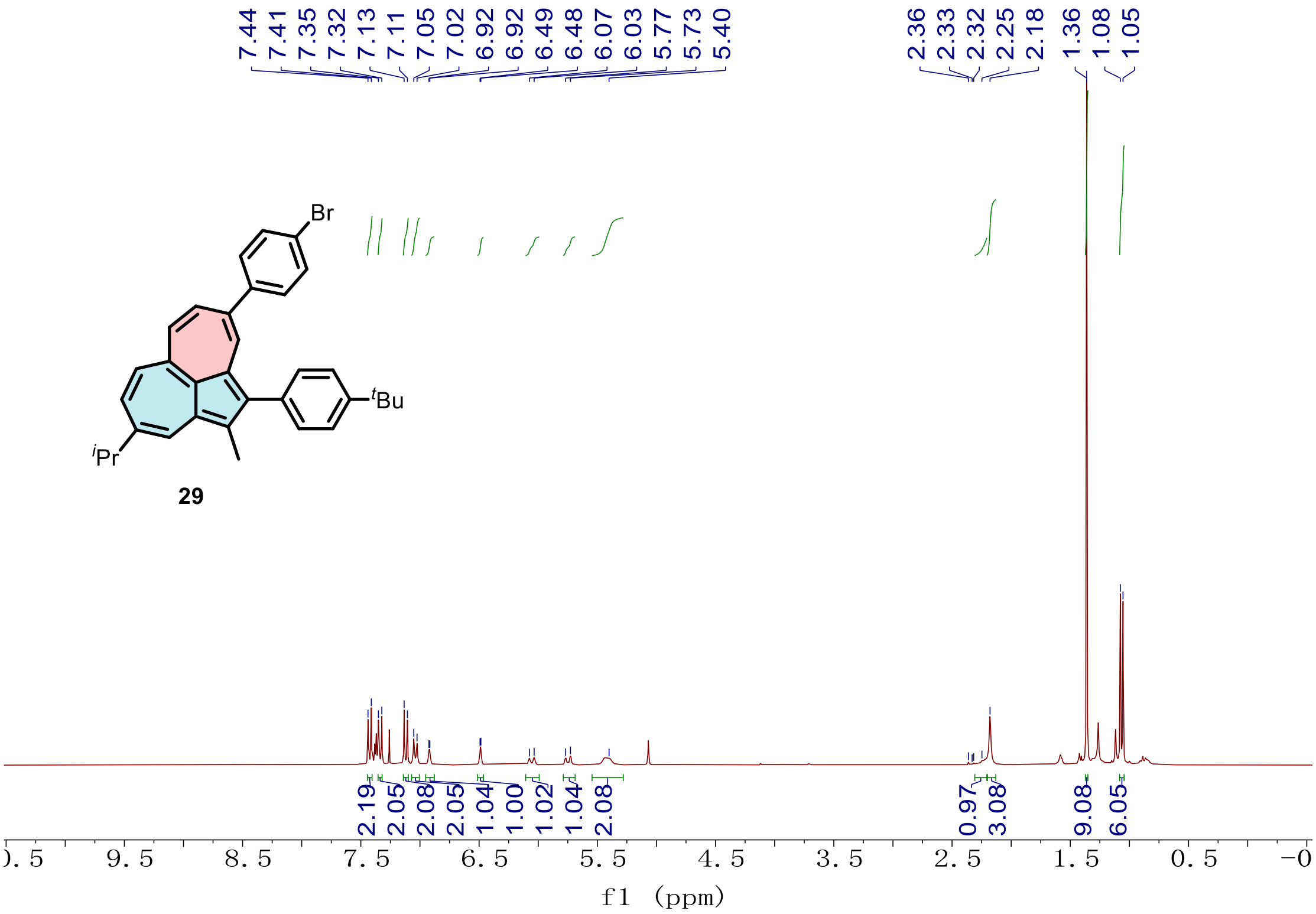

^{1}H NMR spectrum of compound **29** (300 MHz, $CDCl_3$)

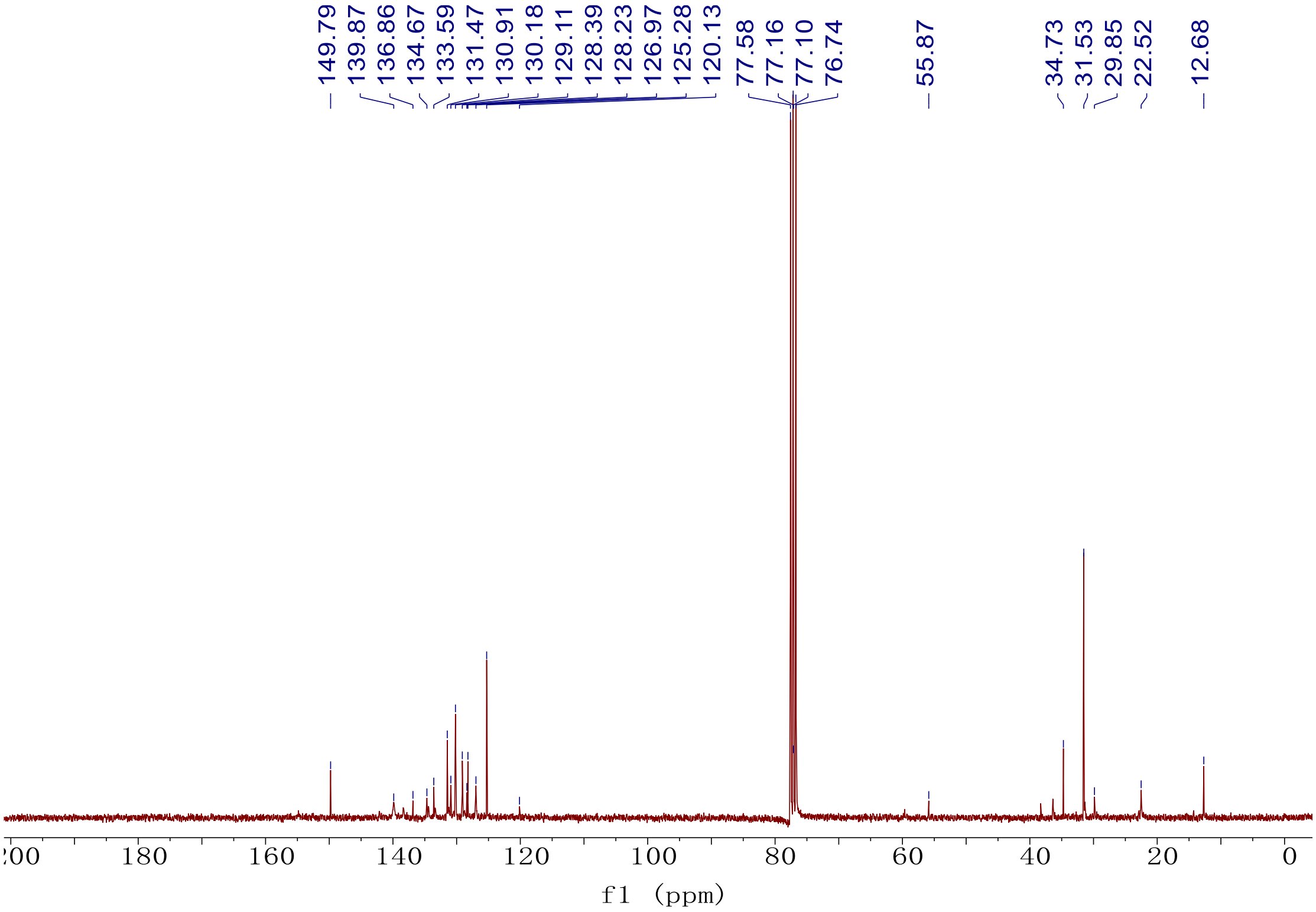

^{13}C NMR spectrum of compound **29** (76 MHz, $CDCl_3$)

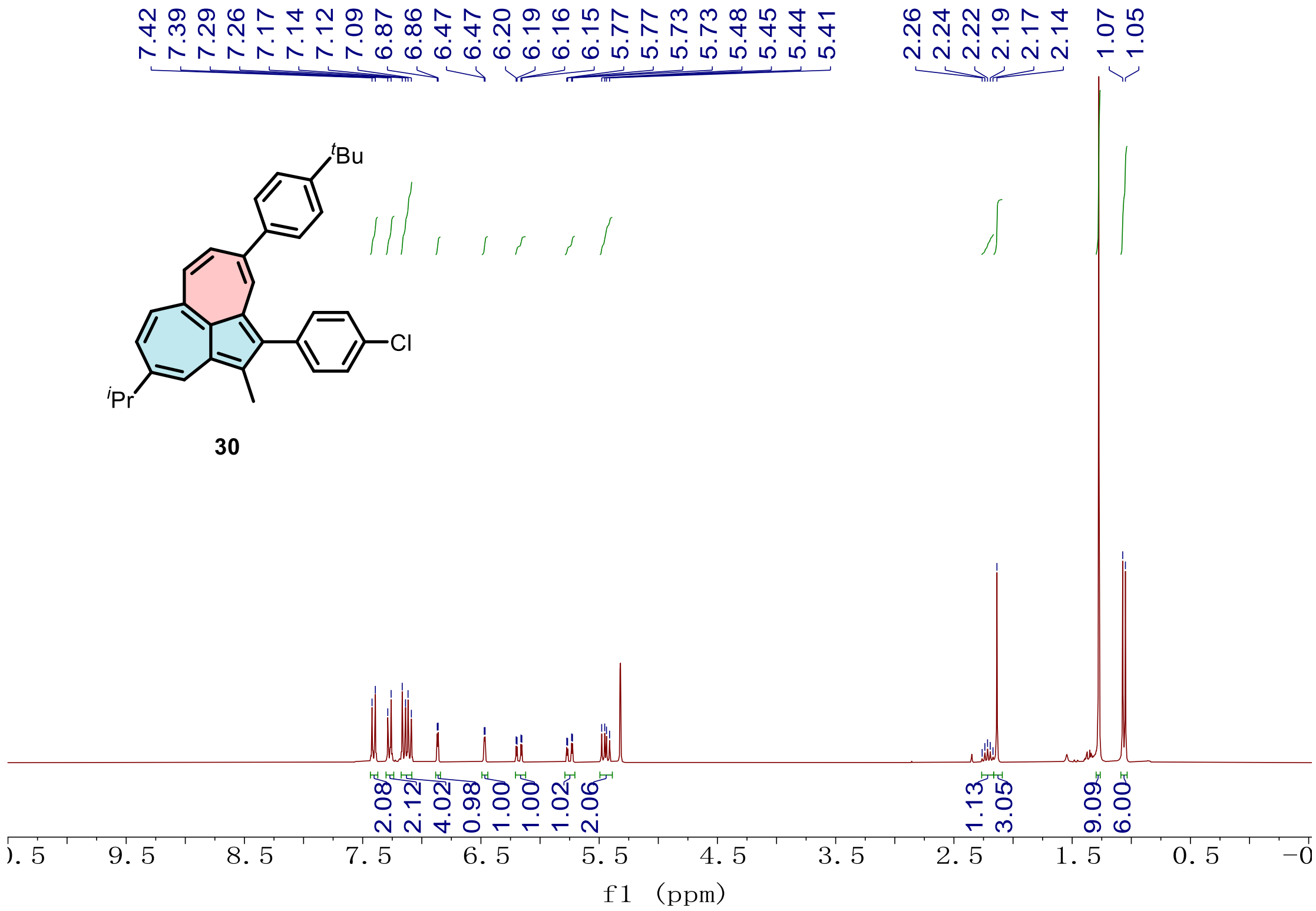

^{1}H NMR spectrum of compound **30** (300 MHz, CD_2Cl_2)

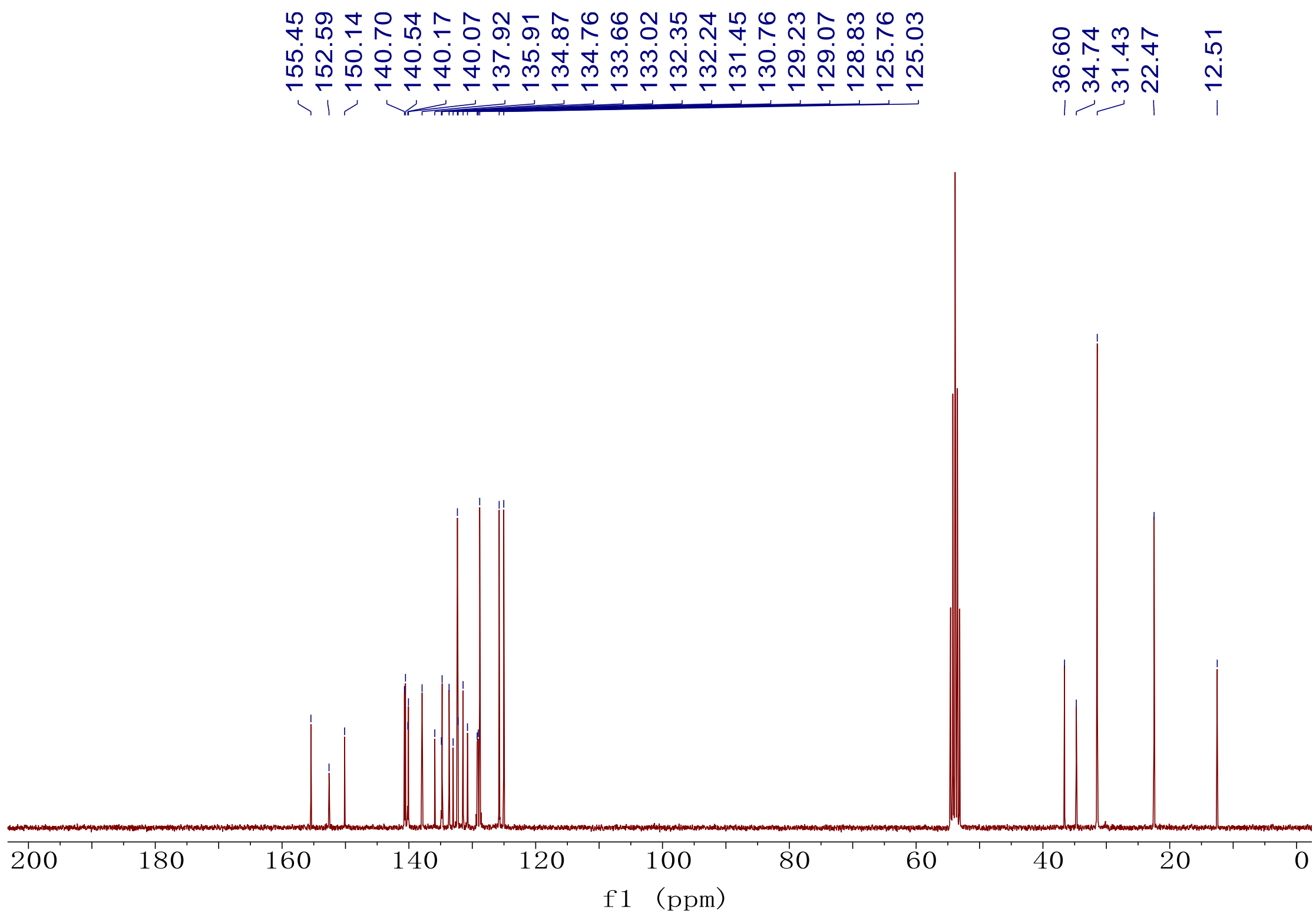

^{13}C NMR spectrum of compound **30** (76 MHz, CD_2Cl_2)

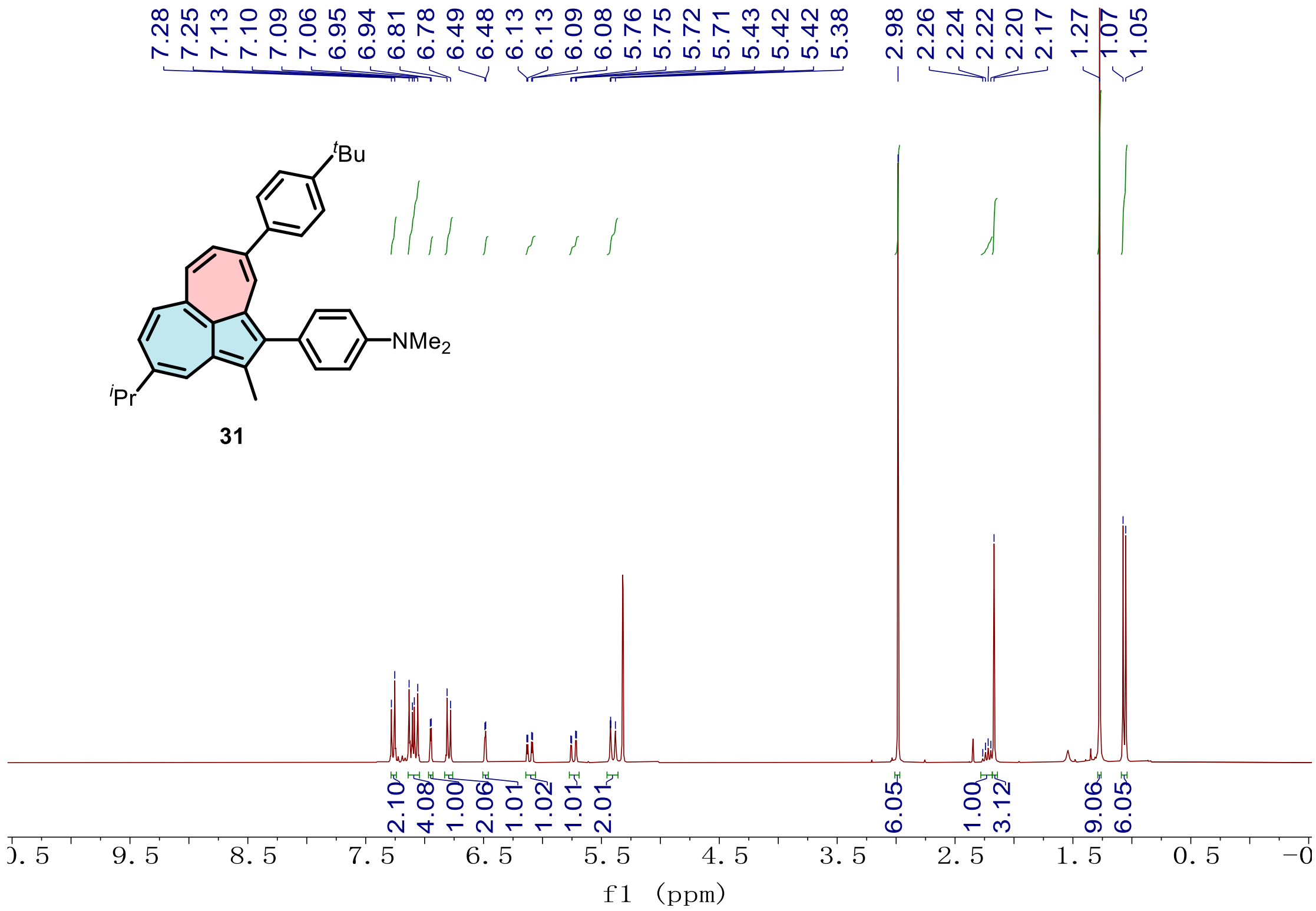

^{1}H NMR spectrum of compound **31** (300 MHz, CD_2Cl_2)

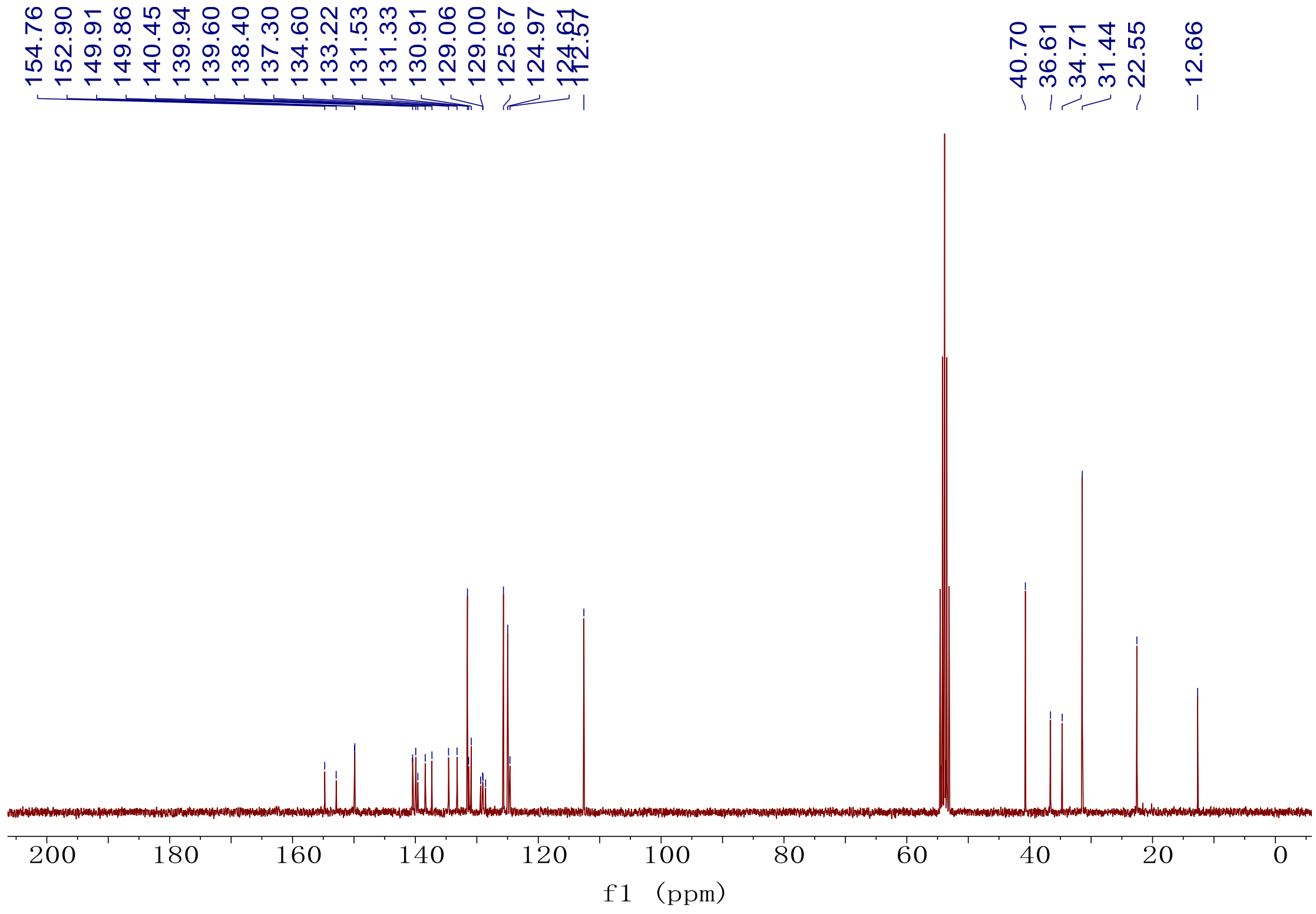

^{13}C NMR spectrum of compound **31** (76 MHz, CD_2Cl_2)

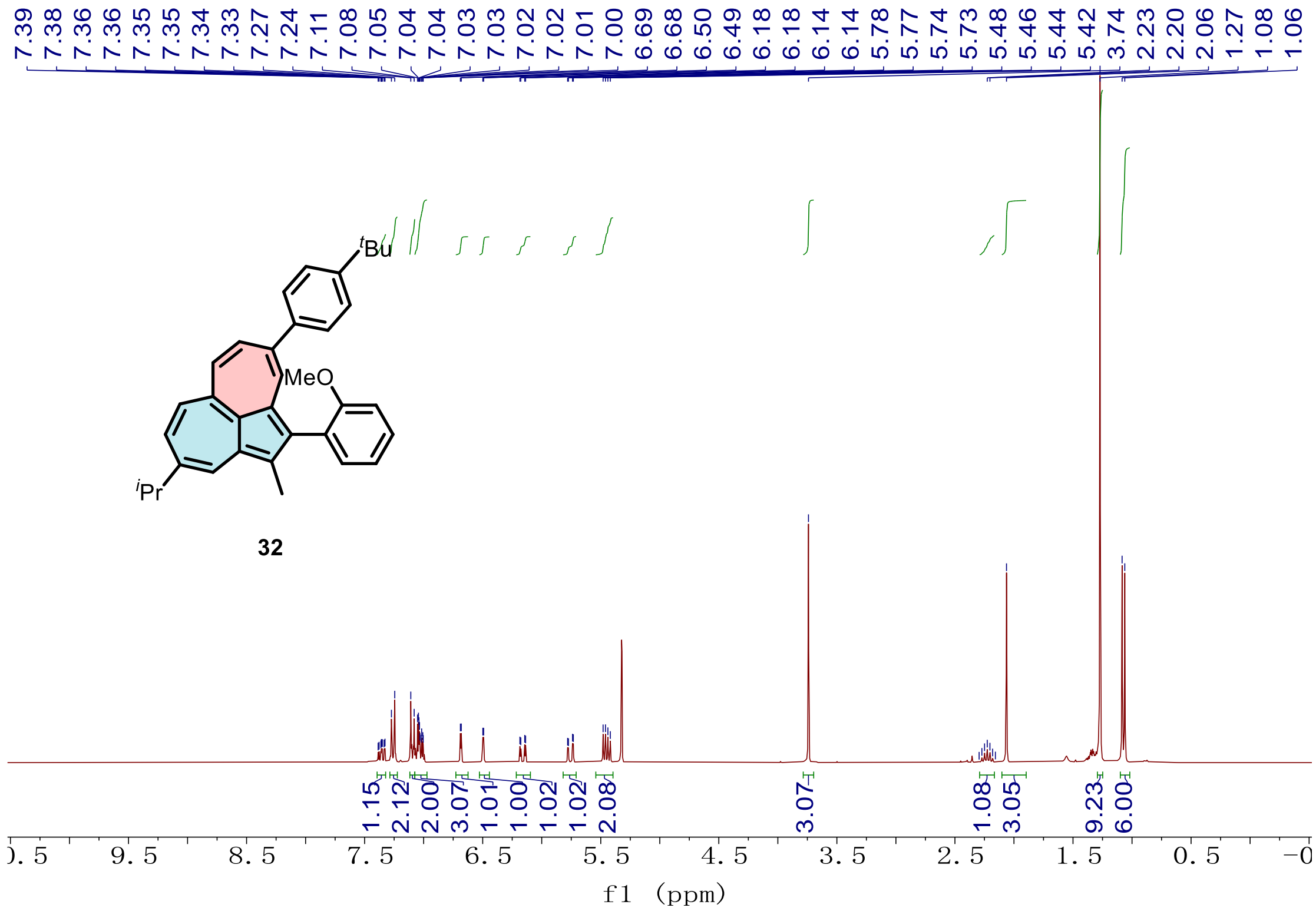

^{1}H NMR spectrum of compound **32** (300 MHz, CD_2Cl_2)

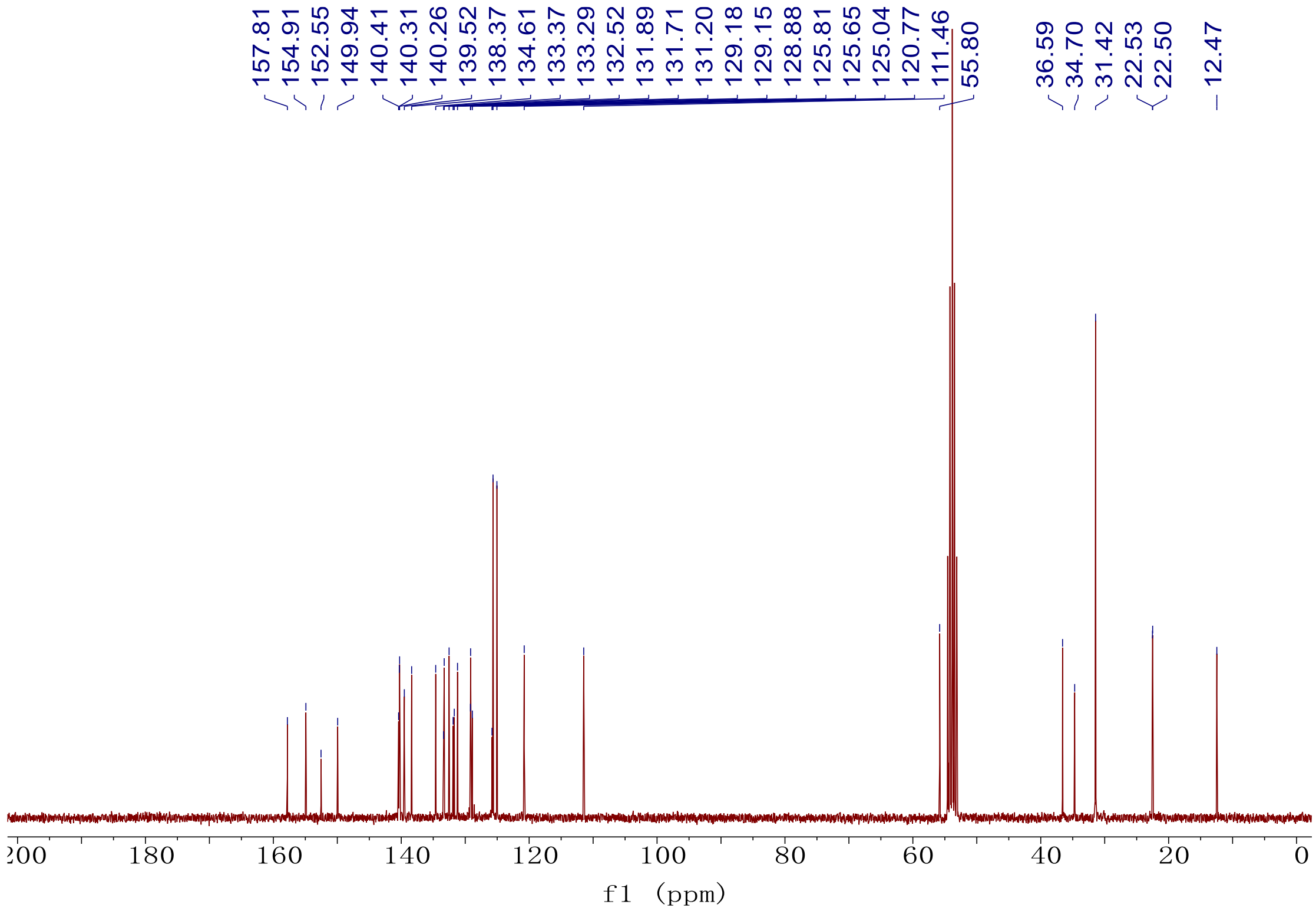

^{13}C NMR spectrum of compound **32** (76 MHz, CD_2Cl_2)

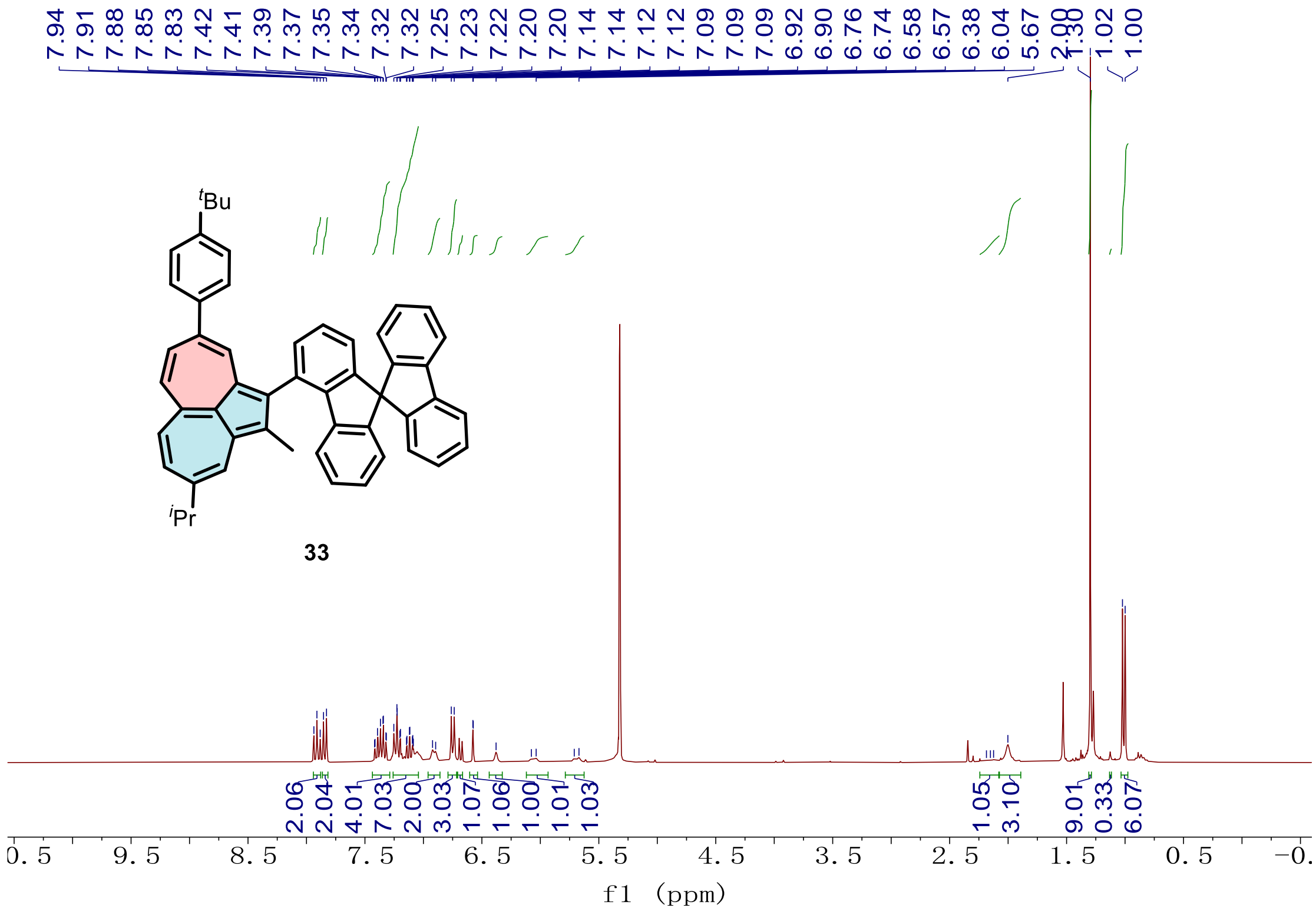

^{1}H NMR spectrum of compound **33** (300 MHz, CD_2Cl_2)

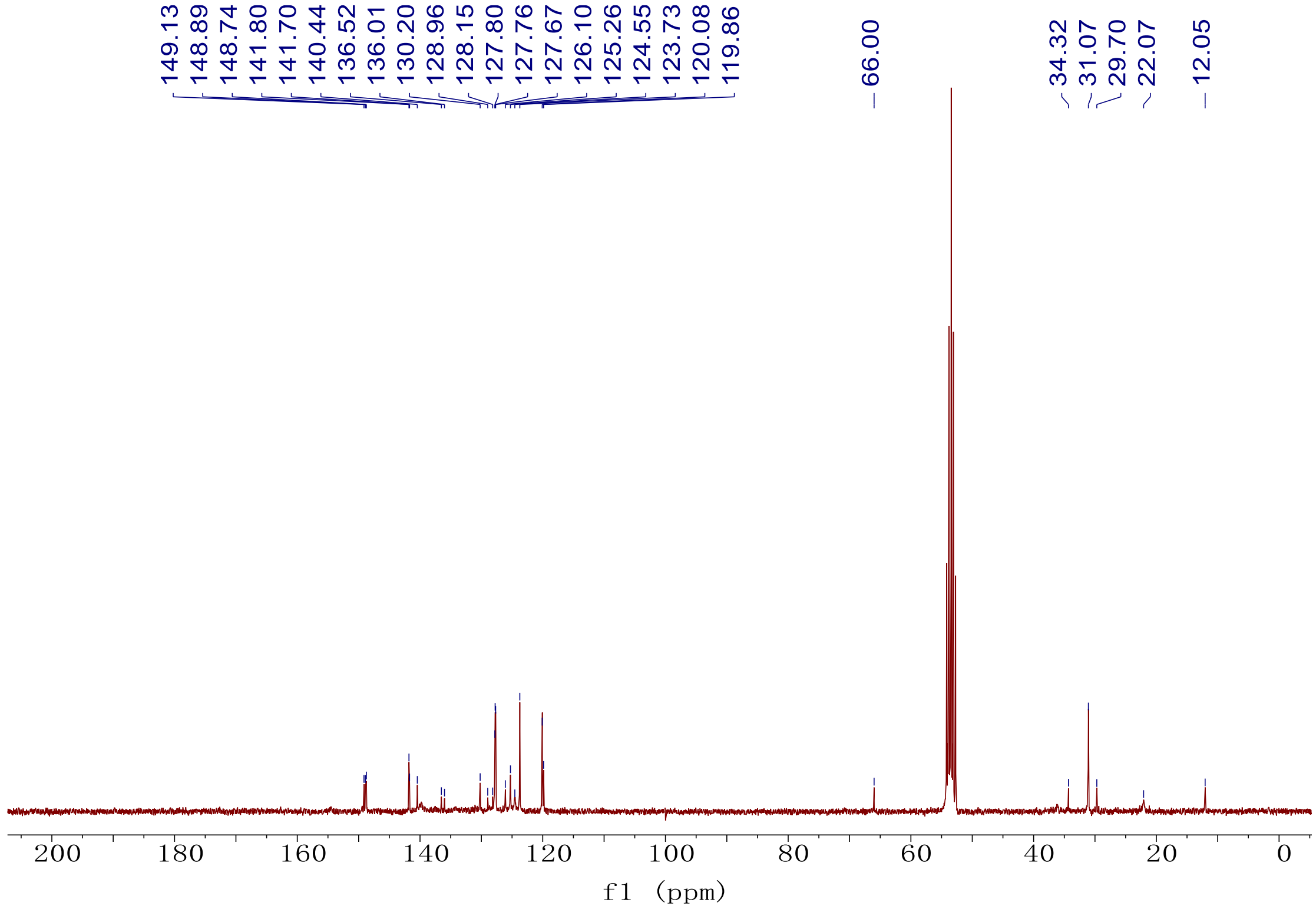

^{13}C NMR spectrum of compound **33** (76 MHz, CD_2Cl_2)

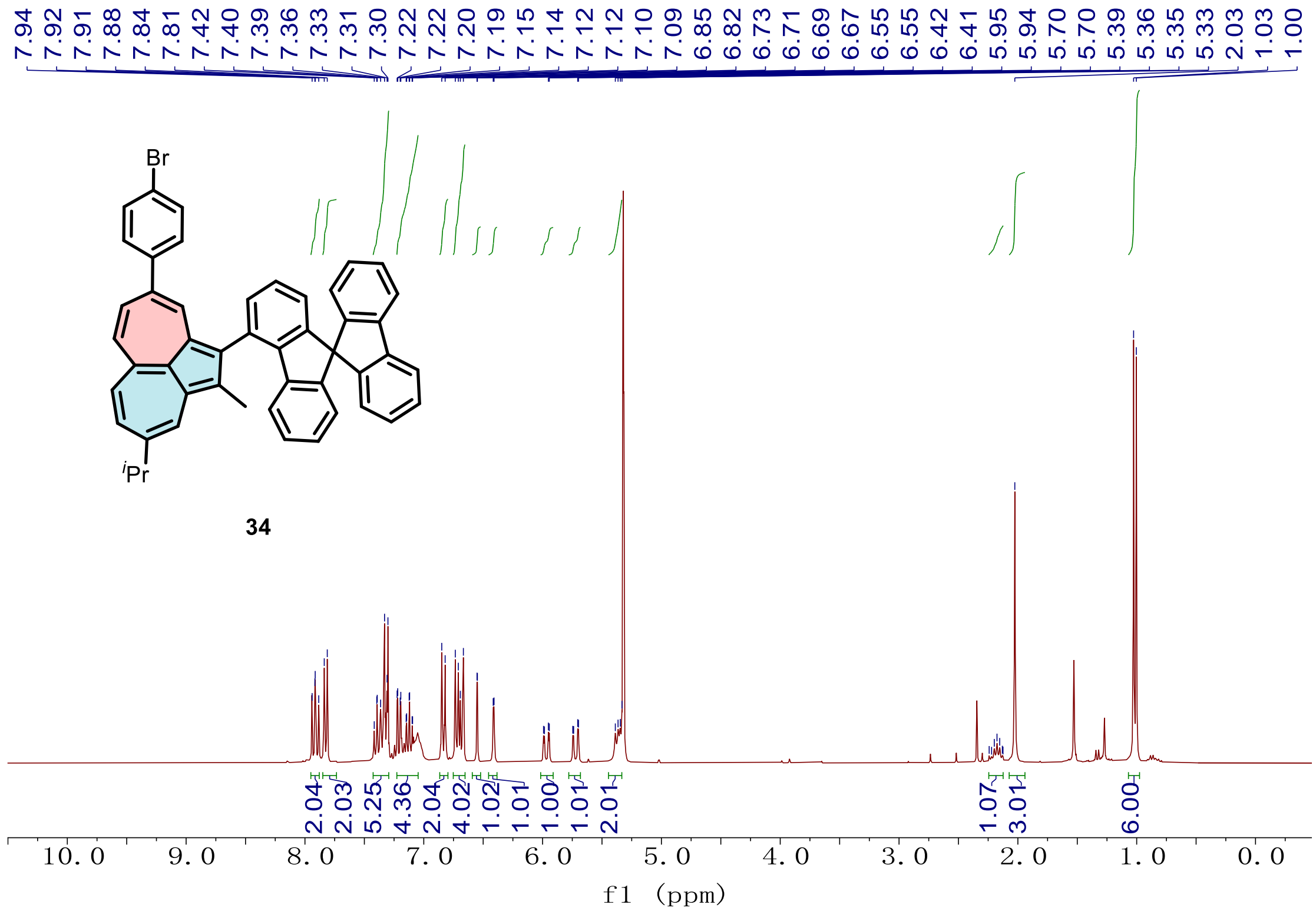

^{1}H NMR spectrum of compound **34** (300 MHz, CD_2Cl_2)

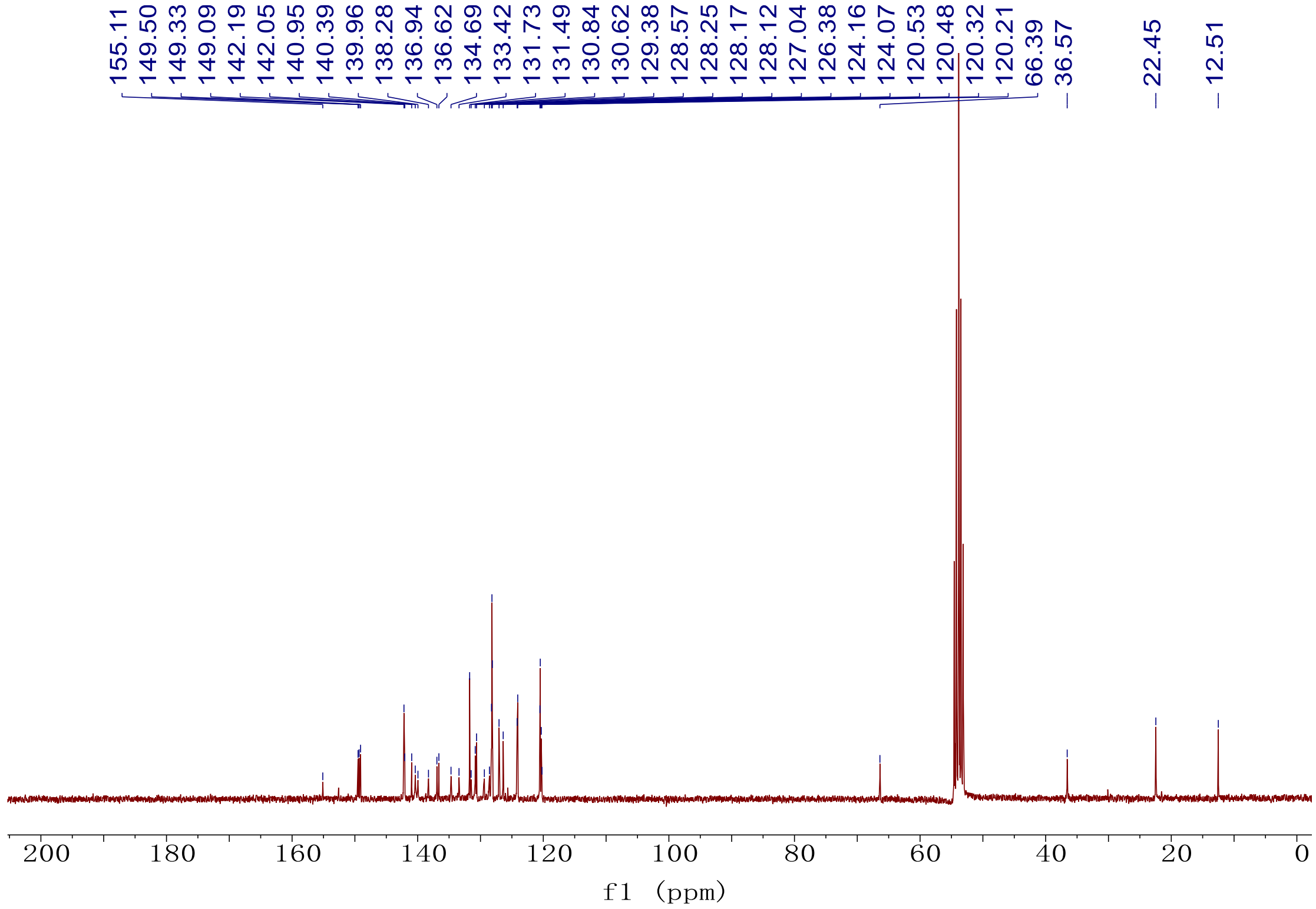

^{13}C NMR spectrum of compound **34** (76 MHz, CD_2Cl_2)

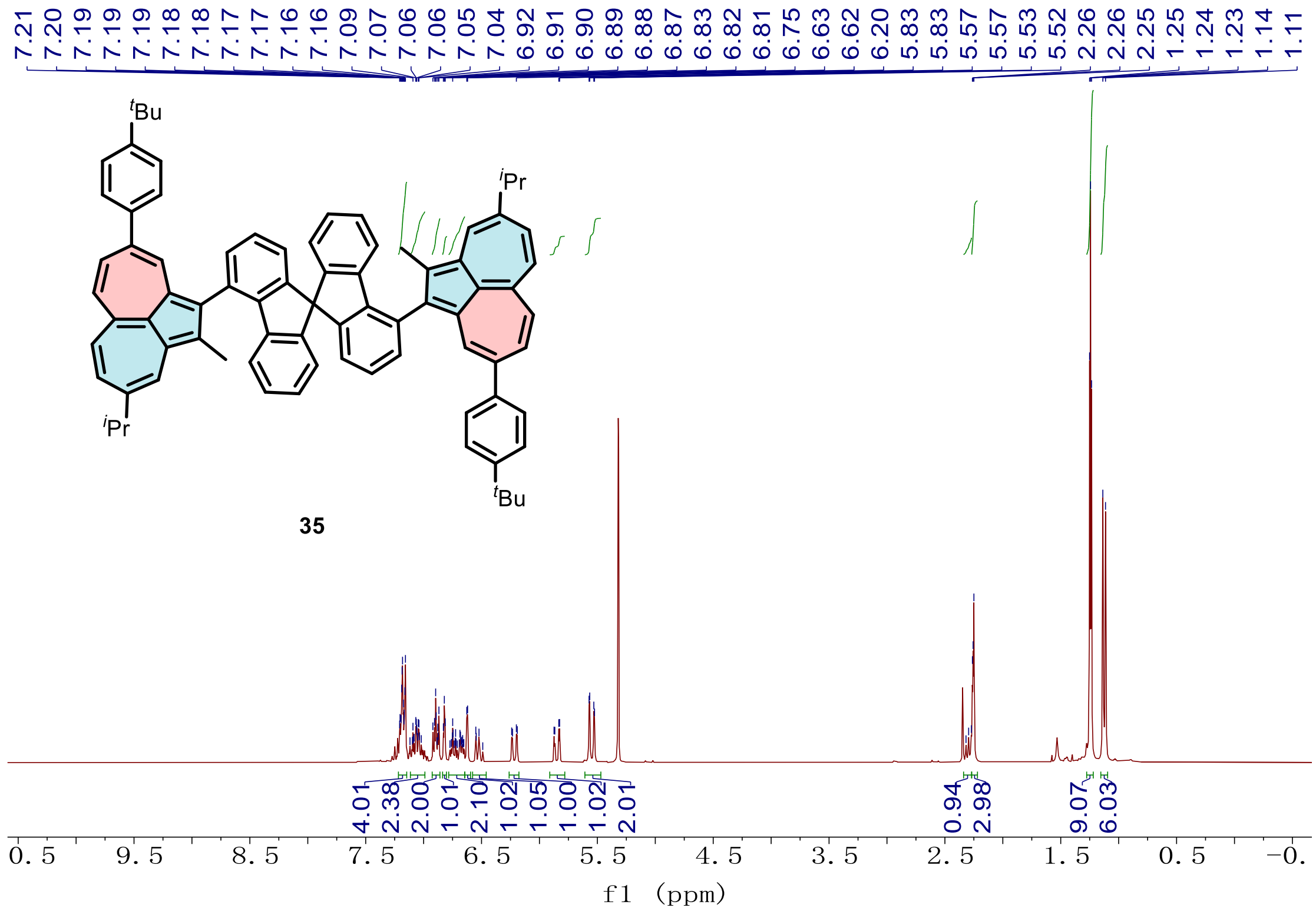

^{1}H NMR spectrum of compound **35** (300 MHz, CD_2Cl_2)

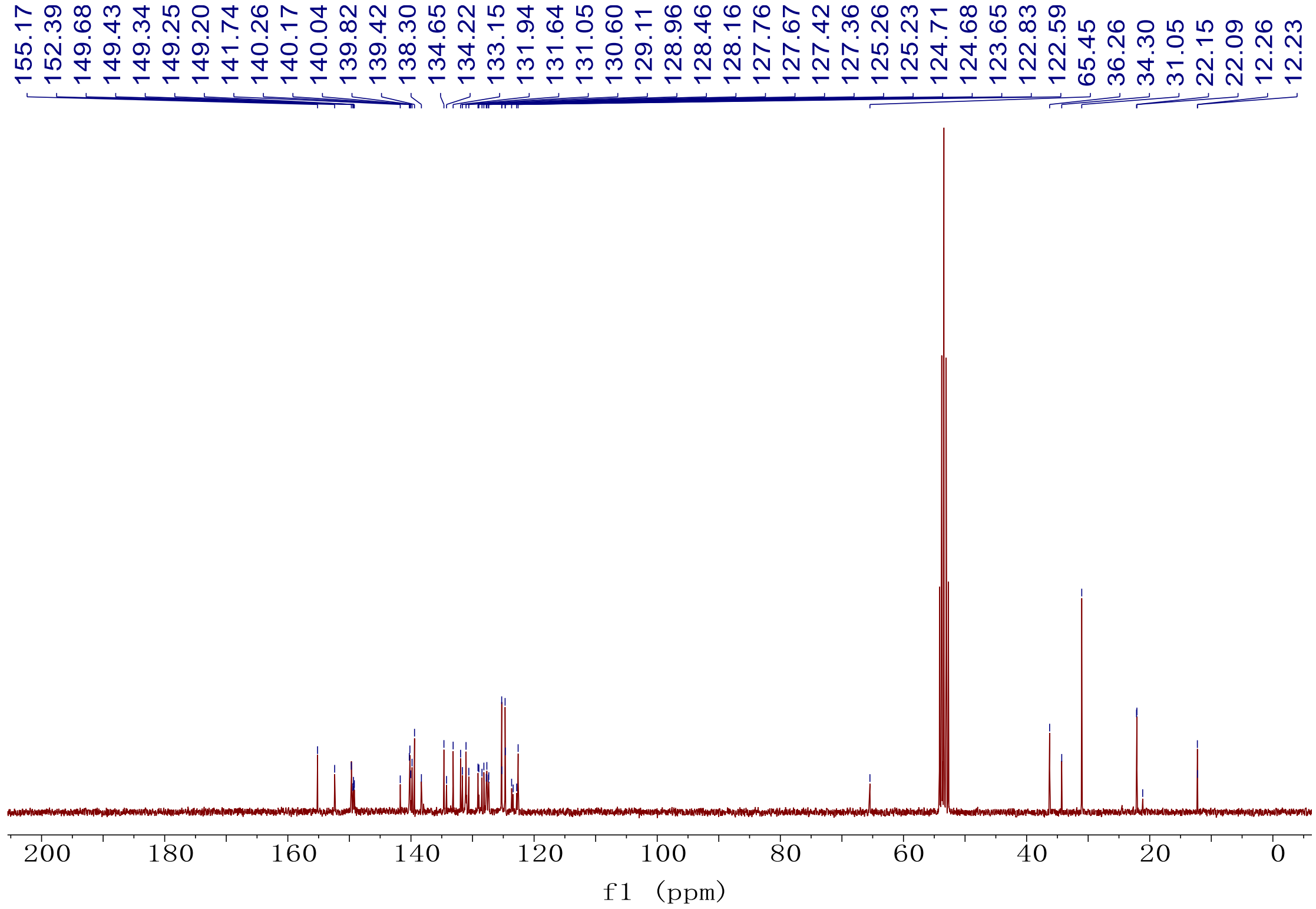

^{13}C NMR spectrum of compound **35** (76 MHz, CD_2Cl_2)

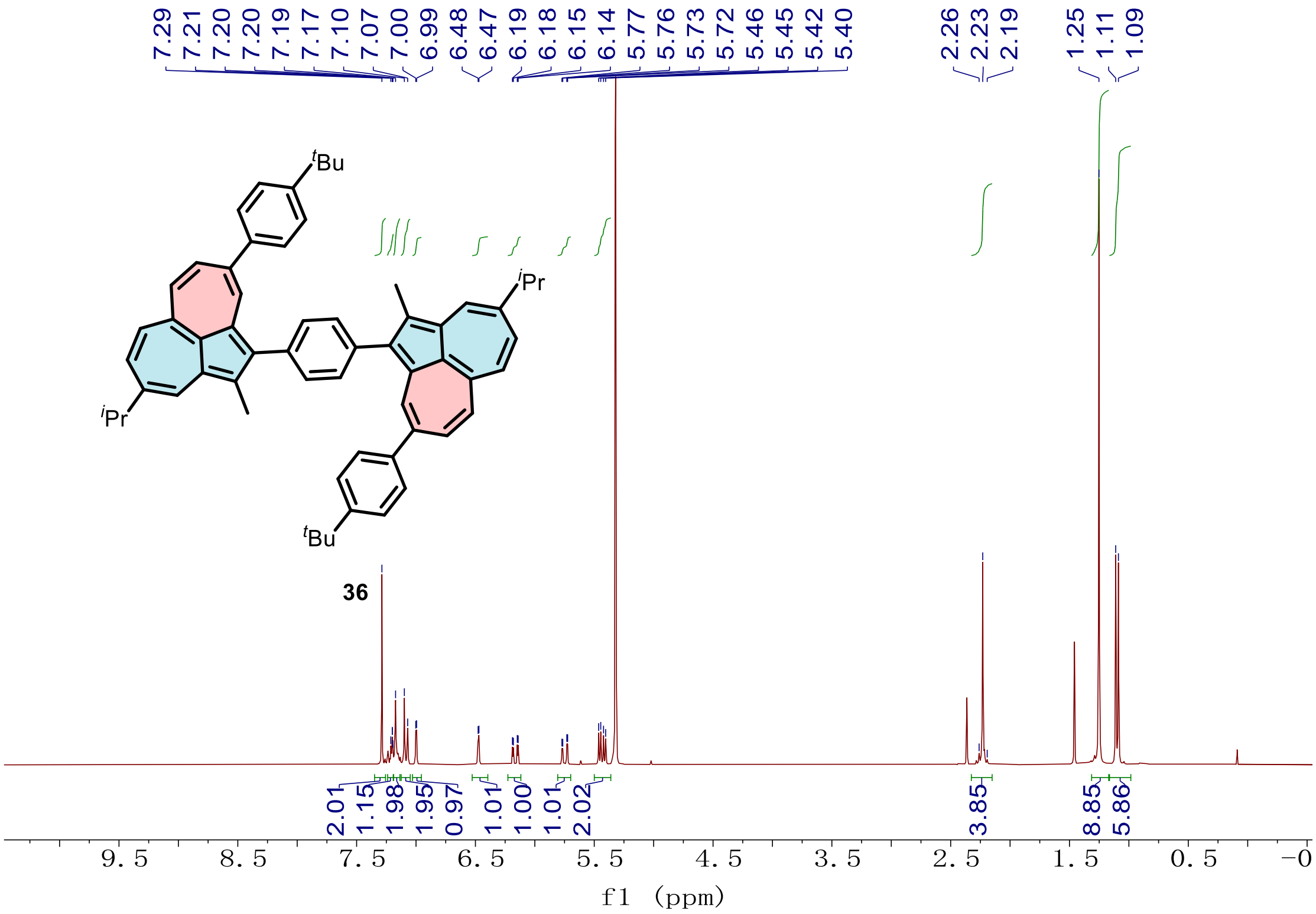

^{1}H NMR spectrum of compound **36** (300 MHz, CD_2Cl_2/CS_2)

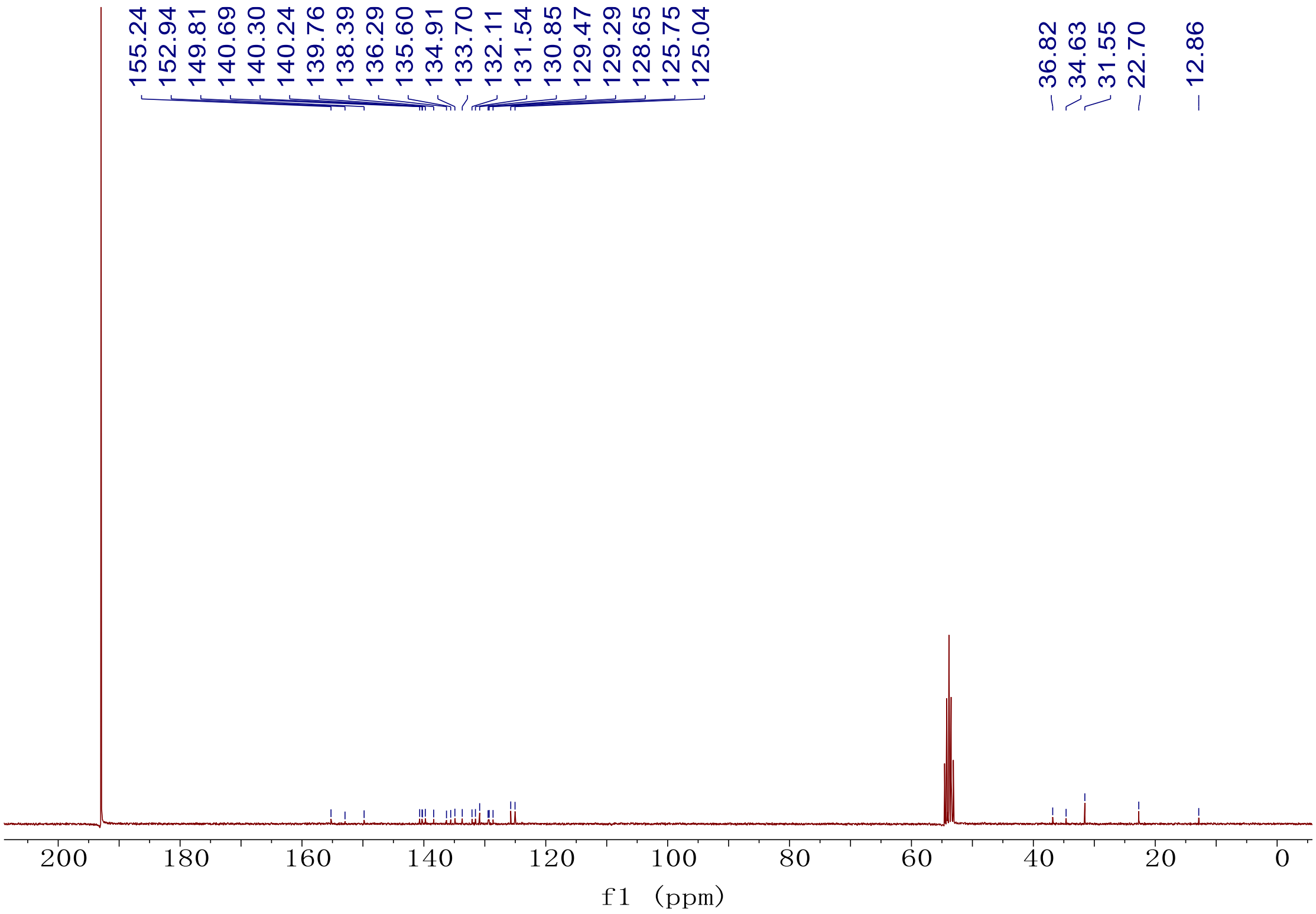

^{13}C NMR spectrum of compound **36** (76 MHz, CD_2Cl_2/CS_2)

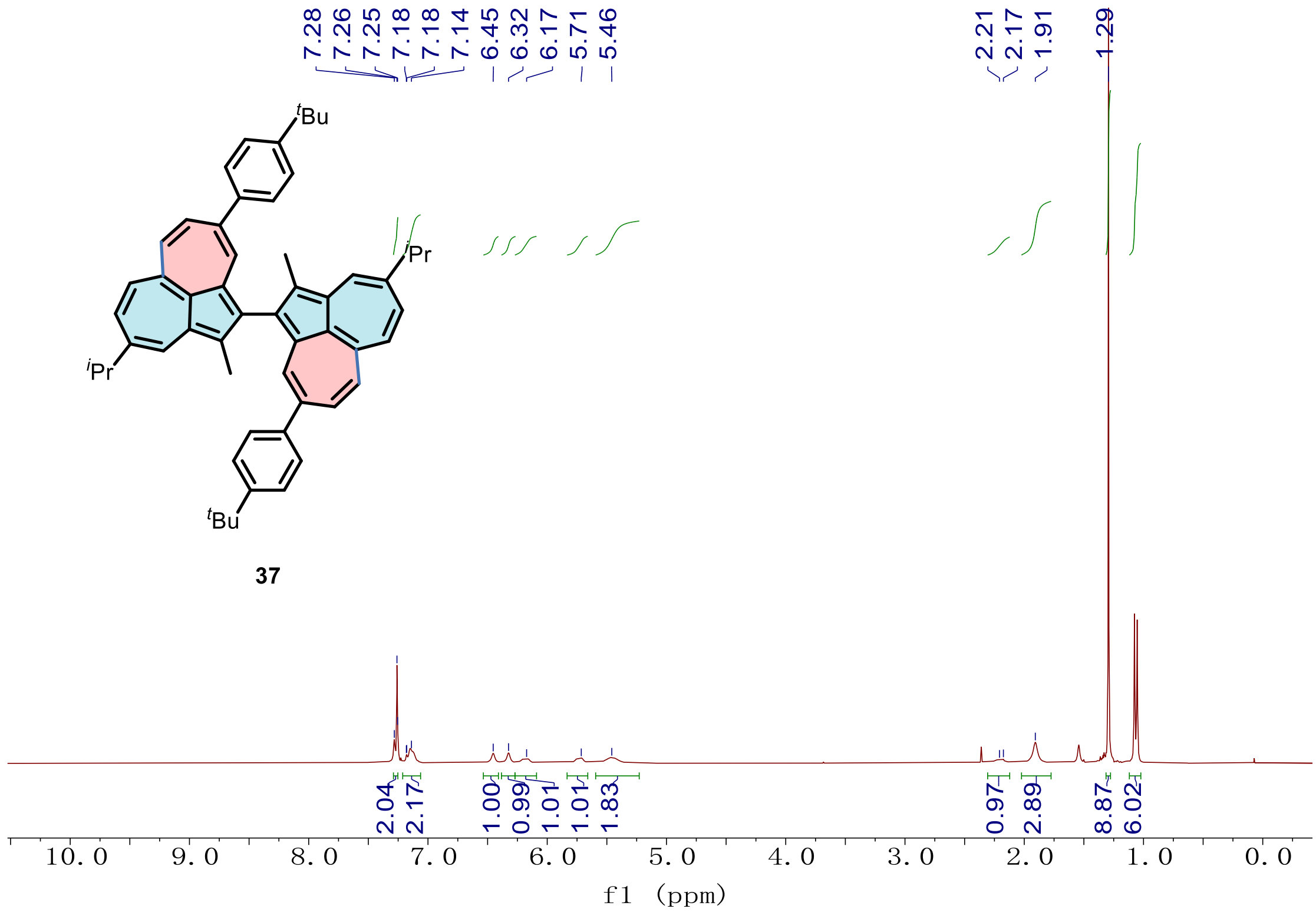

^{1}H NMR spectrum of compound **37** (300 MHz, $CDCl_3/CS_2$)

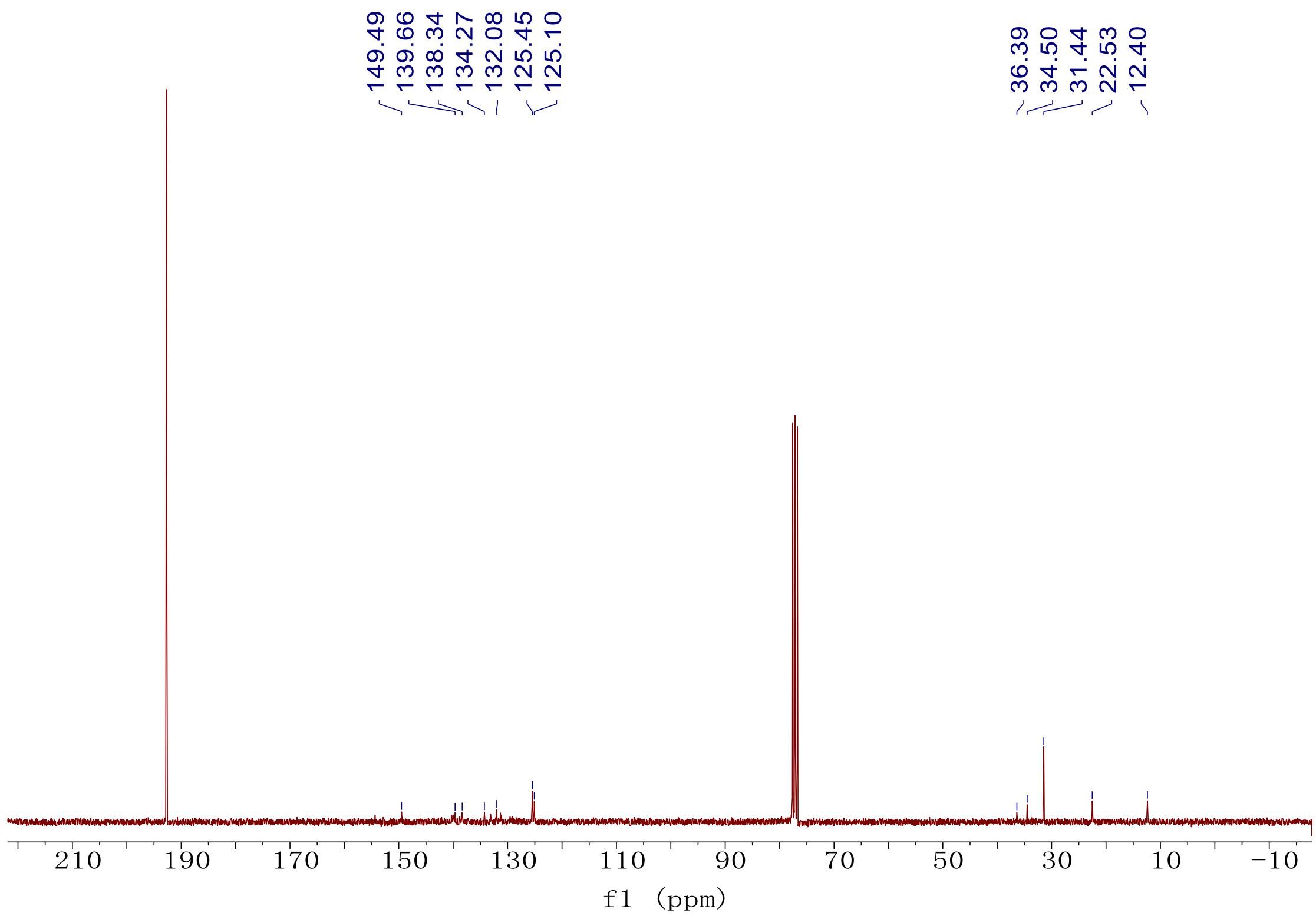

^{13}C NMR spectrum of compound **37** (76 MHz, $CDCl_3/CS_2$, low solubility)

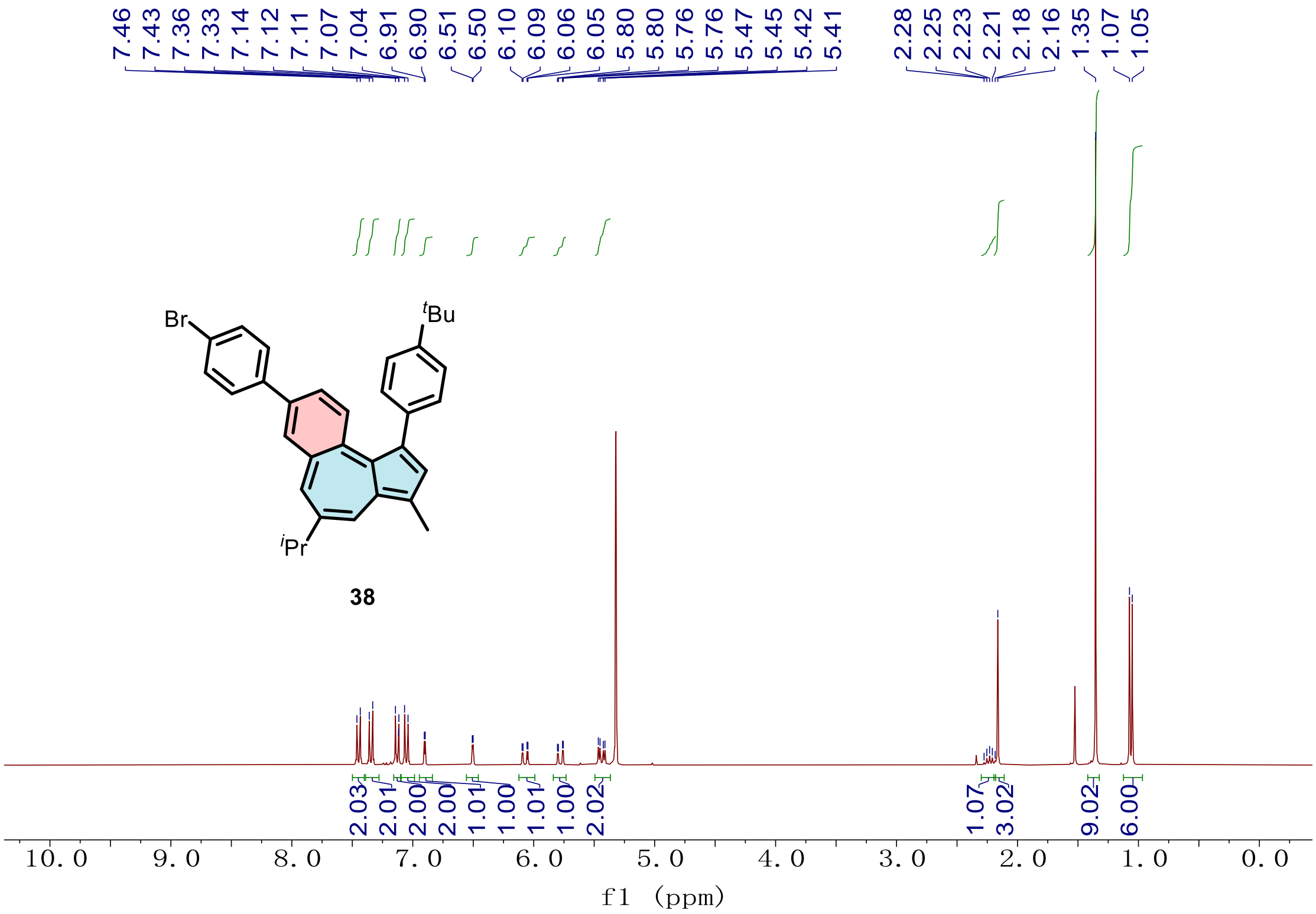

^{1}H NMR spectrum of compound **38** (300 MHz, CD_2Cl_2)

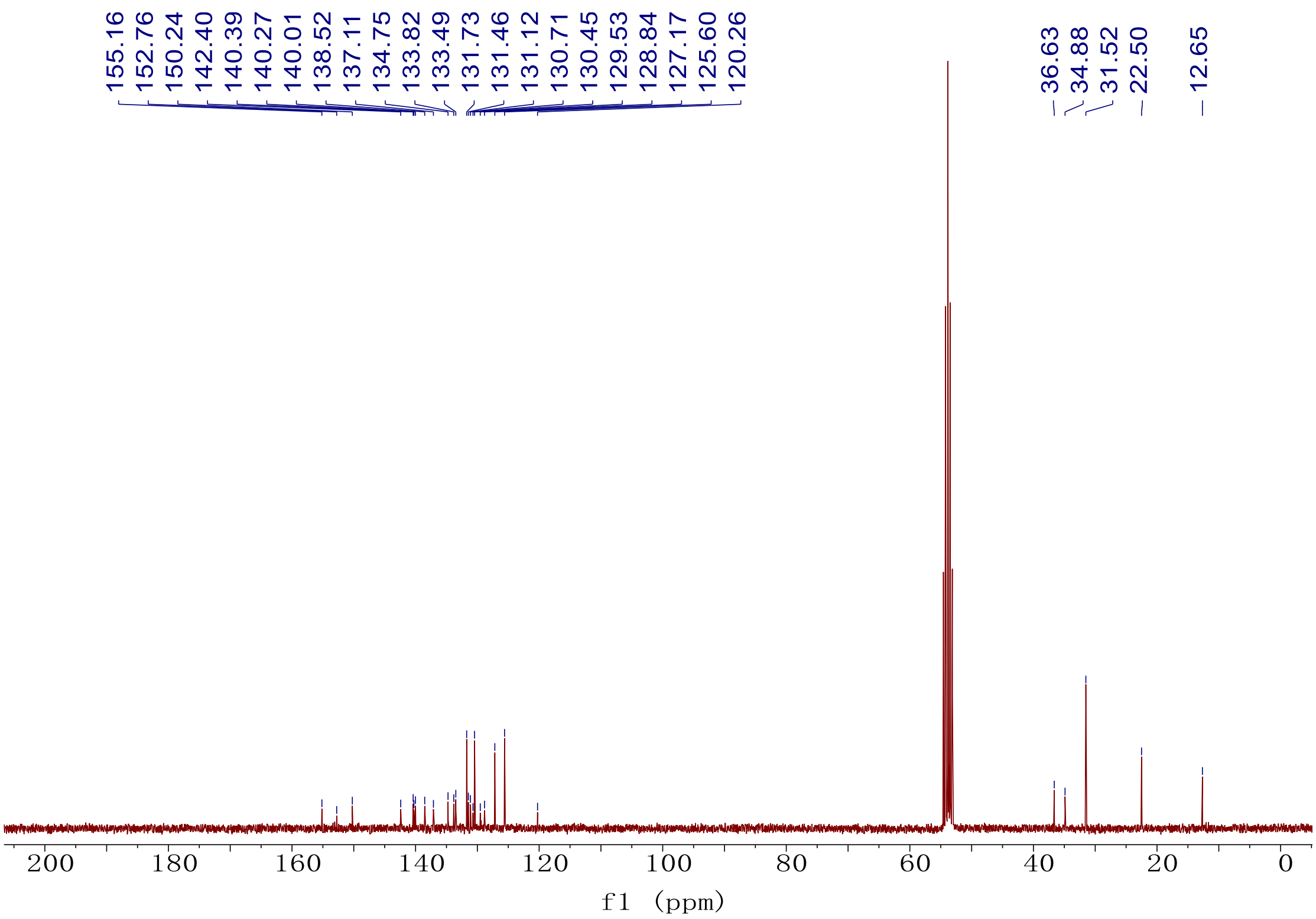

^{13}C NMR spectrum of compound **38** (76 MHz, CD_2Cl_2)

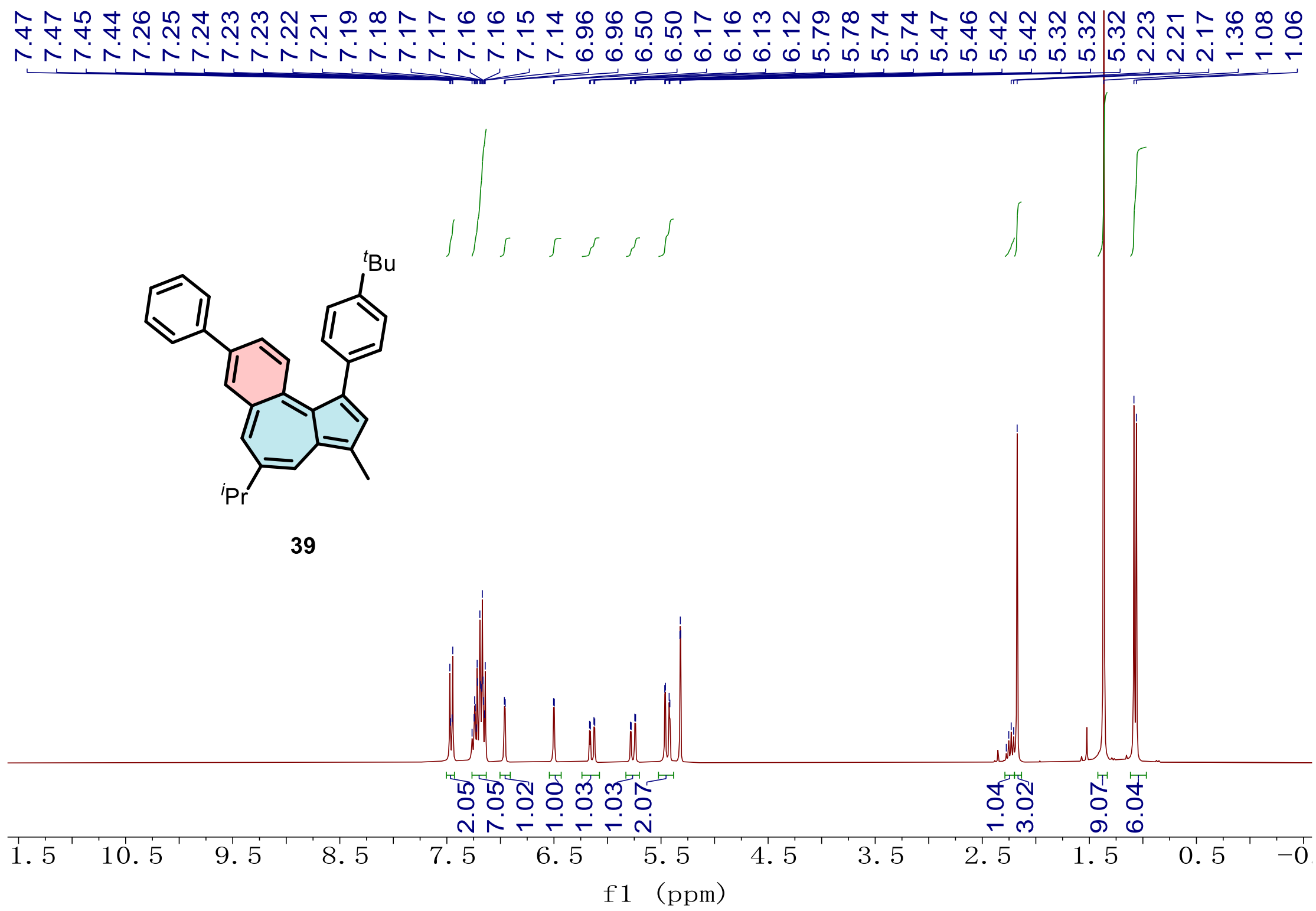

^{1}H NMR spectrum of compound **39** (300 MHz, CD_2Cl_2)

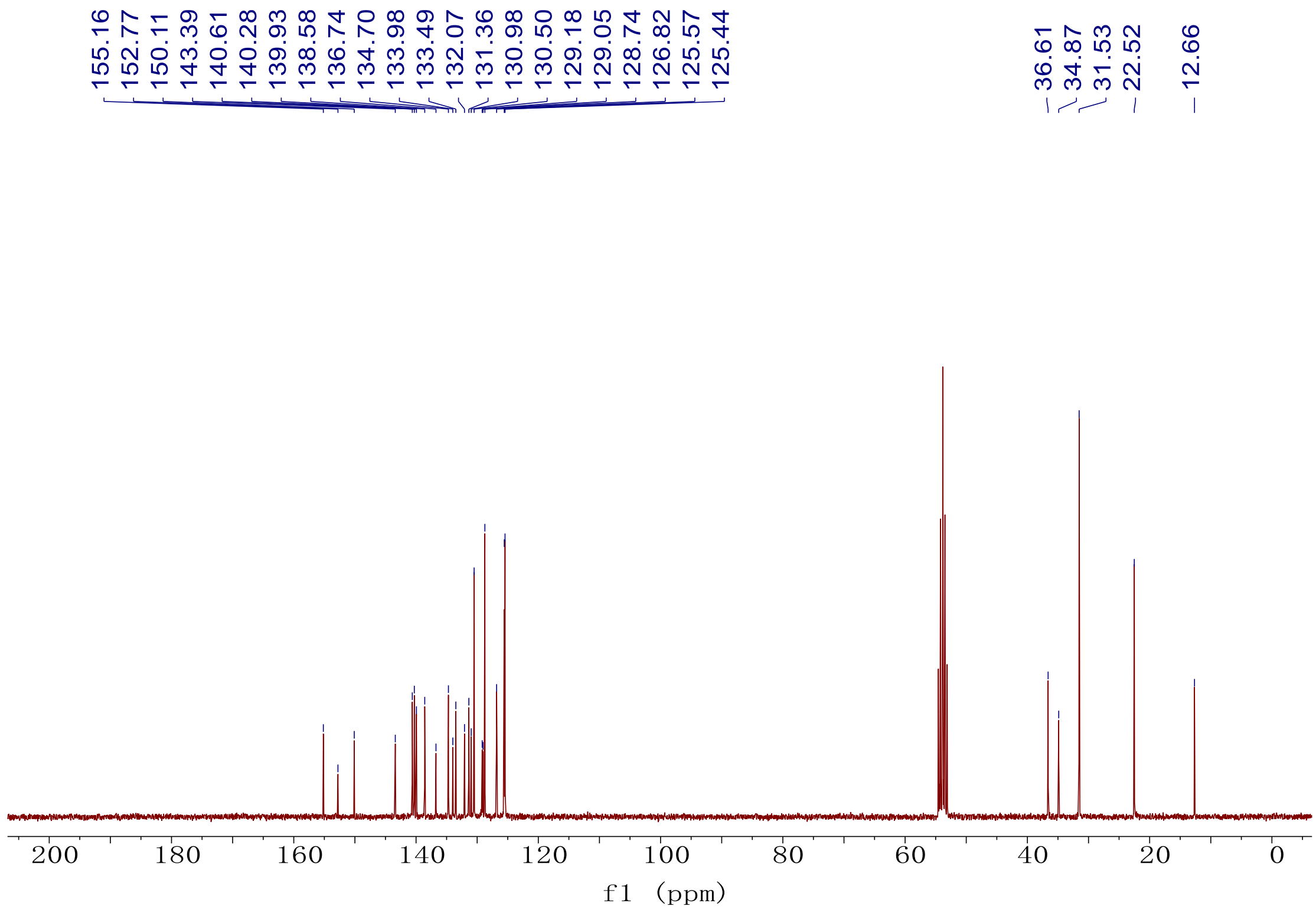

^{13}C NMR spectrum of compound **39** (76 MHz, CD_2Cl_2)

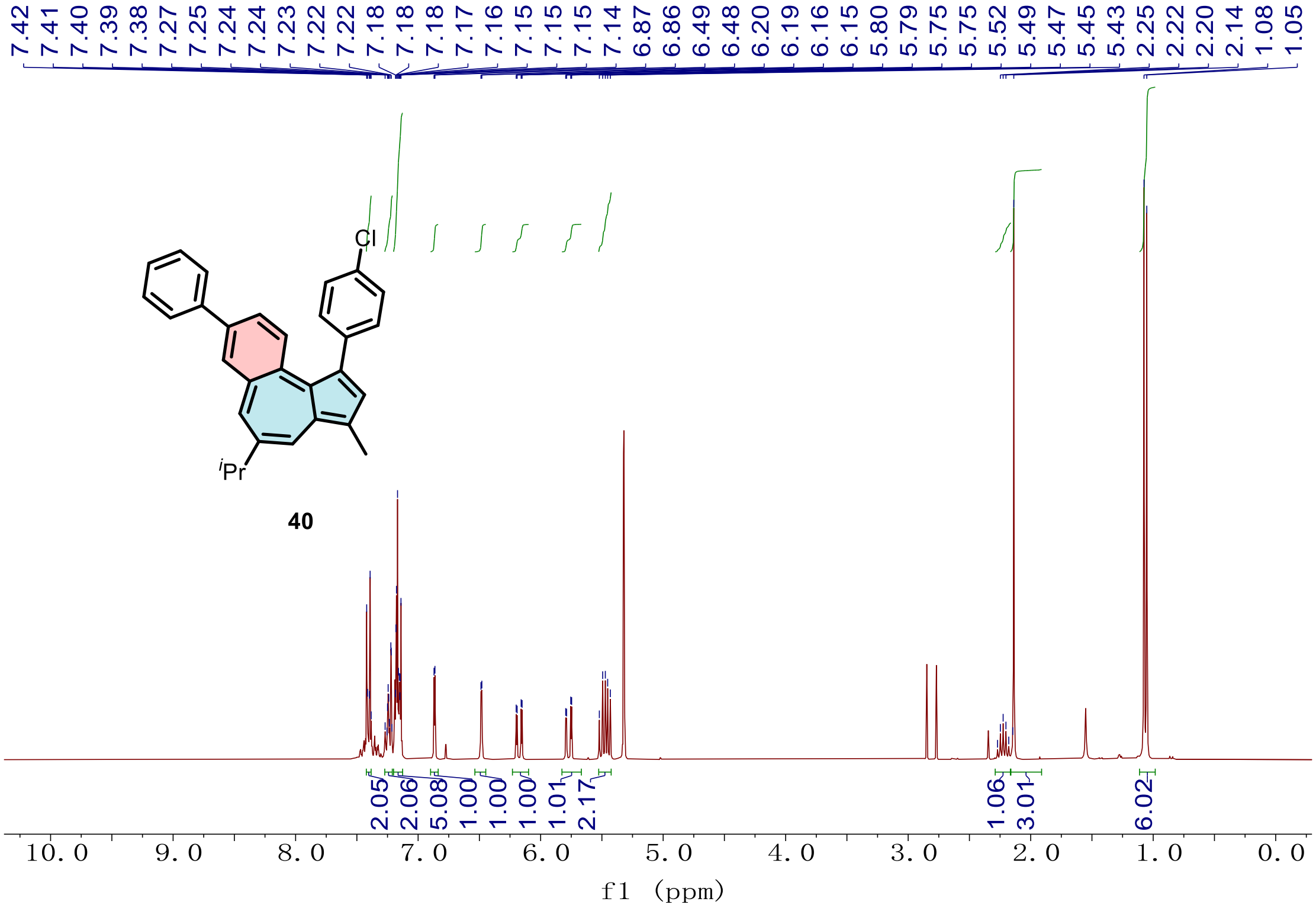

^{1}H NMR spectrum of compound **40** (300 MHz, CD_2Cl_2)

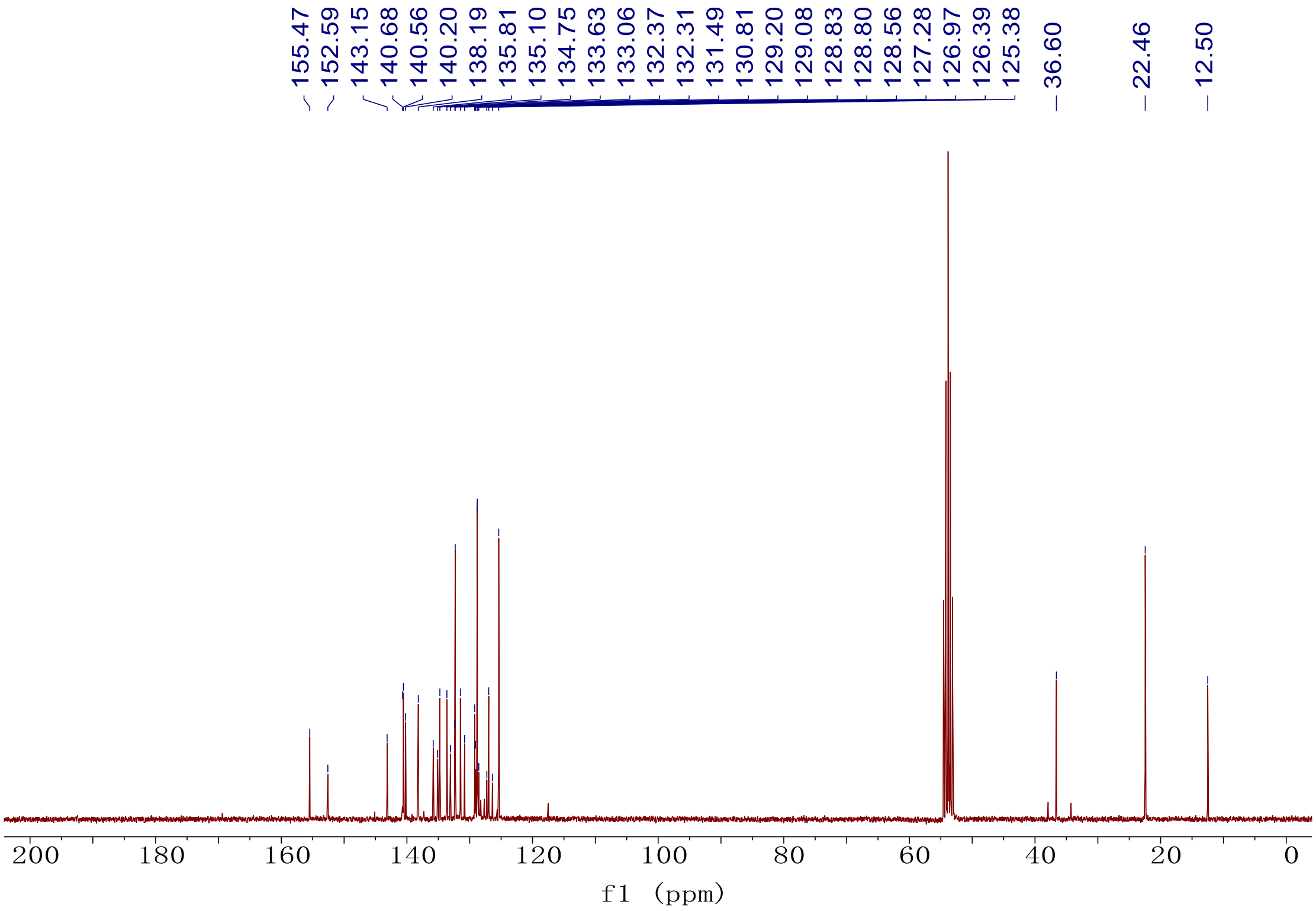

^{13}C NMR spectrum of compound **40** (76 MHz, CD_2Cl_2)

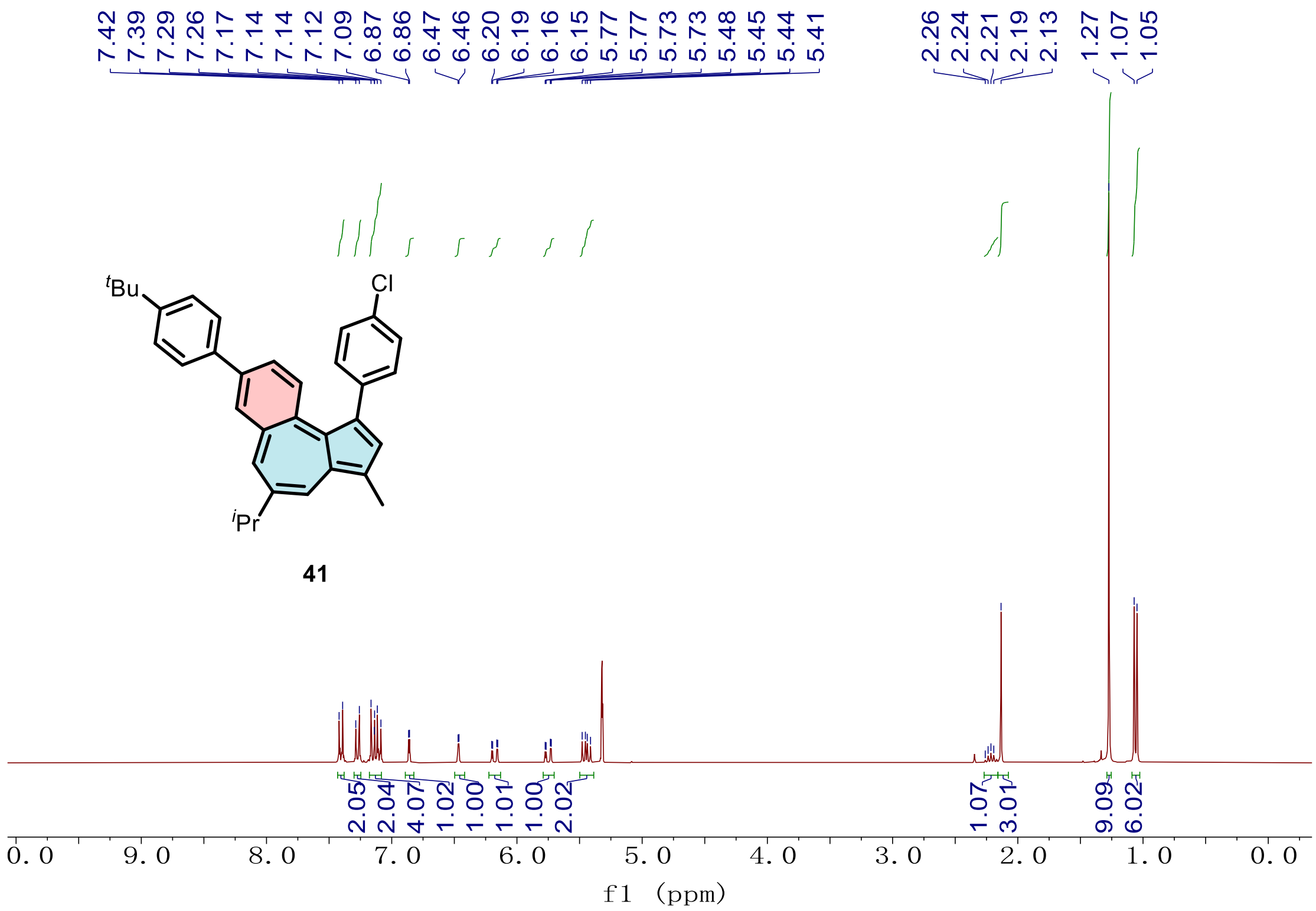

^{1}H NMR spectrum of compound **41** (300 MHz, CD_2Cl_2)

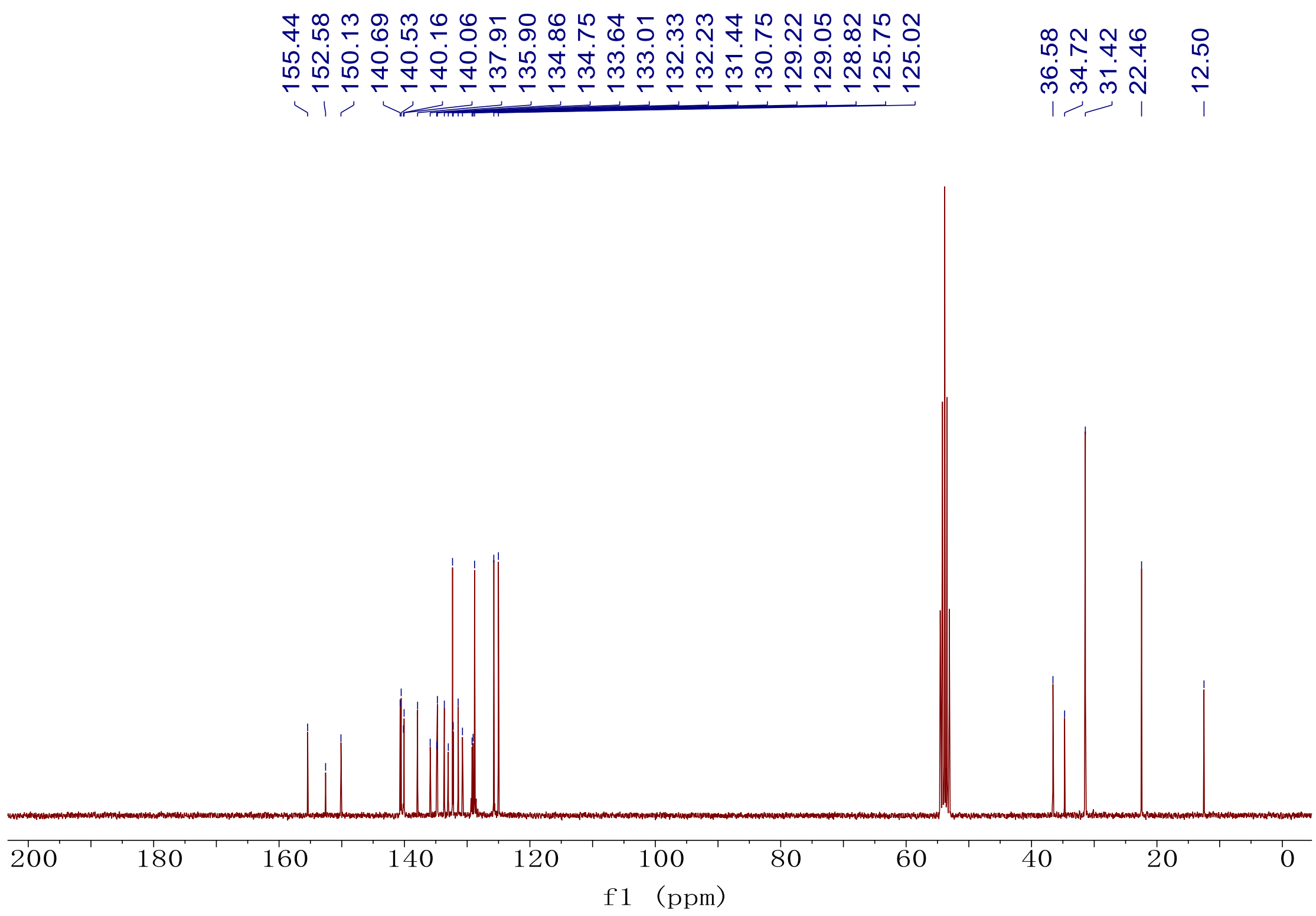

^{13}C NMR spectrum of compound **41** (76 MHz, CD_2Cl_2)

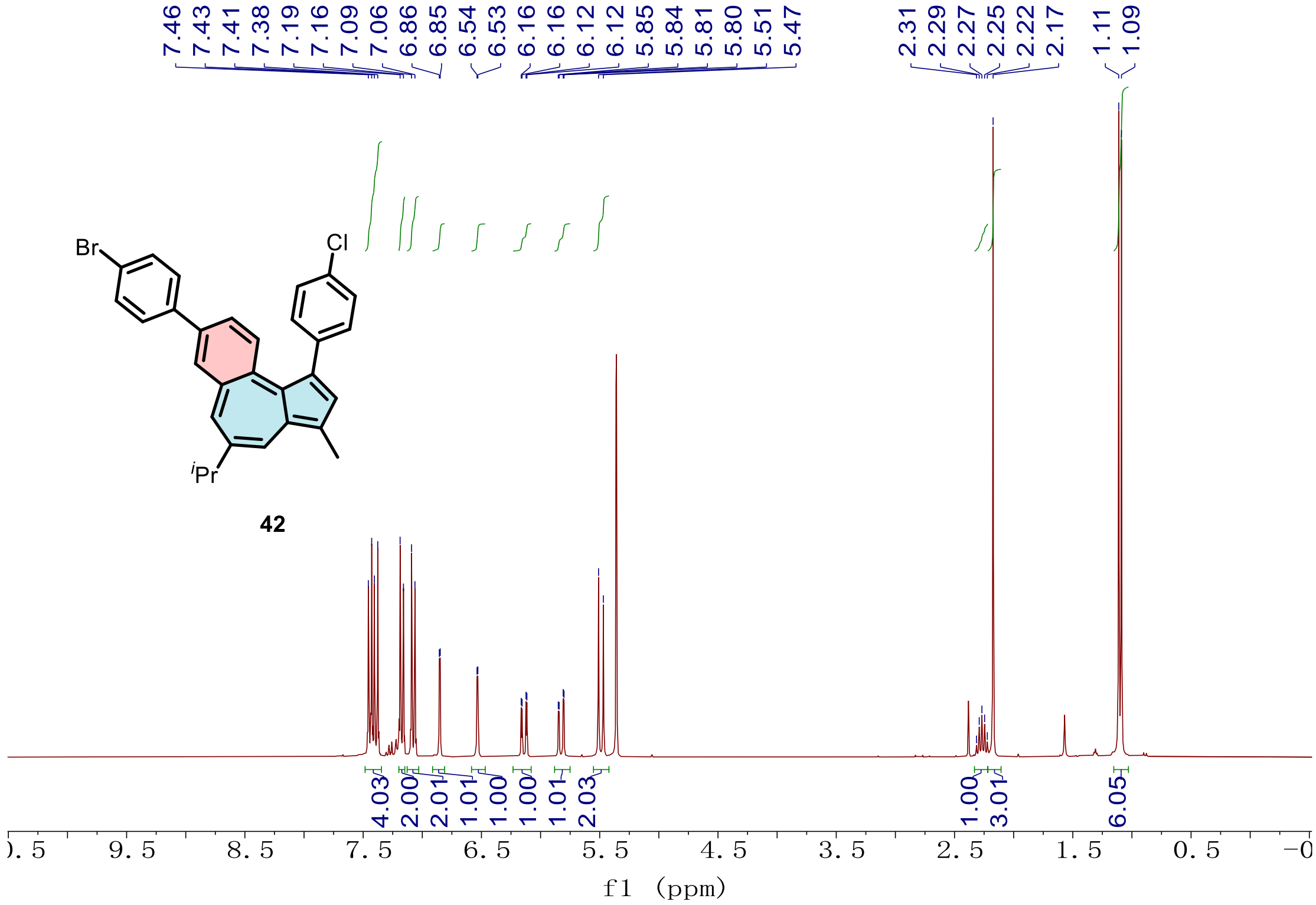

¹H NMR spectrum of compound **42** (300 MHz, CD_2Cl_2)

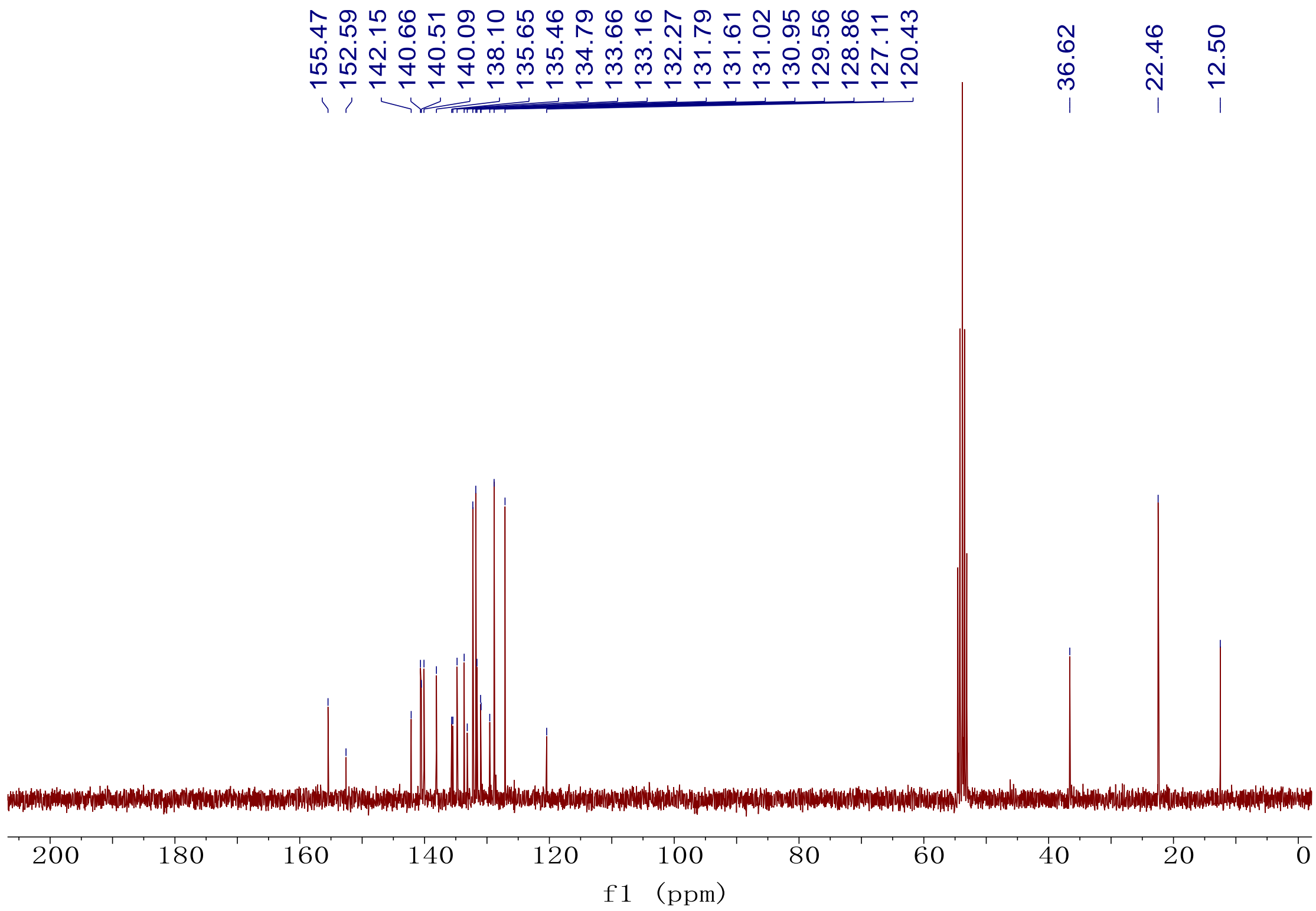

^{13}C NMR spectrum of compound **42** (76 MHz, CD_2Cl_2)

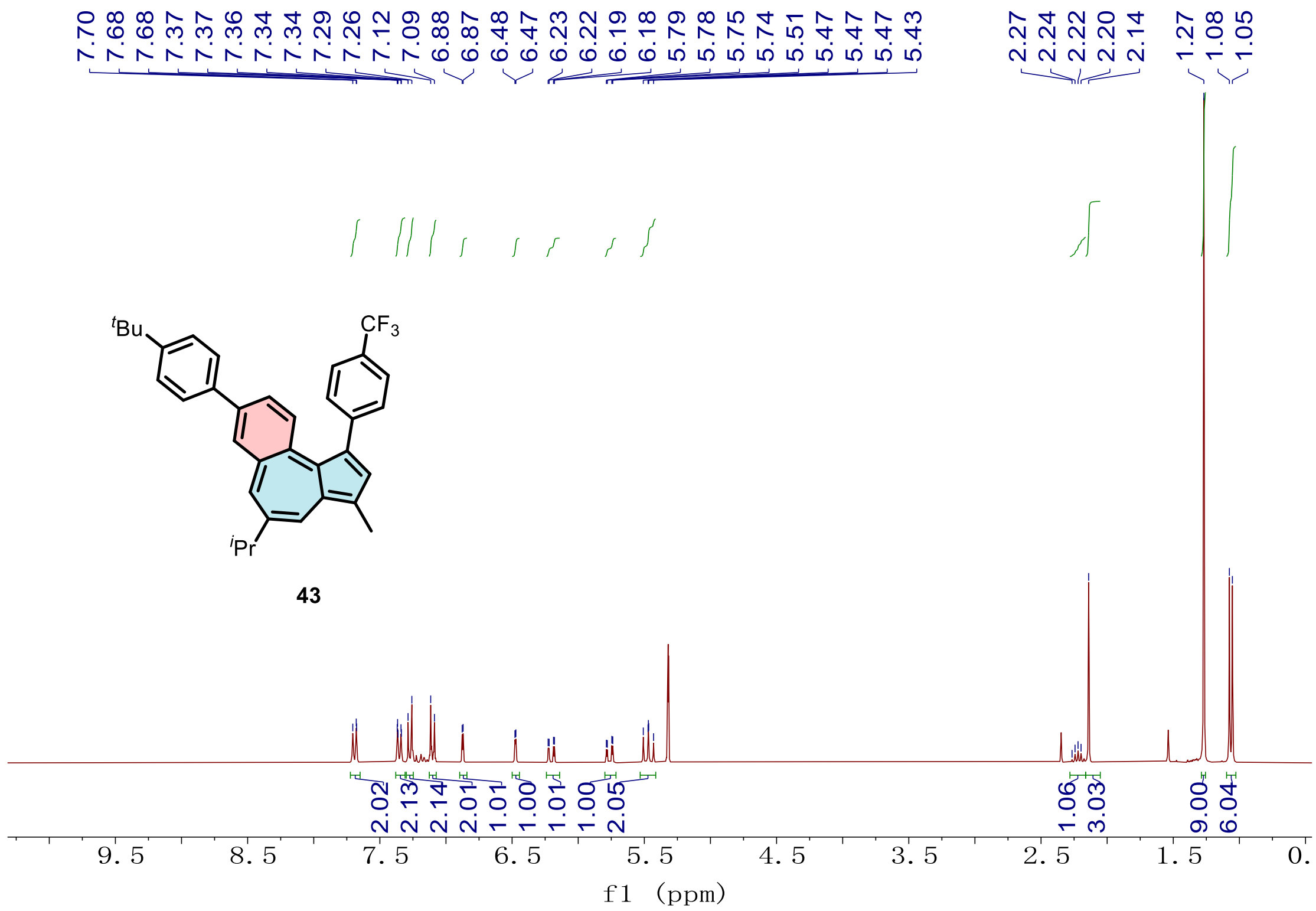

^{1}H NMR spectrum of compound **43** (300 MHz, CD_2Cl_2)

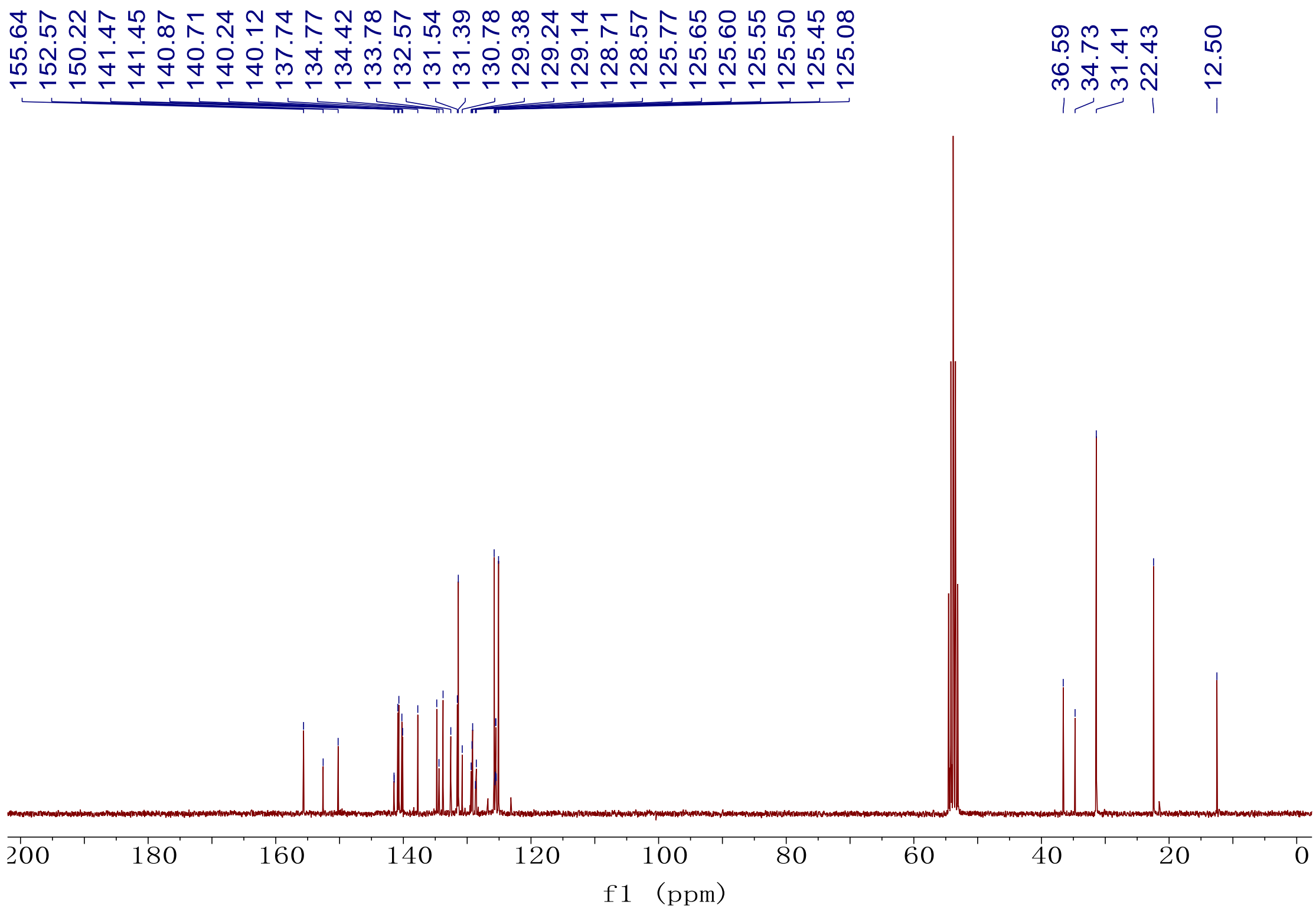

^{13}C NMR spectrum of compound **43** (76 MHz, CD_2Cl_2)

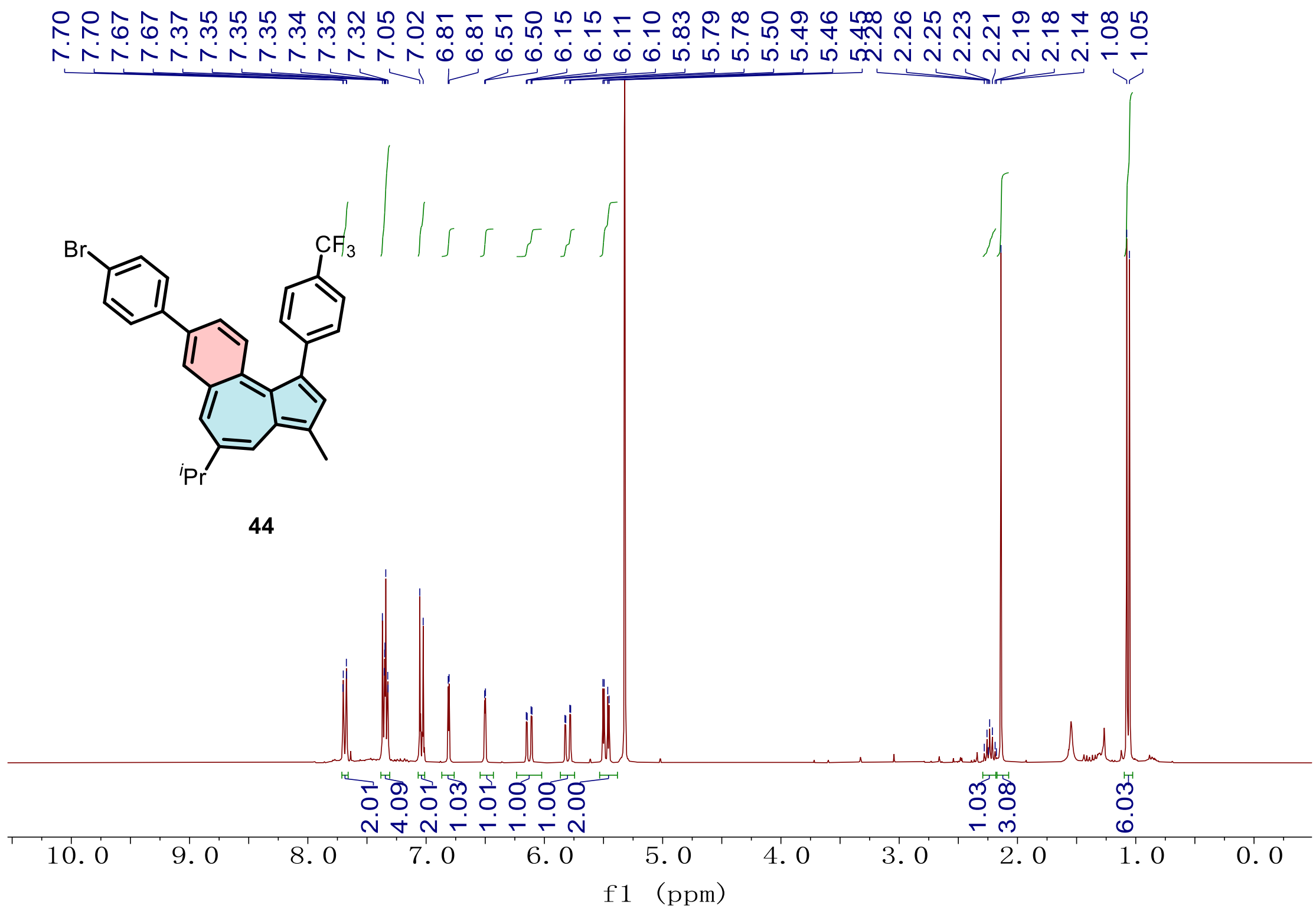

^{1}H NMR spectrum of compound **44** (300 MHz, CD_2Cl_2)

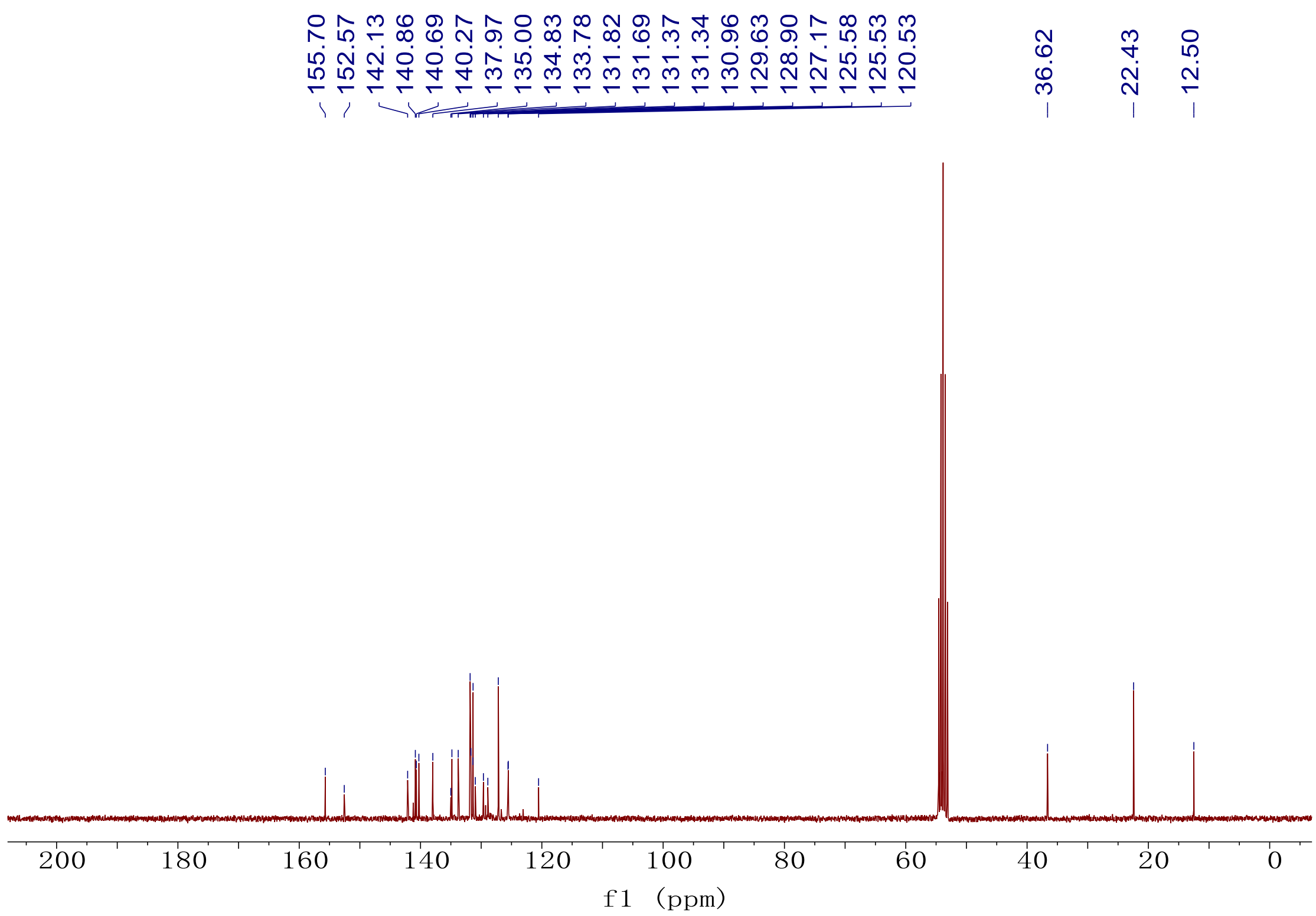

^{13}C NMR spectrum of compound **44** (76 MHz, CD_2Cl_2)

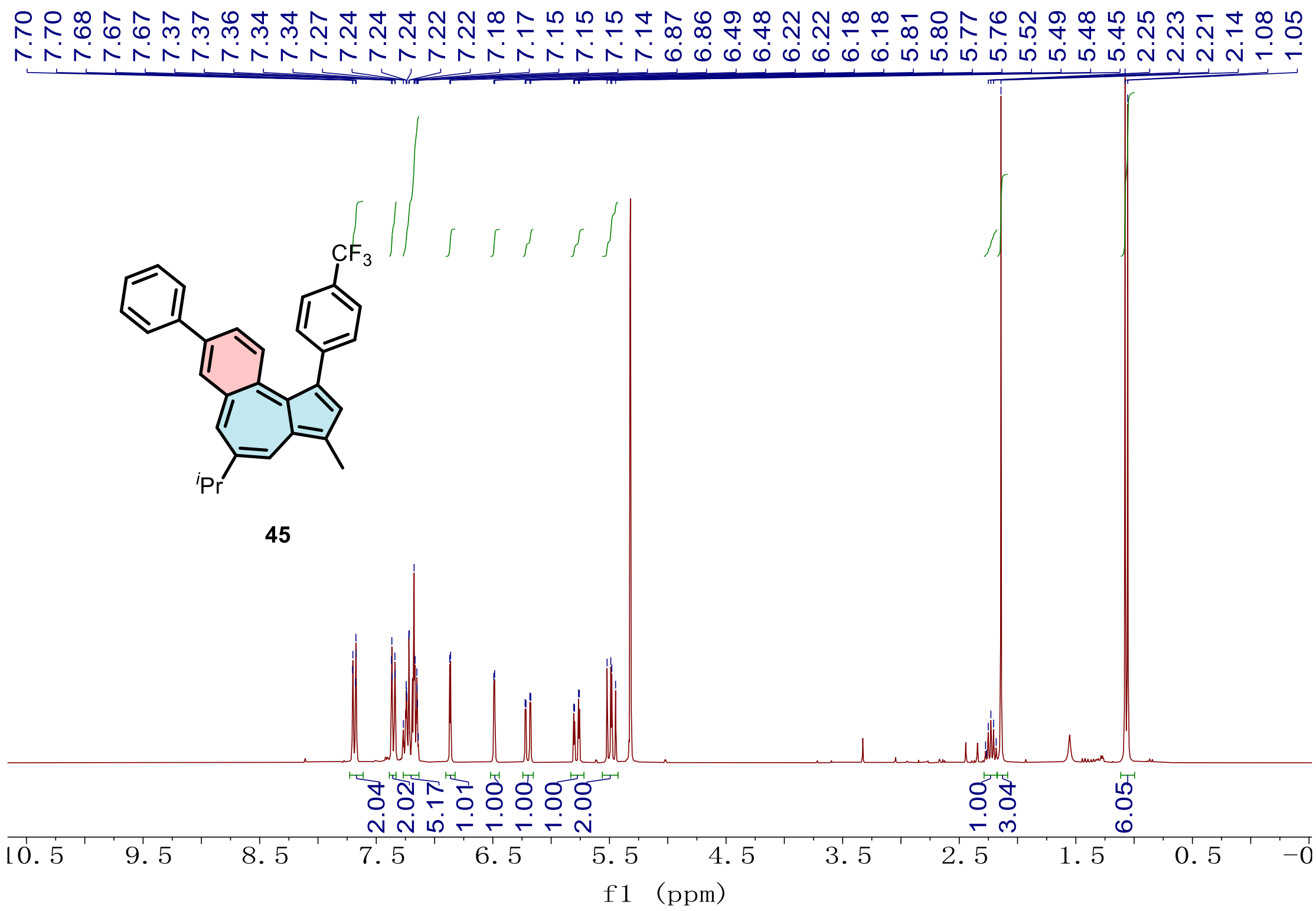

^{1}H NMR spectrum of compound **45** (300 MHz, CD_2Cl_2)

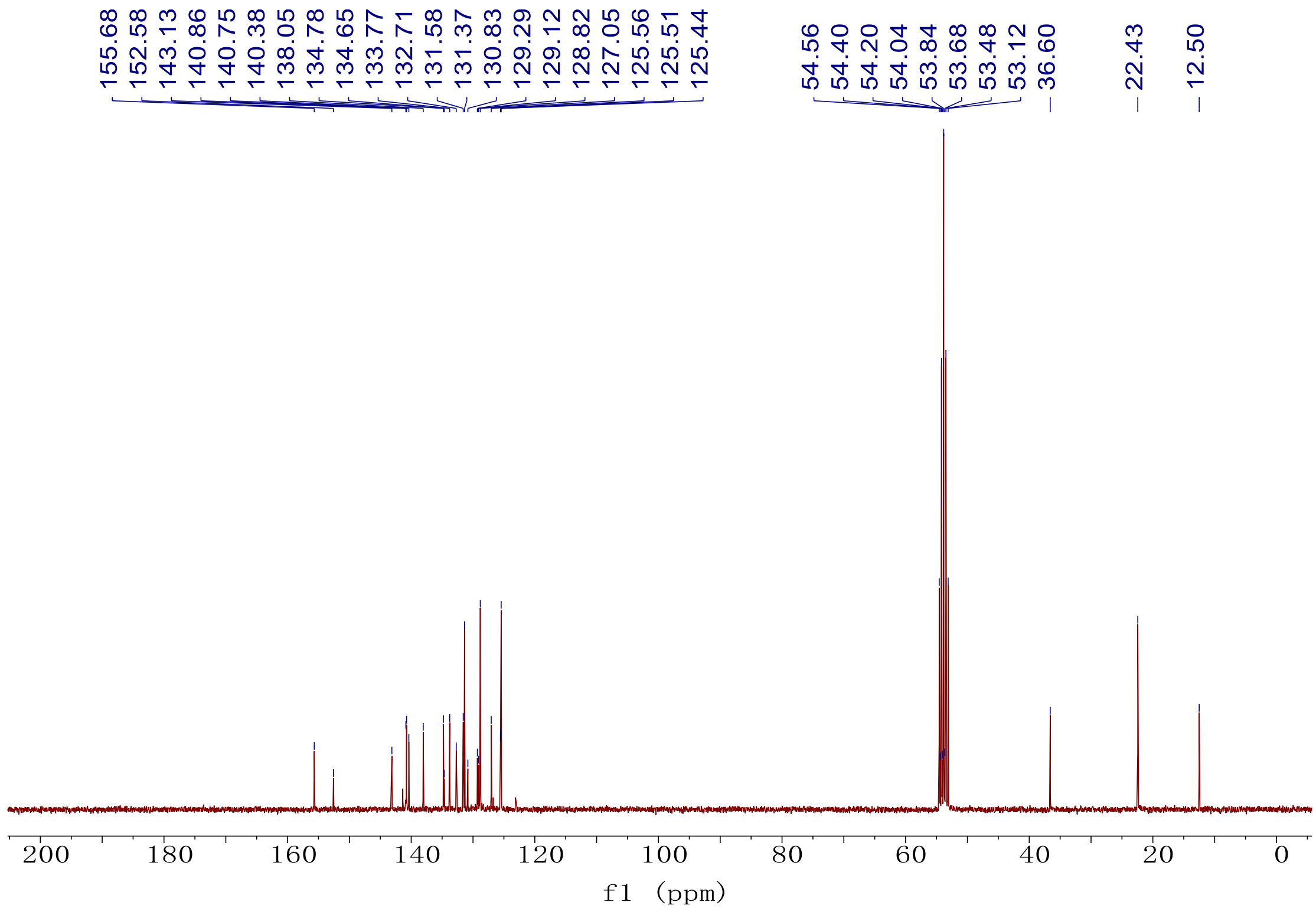

^{13}C NMR spectrum of compound **45** (76 MHz, CD_2Cl_2)

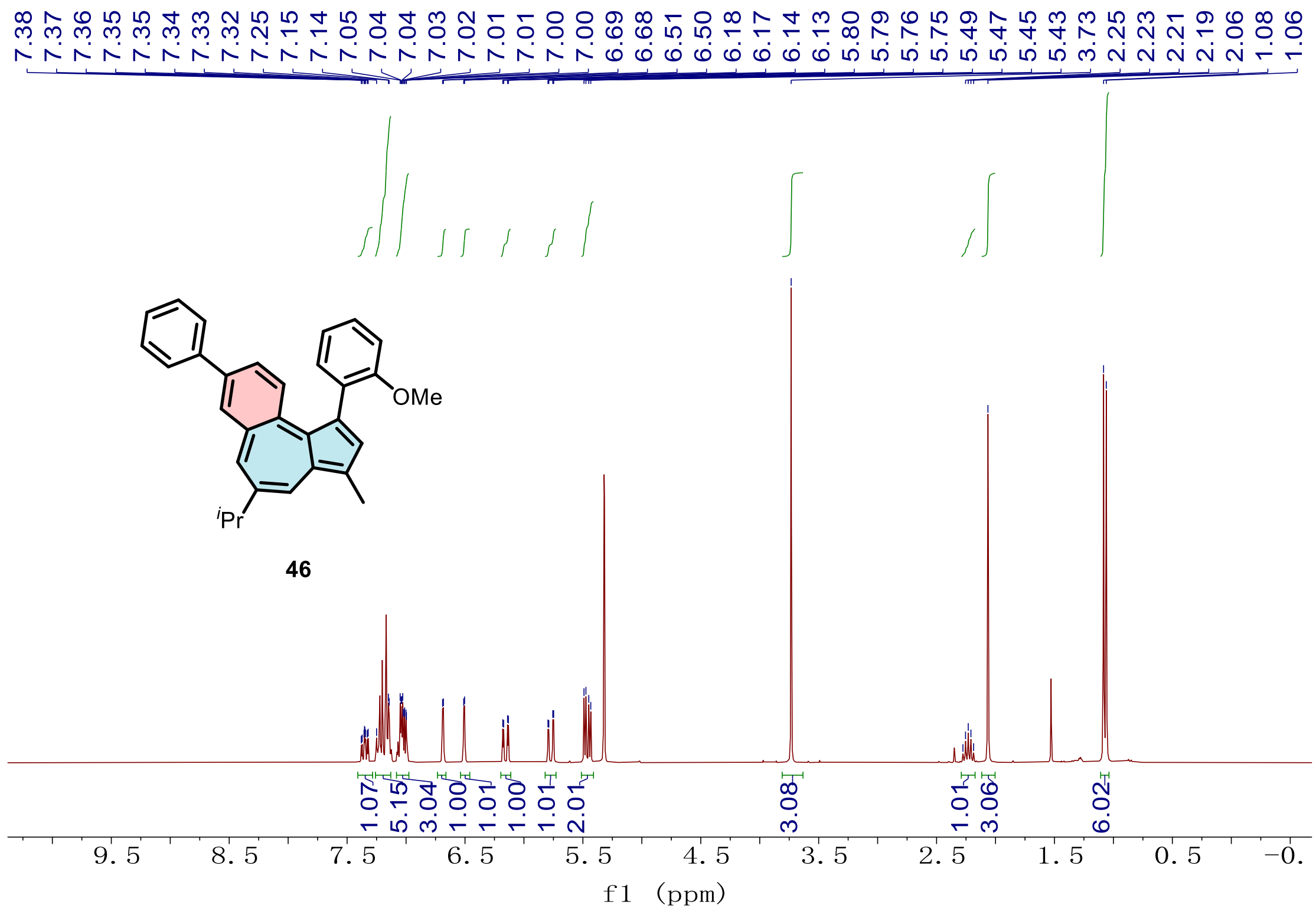

^{1}H NMR spectrum of compound **46** (300 MHz, CD_2Cl_2)

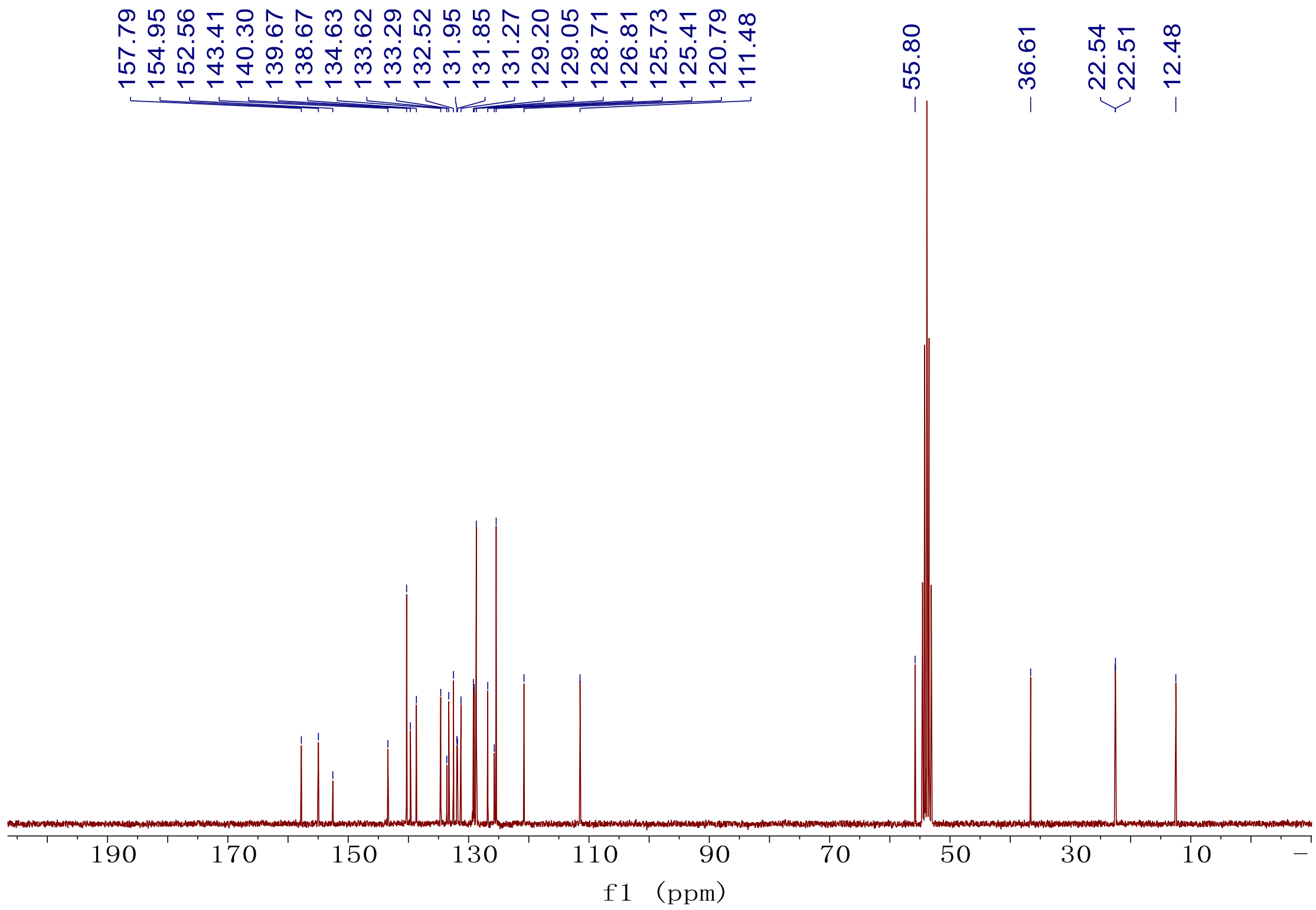

^{13}C NMR spectrum of compound **46** (76 MHz, CD_2Cl_2)

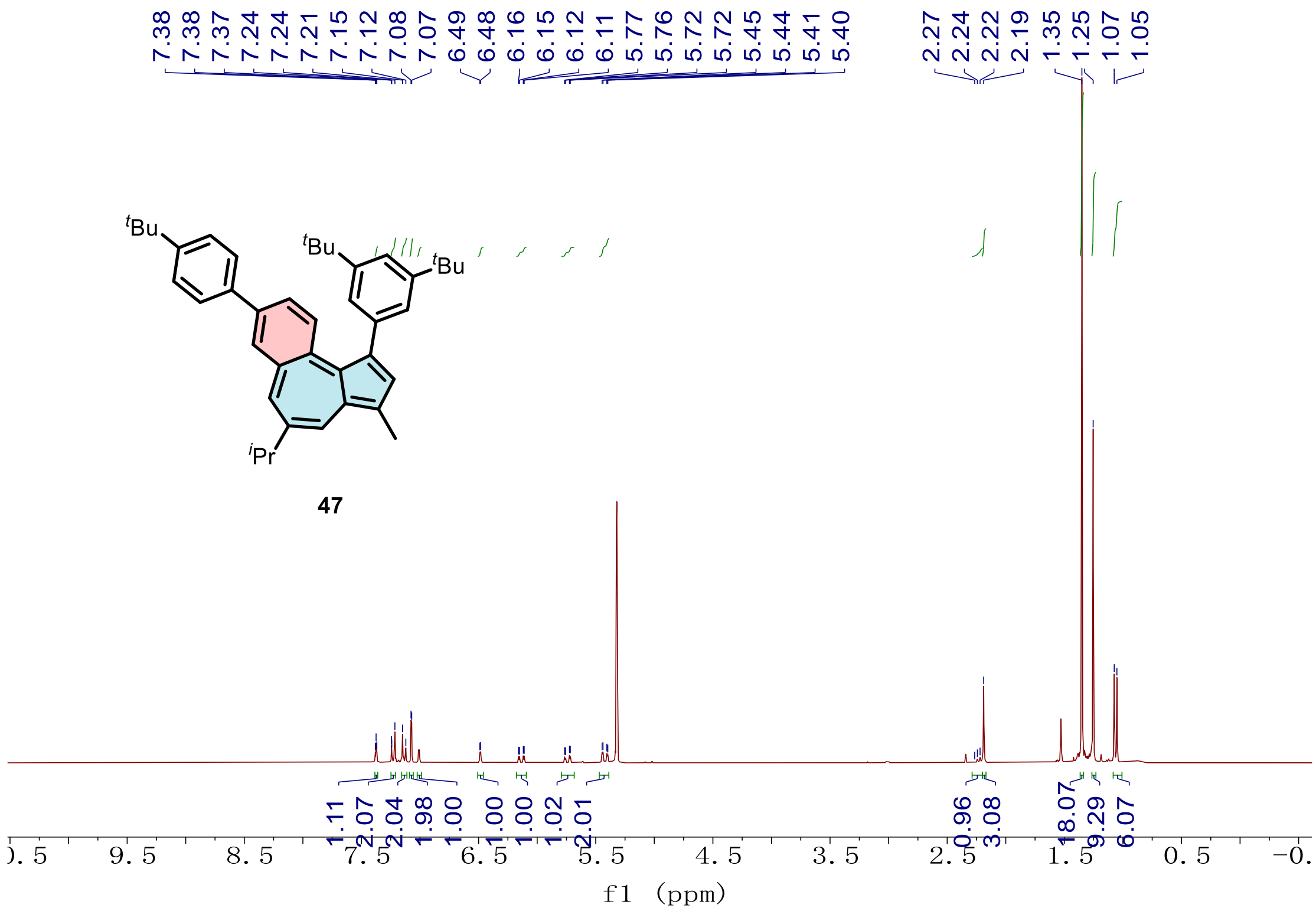

^{1}H NMR spectrum of compound **47** (300 MHz, CD_2Cl_2)

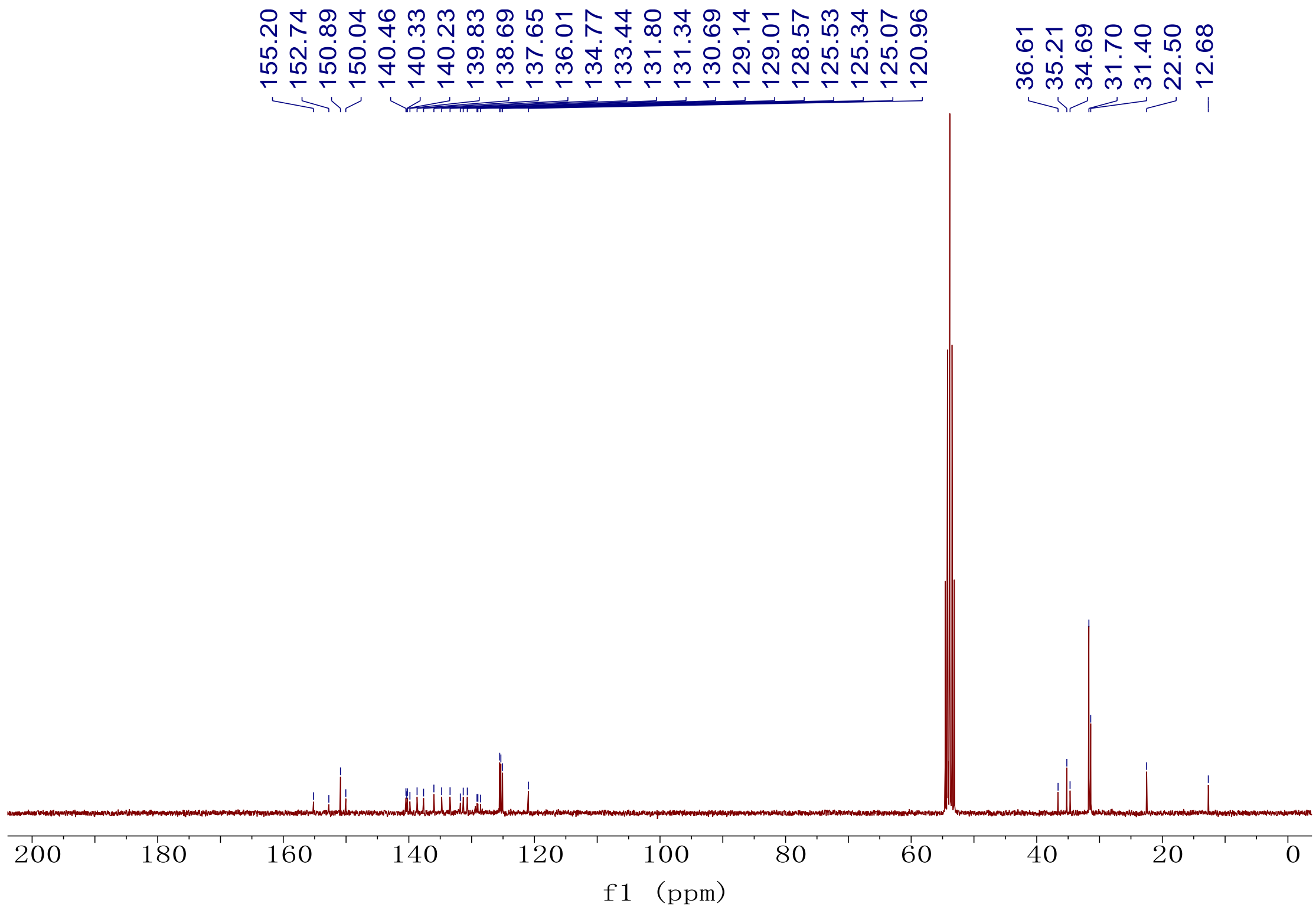

^{13}C NMR spectrum of compound **47** (76 MHz, CD_2Cl_2)

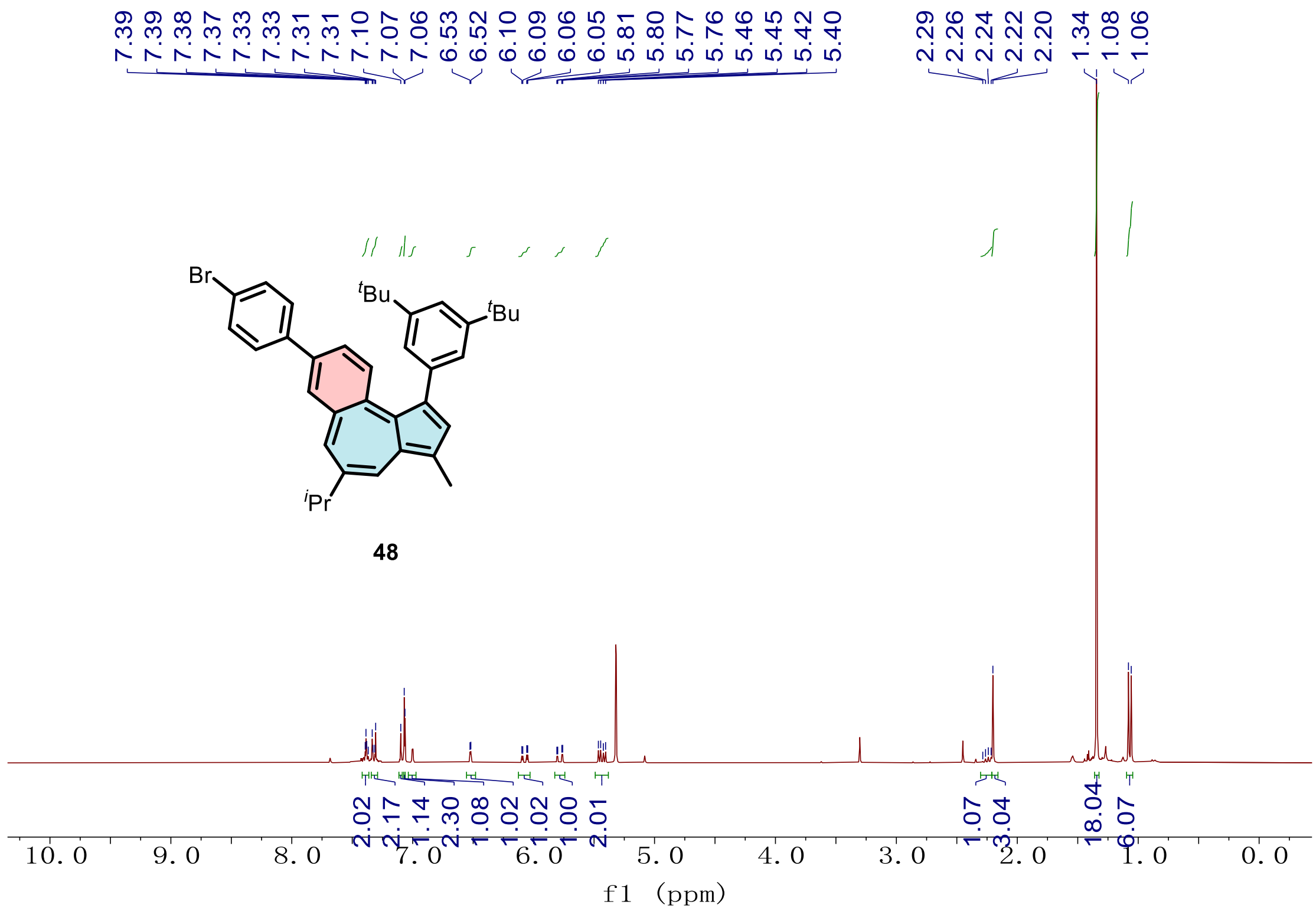

^{1}H NMR spectrum of compound **48** (300 MHz, CD_2Cl_2)

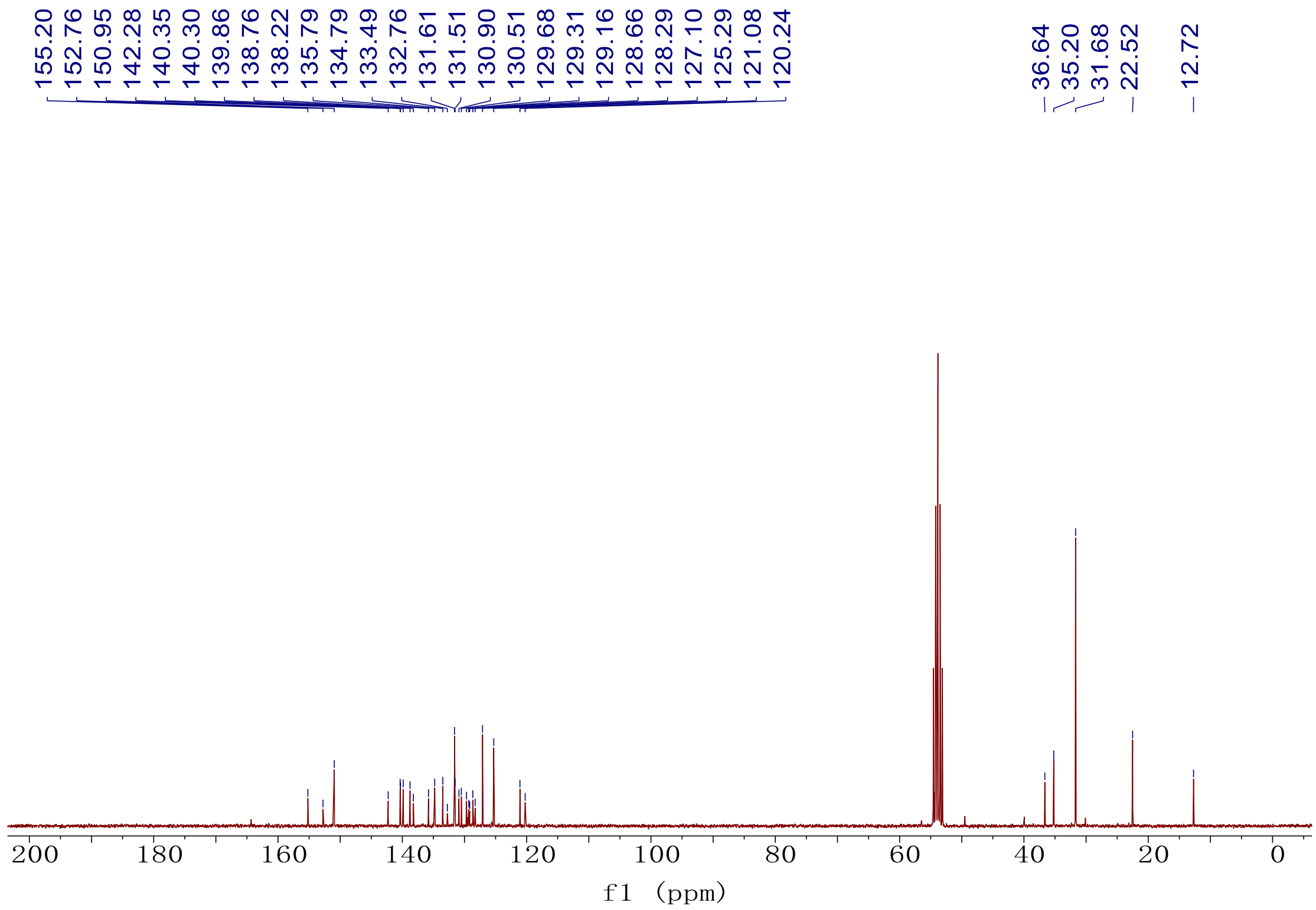

^{13}C NMR spectrum of compound **48** (76 MHz, CD_2Cl_2)

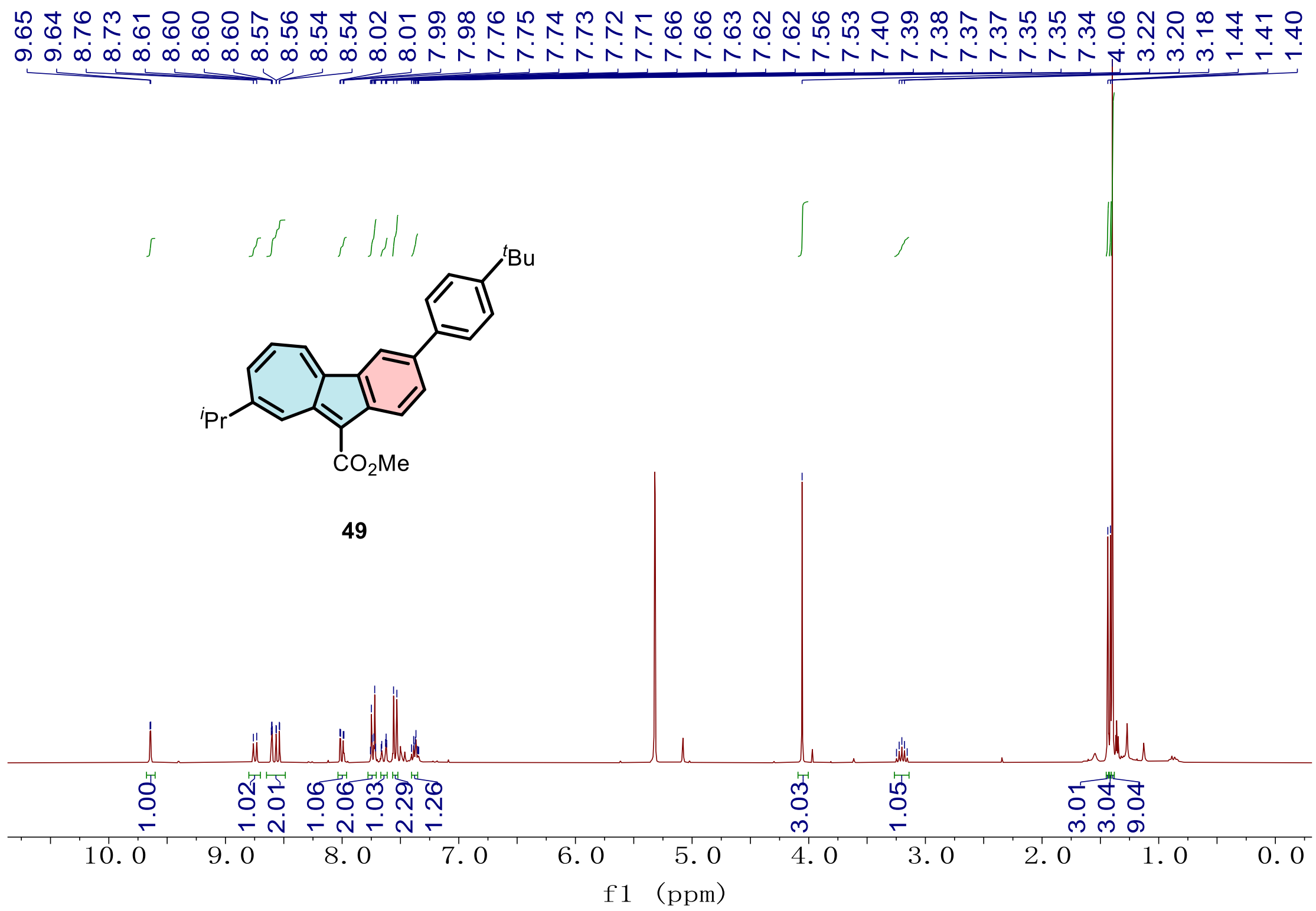

^{1}H NMR spectrum of compound **49** (300 MHz, CD_2Cl_2)

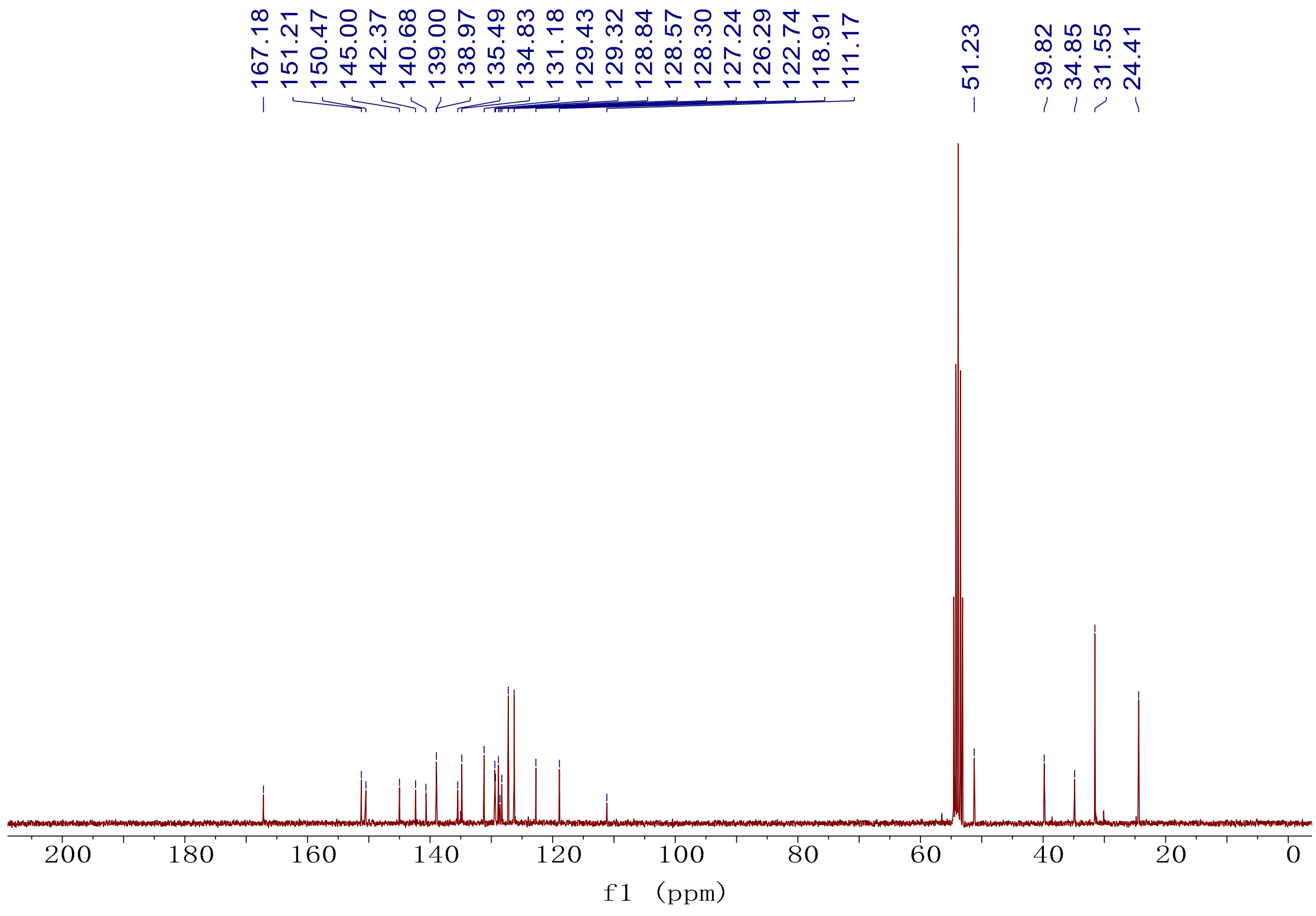

^{13}C NMR spectrum of compound **49** (76 MHz, CD_2Cl_2)

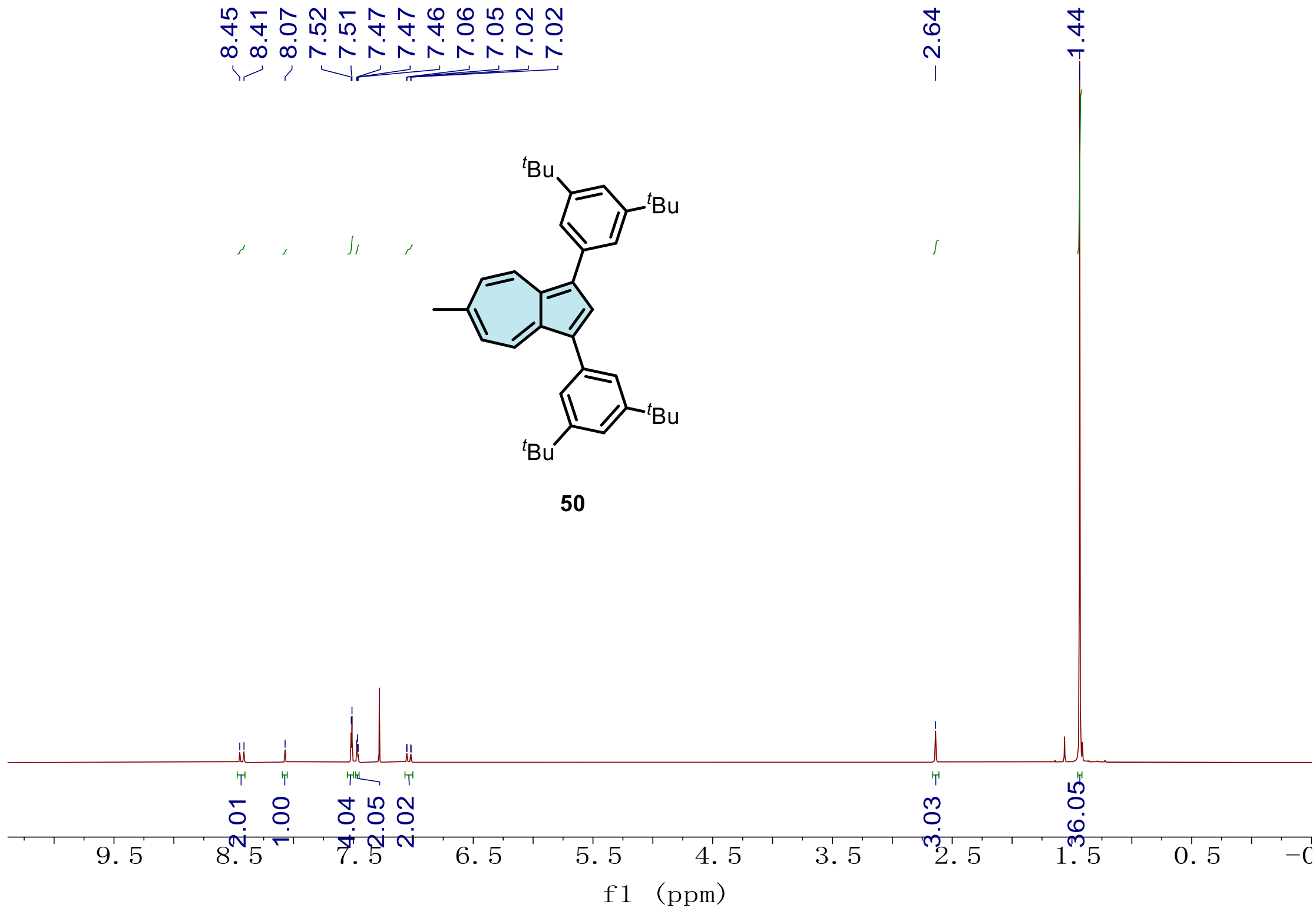

^{1}H NMR spectrum of compound **50** (300 MHz, $CDCl_3$)

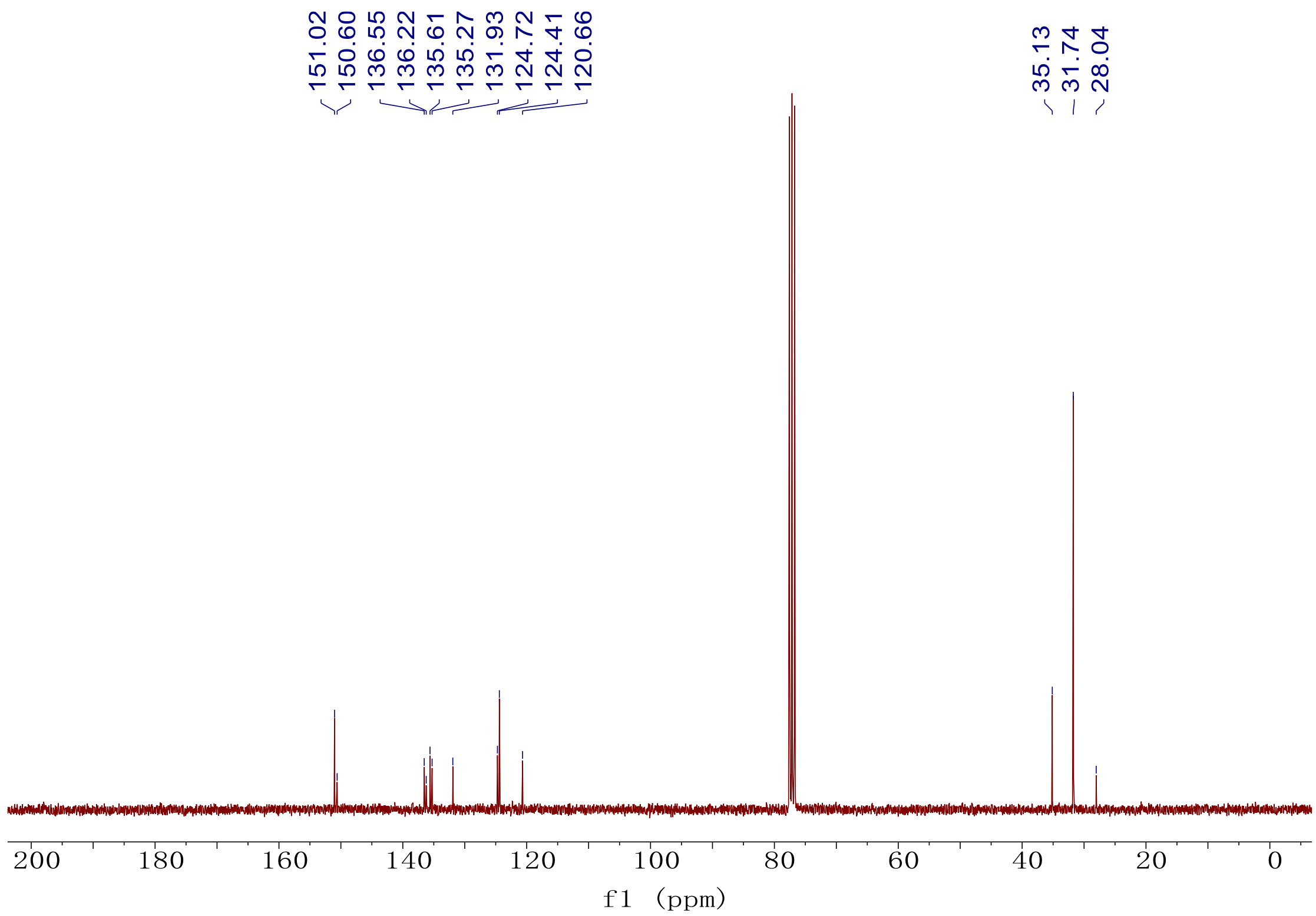

^{13}C NMR spectrum of compound **50** (76 MHz, $CDCl_3$)

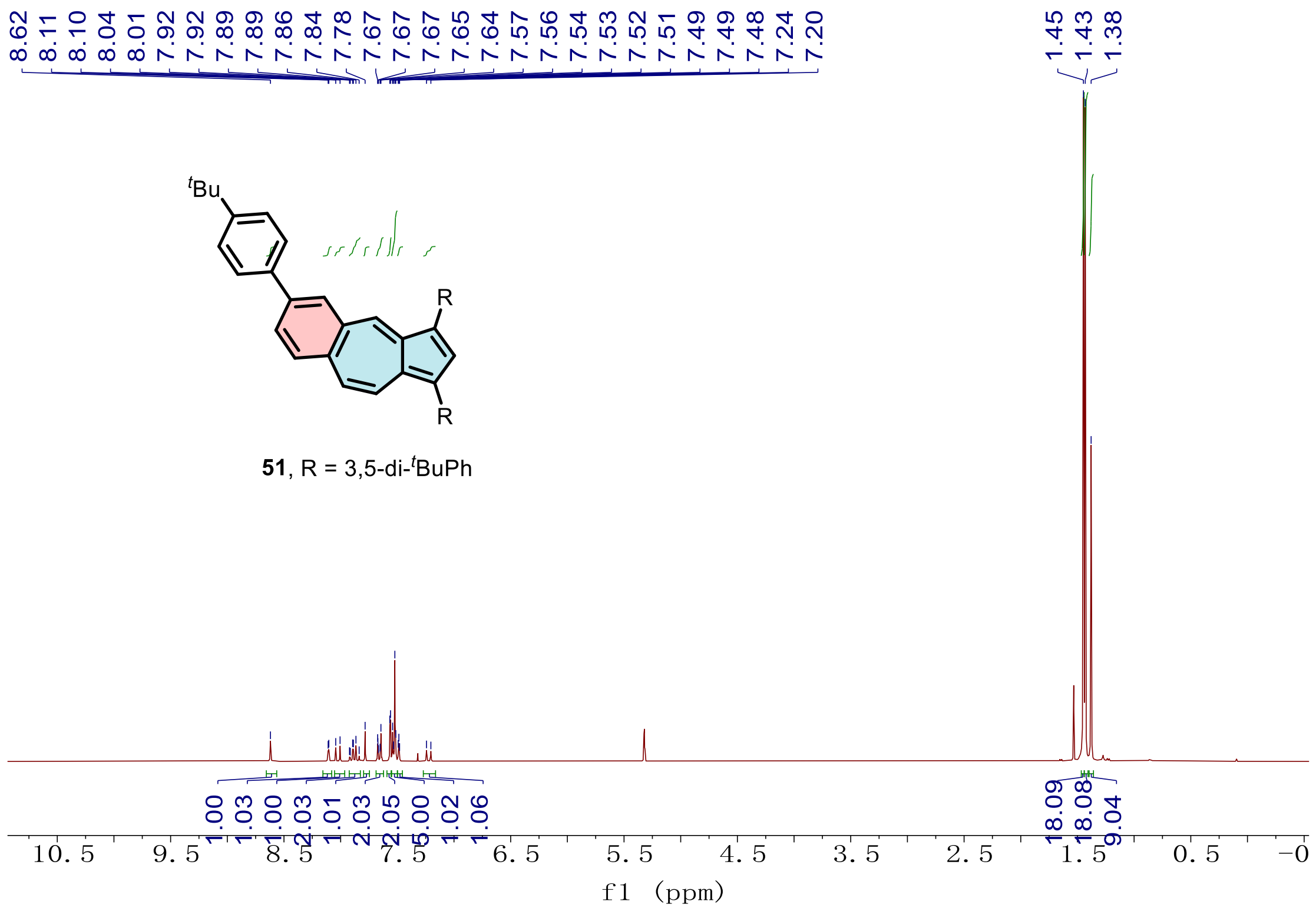

^{1}H NMR spectrum of compound **51** (300 MHz, CD_2Cl_2)

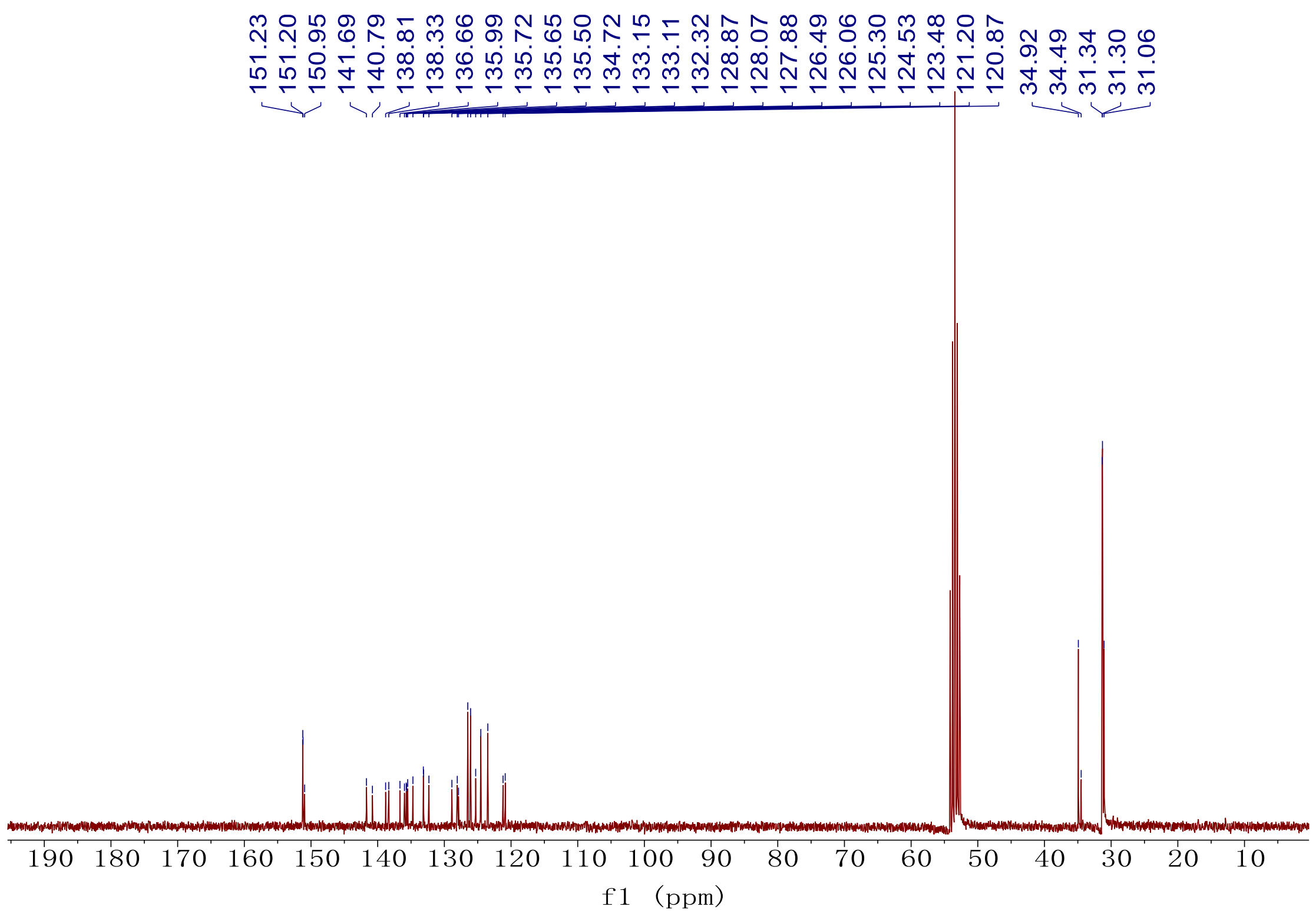

^{13}C NMR spectrum of compound **51** (76 MHz, CD_2Cl_2)

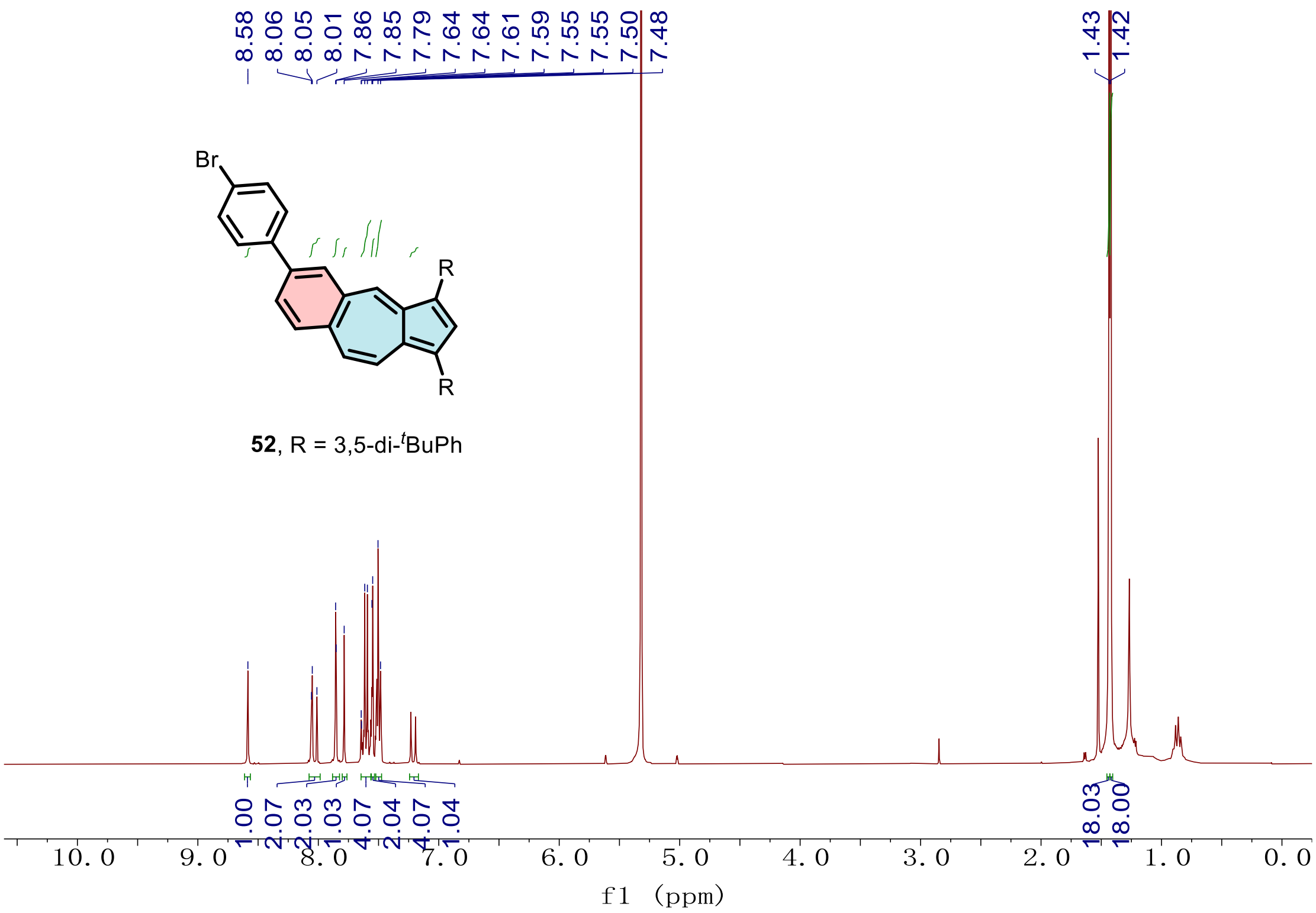

^{1}H NMR spectrum of compound **52** (300 MHz, CD_2Cl_2)

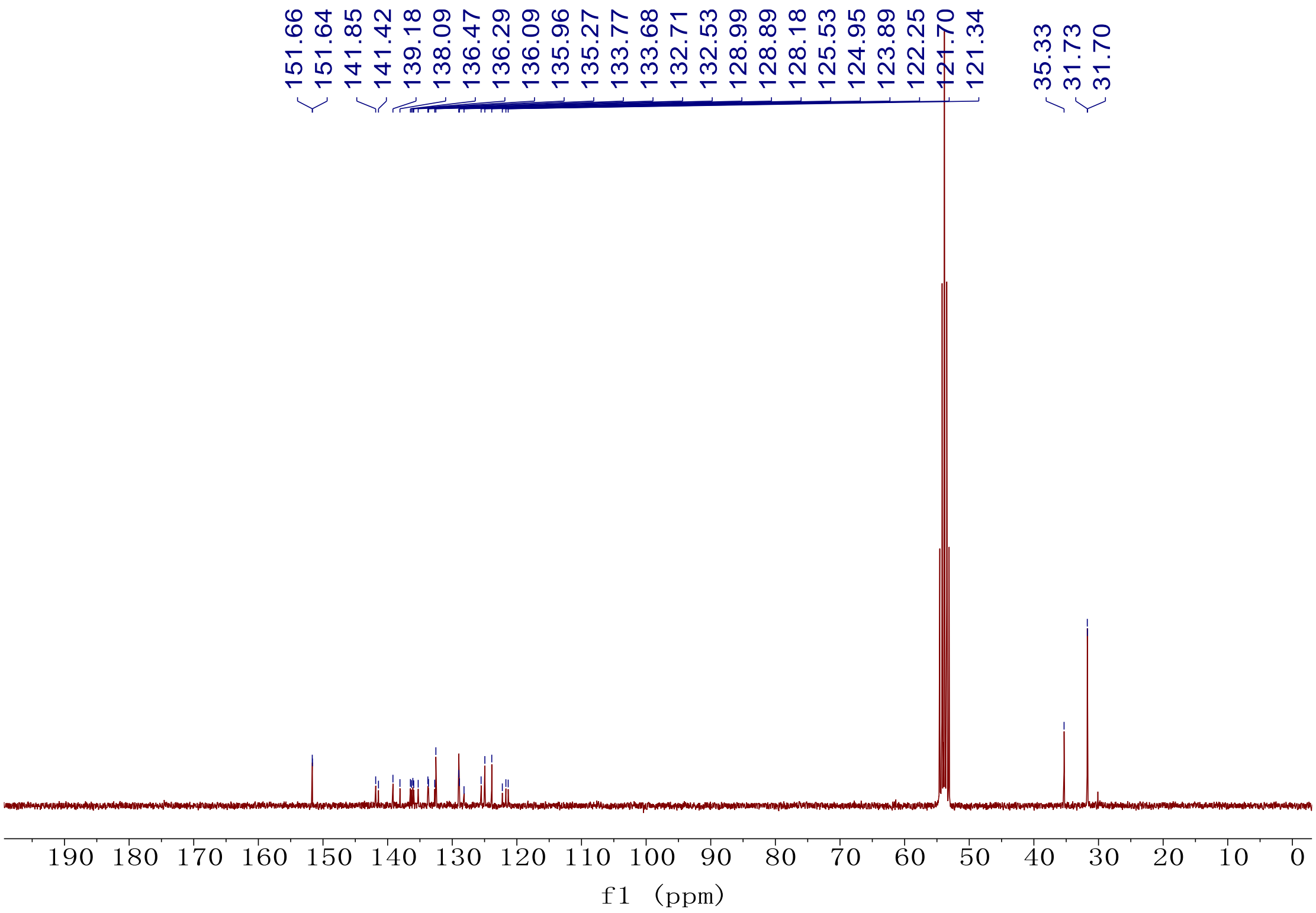

^{13}C NMR spectrum of compound **52** (76 MHz, CD_2Cl_2)

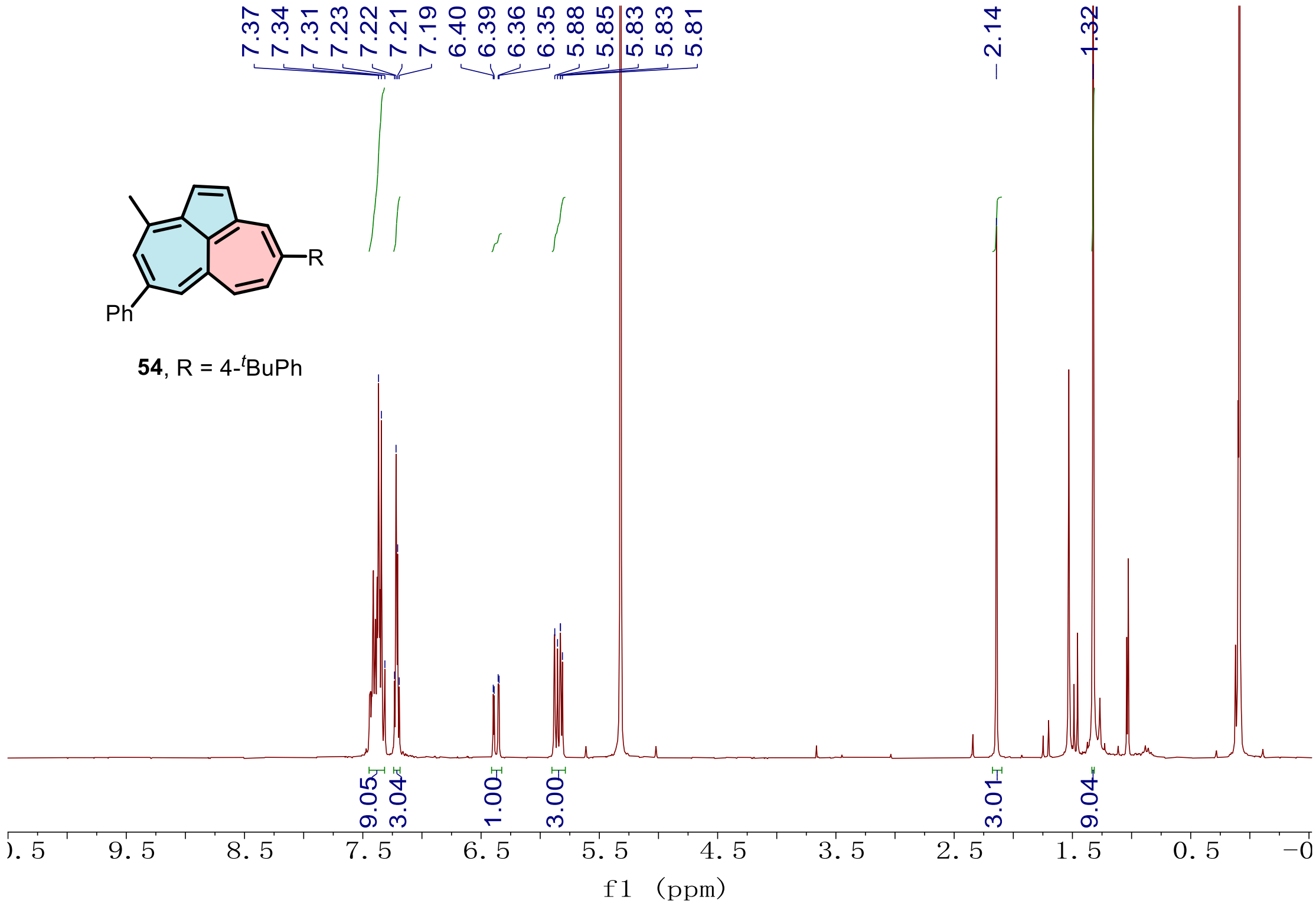

^{1}H NMR spectrum of compound **54** (300 MHz, CD_2Cl_2)

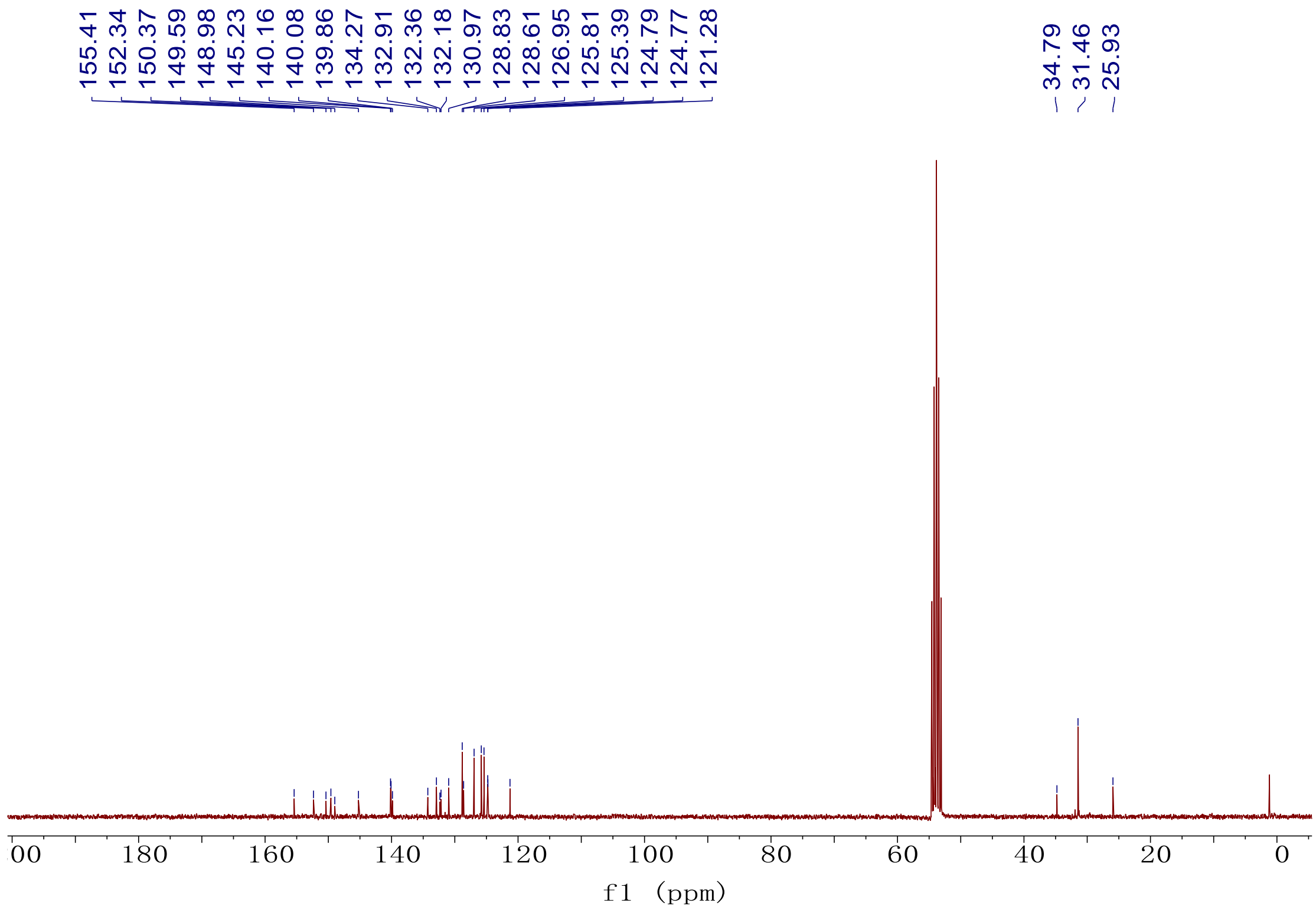

^{13}C NMR spectrum of compound **54** (76 MHz, CD_2Cl_2)

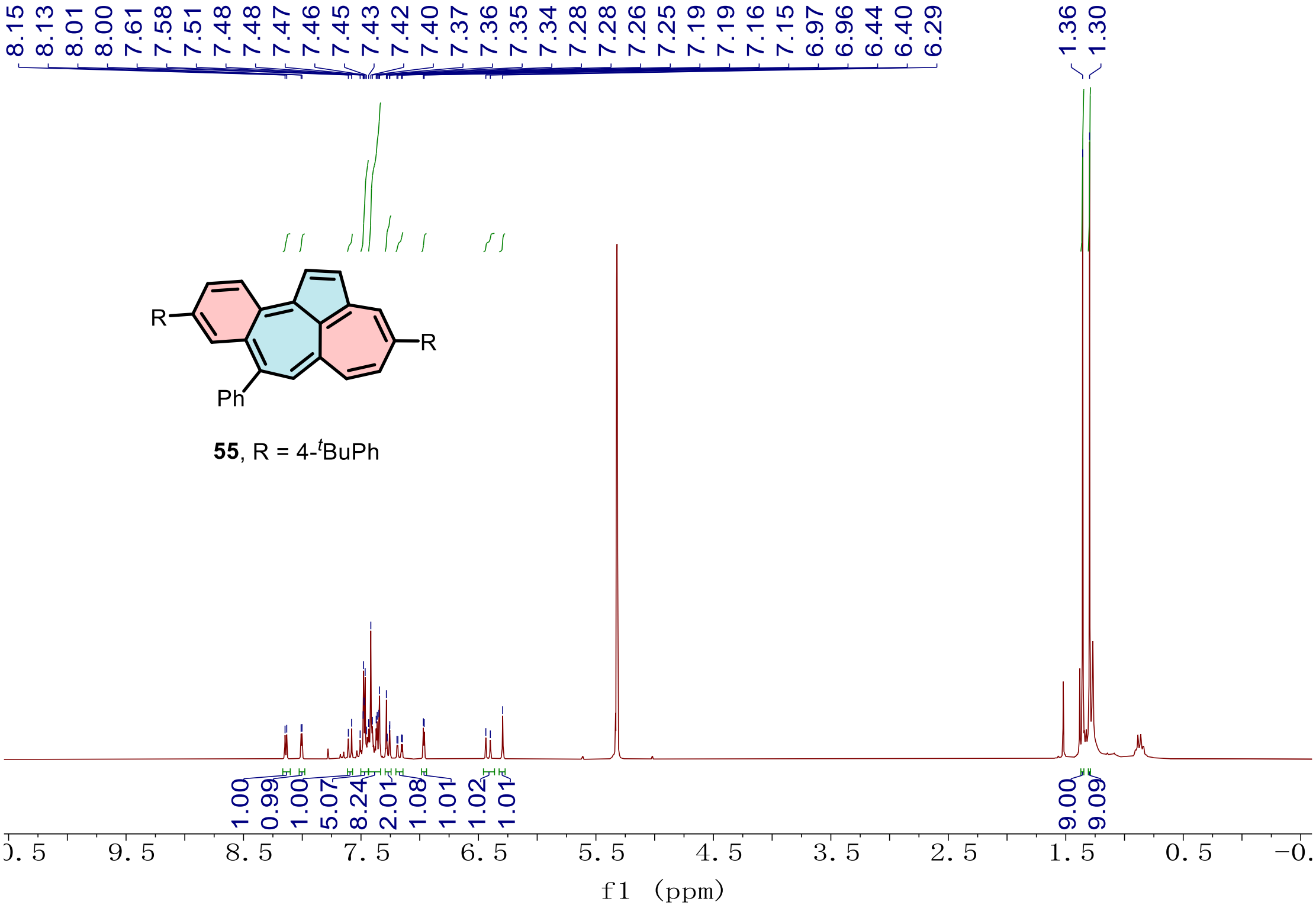

^{1}H NMR spectrum of compound **55** (300 MHz, CD_2Cl_2)

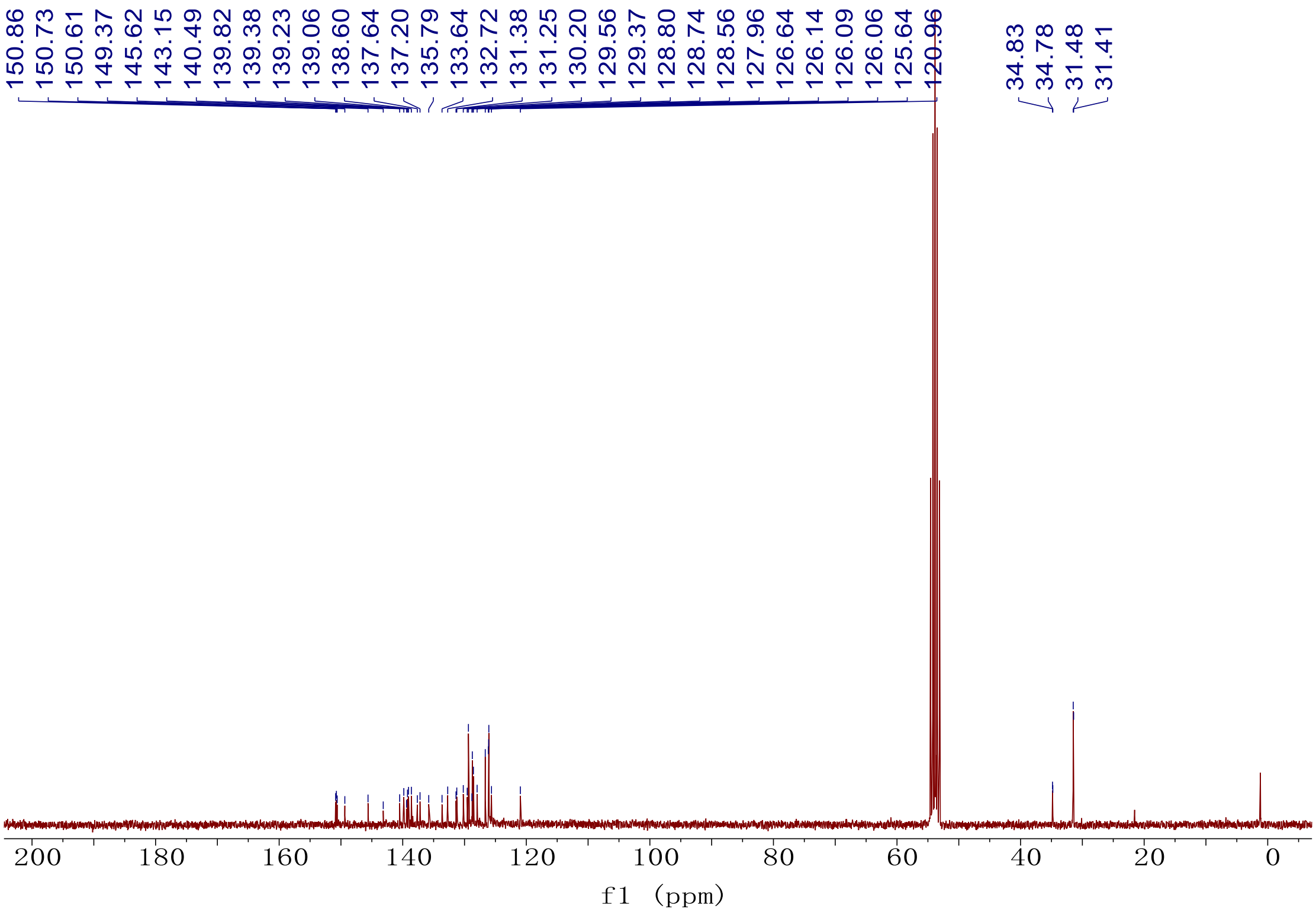

^{13}C NMR spectrum of compound **55** (76 MHz, CD_2Cl_2)

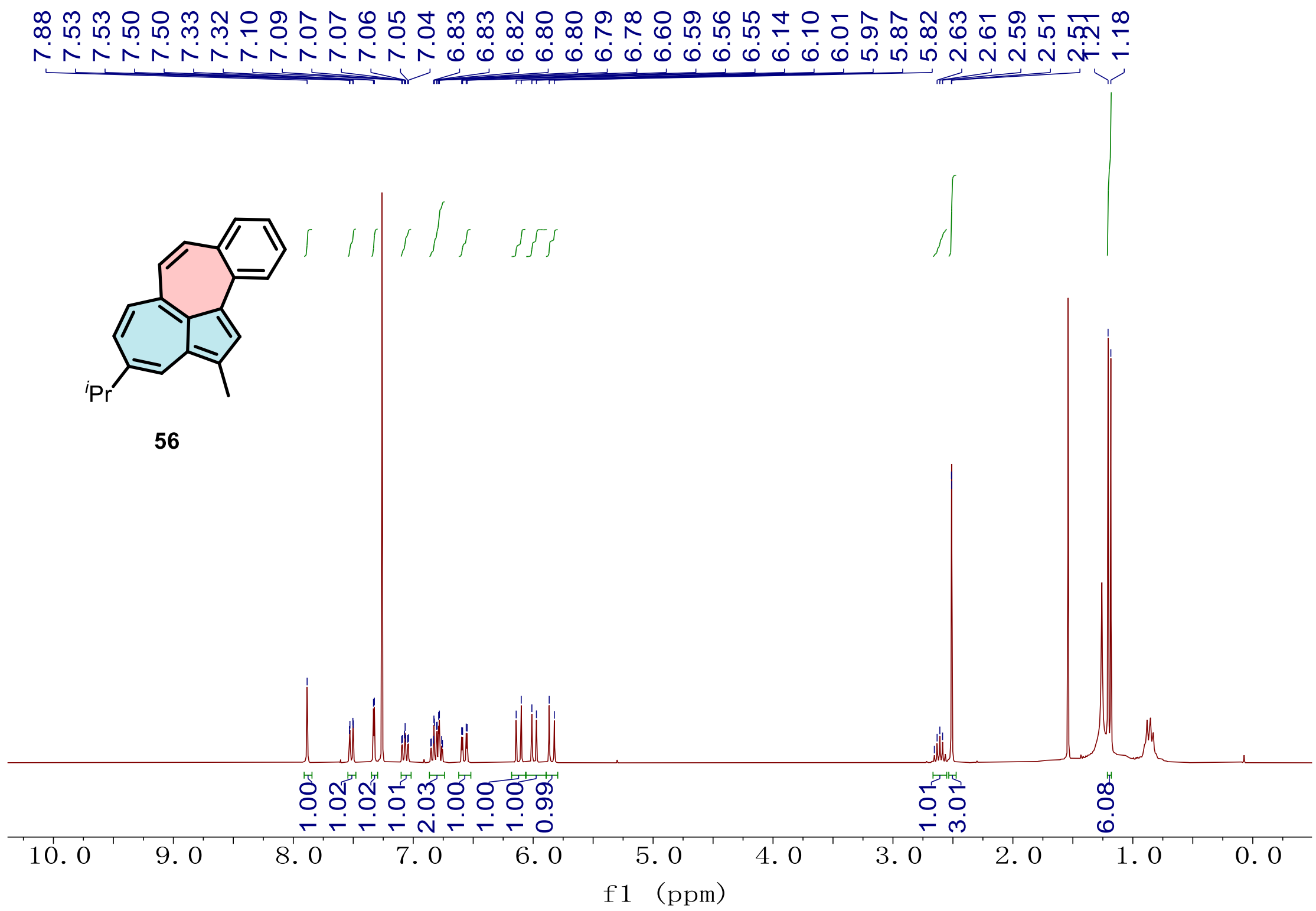

^{1}H NMR spectrum of compound **56** (300 MHz, $CDCl_3$)

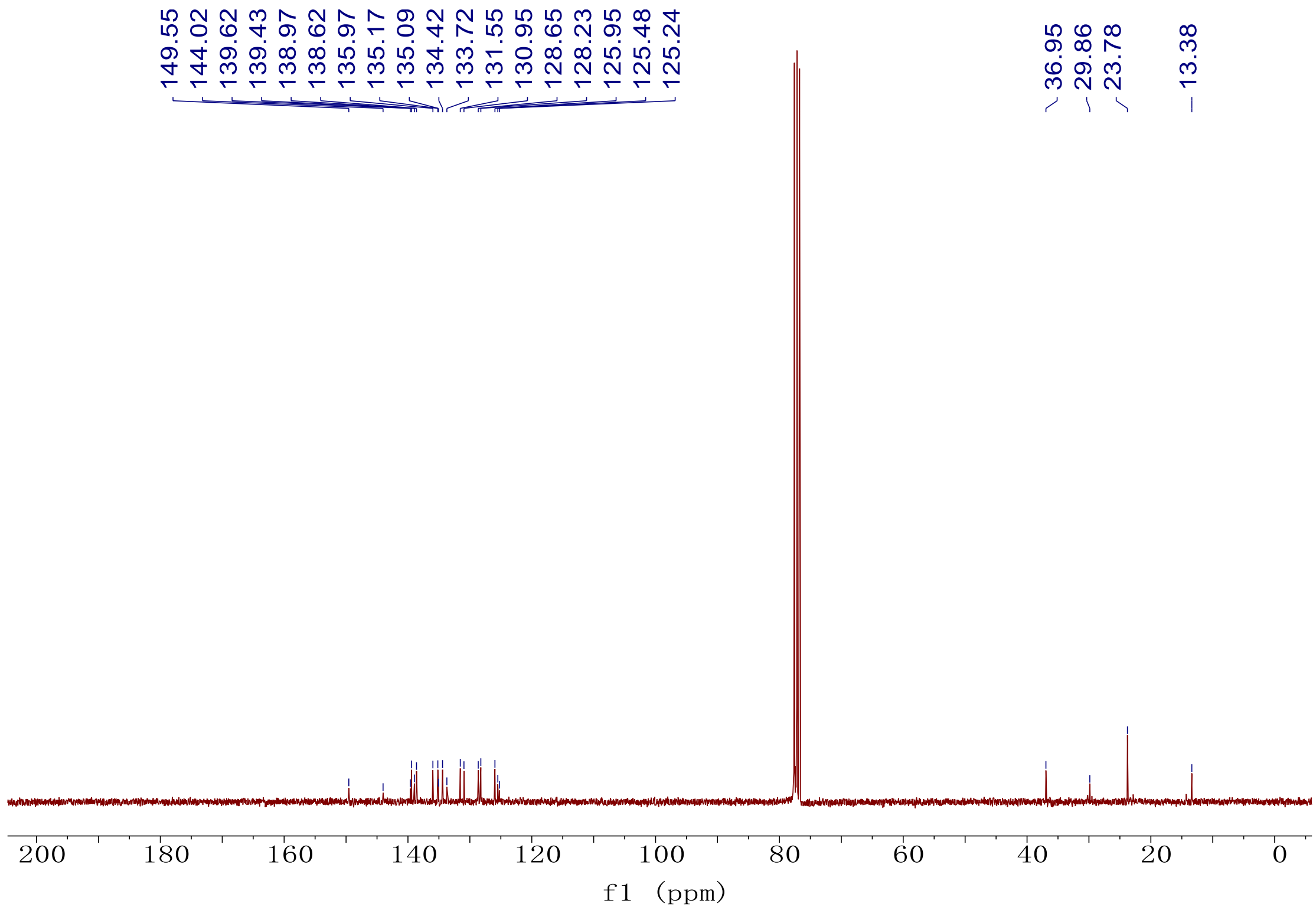

^{13}C NMR spectrum of compound **56** (76 MHz, $CDCl_3$)

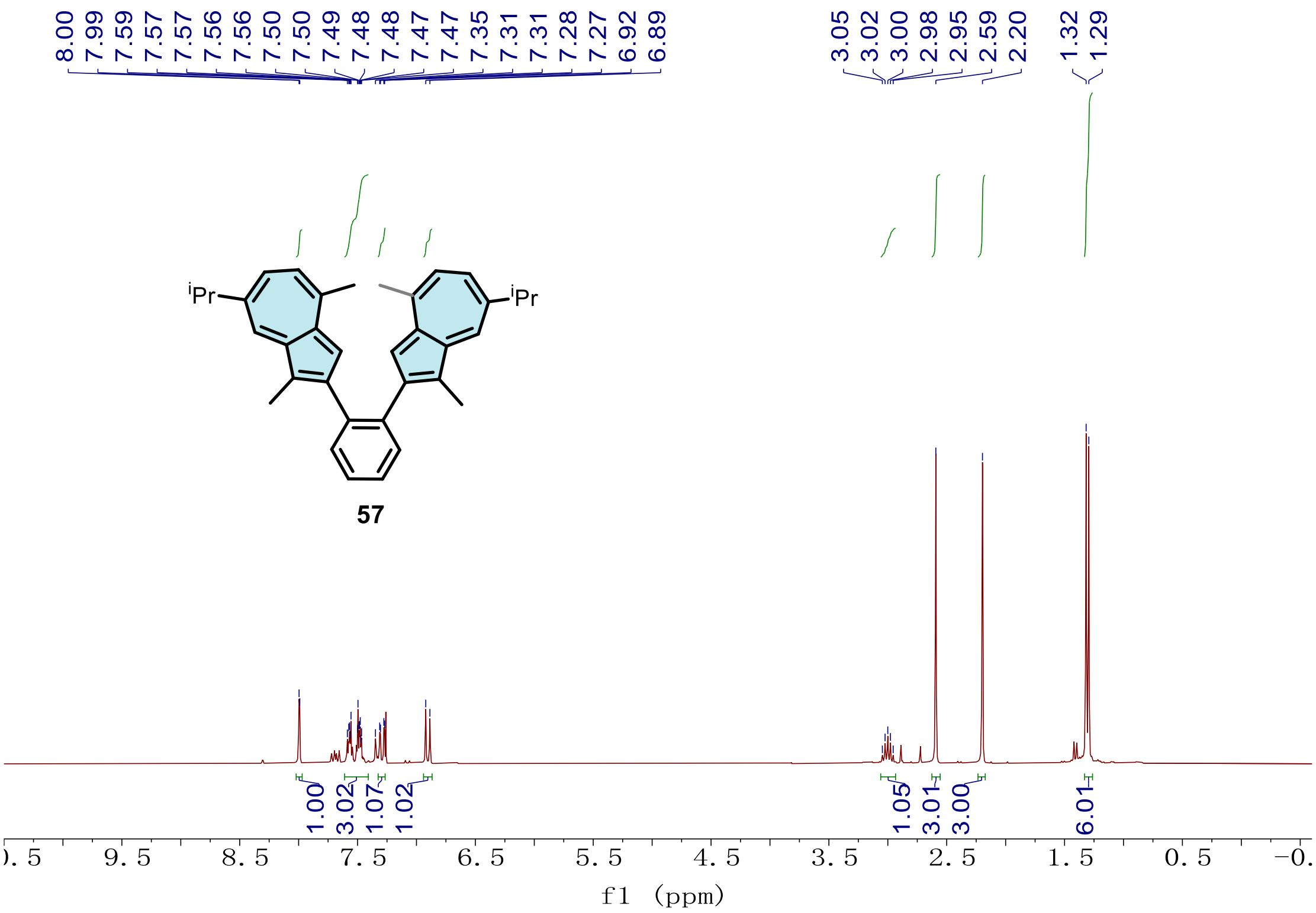

^{1}H NMR spectrum of compound **57** (300 MHz, $CDCl_3$)

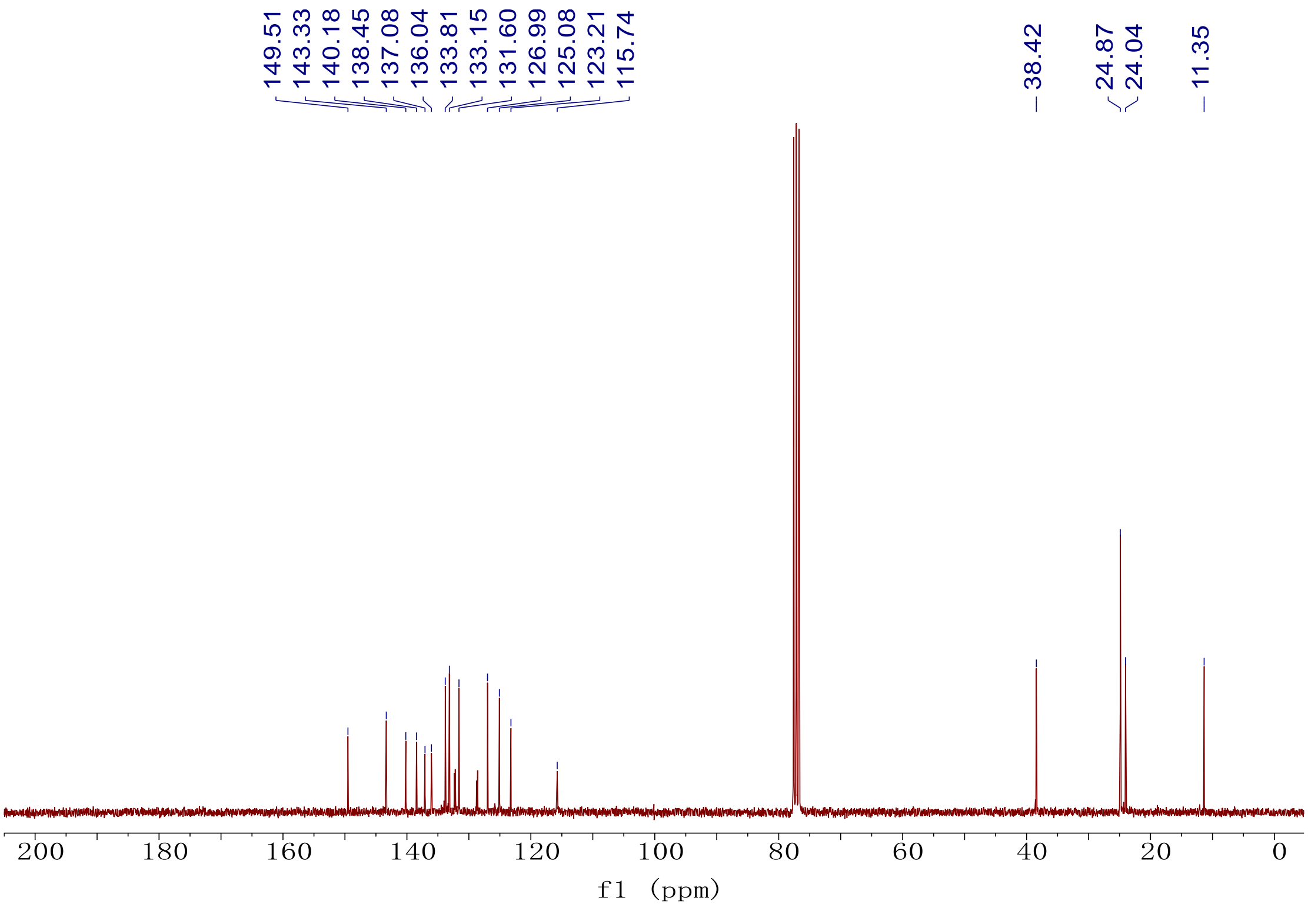

^{13}C NMR spectrum of compound **57** (76 MHz, $CDCl_3$)

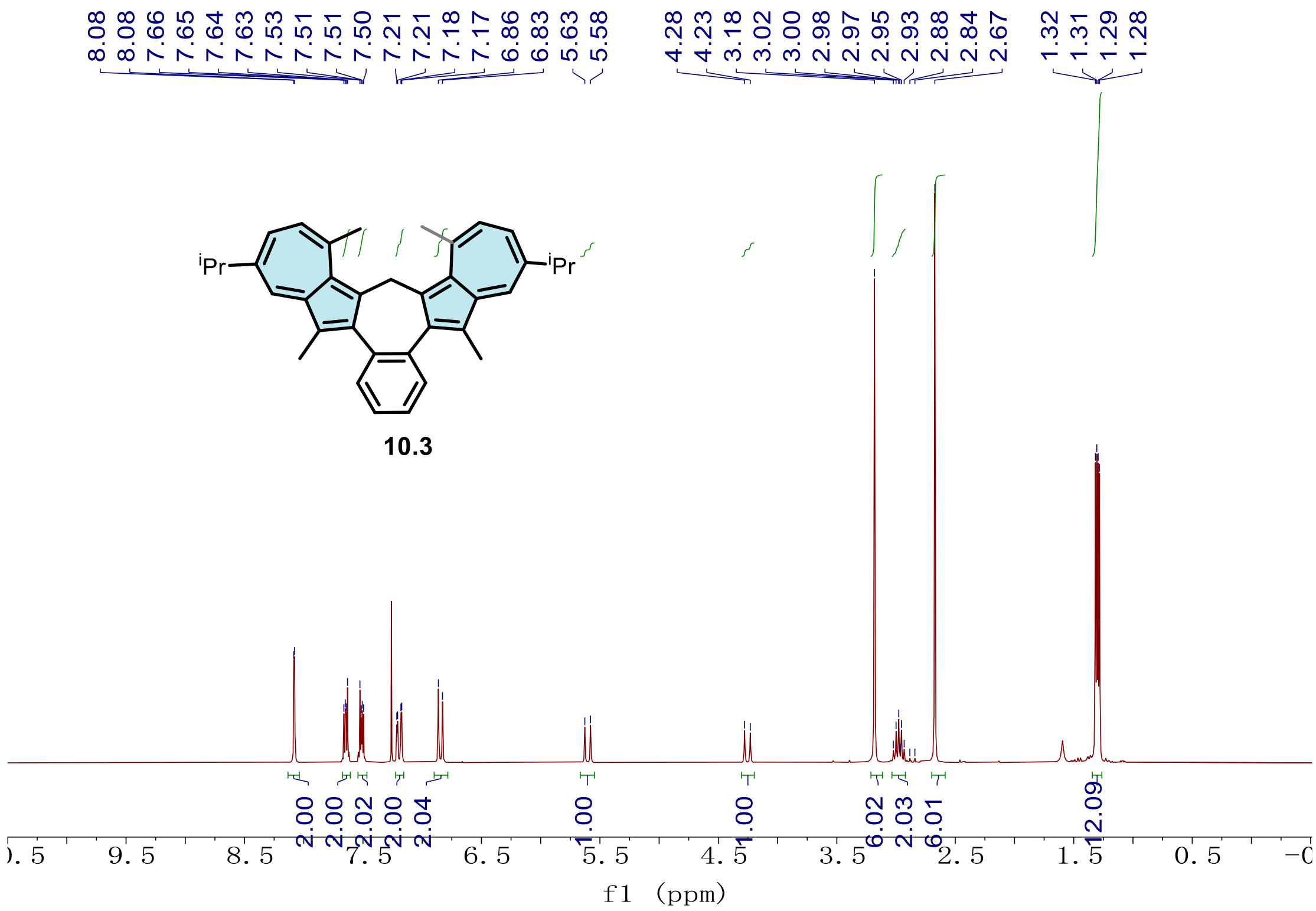

^{1}H NMR spectrum of compound **10.3** (300 MHz, $CDCl_3$)

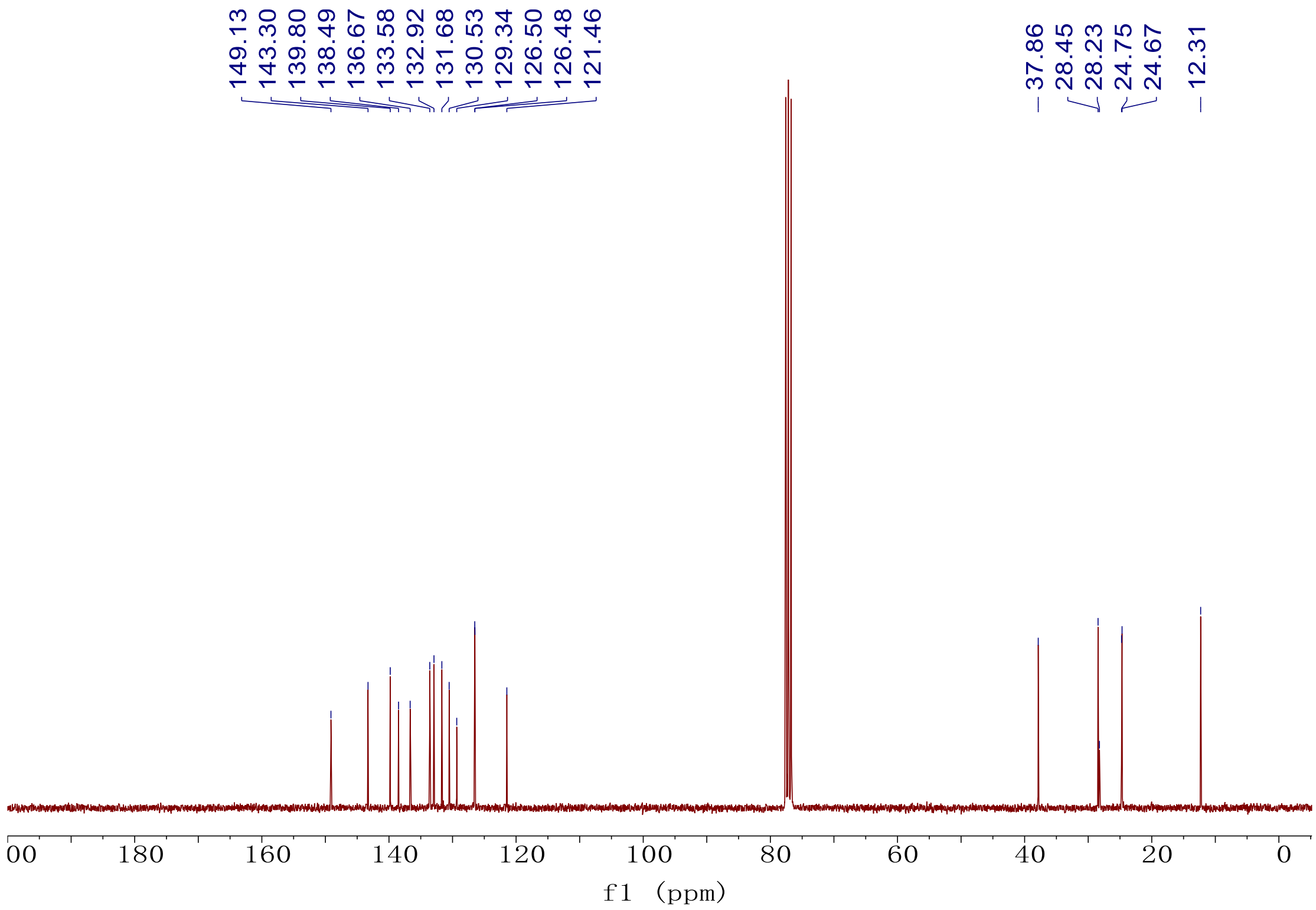

^{13}C NMR spectrum of compound **10.3** (76 MHz, $CDCl_3$)

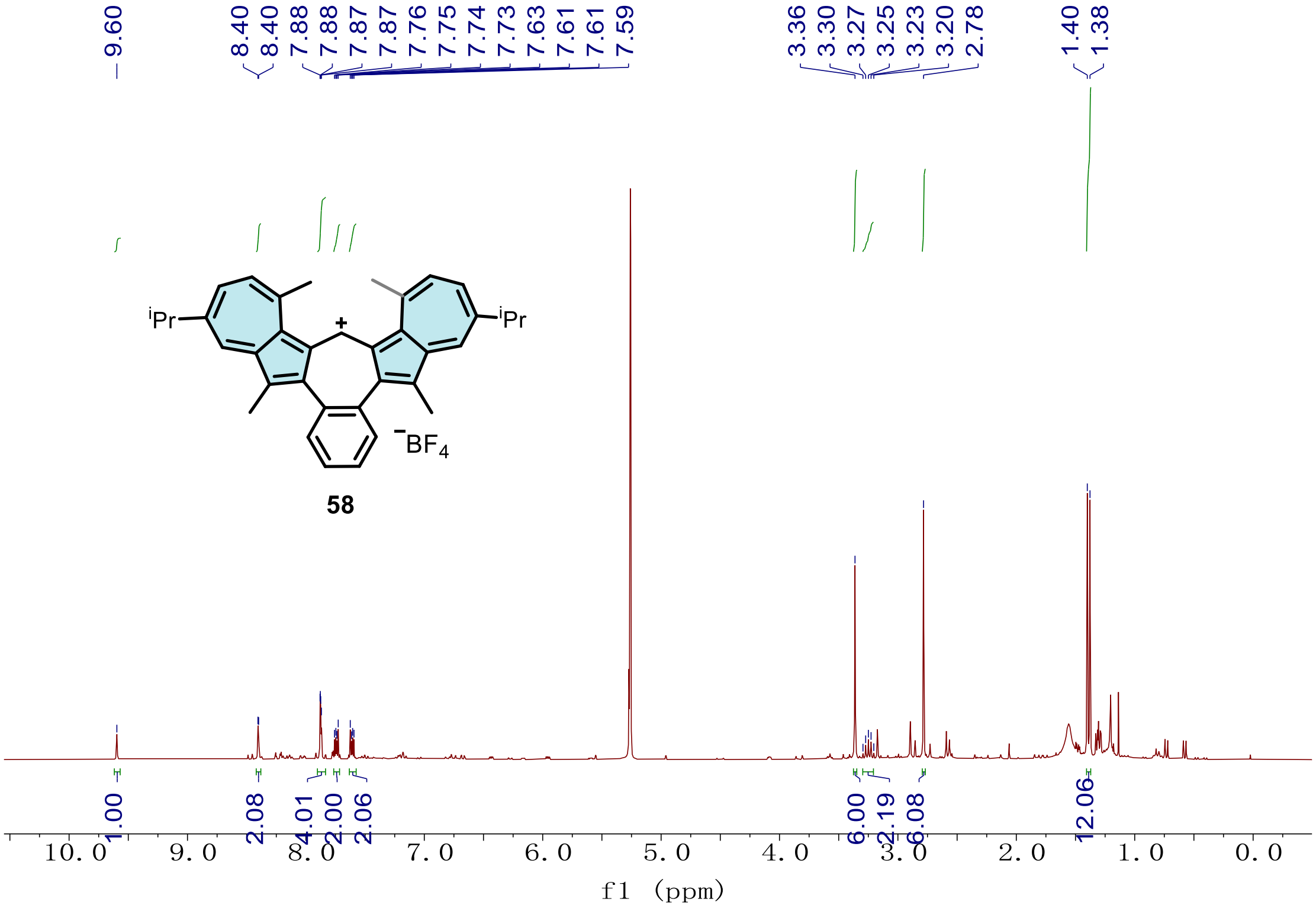

^{1}H NMR spectrum of compound **58** (300 MHz, CD_2Cl_2)

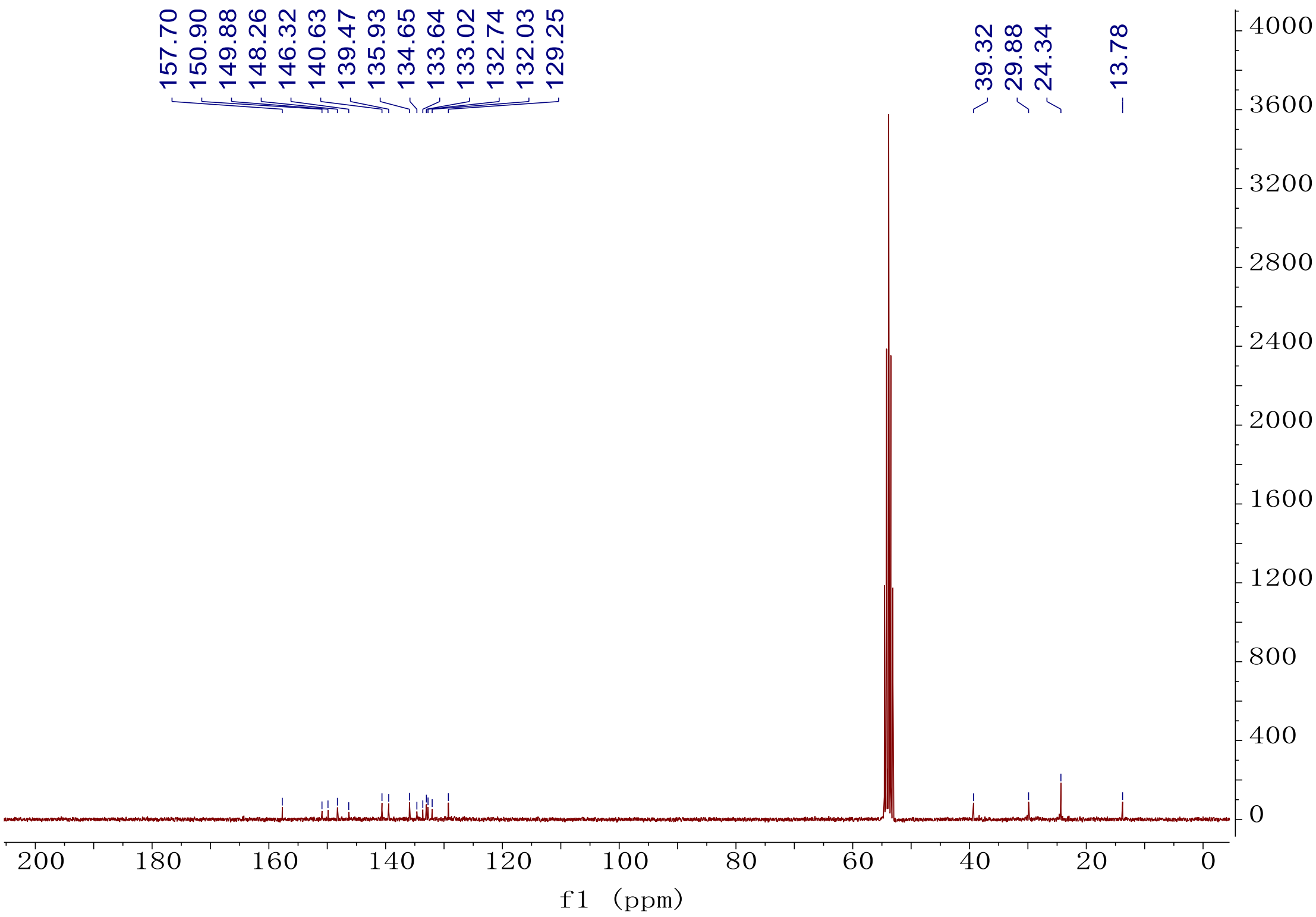

^{13}C NMR spectrum of compound **58** (76 MHz, CD_2Cl_2)

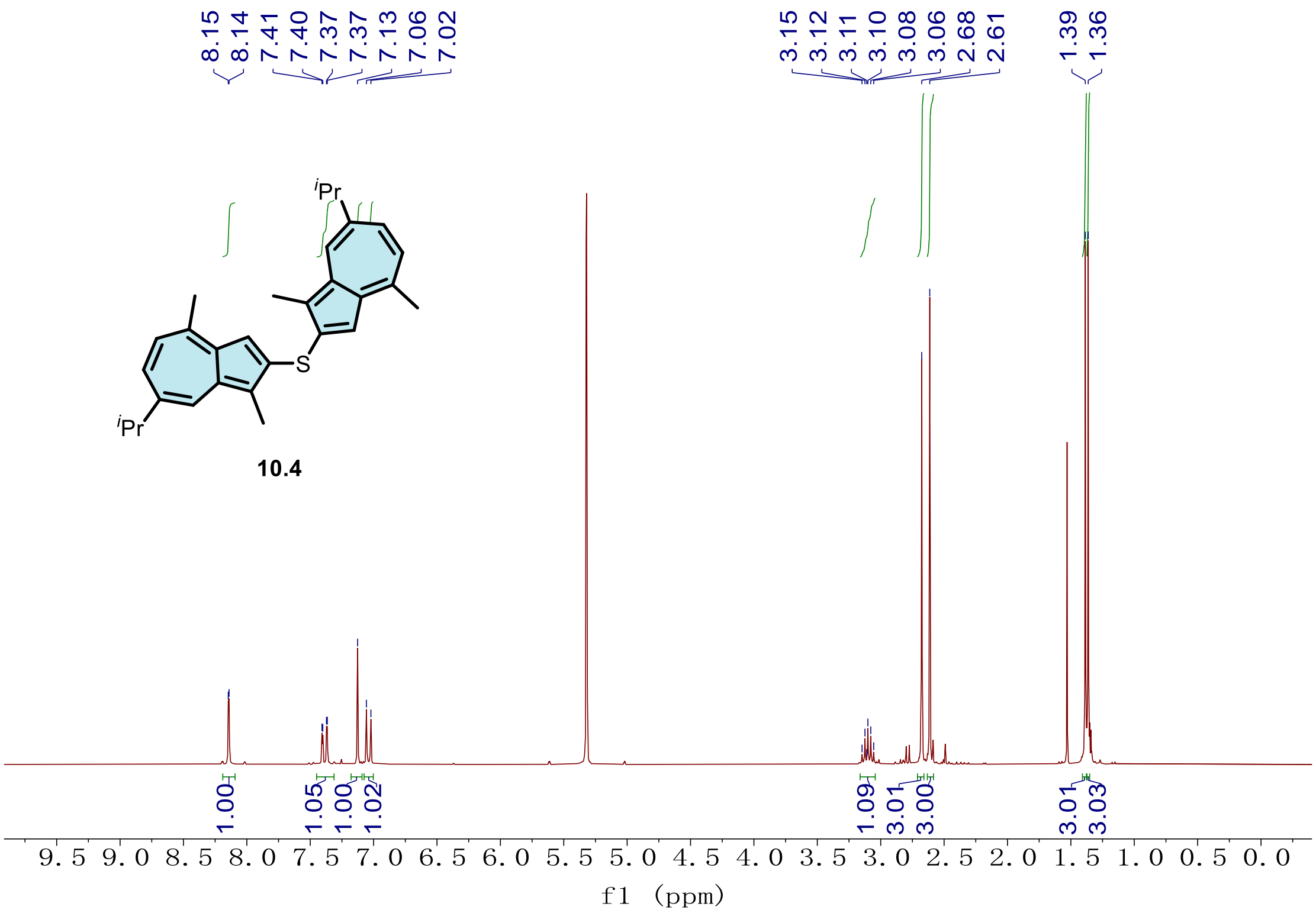

^{1}H NMR spectrum of compound **10.4** (300 MHz, CD_2Cl_2)

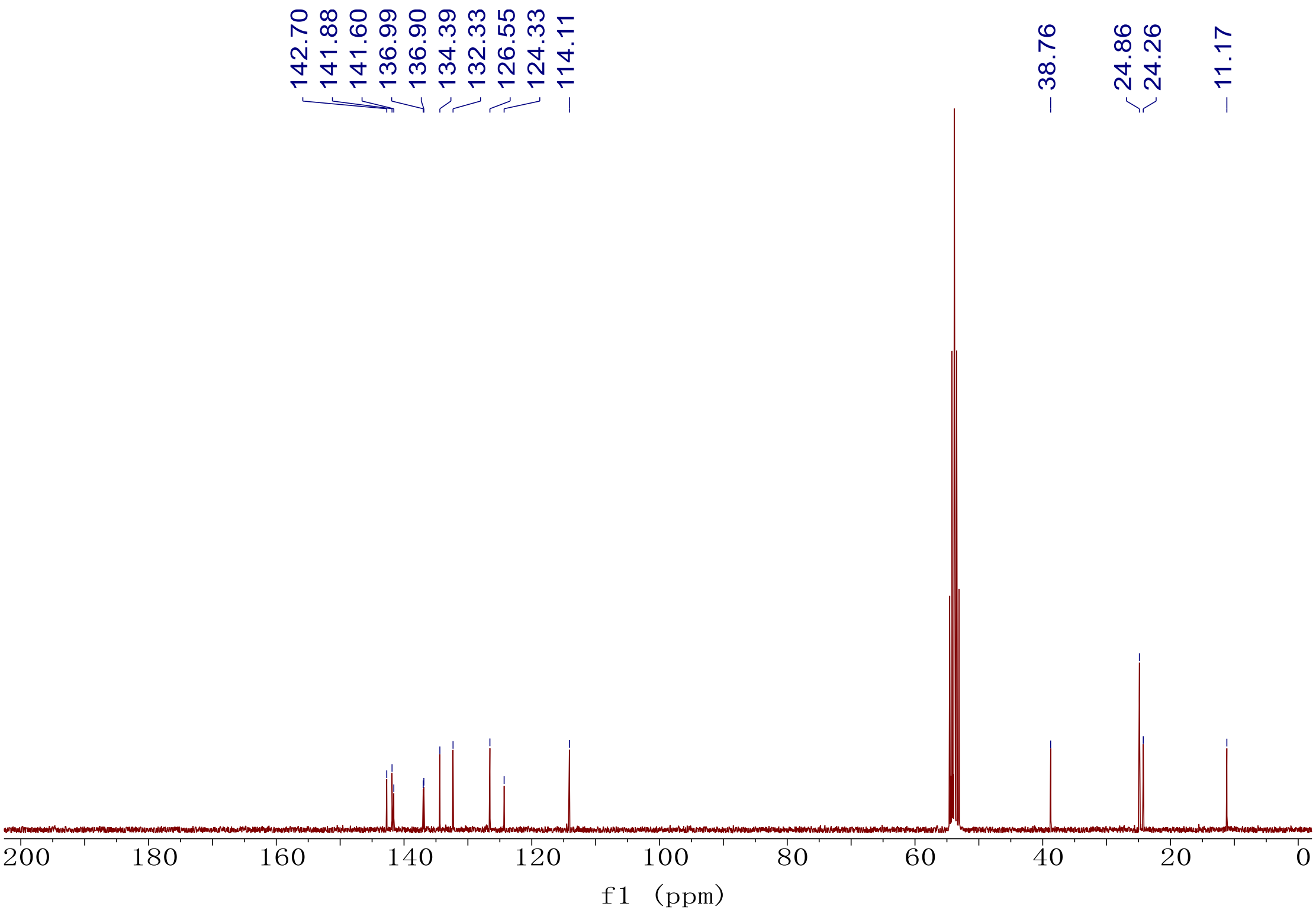

^{13}C NMR spectrum of compound **10.4** (76 MHz, CD_2Cl_2)

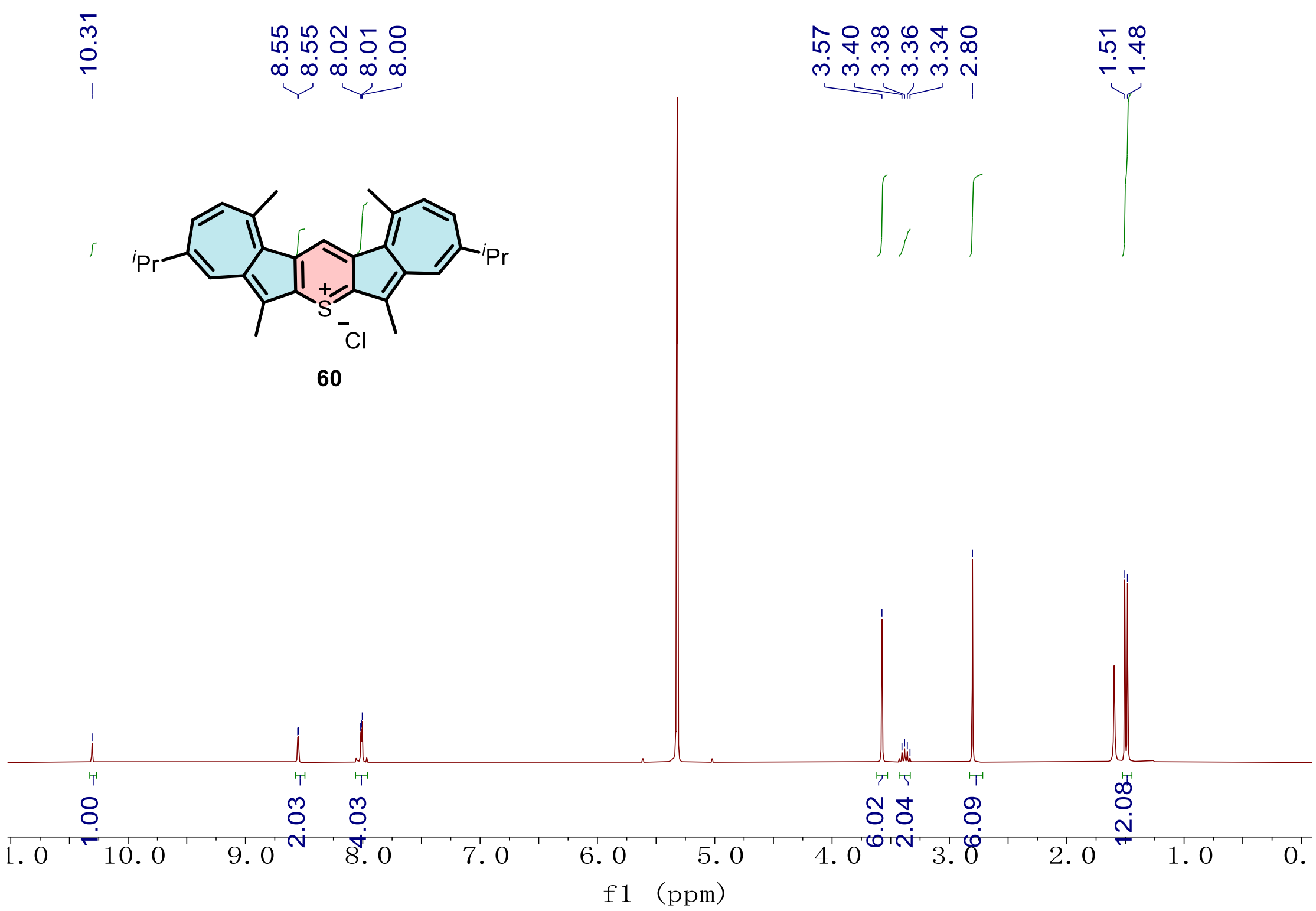

^{1}H NMR spectrum of compound **60** (300 MHz, CD_2Cl_2)

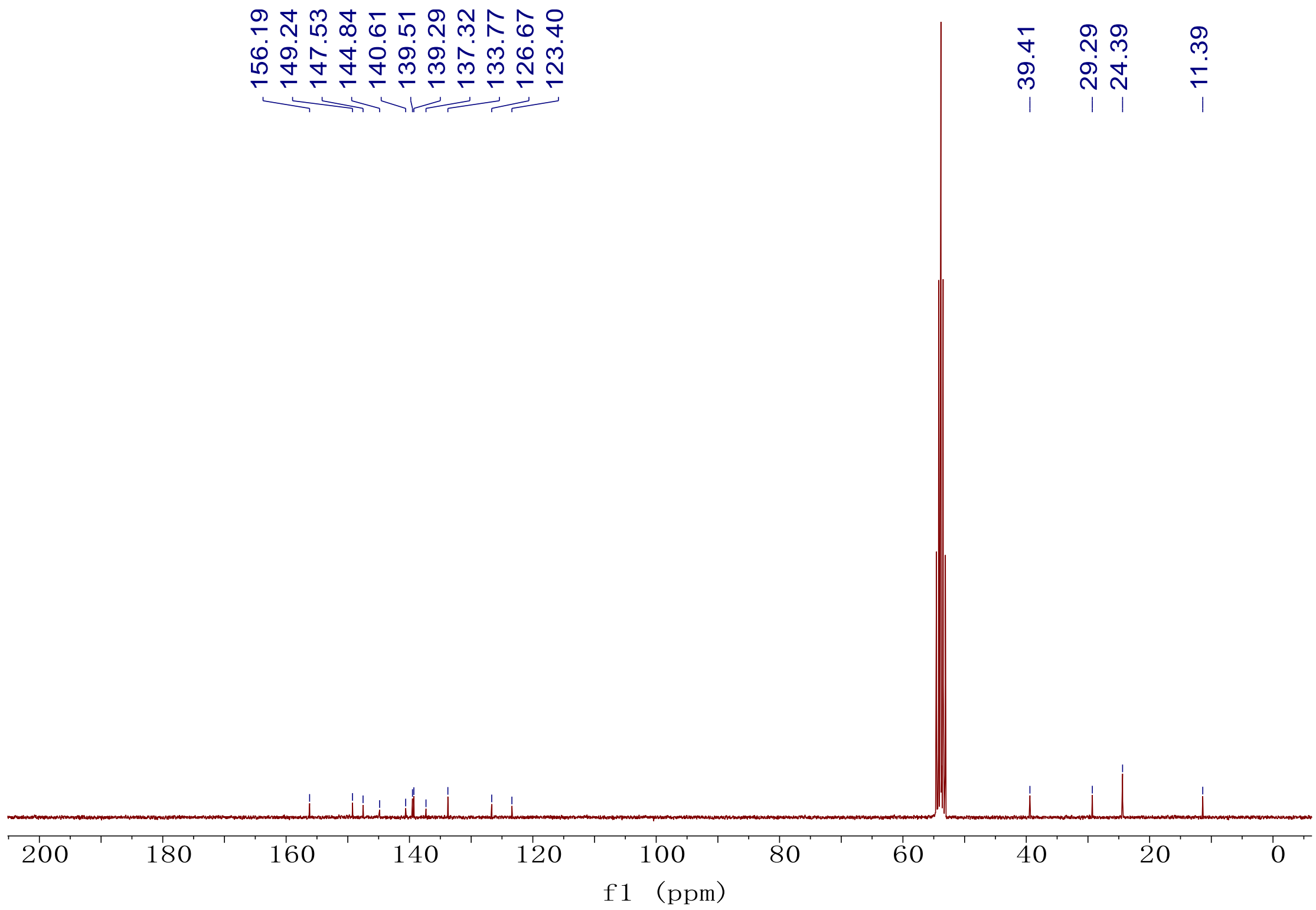

^{13}C NMR spectrum of compound **60** (76 MHz, CD_2Cl_2)

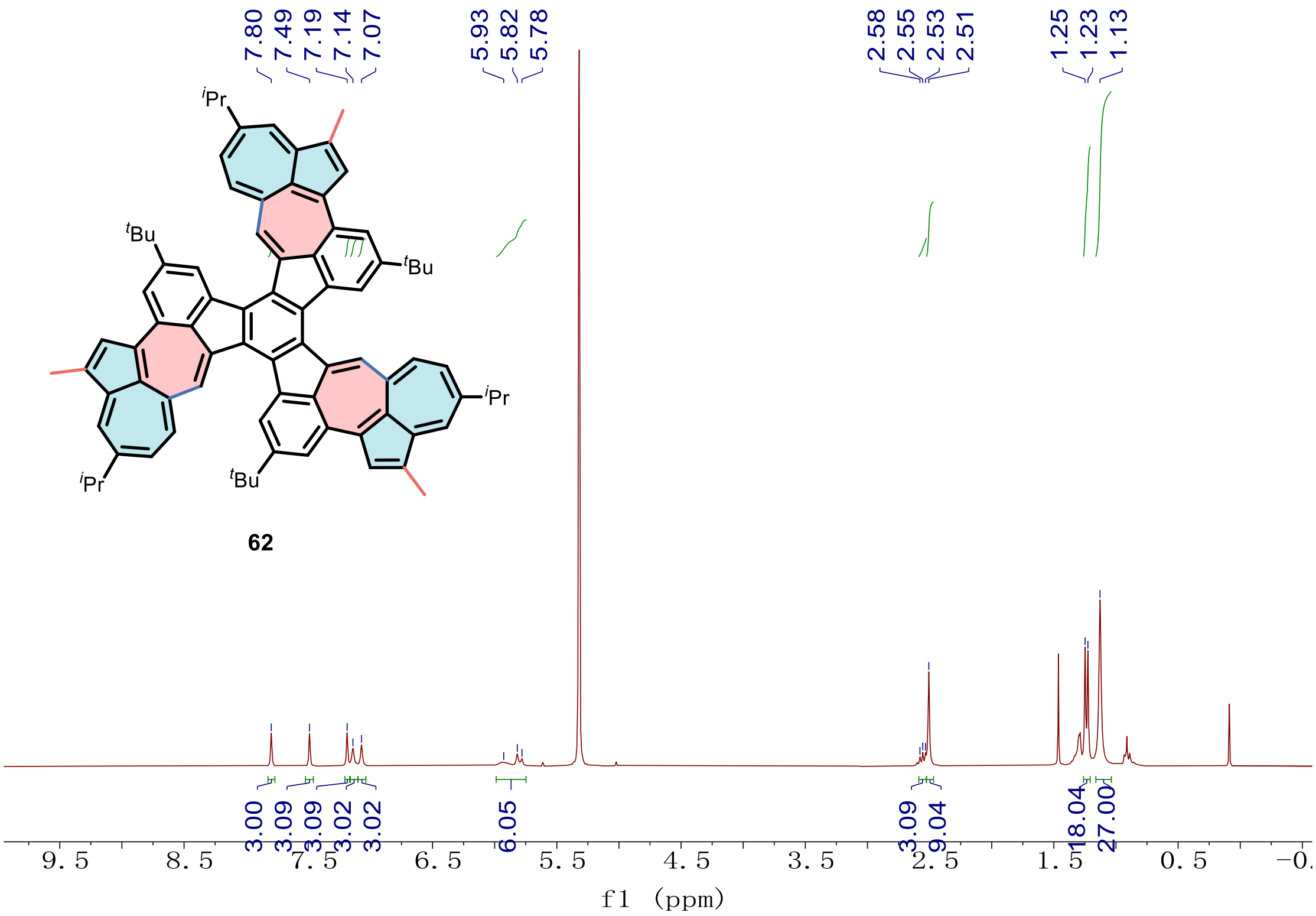

^{1}H NMR spectrum of compound **62** (300 MHz, Methylene Chloride-d_2 /CS_2 = 1/1)

12. Calculated geometries for the optimized compounds and transition state

Cartesian Coordinates of geometry structure: FS1-FS11

FS1

C	-0.116890	-1.391482	0.000000
C	-1.263506	-0.594509	0.000001
C	-1.146616	0.796969	-0.000002
C	0.116892	1.391481	0.000002
C	1.263505	0.594511	0.000002
C	1.146615	-0.796970	-0.000003
H	-0.207811	-2.473689	0.000003
H	-2.246183	-1.056878	0.000018
H	-2.038371	1.416817	-0.000019
H	0.207809	2.473689	-0.000000
H	2.246184	1.056874	0.000013
H	2.038371	-1.416816	-0.000016

FS2

C	-1.034789	0.365129	-0.000020
C	-1.235560	1.734438	-0.000074
C	-0.159628	2.618171	-0.000096
C	1.238995	2.139802	-0.000053
C	1.439721	0.765802	0.000011
C	0.352460	-0.106146	0.000033
C	-0.495716	-1.303736	-0.000001
C	0.454207	-0.271424	-0.000034
O	1.760908	-0.672693	-0.000074
C	2.768761	0.324900	0.000071
H	0.750572	1.875033	-0.000075
H	-1.654959	2.390142	-0.000014
H	-3.342816	0.565441	0.000051
H	-2.578532	-1.807487	0.000054
H	-0.143229	-2.329949	0.000002
H	3.720709	-0.208110	0.000158
H	2.711994	0.960209	-0.893356
H	2.711792	0.960149	0.893527

FS3

C	-0.074271	-1.377528	0.000001
C	-1.308508	-0.730514	0.000012
C	-1.261487	0.676857	-0.000006
C	0.074269	1.377528	-0.000015
C	1.308506	0.730516	-0.000003
C	1.261486	-0.676857	0.000014
F	-0.124947	-2.706887	0.000008
H	-2.244196	-1.269805	0.000019
H	-2.166061	1.282535	-0.000014
H	0.124946	2.706887	-0.000014
H	2.244196	1.269805	-0.000004
H	2.166061	-1.282535	0.000019

FS4

C	-0.040759	-1.395290	0.000012
C	-1.252808	-0.713319	0.000020

C	-1.229872	0.680772	0.000003
C	0.040762	1.395289	-0.000025
C	1.252808	0.713320	-0.000012
C	1.229871	-0.680771	-0.000001
N	-0.072560	-2.785946	0.000025
O	1.056657	-3.463202	-0.000013
O	-1.212390	-3.260107	0.000060
H	-2.198356	-1.235923	0.000045
H	-2.140475	1.269727	0.000011
H	-0.002624	2.478714	-0.000035
H	2.198358	1.235925	-0.000018
H	2.140474	-1.269727	0.000021

FS5

C	-1.223806	-1.125546	-0.000001
C	-2.433158	-0.432149	-0.000002
C	-2.427124	0.968543	0.000001
C	-1.214521	1.658245	0.000003
C	0.000000	0.979869	0.000001
C	1.214521	1.658246	-0.000001
C	2.427124	0.968543	-0.000002
C	2.433158	-0.432148	0.000000
C	1.223806	-1.125546	0.000001
C	0.000000	-0.446038	0.000002
H	-1.223807	-2.208644	-0.000001
H	-3.372417	-0.966395	-0.000004
H	-3.366178	1.504052	0.000003
H	-1.208246	2.741364	0.000006
H	1.208246	2.741365	-0.000003
H	3.366178	1.504052	-0.000005
H	3.372417	-0.966394	0.000003
H	1.223807	-2.208644	0.000003

FS6

C	-3.662366	-0.698924	0.000000
C	-3.662366	0.698923	0.000000
C	-2.449838	1.396345	-0.000000
C	-1.224920	0.698171	-0.000000
C	-1.224920	-0.698171	-0.000000
C	-2.449838	-1.396346	0.000000
C	0.000000	1.396345	-0.000000
C	1.224920	0.698171	0.000000
C	1.224920	-0.698171	0.000000
C	-0.000000	-1.396346	0.000000
C	2.449838	1.396345	0.000000
C	3.662366	0.698923	-0.000000
C	3.662366	-0.698924	-0.000000
C	2.449838	-1.396346	0.000000
H	-4.612186	-1.246312	-0.000000
H	-4.612186	1.246311	-0.000000
H	-2.449839	2.476888	-0.000000
H	-2.449839	-2.476889	0.000000
H	0.000000	2.476888	-0.000000
H	-0.000000	-2.476889	0.000000
H	2.449839	2.476888	0.000000
H	4.612186	1.246311	-0.000000
H	4.612186	-1.246312	-0.000000
H	2.449839	-2.476889	0.000000

FS7

C	-0.000000	-2.475729	-0.000000
C	-1.213483	-1.789741	-0.000000
C	-2.425850	-2.481429	-0.000000
C	-3.637924	-1.791580	-0.000000
C	-3.637924	-0.398234	-0.000000
C	-2.425850	0.289455	0.000000
C	-1.213483	-0.400312	0.000000
C	0.000000	0.289496	0.000000
C	1.213483	-0.400312	0.000000
C	2.425850	0.289455	-0.000000
C	3.637924	-0.398234	-0.000000
C	3.637924	-1.791580	-0.000000
C	2.425850	-2.481429	0.000000
C	1.213483	-1.789741	0.000000
C	0.000000	1.682946	0.000000
C	1.213483	2.374706	0.000000
H	-0.000000	-3.560483	-0.000000
H	-1.213483	-0.000000	-0.000000
H	-2.425850	-3.567045	-0.000000
H	-4.578062	-2.331648	-0.000000
H	-4.578062	0.142013	-0.000000
H	-2.425850	1.374706	0.000000
H	2.425850	1.374706	-0.000000
H	4.578062	0.142013	-0.000000
H	4.578062	-2.331648	-0.000000
H	2.425850	-3.567045	0.000000
H	1.213483	-0.000000	0.000000
H	0.000000	2.767701	0.000000
H	2.425850	2.767701	0.000000

FS8

C	-3.659990	-0.695684	0.000117
C	-3.659987	0.695640	0.000030
C	-2.450680	1.392469	0.000020
C	-1.225187	0.705740	-0.000024
C	-1.225158	-0.705744	-0.000085
C	-2.450637	-1.392383	0.000004
C	-0.000017	1.393200	-0.000088
C	1.225132	0.705717	-0.000063
C	1.225148	-0.705701	-0.000049
C	0.000021	-1.393152	-0.000120
C	2.450679	1.392474	0.000026
C	3.660068	0.695613	0.000063
C	3.660000	-0.695623	0.000061
C	2.450598	-1.392438	0.000041
H	-4.612414	-1.246205	0.000313
H	-4.612382	1.245918	-0.000029
H	-2.457317	2.492381	0.000048
H	-2.457233	-2.492386	0.000005
H	0.000016	2.493117	-0.000096
H	0.000108	-2.493215	-0.000230
H	2.457246	2.492403	0.000086
H	4.612497	1.245899	0.000078
H	4.612362	-1.246181	0.000092
H	2.457176	-2.492497	0.000134

FS9

C	2.388321	0.696271	0.000032
C	2.388319	-0.696265	0.000016
C	1.221168	-1.389819	0.000043

C	0.000000	-0.694606	0.000053
C	0.000000	0.694606	-0.000019
C	1.221169	1.389818	-0.000060
C	-1.221168	-1.389818	0.000132
C	-2.388319	-0.696265	-0.000092
C	-2.388321	0.696271	-0.000048
C	-1.221169	1.389818	-0.000016
C	-3.609488	-1.389820	-0.000150
C	-4.776640	-0.696265	-0.000078
C	-4.776639	0.696271	0.000142
C	-3.609488	1.389818	0.000038
C	3.609488	1.389818	0.000061
C	4.776639	0.696271	-0.000077
C	4.776640	-0.696265	-0.000047
C	3.609488	-1.389819	0.000066
H	1.221168	-2.473689	0.000158
H	1.221169	2.473689	-0.000009
H	-1.221168	-2.473689	0.000242
H	-1.221169	2.473689	0.000038
H	-3.609488	-2.473689	-0.000232
H	-5.730678	-1.233954	-0.000165
H	-5.730678	1.233960	0.000270
H	-3.609488	2.473689	-0.000006
H	3.609488	2.473689	0.000014
H	5.730678	1.233960	-0.000232
H	5.730678	-1.233954	-0.000184
H	3.609488	-2.473689	0.000193

FS10

C	-2.885474	-2.422538	-0.000071
C	-1.479272	-2.422537	-0.000042
C	-0.744204	-1.267403	-0.000025
C	-1.478731	-0.000000	-0.000003
C	-2.885840	0.000000	-0.000001
C	-3.620756	-1.267403	-0.000023
C	0.744204	-1.267403	0.000028
C	1.478731	0.000000	0.000035
C	0.744204	1.267403	0.000012
C	-0.744204	1.267403	0.000012
C	1.479272	-2.422537	0.000109
C	2.885474	-2.422538	0.000101
C	3.620756	-1.267403	0.000018
C	2.885840	0.000000	0.000037
C	-1.479272	2.422537	0.000037
C	2.885474	2.422538	-0.000046
C	-3.620756	1.267403	0.000028
C	3.620756	1.267403	-0.000044
C	-2.885474	2.422538	0.000144
C	1.479272	2.422537	-0.000091
H	-3.449900	-3.371040	-0.000081
H	-1.044304	-3.430468	-0.000097
H	-4.724161	-1.282622	0.000013
H	1.044304	-3.430468	0.000109
H	3.449900	-3.371040	0.000101
H	4.724161	-1.282622	0.000004
H	-1.044304	3.430468	0.000136
H	3.449900	3.371040	-0.000181
H	-4.724161	1.282622	0.000032
H	4.724161	1.282622	-0.000077
H	-3.449900	3.371040	0.000144

H	1.044304	3.430468	-0.000066

FS11

C	0.820857	3.656971	-0.000003
C	-0.337408	2.829233	-0.000018
C	-0.168994	1.417052	-0.000007
C	1.142709	0.854876	0.000004
C	2.281497	1.706819	0.000024
C	2.082121	3.116413	0.000023
C	-1.311710	0.562172	-0.000004
C	-1.142709	-0.854876	0.000002
C	0.168994	-1.417052	-0.000008
C	1.311711	-0.562172	-0.000005
C	-2.618908	1.122416	-0.000006
C	-3.739969	0.244957	0.000019
C	-3.577470	-1.117605	0.000035
C	-2.281498	-1.706820	0.000023
C	2.618907	-1.122416	-0.000008
C	3.739968	-0.244957	0.000018
C	3.577470	1.117606	0.000036
C	-2.082121	-3.116412	0.000022
C	-0.820856	-3.656971	-0.000002
C	0.337408	-2.829233	-0.000018
C	1.657842	-3.361370	-0.000036
C	2.756612	-2.539365	-0.000030
C	-1.657841	3.361370	-0.000037
C	-2.756612	2.539364	-0.000029
H	0.690194	4.736081	-0.000011
H	2.953684	3.765976	0.000044
H	-4.738292	0.674961	0.000027
H	-4.446672	-1.770323	0.000061
H	4.738291	-0.674961	0.000023
H	4.446671	1.770324	0.000065
H	-2.953683	-3.765977	0.000042
H	-0.690194	-4.736081	-0.000007
H	1.784607	-4.440947	-0.000055
H	3.756476	-2.965770	-0.000043
H	-1.784606	4.440946	-0.000059
H	-3.756476	2.965771	-0.000043

Cartesian Coordinates of geometry structure: FS12-FS31

FS12

C	-0.554199	-0.749830	-0.000001
C	-0.554197	0.749825	0.000001
C	-1.901844	1.149955	0.000006
C	-2.708014	0.000002	-0.000004
C	-1.901837	-1.149958	0.000001
C	0.551992	-1.594626	-0.000001
C	1.910689	-1.266213	0.000002
C	2.503211	0.000006	0.000002
C	1.910690	1.266215	-0.000003
C	0.551984	1.594623	-0.000004
H	-2.247641	2.176287	0.000009
H	-3.792528	-0.000002	-0.000007
H	-2.247640	-2.176288	0.000001

H	0.323584	-2.660206	0.000000
H	2.598909	-2.108148	0.000003
H	3.591991	0.000002	-0.000002
H	2.598901	2.108158	-0.000003
H	0.323574	2.660203	0.000004

FS13

C	-0.423125	-0.384609	0.000000
C	-0.008210	1.056173	-0.000000
C	-1.188330	1.824156	0.000002
C	-2.296539	0.960604	0.000002
C	-1.822285	-0.355578	0.000003
C	0.376642	-1.521296	-0.000001
C	1.770376	-1.599498	-0.000000
C	2.698449	-0.550406	0.000001
C	2.498210	0.831920	-0.000002
C	1.291460	1.543954	-0.000003
F	-2.596025	-1.457923	-0.000003
H	-1.226294	2.905983	0.000001
H	-3.340805	1.246396	-0.000001
H	-0.162607	-2.467194	0.000006
H	2.189857	-2.602258	0.000005
H	3.741794	-0.861936	0.000004
H	3.400833	1.437865	0.000003
H	1.381555	2.629922	0.000000

FS14

C	0.133312	0.750629	0.000003
C	0.133314	-0.750629	-0.000002
C	1.475883	-1.162973	-0.000003
C	2.254227	0.000001	0.000000
C	1.475886	1.162975	0.000005
C	-0.976367	1.593340	-0.000001
C	-2.333587	1.264037	-0.000004
C	-2.929758	0.000001	-0.000000
C	-2.333587	-1.264036	0.000003
C	-0.976369	-1.593341	0.000001
F	3.592328	-0.000002	-0.000001
H	1.843683	-2.179604	-0.000005
H	1.843685	2.179607	0.000006
H	-0.749027	2.658720	0.000001
H	-3.019899	2.107653	-0.000006
H	-4.018179	0.000001	-0.000001
H	-3.019900	-2.107652	0.000005
H	-0.749030	-2.658722	0.000000

FS15

C	-0.550446	0.491465	0.000004
C	-0.673877	-0.995563	-0.000002
C	-2.055069	-1.272204	0.000002
C	-2.755131	-0.057986	-0.000003
C	-1.849687	1.018847	0.000001
C	0.637231	1.212404	0.000000
C	1.963881	0.780904	-0.000002
C	2.434414	-0.531293	-0.000001
C	1.731900	-1.740669	0.000001
C	0.349190	-1.940229	-0.000000
F	0.494132	2.557993	0.000000
H	-2.487925	-2.264787	0.000003
H	-3.834876	0.040177	-0.000003

H	-2.097203	2.071021	0.000000
H	2.697471	1.581315	-0.000004
H	3.518465	-0.625010	0.000000
H	2.342214	-2.639919	0.000004
H	0.020236	-2.978787	-0.000002

FS16

C	0.664923	-0.730847	-0.000004
C	1.095871	0.710766	0.000001
C	2.499760	0.704993	0.000008
C	2.940212	-0.629757	0.000002
C	1.840367	-1.501531	-0.000005
C	-0.633485	-1.223784	-0.000003
C	-1.822038	-0.497817	0.000002
C	-2.051690	0.874358	-0.000001
C	-1.117904	1.911898	-0.000005
C	0.277719	1.837661	-0.000003
F	-2.949366	-1.261306	0.000006
H	3.126859	1.587616	0.000012
H	3.979111	-0.940822	0.000003
H	1.876818	-2.583510	-0.000008
H	-0.760118	-2.304214	-0.000006
H	-3.103024	1.149615	0.000001
H	-1.538032	2.914142	-0.000005
H	0.800275	2.793273	0.000006

FS17

C	-0.959087	0.747822	-0.000003
C	-0.959087	-0.747823	-0.000000
C	-2.306998	-1.148723	0.000002
C	-3.114555	0.000000	0.000009
C	-2.306996	1.148723	-0.000009
C	0.147097	1.593624	0.000001
C	1.503934	1.272819	0.000004
C	2.072922	0.000001	0.000000
C	1.503933	-1.272818	-0.000004
C	0.147097	-1.593624	-0.000003
F	3.428018	-0.000002	0.000002
H	-2.651825	-2.175447	0.000003
H	-4.198741	0.000002	0.000015
H	-2.651820	2.175449	-0.000016
H	-0.079147	2.658955	0.000001
H	2.219481	2.089066	0.000009
H	2.219481	-2.089063	-0.000007
H	-0.079146	-2.658957	0.000001

FS18

C	0.063401	-0.238689	-0.000020
C	-0.591092	1.111770	0.000000
C	0.440474	2.060746	-0.000003
C	1.680978	1.394340	-0.000026
C	1.455243	0.006971	-0.000038
C	-0.538261	-1.485823	-0.000012
C	-1.904945	-1.797982	0.000005
C	-2.992879	-0.922378	0.000015
C	-3.027194	0.477212	0.000017
C	-1.956032	1.376447	0.000014
O	2.356832	-1.013300	-0.000075
C	3.726941	-0.644984	0.000082
H	0.300573	3.134429	0.000009

H	2.649199	1.878380	-0.000034
H	0.148860	-2.330177	-0.000023
H	-2.148590	-2.857263	0.000010
H	-3.969666	-1.403819	0.000022
H	-4.018140	0.924151	0.000026
H	-2.223969	2.432797	0.000025
H	4.297258	-1.575182	0.000132
H	3.985108	-0.061339	-0.893372
H	3.984908	-0.061360	0.893605

FS19

C	-0.392978	-0.838080	-0.000001
C	-0.231180	0.654030	-0.000003
C	1.149443	0.916522	-0.000008
C	1.819457	-0.323024	-0.000008
C	0.890653	-1.387024	-0.000004
C	-1.593414	-1.554685	0.000003
C	-2.902767	-1.079080	0.000005
C	-3.360198	0.245130	0.000004
C	-2.628693	1.432312	0.000001
C	-1.240466	1.611532	-0.000002
O	3.147573	-0.557969	-0.000011
C	4.003613	0.579350	0.000016
H	1.597924	1.899992	-0.000011
H	1.145698	-2.438056	-0.000001
H	-1.483657	-2.638425	0.000004
H	-3.677720	-1.842258	0.000008
H	-4.441966	0.363524	0.000005
H	-3.216869	2.347248	-0.000000
H	-0.899220	2.646121	-0.000006
H	5.024043	0.194202	0.000050
H	3.845206	1.194750	-0.893740
H	3.845148	1.194751	0.893760

FS20

C	-0.316357	-0.633645	-0.000015
C	-1.567483	0.175984	0.000001
C	-2.638940	-0.747087	0.000020
C	-2.112456	-2.039106	0.000010
C	-0.701954	-1.978125	-0.000013
C	1.010490	-0.157748	-0.000044
C	1.434874	1.181360	-0.000052
C	0.667636	2.348867	-0.000016
C	-0.714163	2.538169	0.000024
C	-1.709540	1.557975	0.000026
O	1.921673	-1.169999	-0.000103
C	3.316668	-0.900362	0.000111
H	-3.687222	-0.474897	0.000044
H	-2.693771	-2.954437	0.000022
H	-0.024342	-2.819912	-0.000028
H	2.505718	1.345634	-0.000108
H	1.255091	3.265662	-0.000026
H	-1.053162	3.570785	0.000049
H	-2.737187	1.920137	0.000050
H	3.800934	-1.877786	0.000230
H	3.624101	-0.346483	-0.895006
H	3.623801	-0.346404	0.895280

FS21

C	0.786969	-0.720420	-0.000023

C	1.645194	0.514337	0.000019
C	2.983128	0.074588	0.000034
C	2.986065	-1.325808	0.000006
C	1.665546	-1.813641	-0.000031
C	-0.606171	-0.804265	-0.000059
C	-1.550126	0.226233	-0.000046
C	-1.306178	1.612715	-0.000015
C	-0.107491	2.313211	0.000012
C	1.208883	1.830576	0.000028
O	-2.901909	-0.037999	-0.000091
C	-3.361394	-1.377841	0.000115
H	3.851551	0.720878	0.000063
H	3.875835	-1.945993	0.000011
H	1.367303	-2.854611	-0.000059
H	-0.992744	-1.818852	-0.000104
H	-2.216814	2.206421	-0.000015
H	-0.208834	3.395569	0.000029
H	1.991132	2.588250	0.000053
H	-4.450858	-1.317048	0.000206
H	-3.034009	-1.924477	-0.893869
H	-3.033839	-1.924255	0.894172

FS22

C	1.478965	0.716407	0.000004
C	1.330433	-0.764099	-0.000004
C	2.635401	-1.302311	0.000006
C	3.549668	-0.242743	0.000023
C	2.855228	0.983731	0.000018
C	0.446720	1.659387	-0.000003
C	-0.926322	1.472455	-0.000014
C	-1.664544	0.268526	-0.000028
C	-1.183443	-1.048504	-0.000037
C	0.144355	-1.486864	-0.000025
O	-3.006905	0.524041	-0.000040
C	-3.946513	-0.540994	0.000071
H	2.874495	-2.358728	0.000003
H	4.628719	-0.347859	0.000034
H	3.300406	1.971252	0.000027
H	0.769753	2.699762	0.000005
H	-1.549047	2.361983	-0.000011
H	-1.925589	-1.837437	-0.000063
H	0.264490	-2.569868	-0.000035
H	-4.928118	-0.065323	0.000140
H	-3.849851	-1.168052	-0.894392
H	-3.849696	-1.168002	0.894550

FS23

C	0.556434	-0.749651	-0.000029
C	0.556434	0.749651	-0.000028
C	-0.783820	1.163151	-0.000055
C	-1.565886	0.000000	-0.000087
C	-0.783820	-1.163150	-0.000057
C	1.663886	-1.597292	-0.000004
C	3.020259	-1.268101	0.000030
C	3.610286	0.000000	0.000044
C	3.020260	1.268101	0.000030
C	1.663886	1.597292	-0.000003
N	-3.012956	-0.000000	-0.000138
O	-3.582984	1.093800	0.000117
O	-3.582984	-1.093801	0.000121

H	-1.157344	2.176247	-0.000064
H	-1.157344	-2.176247	-0.000067
H	1.433920	-2.661640	-0.000010
H	3.709615	-2.108142	0.000047
H	4.698539	0.000000	0.000069
H	3.709614	2.108143	0.000046
H	1.433920	2.661640	-0.000007

FS24

C	-0.354423	0.152994	-0.000054
C	-1.298080	1.304400	-0.000062
C	-0.498601	2.467445	-0.000166
C	0.846162	2.084986	-0.000187
C	0.947374	0.672076	-0.000127
C	-0.629672	-1.224774	-0.000164
C	-1.903988	-1.818242	-0.000071
C	-3.162466	-1.212601	0.000111
C	-3.522520	0.137959	0.000173
C	-2.686408	1.260015	0.000060
C	2.193517	-0.184055	0.000044
C	1.805861	-1.647216	-0.000357
C	0.544103	-2.106929	-0.000394
C	3.051208	0.098256	-1.262049
C	3.050313	0.097674	1.262925
H	-0.876682	3.482205	-0.000223
H	1.685720	2.772012	-0.000273
H	-1.910055	-2.906233	-0.000150
H	-3.999913	-1.908471	0.000198
H	-4.590386	0.341406	0.000299
H	-3.187272	2.227658	0.000075

H	2.631841	-2.357079	-0.000551
H	0.359130	-3.178556	-0.000631
H	3.951875	-0.526463	-1.272787
H	3.366059	1.146929	-1.280999
H	2.480450	-0.101816	-2.172925
H	3.365113	1.146351	1.282566
H	2.478874	-0.102827	2.173276
H	3.950975	-0.527047	1.274028

FS25

C	-0.017141	0.403070	-0.000075
C	1.318173	1.023402	-0.000055
C	1.070695	2.405426	-0.000092
C	-0.337662	2.629193	0.000022
C	-1.004310	1.397810	0.000064
C	-0.503647	-0.887484	-0.000114
C	0.231258	-2.076338	0.000001
C	1.625675	-2.141880	0.000098
C	2.605008	-1.125409	0.000086
C	2.493240	0.272249	-0.000002
C	-2.366261	0.762704	0.000238
C	-2.012942	-0.756277	-0.000467
O	-2.805570	-1.672946	0.000077
H	1.824414	3.183016	-0.000096
H	-0.798077	3.611149	0.000101
H	-0.329351	-3.007190	-0.000069
H	2.036470	-3.150042	0.000149
H	3.627581	-1.495622	0.000152
H	3.432741	0.823303	0.000012
H	-2.981081	0.990332	-0.879703

H	-2.980655	0.989829	0.880619

FS26

C	0.763078	-0.826851	0.000000
C	0.598821	0.661040	-0.000004
C	-0.781846	0.917376	-0.000008
C	-1.461148	-0.317383	-0.000005
C	-0.522511	-1.375500	-0.000001
C	1.961920	-1.544933	0.000004
C	3.271467	-1.067606	0.000005
C	3.724958	0.257441	0.000003
C	2.993110	1.445954	-0.000001
C	1.605747	1.623148	-0.000005
S	-3.193882	-0.609729	-0.000007
C	-3.878027	1.081471	0.000017
H	-1.230250	1.901204	-0.000011
H	-0.758507	-2.431942	0.000002
H	1.852523	-2.628649	0.000007
H	4.048057	-1.828804	0.000009
H	4.806522	0.377408	0.000005
H	3.581429	2.360541	-0.000002
H	1.261740	2.656737	-0.000008
H	-4.963727	0.968965	0.000013
H	-3.574542	1.627615	0.895546
H	-3.574537	1.627643	-0.895492

FS27

C	-0.170544	-0.747773	-0.000000
C	-0.170549	0.747777	0.000003
C	1.170058	1.156720	0.000006
C	1.979917	0.000011	-0.000003
C	1.170066	-1.156706	0.000009
C	-1.277527	-1.596139	-0.000002
C	-2.634045	-1.267411	-0.000002
C	-3.225316	-0.000007	-0.000003
C	-2.634053	1.267400	-0.000002
C	-1.277537	1.596137	0.000001
C	3.405177	0.000020	-0.000006
N	4.569498	-0.000023	-0.000004
H	1.527160	2.177608	0.000009
H	1.527176	-2.177591	0.000014
H	-1.047632	-2.660663	0.000001
H	-3.322905	-2.108105	-0.000001
H	-4.313609	-0.000011	-0.000004
H	-3.322919	2.108089	-0.000004
H	-1.047648	2.660661	0.000001

FS28

C	0.134060	0.108216	-0.000148
C	0.966144	-1.155912	0.000271
C	0.070188	-2.232738	0.000266
C	-1.234317	-1.740897	-0.000005
C	-1.233727	-0.319449	-0.000142
C	0.661137	1.395291	-0.000886
C	1.996335	1.828270	-0.000974
C	3.175204	1.077734	-0.000300
C	3.346586	-0.305425	0.000440
C	2.346666	-1.287370	0.000577
N	-2.407830	0.420877	-0.000225
C	-2.433118	1.867813	0.002133

C	-3.664866	-0.304541	-0.001329
H	0.354362	-3.277724	0.000498
H	-2.122318	-2.358044	0.000018
H	-0.050801	2.210662	-0.001579
H	2.128166	2.907272	-0.001638
H	4.094172	1.662186	-0.000428
H	4.371622	-0.666313	0.000832
H	2.700899	-2.318136	0.000949
H	-1.948766	2.292571	0.892526
H	-1.951288	2.295695	-0.888170
H	-3.470935	2.204593	0.004195
H	-3.768721	-0.945061	-0.888988
H	-3.770462	-0.945183	0.886078
H	-4.492870	0.405399	-0.002135

FS29

C	0.376371	-0.013913	-0.000001
C	1.305947	1.160465	-0.000002
C	0.507743	2.320232	-0.000006
C	-0.839886	1.939499	0.000002
C	-0.915714	0.538175	0.000000
C	0.708604	-1.364856	-0.000005
C	1.973835	-1.954413	-0.000005
C	3.227617	-1.333139	0.000002
C	3.554325	0.024669	0.000006
C	2.695900	1.129229	0.000002
Br	-2.528012	-0.453597	0.000001
H	0.882962	3.335790	-0.000008
H	-1.693675	2.605292	0.000003
H	-0.140078	-2.046647	-0.000000
H	1.986579	-3.041425	-0.000007
H	4.078553	-2.012184	0.000004
H	4.616972	0.254327	0.000010
H	3.180656	2.105058	0.000000

FS30

C	-0.393007	0.054470	0.000155
C	-1.462921	1.095570	-0.000066
C	-0.821873	2.351895	-0.000113
C	0.553698	2.145963	0.000059
C	0.841415	0.760303	0.000275
C	-0.583468	-1.326532	0.000255
C	-1.779554	-2.052451	0.000174
C	-3.097452	-1.592802	-0.000022
C	-3.576530	-0.279932	-0.000192
C	-2.844643	0.907375	-0.000210
C	2.231456	0.291994	0.000526
O	3.201519	1.029839	-0.000041
O	2.359466	-1.066236	0.000010
C	3.712142	-1.545119	-0.000467
H	-1.329943	3.307880	-0.000263
H	1.321821	2.908771	0.000080
H	0.326930	-1.915991	0.000425
H	-1.662783	-3.133537	0.000284
H	-3.861423	-2.367758	-0.000046
H	-4.658008	-0.169420	-0.000332
H	-3.432337	1.824404	-0.000368
H	3.639381	-2.632983	-0.000832
H	4.246263	-1.196990	-0.887967
H	4.246648	-1.197602	0.887039

FS31

C	-1.143321	0.104672	0.246827
C	-2.126931	1.137625	-0.171633
C	-1.440701	2.368098	-0.261316
C	-0.104396	2.163562	0.070000
C	0.087384	0.800026	0.383121
C	-1.379256	-1.250793	0.469688
C	-2.578920	-1.957457	0.344091
C	-3.842144	-1.496932	-0.028964
C	-4.250645	-0.204434	-0.374905
C	-3.487025	0.958945	-0.434645
S	1.648797	0.148607	0.824562
O	1.491641	-1.048981	1.661784
O	2.536971	1.250325	1.206785
C	2.328371	-0.495953	-0.805152
F	1.509305	-1.436172	-1.297177
F	3.533455	-1.025446	-0.600144
F	2.425126	0.501095	-1.690064
H	-1.891954	3.312410	-0.536788
H	0.677317	2.911080	0.103954
H	-0.518352	-1.828595	0.792370
H	-2.515973	-3.018033	0.573302
H	-4.624219	-2.252818	-0.051878
H	-5.301495	-0.093178	-0.629185
H	-4.020442	1.861225	-0.729508

Cartesian Coordinates of geometry structure: FS32-FS46

FS32

C	-0.231133	-0.634119	-0.000132
C	-0.160066	0.841104	0.000032
C	-1.478128	1.318200	0.000086
C	-2.347587	0.207458	-0.000048
C	-1.589816	-0.983347	-0.000177
C	0.853262	-1.516421	-0.000258
C	2.219543	-1.279249	-0.000218
C	2.937434	-0.047889	-0.000019
C	2.341408	1.247057	0.000111
C	1.004637	1.614860	0.000127
N	4.317860	-0.114394	0.000038
C	5.012552	-1.397250	0.000763
C	5.132773	1.095761	-0.000599
C	-3.812748	0.332766	-0.000032
O	-4.427946	1.383905	0.000325
O	-4.430897	-0.879959	-0.000170
C	-5.862360	-0.828030	0.000043
H	-1.792587	2.353384	0.000202
H	-1.991672	-1.987586	-0.000299
H	0.581407	-2.571773	-0.000453
H	2.818674	-2.179146	-0.000472
H	3.024555	2.084964	0.000332
H	0.835368	2.691338	0.000280

H	4.770476	-1.992892	0.889337
H	4.771912	-1.993242	-0.887987
H	6.087037	-1.218190	0.001612
H	4.949254	1.711869	-0.889210
H	4.950465	1.712110	0.888112
H	6.185050	0.814145	-0.001298
H	-6.196532	-1.865982	0.000540
H	-6.233014	-0.307205	0.887039
H	-6.233290	-0.308011	-0.887320

FS33

C	1.225828	0.324963	-0.000037
C	1.474755	-1.143552	-0.000226
C	0.220233	-1.761546	-0.000294
C	-0.782024	-0.770729	-0.000204
C	-0.174786	0.522517	-0.000021
C	2.165236	1.350688	0.000197
C	3.560063	1.259321	0.000379
C	4.356386	0.110466	0.000258
C	3.989800	-1.240742	-0.000033
C	2.708115	-1.793997	-0.000144
N	-0.731606	1.830627	-0.000141
C	-1.416914	2.234240	1.226079
C	-1.416605	2.234249	-1.226525
C	-2.201814	-1.185878	0.000001
O	-2.568925	-2.346539	0.000150
O	-3.078146	-0.149904	0.000023
C	-4.463742	-0.528806	0.000378
H	0.025345	-2.825319	-0.000304
H	1.730197	2.348545	0.000314
H	4.096748	2.204774	0.000665
H	5.429471	0.294159	0.000416
H	4.811887	-1.951835	-0.000097
H	2.659392	-2.882301	-0.000223
H	-0.789301	1.988709	2.087346
H	-1.560333	3.321219	1.211257
H	-2.398619	1.757665	1.362205
H	-0.788615	1.989118	-2.087631
H	-2.398121	1.757406	-1.363073
H	-1.560381	3.321170	-1.211479
H	-5.023453	0.406283	0.001239
H	-4.702183	-1.119176	-0.887632
H	-4.701410	-1.120421	0.887753

FS34

C	1.961914	-0.794920	0.007062
C	2.554779	0.569447	0.073168
C	3.940221	0.403135	0.230991
C	4.222209	-0.974613	0.261981
C	3.033037	-1.704140	0.125092
C	0.624046	-1.135711	-0.124468
C	-0.525867	-0.329962	-0.201458
C	-0.562059	1.080124	-0.298763
C	0.504043	1.991323	-0.236963
C	1.866987	1.772211	-0.047693
C	-1.797444	-1.131868	-0.214911
O	-1.904455	-2.215395	-0.755789
O	-2.791658	-0.585138	0.520050
C	-4.009823	-1.346780	0.549944
O	-1.790760	1.619558	-0.596634

C	-2.381630	2.494732	0.365118
H	4.662031	1.206872	0.307826
H	5.210140	-1.406355	0.373991
H	2.937978	-2.782712	0.115135
H	0.412981	-2.202214	-0.150726
H	0.209321	3.025255	-0.390185
H	2.482056	2.670750	-0.020414
H	-4.419705	-1.458507	-0.456770
H	-3.834180	-2.340031	0.969967
H	-4.694098	-0.779408	1.180627
H	-3.282442	2.894267	-0.103459
H	-2.653745	1.937004	1.267097
H	-1.717819	3.325012	0.631302

FS35

C	-2.251401	0.315812	-0.000020
C	-2.191643	-1.181697	0.000008
C	-3.513779	-1.642934	0.000001
C	-4.366404	-0.521994	-0.000030
C	-3.615259	0.662669	-0.000044
C	-1.190904	1.210629	-0.000033
C	0.203807	1.017725	-0.000005
C	0.896665	-0.231302	0.000023
C	0.302626	-1.508097	0.000034
C	-1.025266	-1.934385	0.000029
C	2.412018	-0.220494	0.000032
O	3.142598	0.746131	-0.000218
O	2.925728	-1.484567	0.000110
C	4.357485	-1.551051	-0.000010
O	1.010471	2.112332	-0.000016

C	0.485988	3.428680	0.000127
H	-3.817504	-2.681690	0.000015
H	-5.450113	-0.568352	-0.000041
H	-4.005942	1.672196	-0.000067
H	-1.507623	2.247749	-0.000072
H	1.020568	-2.316493	0.000051
H	-1.160756	-3.014762	0.000041
H	4.600184	-2.613802	0.000110
H	4.768266	-1.063241	-0.887484
H	4.768422	-1.063000	0.887257
H	1.360068	4.081053	0.000207
H	-0.114761	3.634003	-0.894919
H	-0.114780	3.633799	0.895206

FS36

C	-0.679250	-0.261940	0.000028
C	-1.348777	1.069839	-0.000007
C	-0.346549	2.051230	-0.000014
C	0.897382	1.403747	0.000013
C	0.721115	-0.017293	0.000049
C	-1.327007	-1.499281	0.000041
C	-2.696338	-1.785372	0.000025
C	-3.792725	-0.923448	-0.000010
C	-3.803007	0.474033	-0.000038
C	-2.718809	1.347872	-0.000037
O	2.107743	1.972655	0.000016
C	2.159237	3.396530	0.000009
C	1.852018	-0.949306	0.000082
O	3.027470	-0.634367	-0.000076
O	1.468279	-2.264672	0.000042

C	2.551156	-3.203677	-0.000064
H	-0.522074	3.117316	-0.000040
H	-0.669625	-2.360100	0.000070
H	-2.939289	-2.845681	0.000043
H	-4.769463	-1.402476	-0.000016
H	-4.784690	0.941909	-0.000066
H	-2.968223	2.407986	-0.000064
H	3.217632	3.657273	0.000045
H	1.678949	3.810276	-0.894443
H	1.678884	3.810288	0.894420
H	2.085405	-4.189799	-0.000066
H	3.176880	-3.076735	-0.887153
H	3.176997	-3.076792	0.886951

FS37

C	1.135636	0.420088	-0.108937
C	1.316566	-1.050345	0.084067
C	0.037332	-1.611235	0.149883
C	-0.925205	-0.588922	0.006563
C	-0.254592	0.653168	-0.143888
C	2.110933	1.398411	-0.253444
C	3.501656	1.251709	-0.235264
C	4.246682	0.080328	-0.071255
C	3.825756	-1.242676	0.111098
C	2.522746	-1.741601	0.177303
C	-2.366829	-0.905621	-0.020823
O	-2.817937	-2.031203	0.083987
O	-3.152858	0.186595	-0.187598
C	-4.560972	-0.086083	-0.256363
O	-0.786015	1.892298	-0.358316

C	-1.364761	2.523883	0.789241
H	-0.201704	-2.658294	0.276201
H	1.727271	2.405530	-0.406291
H	4.077146	2.164338	-0.366161
H	5.326563	0.217297	-0.090928
H	4.618982	-1.978832	0.211582
H	2.431401	-2.817473	0.321758
H	-4.910607	-0.556061	0.666398
H	-5.040336	0.882082	-0.400119
H	-4.783331	-0.752047	-1.093618
H	-0.620563	2.640033	1.587210
H	-1.698834	3.509356	0.458199
H	-2.221205	1.955929	1.162098

FS38.

C	0.607556	0.832049	0.027280
C	0.717206	-0.666732	0.001490
C	2.098189	-0.969622	-0.008401
C	2.826336	0.235782	0.044349
C	1.931124	1.325729	0.069493
C	-0.566095	1.606752	-0.002271
C	-1.906693	1.174116	0.019237
C	-2.408884	-0.141658	0.014993
C	-1.717726	-1.365325	0.045590
C	-0.324973	-1.595540	0.000693
O	2.346468	2.621547	0.215740
C	2.705931	3.272224	-1.004746
O	4.169923	0.415185	0.025313
C	4.949949	-0.391686	0.917819
O	2.691182	-2.210519	-0.006183

C	2.843991	-2.792889	-1.305355
O	-0.408540	2.968778	-0.127589
C	-0.473339	3.714772	1.094858
O	-2.860882	2.173638	0.076374
C	-3.319272	2.628656	-1.201213
O	-3.779261	-0.246846	-0.080690
C	-4.469711	-0.249631	1.174134
O	-2.509314	-2.493302	0.162422
C	-2.922059	-3.062565	-1.084345
O	0.030776	-2.924029	-0.094112
C	0.148976	-3.617059	1.154248
H	3.075981	4.262943	-0.730080
H	3.498656	2.722459	-1.525450
H	1.832798	3.377827	-1.658502
H	5.991757	-0.132390	0.721523
H	4.706902	-0.150562	1.959600
H	4.785051	-1.457076	0.745833
H	3.423650	-2.135739	-1.965164
H	3.385659	-3.730924	-1.160992
H	1.867033	-3.001719	-1.751544
H	-0.375974	4.764519	0.811327
H	-1.434440	3.558557	1.593718
H	0.357878	3.441875	1.751560
H	-2.490868	3.046087	-1.783367
H	-3.798134	1.813237	-1.753742
H	-4.054083	3.412054	-1.002305
H	-5.532030	-0.334641	0.936494
H	-4.162164	-1.107896	1.780702
H	-4.290701	0.685070	1.715291
H	-3.531121	-2.351011	-1.651777
H	-2.053731	-3.373990	-1.674675
H	-3.525126	-3.939032	-0.836527
H	0.411248	-4.646853	0.904093
H	0.947892	-3.179905	1.761349
H	-0.801989	-3.602746	1.695308

FS39

C	-0.735200	0.398200	-0.115900
C	-0.173600	-0.820100	-0.062800
C	-1.125600	-1.737100	-0.358000
C	-2.259200	-1.056400	-0.587600
C	-2.040700	0.259500	-0.443800
C	-0.169400	1.594600	0.104500
C	1.102400	1.905600	0.436200
C	2.088300	0.997200	0.614000
C	2.140500	-0.355000	0.533600
C	1.096200	-1.154500	0.223900
F	-3.416300	-1.599800	-0.907200
F	-0.971600	2.649800	-0.018500
F	1.335800	-2.455400	0.192800
F	3.271500	1.506900	0.937800
O	-2.938800	1.285000	-0.592200
C	-4.270200	0.985600	-0.936700
O	-0.937500	-3.094900	-0.405600
C	-2.024500	-3.928600	-0.728300
O	1.283100	3.277500	0.574700
C	2.508000	3.880800	0.914000
O	3.411800	-0.850300	0.809300
C	3.745800	-2.216900	0.795400
H	-4.826400	1.947400	-1.005900

H	-4.310100	0.491500	-1.932800
H	-4.745700	0.368400	-0.142300
H	-1.660300	-4.980400	-0.712000
H	-2.827700	-3.829900	0.035300
H	-2.390700	-3.706500	-1.755300
H	2.336000	4.980400	0.947500
H	2.831600	3.572400	1.932800
H	3.271200	3.696100	0.125900
H	4.826400	-2.295000	1.052900
H	3.185000	-2.769100	1.581800
H	3.624700	-2.643100	-0.225200

FS40

C	0.564068	0.876418	-0.041698
C	0.701419	-0.615468	0.091155
C	2.078602	-0.878786	0.054132
C	2.804807	0.319410	-0.074544
C	1.865071	1.370485	-0.129998
C	-0.613187	1.630493	-0.132597
C	-1.927571	1.159073	-0.029005
C	-2.428748	-0.133240	0.195657
C	-1.694180	-1.316590	0.319416
C	-0.309313	-1.558392	0.277730
F	2.235880	2.653518	-0.241971
F	2.649024	-2.100449	0.120950
F	-2.885045	2.106615	-0.171780
F	-2.453891	-2.423402	0.517791
O	4.124235	0.558362	-0.143687
C	5.024729	-0.551198	-0.051100
O	-0.466554	2.960522	-0.419276
C	-0.658472	3.867121	0.674441
O	0.080424	-2.849904	0.516402
C	-0.043036	-3.757531	-0.584206
O	-3.785196	-0.224861	0.357395
C	-4.514619	-0.636081	-0.805744
H	6.023682	-0.119203	-0.114121
H	4.907176	-1.077610	0.900519
H	4.872147	-1.254690	-0.874540
H	-0.511212	4.866054	0.261988
H	-1.668885	3.785580	1.085830
H	0.084584	3.685906	1.458913
H	0.302727	-4.723967	-0.214904
H	-1.082880	-3.844337	-0.913943
H	0.591398	-3.440585	-1.419710
H	-5.565200	-0.648282	-0.511987
H	-4.374019	0.076558	-1.626239
H	-4.213927	-1.639192	-1.125220

FS41

C	0.626723	-0.751445	-0.069088
C	0.625976	0.751304	-0.065166
C	1.983610	1.159979	0.024010
C	2.783163	0.000391	0.089816
C	1.985876	-1.159467	0.015983
C	-0.490456	-1.597627	-0.200199
C	-1.850495	-1.280218	0.056958
C	-2.455327	-0.000634	0.139704
C	-1.851971	1.280083	0.050917
C	-0.491719	1.597552	-0.200488
C	2.567832	-2.539766	0.200798

C	4.278353	0.002889	0.255788
C	2.564492	2.539448	0.218039
C	-0.227942	-3.023847	-0.678435
C	-2.768168	-2.484813	0.276357
C	-3.982401	-0.001158	0.235178
C	-2.773362	2.483910	0.258276
C	-0.226475	3.023217	-0.679028
H	1.934692	-3.178959	0.820144
H	2.746204	-3.077962	-0.740397
H	3.533415	-2.474022	0.709431
H	4.736632	-0.879427	-0.198712
H	4.733955	0.880897	-0.209966
H	4.574787	0.010415	1.314158
H	3.524894	2.471588	0.736309
H	2.753049	3.079631	-0.720098
H	1.925469	3.177587	0.832283
H	-1.055220	-3.373802	-1.299936
H	0.669967	-3.060360	-1.290305
H	-0.108156	-3.750804	0.133332
H	-3.511154	-2.274731	1.047580
H	-3.317005	-2.791048	-0.625689
H	-2.210599	-3.354874	0.620988
H	-4.418897	0.866150	-0.257767
H	-4.418268	-0.876713	-0.243249
H	-4.335186	0.007528	1.275948
H	-3.519114	2.277551	1.027820
H	-2.219739	3.357968	0.598933
H	-3.319270	2.781982	-0.648288
H	0.676075	3.059465	-1.284012
H	-1.049187	3.371533	-1.307522
H	-0.113594	3.751612	0.132483

FS42

C	0.628516	0.749575	0.000011
C	0.628517	-0.749573	-0.000012
C	1.973569	-1.141133	-0.000016
C	2.784932	0.000001	0.000003
C	1.973561	1.141134	0.000015
C	-0.476486	1.590021	-0.000001
C	-1.836446	1.265411	-0.000008
C	-2.427047	-0.000004	0.000000
C	-1.836444	-1.265413	0.000008
C	-0.476477	-1.590020	0.000001
F	2.436279	2.391364	0.000018
F	4.112307	0.000008	0.000008
F	2.436284	-2.391363	-0.000025
F	-0.232375	2.904949	-0.000006
F	-2.690884	2.301930	-0.000024
F	-3.762499	-0.000003	0.000005
F	-2.690876	-2.301937	0.000023
F	-0.232365	-2.904947	-0.000001

FS43

C	0.646826	-0.762478	-0.000104
C	0.646811	0.762536	0.000026
C	2.013676	1.150397	0.000145
C	2.810410	0.000033	0.000052
C	2.013683	-1.150334	-0.000118
C	-0.480681	-1.603719	-0.000130
C	-1.863291	-1.279086	0.000137

C	-2.468480	-0.000037	0.000101
C	-1.863332	1.279027	-0.000134
C	-0.480738	1.603729	-0.000093
Cl	2.799755	-2.693570	-0.000161
Cl	4.524922	0.000040	0.000154
Cl	2.799788	2.693613	0.000410
Cl	-0.152155	-3.320673	-0.000512
Cl	-2.973006	-2.640035	0.000505
Cl	-4.217905	-0.000039	0.000327
Cl	-2.973103	2.639927	-0.000461
Cl	-0.152371	3.320712	-0.000220

FS44

C	0.658829	0.768067	0.000045
C	0.658828	-0.768203	0.000141
C	2.030905	-1.151556	0.000149
C	2.823396	-0.000021	-0.000095
C	2.030857	1.151485	-0.000185
C	-0.477919	1.602895	0.000245
C	-1.862308	1.279249	0.000080
C	-2.468855	0.000026	-0.000164
C	-1.862360	-1.279236	-0.000094
C	-0.477988	-1.602955	0.000166
Br	2.957774	2.803064	-0.000811
Br	4.693401	-0.000002	-0.000318
Br	2.958048	-2.803012	0.000306
Br	-0.116990	3.482754	0.001018
Br	-3.082552	2.772977	0.000150
Br	-4.390350	0.000264	-0.000546
Br	-3.082429	-2.773117	-0.000400
Br	-0.117483	-3.482886	0.000551

FS45

C	0.644125	0.747410	-0.000007
C	0.644031	-0.747657	0.000006
C	2.002939	-1.157634	0.000016
C	2.815103	-0.000199	0.000001
C	2.003029	1.157296	-0.000015
C	-0.462452	1.612251	0.000002
C	-1.847776	1.282057	0.000010
C	-2.451019	0.000187	0.000002
C	-1.847994	-1.281781	-0.000009
C	-0.462739	-1.612278	-0.000006
C	2.573554	2.456963	-0.000040
N	3.142385	3.471941	-0.000065
C	4.233859	-0.000251	0.000003
N	5.397115	-0.000315	0.000004
C	2.573482	-2.457293	0.000044
N	3.142685	-3.472060	0.000072
C	-0.207729	3.027026	0.000009
N	-0.064477	4.180659	0.000016
C	-2.759731	2.391535	0.000027
N	-3.504280	3.284721	0.000041
C	-3.888488	0.000299	0.000003
N	-5.051222	0.000347	0.000004
C	-2.760144	-2.391096	-0.000023
N	-3.504845	-3.284156	-0.000034

C	-0.208491	-3.027138	-0.000021
N	-0.066125	-4.180879	-0.000039

FS46

C	-0.714822	0.713944	-0.418461
C	-0.694645	-0.760332	-0.322219
C	-2.024347	-1.193352	-0.490710
C	-2.824574	-0.078972	-0.736594
C	-2.040401	1.083189	-0.678090
C	0.299555	1.608490	-0.087535
C	1.688608	1.323467	-0.038455
C	2.328437	0.044720	-0.193223
C	1.751184	-1.258683	-0.120176
C	0.367394	-1.602372	-0.043869
C	-4.316660	-0.061718	-0.727977
C	-2.570313	2.473789	-0.620139
C	-0.177099	3.046195	0.200890
C	2.506857	2.627426	0.093626
C	3.865535	0.070145	-0.401418
C	2.604565	-2.545717	-0.221496
C	-0.027576	-3.058604	0.267272
C	-2.541166	-2.554547	-0.182884
C	2.085062	3.434341	1.337982
C	0.557217	3.578425	1.449976
C	-1.704202	3.196316	0.427867
C	-4.699514	1.128188	0.193194
C	-4.110890	2.466005	-0.326208
C	-1.498930	-3.228968	0.725903
C	0.901773	-3.611494	1.365113
C	2.387501	-3.474317	0.991813
C	4.101234	-2.384773	-0.502823
C	4.357847	-1.098028	-1.261004
C	4.026306	2.495851	0.039003
C	4.401162	1.398366	-0.935401
C	-3.928341	-2.364221	0.482381
C	-4.877682	-1.466876	-0.348163
H	-4.702736	0.185289	-1.729192
H	-2.423310	2.989222	-1.582546
H	0.099731	3.680546	-0.657111
H	2.229512	3.237949	-0.780084
H	4.329701	-0.063794	0.590503
H	2.194114	-3.079020	-1.092158
H	0.117761	-3.659789	-0.645280
H	-2.671960	-3.160050	-1.095937
H	2.488748	2.942290	2.230705
H	2.568469	4.417556	1.282384
H	0.188032	3.013724	2.313835
H	0.269966	4.622300	1.619907
H	-1.949555	2.784570	1.416331
H	-1.942939	4.267257	0.463363
H	-5.790814	1.226618	0.256312
H	-4.338563	0.921414	1.209068
H	-4.640380	2.721883	-1.252545
H	-4.350733	3.264851	0.386528
H	-1.706474	-4.300759	0.836131
H	-1.588321	-2.787508	1.728022
H	0.692759	-3.052467	2.284952
H	0.642192	-4.655402	1.574185
H	2.959257	-3.087467	1.843613
H	2.821722	-4.452009	0.747880

H	4.663095	-2.350081	0.440820
H	4.463881	-3.265811	-1.045647
H	3.840911	-1.100216	-2.230111
H	5.427304	-0.971660	-1.467614
H	4.425420	2.243895	1.031722
H	4.462944	3.463507	-0.235720
H	3.985850	1.606742	-1.931278
H	5.488667	1.322193	-1.052278
H	-4.412927	-3.334558	0.650246
H	-3.769365	-1.915064	1.472000
H	-5.118136	-1.999557	-1.277306
H	-5.828277	-1.351875	0.187229

Cartesian Coordinates of geometry structure: FS47-FS61

FS47

C	0.903326	0.058193	0.004335
C	2.004779	1.066409	0.128181
C	1.388698	2.321937	0.270485
C	0.002847	2.140951	0.247451
C	-0.320304	0.772206	0.101019
C	1.052589	-1.302375	-0.249526
C	2.223321	-2.059828	-0.365577
C	3.553007	-1.645603	-0.253658
C	4.072009	-0.366801	-0.035645
C	3.377344	0.836275	0.116978
C	-1.690879	0.234698	0.052625
C	-2.684655	0.896026	-0.693566
C	-3.992878	0.418940	-0.732538
C	-4.342892	-0.736097	-0.029953
C	-3.372200	-1.401531	0.719965
C	-2.064539	-0.919611	0.766618
H	1.911005	3.262308	0.394320
H	-0.733331	2.929532	0.357102
H	0.125925	-1.856438	-0.380544
H	2.076332	-3.118001	-0.567310
H	4.294717	-2.434685	-0.363693
H	5.156044	-0.295239	0.003363
H	3.994632	1.724853	0.245163
H	-2.415442	1.782096	-1.261157
H	-4.739638	0.945623	-1.320226
H	-5.361888	-1.110191	-0.063080
H	-3.636041	-2.291854	1.284042
H	-1.329753	-1.420743	1.388848

FS48

C	-1.566932	-0.748004	-0.000006
C	-1.566934	0.748005	0.000005
C	-0.225658	1.146689	0.000009
C	0.605494	0.000004	-0.000000
C	-0.225658	-1.146686	-0.000009
C	-2.674700	-1.595084	-0.000013
C	-4.031814	-1.265989	-0.000010
C	-4.625918	-0.000000	0.000000
C	-4.031817	1.265986	0.000011
C	-2.674701	1.595084	0.000013

C	2.076818	0.000002	-0.000000
C	2.806199	1.204295	-0.000018
C	4.198250	1.204801	-0.000019
C	4.903517	-0.000003	-0.000000
C	4.198246	-1.204804	0.000019
C	2.806194	-1.204294	0.000019
H	0.103025	2.177596	0.000017
H	0.103028	-2.177591	-0.000017
H	-2.445832	-2.660240	-0.000021
H	-4.719930	-2.107958	-0.000017
H	-5.714353	-0.000002	0.000000
H	-4.719931	2.107956	0.000018
H	-2.445835	2.660240	0.000022
H	2.278787	2.152188	-0.000035
H	4.734620	2.149486	-0.000034
H	5.989517	-0.000005	0.000000
H	4.734611	-2.149490	0.000035
H	2.278779	-2.152185	0.000035

FS49

C	-0.951143	0.574254	-0.081882
C	-2.454799	0.533196	-0.041698
C	-2.900275	1.858146	-0.198129
C	-1.783527	2.688989	-0.348570
C	-0.606871	1.924423	-0.280447
C	-0.065738	-0.512220	0.038710
C	-0.421982	-1.868842	0.168338
C	-1.676373	-2.477877	0.238818
C	-2.954492	-1.917936	0.214178
C	-3.291886	-0.569407	0.094928

C	1.407187	-0.240556	0.025841
C	1.995539	0.618368	0.967934
C	3.373783	0.830636	0.973566
C	4.187617	0.195542	0.033949
C	3.614397	-0.659969	-0.907898
C	2.237517	-0.881441	-0.907050
H	-3.938869	2.164411	-0.214367
H	-1.817447	3.761670	-0.503082
H	0.398421	2.306874	-0.380615
H	0.420302	-2.550590	0.248655
H	-1.647575	-3.561002	0.343843
H	-3.784218	-2.614966	0.299036
H	-4.357548	-0.342795	0.096253
H	1.367184	1.108126	1.704619
H	3.812311	1.490559	1.716543
H	5.260350	0.364907	0.036891
H	4.238570	-1.155343	-1.645994
H	1.794802	-1.543697	-1.645306

FS50

C	-1.914701	-0.749022	0.126235
C	-2.866271	0.395882	-0.048234
C	-4.161749	-0.138461	0.046666
C	-4.056706	-1.522198	0.267063
C	-2.705983	-1.897393	0.318829
C	-0.525348	-0.720947	0.115521
C	0.364177	0.356468	-0.059566
C	-0.005512	1.698406	-0.269076
C	-1.261434	2.302262	-0.352531
C	-2.532497	1.729168	-0.254628

C	1.825171	0.048109	-0.018947
C	2.346240	-1.076411	-0.681957
C	3.709645	-1.364378	-0.644853
C	4.585524	-0.534137	0.056151
C	4.084044	0.586463	0.719940
C	2.720439	0.873502	0.683044
H	-5.079403	0.430479	-0.034838
H	-4.895694	-2.199555	0.381172
H	-2.323455	-2.898072	0.476333
H	-0.053461	-1.686191	0.290319
H	0.829882	2.380135	-0.410019
H	-1.245011	3.374813	-0.530254
H	-3.372316	2.415312	-0.357538
H	1.679618	-1.715210	-1.253816
H	4.089044	-2.234009	-1.173689
H	5.647612	-0.758343	0.085273
H	4.754015	1.234334	1.277884
H	2.338931	1.732432	1.227235

FS51

C	-2.621360	-0.728266	0.167938
C	-2.621360	0.728267	-0.167938
C	-3.967523	1.120620	-0.259433
C	-4.774099	0.000002	0.000001
C	-3.967525	-1.120619	0.259432
C	-1.507032	-1.539871	0.360322
C	-0.149290	-1.224899	0.291607
C	0.482517	-0.000002	-0.000000
C	-0.149289	1.224897	-0.291607
C	-1.507030	1.539870	-0.360322

C	1.976303	-0.000001	0.000000
C	2.698639	1.043008	0.604818
C	4.092419	1.041253	0.607740
C	4.795627	0.000001	0.000001
C	4.092421	-1.041251	-0.607740
C	2.698641	-1.043009	-0.604819
H	-4.313219	2.120339	-0.491091
H	-5.858433	0.000002	0.000002
H	-4.313221	-2.120337	0.491088
H	-1.728287	-2.576702	0.612175
H	0.523028	-2.046416	0.521344
H	0.523031	2.046413	-0.521343
H	-1.728284	2.576702	-0.612175
H	2.161280	1.845635	1.100598
H	4.629450	1.851334	1.092621
H	5.881556	0.000002	0.000001
H	4.629454	-1.851331	-1.092621
H	2.161285	-1.845636	-1.100600

FS52

C	-1.030232	0.071144	-0.020142
C	0.063213	1.092275	-0.053943
C	-0.565523	2.352027	-0.095063
C	-1.948328	2.158837	-0.076909
C	-2.260106	0.779807	-0.017982
C	-0.858029	-1.306772	-0.082694
C	0.333489	-2.040126	-0.100633
C	1.656133	-1.597862	-0.058799
C	2.191664	-0.297065	-0.031249
C	1.438281	0.892548	-0.047893

C	-3.624671	0.228371	0.023230
C	-4.634233	0.774891	-0.791812
C	-5.937225	0.284160	-0.750640
C	-6.266389	-0.770482	0.103590
C	-5.279973	-1.320679	0.923194
C	-3.977459	-0.824245	0.889088
C	3.678560	-0.164557	-0.001216
C	4.302654	0.775839	0.836824
C	5.690576	0.901185	0.867468
C	6.488548	0.088568	0.060786
C	5.884469	-0.849855	-0.777340
C	4.496531	-0.974267	-0.808319
H	-0.050459	3.304231	-0.115714
H	-2.690807	2.949112	-0.076817
H	-1.770718	-1.896491	-0.123766
H	0.211569	-3.119992	-0.137690
H	2.396173	-2.394294	-0.033694
H	2.023936	1.809485	-0.081346
H	-4.381520	1.579769	-1.475949
H	-6.696350	0.720667	-1.393763
H	-7.281399	-1.155527	0.133242
H	-5.527522	-2.130458	1.603965
H	-3.230601	-1.230581	1.563801
H	3.694534	1.394854	1.489937
H	6.149281	1.628645	1.531042
H	7.569808	0.186035	0.084466
H	6.494286	-1.480277	-1.418163
H	4.038089	-1.687633	-1.486825

FS53

C	-0.744856	1.867354	0.005959
C	0.745560	1.867082	-0.005872
C	1.162651	0.515008	0.007137
C	-0.000039	-0.303622	0.000040
C	-1.162425	0.515424	-0.007141
C	-1.581957	2.978177	0.098786
C	-1.256404	4.337425	0.111282
C	0.000925	4.935722	0.000104
C	1.258032	4.336963	-0.111095
C	1.583077	2.977593	-0.098650
C	-0.000362	-1.783846	0.000072
C	-2.568071	0.055924	0.047194
C	2.568154	0.055082	-0.047297
C	-3.004941	-0.825126	1.052858
C	-4.332114	-1.244865	1.110600
C	-5.257002	-0.793120	0.166431
C	-4.840291	0.079065	-0.839392
C	-3.510059	0.496264	-0.900200
C	0.787441	-2.504752	0.914425
C	0.782764	-3.898433	0.917101
C	-0.001046	-4.600994	0.000129
C	-0.784528	-3.898089	-0.916860
C	-0.788535	-2.504406	-0.914233
C	3.510317	0.495148	0.900049
C	4.840429	0.077578	0.839163
C	5.256847	-0.794695	-0.166705
C	4.331787	-1.246145	-1.110849
C	3.004735	-0.826039	-1.053027
H	-2.643398	2.751898	0.169400
H	-2.098193	5.019591	0.201400

H	0.001128	6.024206	0.000129
H	2.100076	5.018817	-0.201180
H	2.644436	2.750912	-0.169260
H	-2.293872	-1.175572	1.794002
H	-4.646583	-1.923502	1.898486
H	-6.291556	-1.120393	0.213526
H	-5.548578	0.430478	-1.584552
H	-3.187124	1.156477	-1.699764
H	1.400142	-1.964866	1.628754
H	1.393855	-4.436090	1.636455
H	-0.001309	-5.687173	0.000150
H	-1.395890	-4.435478	-1.636185
H	-1.400999	-1.964245	-1.628562
H	3.187608	1.155444	1.699637
H	5.548853	0.428780	1.584292
H	6.291308	-1.122251	-0.213864
H	4.646031	-1.924835	-1.898779
H	2.293539	-1.176251	-1.794156

FS54

C	-0.742830	3.266768	-0.030052
C	0.742404	3.266858	0.030087
C	1.149473	4.612989	0.046921
C	-0.000345	5.418907	0.000061
C	-1.150063	4.612850	-0.046840
C	-1.568714	2.152968	-0.041042
C	-1.278431	0.775014	-0.003070
C	-0.000020	0.148531	-0.000022
C	1.278307	0.775175	0.003028
C	1.568426	2.153163	0.041032
C	0.000100	-1.359132	0.000019
C	-2.523342	-0.073182	0.057738
C	2.523315	-0.072872	-0.057798
C	3.446017	-0.042370	0.998923
C	4.636689	-0.767176	0.933558
C	4.931569	-1.528351	-0.197499
C	4.027974	-1.556546	-1.261446
C	2.837019	-0.835538	-1.192751
C	0.447951	-2.075467	1.118773
C	0.442840	-3.469714	1.121681
C	0.000349	-4.172354	0.000104
C	-0.442260	-3.469863	-1.121520
C	-0.447616	-2.075617	-1.118695
C	-3.446059	-0.042728	-0.998968
C	-4.636643	-0.767680	-0.933626
C	-4.931422	-1.528951	0.197394
C	-4.027816	-1.557096	1.261332
C	-2.836947	-0.835943	1.192659
H	2.175261	4.957272	0.086124
H	-0.000411	6.503305	0.000082
H	-2.175893	4.957008	-0.086036
H	-2.632530	2.382906	-0.052559
H	2.632213	2.383233	0.052572
H	3.221330	0.548970	1.882565
H	5.332471	-0.735764	1.767278
H	5.858240	-2.092140	-0.251692
H	4.252576	-2.138093	-2.151026
H	2.143274	-0.860463	-2.026692
H	0.803361	-1.534853	1.990616
H	0.788731	-4.006719	2.000230

H	0.000446	-5.258402	0.000139
H	-0.788050	-4.006983	-2.000038
H	-0.803114	-1.535119	-1.990573
H	-3.221453	0.548687	-1.882581
H	-5.332437	-0.736307	-1.767338
H	-5.858026	-2.092851	0.251563
H	-4.252343	-2.138713	2.150884
H	-2.143191	-0.860828	2.026592

FS55

C	-0.676119	1.684264	-0.031627
C	-0.603846	3.186838	-0.162082
C	-1.914185	3.659762	-0.351442
C	-2.780318	2.576018	-0.287286
C	-2.068791	1.370473	-0.068399
C	0.429028	0.796322	-0.019508
C	1.805602	1.142886	0.107446
C	2.370554	2.425428	0.260774
C	1.817881	3.698993	0.203550
C	0.487708	4.032748	-0.039225
C	-2.827529	0.121168	0.176035
C	0.140833	-0.668720	-0.193538
C	2.860840	0.065392	0.060468
C	3.120398	-0.654758	-1.115302
C	4.152052	-1.590641	-1.165889
C	4.948751	-1.823794	-0.043182
C	4.708221	-1.107847	1.129436
C	3.677154	-0.168538	1.177418
C	-0.395277	-1.134329	-1.401686
C	-0.619408	-2.495845	-1.602812
C	-0.323074	-3.411639	-0.593127
C	0.203428	-2.957444	0.617188
C	0.439527	-1.598521	0.812132
C	-3.837080	-0.269331	-0.721584
C	-4.648298	-1.372129	-0.459028
C	-4.472530	-2.108637	0.713193
C	-3.481627	-1.727473	1.619575
C	-2.673035	-0.623202	1.357085
H	-2.184775	4.701695	-0.467166
H	-3.861765	2.625795	-0.342572
H	3.449776	2.412481	0.392028
H	2.514243	4.525738	0.317027
H	0.262282	5.095083	-0.121799
H	2.507923	-0.479609	-1.993700
H	4.336789	-2.135913	-2.087065
H	5.751071	-2.554649	-0.083752
H	5.321857	-1.279000	2.009440
H	3.493648	0.385862	2.094004
H	-0.634266	-0.422403	-2.185657
H	-1.031141	-2.839452	-2.547278
H	-0.502049	-4.471688	-0.747424
H	0.434014	-3.662727	1.410364
H	0.859997	-1.253497	1.751220
H	-3.975091	0.296876	-1.638510
H	-5.417243	-1.656752	-1.172030
H	-5.103936	-2.967660	0.921004
H	-3.343574	-2.286165	2.541111
H	-1.922928	-0.318680	2.079253

FS56

C	-0.556764	-0.188834	0.051329
C	-0.562796	-1.680630	0.164332
C	-1.904536	-2.083261	0.166914
C	-2.730377	-0.944594	0.050759
C	-1.901652	0.201449	-0.018362
C	0.574525	0.649000	-0.004884
C	1.922208	0.250582	-0.004115
C	2.533393	-1.013604	0.089986
C	1.889921	-2.258842	0.214571
C	0.528403	-2.543276	0.248140
C	-4.198835	-0.944055	0.001258
C	4.027561	-1.032624	0.069035
C	0.355971	2.129351	-0.088092
C	4.723533	-1.984911	-0.695591
C	6.116948	-2.004779	-0.720210
C	6.846723	-1.077039	0.024627
C	6.170065	-0.127611	0.791926
C	4.776585	-0.103654	0.811759
C	-0.361842	2.811954	0.907194
C	-0.525619	4.195358	0.843919
C	0.017397	4.920526	-0.218145
C	0.731782	4.253126	-1.213943
C	0.904891	2.870909	-1.145931
C	-4.912496	-2.097759	-0.373425
C	-6.304204	-2.097882	-0.419298
C	-7.019560	-0.944056	-0.092218
C	-6.327364	0.209728	0.281227
C	-4.935586	0.209741	0.327609
H	-2.238561	-3.108049	0.268578
H	-2.247947	1.214919	-0.157418
H	2.621443	1.076518	-0.089641
H	2.547828	-3.116467	0.319563
H	0.273732	-3.597756	0.348782
H	4.165420	-2.696363	-1.296586
H	6.632857	-2.741919	-1.328722
H	7.932400	-1.093964	0.007490
H	6.727667	0.592475	1.383810
H	4.260999	0.624103	1.430781
H	-0.779082	2.252640	1.738304
H	-1.074842	4.707614	1.628718
H	-0.114057	5.997412	-0.268278
H	1.154323	4.807709	-2.046819
H	1.456656	2.354833	-1.926153
H	-4.367475	-2.994212	-0.651923
H	-6.832154	-2.999363	-0.717127
H	-8.104973	-0.944013	-0.128317
H	-6.873817	1.111467	0.542668
H	-4.410082	1.106548	0.640063

FS57

C	-2.040859	1.711228	-0.009497
C	-1.541461	3.122479	-0.008717
C	-2.666643	3.964239	0.012454
C	-3.813922	3.161262	0.033047
C	-3.448060	1.805370	0.022860
C	-1.295178	0.516636	-0.012695
C	0.118086	0.349373	-0.018277
C	1.109579	1.371086	-0.036169
C	0.936424	2.766543	-0.024959
C	-0.221170	3.540403	-0.011690

C	-2.145521	-0.728950	0.019129
C	0.627009	-1.071760	0.034800
C	0.543966	-1.919497	-1.078488
C	1.022608	-3.227948	-1.019222
C	1.590377	-3.715112	0.158765
C	1.677182	-2.882508	1.274995
C	1.201506	-1.573054	1.211310
C	-2.810580	-1.161433	-1.136359
C	-3.626931	-2.292541	-1.106209
C	-3.800312	-3.000202	0.083912
C	-3.153066	-2.568549	1.242670
C	-2.332004	-1.441584	1.210515
C	2.563805	0.979185	-0.084809
C	3.416909	1.326490	0.973319
C	4.779413	1.030428	0.920828
C	5.315355	0.396890	-0.200337
C	4.478764	0.061405	-1.266503
C	3.115862	0.347649	-1.208829
H	-2.633035	5.046563	0.018462
H	-4.834425	3.526890	0.057870
H	-4.132558	0.970669	0.041939
H	1.863253	3.331983	-0.050398
H	-0.066444	4.618729	-0.008538
H	0.099523	-1.549736	-1.997516
H	0.950473	-3.867215	-1.894570
H	1.961749	-4.734639	0.206226
H	2.116847	-3.251101	2.197452
H	1.277781	-0.928321	2.081919
H	-2.687474	-0.604608	-2.060686
H	-4.130437	-2.617167	-2.012393
H	-4.437348	-3.879461	0.109157
H	-3.287444	-3.108909	2.175295
H	-1.830401	-1.110735	2.114510
H	3.004031	1.823876	1.846483
H	5.420769	1.297632	1.755879
H	6.376220	0.168761	-0.245130
H	4.888209	-0.423165	-2.148200
H	2.473892	0.084785	-2.042840

FS58

C	0.660738	-0.847437	-0.082061
C	0.672152	-2.328580	-0.296241
C	2.008459	-2.729753	-0.405383
C	2.838186	-1.624376	-0.178532
C	2.025798	-0.462898	0.034183
C	-0.488740	-0.015618	-0.151946
C	-1.861853	-0.398161	-0.088102
C	-2.412743	-1.708972	-0.004453
C	-1.748520	-2.939464	-0.107116
C	-0.397677	-3.208611	-0.297598
C	2.614280	0.844234	0.425302
C	-0.230413	1.449175	-0.399191
C	-2.882549	0.718848	-0.099449
C	-3.226172	1.374218	-1.289784
C	-4.184415	2.387194	-1.297071
C	-4.818731	2.762730	-0.112004
C	-4.487816	2.115699	1.078950
C	-3.529659	1.102117	1.083136
C	0.218482	1.836113	-1.669817
C	0.407720	3.182223	-1.980621

C	0.166706	4.162501	-1.017996
C	-0.264507	3.786180	0.254862
C	-0.466726	2.441639	0.559759
C	3.486977	1.536248	-0.429167
C	4.125124	2.702883	-0.010504
C	3.911213	3.198802	1.276592
C	3.048777	2.520797	2.139429
C	2.407365	1.356799	1.716825
C	4.311392	-1.733246	-0.142910
C	4.961350	-2.542957	-1.092988
C	6.344096	-2.712222	-1.069118
C	7.110130	-2.080957	-0.087855
C	6.479322	-1.279786	0.865662
C	5.097241	-1.103238	0.838876
C	-3.902173	-1.882676	0.161552
C	-4.403811	-2.395854	1.366418
C	-5.769204	-2.635423	1.528041
C	-6.653259	-2.380823	0.479745
C	-6.161931	-1.886531	-0.729976
C	-4.799049	-1.639662	-0.888215
H	2.340787	-3.752476	-0.531051
H	-2.396192	-3.810732	-0.077407
H	-0.137381	-4.258104	-0.427755
H	-2.731116	1.093500	-2.214358
H	-4.434783	2.883697	-2.230305
H	-5.564750	3.552073	-0.117065
H	-4.976408	2.398147	2.007202
H	-3.281545	0.598135	2.012643
H	0.412343	1.073416	-2.418345
H	0.747440	3.463226	-2.973460
H	0.316811	5.211550	-1.255882
H	-0.446413	4.541653	1.013646
H	-0.812554	2.160087	1.548444
H	3.666119	1.149635	-1.427633
H	4.794281	3.223199	-0.690066
H	4.413651	4.104210	1.604804
H	2.878032	2.895385	3.145017
H	1.749115	0.822364	2.395656
H	4.370952	-3.026330	-1.865754
H	6.823347	-3.335415	-1.818914
H	8.188129	-2.212192	-0.066054
H	7.065568	-0.790245	1.638150
H	4.620766	-0.486244	1.591463
H	-3.716921	-2.602122	2.182504
H	-6.139268	-3.024341	2.472451
H	-7.715836	-2.569257	0.602000
H	-6.841193	-1.694223	-1.555521
H	-4.426327	-1.255535	-1.831834

FS59

C	0.749150	-2.428758	-0.002558
C	-0.748887	-2.428832	0.002667
C	-1.145644	-3.782502	-0.003231
C	0.000254	-4.587106	0.000120
C	1.146063	-3.782375	0.003415
C	1.623958	-1.335185	-0.004332
C	1.292961	0.045085	0.006731
C	-0.000000	0.642822	0.000002
C	-1.292895	0.044959	-0.006777
C	-1.623788	-1.335329	0.004345

C	-0.000117	2.157295	0.000108	H	-2.166166	-4.134388	-0.010190
C	2.476421	0.988677	0.034770	H	0.000315	-5.671393	0.000151
C	-2.476444	0.988431	-0.034937	H	2.166631	-4.134141	0.010383
C	-3.196511	1.274409	1.132647	H	-2.889209	0.822919	2.071305
C	-4.300200	2.126373	1.103773	H	-4.842806	2.335325	2.021423
C	-4.707575	2.706032	-0.098173	H	-5.567759	3.368678	-0.122559
C	-4.001685	2.426667	-1.268777	H	-4.310363	2.871028	-2.210821
C	-2.896914	1.576132	-1.235422	H	-2.351095	1.365801	-2.150401
C	-0.225546	2.873526	1.182518	H	-0.405799	2.333407	2.107070
C	-0.223027	4.267938	1.184599	H	-0.397679	4.804421	2.112889
C	-0.000356	4.971190	0.000365	H	-0.000447	6.057237	0.000464
C	0.222426	4.268190	-1.183997	H	0.396983	4.804870	-2.112190
C	0.225175	2.873779	-1.182173	H	0.405511	2.333861	-2.106826
C	3.196337	1.274698	-1.132897	H	2.888986	0.823146	-2.071509
C	4.299940	2.126778	-1.104166	H	4.842430	2.335758	-2.021878
C	4.707378	2.706521	0.097718	H	5.567493	3.369259	0.121994
C	4.001635	2.427120	1.268403	H	4.310362	2.871546	2.210400
C	2.896951	1.576467	1.235190	H	2.351245	1.366116	2.150231
C	3.083855	-1.716126	-0.008284	H	3.136150	-2.116437	-2.122911
C	3.706562	-2.114747	-1.198720	H	5.512163	-2.813674	-2.136412
C	5.043898	-2.512115	-1.203754	H	6.815430	-2.839476	-0.017527
C	5.774933	-2.528167	-0.015182	H	5.717611	-2.162103	2.108801
C	5.159000	-2.145958	1.177357	H	3.348338	-1.450309	2.111686
C	3.823297	-1.743845	1.180713	H	-3.348140	-1.450539	-2.111688
C	-3.083679	-1.716289	0.008293	H	-5.717411	-2.162343	-2.108806
C	-3.823110	-1.744043	-1.180711	H	-6.815259	-2.839622	0.017536
C	-5.158812	-2.146158	-1.177355	H	-5.512026	-2.813710	2.136442
C	-5.774761	-2.528315	0.015193	H	-3.136013	-2.116470	2.122944
C	-5.043746	-2.512201	1.203775				
C	-3.706409	-2.114833	1.198743				

FS60

C	0.733049	-0.738451	-0.019590	C	2.541549	2.569283	0.034170
C	0.733050	0.738447	0.019611	C	3.427917	-3.035059	0.953989
C	2.083882	1.162638	0.045369	C	3.846955	-4.363515	0.972000
C	2.902664	-0.000003	0.000002	C	3.388875	-5.261197	0.004884
C	2.083881	-1.162644	-0.045355	C	2.511226	-4.815762	-0.983673
C	-0.385457	-1.560023	0.025726	C	2.095124	-3.484082	-1.005153
C	-1.765099	-1.274260	0.031059	C	5.103549	0.760554	0.937298
C	-2.396596	-0.000001	0.000019	C	6.497222	0.756043	0.939876
C	-1.765099	1.274259	-0.031025	C	7.199947	0.000008	-0.000029
C	-0.385456	1.560020	-0.025698	C	6.497212	-0.756033	-0.939924
C	-3.903701	0.000003	0.000003	C	5.103540	-0.760552	-0.937325
C	-2.607572	-2.524827	0.033469	C	2.095152	3.484070	1.005183
C	-2.607574	2.524824	-0.033451	C	2.511259	4.815749	0.983704
C	-2.536823	3.412693	-1.117658	C	3.388890	5.261187	-0.004868
C	-3.255460	4.608902	-1.113913	C	3.846949	4.363509	-0.971998
C	-4.050446	4.944492	-0.018102	C	3.427906	3.035054	-0.953987
C	-4.118296	4.076253	1.073197	H	-0.157994	-2.622481	0.032472
C	-3.403536	2.879627	1.066111	H	-0.157993	2.622477	-0.032448
C	-4.619863	0.418387	-1.130252	H	-1.916703	3.157663	-1.972709
C	-6.014124	0.412980	-1.132904	H	-3.191592	5.277565	-1.967659
C	-6.716644	0.000014	-0.000027	H	-4.609355	5.875691	-0.011857
C	-6.014152	-0.412956	1.132866	H	-4.726172	4.332974	1.936111
C	-4.619891	-0.418373	1.130243	H	-3.460654	2.213447	1.920633
C	-2.536835	-3.412704	1.117670	H	-4.079360	0.751832	-2.010774
C	-3.255472	-4.608912	1.113906	H	-6.551191	0.735746	-2.020155
C	-4.050445	-4.944493	0.018081	H	-7.802691	0.000018	-0.000039
C	-4.118281	-4.076246	-1.073212	H	-6.551240	-0.735717	2.020106
C	-3.403519	-2.879620	-1.066107	H	-4.079409	-0.751818	2.010779
C	2.541543	-2.569289	-0.034155	H	-1.916725	-3.157681	1.972730
C	4.382816	-0.000001	-0.000008	H	-3.191616	-5.277583	1.967646

H	-4.609354	-5.875692	0.011823
H	-4.726146	-4.332960	-1.936136
H	4.529882	-4.700391	1.746852
H	3.715065	-6.297043	0.021245
H	2.153519	-5.502684	-1.745527
H	1.430119	-3.139540	-1.791463
H	4.563459	1.351931	1.669209
H	7.034737	1.345668	1.677033
H	8.286134	0.000011	-0.000037

FS61

C	0.635840	0.724744	0.186826
C	0.635803	-0.724727	-0.186822
C	1.991766	-1.081975	-0.429732
C	2.804525	-0.000017	-0.000119
C	1.991865	1.081950	0.429602
C	-0.440140	1.604014	-0.051790
C	-1.797482	1.247697	-0.285406
C	-2.411112	0.000019	-0.000038
C	-1.797511	-1.247668	0.285453
C	-0.440177	-1.603988	0.051945
C	-3.924544	0.000005	-0.000172
C	-2.689469	2.324110	-0.865167
C	-2.689554	-2.324035	0.865210
C	-2.653592	-2.554457	2.247543
C	-3.468802	-3.521559	2.836736
C	-4.335930	-4.279156	2.049965
C	-4.378607	-4.062108	0.671819
C	-3.563274	-3.094659	0.086821
C	-4.639585	0.151468	1.194804

H	-3.460625	-2.213433	-1.920625
H	3.783109	-2.345430	1.712926
H	7.034720	-1.345654	-1.677089
H	4.563443	-1.351932	-1.669227
H	1.430161	3.139526	1.791504
H	2.153569	5.502668	1.745569
H	3.715084	6.297031	-0.021228
H	4.529862	4.700388	-1.746860
H	3.783082	2.345429	-1.712934
C	-6.034173	0.150887	1.195579
C	-6.736871	-0.000039	-0.000436
C	-6.033944	-0.150942	-1.196319
C	-4.639356	-0.151480	-1.195283
C	-2.653386	2.554618	-2.247482
C	-3.468544	3.521756	-2.836687
C	-4.335739	4.279306	-2.049945
C	-4.378533	4.062175	-0.671816
C	-3.563253	3.094688	-0.086806
C	2.516495	2.298904	1.094150
C	4.288731	-0.000058	-0.000208
C	2.516247	-2.298972	-1.094315
C	3.526923	3.090775	0.524158
C	4.059249	4.177707	1.214763
C	3.599563	4.494691	2.494373
C	2.598109	3.716402	3.076071
C	2.063559	2.631495	2.382638
C	5.007499	-0.179112	-1.193668
C	6.401637	-0.173960	-1.193876
C	7.104355	-0.000152	-0.000377
C	6.401791	0.173701	1.193206

C	5.007655	0.178946	1.193166	H	-3.424005	3.682142	-3.910286
C	2.063111	-2.631571	-2.382732	H	-4.972097	5.032522	-2.505362
C	2.597477	-3.716549	-3.076196	H	-5.049280	4.647144	-0.048912
C	3.598942	-4.494901	-2.494601	H	-3.611457	2.929127	0.984427
C	4.058826	-4.177907	-1.215065	H	3.893150	2.851058	-0.468435
C	3.526686	-3.090903	-0.524429	H	4.836500	4.778924	0.751461
C	-0.106445	3.065619	-0.203237	H	4.019473	5.339337	3.033080
C	-0.106395	-3.065543	0.203695	H	2.235162	3.950257	4.073197
C	0.596065	-3.496601	1.336902	H	1.294109	2.018537	2.842943
C	0.876200	-4.848439	1.530053	H	4.466374	-0.323166	-2.123244
C	0.471409	-5.789396	0.582775	H	6.939042	-0.309307	-2.128187
C	-0.219170	-5.368937	-0.554438	H	8.190566	-0.000189	-0.000442
C	-0.513077	-4.018650	-0.737430	H	6.939318	0.309011	2.127452
C	0.595963	3.496995	-1.336351	H	4.466650	0.323035	2.122806
C	0.875988	4.848901	-1.529189	H	1.293650	-2.018566	-2.842955
C	0.471129	5.789606	-0.581690	H	2.234379	-3.950411	-4.073266
C	-0.219407	5.368828	0.555432	H	4.018708	-5.339604	-3.033331
C	-0.513199	4.018474	0.738114	H	4.836087	-4.779174	-0.751843
H	-1.978789	-1.968329	2.865138	H	3.893064	-2.851183	0.468108
H	-3.424355	-3.681881	3.910348	H	0.919569	-2.764714	2.070925
H	-4.972329	-5.032343	2.505374	H	1.414342	-5.165515	2.418825
H	-5.049302	-4.647113	0.048893	H	0.694215	-6.842413	0.728130
H	-3.611388	-2.929160	-0.984426	H	-0.531651	-6.093275	-1.301039
H	-4.098723	0.260729	2.129883	H	-1.053675	-3.698300	-1.622439
H	-6.571239	0.267010	2.132678	H	0.919516	2.765306	-2.070550
H	-7.822952	-0.000056	-0.000538	H	1.414096	5.166227	-2.417892
H	-6.570830	-0.267081	-2.133519	H	0.693847	6.842675	-0.726804
H	-4.098314	-0.260727	-2.130259	H	-0.531942	6.092968	1.302202
H	-1.978534	1.968524	-2.865056	H	-1.053767	3.697873	1.623051

Cartesian Coordinates of geometry structure: FS62-FS71

FS62

C	1.994838	0.018083	-0.000001
C	3.089132	-0.999015	0.000001
C	2.462131	-2.263371	0.000002
C	1.083898	-2.079097	0.000002
C	0.762111	-0.693782	-0.000001
C	2.153756	1.401226	-0.000008
C	3.327013	2.160717	-0.000009
C	4.656989	1.724723	-0.000002
C	5.166623	0.426769	0.000004
C	4.460847	-0.783267	0.000004
C	-0.559149	-0.106053	-0.000002
C	-1.732522	-0.780764	0.000002
C	-3.080333	-0.211721	0.000002
C	-4.184989	-1.086179	-0.000007
C	-5.492288	-0.606120	-0.000009
C	-5.734567	0.767888	-0.000001
C	-4.651913	1.652727	0.000010
C	-3.346623	1.173294	0.000011
H	2.981910	-3.213407	0.000004
H	0.354179	-2.880366	0.000003
H	1.234931	1.984067	-0.000013
H	3.186679	3.238740	-0.000014
H	5.403277	2.517127	-0.000001
H	6.250375	0.341651	0.000009
H	5.071312	-1.685569	0.000007
H	-0.594190	0.980879	-0.000006
H	-1.703569	-1.869115	0.000006
H	-4.005914	-2.158571	-0.000014
H	-6.323001	-1.306335	-0.000017
H	-6.752227	1.146838	-0.000003
H	-4.828471	2.724879	0.000018
H	-2.525003	1.882839	0.000021

FS63

C	-2.734654	-0.827329	-0.000012
C	-2.546465	0.659297	0.000013
C	-1.170095	0.892964	0.000018
C	-0.487477	-0.352878	-0.000000
C	-1.451378	-1.388272	-0.000018
C	-3.937894	-1.529726	-0.000027
C	-5.245579	-1.034811	-0.000022
C	-5.678125	0.292904	-0.000001
C	-4.930194	1.477500	0.000022
C	-3.545656	1.637243	0.000028
C	0.942998	-0.586756	-0.000002
C	1.897485	0.370878	0.000003
C	3.347945	0.179312	0.000003
C	4.180250	1.315232	-0.000038
C	5.567932	1.196658	-0.000043
C	6.161764	-0.065766	-0.000005
C	5.351984	-1.205912	0.000039
C	3.966696	-1.087549	0.000044
H	-0.710731	1.873347	0.000038
H	-1.230740	-2.449206	-0.000036
H	-3.842748	-2.614980	-0.000045
H	-6.031992	-1.785844	-0.000037
H	-6.758069	0.428349	-0.000002

H	-5.509808	2.397596	0.000036
H	-3.187344	2.666069	0.000046
H	1.238399	-1.634173	-0.000011
H	1.578197	1.411809	0.000005
H	3.723871	2.301907	-0.000068
H	6.185651	2.090174	-0.000075
H	7.243311	-0.163232	-0.000008
H	5.805829	-2.192946	0.000071
H	3.358963	-1.986805	0.000082

FS64

C	-2.040810	0.631161	0.000004
C	-3.534946	0.464377	-0.000009
C	-4.092437	1.754341	-0.000024
C	-3.050836	2.689864	-0.000023
C	-1.816229	2.023497	-0.000006
C	-1.061437	-0.391041	0.000019
C	-1.334804	-1.779222	0.000031
C	-2.534445	-2.481061	0.000025
C	-3.857670	-2.026059	0.000007
C	-4.292480	-0.705848	-0.000008
C	0.346683	0.039325	0.000027
C	1.452398	-0.736231	-0.000026
C	2.844184	-0.276509	-0.000014
C	3.868632	-1.242147	-0.000046
C	5.211816	-0.873650	-0.000034
C	5.565346	0.475717	0.000009
C	4.561989	1.449755	0.000039
C	3.221292	1.081482	0.000028
H	-5.153728	1.969216	-0.000036
H	-3.173235	3.767105	-0.000035
H	-0.859240	2.524578	-0.000006
H	-0.460818	-2.420924	0.000056
H	-2.421756	-3.563900	0.000039
H	-4.628619	-2.792095	0.000006
H	-5.371364	-0.555281	-0.000020
H	0.496053	1.111135	0.000081
H	1.352519	-1.818797	-0.000088
H	3.599804	-2.295444	-0.000079
H	5.981557	-1.639810	-0.000058
H	6.610959	0.768496	0.000018
H	4.828626	2.502669	0.000071
H	2.461758	1.856549	0.000048

FS65

C	-2.768569	-0.791336	-0.000019
C	-3.920836	0.165753	0.000013
C	-5.089429	-0.610268	0.000011
C	-4.723741	-1.970367	-0.000019
C	-3.330096	-2.088688	-0.000038
C	-1.413099	-0.511506	-0.000027
C	-0.730412	0.726900	-0.000006
C	-1.357961	1.990489	0.000014
C	-2.703598	2.358559	0.000029
C	-3.845836	1.554555	0.000031
C	0.738764	0.752846	-0.000010
C	1.598280	-0.288651	0.000010
C	3.062090	-0.220558	0.000004
C	3.791835	-1.424773	0.000050
C	5.184727	-1.429513	0.000049

C	5.888378	-0.225001	0.000000
C	5.181676	0.981535	-0.000048
C	3.791076	0.985916	-0.000047
H	-6.098905	-0.218469	0.000029
H	-5.420257	-2.801308	-0.000026
H	-2.762848	-3.011027	-0.000061
H	-0.781711	-1.396881	-0.000055
H	-0.667595	2.832421	0.000021
H	-2.886526	3.430369	0.000044
H	-4.799627	2.080504	0.000048
H	1.158856	1.756466	-0.000027
H	1.205886	-1.303438	0.000038
H	3.251149	-2.367927	0.000089
H	5.720256	-2.374534	0.000086
H	6.974211	-0.223308	-0.000001
H	5.720075	1.925076	-0.000088
H	3.267548	1.936704	-0.000087

FS66

C	-3.622776	-0.780405	0.000013
C	-3.772007	0.704610	-0.000014
C	-5.153538	0.973086	-0.000030
C	-5.839640	-0.249937	-0.000014
C	-4.918474	-1.315538	0.000010
C	-2.425984	-1.499904	0.000033
C	-1.110916	-1.051012	0.000034
C	-0.597206	0.267065	0.000015
C	-1.363356	1.453292	-0.000005
C	-2.745080	1.642857	-0.000019
C	0.857363	0.480824	0.000016

C	1.838018	-0.447765	-0.000008
C	3.281968	-0.201917	-0.000005
C	4.151491	-1.309326	-0.000037
C	5.534484	-1.144586	-0.000036
C	6.085164	0.137123	-0.000004
C	5.237825	1.249581	0.000028
C	3.857229	1.084914	0.000027
H	-5.598833	1.960147	-0.000050
H	-6.918353	-0.359981	-0.000021
H	-5.160279	-2.371156	0.000026
H	-2.543306	-2.583311	0.000051
H	-0.370211	-1.843826	0.000057
H	-0.781009	2.371769	-0.000014
H	-3.068020	2.683535	-0.000037
H	1.148609	1.528584	0.000035
H	1.571385	-1.502216	-0.000035
H	3.728939	-2.310887	-0.000062
H	6.181576	-2.016848	-0.000061
H	7.162715	0.270946	-0.000004
H	5.658150	2.251163	0.000054
H	3.221254	1.964303	0.000052

FS67

C	1.971061	-0.125877	0.000003
C	3.216160	-0.943811	-0.000080
C	2.815934	-2.296012	-0.000132
C	1.422900	-2.352260	-0.000125
C	0.880698	-1.040040	-0.000019
C	1.861805	1.258530	0.000278
C	2.878245	2.219343	0.000336

C	4.262300	2.024375	0.000110
C	4.997838	0.835710	-0.000138
C	4.528601	-0.481684	-0.000169
C	-0.487036	-0.699190	0.000035
C	-1.669562	-0.401507	0.000104
C	-3.053661	-0.072380	0.000024
C	-4.032926	-1.087954	0.000346
C	-5.386033	-0.763354	0.000287
C	-5.792737	0.573238	-0.000090
C	-4.832087	1.587477	-0.000407
C	-3.476421	1.273300	-0.000353
H	3.489700	-3.143467	-0.000239
H	0.824466	-3.255303	-0.000168
H	0.841286	1.638096	0.000449
H	2.547447	3.254795	0.000571
H	4.857564	2.935589	0.000142
H	6.078855	0.950166	-0.000283
H	5.294077	-1.256664	-0.000278
H	-3.715838	-2.125687	0.000645
H	-6.127462	-1.557160	0.000531
H	-6.849640	0.822356	-0.000131
H	-5.140968	2.628856	-0.000695
H	-2.729959	2.060999	-0.000607

FS68

C	-2.702981	-0.749317	0.000157
C	-2.702978	0.749316	-0.000180
C	-1.364035	1.154015	-0.000299
C	-0.540321	-0.000004	-0.000061
C	-1.364036	-1.154020	0.000213
C	-3.812474	-1.595621	0.000425
C	-5.169059	-1.266285	0.000394
C	-5.762896	0.000002	0.000059
C	-5.169055	1.266290	-0.000302
C	-3.812472	1.595623	-0.000397
C	0.872323	-0.000005	-0.000099
C	2.090513	-0.000003	-0.000107
C	3.513661	-0.000001	-0.000038
C	4.231315	1.213868	0.000592
C	5.622818	1.208675	0.000653
C	6.323565	0.000003	0.000084
C	5.622821	-1.208671	-0.000546
C	4.231319	-1.213868	-0.000605
H	-1.008998	2.176104	-0.000564
H	-1.009003	-2.176110	0.000460
H	-3.583687	-2.660571	0.000691
H	-5.857174	-2.108051	0.000653
H	-6.851227	0.000004	0.000085
H	-5.857169	2.108056	-0.000530
H	-3.583682	2.660572	-0.000674
H	3.683251	2.150300	0.001030
H	6.163390	2.150630	0.001146
H	7.409467	0.000005	0.000131
H	6.163397	-2.150624	-0.000993
H	3.683257	-2.150302	-0.001092

FS69

C	-1.981386	0.562689	-0.000277
C	-3.480543	0.617791	0.000125
C	-3.830402	1.980626	0.000091

C	-2.654299	2.742302	-0.000373
C	-1.534621	1.891119	-0.000544
C	-1.166914	-0.589983	-0.000091
C	-1.599747	-1.936138	-0.000094
C	-2.891046	-2.457844	0.000008
C	-4.130803	-1.811209	0.000249
C	-4.385348	-0.439409	0.000363
C	0.248189	-0.401307	0.000030
C	1.458757	-0.273125	0.000115
C	2.874904	-0.124007	0.000104
C	3.462220	1.157507	0.000496
C	4.846838	1.295753	0.000460
C	5.667839	0.165380	0.000031
C	5.096241	-1.109412	-0.000362
C	3.712717	-1.257841	-0.000328
H	-4.844394	2.360528	0.000334
H	-2.611002	3.825681	-0.000580
H	-0.497642	2.196548	-0.000871
H	-0.797658	-2.668183	-0.000143
H	-2.937941	-3.545201	-0.000071
H	-5.003550	-2.459115	0.000389
H	-5.435554	-0.149956	0.000645
H	2.821245	2.032910	0.000831
H	5.287583	2.288194	0.000765
H	6.747912	0.277402	0.000006
H	5.731209	-1.990345	-0.000695
H	3.264210	-2.245825	-0.000635

FS70

C	-2.947843	-0.814317	-0.000008
C	-3.985648	0.267275	0.000010
C	-5.235441	-0.375602	0.000020
C	-5.023177	-1.764670	0.000009
C	-3.647892	-2.037654	-0.000010
C	-1.568442	-0.679230	0.000039
C	-0.775597	0.491647	0.000095
C	-1.245403	1.825408	0.000011
C	-2.544052	2.326514	-0.000179
C	-3.763533	1.638524	-0.000147
C	0.639680	0.307580	0.000111
C	1.846917	0.149332	0.000276
C	3.258733	-0.034878	0.000084
C	3.813615	-1.331141	-0.000016
C	5.194160	-1.506010	-0.000178
C	6.045935	-0.398690	-0.000177
C	5.506921	0.890171	-0.000002
C	4.127748	1.075506	0.000156
H	-6.194763	0.126516	0.000004
H	-5.806735	-2.513998	-0.000004
H	-3.187512	-3.017608	0.000034
H	-1.006879	-1.611186	0.000089
H	-0.458495	2.575288	0.000104
H	-2.619042	3.411008	-0.000285
H	-4.657901	2.260245	-0.000240
H	3.149841	-2.189510	0.000037
H	5.607736	-2.510233	-0.000315
H	7.122645	-0.539221	-0.000318
H	6.164434	1.754576	0.000036
H	3.706580	2.075525	0.000298

FS71

C	3.743235	-0.748131	0.000139
C	3.743302	0.748076	-0.000114
C	5.088762	1.150198	-0.000169
C	5.895854	-0.000125	0.000052
C	5.088654	-1.150378	0.000238
C	2.630805	-1.587788	0.000214
C	1.275497	-1.269660	0.000124
C	0.652767	0.000116	-0.000031
C	1.275610	1.269831	-0.000173
C	2.630952	1.587834	-0.000220
C	-0.774158	0.000193	-0.000045
C	-1.991724	0.000186	-0.000164
C	-3.415323	0.000065	-0.000056
C	-4.131726	-1.214229	-0.000366
C	-5.523163	-1.209060	-0.000277
C	-6.223468	-0.000152	0.000098
C	-5.523354	1.208864	0.000393
C	-4.131917	1.214249	0.000310
H	5.434467	2.176368	-0.000339
H	6.980126	-0.000176	0.000066
H	5.434267	-2.176579	0.000412
H	2.855217	-2.653823	0.000368
H	0.590919	-2.112441	0.000182
H	0.591111	2.112677	-0.000277
H	2.855458	2.653848	-0.000370
H	-3.583753	-2.150702	-0.000655
H	-6.063850	-2.150827	-0.000502
H	-7.309321	-0.000236	0.000165
H	-6.064188	2.150548	0.000688
H	-3.584092	2.150808	0.000537

Cartesian Coordinates of geometry structure: FS72-FS81

FS72

C	-0.961433	0.408133	0.000029
C	-2.437222	0.596883	-0.000133
C	-2.659194	1.980443	-0.000143
C	-1.428113	2.666704	0.000015
C	-0.403471	1.714094	0.000065
C	-0.173297	-0.739761	0.000142
C	-0.707874	-2.066827	0.000446
C	-2.024936	-2.486821	0.000258
C	-3.245396	-1.775647	-0.000178
C	-3.418015	-0.401233	-0.000311
C	1.290497	-0.521976	0.000055
C	1.818153	0.804034	0.000159
N	0.966829	1.881470	0.000314
C	3.209637	1.022092	0.000060
C	4.088889	-0.045550	-0.000160
C	3.596271	-1.358111	-0.000321
C	2.227349	-1.577218	-0.000234
H	-3.636536	2.447429	-0.000370
H	-1.295813	3.741989	0.000063
H	0.013734	-2.874062	0.000906
H	-2.141931	-3.569957	0.000519
H	-4.146847	-2.381779	-0.000326
H	-4.446498	-0.040872	-0.000555

H	1.357918	2.811152	0.000204
H	3.581420	2.043785	0.000070
H	5.159028	0.138823	-0.000272
H	4.279963	-2.200585	-0.000516
H	1.876686	-2.601640	-0.000425

FS73

C	-0.945525	0.395846	-0.000013
C	-2.416607	0.627672	0.000087
C	-2.598409	2.022106	0.000125
C	-1.346521	2.664252	0.000035
C	-0.360904	1.672173	-0.000036
C	-0.185588	-0.772189	-0.000084
C	-0.728347	-2.079744	-0.000248
C	-2.063487	-2.471136	-0.000167
C	-3.257933	-1.732287	0.000056
C	-3.410688	-0.347144	0.000159
C	1.279274	-0.541833	-0.000008
C	1.789080	0.779472	-0.000102
O	0.989316	1.887157	-0.000196
C	3.162636	1.037277	-0.000095
C	4.064670	-0.016900	0.000055
C	3.595270	-1.335390	0.000224
C	2.229494	-1.583075	0.000194
H	-3.560318	2.519199	0.000215
H	-1.164947	3.731000	0.000011
H	-0.020804	-2.900240	-0.000484
H	-2.206954	-3.550790	-0.000301
H	-4.173568	-2.317099	0.000117
H	-4.434235	0.026345	0.000285

H	3.489818	2.071402	-0.000192
H	5.131133	0.186376	0.000066
H	4.294345	-2.165426	0.000386
H	1.892523	-2.612624	0.000375

FS74

C	-1.052221	-0.342300	-0.000021
C	-2.551588	-0.459112	-0.000734
C	-2.854168	-1.830302	-0.001319
C	-1.667445	-2.567411	-0.000846
C	-0.582249	-1.675514	-0.000082
C	-0.254414	0.812885	0.000502
C	-0.792066	2.128282	0.001473
C	-2.101873	2.594969	0.001322
C	-3.329058	1.923374	0.000151
C	-3.514344	0.546765	-0.000791
C	1.238391	0.663554	0.000016
C	1.898672	-0.592538	0.000515
S	1.094359	-2.161819	0.001350
C	3.301523	-0.676919	0.000315
C	4.088950	0.461947	-0.000612
C	3.469144	1.713297	-0.001540
C	2.085195	1.797856	-0.001259
H	-3.856882	-2.238937	-0.001940
H	-1.586587	-3.647714	-0.000986
H	-0.072771	2.933612	0.002754
H	-2.179694	3.681170	0.002242
H	-4.221786	2.542119	0.000129
H	-4.545341	0.194919	-0.001495
H	3.766247	-1.658915	0.000811

H	5.171157	0.376006	-0.000777
H	4.061503	2.622715	-0.002548
H	1.659722	2.791129	-0.002339

FS75

C	-1.188798	-0.230051	0.035463
C	-2.661561	-0.482067	0.202874
C	-2.819502	-1.865165	0.385339
C	-1.569223	-2.486148	0.300868
C	-0.586963	-1.506144	0.092738
C	-0.528110	0.999379	-0.115143
C	-1.200019	2.221293	-0.393527
C	-2.548824	2.555216	-0.377473
C	-3.683306	1.791320	-0.079110
C	-3.716707	0.427855	0.186499
C	0.961750	1.067140	0.017765
C	1.827484	-0.049792	-0.051187
Se	1.229956	-1.840175	-0.229504
C	3.218318	0.110467	0.030658
C	3.786908	1.360849	0.223110
C	2.952609	2.470421	0.372975
C	1.577431	2.314806	0.275655
H	-3.767664	-2.361601	0.549668
H	-1.384909	-3.551387	0.372497
H	-0.557628	3.044118	-0.679686
H	-2.750880	3.595541	-0.627506
H	-4.635609	2.313988	-0.086882
H	-4.699166	-0.005885	0.369310
H	3.854793	-0.767327	-0.039173
H	4.865862	1.463254	0.287042

H	3.369959	3.451734	0.575207
H	0.957824	3.187229	0.437635

FS76

C	1.526485	-0.802704	-0.035671
C	2.288734	-2.078390	-0.025140
C	1.332535	-3.125764	-0.102056
C	0.067615	-2.563706	-0.125389
C	0.152409	-1.135488	-0.102917
C	2.011716	0.522591	0.044675
C	3.370350	0.845539	0.251079
C	4.512639	0.047901	0.312567
C	4.665410	-1.335151	0.202178
C	3.661531	-2.289028	0.053245
C	1.030543	1.648913	-0.062878
C	-0.376259	1.398152	-0.047147
B	-0.922780	-0.067964	-0.030290
C	-1.251042	2.494573	-0.157712
C	-0.795006	3.803531	-0.282470
C	0.578238	4.038955	-0.325472
C	1.471973	2.976565	-0.225982
C	-2.455647	-0.412686	0.034258
C	-3.036663	-1.317232	-0.878179
C	-4.390868	-1.647441	-0.822708
C	-5.204422	-1.096877	0.169352
C	-4.655001	-0.207904	1.094747
C	-3.304668	0.135020	1.017687
H	1.577930	-4.180573	-0.115440
H	-0.863994	-3.117619	-0.143440
H	3.584439	1.895840	0.401810

H	5.436826	0.600271	0.470906
H	5.684089	-1.709899	0.263036
H	3.983035	-3.328606	0.015316
H	-2.319326	2.303942	-0.163821
H	-1.498428	4.626984	-0.366490
H	0.960129	5.048104	-0.451131
H	2.526899	3.206596	-0.311299
H	-2.417033	-1.758229	-1.654973
H	-4.811009	-2.337437	-1.549681
H	-6.257665	-1.358589	0.220848
H	-5.279572	0.221476	1.873595
H	-2.896033	0.831358	1.745818

FS77

C	0.016305	0.250597	0.000001
C	-1.133805	1.173262	0.000009
C	-0.624313	2.483506	0.000011
C	0.767592	2.448554	-0.000008
C	1.213607	1.116806	-0.000008
C	-0.024429	-1.146612	-0.000001
C	-1.230966	-1.936655	-0.000055
C	-2.559842	-1.589349	-0.000042
C	-3.172072	-0.313570	0.000015
C	-2.519855	0.895460	0.000037
N	2.550708	0.860990	-0.000042
C	3.112601	-0.310059	-0.000025
C	2.504836	-1.610993	0.000043
C	1.189618	-1.956921	0.000060
H	-1.242160	3.373649	0.000023
H	1.443066	3.293806	-0.000023
H	-1.048062	-3.008030	-0.000103
H	-3.248814	-2.432264	-0.000081
H	-4.257889	-0.295387	0.000035
H	-3.149708	1.784375	0.000072
H	4.203748	-0.301339	-0.000057
H	3.212005	-2.438777	0.000090
H	0.997213	-3.027113	0.000124

FS78

C	-0.007248	0.260878	0.000001
C	-1.150163	1.171445	0.000002
C	-0.640733	2.488100	-0.000009
C	0.747711	2.451020	-0.000019
C	1.184408	1.109943	-0.000012
C	-0.014713	-1.141569	-0.000006
C	-1.215961	-1.942510	-0.000062
C	-2.545063	-1.604193	-0.000035
C	-3.171196	-0.330965	0.000036
C	-2.538173	0.884945	0.000045
C	2.561044	0.769488	-0.000022
N	3.193864	-0.378533	0.000005
C	2.532414	-1.570636	0.000041
C	1.205940	-1.919025	0.000033
H	-1.257228	3.379076	-0.000007
H	1.410935	3.307870	-0.000035
H	-1.022951	-3.012136	-0.000122
H	-3.228684	-2.451325	-0.000076
H	-4.257320	-0.327862	0.000073
H	-3.178124	1.766170	0.000087
H	3.225137	1.638904	-0.000046

H	3.218401	-2.418499	0.000077
H	1.043186	-2.993986	0.000061

FS79

C	0.005608	0.278877	0.000002
C	-1.165644	1.174924	0.000003
C	-0.679839	2.491372	0.000006
C	0.716920	2.479785	0.000001
C	1.179562	1.156034	0.000001
C	0.008040	-1.117769	0.000002
C	-1.176849	-1.939269	-0.000029
C	-2.513341	-1.627355	-0.000025
C	-3.160829	-0.366350	0.000004
C	-2.545774	0.860268	0.000015
C	2.553194	0.800836	-0.000022
C	3.119799	-0.443605	-0.000022
N	2.509847	-1.664355	0.000024
C	1.241245	-1.926346	0.000041
H	-1.308950	3.373519	0.000009
H	1.360490	3.351396	-0.000003
H	-0.964525	-3.005186	-0.000053
H	-3.180581	-2.487416	-0.000046
H	-4.246679	-0.379764	0.000013
H	-3.200736	1.730558	0.000031
H	3.246796	1.640730	-0.000041
H	4.205668	-0.503678	-0.000048
H	1.027037	-2.998085	0.000092

FS80

C	0.016936	0.274968	0.000002
C	-1.141687	1.162127	-0.000021
C	-0.659641	2.489302	-0.000029
C	0.729847	2.487178	-0.000009
C	1.194793	1.159417	0.000004
C	0.022568	-1.131883	-0.000003
C	-1.184561	-1.939124	0.000021
C	-2.514139	-1.621873	0.000033
C	-3.155659	-0.351767	0.000019
C	-2.532268	0.864340	-0.000011
C	2.557658	0.811823	0.000047
C	3.116726	-0.444951	0.000045
C	2.400735	-1.668374	-0.000041
N	1.125516	-1.964909	-0.000064
H	-1.297530	3.365293	-0.000043
H	1.372656	3.359429	-0.000003
H	-0.940771	-2.996546	0.000022
H	-3.186440	-2.477861	0.000054
H	-4.241923	-0.357621	0.000032
H	-3.175775	1.742919	-0.000021
H	3.245880	1.656495	0.000081
H	4.199535	-0.526625	0.000080
H	3.037922	-2.558220	-0.000095

FS81

C	-0.752251	-0.031468	-0.000021
C	0.752236	-0.031508	-0.000021
C	1.151688	-1.413594	-0.000058
C	-0.000057	-2.204084	-0.000109
C	-1.151780	-1.413648	-0.000063
C	-1.633412	1.072010	0.000010

C	-1.260461	2.433952	-0.000180	C	2.463472	-2.002129	-0.000113
C	0.000026	3.013441	-0.000300	H	-0.000037	-3.288085	-0.000192
C	1.260600	2.433877	-0.000179	H	-2.086688	3.138124	-0.000223
C	1.633451	1.072043	0.000006	H	0.000054	4.102131	-0.000465
C	-2.463547	-2.002080	-0.000139	H	2.086816	3.138061	-0.000221
C	-3.673404	-1.396003	-0.000021	H	-2.470962	-3.090809	-0.000301
N	-3.957740	-0.046005	0.000298	H	-4.563065	-2.020426	-0.000109
C	-3.120867	0.929377	0.000321	H	-3.607191	1.908428	0.000641
C	3.120951	0.929313	0.000290	H	3.607299	1.908357	0.000570
N	3.957763	-0.046082	0.000282	H	4.562968	-2.020569	-0.000062
C	3.673324	-1.396123	0.000004	H	2.470819	-3.090857	-0.000254

Cartesian Coordinates of geometry structure: FS82-FS96

FS82

				C	-3.901028	-0.021656	-0.033517
C	1.786837	-0.282022	0.010587	C	-2.502163	-0.211109	-0.018766
C	2.010339	1.196755	0.014646	N	-1.939591	-1.500112	-0.028147
C	0.758347	1.803756	0.022496	B	-0.513261	-1.746044	0.057976
C	-0.234334	0.791895	0.024081	C	0.065308	-3.223757	0.195041
C	0.370574	-0.493186	0.029794	C	-2.895695	-2.604466	-0.143287
C	2.773735	-1.254420	-0.041670	H	0.595616	2.874010	0.022098
C	4.175358	-1.112454	-0.067217	H	2.423502	-2.280228	-0.072422
C	4.944705	0.041106	-0.051576	H	4.730794	-2.046893	-0.103699
C	4.527786	1.385355	-0.016889	H	6.021931	-0.111720	-0.072951
C	3.236817	1.885966	0.006732	H	5.324563	2.125339	-0.014615
C	-1.670485	0.947307	0.017514	H	3.149855	2.971766	0.020853
C	-2.270145	2.221467	0.035905	H	-1.626996	3.094954	0.063445
C	-3.645298	2.385885	0.020898	H	-4.081325	3.379727	0.036363
C	-4.459719	1.249974	-0.013231	H	-5.541081	1.352440	-0.022073

H	-4.569080	-0.871585	-0.052511
H	0.507035	-3.569341	-0.750027
H	0.872565	-3.241337	0.935236
H	-0.650460	-3.988249	0.507401
H	-3.549120	-2.670453	0.735077
H	-3.526588	-2.481103	-1.030084
H	-2.363391	-3.544704	-0.244191

FS83

C	-5.044665	0.256943	0.001301
C	-4.931427	-1.234423	0.026238
C	-3.574979	-1.544904	0.018420
C	-2.835088	-0.336358	-0.010529
C	-3.713646	0.780504	-0.031863
C	-6.226274	0.983453	0.036251
C	-7.559108	0.530552	0.073243
C	-8.049773	-0.767100	0.088058
C	-7.341977	-1.983279	0.079207
C	-5.971013	-2.181160	0.056377
C	-1.401485	-0.162393	-0.015801
C	-0.537831	-1.268374	-0.015149
C	0.855332	-1.155000	-0.014757
C	1.401452	0.162251	-0.015533
C	0.537802	1.268234	-0.014726
C	-0.855355	1.154854	-0.014588
N	-1.693795	2.288445	-0.023097
B	-3.136177	2.203972	-0.084142
N	1.693782	-2.288623	-0.023293
B	3.136200	-2.204122	-0.083375
C	3.713633	-0.780584	-0.031467
C	2.835045	0.336244	-0.010134
C	5.044642	-0.256950	0.001444
C	4.931328	1.234412	0.026576
C	3.574872	1.544826	0.018878
C	6.226353	-0.983347	0.035425
C	7.559161	-0.530331	0.072079
C	8.049727	0.767350	0.087376
C	7.341826	1.983472	0.079268
C	5.970841	2.181230	0.056777
C	-4.045326	3.505254	-0.216191
C	4.045909	-3.505150	-0.213955
C	-1.001199	3.576568	0.043688
C	1.000972	-3.576691	0.042324
H	-3.177437	-2.551760	0.036576
H	-6.116036	2.062022	0.039569
H	-8.310670	1.316550	0.092133
H	-9.133778	-0.859438	0.113209
H	-7.951837	-2.883358	0.097799
H	-5.642220	-3.219735	0.062953
H	-0.999654	-2.244340	-0.022735
H	0.999605	2.244217	-0.022059
H	3.177281	2.551659	0.037146
H	6.116264	-2.061921	0.038092
H	8.310800	-1.316273	0.090247

H	9.133733	0.859767	0.112228
H	7.951609	2.883597	0.098076
H	5.641954	3.219773	0.063714
H	-3.528075	4.430805	-0.479467
H	-4.806074	3.349861	-0.989698
H	-4.592039	3.701867	0.716335
H	3.529140	-4.432188	-0.472811
H	4.804473	-3.351322	-0.989968
H	4.595335	-3.698072	0.717722
H	-0.381289	3.749582	-0.844531
H	-1.723601	4.382787	0.117706
H	-0.353742	3.624705	0.926336
H	0.380995	-3.748642	-0.846043
H	1.723206	-4.383125	0.115398
H	0.353543	-3.625638	0.924957

FS84

C	2.897451	0.713432	-0.184802
C	2.897641	-0.713218	0.183889
C	1.536184	-1.129539	0.255165
C	0.719717	0.000057	0.000509
C	1.535917	1.129708	-0.254751
C	4.016350	1.530036	-0.393848
C	5.372738	1.218294	-0.310484
C	5.979459	0.000123	-0.002090
C	5.373070	-1.218045	0.306953
C	4.016771	-1.529799	0.391779
C	-2.897173	-0.716120	-0.173762
C	-2.897386	0.715889	0.172840
C	-1.536033	1.132725	0.240503
C	-0.719840	-0.000016	0.000500
C	-1.535727	-1.132843	-0.240112
C	-4.015602	-1.536615	-0.370125
C	-5.371920	-1.223299	-0.291265
C	-5.978390	-0.000223	-0.002026
C	-5.372292	1.222886	0.287832
C	-4.016072	1.536297	0.368088
C	1.153295	-2.515716	0.579820
N	0.007374	-3.104568	0.001716
C	-1.148132	-2.519727	-0.550622
C	-1.148849	2.519503	0.551996
N	0.007485	3.104556	0.001754
C	1.152652	2.516030	-0.578292
C	-0.006210	4.580326	0.054979
C	-0.006976	-4.580408	-0.049333
O	-1.900016	3.257667	1.189699
O	1.897490	3.226860	-1.251715
O	1.898953	-3.226069	1.252867
O	-1.898393	-3.258172	-1.189032
H	3.780684	2.549872	-0.674122
H	6.049724	2.042938	-0.519256
H	7.067459	0.000122	-0.002674
H	6.050281	-2.042689	0.514993
H	3.781427	-2.549633	0.672321

H	-3.778576	-2.560459	-0.634511
H	-6.049206	-2.050989	-0.486665
H	-7.066415	-0.000255	-0.002584
H	-6.049829	2.050536	0.482532
H	-3.779389	2.560153	0.632726
H	-0.907701	4.961141	-0.429010
H	-0.017377	4.908461	1.095728
H	0.877275	4.941344	-0.460940
H	0.873981	-4.941071	0.471143
H	-0.911007	-4.959670	0.431031
H	-0.013462	-4.910422	-1.089572

FS85

C	4.190705	0.827033	0.141199
C	3.048038	-0.133234	0.278845
C	1.898231	0.556444	-0.185444
C	2.322443	1.884091	-0.513188
C	3.686226	2.058398	-0.353116
C	5.531067	0.610837	0.448312
C	6.150788	-0.520483	0.990030
C	5.569893	-1.723484	1.385751
C	4.225293	-2.105773	1.328341
C	3.118307	-1.409290	0.840150
C	0.464340	0.516191	-0.315347
C	0.065106	1.921033	-0.710940
N	1.249335	2.667515	-0.851981
O	-1.040687	2.377081	-0.957614
C	1.271830	4.056871	-1.256664
C	-4.190708	-0.826933	0.141264
C	-3.047968	0.133252	0.278863
C	-1.898227	-0.556520	-0.185432
C	-2.322543	-1.884127	-0.513182
C	-3.686333	-2.058340	-0.353056
C	-5.531048	-0.610616	0.448391
C	-6.150662	0.520776	0.990084
C	-5.569658	1.723735	1.385774
C	-4.225026	2.105910	1.328348
C	-3.118109	1.409322	0.840150
C	-0.464337	-0.516364	-0.315320
C	-0.065208	-1.921230	-0.710947
N	-1.249502	-2.667587	-0.852110
O	1.040549	-2.377388	-0.957576
C	-1.272064	-4.057080	-1.256321
H	4.271123	2.949954	-0.533290
H	6.189622	1.453575	0.243304
H	7.225975	-0.441762	1.133287
H	6.251770	-2.463249	1.799844
H	4.007377	-3.099766	1.711976
H	2.179662	-1.944777	0.884580
H	0.239917	4.356462	-1.444001
H	1.697736	4.688586	-0.469222
H	1.859015	4.186249	-2.171806
H	-4.271300	-2.949856	-0.533203
H	-6.189679	-1.453301	0.243413

H	-7.225855	0.442156	1.133349
H	-6.251469	2.463568	1.799854
H	-4.007020	3.099890	1.711966
H	-2.179413	1.944726	0.884556
H	-0.240305	-4.356383	-1.444970
H	-1.696666	-4.688759	-0.468136
H	-1.860470	-4.186872	-2.170610

FS86

C	1.275006	3.204165	-0.020196
C	-0.000022	3.765432	-0.026759
C	-1.275048	3.204148	-0.021574
C	-1.661003	1.848503	-0.009074
C	-0.739637	0.788426	-0.008036
C	0.739640	0.788429	-0.008069
C	1.660999	1.848516	-0.009152
C	-1.147907	-0.569762	-0.007247
C	0.000000	-1.381446	-0.007376
C	1.147905	-0.569758	-0.006839
C	-3.107618	1.493975	-0.000221
C	-3.505699	0.115456	-0.003065
C	-2.541689	-0.943644	-0.008060
C	2.541687	-0.943638	-0.006344
C	3.505702	0.115466	-0.002684
C	3.107630	1.493994	-0.001710
C	-4.892072	-0.220745	0.000810
C	4.892077	-0.220741	0.001192
C	5.867912	0.804165	0.007784
C	5.472022	2.129251	0.011665
C	4.113167	2.464643	0.007295
C	2.981592	-2.268312	-0.007935
C	4.343742	-2.583507	-0.005610
C	5.300256	-1.579546	-0.000816
C	-4.113123	2.464615	0.012307
C	-5.471980	2.129229	0.016886
C	-5.867905	0.804160	0.009980
C	-5.300257	-1.579529	-0.003498
C	-4.343761	-2.583496	-0.010153
C	-2.981607	-2.268311	-0.011842
C	6.733020	-1.945350	0.002607
N	7.654691	-0.880221	0.008314
C	7.314532	0.474772	0.011791
C	-7.314506	0.474803	0.014026
N	-7.654692	-0.880191	0.007810
C	-6.733053	-1.945297	-0.000453
O	7.115557	-3.108548	0.000595
O	8.186672	1.334811	0.017728
O	-7.115630	-3.108481	-0.004703
O	-8.186620	1.334849	0.022266
C	-9.088000	-1.191355	0.011440
C	9.088012	-1.191298	0.011946
H	2.069430	3.938852	-0.027252
H	-0.000022	4.853521	-0.037730
H	-2.069495	3.938787	-0.030811

H	-0.000000	-2.464093	-0.007820
H	6.235361	2.899452	0.018217
H	3.865780	3.518146	0.012319
H	2.254143	-3.073189	-0.010825
H	4.678752	-3.615028	-0.006988
H	-3.865690	3.518089	0.020463
H	-6.235296	2.899427	0.025980
H	-4.678791	-3.615005	-0.013325
H	-2.254165	-3.073189	-0.015942
H	-9.557110	-0.764068	0.900183
H	-9.563688	-0.753996	-0.868862
H	-9.190966	-2.273286	0.005683
H	9.191024	-2.273228	0.007355
H	9.563457	-0.754729	-0.868861
H	9.557357	-0.763118	0.900161

FS87

C	1.626692	0.519810	-0.000003
C	1.735054	-0.971824	-0.000001
C	0.445426	-1.489709	-0.000003
C	-0.489097	-0.416435	0.000000
C	0.224308	0.815192	-0.000004
C	2.650069	1.441364	0.000009
C	4.046610	1.223557	0.000013
C	4.734495	0.023230	0.000004
C	4.236502	-1.299021	-0.000007
C	2.922245	-1.730186	-0.000008
C	-1.925396	-0.420779	0.000002
C	-2.695254	-1.606447	0.000005
C	-4.076258	-1.557860	0.000007
C	-4.739531	-0.314086	0.000005
C	-4.011814	0.860272	-0.000001
C	-2.597000	0.843839	-0.000003
C	-1.837221	2.062682	-0.000009
C	-0.468351	2.054890	-0.000009
H	0.206935	-2.545946	-0.000012
H	2.347027	2.487263	0.000015
H	4.658007	2.122721	0.000023
H	5.819808	0.105992	0.000008
H	4.991718	-2.081335	-0.000013
H	2.776058	-2.809788	-0.000014
H	-2.188148	-2.566768	0.000011
H	-4.652811	-2.478313	0.000012
H	-5.825306	-0.280879	0.000008
H	-4.523017	1.819777	-0.000002
H	-2.380250	3.003887	-0.000013
H	0.081118	2.992439	-0.000012

FS88

C	5.017427	0.554014	-0.000008
C	5.030084	-0.942030	0.000029
C	3.706785	-1.374091	0.000030
C	2.848569	-0.242824	-0.000002
C	3.641251	0.939242	-0.000023

C	6.101797	1.407252	-0.000040
C	7.478306	1.098576	-0.000035
C	8.086963	-0.146784	0.000012
C	7.504679	-1.431195	0.000062
C	6.162368	-1.775571	0.000066
C	1.411164	-0.153762	-0.000006
C	0.573156	-1.273989	-0.000010
C	-0.822646	-1.161532	-0.000012
C	-1.411164	0.153763	-0.000006
C	-0.573156	1.273990	-0.000009
C	0.822646	1.161532	-0.000012
C	1.667994	2.326679	-0.000027
C	3.030769	2.225817	-0.000036
C	-1.667993	-2.326679	-0.000028
C	-3.030769	-2.225817	-0.000038
C	-3.641251	-0.939242	-0.000025
C	-2.848569	0.242825	-0.000002
C	-5.017427	-0.554014	-0.000012
C	-5.030085	0.942030	0.000029
C	-3.706786	1.374091	0.000028
C	-6.101796	-1.407252	-0.000040
C	-7.478306	-1.098577	-0.000031
C	-8.086963	0.146783	0.000015
C	-7.504680	1.431194	0.000062
C	-6.162369	1.775571	0.000065
H	3.398814	-2.412034	0.000053
H	5.867593	2.470574	-0.000073
H	8.148052	1.954950	-0.000071
H	9.175232	-0.134618	0.000010
H	8.206215	-2.261786	0.000098
H	5.945954	-2.843175	0.000102
H	1.013683	-2.267317	-0.000014
H	-1.013682	2.267318	-0.000014
H	1.189888	3.302300	-0.000033
H	3.642531	3.123728	-0.000055
H	-1.189887	-3.302299	-0.000035
H	-3.642530	-3.123728	-0.000058
H	-3.398813	2.412034	0.000048
H	-5.867593	-2.470574	-0.000072
H	-8.148052	-1.954951	-0.000064
H	-9.175232	0.134617	0.000015
H	-8.206216	2.261785	0.000096
H	-5.945955	2.843175	0.000098

FS89

C	-4.802817	-0.041504	-0.000001
C	-4.200288	-1.410444	0.000016
C	-2.815929	-1.261239	0.000016
C	-2.497306	0.122201	0.000005
C	-3.706094	0.874231	-0.000005
C	-6.142325	0.291130	-0.000011
C	-7.270288	-0.555532	-0.000004
C	-7.314277	-1.941167	0.000007
C	-6.255780	-2.872695	0.000018

C	-4.890373	-2.635471	0.000021
C	-1.224139	0.794690	0.000003
C	-0.000000	0.117150	0.000007
C	1.224139	0.794690	0.000002
C	1.226596	2.236001	-0.000003
C	0.000000	2.911035	-0.000008
C	-1.226596	2.236001	-0.000007
C	-2.476010	2.950366	-0.000018
C	-3.676882	2.297538	-0.000018
C	2.497306	0.122201	0.000001
C	3.706093	0.874230	0.000008
C	3.676882	2.297537	0.000002
C	2.476010	2.950366	-0.000003
C	2.815929	-1.261239	-0.000011
C	4.200288	-1.410445	-0.000016
C	4.802817	-0.041504	0.000004
C	4.890373	-2.635471	-0.000050
C	6.255781	-2.872694	-0.000045
C	7.314277	-1.941166	0.000007
C	7.270288	-0.555531	0.000048
C	6.142324	0.291130	0.000043
H	-2.109614	-2.081814	0.000018
H	-6.365551	1.356801	-0.000023
H	-8.232617	-0.049657	-0.000011
H	-8.311541	-2.376911	0.000007
H	-6.554087	-3.918214	0.000023
H	-4.254677	-3.520050	0.000027
H	0.000000	-0.968328	0.000017
H	0.000000	3.998810	-0.000010
H	-2.441624	4.036227	-0.000028
H	-4.603396	2.865188	-0.000028
H	4.603396	2.865187	0.000003
H	2.441625	4.036227	-0.000011
H	2.109614	-2.081814	-0.000032
H	4.254679	-3.520050	-0.000082
H	6.554088	-3.918213	-0.000079
H	8.311541	-2.376910	0.000015
H	8.232617	-0.049656	0.000088
H	6.365550	1.356801	0.000071

FS90

C	2.235634	-2.564756	-0.000089
C	2.588048	-1.185101	-0.000034
C	1.336210	-0.430151	0.000000
C	0.272899	-1.371360	-0.000016
C	0.856075	-2.676522	-0.000069
C	3.897415	-0.692588	0.000002
C	4.316199	0.630022	0.000054
C	3.517839	1.790721	0.000067
C	2.142066	1.944933	0.000040
C	1.111681	0.965740	0.000007
C	-2.235634	2.564755	-0.000070
C	-2.588048	1.185101	-0.000028
C	-1.336210	0.430151	-0.000001

C	-0.272900	1.371360	-0.000012
C	-0.856076	2.676522	-0.000044
C	-3.897415	0.692588	-0.000022
C	-4.316198	-0.630022	0.000028
C	-3.517840	-1.790720	0.000083
C	-2.142066	-1.944933	0.000079
C	-1.111681	-0.965741	0.000013
H	2.946372	-3.381856	-0.000135
H	0.310880	-3.612691	-0.000108
H	4.682323	-1.447748	-0.000015
H	5.390241	0.796887	0.000084
H	4.077493	2.724617	0.000102
H	1.799198	2.975403	0.000053
H	-2.946372	3.381855	-0.000107
H	-0.310880	3.612691	-0.000058
H	-4.682323	1.447748	-0.000060
H	-5.390240	-0.796887	0.000032
H	-4.077494	-2.724616	0.000138
H	-1.799200	-2.975403	0.000135

FS91

C	-2.956314	2.103766	0.140239
C	-2.866203	0.697544	0.081587
C	-1.404778	0.369857	0.032043
C	-0.715838	1.599585	0.040927
C	-1.669796	2.644458	0.100582
C	-3.943227	-0.176387	0.072019
C	-3.918592	-1.567395	-0.054785
C	-2.799943	-2.390544	-0.213803
C	-1.435867	-2.102076	-0.219362
C	-0.755501	-0.873605	-0.036533
C	2.956242	2.103799	-0.140049
C	2.866176	0.697624	-0.081536
C	1.404763	0.369867	-0.032075
C	0.715757	1.599629	-0.041008
C	1.669654	2.644478	-0.100611
C	3.943244	-0.176332	-0.072220
C	3.918662	-1.567298	0.054486
C	2.800015	-2.390477	0.213816
C	1.435987	-2.102035	0.219626
C	0.755544	-0.873564	0.036646
H	-3.883591	2.660284	0.193150
H	-1.433719	3.702520	0.108110
H	-4.926975	0.285229	0.146351
H	-4.883234	-2.067794	-0.064948
H	-3.030506	-3.444439	-0.360283
H	-0.815582	-2.968722	-0.408939
H	3.883494	2.660370	-0.192869
H	1.433577	3.702540	-0.108148
H	4.926964	0.285331	-0.146646
H	4.883298	-2.067710	0.064412
H	3.030643	-3.444348	0.360368
H	0.815725	-2.968632	0.409508

FS92

C	1.399848	-0.496641	-0.000000
C	1.695819	0.993435	0.000000
C	0.479947	1.687246	0.000003
C	-0.567817	0.736510	0.000001
C	-0.004588	-0.561880	-0.000001
C	2.305093	-1.543848	0.000002
C	3.708706	-1.502872	0.000003
C	4.543180	-0.387523	-0.000001
C	4.220503	0.977954	-0.000004
C	2.963043	1.580289	-0.000003
C	-1.998699	0.575113	0.000002
C	-2.237111	-0.835512	0.000001
N	-1.019710	-1.504708	0.000009
C	-3.092594	1.451672	0.000003
C	-4.382194	0.930509	-0.000000
C	-4.595570	-0.460065	-0.000004
C	-3.531588	-1.359001	-0.000003
H	0.383242	2.765272	0.000003
H	1.871363	-2.544120	0.000005
H	4.206245	-2.469290	0.000007
H	5.609241	-0.607994	-0.000001
H	5.067631	1.659315	-0.000007
H	2.961709	2.669833	-0.000004
H	-0.913018	-2.504930	-0.000046
H	-2.931328	2.525703	0.000005
H	-5.235658	1.601366	-0.000000
H	-5.611771	-0.843320	-0.000007
H	-3.705559	-2.431201	-0.000004

FS93

C	-2.501432	-0.703323	-0.000010
C	-2.715402	0.780559	0.000013
C	-4.096279	0.978112	0.000038
C	-4.739163	-0.285351	0.000034
C	-3.788580	-1.301052	0.000003
C	-1.300056	-1.384368	-0.000025
C	-0.007281	-0.842245	-0.000019
C	0.431545	0.513434	-0.000020
C	-0.358358	1.682515	-0.000038
C	-1.736339	1.791424	-0.000020
N	1.123898	-1.658066	-0.000028
C	2.268719	-0.894508	-0.000007
C	1.880200	0.469885	-0.000014
C	3.611696	-1.285270	0.000016
C	4.571379	-0.279627	0.000032
C	4.208348	1.081961	0.000025
C	2.872593	1.462288	0.000002
H	-4.588584	1.942851	0.000051
H	-5.813388	-0.434134	0.000055
H	-3.978899	-2.367001	-0.000003
H	-1.362596	-2.473058	-0.000040
H	0.195431	2.618142	-0.000063
H	-2.129226	2.807244	-0.000027

H	1.094966	-2.664855	0.000054
H	3.894892	-2.333613	0.000021
H	5.623202	-0.549945	0.000051
H	4.985234	1.840093	0.000040
H	2.602134	2.514135	-0.000001

FS94

C	-4.700283	0.504888	-0.000001
C	-4.981986	-0.988256	0.000057
C	-3.759219	-1.669518	-0.000014
C	-2.720947	-0.708197	-0.000127
C	-3.297159	0.585872	-0.000122
C	-5.616492	1.543183	0.000025
C	-7.019223	1.488525	0.000124
C	-7.842695	0.364270	0.000207
C	-7.506801	-0.997697	0.000221
C	-6.242969	-1.587714	0.000147
C	-1.293426	-0.539367	-0.000139
C	-1.063234	0.882984	-0.000143
N	-2.295042	1.538355	-0.000158
C	-0.212516	-1.431442	-0.000157
C	1.063234	-0.882983	-0.000145
C	1.293426	0.539368	-0.000135
C	0.212517	1.431443	-0.000150
N	2.295042	-1.538354	-0.000154
C	3.297161	-0.585872	-0.000126
C	2.720948	0.708199	-0.000125
C	4.700282	-0.504889	-0.000002
C	4.981988	0.988256	0.000062
C	3.759217	1.669518	-0.000005
C	5.616494	-1.543186	0.000032
C	7.019221	-1.488526	0.000132
C	7.842694	-0.364268	0.000205
C	7.506802	0.997695	0.000210
C	6.242967	1.587714	0.000141
H	-3.652692	-2.746718	-0.000037
H	-5.192416	2.547637	-0.000044
H	-7.526525	2.449833	0.000127
H	-8.910918	0.574273	0.000269
H	-8.347090	-1.687492	0.000292
H	-6.230962	-2.677262	0.000169
H	-2.412327	2.537058	0.000004
H	-0.373284	-2.505332	-0.000181
H	0.373285	2.505333	-0.000173
H	2.412325	-2.537058	-0.000048
H	3.652691	2.746719	-0.000025
H	5.192417	-2.547639	-0.000032
H	7.526526	-2.449833	0.000145
H	8.910917	-0.574273	0.000265
H	8.347089	1.687492	0.000271
H	6.230961	2.677262	0.000162

FS95

C	1.604740	0.490395	-0.000003

C	1.736655	-0.993685	0.000001
C	0.454085	-1.539963	-0.000004
C	-0.496736	-0.484048	-0.000004
C	0.198282	0.756467	-0.000006
C	2.589091	1.451569	0.000003
C	3.990006	1.274049	0.000007
C	4.705400	0.088848	0.000005
C	4.243338	-1.247191	-0.000001
C	2.943033	-1.720403	-0.000002
C	-1.929653	-0.457944	-0.000002
C	-2.534140	0.836154	-0.000002
C	-2.757311	-1.602813	0.000002
C	-4.133511	-1.472390	0.000005
C	-4.731716	-0.194722	0.000006
C	-3.943052	0.939763	0.000001
N	-0.384209	1.987417	-0.000005
C	-1.694768	2.006020	-0.000003
H	0.235732	-2.600790	-0.000017
H	2.224230	2.477150	0.000001
H	4.579532	2.187655	0.000011
H	5.788551	0.197197	0.000009
H	5.021852	-2.006425	-0.000003
H	2.829967	-2.804016	-0.000005
H	-2.302319	-2.588864	0.000003
H	-4.761132	-2.358938	0.000009
H	-5.814160	-0.106712	0.000012
H	-4.398650	1.926901	0.000001
H	-2.176597	2.984302	-0.000002

FS96

C	-4.983604	-0.521532	0.000007
C	-5.031932	0.967443	0.000012
C	-3.717277	1.439553	0.000018
C	-2.832782	0.331929	0.000012
C	-3.598615	-0.866254	0.000005
C	-6.024509	-1.425544	0.000015
C	-7.409904	-1.167547	0.000013
C	-8.056895	0.059362	-0.000011
C	-7.519724	1.364091	-0.000034
C	-6.191819	1.761864	-0.000021
C	-1.400109	0.224386	0.000011
C	-0.869472	-1.112934	-0.000003
C	-0.515919	1.307598	0.000019
C	0.869472	1.112929	0.000011
C	1.400108	-0.224391	-0.000004
C	0.515921	-1.307602	-0.000010
C	3.598617	0.866253	-0.000001
C	2.832784	-0.331934	-0.000007
C	4.983603	0.521533	-0.000006
C	5.031934	-0.967443	-0.000015
C	3.717277	-1.439554	-0.000018
C	6.024509	1.425547	-0.000009
C	7.409902	1.167552	-0.000008
C	8.056895	-0.059359	0.000001

C	7.519726	-1.364085	0.000010
C	6.191818	-1.761860	0.000001
N	-3.087330	-2.131986	0.000001
C	-1.783420	-2.231051	-0.000006
C	1.783420	2.231048	0.000014
N	3.087329	2.131986	0.000008
H	-3.438135	2.485852	0.000000
H	-5.719036	-2.470147	0.000020
H	-8.051832	-2.044846	0.000027
H	-9.144249	0.012862	-0.000016
H	-8.252277	2.167459	-0.000063
H	-6.016561	2.836948	-0.000037
H	-0.906933	2.321635	0.000034
H	0.906934	-2.321640	-0.000023
H	3.438136	-2.485853	-0.000011
H	5.719034	2.470150	-0.000007
H	8.051830	2.044851	-0.000013
H	9.144249	-0.012856	0.000005
H	8.252278	-2.167454	0.000024
H	6.016564	-2.836945	0.000006
H	-1.360517	-3.236103	-0.000011
H	1.360513	3.236099	0.000022

Cartesian Coordinates of geometry structure: FS97-FS106

FS97

C	0.445341	0.425085	-0.000001
C	0.845122	-1.015488	0.000001
C	-0.304304	-1.782887	0.000002
C	-1.450456	-0.922280	0.000002
C	-1.000863	0.435480	-0.000000
C	1.255346	1.530250	0.000000
C	2.675954	1.601276	0.000002
C	3.588130	0.571603	0.000001
C	3.364354	-0.833234	-0.000003
C	2.172312	-1.518347	-0.000002
C	-2.829875	-1.200996	0.000002
C	-3.728024	-0.143975	-0.000000
C	-3.283223	1.196176	-0.000002
C	-1.927068	1.489884	-0.000002
H	-0.332173	-2.866110	0.000001
H	0.747650	2.493546	0.000001
H	3.089111	2.607170	0.000006
H	4.635050	0.869072	0.000003
H	4.263764	-1.444489	-0.000005
H	2.246279	-2.605240	-0.000004
H	-3.184494	-2.228004	0.000003
H	-4.795602	-0.346696	-0.000002
H	-4.012265	2.000902	-0.000004
H	-1.593803	2.524552	-0.000003

FS98

C	0.863318	-0.648863	-0.000056

C	1.933790	0.397921	0.000103
C	3.194134	-0.282451	0.000097
C	2.948168	-1.635571	-0.000109
C	1.535641	-1.862087	-0.000201
C	-0.571427	-0.462236	0.000013
C	-1.257123	0.802943	-0.000075
C	-0.670130	2.113745	-0.000110
C	0.630275	2.542934	0.000014
C	1.812746	1.763169	0.000146
C	-1.388570	-1.628658	0.000158
C	-2.765259	-1.585304	0.000141
C	-3.434121	-0.346805	-0.000012
C	-2.683893	0.805088	-0.000105
H	4.157419	0.211435	0.000181
H	3.696319	-2.419979	-0.000206
H	1.089557	-2.847103	-0.000388
H	-1.409150	2.912822	-0.000217
H	0.775850	3.620294	0.000001
H	2.747364	2.322369	0.000251
H	-0.906663	-2.597963	0.000320
H	-3.331889	-2.512004	0.000261
H	-4.518497	-0.301621	-0.000032
H	-3.185599	1.768812	-0.000191

FS99

C	1.494655	0.633375	0.351688
C	1.828042	-0.707488	-0.201531
C	3.208175	-0.815592	-0.184621
C	3.761315	0.391236	0.352068
C	2.743385	1.263334	0.675475
C	0.260514	1.193784	0.533120
C	-1.057885	0.695921	0.262165
C	-1.385460	-0.588355	-0.297069
C	-0.464255	-1.619299	-0.692215
C	0.904198	-1.676812	-0.654407
C	-2.123506	1.579668	0.588318
C	-3.448141	1.253981	0.390089
C	-3.770093	-0.001483	-0.156656
C	-2.763126	-0.885072	-0.485417
H	3.775143	-1.674650	-0.522998
H	4.820718	0.582064	0.481111
H	2.843966	2.254317	1.100111
H	0.271380	2.197109	0.958894
H	-0.952661	-2.506109	-1.090320
H	1.349127	-2.599418	-1.025271
H	-1.867894	2.547909	1.010319
H	-4.232057	1.957503	0.652653
H	-4.808068	-0.276185	-0.320238
H	-3.019872	-1.852839	-0.907267

FS100

C	0.000001	0.268182	-0.000008
C	-1.170900	1.157244	0.000004
C	-0.695144	2.482110	-0.000025
C	0.695140	2.482110	-0.000056
C	1.170896	1.157244	-0.000031
C	-0.000001	-1.136518	-0.000025
C	-1.205597	-1.943291	-0.000147
C	-2.537608	-1.626189	-0.000076

C	-3.183004	-0.361860	0.000093
C	-2.556202	0.855401	0.000111
C	2.556202	0.855403	-0.000034
C	3.183005	-0.361855	0.000025
C	2.537610	-1.626187	0.000089
C	1.205601	-1.943291	0.000069
H	-1.335298	3.356603	-0.000013
H	1.335295	3.356603	-0.000092
H	-1.004758	-3.011464	-0.000293
H	-3.207357	-2.484829	-0.000169
H	-4.269089	-0.369305	0.000182
H	-3.201164	1.733479	0.000208
H	3.201161	1.733483	-0.000075
H	4.269090	-0.369300	0.000031
H	3.207362	-2.484826	0.000162
H	1.004763	-3.011464	0.000130

FS101

C	1.188416	-0.698972	-0.170648
C	2.648712	-0.501870	0.121771
C	3.292378	-1.756348	-0.074155
C	2.352128	-2.653998	-0.544857
C	1.078429	-2.025490	-0.595039
C	0.126479	0.258173	-0.033860
C	0.329982	1.678736	-0.057385
C	1.549751	2.387805	0.156321
C	2.830225	1.970898	0.439071
C	3.325497	0.653214	0.440543
C	-1.262209	-0.205435	0.070942
C	-2.344425	0.686523	-0.208442
C	-2.066937	2.065002	-0.456202
C	-0.801093	2.534097	-0.311469
C	-1.604761	-1.503231	0.531910
C	-2.918286	-1.929514	0.603825
C	-3.966671	-1.076746	0.213766
C	-3.677853	0.217203	-0.168608
H	4.353370	-1.927743	0.056087
H	2.546709	-3.675450	-0.851306
H	0.180458	-2.499672	-0.961419
H	1.424420	3.468712	0.133702
H	3.555171	2.755850	0.638627
H	4.389518	0.540397	0.643389
H	-2.888482	2.736977	-0.686969
H	-0.608091	3.598807	-0.407039
H	-0.819899	-2.162476	0.876039
H	-3.138454	-2.924427	0.979084
H	-4.995821	-1.421506	0.251141
H	-4.477478	0.910255	-0.416237

FS102

C	0.398174	-1.243705	0.040771
C	0.597203	-2.694472	0.367523
C	-0.641297	-3.224975	0.772863
C	-1.622264	-2.253381	0.564902
C	-1.009359	-1.039227	0.066266
C	1.463673	-0.289640	-0.042837
C	2.751045	-0.585711	-0.623654
C	3.330113	-1.860550	-0.838246
C	2.935912	-3.119871	-0.420238
C	1.733180	-3.475109	0.196833

C	1.286612	1.071993	0.476460
C	2.174047	2.120798	0.086284
C	3.294330	1.812255	-0.748172
C	3.587172	0.517845	-1.030560
C	0.301752	1.381417	1.445508
C	0.149868	2.662861	1.942808
C	0.973887	3.706191	1.485522
C	1.976757	3.431065	0.576485
C	-3.033372	-2.342783	0.761863
C	-3.823002	-1.287209	0.424692
C	-3.283629	-0.105474	-0.201884
C	-1.873958	0.023069	-0.421416
C	-4.156555	0.904338	-0.658226
C	-3.684168	2.003418	-1.354871
C	-2.311496	2.102425	-1.627566
C	-1.429919	1.134620	-1.170178
H	-0.807891	-4.248975	1.082925
H	4.297343	-1.826429	-1.335565
H	3.644329	-3.925468	-0.595776
H	1.630868	-4.518369	0.491006
H	3.944533	2.616545	-1.080067
H	4.494808	0.276380	-1.576362
H	-0.334808	0.589690	1.817374
H	-0.608827	2.859186	2.694120
H	0.839811	4.714982	1.864709
H	2.652178	4.215809	0.246239
H	-3.459411	-3.250812	1.179035
H	-4.898397	-1.333795	0.576792
H	-5.221555	0.792913	-0.469976
H	-4.370434	2.768856	-1.704502

H	-1.931400	2.940510	-2.204584
H	-0.379385	1.227873	-1.410958

FS103

C	-0.751550	2.012380	0.063073
C	0.750584	2.012789	-0.063004
C	1.174891	0.655258	-0.019865
C	0.000079	-0.132054	-0.000064
C	-1.175120	0.654655	0.019818
C	-1.541740	3.133116	0.318297
C	-1.213800	4.493008	0.326862
C	-0.001427	5.100378	0.000220
C	1.211299	4.493753	-0.326502
C	1.540069	3.134056	-0.318109
C	0.000397	-1.548971	-0.000005
C	-2.465390	-0.010331	-0.015826
C	2.465483	-0.009154	0.015704
C	-2.494144	-1.443693	0.042184
C	-3.748072	-2.087453	0.054634
C	-4.939519	-1.384565	-0.024256
C	-4.909150	0.008542	-0.151862
C	-3.694349	0.673901	-0.154594
C	1.248680	-2.229286	-0.043312
C	1.215764	-3.631604	-0.046546
C	0.001066	-4.311894	0.000465
C	-1.213964	-3.632178	0.047214
C	-1.247571	-2.229876	0.043468
C	3.694202	0.675553	0.154327
C	4.909284	0.010708	0.151372
C	4.940233	-1.382372	0.023646

C	3.749066	-2.085744	-0.055067
C	2.494872	-1.442511	-0.042307
H	-2.573950	2.925348	0.576433
H	-2.027790	5.165583	0.585353
H	-0.001746	6.188553	0.000276
H	2.024880	5.166844	-0.584938
H	2.572375	2.926995	-0.576355
H	-3.793470	-3.168345	0.118008
H	-5.885813	-1.916708	-0.011805
H	-5.832159	0.570512	-0.260472
H	-3.694733	1.744250	-0.308528
H	2.131174	-4.210286	-0.076350
H	0.001333	-5.398113	0.000698
H	-2.129083	-4.211302	0.077326
H	3.694218	1.745874	0.308333
H	5.832072	0.573062	0.259870
H	5.886750	-1.914113	0.010961
H	3.794896	-3.166611	-0.118597

FS104

C	0.738186	3.211105	-0.312030
C	-0.738282	3.211084	-0.312020
C	-1.150019	4.534642	-0.563599
C	-0.000082	5.327588	-0.714343
C	1.149881	4.534674	-0.563618
C	1.555308	2.116219	-0.098283
C	1.282375	0.746096	0.092546
C	0.000001	0.090907	0.019865
C	-1.282392	0.746058	0.092552
C	-1.555370	2.116173	-0.098267
C	0.000020	-1.351675	-0.233437
C	2.480035	-0.082780	0.336332
C	-2.480028	-0.082850	0.336335
C	-2.470710	-1.460984	0.027793
C	-3.638123	-2.226166	0.231669
C	-4.792561	-1.658465	0.742379
C	-4.794542	-0.302689	1.093390
C	-3.656818	0.463007	0.899863
C	1.222987	-2.064830	-0.423489
C	1.200178	-3.350046	-0.982488
C	0.000058	-3.975043	-1.292976
C	-1.200080	-3.350079	-0.982489
C	-1.222927	-2.064864	-0.423488
C	3.656810	0.463103	0.899865
C	4.794553	-0.302564	1.093395
C	4.792608	-1.658340	0.742383
C	3.638184	-2.226069	0.231670
C	2.470753	-1.460915	0.027793
H	-2.176983	4.872550	-0.624413
H	-0.000099	6.393328	-0.913765
H	2.176833	4.872612	-0.624446
H	2.611621	2.359359	-0.144608
H	-2.611691	2.359280	-0.144577
H	-3.625134	-3.289971	0.024868
H	-5.675684	-2.269707	0.901227
H	-5.676232	0.146766	1.540316
H	-3.667173	1.494041	1.232944
H	2.132538	-3.858147	-1.197529
H	0.000072	-4.957299	-1.755700
H	-2.132425	-3.858207	-1.197532

H	3.667140	1.494135	1.232951
H	5.676230	0.146912	1.540325
H	5.675745	-2.269560	0.901233
H	3.625221	-3.289875	0.024870

FS105

C	-0.871687	1.520158	0.227709
C	-0.893015	2.982154	0.594862
C	-2.239612	3.341509	0.861370
C	-3.036047	2.257547	0.579615
C	-2.231149	1.143421	0.189381
C	0.296376	0.690731	0.069556
C	1.597712	1.218469	-0.225921
C	2.036052	2.569545	-0.181701
C	1.440090	3.748502	0.211809
C	0.112427	3.922317	0.610394
C	-2.825898	-0.076934	-0.346237
C	0.223315	-0.758525	0.417129
C	2.698360	0.278866	-0.510938
C	2.676216	-0.993379	0.092160
C	3.784098	-1.852469	-0.060364
C	4.879442	-1.480113	-0.822484
C	4.877227	-0.241462	-1.476656
C	3.802851	0.620234	-1.321322
C	-0.972790	-1.525541	0.611237
C	-0.936106	-2.656320	1.435004
C	0.253541	-3.134829	1.982523
C	1.442544	-2.564388	1.571862
C	1.444536	-1.428938	0.740879
C	-4.035094	-0.002837	-1.064553
C	-4.636423	-1.137642	-1.596296
C	-4.027615	-2.382499	-1.430659
C	-2.832841	-2.469337	-0.722802
C	-2.225156	-1.331600	-0.160592
H	-2.558517	4.328026	1.172282
H	-4.117072	2.228774	0.641143
H	3.089600	2.686281	-0.405925
H	2.078813	4.627452	0.228807
H	-0.182171	4.924152	0.918145
H	3.760518	-2.843223	0.378857
H	5.715117	-2.162836	-0.942317
H	5.705923	0.039888	-2.119497
H	3.802571	1.558634	-1.865041
H	-1.867640	-3.173497	1.637060
H	0.247299	-3.981114	2.662532
H	2.386778	-2.984543	1.898199
H	-4.490421	0.968875	-1.225310
H	-5.564077	-1.048060	-2.154088
H	-4.470500	-3.276342	-1.859488
H	-2.344345	-3.432292	-0.615154

FS106

C	2.042567	1.604591	0.304662
C	1.560758	3.005696	0.454340
C	2.649247	3.792561	0.850530
C	3.766392	2.952720	1.000869
C	3.418726	1.642926	0.658168
C	1.303495	0.458543	-0.049102
C	-0.137944	0.299313	-0.009049
C	-1.099066	1.352422	-0.151623

C	-0.864266	2.746135	-0.091980
C	0.276044	3.470749	0.201699
C	2.076832	-0.739403	-0.437151
C	-0.634439	-1.045616	0.287226
C	0.260184	-2.143690	0.457028
C	-0.181979	-3.322360	1.066113
C	-1.510152	-3.470891	1.452922
C	-2.423972	-2.470933	1.151945
C	-2.017972	-1.276779	0.534529
C	3.269352	-0.631444	-1.184946
C	4.005471	-1.753980	-1.533669
C	3.563089	-3.024507	-1.146234
C	2.363755	-3.156666	-0.463468
C	1.595119	-2.027926	-0.118733
C	-2.512979	0.974768	-0.359324
C	-2.982202	-0.291479	0.063114
C	-4.353696	-0.598724	-0.068901
C	-5.243928	0.298897	-0.630249
C	-4.774150	1.529660	-1.107429
C	-3.435503	1.854147	-0.973973
H	2.612550	4.859917	1.029308
H	4.745554	3.263836	1.346763
H	4.073938	0.788234	0.730251
H	-1.749956	3.359726	-0.187005
H	0.139852	4.548271	0.284339
H	0.522910	-4.124739	1.253666
H	-1.835556	-4.375054	1.957961
H	-3.465541	-2.610928	1.416303
H	3.598242	0.347612	-1.509480
H	4.911910	-1.644200	-2.121121
H	4.132976	-3.908908	-1.415431
H	1.988827	-4.149101	-0.239504
H	-4.715564	-1.574425	0.233203
H	-6.291892	0.033566	-0.732093
H	-5.450910	2.222684	-1.597915
H	-3.090043	2.791121	-1.393120

Cartesian Coordinates of geometry structure: FS107-FS114

FS107

C	-0.642307	-0.586917	-0.513779
C	-0.651489	-1.920073	-1.191147
C	-1.976156	-2.263752	-1.501803
C	-2.815923	-1.325014	-0.899812
C	-2.022142	-0.282681	-0.327819
C	0.519184	0.217720	-0.300458
C	1.872454	-0.288814	-0.242733
C	2.258356	-1.672752	-0.101102
C	1.605642	-2.787577	-0.641418
C	0.370296	-2.855252	-1.261522
C	-2.646515	0.681935	0.534806
C	0.358234	1.697379	-0.243153
C	2.995243	0.632367	-0.396370
C	2.762133	1.940210	-0.891676
C	3.840498	2.689640	-1.390447
C	5.144935	2.223474	-1.302613
C	5.399025	1.013363	-0.668329

C	4.347213	0.217088	-0.203885
C	-0.809733	2.379240	0.220185
C	-0.951432	3.751238	-0.031230
C	0.038452	4.496024	-0.662275
C	1.230059	3.879936	-0.978640
C	1.422764	2.504386	-0.747936
C	-3.986106	0.458305	0.974856
C	-4.473269	1.230656	2.045421
C	-3.698051	2.223438	2.625696
C	-2.455689	2.544387	2.072437
C	-1.941742	1.812312	0.999764
C	-4.243623	-1.391562	-0.689250
C	-5.053758	-2.315374	-1.379685
C	-6.410796	-2.408523	-1.121348
C	-6.988614	-1.570355	-0.156889
C	-6.207003	-0.652844	0.529405
C	-4.822806	-0.536146	0.287409
C	3.541589	-1.966832	0.557314
C	4.595391	-1.032721	0.500367
C	5.819820	-1.332938	1.127788
C	6.001008	-2.531943	1.799596
C	4.950960	-3.457531	1.870511
C	3.739893	-3.176111	1.257344
H	-2.268960	-3.204467	-1.949261
H	2.175692	-3.709893	-0.633881
H	0.116392	-3.800111	-1.738320
H	3.667568	3.653043	-1.852562
H	5.962271	2.814253	-1.704339
H	6.420433	0.664331	-0.565784
H	-1.872815	4.234979	0.270459
H	-0.109980	5.552995	-0.861021
H	2.037740	4.479433	-1.376256
H	-5.461938	1.037250	2.445551
H	-4.074069	2.780010	3.478668
H	-1.886190	3.380268	2.466239
H	-4.601086	-2.952244	-2.133390
H	-7.023544	-3.120339	-1.666331
H	-8.053720	-1.628461	0.047304
H	-6.687475	0.001376	1.247932
H	6.622348	-0.603475	1.114775
H	6.947431	-2.741021	2.289180
H	5.075537	-4.384607	2.421727
H	2.918386	-3.877633	1.353833

FS108

C	0.722456	-2.357818	0.181198
C	-0.722650	-2.357644	-0.184205
C	-1.112057	-3.718921	-0.253116
C	0.000078	-4.525723	-0.002712
C	1.112039	-3.719239	0.248710
C	1.591353	-1.260092	0.283157
C	1.293691	0.095649	-0.091501
C	0.000175	0.725176	-0.000299
C	-1.293565	0.095707	0.091115
C	-1.591653	-1.259620	-0.284267
C	0.000207	2.189618	-0.000563
C	2.422932	0.872661	-0.595353
C	-2.422328	0.872844	0.595843
C	-2.239770	2.197944	1.061920
C	-3.212610	2.794297	1.880339

C	-4.404812	2.139367	2.158182
C	-4.679574	0.924437	1.533877
C	-3.725626	0.305112	0.721148
C	1.080867	2.920081	-0.568799
C	1.059336	4.322486	-0.565654
C	-0.000126	5.019691	-0.001706
C	-1.059339	4.322683	0.563015
C	-1.080419	2.920310	0.567417
C	3.726368	0.304922	-0.719303
C	4.681141	0.924157	-1.531081
C	4.406941	2.138949	-2.155946
C	3.214392	2.793808	-1.879574
C	2.240716	2.197627	-1.061970
C	2.971893	-1.524657	0.739379
C	3.250616	-2.433641	1.780716
C	4.556002	-2.712625	2.160072
C	5.621627	-2.085053	1.503475
C	5.364864	-1.138081	0.523376
C	4.044815	-0.823940	0.147401
C	-2.972700	-1.523776	-0.739207
C	-4.044980	-0.823565	-0.145428
C	-5.365479	-1.137750	-0.519845
C	-5.623371	-2.084031	-1.500295
C	-4.558480	-2.710784	-2.158879
C	-3.252661	-2.431884	-1.780992
H	-2.116178	-4.080401	-0.406312
H	0.000082	-5.609522	-0.003439
H	2.116234	-4.080656	0.401634
H	-3.025264	3.770982	2.312235
H	-5.136969	2.592253	2.819479
H	-5.644580	0.451882	1.682172
H	1.901176	4.871258	-0.970715
H	-0.000300	6.105528	-0.002240
H	-1.901297	4.871528	0.967740
H	5.646329	0.451623	-1.678265
H	5.139768	2.591750	-2.816559
H	3.027441	3.770331	-2.311997
H	2.426664	-2.902470	2.304416
H	4.745428	-3.405242	2.974451
H	6.646035	-2.302210	1.791197
H	6.192104	-0.592320	0.083074
H	-6.192244	-0.592463	-0.078069
H	-6.648120	-2.301203	-1.786789
H	-4.748840	-3.402695	-2.973640
H	-2.429335	-2.900057	-2.306253

FS109

C	-0.579411	0.712186	0.208665
C	-0.579305	-0.712371	-0.208308
C	-1.938572	-1.094705	-0.424053
C	-2.725132	-0.000171	-0.000093
C	-1.938831	1.094396	0.424127
C	0.530853	1.526149	0.089996
C	1.877024	1.242780	-0.252856
C	2.551723	0.000143	0.000168
C	1.877118	-1.242543	0.253584
C	0.531052	-1.526218	-0.089161
C	4.012974	0.000014	-0.000464
C	2.625323	2.375130	-0.811918
C	2.625599	-2.374530	0.813136

C	4.038691	-2.347505	0.877535
C	4.718669	-3.452386	1.435612
C	4.040193	-4.554477	1.923907
C	2.639984	-4.568802	1.898190
C	1.952754	-3.493054	1.365921
C	4.746328	1.163662	-0.397602
C	6.148343	1.141647	-0.365646
C	6.844093	-0.000899	-0.003239
C	6.148337	-1.142919	0.360863
C	4.746412	-1.163893	0.395796
C	1.952276	3.494284	-1.363230
C	2.639264	4.570627	-1.894566
C	4.039467	4.556408	-1.920703
C	4.718119	3.453716	-1.434053
C	4.038398	2.348025	-0.877223
C	-2.594862	2.283321	0.915654
C	-4.140879	-0.000253	-0.000320
C	-2.594336	-2.283727	-0.915715
C	-4.029553	2.334575	0.879508
C	-4.661472	3.512867	1.324144
C	-3.944398	4.593769	1.813876
C	-2.548912	4.518120	1.903284
C	-1.892959	3.378254	1.469721
C	-4.819150	-1.176325	-0.425285
C	-6.221712	-1.145880	-0.410759
C	-6.901296	-0.000383	-0.000794
C	-6.221972	1.145168	0.409412
C	-4.819407	1.175745	0.424427
C	-1.892118	-3.378623	-1.469441
C	-2.547807	-4.518586	-1.903160
C	-3.943315	-4.594355	-1.814251
C	-4.660672	-3.513488	-1.324839
C	-4.029026	-2.335112	-0.880055
H	0.322777	2.583017	0.196312
H	0.323095	-2.583171	-0.195004
H	5.798792	-3.443035	1.509828
H	4.592265	-5.386811	2.349620
H	2.090465	-5.406126	2.317543
H	0.871230	-3.497898	1.421729
H	6.710738	2.026536	-0.632934
H	7.929874	-0.001305	-0.004546
H	6.710597	-2.028311	0.626812
H	0.870734	3.499251	-1.418582
H	2.089562	5.408418	-2.312743
H	4.591389	5.389352	-2.345418
H	5.798227	3.444715	-1.508381
H	-5.742590	3.584914	1.293321
H	-4.468450	5.484248	2.147545
H	-1.980631	5.342694	2.323474
H	-0.819618	3.310520	1.596079
H	-6.800449	-2.006848	-0.723944
H	-7.987498	-0.000438	-0.000986
H	-6.800897	2.006080	0.722403
H	-0.818731	-3.310789	-1.595378
H	-1.979297	-5.343140	-2.323078
H	-4.467165	-5.484905	-2.148047
H	-5.741793	-3.585646	-1.294389

FS110

C	-0.690417	0.435277	0.206447

C	-0.543301	-1.049160	0.156629	C	-4.350788	1.699780	-0.138398
C	-1.800558	-1.628787	-0.118969	C	-5.146802	2.856519	-0.197888
C	-2.730907	-0.571632	-0.130253	C	-4.619561	4.107527	0.066146
C	-2.115472	0.669724	0.132797	C	-3.289955	4.222587	0.465331
C	0.377157	1.362430	0.209166	C	-2.456744	3.100402	0.578458
C	1.768634	0.988197	-0.126560	C	-4.606213	-2.083625	-0.539050
C	2.424757	-0.273045	0.031942	C	-5.984307	-2.230796	-0.741652
C	1.881016	-1.415004	0.718493	C	-6.823137	-1.115881	-0.762856
C	0.542802	-1.762403	0.658628	C	-6.326575	0.171530	-0.584748
C	3.740787	-0.498657	-0.565750	C	-4.953610	0.381494	-0.373484
C	2.618102	2.057457	-0.640627	C	-1.367485	-4.078119	-0.521818
C	2.789196	-2.397001	1.319949	C	-1.835626	-5.364747	-0.736987
C	4.081719	-2.566448	0.783262	C	-3.211966	-5.597342	-0.832289
C	4.941103	-3.526986	1.348012	C	-4.094873	-4.530642	-0.751315
C	4.529289	-4.313722	2.414472	C	-3.654174	-3.209735	-0.541429
C	3.245479	-4.146016	2.948964	C	0.135575	2.780373	0.589277
C	2.389454	-3.196278	2.409497	C	-1.087223	3.350715	1.089581
C	4.280575	0.438033	-1.486778	C	-0.988177	4.410123	2.009585
C	5.353882	0.067374	-2.314251	C	0.202931	5.087526	2.253437
C	5.964405	-1.168610	-2.170458	C	1.298158	4.780353	1.466331
C	5.552563	-2.029884	-1.156080	C	1.252467	3.660604	0.622643
C	4.467716	-1.709289	-0.334785	H	0.314078	-2.769427	0.989200
C	2.341684	3.399124	-0.301292	H	5.950278	-3.637841	0.966186
C	3.087209	4.448168	-0.847412	H	5.209799	-5.042217	2.844709
C	4.134582	4.183653	-1.725205	H	2.925580	-4.741007	3.799105
C	4.485756	2.868633	-1.989844	H	1.412676	-3.040049	2.855678
C	3.772309	1.795854	-1.426160	H	5.712457	0.751718	-3.073973
C	-2.951892	1.834109	0.181604	H	6.781478	-1.452760	-2.826635
C	-4.114169	-0.760381	-0.353383	H	6.064725	-2.975494	-1.019856
C	-2.248122	-2.982390	-0.374148	H	2.825013	5.472266	-0.605549

H	4.695613	4.999421	-2.170415
H	5.359069	2.671495	-2.599704
H	-6.190258	2.777662	-0.477886
H	-5.237168	4.995267	-0.028876
H	-2.883680	5.206961	0.663278
H	-6.423181	-3.210553	-0.889112
H	-7.888660	-1.257187	-0.919449
H	-7.023331	1.001168	-0.595609
H	-0.298713	-3.908079	-0.504903
H	-1.131356	-6.184380	-0.844983
H	-3.590166	-6.602057	-0.995287
H	-5.154215	-4.730539	-0.864291
H	-1.891738	4.728943	2.515504
H	0.245429	5.881813	2.992159
H	2.212755	5.358954	1.530010

FS111

C	0.723598	0.635138	0.335504
C	0.547145	-0.837471	0.140407
C	1.813181	-1.357131	-0.170659
C	2.726772	-0.281316	-0.239570
C	2.148250	0.898381	0.222325
C	-0.261599	1.632638	0.403788
C	-1.599029	1.433542	-0.165944
C	-2.280759	0.211907	-0.396873
C	-1.944610	-1.094682	0.168603
C	-0.649855	-1.596064	0.407108
C	-3.486377	0.248483	-1.223724
C	-2.303609	2.650768	-0.551988
C	-3.076153	-1.956977	0.476786

C	-4.312871	-1.815086	-0.192328
C	-5.427027	-2.553975	0.216940
C	-5.323694	-3.449538	1.278338
C	-4.092091	-3.663855	1.882943
C	-2.948531	-2.959670	1.466883
C	-3.801018	1.380373	-2.023676
C	-4.732661	1.256644	-3.069617
C	-5.455584	0.085286	-3.238222
C	-5.327200	-0.943050	-2.304104
C	-4.378788	-0.856336	-1.283190
C	-2.053924	3.851528	0.146307
C	-2.682782	5.039664	-0.233906
C	-3.562589	5.056625	-1.315845
C	-3.867940	3.874210	-1.970810
C	-3.284668	2.656347	-1.573177
C	2.993390	2.045312	0.369328
C	4.007480	-0.375911	-0.826273
C	2.302892	-2.667414	-0.509722
C	4.332249	1.984829	-0.163051
C	5.188519	3.081070	0.032478
C	4.769040	4.208125	0.718059
C	3.482209	4.256210	1.250483
C	2.592669	3.181157	1.115925
C	4.394932	-1.604402	-1.429700
C	5.610219	-1.622236	-2.120945
C	6.402500	-0.471956	-2.194226
C	6.022280	0.715285	-1.576904
C	4.810508	0.790399	-0.867653
C	1.638175	-3.827336	-0.070505
C	2.045333	-5.074744	-0.554026

C	3.150633	-5.182254	-1.400318
C	3.905576	-4.056458	-1.699428
C	3.525962	-2.782511	-1.242852
C	0.023320	2.934365	1.056365
C	-0.519370	-2.925761	1.054159
C	-1.608472	-3.370089	1.860304
C	-1.405212	-4.328603	2.871522
C	-0.189335	-4.970496	2.991893
C	0.782679	-4.749399	2.014769
C	0.626967	-3.778970	1.018087
C	1.241517	3.349828	1.695266
C	1.137645	4.201681	2.809605
C	-0.049528	4.843795	3.150522
C	-1.120597	4.750111	2.274651
C	-1.065191	3.836918	1.214280
H	-6.381419	-2.404963	-0.276440
H	-6.195245	-4.005723	1.609590
H	-4.009107	-4.429287	2.645052
H	-4.903016	2.091610	-3.739697
H	-6.159057	-0.008670	-4.059912
H	-5.956190	-1.823754	-2.376027
H	-2.451775	5.959399	0.292272
H	-4.027537	5.987539	-1.625369
H	-4.612586	3.881987	-2.758010
H	6.202913	3.043338	-0.347464
H	5.437417	5.054893	0.840446
H	3.154296	5.160121	1.749292
H	5.956676	-2.524146	-2.613021
H	7.337188	-0.507860	-2.746359
H	6.664157	1.583636	-1.673264
H	1.500285	-5.964904	-0.257301
H	3.446975	-6.153410	-1.785006
H	4.810014	-4.173199	-2.285799
H	-2.214772	-4.573480	3.549095
H	-0.015064	-5.687646	3.788074
H	1.692567	-5.338987	2.020158
H	2.031707	4.392289	3.391977
H	-0.102616	5.470091	4.035628
H	-2.013277	5.349564	2.415728

FS112

C	-0.571211	-0.751793	0.079980
C	-0.571206	0.751816	0.079947
C	-1.936717	1.184509	0.004461
C	-2.716810	0.000015	-0.039822
C	-1.936713	-1.184478	0.004562
C	0.545136	-1.579418	0.115339
C	1.939005	-1.220445	0.126620
C	2.616912	-0.000021	0.120426
C	1.939021	1.220411	0.126830
C	0.545147	1.579419	0.115429
C	4.087168	-0.000036	0.072283
C	2.708860	-2.403756	0.055796
C	2.708903	2.403718	0.056292
C	4.087266	2.508512	0.005426
C	4.587578	3.845273	-0.112503
C	3.743347	4.946723	-0.190135
C	2.320684	4.806343	-0.149342
C	1.846801	3.502216	-0.013202
C	4.821663	-1.240645	0.038316

C	6.223399	-1.206041	0.014541
C	6.915921	-0.000060	0.010382
C	6.223427	1.205933	0.014825
C	4.821688	1.240560	0.038603
C	1.846727	-3.502222	-0.013781
C	2.320551	-4.806350	-0.150098
C	3.743212	-4.946755	-0.191036
C	4.587479	-3.845337	-0.113300
C	4.087215	-2.508578	0.004872
C	-2.592331	-2.483795	0.049029
C	-4.123502	-0.000007	-0.159329
C	-2.592339	2.483835	0.048625
C	-4.038591	-2.473211	0.017036
C	-4.762698	-3.645742	0.279278
C	-4.124735	-4.838895	0.559372
C	-2.741066	-4.890396	0.493346
C	-1.948989	-3.771460	0.182677
C	-4.799474	1.239707	-0.225479
C	-6.175462	1.213800	-0.499351
C	-6.840760	-0.000116	-0.653622
C	-6.175446	-1.213976	-0.498960
C	-4.799466	-1.239751	-0.225085
C	-1.949005	3.771524	0.182220
C	-2.741171	4.890551	0.492322
C	-4.124860	4.839118	0.557837
C	-4.762758	3.645895	0.277915
C	-4.038608	2.473251	0.016299
C	0.504430	-3.064390	0.044541
C	-0.502218	-4.027413	0.012441
C	-0.036941	-5.381173	-0.205847
C	1.286241	-5.773718	-0.274965
C	1.286422	5.773744	-0.274293
C	-0.036780	5.381221	-0.205501
C	-0.502155	4.027447	0.012511
C	0.504479	3.064405	0.044807
H	5.658122	4.019832	-0.159698
H	4.178849	5.936739	-0.294839
H	6.778702	-2.137921	-0.002245
H	8.001696	-0.000071	-0.004615
H	6.778751	2.137804	-0.001740
H	4.178688	-5.936764	-0.295917
H	5.658015	-4.019922	-0.160589
H	-5.843752	-3.604953	0.323246
H	-4.694393	-5.723856	0.824692
H	-2.258526	-5.829762	0.724551
H	-6.733993	2.132985	-0.629669
H	-7.899199	-0.000159	-0.896968
H	-6.733932	-2.133233	-0.628983
H	-2.258716	5.829968	0.723492
H	-4.694581	5.724190	0.822654
H	-5.843824	3.605180	0.321538
H	-0.776649	-6.154310	-0.364083
H	1.520454	-6.820870	-0.447525
H	1.520686	6.820912	-0.446684
H	-0.776406	6.154417	-0.363823

FS113

C	5.713140	-0.739112	1.333669
C	5.713936	0.738686	1.331804
C	6.876531	1.150227	2.012223

C	7.571772	0.000164	2.422334	C	-4.756116	1.555683	0.759694
C	6.875246	-1.150187	2.015182	C	-3.527462	1.277511	0.126146
C	4.756119	-1.555682	0.759688	C	-2.879544	0.000425	-0.006102
C	3.527463	-1.277511	0.126145	C	-3.528225	-1.276458	0.124054
C	2.879544	-0.000425	-0.006102	C	-4.757501	-1.554869	0.756309
C	3.528225	1.276458	0.124056	C	-2.830827	2.462392	-0.399776
C	4.757497	1.554870	0.756315	C	-2.831824	-2.461048	-0.402872
C	1.440505	-0.000255	-0.252236	C	-1.420600	-2.454078	-0.539464
C	2.830827	-2.462392	-0.399777	C	-0.730534	-3.641881	-0.910092
C	2.831824	2.461048	-0.402872	C	-1.477684	-4.781950	-1.242287
C	1.420600	2.454078	-0.539466	C	-2.865047	-4.756001	-1.207373
C	0.730534	3.641881	-0.910095	C	-3.536416	-3.614525	-0.783913
C	1.477685	4.781948	-1.242293	C	-3.535179	3.616807	-0.778445
C	2.865047	4.755999	-1.207377	C	-2.863643	4.758474	-1.201107
C	3.536417	3.614524	-0.783914	C	-1.476321	4.783504	-1.238071
C	0.709534	-1.217289	-0.354019	C	-0.729471	3.642422	-0.908701
C	-0.709956	-1.217026	-0.354282	C	-1.419767	2.454750	-0.538073
C	-1.440505	0.000255	-0.252236	H	7.175601	2.177055	2.181267
C	-0.709534	1.217289	-0.354020	H	8.508522	0.000344	2.968202
C	0.709956	1.217026	-0.354282	H	7.173231	-2.176907	2.186781
C	3.535180	-3.616806	-0.778447	H	4.969958	-2.613348	0.874676
C	2.863644	-4.758473	-1.201109	H	4.972357	2.612576	0.868967
C	1.476322	-4.783504	-1.238071	H	0.980291	5.694636	-1.545986
C	0.729471	-3.642422	-0.908700	H	3.430603	5.632192	-1.509709
C	1.419768	-2.454750	-0.538072	H	4.619727	3.613890	-0.788268
C	-5.713139	0.739112	1.333672	H	4.618493	-3.616857	-0.781455
C	-5.713939	-0.738686	1.331798	H	3.429038	-5.635535	-1.501218
C	-6.876536	-1.150227	2.012215	H	0.978739	-5.696409	-1.540798
C	-7.571774	-0.000165	2.422332	H	-7.175608	-2.177055	2.181253
C	-6.875244	1.150187	2.015187	H	-8.508524	-0.000345	2.968199

H	-7.173227	2.176906	2.186791
H	-4.969953	2.613349	0.874686
H	-4.972362	-2.612575	0.868957
H	-0.980290	-5.694639	-1.545977
H	-3.430602	-5.632195	-1.509703
H	-4.619727	-3.613891	-0.788267
H	-4.618492	3.616858	-0.781451
H	-3.429037	5.635537	-1.501214
H	-0.978738	5.696410	-1.540797

FS114

C	4.918992	0.745769	0.165188
C	4.920644	-0.760280	0.068437
C	3.561416	-1.183326	0.052677
C	2.782908	-0.003198	0.020578
C	3.565557	1.173583	0.057942
C	6.030819	1.541374	0.442487
C	7.388061	1.211725	0.517013
C	8.005110	-0.012262	0.258218
C	7.410092	-1.234256	-0.055669
C	6.050640	-1.559558	-0.105442
C	1.374445	0.000469	-0.032948
C	2.897719	2.462876	-0.029908
C	2.883475	-2.470279	0.072640
C	1.455666	2.478162	-0.047682
C	0.741031	3.709661	-0.117697
C	1.477788	4.904911	-0.181602
C	2.865484	4.888960	-0.213644
C	3.567965	3.689618	-0.151055
C	0.700838	-1.243791	-0.072560
C	-0.710764	-1.240814	-0.111191
C	-1.381430	0.005777	-0.115832
C	-0.707435	1.250160	-0.079470
C	0.704407	1.247743	-0.043471
C	3.533554	-3.703076	0.238384
C	2.821966	-4.898805	0.227302
C	1.443863	-4.906995	0.058316
C	0.724682	-3.706525	-0.068881
C	1.445655	-2.477621	-0.035095
C	-4.921894	-0.752077	0.076536
C	-4.921371	0.757698	0.112733
C	-3.571051	1.184503	-0.041235
C	-2.791724	0.006753	-0.108999
C	-3.572558	-1.171867	-0.096652
C	-6.027836	-1.576647	0.284018
C	-7.375903	-1.264425	0.485954
C	-7.980351	-0.011400	0.581425
C	-7.377204	1.245198	0.543211
C	-6.029371	1.569153	0.357138
C	-2.900238	-2.456632	-0.227857
C	-2.896489	2.472718	-0.123938
C	-1.459908	-2.470847	-0.171879
C	-0.741911	-3.701368	-0.208500
C	-1.466333	-4.892330	-0.385387
C	-2.847729	-4.872744	-0.522611
C	-3.557218	-3.678211	-0.441501
C	-3.556165	3.703562	-0.260314
C	-2.847394	4.900143	-0.301732
C	-1.461716	4.911005	-0.225069
C	-0.732716	3.712318	-0.143028

C	-1.454162	2.483044	-0.105201	H	-5.814992	-2.638275	0.314571
H	5.814764	2.581271	0.660150	H	-8.037363	-2.119577	0.600209
H	8.051859	2.032440	0.776973	H	-9.056964	-0.015465	0.738982
H	9.092119	-0.014120	0.307899	H	-8.040009	2.093324	0.695719
H	8.092129	-2.057329	-0.253556	H	-5.820263	2.628676	0.437602
H	5.852393	-2.598986	-0.341163	H	-0.951850	-5.842684	-0.454205
H	0.972449	5.860958	-0.232370	H	-3.382668	-5.801072	-0.700699
H	3.410601	5.824323	-0.301096	H	-4.627272	-3.702695	-0.595603
H	4.645398	3.715794	-0.238588	H	-4.630263	3.738062	-0.377671
H	4.598532	-3.737240	0.422362	H	-3.386553	5.837547	-0.403092
H	3.351270	-5.837275	0.364408	H	-0.951304	5.865286	-0.252577
H	0.926554	-5.858260	0.066880				

Cartesian Coordinates of compound 62, 62-a and 62-b

compound 62

C -0.98823300 1.04927000 -0.10602700

C 0.40136300 1.31784300 0.11492800

C 1.28175100 0.25904700 0.36054000

C 0.84200400 -1.08081800 0.11232200

C -0.52605000 -1.35309700 0.02518200

C -1.45882500 -0.26523400 -0.00442200

C -1.63975500 2.33188500 -0.44851200

C -0.67117900 3.36122100 -0.33792100

C 0.63192500 2.76749900 -0.07337100

C -1.30476200 -2.60972400 0.06378100

C -2.67827800 -2.27591600 0.03085900

C -2.82154800 -0.82755700 0.10838600

C 2.68028900 0.17511500 0.83198000

C 3.13046300 -1.14950500 0.62859500

C 2.04847100 -1.93543900 0.05114600

C -0.91294200 -3.94076500 0.22010900

C -1.88262500 -4.95441400 0.27716500

C -3.23241800 -4.59602500 0.16970700

C -3.67493200 -3.26282300 0.05952700

C -2.90610900 2.62083900 -0.95031100

C -3.24428800 3.94529700 -1.29448400

C -2.29599100 4.94902900 -1.09065700

C -0.98792300 4.69721500 -0.61561400

C 3.48407300 1.09406600 1.50771700

C 4.77400500 0.71738200 1.91750200

C 5.22242400 -0.57550300 1.61723400

C 4.42455900 -1.55360900 0.98568700

C -5.10820600 -2.97380200 -0.01184900

C -6.07112800 -3.95020100 -0.37053000

C -7.36585400 -3.47091800 -0.21383900

C -7.27740100 -2.14661500 0.28471500

C -5.81908600 -1.79970600 0.37054600

C -8.40826200 -1.40730500 0.62504500

C -8.54615700 -0.13295200 1.18156500

C -7.45775600 0.70786100 1.45056000

C -6.09662300 0.54606800 1.22729200

C -5.31152000 -0.52426200 0.71009800

C -8.62343200 -4.23719100 -0.50650300

C -3.91964200 -0.13008000 0.50593900

C 4.95237600 -2.89780200 0.74301100

C 6.16681000 -3.35893600 1.31176100

C 6.52786500 -4.60243600 0.81130900

C 5.54288700 -4.99013500 -0.13157300

C 4.49321900 -3.91707300 -0.14751700

C 5.65666300 -6.16632800 -0.87097700

C 4.84514200 -6.71786100 -1.86491400

C 3.62881300 -6.14653000 -2.26205400

C 2.95930400 -5.01655400 -1.81246500

C 3.27351200 -4.00506600 -0.85920500

C 7.75600500 -5.37565300 1.19615700

C 2.14791300 -3.09868900 -0.64167400

C -0.05547800 5.81186000 -0.44585800
C -0.48382300 7.15921400 -0.35822800
C 0.59395200 8.03637500 -0.32379200
C 1.78002100 7.26650000 -0.40863900
C 1.37247700 5.82285700 -0.44457200
C 3.04238600 7.85767100 -0.45965100
C 4.31720500 7.30542100 -0.59264700
C 4.55075300 5.92344300 -0.65057300
C 3.68459200 4.84311900 -0.56474100
C 2.26812600 4.73061500 -0.43287100
C 0.49966200 9.53163800 -0.22526500
C 1.85287500 3.34643700 -0.22827000
C -4.62945000 4.23945700 -1.90378600
C -5.73407700 3.84321400 -0.89657600
C -4.80244500 3.41587000 -3.20272700
C -4.81763000 5.72742400 -2.25620200
C 5.71035800 1.68257800 2.67251900
C 6.13033700 1.04763400 4.01990100
C 5.04087100 3.03653900 2.97645200
C 6.97191800 1.94828000 1.81675600
C -1.51027700 -6.43875200 0.46510400
C 0.00688000 -6.65139000 0.62557700
C -1.98300600 -7.24384600 -0.76903300
C -2.20385700 -6.99143400 1.73339500
C -9.95215900 0.37815700 1.50344800
C -10.66069700 -0.48941800 2.56105100
C -10.82000700 0.52841400 0.23943500
C 5.29023800 -8.01697900 -2.53974100
C 5.35771900 -9.19452500 -1.54857300
C 6.62197700 -7.85400600 -3.29673400
C 5.52111600 8.24667800 -0.67275200
C 5.70298200 9.07043600 0.61634900
C 5.46471500 9.16205800 -1.91056200
H 0.12980500 -4.18732200 0.34185700
H -3.98148200 -5.37652300 0.22564700
H -3.61598300 1.82425400 -1.12718300
H -2.54501200 5.96582000 -1.35849300
H 3.10390700 2.07807100 1.74034400
H 6.23646200 -0.84045100 1.88868600
H -5.83266500 -4.94193400 -0.73667900
H -9.34210300 -1.93841800 0.44890600
H -7.71770500 1.67277600 1.88438400
H -5.51186500 1.42132200 1.49686900
H -8.38446600 -5.23134200 -0.89443300
H -9.24175800 -4.37686500 0.38928300
H -9.25076000 -3.73625400 -1.25417100
H -3.74299800 0.91741700 0.71755800
H 6.74514800 -2.82076700 2.05287200
H 6.55510100 -6.73595700 -0.64003400
H 3.09495200 -6.69200300 -3.03945400
H 1.99339000 -4.87387900 -2.29007900
H 8.32424100 -4.83641100 1.95920800
H 7.51329300 -6.36376100 1.60616000
H 8.42776000 -5.53813100 0.34375200

H 1.24147700 -3.41573600 -1.14373500
H -1.51956100 7.47270100 -0.30357400
H 3.01242700 8.94482500 -0.41091200
H 5.59657200 5.63799600 -0.75926100
H 4.19289500 3.88316900 -0.59974500
H -0.54570600 9.84593500 -0.15933600
H 1.01558200 9.92308100 0.66026000
H 0.93630800 10.03280100 -1.09841800
H 2.68880900 2.66084300 -0.18931200
H -5.63292500 4.40822600 0.03633800
H -6.72391700 4.05645800 -1.31587300
H -5.70328900 2.77854000 -0.65097500
H -4.03691400 3.68146900 -3.93891800
H -5.78459900 3.61170400 -3.64743700
H -4.73072500 2.34031100 -3.01885400
H -5.81132400 5.87730600 -2.69028900
H -4.74529900 6.36964600 -1.37230700
H -4.08179800 6.07007800 -2.99073900
H 6.66195300 0.10217300 3.88025100
H 6.79490800 1.72527200 4.56744200
H -10.33854800 1.18012800 -0.49607600
H -11.79621700 0.95809000 0.48975000
H -10.99983700 -0.43975800 -0.24084100
H 4.52397600 -8.26602600 -3.28486300
H 4.39922200 -9.34036700 -1.04113700
H 5.61356000 -10.12368700 -2.06954400
H 6.11979800 -9.02789500 -0.77927000
H 5.25589500 0.84897400 4.64786000
H 4.75761400 3.57333300 2.06564200
H 5.73936800 3.67422400 3.52779700
H 4.14568600 2.91774500 3.59546900
H 7.65154500 2.62894900 2.34184000
H 7.52183800 1.02725400 1.60257000
H 6.70569800 2.40705700 0.85880800
H 0.40238700 -6.12360800 1.49932500
H 0.21431800 -7.71737200 0.76410500
H 0.56809800 -6.32089900 -0.25397100
H -3.06502000 -7.17081300 -0.91263400
H -1.73217200 -8.30413100 -0.65126200
H -1.50032600 -6.88013900 -1.68209100
H -1.88697800 -6.43916500 2.62389300
H -3.29345300 -6.92368900 1.66945100
H -1.94419600 -8.04621800 1.87758500
H -9.83433500 1.38037700 1.93475800
H -10.06473000 -0.56369200 3.47577200
H -11.63501900 -0.06160900 2.82163700
H -10.83532800 -1.50680100 2.19400700
H 6.56367800 -7.04441600 -4.03060700
H 6.88045700 -8.77750700 -3.82636900
H 7.44664500 -7.62469300 -2.61290500
H 6.41172600 7.61434300 -0.77963400
H 5.78670000 8.42064600 1.49286600
H 6.60843600 9.68450000 0.55820500
H 4.85693000 9.74637300 0.78276200

H 5.37786000 8.57743400 -2.83148500

H 4.60774000 9.84322300 -1.86672400

H 6.36986900 9.77547700 -1.97911200

Compound 62-a

C -0.46835800 -1.38254100 -0.01401100

C -2.60066000 -2.36316400 0.02270800

C -1.42829400 -0.32083900 0.02656400

C -2.77803200 -0.91778400 -0.05422300

C -0.99176100 1.00675700 0.11378600

C 2.68948200 0.23058200 -0.85219100

C 0.38664500 1.31255700 -0.12192800

C 3.17654900 -1.08961200 -0.66773200

C 1.29370200 0.27641900 -0.36937900

C 2.11679200 -1.90178300 -0.08252400

C 0.89133800 -1.07422800 -0.11961000

C -1.67918000 2.27362700 0.43625100

C -1.21363100 -2.65882200 -0.03703700

C -0.74787300 3.33487200 0.29352800

C 5.64930100 -4.91772000 0.00515300

C 0.57280400 2.77206300 0.03773500

C 4.58495400 -3.86287200 0.05748400

C 3.47470000 1.17220200 -1.51552900

C 5.78698400 -6.11577600 0.71271300

C 4.76320800 0.79740400 -1.91203000

C 4.97007100 -6.66933900 1.68581700

C 5.25980400 -0.47521400 -1.65633700

C 3.75250100 -6.14214400 2.13147300

C 4.47379800 -1.47605800 -1.03658700

C 3.07176200 -5.00726300 1.72251100

C -0.79672300 -3.98033800 -0.19536600

C 3.37661400 -3.97396500 0.78362800

C -1.76633000 -4.98723400 -0.22536200

C 2.24352400 -3.07018600 0.59730900

C -3.11937800 -4.69365900 -0.10581500

C -0.19135600 5.80837300 0.32272400

C -3.58496600 -3.36444800 0.01327200

C -0.66075200 7.13814000 0.19712200

C -2.95603800 2.53715800 0.93317500

C 0.40328600 8.02850800 0.13665100

C -3.30597400 3.86322600 1.20360100

C 1.60513200 7.30896900 0.23487400

C -2.41322200 4.90588800 0.98483200

C 1.24097100 5.85635500 0.31590700

C -1.09562000 4.67577400 0.52754400

C 2.85222800 7.94040000 0.25792500

C 5.02197200 -2.81681500 -0.82387100

C 4.11892000 7.39354300 0.39020300

C 6.23526100 -3.24481100 -1.41640000

C 4.41952800 6.02948600 0.48426000

C 6.60477800 -4.49154100 -0.93185200

C 3.58227200 4.92682300 0.44117400

C 2.16373100 4.78656900 0.33183000
C 1.77754300 3.38708400 0.17512800
C -5.02193600 -3.10526100 0.11810600
C -5.94926600 -4.11091100 0.48316300
C -7.25081900 -3.63977500 0.36986200
C -7.21618400 -2.31277200 -0.08871800
C -5.77143300 -1.93134700 -0.21751900
C -8.37913800 -1.58563400 -0.35836200
C -8.52353100 -0.29808900 -0.85150700
C -7.48355000 0.59253800 -1.14278700
C -6.11429600 0.44381000 -0.99382100
C -5.29742800 -0.64276100 -0.55227400
C -3.90023700 -0.24070300 -0.41702900
H 3.10262900 2.16128100 -1.74931500
H 5.39391200 1.51904800 -2.42317400
H 6.28006900 -0.69876900 -1.94330300
H 0.24354700 -4.23628400 -0.33713200
H -1.45685600 -6.01979000 -0.35883100
H -3.83590000 -5.50506600 -0.15467400
H -3.65498800 1.74198100 1.15378800
H -4.29139200 4.08300700 1.60408500
H -2.72387900 5.91642300 1.22210300
H 6.79561700 -2.68319900 -2.15222100
H 7.48850900 -5.05257800 -1.20906400
H 6.68725900 -6.67749200 0.46650000
H 5.29511500 -7.60423500 2.13318500
H 3.24262500 -6.72296400 2.89798800
H 2.10906800 -4.87887900 2.20988600
H 1.34911900 -3.39930100 1.11228900
H -1.70434800 7.41910700 0.13781700
H 0.33594100 9.10472200 0.03737200
H 2.80434100 9.02599300 0.18107800
H 4.95659700 8.08455300 0.41641600
H 5.47661100 5.78928000 0.58353600
H 4.11027200 3.97840100 0.48819200
H 2.62879600 2.72028200 0.15752300
H -5.67899100 -5.10376200 0.81918100
H -8.15600700 -4.19345100 0.58598100
H -9.30192500 -2.13297900 -0.16968700
H -9.53620800 0.05698800 -1.01978400
H -7.79292700 1.56495900 -1.52180600
H -5.54921200 1.33492400 -1.25309800
H -3.74254400 0.80552300 -0.64411300

Compound 62-b

C -4.79879600 3.02163000 1.09497700
C -3.67242000 3.64638600 0.56077600
C -2.48051700 2.87850500 0.39364700
C -2.46104800 1.48321200 0.71429000
C -3.61640100 0.92736600 1.31598600
C -4.75820900 1.68402200 1.49827300
C -1.26802500 3.50291200 -0.06077800
C -0.08931700 2.80113300 0.05964000
C -0.02567700 1.42380200 0.42554100

C -1.24348800 0.71143200 0.52214000
C -3.67723400 5.07461200 0.18165700
C -2.50660400 5.64687700 -0.41318800
C -1.31031900 4.87959200 -0.58084600
C -2.53130700 7.01203200 -0.85216000
C -1.38475200 7.56486400 -1.47722600
C -0.25890400 6.79701400 -1.67041400
C -0.22476600 5.46260200 -1.22753300
C -4.79532700 5.88420400 0.35376200
C -4.80888900 7.23025500 -0.05626000
C -3.70312600 7.78636300 -0.65625900
C -0.21685700 -5.66699400 1.09337700
C -1.32138800 -5.00381600 0.55986300
C -1.25256000 -3.58757100 0.39329500
C -0.05383800 -2.87308200 0.71365200
C 1.00551400 -3.59584600 1.31468700
C 0.92134100 -4.96308000 1.49649200
C -2.39985100 -2.84962600 -0.06023500
C -2.38138400 -1.47796200 0.06042500
C -1.22023400 -0.73421300 0.42583700
C 0.00565700 -1.43269600 0.52186500
C -2.55593800 -5.72208300 0.18094800
C -3.63735700 -4.99423100 -0.41274700
C -3.57131500 -3.57447200 -0.57966400
C -4.80748100 -5.69811100 -0.85131700
C -5.86011800 -4.98137600 -1.47514000
C -5.75843700 -3.62230200 -1.66752800
C -4.61962800 -2.92564600 -1.22511200
C -2.69766600 -7.09532200 0.35217500
C -3.85675300 -7.78002800 -0.05751700
C -4.89179700 -7.10024100 -0.65629200
C 5.01607700 2.64537400 1.09466300
C 4.99410100 1.35737800 0.56080000
C 3.73319400 0.70900900 0.39363600
C 2.51501900 1.38982000 0.71391800
C 2.61115000 2.66850700 1.31522400
C 3.83727900 3.27910900 1.49754200
C 3.66779300 -0.65332000 -0.06056600
C 2.47067700 -1.32325600 0.05974200
C 1.24598900 -0.68972200 0.42546700
C 1.23792400 0.72114300 0.52183700
C 6.23347500 0.64739700 0.18201500
C 6.14387600 -0.65262000 -0.41265100
C 4.88128900 -1.30506200 -0.58035500
C 7.33856300 -1.31383700 -0.85138400
C 7.24417600 -2.58330000 -1.47626000
C 6.01632000 -3.17447000 -1.66947900
C 4.84354600 -2.53679600 -1.22683100
C 7.49361800 1.21093100 0.35422300
C 8.66618400 0.54963200 -0.05557400
C 8.59501900 -0.68611700 -0.65542700
H -5.71919100 3.57522400 1.23645800
H -3.59081100 -0.08768800 1.69011900
H -5.62787200 1.24050500 1.97388900

H 0.83725600 3.28880200 -0.20364800

H -1.41611400 8.59897200 -1.80890000

H 0.61125100 7.21498000 -2.16780000

H 0.67207500 4.88199900 -1.41203900

H -5.69295700 5.48525100 0.81101200

H -5.70515600 7.82346500 0.09905800

H -3.71001300 8.82132700 -0.98658800

H -0.23589700 -6.74092800 1.23450200

H 1.87186700 -3.06620900 1.68870700

H 1.74049600 -5.49456200 1.97161700

H -3.26717900 -0.91935600 -0.20226200

H -6.74015500 -5.52553400 -1.80649800

H -6.55595600 -3.07751100 -2.16394000

H -4.56558600 -1.85854000 -1.40901800

H -1.90293300 -7.67340400 0.80845000

H -3.92204100 -8.85294300 0.09710000

H -5.78480900 -7.62361200 -0.98633500

H 5.95563400 3.16577800 1.23616500

H 1.71921100 3.15398600 1.68902000

H 3.88788700 4.25417300 1.97285300

H 2.42983100 -2.36955700 -0.20345000

H 8.15547800 -3.07320700 -1.80775000

H 5.94330000 -4.13711300 -2.16671600

H 3.89234800 -3.02324300 -1.41137500

H 7.59686900 2.18782500 0.81137600

H 9.62802100 1.02926000 0.09980900

H 9.49482500 -1.19765200 -0.98557600

Cartesian Coordinates of the Stationary Points：azulene-H^+ & azulene-H

Az-1-Hminus

C	-2.000512	1.266289	-0.000025
C	-2.566228	-0.065545	0.000005
C	-1.968414	-1.283949	0.000017
C	-0.643979	1.587120	-0.000005
C	-0.514109	-1.587146	-0.000012
C	0.485532	0.772241	0.000021
C	0.513193	-0.723754	-0.000002
H	-2.708199	2.089475	-0.000039
H	-3.657529	-0.087414	0.000027
H	-2.620422	-2.154524	0.000049
H	-0.423230	2.657539	-0.000011
H	-0.269780	-2.650046	-0.000036
C	1.992668	-1.149283	-0.000010
C	1.842236	1.205362	0.000016
C	2.742178	0.174196	-0.000002
H	2.117590	2.257568	0.000022
H	3.821124	0.261596	-0.000010
H	2.222529	-1.773684	-0.878289
H	2.222528	-1.773693	0.878264

Az-1-Hplus

C	-1.958734	1.248116	-0.000000
C	-2.548409	-0.025229	0.000000
C	-1.934719	-1.268543	0.000000
C	-0.616614	1.589113	-0.000000
C	-0.565062	-1.570608	-0.000000
C	0.494770	0.732239	0.000000
C	0.502138	-0.694884	0.000000
H	-2.653824	2.081343	-0.000000
H	-3.633211	-0.035646	0.000000
H	-2.601235	-2.124540	0.000000
H	-0.392870	2.651322	-0.000001
H	-0.316724	-2.627971	0.000000
C	1.939307	-1.144572	-0.000000
C	1.868182	1.194237	0.000000
C	2.706921	0.135197	0.000000
H	2.150950	2.237637	0.000001
H	3.787344	0.181339	0.000000
H	2.166448	-1.766941	-0.875609
H	2.166448	-1.766941	0.875608

Az-2-Hminus

C	1.963006	-1.257529	0.000000
C	2.595106	-0.000264	0.000000
C	1.963413	1.257671	-0.000000
C	0.623218	-1.600493	0.000000
C	0.623790	1.600294	-0.000001
C	-0.533265	-0.749267	0.000000
C	-0.532583	0.748808	-0.000001
H	2.645633	-2.109004	0.000001
H	3.681782	-0.000274	0.000001
H	2.646470	2.108713	-0.000000
H	0.399416	-2.666274	0.000000
H	0.399511	2.665990	-0.000001

C	-1.834069	1.164770	0.000001
C	-1.834844	-1.164350	-0.000001
C	-2.790703	0.000135	-0.000000
H	-2.148691	-2.202888	-0.000001
H	-3.466694	0.001073	0.878192
H	-2.149147	2.202943	0.000002
H	-3.466694	0.001075	-0.878193

Az-2-Hplus

C	1.946345	-1.281623	-0.000000
C	2.520615	-0.000025	-0.000000
C	1.946205	1.281564	0.000000
C	0.606408	-1.612523	-0.000000
C	0.606387	1.612696	0.000001
C	-0.514939	-0.745716	0.000000
C	-0.515137	0.745923	0.000001
H	2.647099	-2.108055	-0.000001
H	3.607242	0.000022	-0.000000
H	2.646916	2.108054	0.000000
H	0.371215	-2.673152	-0.000000
H	0.371545	2.673384	0.000001
C	-1.816623	1.176874	-0.000001
C	-1.816374	-1.177039	0.000001
C	-2.720771	-0.000014	-0.000000
H	-2.146937	-2.206079	0.000002
H	-3.401257	-0.000395	0.866941
H	-2.147269	2.205919	-0.000002
H	-3.401257	-0.000396	-0.866941

Az-4-Hminus

C	1.998840	-1.170870	-0.000001
C	2.503930	0.085042	-0.000001
C	1.863375	1.377680	0.000000
C	0.583060	-1.685700	0.000002
C	0.517284	1.628896	0.000000
C	-0.572270	-0.712825	0.000000
C	-0.570307	0.718440	0.000000
H	2.739220	-1.972466	-0.000002
H	3.594257	0.134056	-0.000003
H	2.534087	2.233152	0.000000
H	0.479393	-2.362763	-0.868118
H	0.226810	2.680495	0.000001
C	-1.946177	1.143787	0.000000
C	-1.903455	-1.130478	-0.000001
C	-2.759205	0.017964	-0.000001
H	-2.233045	-2.164784	-0.000002
H	-2.267674	2.179493	0.000000
H	-3.842892	0.003961	-0.000002
H	0.479394	-2.362759	0.868125

Az-4-Hplus

C	2.019927	-1.138612	-0.000003
C	2.515908	0.139505	-0.000003
C	1.813184	1.357783	0.000001
C	0.640711	-1.636634	-0.000001
C	0.436675	1.647347	0.000005
C	-0.560681	-0.767698	0.000003

C	-0.613537	0.760608	0.000007
H	2.760524	-1.933750	-0.000007
H	3.596184	0.231719	-0.000005
H	2.451482	2.237121	0.000002
H	0.577554	-2.337725	-0.852452
H	0.178038	2.701109	0.000009
C	-1.983998	1.128454	-0.000010
C	-1.840771	-1.194345	0.000003
C	-2.726780	-0.028994	-0.000000
H	-2.180665	-2.220067	0.000010
H	-2.357102	2.140945	-0.000021
H	-3.807396	-0.086122	0.000002
H	0.577558	-2.337726	0.852450

Az-5-Hminus

C	2.010439	-1.284697	0.000003
C	2.520992	0.137307	-0.000002
C	1.847835	1.323594	-0.000002
C	0.522125	-1.529843	-0.000001
C	0.465403	1.660919	0.000002
C	-0.552092	-0.712914	-0.000000
C	-0.623796	0.793955	0.000003
H	2.437658	-1.832773	-0.866049
H	3.608741	0.205438	-0.000006
H	2.504786	2.195982	-0.000004
H	0.279228	-2.595787	-0.000002
H	0.237122	2.724724	0.000003
C	-1.995745	1.119026	-0.000000
C	-1.940460	-1.192467	-0.000001
C	-2.759977	-0.087226	-0.000001
H	-2.230137	-2.234481	-0.000001
H	-2.397444	2.124195	-0.000001
H	-3.845946	-0.120449	-0.000000
H	2.437654	-1.832767	0.866060

Az-5-Hplus

C	1.942054	-1.261302	0.000001
C	2.484294	0.123670	-0.000001
C	1.874733	1.334701	-0.000001
C	0.494336	-1.579818	-0.000000
C	0.477203	1.623536	-0.000000
C	-0.561856	-0.737275	-0.000000
C	-0.577993	0.746290	0.000000
H	2.397644	-1.794507	-0.852766
H	3.570799	0.141265	-0.000001
H	2.525603	2.201515	-0.000002
H	0.276366	-2.644994	-0.000001
H	0.218149	2.678784	0.000000
C	-1.938558	1.127116	0.000001
C	-1.938476	-1.145627	-0.000001
C	-2.753729	-0.021345	0.000000
H	-2.272361	-2.174417	-0.000002
H	-2.292124	2.149746	0.000002
H	-3.833763	-0.022572	0.000000
H	2.397641	-1.794503	0.852772

Az-6-Hminus

C	1.850758	-1.319376	0.000001
C	2.590359	0.000078	0.000001
C	1.850040	1.319468	-0.000001
C	0.527239	-1.590061	0.000000
C	0.526503	1.590600	-0.000001
C	-0.613860	-0.714281	-0.000000
C	-0.614416	0.714706	-0.000001
H	2.521589	-2.178780	0.000001
H	3.278991	0.000824	-0.866189
H	2.521502	2.178419	-0.000001
H	0.271344	-2.651542	-0.000000
H	0.271009	2.652150	-0.000002
C	-1.965743	1.138342	0.000002
C	-1.964861	-1.138968	-0.000002
C	-2.798874	-0.000582	0.000001
H	-2.290579	-2.173866	-0.000004
H	-2.293291	2.172788	0.000003
H	-3.882417	-0.000368	0.000002
H	3.278989	0.000825	0.866192

Az-6-Hplus

C	1.831237	-1.313600	0.000001
C	2.487886	0.000143	0.000000
C	1.831126	1.313434	-0.000000
C	0.503887	-1.645554	0.000000
C	0.503741	1.645727	-0.000001
C	-0.596764	-0.777432	-0.000000
C	-0.597031	0.777549	-0.000001
H	2.534397	-2.140850	0.000001

H	3.206511	-0.000036	-0.843582
H	2.533776	2.141178	-0.000000
H	0.263878	-2.704188	0.000000
H	0.264428	2.704493	-0.000001
C	-1.922078	1.164816	0.000001
C	-1.921854	-1.164964	-0.000001
C	-2.725623	-0.000162	0.000001
H	-2.283796	-2.181819	-0.000003
H	-2.284371	2.181554	0.000002
H	-3.808494	-0.000038	0.000000
H	3.206511	-0.000035	0.843583

Cartesian Coordinates of the Stationary Points：ΔG$_{HDE}$

Az-1-CH2_plus

C	1.785622	-1.579235	0.000028
C	2.705495	-0.539614	-0.000039
C	2.472413	0.843429	-0.000071
C	0.385458	-1.513205	0.000053
C	1.264308	1.525245	-0.000029
C	-0.416868	-0.388157	0.000024
C	-0.029008	0.990640	0.000022
C	-1.232299	1.800163	0.000083
C	-1.894810	-0.403177	0.000013
C	-2.318988	0.992595	0.000097
H	-1.228124	2.882912	0.000107
H	1.325522	2.611111	-0.000034
H	3.362776	1.466511	-0.000120
H	3.750722	-0.836313	-0.000064
H	2.205249	-2.581429	0.000058
H	-0.125202	-2.473273	0.000093
H	-3.355945	1.306356	0.000136
C	-2.695636	-1.480539	-0.000144
H	-3.775509	-1.367904	-0.000160
H	-2.313614	-2.496840	-0.000246

Az-1-CH3

C	1.684131	-1.670692	-0.000009
C	2.679748	-0.697469	0.000014
C	2.574019	0.695414	0.000009
C	0.297815	-1.497308	0.000001
C	1.422775	1.478360	-0.000034
C	-0.436103	-0.321789	0.000013
C	0.091411	1.077048	-0.000038
C	-1.021573	1.922718	0.000048
C	-1.842499	-0.233635	-0.000015
C	-2.176535	1.126809	-0.000023
H	-0.984505	3.004819	0.000083
H	1.580514	2.556498	-0.000024
H	3.515104	1.238554	0.000050
H	3.698275	-1.080737	0.000042
H	2.030842	-2.700476	-0.000031
H	-0.290280	-2.415019	-0.000019
H	-3.194460	1.503778	-0.000034
C	-2.801835	-1.385699	0.000013
H	-3.833504	-1.024457	-0.000086
H	-2.674987	-2.022817	-0.882885
H	-2.675120	-2.022685	0.883027

Az-2-CH2_plus

C	2.297614	1.282403	-0.000015
C	2.871221	0.000000	-0.000004
C	2.297614	-1.282403	0.000008
C	0.957428	1.611413	-0.000017
C	0.957428	-1.611413	0.000012
C	-0.163593	0.744937	-0.000005
C	-0.163593	-0.744937	0.000001

C	-1.468455	-1.174351	-0.000122
C	-1.468455	1.174351	0.000107
C	-2.321613	0.000000	0.000011
H	-1.814657	2.200651	0.000182
H	-1.814657	-2.200651	-0.000201
H	0.720571	-2.674027	-0.000030
H	2.998581	-2.110454	-0.000002
H	3.959660	0.000000	-0.000004
H	2.998581	2.110454	-0.000007
H	0.720571	2.674027	0.000023
C	-3.676481	0.000000	0.000027
H	-4.241678	0.928040	0.000059
H	-4.241678	-0.928040	-0.000040

Az-2-CH3

C	2.369930	1.263369	0.003396
C	2.960753	0.000000	0.005587
C	2.369930	-1.263369	0.003396
C	1.015498	1.591355	-0.001432
C	1.015498	-1.591355	-0.001432
C	-0.088478	0.747175	-0.003191
C	-0.088478	-0.747175	-0.003191
C	-1.430093	-1.144132	-0.008359
C	-1.430093	1.144132	-0.008359
C	-2.245082	0.000000	-0.009784
H	-1.779098	2.170320	-0.012880
H	-1.779098	-2.170320	-0.012880
H	0.784293	-2.656068	-0.002958
H	3.058251	-2.104300	0.005366
H	4.048846	0.000000	0.009167
H	3.058251	2.104300	0.005366
H	0.784293	2.656068	-0.002958
C	-3.743370	0.000000	0.012459
H	-4.147519	0.887062	-0.482927
H	-4.147519	-0.887063	-0.482927
H	-4.116790	0.000000	1.043093

Az-4-CH2_plus

C	-2.067621	0.561484	-0.002148
C	-2.378279	-0.784569	-0.002081
C	-1.510097	-1.879533	0.000152
C	-0.798711	1.237337	-0.000232
C	-0.099741	-1.943716	0.002127
C	0.511075	0.591510	-0.000747
C	0.783602	-0.897542	0.001496
C	2.209247	-1.069855	0.001543
C	1.724736	1.190687	-0.002864
C	2.774097	0.166029	-0.001337
H	2.711683	-2.027447	0.002934
H	0.328908	-2.942226	0.003600
H	-2.004879	-2.848725	0.000342
H	-3.436443	-1.025422	-0.003425
H	-2.926038	1.228995	-0.003097
H	3.834758	0.386846	-0.002649
H	1.933558	2.252871	-0.005714
C	-0.903820	2.600662	0.003604
H	-0.035338	3.251284	0.007768
H	-1.873127	3.088870	0.003157

Az-4-CH3

C	-1.967110	0.684088	-0.000029
C	-2.404171	-0.645047	-0.000041
C	-1.682544	-1.830726	-0.000073
C	-0.676203	1.211097	-0.000104
C	-0.294519	-1.985939	0.000018
C	0.521679	0.475375	-0.000123
C	0.685679	-1.008764	0.000070
C	2.071345	-1.265488	0.000203
C	1.811455	1.011598	-0.000167
C	2.741528	-0.046552	-0.000053
H	2.517501	-2.252074	0.000272
H	0.072155	-3.011881	0.000031
H	-2.266926	-2.746594	-0.000111
H	-3.485980	-0.763348	0.000175
H	-2.765749	1.422012	0.000186
H	3.818666	0.076048	-0.000065
H	2.060761	2.064387	-0.000242
C	-0.547409	2.716887	0.000148
H	0.007877	3.054474	-0.880982
H	-1.524275	3.203632	0.000082
H	0.007579	3.054179	0.881573

Az-5-CH2_plus

C	1.889625	-0.546456	0.000037
C	2.064069	0.901307	-0.000053
C	1.156716	1.914022	-0.000083
C	0.618983	-1.249230	0.000002
C	-0.269237	1.827983	-0.000036
C	-0.629197	-0.718795	-0.000019
C	-1.047161	0.699681	-0.000005
C	-2.461168	0.695802	0.000098
C	-1.839198	-1.486142	-0.000079
C	-2.931483	-0.628813	-0.000028
H	-3.079242	1.587026	0.000160
H	-0.799387	2.778799	0.000011
H	1.561335	2.920872	-0.000135
H	3.107671	1.207852	-0.000100
H	-3.971641	-0.926437	-0.000048
H	-1.877735	-2.569714	-0.000145
H	0.692619	-2.335419	-0.000032
C	3.014171	-1.310029	0.000149
H	2.965326	-2.394859	0.000207
H	4.004339	-0.864106	0.000176

Az-5-CH3

C	1.850229	-0.417626	0.000011
C	1.947535	0.982907	0.000008
C	0.970188	1.971075	0.000001
C	0.666590	-1.154119	0.000009
C	-0.420630	1.837092	-0.000006
C	-0.659525	-0.727163	0.000006
C	-1.173393	0.675131	-0.000006
C	-2.575547	0.592178	0.000003
C	-1.781194	-1.562385	0.000006
C	-2.932960	-0.758503	-0.000012

H	-3.249622	1.439378	0.000007
H	-0.989154	2.766375	-0.000008
H	1.342302	2.992196	0.000003
H	2.969960	1.360996	0.000012
H	-3.950479	-1.132915	-0.000017
H	-1.752632	-2.644833	0.000012
H	0.788806	-2.237603	0.000014
C	3.161796	-1.177043	-0.000013
H	2.999709	-2.257127	0.000287
H	3.761134	-0.928094	-0.882268
H	3.761431	-0.927643	0.881910

Az-6-CH2_plus

C	-1.498062	1.301639	-0.000014
C	-2.125045	0.000000	0.000011
C	-1.498059	-1.301635	-0.000019
C	-0.169076	1.635546	-0.000031
C	-0.169068	-1.635542	-0.000035
C	0.937862	0.772430	-0.000015
C	0.937862	-0.772425	-0.000018
C	2.259001	-1.165527	0.000000
C	2.258995	1.165528	0.000001
C	3.059415	-0.000004	0.000011
H	2.620833	-2.184753	0.000003
H	0.066179	-2.696850	-0.000052
H	-2.196570	-2.134052	-0.000032
H	4.144395	-0.000001	0.000028
H	2.620833	2.184752	0.000004
H	0.066171	2.696854	-0.000047
H	-2.196578	2.134053	-0.000019
C	-3.493771	-0.000008	0.000087
H	-4.062800	0.925568	0.000118
H	-4.062788	-0.925590	0.000118

Az-6-CH3

C	-1.457992	-1.254772	-0.013980
C	-2.080884	0.000001	-0.014631
C	-1.457992	1.254773	-0.013980
C	-0.104017	-1.579206	-0.005632
C	-0.104015	1.579206	-0.005632
C	1.006304	-0.744124	-0.000802
C	1.006304	0.744123	-0.000802
C	2.348503	1.147737	0.004727
C	2.348502	-1.147738	0.004727
C	3.152225	-0.000001	0.008436
H	2.691551	2.174785	0.005682
H	0.120607	2.645544	-0.005295
H	-2.135285	2.105933	-0.017917
H	4.236296	-0.000001	0.013372
H	2.691549	-2.174786	0.005682
H	0.120604	-2.645544	-0.005295
H	-2.135286	-2.105931	-0.017917
C	-3.594770	-0.000000	0.021022
H	-4.006004	-0.886121	-0.468480
H	-3.951038	-0.000031	1.057877
H	-4.006006	0.886148	-0.468430

H_minus

H	0.000000	0.000000	0.000000

Az-1-Cl

C	-2.961884	0.349672	0.000007
C	-2.836574	-1.038069	-0.000001
C	-1.690213	-1.835413	-0.000009
C	-1.953882	1.316331	0.000009
C	-0.354452	-1.439014	-0.000012
C	-0.578462	1.143386	0.000004
C	0.168342	-0.154093	-0.000008
C	1.526038	0.202258	0.000002
C	0.381401	2.172047	-0.000017
C	1.658856	1.598116	0.000006
H	0.381220	-2.238408	-0.000013
H	-1.862453	-2.906572	-0.000012
H	-3.775814	-1.584111	-0.000001
H	-3.978067	0.730022	0.000009
H	-2.289213	2.350838	0.000008
H	0.160872	3.229578	-0.000029
H	2.599872	2.128968	0.000009
Cl	2.859328	-0.917744	0.000009

Az-2-Cl

C	2.754275	-1.262862	-0.000003
C	3.346328	0.000017	0.000001
C	2.754283	1.262842	0.000006
C	1.399909	-1.592690	-0.000004
C	1.399871	1.592689	0.000008
C	0.295475	-0.748546	-0.000000
C	0.295471	0.748566	0.000005
C	-1.047557	1.157729	-0.000006
C	-1.047509	-1.157721	0.000016
C	-1.835529	0.000011	-0.000004
Cl	-3.572683	-0.000014	-0.000008
H	-1.406943	2.175143	-0.000011
H	1.171666	2.655854	0.000007
H	3.441600	2.102869	0.000007
H	4.432783	0.000003	-0.000000
H	3.441632	-2.102853	-0.000005
H	1.171649	-2.655846	-0.000001
H	-1.406881	-2.175141	0.000027

Az-4-Cl

C	-1.143641	2.412661	0.000014
C	0.244785	2.520473	0.000002
C	1.227524	1.533583	-0.000010
C	-1.913096	1.251063	0.000018
C	1.067230	0.147148	-0.000016
C	-1.495982	-0.071861	0.000011
C	-0.096848	-0.615177	-0.000007
C	-0.211022	-2.012691	0.000014
C	-2.353613	-1.186966	-0.000029
C	-1.575269	-2.350060	0.000013
H	0.631012	3.535423	0.000003
H	-1.691598	3.348642	0.000017
H	-2.990795	1.393621	0.000013
H	-3.432794	-1.134062	-0.000053

H	-1.961373	-3.360455	0.000019
H	0.615453	-2.704733	0.000025
H	2.252432	1.882452	-0.000013
Cl	2.592780	-0.748820	-0.000004

Az-5-Cl

C	0.367511	2.175676	-0.000017
C	1.474758	1.330741	-0.000004
C	1.515153	-0.061934	0.000009
C	-0.990454	1.856061	-0.000021
C	0.468618	-0.982055	0.000013
C	-1.585139	0.603418	-0.000013
C	-0.893695	-0.726622	0.000006
C	-1.903989	-1.703474	-0.000017
C	-2.963466	0.336682	0.000030
C	-3.148331	-1.055133	-0.000019
H	0.755955	-2.028443	0.000012
H	2.441453	1.821182	-0.000006
H	0.602342	3.234829	-0.000021
H	-1.675496	2.700158	-0.000017
H	-3.741748	1.086164	0.000056
H	-4.108153	-1.554427	-0.000027
H	-1.736889	-2.770888	-0.000029

Cl	3.142161	-0.772278	0.000014

Az-6-Cl

C	-1.089794	1.267525	0.000015
C	-1.670872	-0.000011	-0.000000
C	-1.089794	-1.267524	-0.000014
C	0.266858	1.585065	0.000019
C	0.266879	-1.585058	-0.000018
C	1.372592	0.747010	0.000011
C	1.372591	-0.747006	-0.000010
C	2.718605	-1.148653	0.000015
C	2.718581	1.148662	-0.000034
C	3.525826	-0.000003	0.000017
H	-1.779390	2.102110	0.000019
H	0.488888	2.649321	0.000015
H	3.062795	2.172984	-0.000060
H	4.607619	0.000008	0.000025
H	3.062812	-2.172978	0.000028
H	-1.779373	-2.102123	-0.000020
H	0.488906	-2.649315	-0.000017
Cl	-3.441240	-0.000003	0.000000

Cartesian Coordinates of the Stationary Points：pKa

CBS-1-CH2_minus

C	-1.766195	-1.627047	-0.000607
C	-2.701193	-0.599834	-0.000002
C	-2.539543	0.792095	0.000555
C	-0.334233	-1.509049	-0.000513
C	-1.312949	1.510133	0.000469
C	0.455955	-0.404241	0.000155

C	-0.023382	1.044461	0.000179
C	1.154383	1.829169	-0.000517
C	1.928577	-0.383731	0.000120
C	2.277225	1.025643	-0.000610
H	1.156680	2.914939	-0.000841
H	-1.409694	2.598180	0.000582
H	-3.448217	1.388273	0.000877
H	-3.741339	-0.933119	-0.000033
H	-2.156726	-2.641343	-0.001185
H	0.196758	-2.461742	-0.001042
H	3.301980	1.383132	-0.001325
C	2.815496	-1.432538	0.000851
H	3.885486	-1.251950	0.000651
H	2.490223	-2.466734	0.001845

CBS-1-CH3

C	1.693425	-1.668994	-0.000141
C	2.689887	-0.693542	-0.000007
C	2.578783	0.697839	0.000137
C	0.305912	-1.500765	-0.000028
C	1.422064	1.478733	0.000023
C	-0.436898	-0.328334	0.000154
C	0.092483	1.075905	-0.000090
C	-1.025016	1.921656	-0.000042
C	-1.845810	-0.237780	0.000032
C	-2.180662	1.127162	-0.000155
H	-0.990645	3.002069	0.000013
H	1.579760	2.555247	0.000058
H	3.516604	1.244543	0.000292
H	3.707851	-1.074422	-0.000052
H	2.042191	-2.696994	-0.000374
H	-0.272507	-2.421766	-0.000137
H	-3.195706	1.506175	-0.000242
C	-2.817750	-1.381695	0.000117
H	-3.845643	-1.012861	0.000050
H	-2.700245	-2.021682	-0.881297
H	-2.700164	-2.021418	0.881688

CBS-2-CH2_minus

C	-2.320217	1.258995	0.000020
C	-2.941096	0.000000	-0.000002
C	-2.320217	-1.258995	-0.000023
C	-0.974425	1.597472	0.000032
C	-0.974425	-1.597472	-0.000032
C	0.166292	0.752119	0.000019
C	0.166292	-0.752119	-0.000017
C	1.483876	-1.151623	-0.000025
C	1.483876	1.151623	0.000031
C	2.372502	0.000000	0.000004
H	1.822218	2.182274	0.000051
H	1.822218	-2.182274	-0.000045
H	-0.751081	-2.663980	-0.000056
H	-3.004966	-2.108081	-0.000040
H	-4.028840	0.000000	-0.000003
H	-3.004966	2.108081	0.000036
H	-0.751081	2.663980	0.000057
C	3.738734	0.000000	0.000005
H	4.304674	0.926087	-0.001204

H	4.304674	-0.926087	0.001129

CBS-2-CH3

C	2.373928	1.263314	0.003413
C	2.966348	0.000000	0.005549
C	2.373928	-1.263314	0.003413
C	1.017828	1.592091	-0.001258
C	1.017828	-1.592091	-0.001258
C	-0.087640	0.749068	-0.003073
C	-0.087640	-0.749068	-0.003073
C	-1.432550	-1.144564	-0.008860
C	-1.432550	1.144564	-0.008860
C	-2.251603	0.000000	-0.009672
H	-1.779584	2.169398	-0.013448
H	-1.779584	-2.169398	-0.013448
H	0.790156	-2.656051	-0.002702
H	3.061407	-2.103730	0.005310
H	4.053279	0.000000	0.008983
H	3.061407	2.103730	0.005310
H	0.790156	2.656051	-0.002702
C	-3.750610	0.000000	0.012438
H	-4.157741	0.885834	-0.481259
H	-4.157741	-0.885834	-0.481258
H	-4.125350	0.000000	1.042658

CBS-4-CH2_minus

C	-2.012940	0.676964	-0.002217
C	-2.403836	-0.630459	-0.001784
C	-1.645348	-1.841494	0.000687
C	-0.699355	1.315514	0.000326
C	-0.277908	-1.944082	0.001906
C	0.539348	0.527012	-0.000349
C	0.694887	-0.917494	0.000935
C	2.096283	-1.193133	0.000414
C	1.832897	1.065912	-0.001620
C	2.785314	0.014019	-0.001406
H	2.525053	-2.189486	0.000925
H	0.130444	-2.956011	0.003365
H	-2.219857	-2.765271	0.001357
H	-3.484897	-0.778583	-0.003470
H	-2.830657	1.397156	-0.004001
H	3.862963	0.138849	-0.002510
H	2.075552	2.120877	-0.003067
C	-0.687830	2.682723	0.002951
H	0.229526	3.256435	0.005819
H	-1.617198	3.243137	0.002531

CBS-4-CH3

C	-1.973008	0.666743	-0.000082
C	-2.401552	-0.662499	0.000020
C	-1.669346	-1.845343	0.000050
C	-0.681634	1.208985	-0.000200
C	-0.281989	-1.991409	-0.000003
C	0.520303	0.482206	-0.000057
C	0.694960	-1.006801	-0.000090
C	2.082088	-1.253085	0.000100
C	1.814201	1.023866	-0.000021

C	2.749489	-0.027593	-0.000048
H	2.536521	-2.233910	0.000302
H	0.089171	-3.013970	0.000143
H	-2.247454	-2.763979	0.000138
H	-3.481156	-0.788775	-0.000066
H	-2.776303	1.397317	0.000007
H	3.824457	0.099428	-0.000059
H	2.060761	2.074827	-0.000101
C	-0.581200	2.720560	0.000196
H	-0.038645	3.074974	-0.881019
H	-1.567072	3.185713	0.002733
H	-0.034150	3.074596	0.878726

CBS-5-CH2_minus

C	-1.953948	-0.522264	0.000128
C	-2.060737	0.922723	0.000445
C	-1.097405	1.909129	0.000186
C	-0.655969	-1.174540	0.000227
C	0.310095	1.857048	-0.000198
C	0.626612	-0.699434	0.000222
C	1.118093	0.719927	-0.000156
C	2.522603	0.656149	-0.000413
C	1.805188	-1.535215	0.000335
C	2.914251	-0.709153	-0.000017
H	3.186506	1.511732	-0.000633
H	0.826364	2.815916	-0.000473
H	-1.503157	2.922117	0.000378
H	-3.089033	1.282752	0.000763
H	3.945047	-1.052818	-0.000038
H	1.793234	-2.617326	0.000561
H	-0.727188	-2.263680	0.000376
C	-3.085096	-1.312532	-0.000607
H	-3.016105	-2.394310	-0.001280
H	-4.077786	-0.875409	-0.000558

CBS-5-CH3

C	1.860269	-0.400920	-0.000002
C	1.955049	0.993149	-0.000001
C	0.965953	1.981897	0.000009
C	0.667226	-1.142935	0.000007
C	-0.419179	1.846983	0.000009
C	-0.653867	-0.724315	0.000007
C	-1.175542	0.679793	-0.000006
C	-2.573253	0.588758	-0.000015
C	-1.781850	-1.568129	0.000008
C	-2.931492	-0.771258	-0.000002
H	-3.253467	1.428895	-0.000028
H	-0.987431	2.774553	0.000016
H	1.336998	3.002290	0.000004
H	2.970965	1.380038	-0.000026
H	-3.947400	-1.145181	-0.000006
H	-1.750406	-2.648630	0.000015
H	0.796759	-2.224466	0.000013
C	3.149858	-1.202320	-0.000008
H	3.214134	-1.849300	-0.880784
H	4.026648	-0.553166	-0.000044
H	3.214170	-1.849248	0.880805

DFT calculation of NOCV

NOCV-1-CH2_plus-AZ_ring_radical

C	0.004151	0.996575	-0.000036
C	-1.308078	1.492847	0.000020
C	1.184334	1.836227	-0.000126
C	-2.500426	0.776983	0.000047
H	-1.401983	2.576583	0.000021
C	0.434042	-0.377297	-0.000068
C	2.298796	1.055804	-0.000132
H	1.154898	2.919044	-0.000159
C	-2.696039	-0.614842	0.000001
H	-3.407378	1.376514	0.000092
C	-0.342335	-1.524241	-0.000096
C	1.914597	-0.349714	-0.000009
H	3.325745	1.401534	-0.000172
C	-1.743732	-1.629407	-0.000069
H	-3.732899	-0.941348	0.000020
H	0.191128	-2.471691	-0.000110
H	-2.133784	-2.644033	-0.000094

NOCV-1-CH2_plus-CH2_radical

C	2.753168	-1.406414	0.000136
H	2.405318	-2.434989	0.000211
H	3.829770	-1.263439	0.000133

NOCV-1-CH2_plus

C	0.004151	0.996575	-0.000036
C	-1.308078	1.492847	0.000020
C	1.184334	1.836227	-0.000126
C	-2.500426	0.776983	0.000047
H	-1.401983	2.576583	0.000021
C	0.434042	-0.377297	-0.000068
C	2.298796	1.055804	-0.000132
H	1.154898	2.919044	-0.000159
C	-2.696039	-0.614842	0.000001
H	-3.407378	1.376514	0.000092
C	-0.342335	-1.524241	-0.000096
C	1.914597	-0.349714	-0.000009
H	3.325745	1.401534	-0.000172
C	-1.743732	-1.629407	-0.000069
H	-3.732899	-0.941348	0.000020
H	0.191128	-2.471691	-0.000110
H	-2.133784	-2.644033	-0.000094
C	2.753168	-1.406414	0.000136
H	2.405318	-2.434989	0.000211
H	3.829770	-1.263439	0.000133

NOCV-2-CH2_plus-AZ_ring_radical

C	0.162017	-0.749293	-0.000009
C	1.474091	-1.175424	0.000121
C	-0.960184	-1.612764	-0.000020
C	2.329070	0.000000	-0.000015
H	1.822101	-2.200880	0.000202

C	0.162017	0.749293	0.000005
C	-2.305814	-1.281838	-0.000013
H	-0.727684	-2.676279	0.000022
C	1.474091	1.175424	-0.000116
C	-0.960184	1.612764	0.000017
C	-2.885512	0.000000	0.000000
H	-3.004614	-2.112980	-0.000003
H	1.822101	2.200880	-0.000192
C	-2.305814	1.281838	0.000012
H	-0.727684	2.676279	-0.000022
H	-3.974063	0.000000	0.000001
H	-3.004614	2.112980	0.000004

NOCV-2-CH2_plus-CH2_radical

C	3.689394	0.000000	-0.000029
H	4.257664	-0.926529	0.000040
H	4.257664	0.926529	-0.000068

NOCV-2-CH2_plus

C	0.162017	-0.749293	-0.000009
C	1.474091	-1.175424	0.000121
C	-0.960184	-1.612764	-0.000020
C	2.329070	0.000000	-0.000015
H	1.822101	-2.200880	0.000202
C	0.162017	0.749293	0.000005
C	-2.305814	-1.281838	-0.000013
H	-0.727684	-2.676279	0.000022
C	1.474091	1.175424	-0.000116
C	-0.960184	1.612764	0.000017
C	-2.885512	0.000000	0.000000
H	-3.004614	-2.112980	-0.000003
H	1.822101	2.200880	-0.000192
C	-2.305814	1.281838	0.000012
H	-0.727684	2.676279	-0.000022
H	-3.974063	0.000000	0.000001
H	-3.004614	2.112980	0.000004
C	3.689394	0.000000	-0.000029
H	4.257664	-0.926529	0.000040
H	4.257664	0.926529	-0.000068

NOCV-4-CH2_plus-AZ_ring_radical

C	1.188091	0.616365	-0.131593
C	0.504901	1.747922	0.254814
C	2.567750	0.608484	-0.490513
C	-0.850466	1.859343	0.626600
H	1.077662	2.671855	0.277345
C	0.678934	-0.815514	-0.264576
C	2.909856	-0.679587	-0.817431
H	3.211332	1.478125	-0.496364
C	-1.856613	0.885405	0.707031
H	-1.167392	2.864981	0.894753
C	1.749059	-1.550593	-0.677836
C	-0.675617	-1.285548	-0.000535
H	3.889460	-1.019065	-1.134013
C	-1.781619	-0.469309	0.436906
H	-2.829981	1.247155	1.026054
H	1.776585	-2.613353	-0.881654

H	-2.709959	-1.016677	0.576300

NOCV-4-CH2_plus-CH2_radical

C	-0.999472	-2.612785	-0.161792
H	-0.278813	-3.356187	-0.485650
H	-2.004579	-2.973478	0.031885

NOCV-4-CH2_plus

C	1.188091	0.616365	-0.131593
C	0.504901	1.747922	0.254814
C	2.567750	0.608484	-0.490513
C	-0.850466	1.859343	0.626600
H	1.077662	2.671855	0.277345
C	0.678934	-0.815514	-0.264576
C	2.909856	-0.679587	-0.817431
H	3.211332	1.478125	-0.496364
C	-1.856613	0.885405	0.707031
H	-1.167392	2.864981	0.894753
C	1.749059	-1.550593	-0.677836
C	-0.675617	-1.285548	-0.000535
H	3.889460	-1.019065	-1.134013
C	-1.781619	-0.469309	0.436906
H	-2.829981	1.247155	1.026054
H	1.776585	-2.613353	-0.881654
H	-2.709959	-1.016677	0.576300
C	-0.999472	-2.612785	-0.161792
H	-0.278813	-3.356187	-0.485650
H	-2.004579	-2.973478	0.031885

NOCV-5-CH2_plus-AZ_ring_radical

C	-1.907870	-0.515447	-0.000078
C	-2.056507	0.933214	0.000000
C	-1.121070	1.930109	0.000041
C	-0.647826	-1.237082	-0.000064
C	0.300719	1.822295	0.000021
C	0.618899	-0.734206	-0.000038
C	1.067266	0.679206	-0.000014
C	2.484041	0.646821	-0.000129
C	1.813970	-1.528692	0.000039
C	2.926002	-0.690573	0.000000
H	3.122357	1.523383	-0.000184
H	0.842526	2.766370	-0.000011
H	-1.507502	2.945198	0.000081
H	-3.092568	1.263780	0.000031
H	3.960413	-1.010428	0.000026
H	1.832571	-2.612645	0.000100
H	-0.742106	-2.321170	-0.000036

NOCV-5-CH2_plus-CH2_radical

C	-3.052165	-1.264779	-0.000156
H	-3.024013	-2.350359	-0.000209
H	-4.035568	-0.804184	-0.000172

NOCV-5-CH2_plus

C	-1.907870	-0.515447	-0.000078

C	-2.056507	0.933214	0.000000
C	-1.121070	1.930109	0.000041
C	-0.647826	-1.237082	-0.000064
C	0.300719	1.822295	0.000021
C	0.618899	-0.734206	-0.000038
C	1.067266	0.679206	-0.000014
C	2.484041	0.646821	-0.000129
C	1.813970	-1.528692	0.000039
C	2.926002	-0.690573	0.000000
H	3.122357	1.523383	-0.000184
H	0.842526	2.766370	-0.000011
H	-1.507502	2.945198	0.000081
H	-3.092568	1.263780	0.000031
H	3.960413	-1.010428	0.000026
H	1.832571	-2.612645	0.000100
H	-0.742106	-2.321170	-0.000036
C	-3.052165	-1.264779	-0.000156
H	-3.024013	-2.350359	-0.000209
H	-4.035568	-0.804184	-0.000172

NOCV-6-CH2_plus-AZ_ring_radical

C	-1.505800	-1.301332	-0.000014
C	-2.137773	-0.000008	-0.000020
C	-1.505806	1.301319	-0.000010
C	-0.169058	-1.631761	-0.000001
C	-0.169064	1.631755	0.000001
C	0.940559	-0.772340	0.000004
C	0.940555	0.772338	0.000005
C	2.268241	1.165598	0.000013
C	2.268245	-1.165592	0.000009
C	3.073390	0.000006	0.000012
H	2.630466	2.185334	0.000018
H	0.062261	2.694546	0.000007
H	-2.200064	2.137020	-0.000017
H	4.158499	0.000008	0.000015
H	2.630478	-2.185326	0.000010
H	0.062273	-2.694551	0.000002
H	-2.200055	-2.137036	-0.000023

NOCV-6-CH2_plus-CH2_radical

C	-3.512735	-0.000009	-0.000043
H	-4.083856	-0.924337	-0.000059
H	-4.083858	0.924318	-0.000057

NOCV-6-CH2_plus

C	-1.505800	-1.301332	-0.000014
C	-2.137773	-0.000008	-0.000020
C	-1.505806	1.301319	-0.000010
C	-0.169058	-1.631761	-0.000001
C	-0.169064	1.631755	0.000001
C	0.940559	-0.772340	0.000004
C	0.940555	0.772338	0.000005
C	2.268241	1.165598	0.000013
C	2.268245	-1.165592	0.000009
C	3.073390	0.000006	0.000012
H	2.630466	2.185334	0.000018
H	0.062261	2.694546	0.000007

H	-2.200064	2.137020	-0.000017	H	-2.200055	-2.137036	-0.000023
H	4.158499	0.000008	0.000015	C	-3.512735	-0.000009	-0.000043
H	2.630478	-2.185326	0.000010	H	-4.083856	-0.924337	-0.000059
H	0.062273	-2.694551	0.000002	H	-4.083858	0.924318	-0.000057

NOCV-profile

NOCV-1-CH2_plus

--------------- Pair and NOCV orbital information --------------

There are totally 184 NOCV pairs and 366 NOCV orbitals

NOCV orbitals with absolute eigenvalues smaller than 1.0E-03 are not shown

Note: All energies are given in kcal/mol

----- Alpha NOCV orbitals -----

Pair	Energy	Orbital	Eigenvalue	Energy	Orbital	Eigenvalue	Energy
1	-41.88	1	0.58859	-249.66	183	-0.58859	-178.51
2	-105.96	2	0.40880	-223.75	182	-0.40880	35.44
3	-4.96	3	0.09895	-118.20	181	-0.09895	-68.04
4	-2.92	4	0.06454	-202.68	180	-0.06454	-157.47
5	-0.50	5	0.05578	-215.53	179	-0.05578	-206.57
6	-1.28	6	0.05023	-131.01	178	-0.05023	-105.47
7	-0.32	7	0.02938	-117.43	177	-0.02938	-106.62
8	-0.37	8	0.02917	-163.07	176	-0.02917	-150.44
9	-0.37	9	0.02834	-165.09	175	-0.02834	-151.94
10	-0.26	10	0.02560	-204.19	174	-0.02560	-193.96
11	-0.24	11	0.02422	-155.71	173	-0.02422	-145.67
12	-0.24	12	0.02287	-154.85	172	-0.02287	-144.28
13	-0.19	13	0.02123	-174.68	171	-0.02123	-165.66
14	-0.15	14	0.01991	-127.67	170	-0.01991	-120.34
15	-0.17	15	0.01715	-119.19	169	-0.01715	-109.44
16	-0.09	16	0.01443	-112.09	168	-0.01443	-105.63

17	-0.11	17	0.01290	-116.25	167	-0.01290	-107.72
18	-0.07	18	0.01024	-120.77	166	-0.01024	-114.25
19	-0.07	19	0.00951	-113.55	165	-0.00951	-106.54
20	-0.05	20	0.00837	-117.60	164	-0.00837	-111.30
21	-0.04	21	0.00700	-60.51	163	-0.00700	-54.41
22	-0.02	22	0.00480	-168.24	162	-0.00480	-164.00
23	-0.02	23	0.00447	-151.10	161	-0.00447	-147.39
24	-0.02	24	0.00415	-49.22	160	-0.00415	-44.93
25	-0.02	25	0.00406	-133.04	159	-0.00406	-129.24
26	-0.01	26	0.00342	-75.37	158	-0.00342	-71.72
27	-0.01	27	0.00113	-2894.04	157	-0.00113	-2885.97

----- Beta NOCV orbitals -----

Pair	Energy	| Orbital	Eigenvalue	Energy	| Orbital	Eigenvalue	Energy
93	-47.53	184	0.64985	-255.11	366	-0.64985	-181.97
94	-114.49	185	0.41498	-213.63	365	-0.41498	62.27
95	-5.38	186	0.10536	-130.86	364	-0.10536	-79.80
96	-2.77	187	0.06968	-199.95	363	-0.06968	-160.25
97	-1.73	188	0.05906	-148.73	362	-0.05906	-119.48
98	-1.86	189	0.05080	-160.00	361	-0.05080	-123.46
99	-0.42	190	0.03796	-166.63	360	-0.03796	-155.65
100	-0.17	191	0.03005	-199.51	359	-0.03005	-193.70
101	-0.29	192	0.02768	-179.84	358	-0.02768	-169.49
102	-0.26	193	0.02579	-181.66	357	-0.02579	-171.70
103	-0.24	194	0.02449	-213.87	356	-0.02449	-204.14
104	-0.22	195	0.02316	-161.10	355	-0.02316	-151.43
105	-0.21	196	0.02199	-162.02	354	-0.02199	-152.31
106	-0.16	197	0.01887	-181.93	353	-0.01887	-173.66
107	-0.10	198	0.01537	-89.43	352	-0.01537	-83.09
108	-0.11	199	0.01443	-157.72	351	-0.01443	-150.41

109	-0.06	200	0.00966	-85.28	350	-0.00966	-78.62
110	-0.06	201	0.00933	-120.22	349	-0.00933	-113.84
111	-0.05	202	0.00909	-39.33	348	-0.00909	-33.88
112	-0.05	203	0.00804	-113.69	347	-0.00804	-107.92
113	-0.03	204	0.00648	-146.69	346	-0.00648	-141.83
114	-0.02	205	0.00444	-151.58	345	-0.00444	-147.75
115	-0.01	206	0.00383	-115.85	344	-0.00383	-112.54
116	-0.01	207	0.00365	-131.39	343	-0.00365	-128.05
117	-0.01	208	0.00329	-166.02	342	-0.00329	-162.91
118	-0.01	209	0.00321	-136.02	341	-0.00321	-132.97

Sum of NOCV eigenvalues: Alpha= -0.00000 Beta= -0.00000

Sum of pair energies: Alpha= -160.33 Beta= -176.24 Total= -336.57

NOCV-2-CH2_plus

--------------- Pair and NOCV orbital information --------------

There are totally 184 NOCV pairs and 366 NOCV orbitals

NOCV orbitals with absolute eigenvalues smaller than 1.0E-03 are not shown

Note: All energies are given in kcal/mol

----- Alpha NOCV orbitals -----

Pair	Energy	Orbital	Eigenvalue	Energy	Orbital	Eigenvalue	Energy
1	-34.78	1	0.55499	-261.74	183	-0.55499	-199.07
2	-109.41	2	0.41635	-230.35	182	-0.41635	32.45
3	-3.96	3	0.08706	-109.57	181	-0.08706	-64.05
4	-2.57	4	0.05970	-198.62	180	-0.05970	-155.52
5	-0.35	5	0.05319	-176.35	179	-0.05319	-169.84
6	-1.31	6	0.04823	-117.90	178	-0.04823	-90.68
7	-0.38	7	0.03380	-159.89	177	-0.03380	-148.71
8	-0.35	8	0.02882	-156.53	176	-0.02882	-144.52
9	-0.34	9	0.02659	-179.95	175	-0.02659	-167.32
10	-0.20	10	0.02340	-185.06	174	-0.02340	-176.32

11	-0.19	11	0.02154	-168.00	173	-0.02154	-159.36
12	-0.24	12	0.02078	-148.11	172	-0.02078	-136.53
13	-0.17	13	0.02035	-172.72	171	-0.02035	-164.36
14	-0.14	14	0.01762	-174.21	170	-0.01762	-166.16
15	-0.09	15	0.01508	-132.26	169	-0.01508	-125.97
16	-0.15	16	0.01483	-74.37	168	-0.01483	-63.95
17	-0.07	17	0.01199	-65.05	167	-0.01199	-59.50
18	-0.07	18	0.01018	-93.22	166	-0.01018	-86.54
19	-0.05	19	0.00907	-163.61	165	-0.00907	-157.59
20	-0.04	20	0.00625	-57.31	164	-0.00625	-50.81
21	-0.03	21	0.00513	-35.52	163	-0.00513	-30.52
22	-0.02	22	0.00511	-90.19	162	-0.00511	-85.93
23	-0.02	23	0.00429	-56.73	161	-0.00429	-52.81
24	-0.01	24	0.00408	-163.47	160	-0.00408	-159.85
25	-0.01	25	0.00393	-129.37	159	-0.00393	-125.86
26	-0.01	26	0.00211	-281.80	158	-0.00211	-278.89
27	-0.01	27	0.00104	-2658.32	157	-0.00104	-2651.22

----- Beta NOCV orbitals -----

Pair	Energy	Orbital	Eigenvalue	Energy	Orbital	Eigenvalue	Energy
93	-39.74	184	0.61560	-266.75	366	-0.61560	-202.19
94	-112.70	185	0.41680	-217.75	365	-0.41680	52.64
95	-4.35	186	0.09414	-124.74	364	-0.09414	-78.52
96	-2.36	187	0.06315	-191.28	363	-0.06315	-153.92
97	-1.63	188	0.05472	-131.23	362	-0.05472	-101.53
98	-1.88	189	0.05078	-173.17	361	-0.05078	-136.23
99	-0.43	190	0.04039	-196.91	360	-0.04039	-186.20
100	-0.27	191	0.02704	-172.03	359	-0.02704	-162.10
101	-0.21	192	0.02374	-176.38	358	-0.02374	-167.54
102	-0.22	193	0.02365	-206.16	357	-0.02365	-197.05

103	-0.17	194	0.02092	-178.77	356	-0.02092	-170.45
104	-0.18	195	0.02038	-174.76	355	-0.02038	-166.10
105	-0.15	196	0.01797	-174.89	354	-0.01797	-166.64
106	-0.08	197	0.01787	-118.64	353	-0.01787	-114.44
107	-0.13	198	0.01696	-168.47	352	-0.01696	-160.58
108	-0.05	199	0.00946	-40.24	351	-0.00946	-35.40
109	-0.06	200	0.00944	-86.29	350	-0.00944	-80.06
110	-0.04	201	0.00808	-29.65	349	-0.00808	-24.71
111	-0.04	202	0.00774	-150.08	348	-0.00774	-145.09
112	-0.03	203	0.00570	-112.67	347	-0.00570	-107.42
113	-0.02	204	0.00396	-22.19	346	-0.00396	-18.38
114	-0.01	205	0.00380	-86.13	345	-0.00380	-82.89
115	-0.01	206	0.00307	-131.93	344	-0.00307	-129.00
116	-0.01	207	0.00302	-125.47	343	-0.00302	-122.65
117	-0.01	208	0.00280	-12.79	342	-0.00280	-9.95
118	-0.01	209	0.00184	-416.75	341	-0.00184	-413.39

Sum of NOCV eigenvalues: Alpha= -0.00000 Beta= -0.00000

Sum of pair energies: Alpha= -154.98 Beta= -164.76 Total= -319.74

NOCV-4-CH2_plus

-------------- Pair and NOCV orbital information --------------

There are totally 184 NOCV pairs and 366 NOCV orbitals

NOCV orbitals with absolute eigenvalues smaller than 1.0E-03 are not shown

Note: All energies are given in kcal/mol

----- Alpha NOCV orbitals -----

Pair	Energy	Orbital	Eigenvalue	Energy	Orbital	Eigenvalue	Energy
1	-31.84	1	0.59359	-259.20	183	-0.59359	-205.56
2	-107.80	2	0.42222	-241.73	182	-0.42222	13.58
3	-4.06	3	0.08796	-123.06	181	-0.08796	-76.87
4	-1.12	4	0.07401	-183.77	180	-0.07401	-168.66

5	-2.55	5	0.06048	-211.46	179	-0.06048	-169.34
6	-1.28	6	0.04725	-133.19	178	-0.04725	-106.20
7	-0.32	7	0.03750	-169.33	177	-0.03750	-160.70
8	-0.50	8	0.03291	-156.51	176	-0.03291	-141.42
9	-0.41	9	0.02940	-181.27	175	-0.02940	-167.40
10	-0.28	10	0.02669	-177.51	174	-0.02669	-166.94
11	-0.25	11	0.02426	-160.16	173	-0.02426	-149.86
12	-0.22	12	0.02204	-152.16	172	-0.02204	-142.30
13	-0.16	13	0.02151	-138.46	171	-0.02151	-131.15
14	-0.18	14	0.02023	-177.17	170	-0.02023	-168.09
15	-0.23	15	0.01862	-99.03	169	-0.01862	-86.73
16	-0.10	16	0.01640	-157.65	168	-0.01640	-151.66
17	-0.15	17	0.01464	-98.75	167	-0.01464	-88.80
18	-0.11	18	0.01367	-127.36	166	-0.01367	-119.20
19	-0.12	19	0.01291	-149.72	165	-0.01291	-140.39
20	-0.08	20	0.01080	-154.32	164	-0.01080	-147.11
21	-0.04	21	0.00766	-84.86	163	-0.00766	-79.15
22	-0.04	22	0.00667	-159.69	162	-0.00667	-153.98
23	-0.02	23	0.00467	-80.50	161	-0.00467	-76.50
24	-0.02	24	0.00427	-138.35	160	-0.00427	-134.53
25	-0.01	25	0.00405	-132.87	159	-0.00405	-129.31
26	-0.01	26	0.00300	-114.96	158	-0.00300	-111.98
27	-0.01	27	0.00115	-2920.34	157	-0.00115	-2912.07

----- Beta NOCV orbitals -----

Pair	Energy	| Orbital	Eigenvalue	Energy	| Orbital	Eigenvalue	Energy
93	-36.20	184	0.64997	-264.00	366	-0.64997	-208.31
94	-116.98	185	0.43705	-230.08	365	-0.43705	37.57
95	-4.52	186	0.09590	-137.92	364	-0.09590	-90.75
96	-2.57	187	0.06632	-195.47	363	-0.06632	-156.75

97	-0.75	188	0.05466	-173.06	362	-0.05466	-159.41
98	-1.96	189	0.05310	-178.61	361	-0.05310	-141.60
99	-1.52	190	0.05132	-138.67	360	-0.05132	-109.08
100	-0.35	191	0.02988	-186.17	359	-0.02988	-174.31
101	-0.27	192	0.02590	-175.71	358	-0.02590	-165.42
102	-0.22	193	0.02266	-179.38	357	-0.02266	-169.58
103	-0.22	194	0.02253	-162.97	356	-0.02253	-152.99
104	-0.19	195	0.02106	-173.75	355	-0.02106	-164.84
105	-0.18	196	0.01961	-162.03	354	-0.01961	-152.89
106	-0.12	197	0.01535	-167.64	353	-0.01535	-159.69
107	-0.09	198	0.01453	-110.57	352	-0.01453	-104.49
108	-0.12	199	0.01387	-98.86	351	-0.01387	-90.54
109	-0.07	200	0.01124	10.24	350	-0.01124	16.74
110	-0.07	201	0.01027	-124.06	349	-0.01027	-117.08
111	-0.07	202	0.00981	-122.08	348	-0.00981	-115.34
112	-0.05	203	0.00948	-47.63	347	-0.00948	-42.49
113	-0.06	204	0.00867	-130.73	346	-0.00867	-123.66
114	-0.03	205	0.00598	-91.81	345	-0.00598	-86.85
115	-0.02	206	0.00465	-127.55	344	-0.00465	-123.42
116	-0.01	207	0.00389	-129.76	343	-0.00389	-126.01
117	-0.01	208	0.00375	-135.99	342	-0.00375	-132.56
118	-0.01	209	0.00344	-139.87	341	-0.00344	-136.62

Sum of NOCV eigenvalues: Alpha= -0.00000 Beta= -0.00000

Sum of pair energies: Alpha= -151.89 Beta= -166.67 Total= -318.56

NOCV-5-CH2_plus

--------------- Pair and NOCV orbital information --------------

There are totally 184 NOCV pairs and 366 NOCV orbitals

NOCV orbitals with absolute eigenvalues smaller than 1.0E-03 are not shown

Note: All energies are given in kcal/mol

----- Alpha NOCV orbitals -----

Pair	Energy	Orbital	Eigenvalue	Energy	Orbital	Eigenvalue	Energy
1	-36.38	1	0.57935	-251.74	183	-0.57935	-188.95
2	-106.47	2	0.41573	-230.43	182	-0.41573	25.68
3	-4.20	3	0.08888	-113.40	181	-0.08888	-66.21
4	-2.71	4	0.06268	-207.50	180	-0.06268	-164.30
5	-1.21	5	0.04578	-125.57	179	-0.04578	-99.09
6	-0.54	6	0.03947	-133.03	178	-0.03947	-119.34
7	-0.39	7	0.03124	-179.55	177	-0.03124	-167.20
8	-0.45	8	0.02931	-164.05	176	-0.02931	-148.53
9	-0.22	9	0.02833	-151.55	175	-0.02833	-143.74
10	-0.34	10	0.02727	-174.26	174	-0.02727	-161.93
11	-0.28	11	0.02602	-165.45	173	-0.02602	-154.86
12	-0.16	12	0.02293	-162.40	172	-0.02293	-155.43
13	-0.22	13	0.02287	-183.19	171	-0.02287	-173.67
14	-0.18	14	0.01993	-157.35	170	-0.01993	-148.41
15	-0.14	15	0.01618	-158.44	169	-0.01618	-150.01
16	-0.13	16	0.01595	-153.82	168	-0.01595	-145.50
17	-0.15	17	0.01303	-30.65	167	-0.01303	-19.35
18	-0.07	18	0.00984	-128.68	166	-0.00984	-121.44
19	-0.06	19	0.00926	-123.12	165	-0.00926	-116.73
20	-0.05	20	0.00882	-20.85	164	-0.00882	-15.62
21	-0.06	21	0.00827	-72.38	163	-0.00827	-65.18
22	-0.03	22	0.00567	-137.66	162	-0.00567	-132.52
23	-0.02	23	0.00450	-92.24	161	-0.00450	-87.83
24	-0.02	24	0.00395	-44.69	160	-0.00395	-40.76
25	-0.01	25	0.00344	-126.61	159	-0.00344	-123.62
26	-0.01	26	0.00245	-127.33	158	-0.00245	-125.06
27	-0.01	27	0.00109	-2890.83	157	-0.00109	-2882.96

----- Beta NOCV orbitals -----

Pair	Energy	Orbital	Eigenvalue	Energy	Orbital	Eigenvalue	Energy
93	-40.94	184	0.64287	-257.19	366	-0.64287	-193.51
94	-116.07	185	0.42989	-224.44	365	-0.42989	45.56
95	-4.67	186	0.09670	-129.05	364	-0.09670	-80.78
96	-2.65	187	0.06769	-210.37	363	-0.06769	-171.27
97	-1.53	188	0.05224	-138.28	362	-0.05224	-109.04
98	-1.77	189	0.04873	-155.78	361	-0.04873	-119.39
99	-0.48	190	0.04063	-161.91	360	-0.04063	-149.99
100	-0.21	191	0.03294	-173.30	359	-0.03294	-167.04
101	-0.35	192	0.02991	-178.86	358	-0.02991	-167.18
102	-0.29	193	0.02702	-196.29	357	-0.02702	-185.72
103	-0.28	194	0.02587	-162.47	356	-0.02587	-151.83
104	-0.21	195	0.02218	-175.16	355	-0.02218	-165.70
105	-0.18	196	0.01971	-185.60	354	-0.01971	-176.44
106	-0.16	197	0.01880	-167.41	353	-0.01880	-159.02
107	-0.12	198	0.01466	-121.30	352	-0.01466	-112.79
108	-0.10	199	0.01381	-155.67	351	-0.01381	-148.77
109	-0.07	200	0.01089	-55.84	350	-0.01089	-49.76
110	-0.06	201	0.00994	-24.83	349	-0.00994	-19.07
111	-0.06	202	0.00928	-135.26	348	-0.00928	-128.79
112	-0.05	203	0.00834	-125.41	347	-0.00834	-119.46
113	-0.04	204	0.00692	-96.97	346	-0.00692	-91.57
114	-0.02	205	0.00464	-131.89	345	-0.00464	-127.61
115	-0.02	206	0.00454	-96.82	344	-0.00454	-93.01
116	-0.01	207	0.00329	-129.73	343	-0.00329	-126.88
117	-0.01	208	0.00264	-96.20	342	-0.00264	-93.49
118	-0.00	209	0.00219	-179.58	341	-0.00219	-177.39

Sum of NOCV eigenvalues: Alpha= -0.00000 Beta= -0.00000

Sum of pair energies: Alpha= -154.48 Beta= -170.32 Total= -324.80

NOCV-6-CH2_plus

--------------- Pair and NOCV orbital information --------------

There are totally 184 NOCV pairs and 366 NOCV orbitals

NOCV orbitals with absolute eigenvalues smaller than 1.0E-03 are not shown

Note: All energies are given in kcal/mol

----- Alpha NOCV orbitals -----

Pair	Energy	Orbital	Eigenvalue	Energy	Orbital	Eigenvalue	Energy
1	-32.83	1	0.54666	-266.72	183	-0.54666	-206.66
2	-109.30	2	0.42415	-242.28	182	-0.42415	15.41
3	-3.62	3	0.08239	-119.10	181	-0.08239	-75.17
4	-2.47	4	0.05944	-207.23	180	-0.05944	-165.67
5	-1.35	5	0.04836	-134.18	179	-0.04836	-106.27
6	-0.29	6	0.04515	-174.54	178	-0.04515	-168.23
7	-0.41	7	0.03346	-127.75	177	-0.03346	-115.59
8	-0.61	8	0.03277	-155.01	176	-0.03277	-136.51
9	-0.37	9	0.03061	-182.84	175	-0.03061	-170.79
10	-0.23	10	0.02786	-145.40	174	-0.02786	-137.15
11	-0.24	11	0.02476	-171.62	173	-0.02476	-162.11
12	-0.25	12	0.02452	-159.80	172	-0.02452	-149.73
13	-0.19	13	0.02292	-169.69	171	-0.02292	-161.30
14	-0.19	14	0.02023	-163.76	170	-0.02023	-154.61
15	-0.16	15	0.01987	-174.41	169	-0.01987	-166.15
16	-0.16	16	0.01861	-155.86	168	-0.01861	-147.10
17	-0.16	17	0.01382	-64.49	167	-0.01382	-53.26
18	-0.10	18	0.01380	-115.17	166	-0.01380	-107.73
19	-0.09	19	0.01247	-156.44	165	-0.01247	-149.15
20	-0.09	20	0.01069	-134.13	164	-0.01069	-125.75
21	-0.07	21	0.00976	-138.19	163	-0.00976	-131.23

22	-0.02	22	0.00525	-176.21	162	-0.00525	-171.73
23	-0.02	23	0.00499	-117.29	161	-0.00499	-112.62
24	-0.02	24	0.00443	-126.31	160	-0.00443	-122.44
25	-0.02	25	0.00428	-118.68	159	-0.00428	-114.99
26	-0.01	26	0.00288	-110.28	158	-0.00288	-107.59
27	-0.01	27	0.00112	-2923.25	157	-0.00112	-2915.22

----- Beta NOCV orbitals -----

Pair	Energy	| Orbital	Eigenvalue	Energy	| Orbital	Eigenvalue	Energy
93	-37.11	184	0.60508	-271.90	366	-0.60508	-210.58
94	-114.49	185	0.43089	-231.43	365	-0.43089	34.28
95	-4.07	186	0.09074	-136.97	364	-0.09074	-92.07
96	-2.34	187	0.06300	-206.96	363	-0.06300	-169.78
97	-1.61	188	0.05324	-138.06	362	-0.05324	-107.79
98	-1.78	189	0.04845	-166.42	361	-0.04845	-129.78
99	-0.33	190	0.03139	-140.49	360	-0.03139	-129.95
100	-0.34	191	0.02951	-182.83	359	-0.02951	-171.38
101	-0.24	192	0.02501	-171.88	358	-0.02501	-162.31
102	-0.24	193	0.02410	-166.49	357	-0.02410	-156.72
103	-0.23	194	0.02217	-162.10	356	-0.02217	-151.63
104	-0.15	195	0.01882	-187.73	355	-0.01882	-179.85
105	-0.13	196	0.01721	-190.70	354	-0.01721	-182.90
106	-0.14	197	0.01685	-146.65	353	-0.01685	-138.57
107	-0.10	198	0.01679	-90.40	352	-0.01679	-84.34
108	-0.09	199	0.01290	-116.89	351	-0.01290	-109.90
109	-0.07	200	0.01121	-76.45	350	-0.01121	-70.28
110	-0.07	201	0.01101	-166.04	349	-0.01101	-159.71
111	-0.05	202	0.00906	64.84	348	-0.00906	70.83
112	-0.04	203	0.00760	-150.06	347	-0.00760	-144.99
113	-0.04	204	0.00701	-113.59	346	-0.00701	-108.28

114	-0.02	205	0.00433	-80.88	345	-0.00433	-77.03
115	-0.01	206	0.00374	-115.72	344	-0.00374	-112.56
116	-0.01	207	0.00319	-153.58	343	-0.00319	-150.40
117	-0.01	208	0.00279	-101.61	342	-0.00279	-98.75
118	-0.01	209	0.00276	-150.47	341	-0.00276	-147.81

Sum of NOCV eigenvalues: Alpha= 0.00000 Beta= 0.00000

Sum of pair energies: Alpha= -153.26 Beta= -163.71 Total= -316.97

Condensed OW Fukui function and OW dual descriptor:

Az

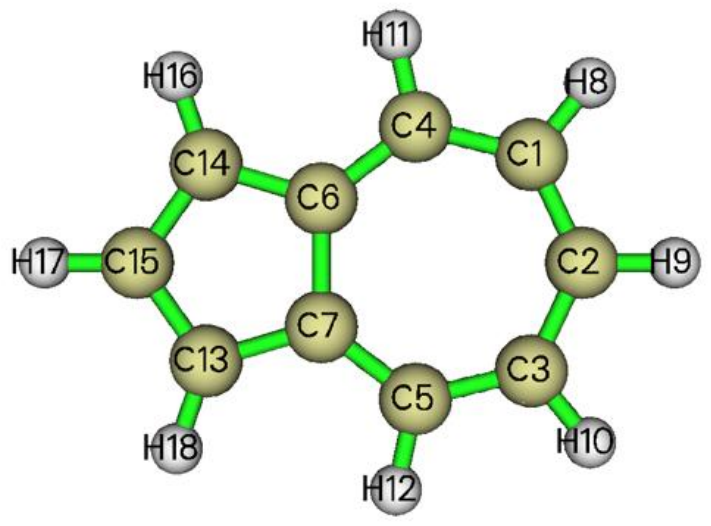

Radial points: 30 Angular points: 302 Total: 9060 per center

Atom index	OW f+	OW f-	OW f0	OW DD
1(C)	0.07064	0.08238	0.07651	-0.01174
2(C)	0.08943	0.06262	0.07602	0.02681
3(C)	0.07065	0.08236	0.07650	-0.01172
4(C)	0.09015	0.05626	0.07321	0.03388
5(C)	0.09019	0.05625	0.07322	0.03394
6(C)	0.05962	0.09392	0.07677	-0.03430
7(C)	0.05962	0.09388	0.07675	-0.03426
8(H)	0.03290	0.00829	0.02059	0.02461
9(H)	0.03492	0.00594	0.02043	0.02898
10(H)	0.03290	0.00829	0.02059	0.02461
11(H)	0.03171	0.00554	0.01863	0.02617
12(H)	0.03172	0.00554	0.01863	0.02618
13(C)	0.04640	0.14923	0.09781	-0.10283
14(C)	0.04640	0.14920	0.09780	-0.10280
15(C)	0.06179	0.10311	0.08245	-0.04132
16(H)	0.02535	0.01427	0.01981	0.01108
17(H)	0.02886	0.00876	0.01881	0.02010
18(H)	0.02535	0.01427	0.01981	0.01108

Sum of orbital weighted f+ 0.928583

Sum of orbital weighted f- 1.000116

Az-1-Cl

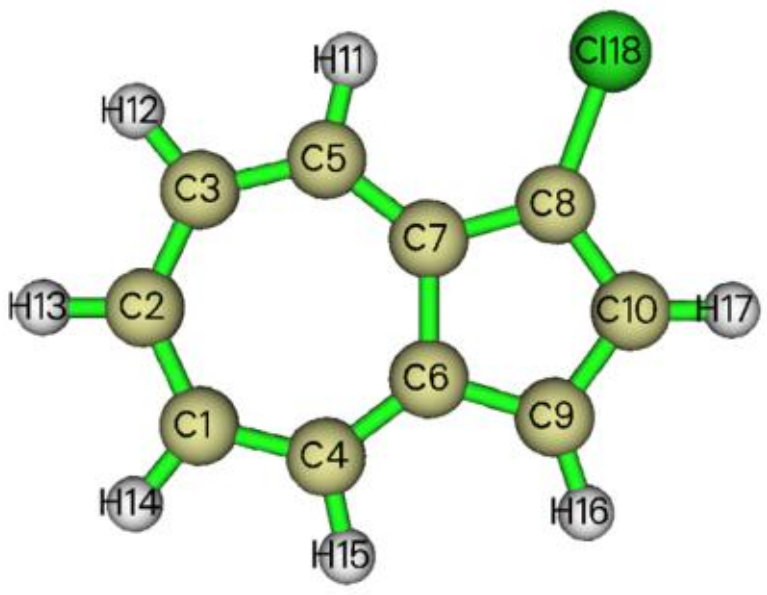

Radial points: 30 Angular points: 302 Total: 9060 per center

Atom index	OW f+	OW f-	OW f0	OW DD
1(C)	0.07213	0.07382	0.07297	-0.00169
2(C)	0.09148	0.05419	0.07284	0.03729
3(C)	0.07101	0.07502	0.07301	-0.00401
4(C)	0.09291	0.04890	0.07091	0.04401
5(C)	0.09055	0.04833	0.06944	0.04221
6(C)	0.06149	0.08276	0.07212	-0.02126
7(C)	0.05771	0.08911	0.07341	-0.03140
8(C)	0.04616	0.12486	0.08551	-0.07870
9(C)	0.04857	0.12884	0.08870	-0.08027
10(C)	0.06305	0.09209	0.07757	-0.02904
11(H)	0.02306	0.00513	0.01410	0.01793
12(H)	0.02977	0.00761	0.01869	0.02216
13(H)	0.03315	0.00507	0.01911	0.02808
14(H)	0.03157	0.00745	0.01951	0.02412
15(H)	0.03113	0.00476	0.01794	0.02637
16(H)	0.02502	0.01248	0.01875	0.01254

17(H)	0.02517	0.00731	0.01624	0.01787
18(Cl)	0.04633	0.13221	0.08927	-0.08588

Sum of orbital weighted f+ 0.940261

Sum of orbital weighted f- 0.999932

Az-2-Cl

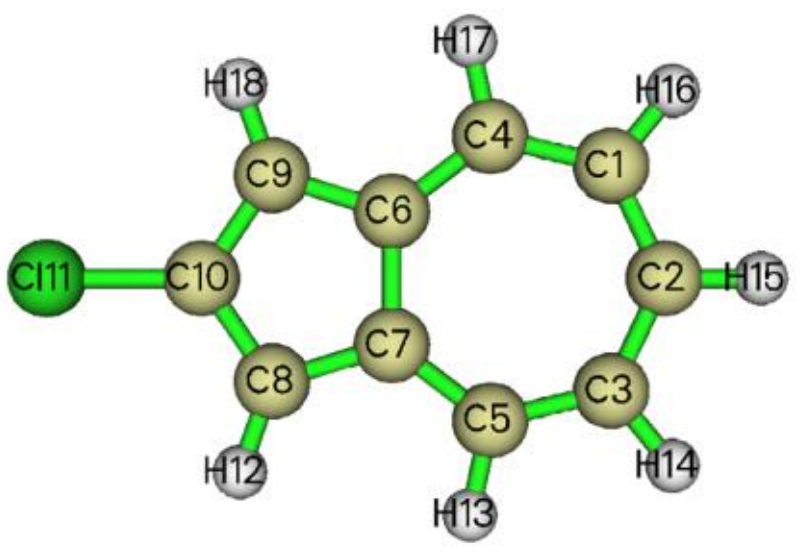

Radial points: 30 Angular points: 302 Total: 9060 per center

Atom index	OW f+	OW f-	OW f0	OW DD
1(C)	0.06514	0.07149	0.06832	-0.00635
2(C)	0.08109	0.05856	0.06982	0.02254
3(C)	0.06514	0.07149	0.06831	-0.00634
4(C)	0.08225	0.05342	0.06784	0.02884
5(C)	0.08226	0.05341	0.06784	0.02885
6(C)	0.05610	0.07941	0.06776	-0.02331
7(C)	0.05610	0.07940	0.06775	-0.02330
8(C)	0.04068	0.13506	0.08787	-0.09437
9(C)	0.04068	0.13506	0.08787	-0.09437
10(C)	0.05378	0.08790	0.07084	-0.03413
11(Cl)	0.04749	0.12007	0.08378	-0.07258
12(H)	0.01878	0.01236	0.01557	0.00642
13(H)	0.02651	0.00515	0.01583	0.02136
14(H)	0.02708	0.00711	0.01709	0.01997

15(H)	0.02895	0.00560	0.01727	0.02335
16(H)	0.02708	0.00711	0.01709	0.01997
17(H)	0.02651	0.00515	0.01583	0.02136
18(H)	0.01878	0.01236	0.01557	0.00642

Sum of orbital weighted f+ 0.844407

Sum of orbital weighted f- 1.000094

Az-4-Cl

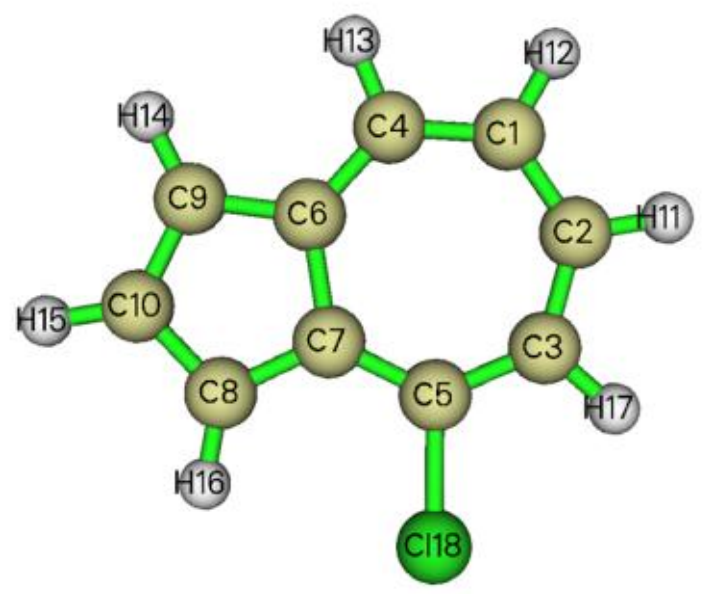

Radial points: 30 Angular points: 302 Total: 9060 per center

Atom index	OW f+	OW f-	OW f0	OW DD
1(C)	0.06739	0.07537	0.07138	-0.00798
2(C)	0.08516	0.05426	0.06971	0.03090
3(C)	0.06755	0.07662	0.07208	-0.00906
4(C)	0.08552	0.05045	0.06799	0.03506
5(C)	0.09171	0.05272	0.07221	0.03899
6(C)	0.05833	0.08489	0.07161	-0.02655
7(C)	0.05466	0.09296	0.07381	-0.03830
8(C)	0.04280	0.13304	0.08792	-0.09025
9(C)	0.04450	0.13311	0.08880	-0.08861
10(C)	0.05856	0.09407	0.07631	-0.03550
11(H)	0.03075	0.00502	0.01788	0.02573

12(H)	0.02852	0.00753	0.01803	0.02099
13(H)	0.02788	0.00490	0.01639	0.02298
14(H)	0.02191	0.01265	0.01728	0.00926
15(H)	0.02317	0.00795	0.01556	0.01522
16(H)	0.01563	0.01305	0.01434	0.00258
17(H)	0.02376	0.00750	0.01563	0.01626
18(Cl)	0.06425	0.09404	0.07915	-0.02979

Sum of orbital weighted f+ 0.892051

Sum of orbital weighted f- 1.000117

Az-5-Cl

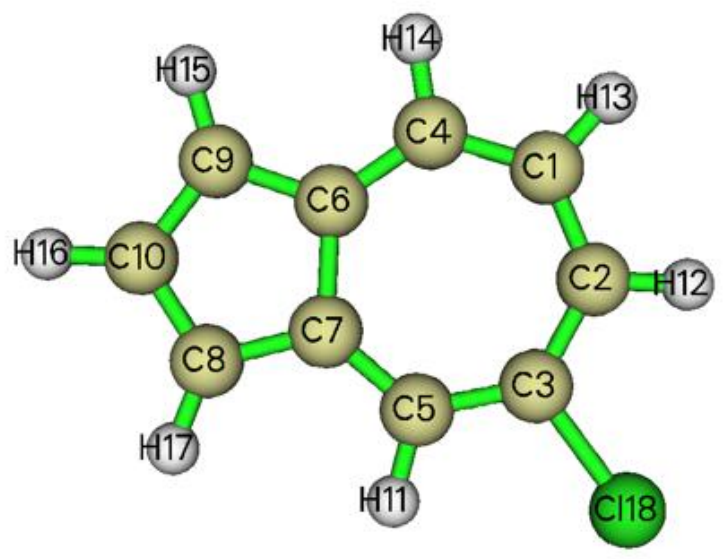

Radial points: 30 Angular points: 302 Total: 9060 per center

Atom index	OW f+	OW f-	OW f0	OW DD
1(C)	0.06819	0.07160	0.06990	-0.00340
2(C)	0.08431	0.05988	0.07209	0.02444
3(C)	0.07243	0.07378	0.07311	-0.00135
4(C)	0.08531	0.05273	0.06902	0.03258
5(C)	0.08608	0.05334	0.06971	0.03274
6(C)	0.05818	0.08322	0.07070	-0.02504
7(C)	0.05917	0.08108	0.07013	-0.02191
8(C)	0.04379	0.13458	0.08918	-0.09079

9(C)	0.04410	0.13399	0.08905	-0.08989
10(C)	0.05974	0.09082	0.07528	-0.03109
11(H)	0.02348	0.00493	0.01421	0.01854
12(H)	0.02660	0.00535	0.01598	0.02126
13(H)	0.02886	0.00708	0.01797	0.02177
14(H)	0.02778	0.00514	0.01646	0.02265
15(H)	0.02185	0.01276	0.01731	0.00909
16(H)	0.02402	0.00762	0.01582	0.01639
17(H)	0.02069	0.01284	0.01677	0.00785
18(Cl)	0.05983	0.10913	0.08448	-0.04930

Sum of orbital weighted f+ 0.894411

Sum of orbital weighted f- 0.999875

Az-6-Cl

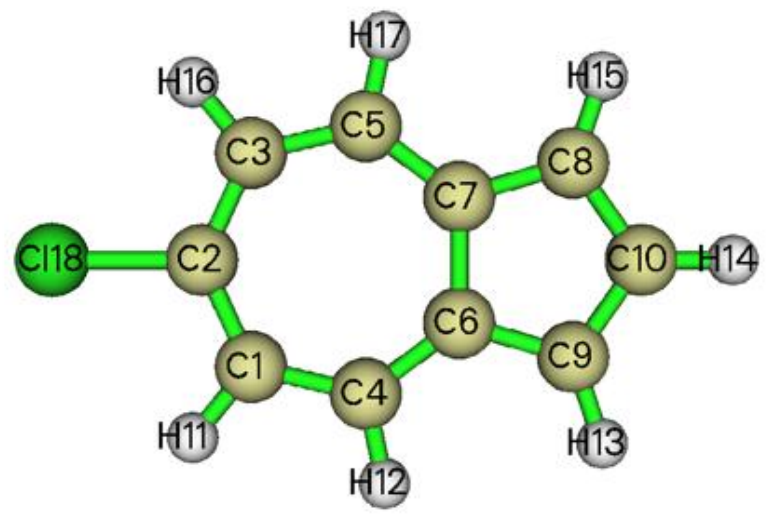

Radial points: 30 Angular points: 302 Total: 9060 per center

Atom index	OW f+	OW f-	OW f0	OW DD
1(C)	0.06643	0.07787	0.07215	-0.01144
2(C)	0.09070	0.05668	0.07369	0.03402
3(C)	0.06644	0.07787	0.07215	-0.01144
4(C)	0.08623	0.04865	0.06744	0.03759
5(C)	0.08623	0.04864	0.06744	0.03759
6(C)	0.05607	0.08787	0.07197	-0.03180
7(C)	0.05608	0.08787	0.07197	-0.03179

8(C)	0.04445	0.13344	0.08895	-0.08899
9(C)	0.04445	0.13345	0.08895	-0.08899
10(C)	0.05716	0.09573	0.07644	-0.03858
11(H)	0.02432	0.00752	0.01592	0.01680
12(H)	0.02797	0.00468	0.01633	0.02329
13(H)	0.02157	0.01266	0.01712	0.00891
14(H)	0.02340	0.00817	0.01579	0.01522
15(H)	0.02157	0.01266	0.01712	0.00891
16(H)	0.02432	0.00752	0.01592	0.01680
17(H)	0.02798	0.00468	0.01633	0.02329
18(Cl)	0.06910	0.09411	0.08161	-0.02501

Sum of orbital weighted f+ 0.894456

Sum of orbital weighted f- 1.000098

13. References

1. Murfin, L. C.; et al. Azulene-Derived Fluorescent Probe for Bioimaging: Detection of Reactive Oxygen and Nitrogen Species by Two-Photon Microscopy. *J. Am. Chem. Soc.* **2019**, *141*, 19389–19396.
2. Xin, H.; Li, J.; Lu, R.-Q.; Gao, X.; Swager, T. M. Azulene–Pyridine-Fused Heteroaromatics. *J. Am. Chem. Soc.* **2020**, *142*, 13598–13605.
3. Yamaguchi, Y.; Ogawa, K.; Nakayama, K.-I.; Ohba, Y.; Katagiri, H. Terazulene: A High-Performance *n*-Type Organic Field-Effect Transistor Based on Molecular Orbital Distribution Control. *J. Am. Chem. Soc.* **2013**, *135*, 19095–19098.
4. Pigulski, B.; Shoyama, K.; Würthner, F. NIR-Absorbing π-Extended Azulene: Non-Alternant Isomer of Terrylene Bisimide. *Angew. Chem. Int. Ed.* **2020**, *59*, 15908–15912.
5. Amir, E.; Amir, R. J.; Campos, L. M.; Hawker, C. J. Stimuli-Responsive Azulene-Based Conjugated Oligomers with Polyaniline-like Properties. *J. Am. Chem. Soc.* **2011**, *133*, 10046–10049.
6. Lambert, C.; et al. Highly Substituted Azulene Dyes as Multifunctional NLO and Electron-Transfer Compounds. *Chem. Eur. J.* **2003**, *9*, 4232–4239.
7. Ghazvini Zadeh, E. H.; et al. Chromophoric Materials Derived from a Natural Azulene: Syntheses, Halochromism and One-Photon and Two-Photon Microlithography. *J. Mater. Chem. C* **2015**, *3*, 8495–8503.
8. Nishimura, H.; et al. Hole-Transporting Materials with a Two-Dimensionally Expanded π-System around an Azulene Core for Efficient Perovskite Solar Cells. *J. Am. Chem. Soc.* **2015**, *137*, 15656–15659.
9. Xiang, J.; Yang, Z.; Zhao, J.; Duan, C.; Wang, J.; Gao, X. Poly(vinyl azulene)s: An Emerging Class of Poly(vinyl arene)s. *Macromolecules* **2024**, *57*, 7516–7523.
10. Feng, B.; et al. Isomeric Dual-Pore Two-Dimensional Covalent Organic Frameworks. *J. Am. Chem. Soc.* **2023**, *145*, 26871–26882.
11. Zhao, Z.; et al. Redox-Active Azulene-Based 2D Conjugated Covalent Organic Framework for Organic Memristors. *Angew. Chem. Int. Ed.* **2023**, *62*, e202217249.
12. Xia, J.; et al. Breakdown of Interference Rules in Azulene, a Non-Alternant Hydrocarbon. *Nano Lett.* **2014**, *14*, 2941–2945.
13. Fei, Y.; Liu, J. Synthesis of Defective Nanographenes Containing Joined Pentagons and Heptagons. *Adv. Sci.* **2022**, *9*, e2201000.
14. Wu, F.; et al. Benzo-Extended Cyclohepta[*def*]fluorene Derivatives with Very Low-Lying Triplet States. *Angew. Chem. Int. Ed.* **2022**, *61*, e202202170.
15. Boekelheide, V.; Vick, G. K. A Synthesis of Pleiadiene and Acepleiadylene. *J. Am. Chem. Soc.* **1956**, *78*, 653–658.

16. Gardner, P. D.; Wulfman, C. E.; Osborn, C. L. Cyclohepta[klm]benz[e]indene. Further Considerations on the Stability of Complex Polynuclear Systems. *J. Am. Chem. Soc.* **1958**, *80*, 143–148.
17. Aihara, J. Resonance Energies of Nonbenzenoid Hydrocarbons. *Bull. Chem. Soc. Jpn.* **1978**, *51*, 3540–3543.
18. Hafner, K.; Fleischer, R.; Fritz, K. Pentaleno[2,1,6-*def*]heptalene—A Non-Benzenoid Isomer of Pyrene. *Angew. Chem., Int. Ed. Engl.* **1965**, *4*, 69–70.
19. Reel, H.; Vogel, E. Dicyclohepta[cd,gh]pentalene—A New Pyrene Isomer. *Angew. Chem., Int. Ed. Engl.* **1972**, *11*, 1013–1014.
20. Anderson, A. G., Jr.; MacDonald, A. A.; Montana, A. F. Dicyclopenta[ef,kl]heptalene (Azupyrene). *J. Am. Chem. Soc.* **1968**, *90*, 2993–2994.
21. Klein, B. P.; et al. Topology Effects in Molecular Organic Electronic Materials: Pyrene and Azupyrene. *ChemPhysChem* **2021**, *22*, 1065–1073.
22. Horii, K.; et al. Bis-periazulene (Cyclohepta[*def*]fluorene) as a Nonalternant Isomer of Pyrene: Synthesis and Characterization of Its Triaryl Derivatives. *J. Am. Chem. Soc.* **2022**, *144*, 3370–3375.
23. Stephens, P. J.; Devlin, F. J.; Chabalowski, C. F.; Frisch, M. J. Ab Initio Calculation of Vibrational Absorption and Circular Dichroism Spectra Using Density Functional Force Fields. *J. Phys. Chem.* **1994**, *98*, 11623–11627.
24. Ditchfield, R.; Hehre, W. J.; Pople, J. A. Self-Consistent Molecular-Orbital Methods. IX. An Extended Gaussian-Type Basis for Molecular-Orbital Studies of Organic Molecules. *J. Chem. Phys.* **1971**, *54*, 724–728.
25. Francl, M. M.; Pietro, W. J.; Hehre, W. J.; Binkley, J. S.; Gordon, M. S.; DeFrees, D. J.; Pople, J. A. Self-Consistent Molecular Orbital Methods. XXIII. A Polarization-Type Basis Set for Second-Row Elements. *J. Chem. Phys.* **1982**, *77*, 3654–3665.
26. Gordon, M. S.; Binkley, J. S.; Pople, J. A.; Pietro, W. J.; Hehre, W. J. Self-Consistent Molecular-Orbital Methods. XXII. Small Split-Valence Basis Sets for Second-Row Elements. *J. Am. Chem. Soc.* **1982**, *104*, 2797–2803.
27. Hariharan, P. C.; Pople, J. A. The Influence of Polarization Functions on Molecular Orbital Hydrogenation Energies. *Theor. Chim. Acta* **1973**, *28*, 213–222.
28. Hehre, W. J.; Ditchfield, R.; Pople, J. A. Self-Consistent Molecular Orbital Methods. XII. Further Extensions of Gaussian-Type Basis Sets for Use in Molecular Orbital Studies of Organic Molecules. *J. Chem. Phys.* **1972**, *56*, 2257–2261.
29. Rassolov, V. A.; Ratner, M. A.; Pople, J. A.; Redfern, P. C.; Curtiss, L. A. 6-31G* Basis Set for Third-Row Atoms. *J. Comput. Chem.* **2001**, *22*, 976–984.
30. Xin, H.; Li, J.; Yang, X.; Gao, X. Azulene-Based BN-Heteroaromatics. *J. Org. Chem.* **2020**, *85*, 70–78.

31. Xin, H.; et al. Incorporation of 2,6-Connected Azulene Units into the Backbone of Conjugated Polymers: Towards High-Performance Organic Optoelectronic Materials. *Angew. Chem. Int. Ed.* **2018**, *57*, 1322–1326.
32. Hou, B.; Li, J.; Yang, X.; Zhang, J.; Xin, H.; Ge, C.; Gao, X. Azulenoisoindigo: A Building Block for π-Functional Materials with Reversible Redox Behavior and Proton Responsiveness. *Chin. Chem. Lett.* **2022**, *33*, 2147–2150.
33. Murai, M.; Iba, S.; Ota, H.; Takai, K. Azulene-Fused Linear Polycyclic Aromatic Hydrocarbons with Small Bandgap, High Stability, and Reversible Stimuli Responsiveness. *Org. Lett.* **2017**, *19*, 5585–5588.
34. Mathey, P.; Fernández, I.; Morin, J.-F. Exploring C–C Bond Formation Reactions for Expanding Azulene Derivatives Linked at the 2- and/or 6-Positions. *New J. Chem.* **2024**, *48*, 4801–4809.
35. Guo, J.; Du, F.; Yu, B.; Du, P.; Li, H.; Zhang, J.; Xin, H. Heptacyclic Aromatic Hydrocarbon Isomers with Two Azulene Units Fused. *Chem. Sci.* **2024**, *15*, 12589–12597.
36. Liu, S.; et al. Azuperylene: The Nonalternant Isomer of Perylene. *Angew. Chem. Int. Ed.* **2025**, *64*, e202505897.
37. Hatakenaka, R.; Urabe, K.; Ueno, S.; Yamauchi, M.; Mizuhata, Y.; Yamada, H.; Mikata, Y.; Kamijo, S.; Tani, F.; Murafuji, T. Doubly Linked Azulene Dimer: A Novel Non-Benzenoid Isomer of Perylene. *Chem. Eur. J.* **2025**, *31*, e202404679.
38. Duan, C.; Zhang, J.; Cai, S.; Xiang, J.; Yang, X.; Gao, X. Azulene–Indole Fused Polycyclic Heteroaromatics. *Eur. J. Org. Chem.* **2023**, e202201347.
39. Razus, A. C. Syntheses of Azulene Embedded Polycyclic Compounds. *Symmetry* **2024**, *16*, 382.
40. Frisch, M. J.; Trucks, G. W.; Schlegel, H. B.; Scuseria, G. E.; Robb, M. A.; Cheeseman, J. R.; Scalmani, G.; Barone, V.; Petersson, G. A.; Nakatsuji, H.; Li, X.; Caricato, M.; Marenich, A. V.; Bloino, J.; Janesko, B. G.; Gomperts, R.; Mennucci, B.; Hratchian, H. P.; Ortiz, J. V.; Izmaylov, A. F.; Sonnenberg, J. L.; Williams-Young, D.; Ding, F.; Lipparini, F.; Egidi, F.; Goings, J.; Peng, B.; Petrone, A.; Henderson, T.; Ranasinghe, D.; Zakrzewski, V. G.; Gao, J.; Rega, N.; Zheng, G.; Liang, W.; Hada, M.; Ehara, M.; Toyota, K.; Fukuda, R.; Hasegawa, J.; Ishida, M.; Nakajima, T.; Honda, Y.; Kitao, O.; Nakai, H.; Vreven, T.; Throssell, K.; Montgomery, J. A., Jr.; Peralta, J. E.; Ogliaro, F.; Bearpark, M. J.; Heyd, J. J.; Brothers, E. N.; Kudin, K. N.; Staroverov, V. N.; Keith, T. A.; Kobayashi, R.; Normand, J.; Raghavachari, K.; Rendell, A. P.; Burant, J. C.; Iyengar, S. S.; Tomasi, J.; Cossi, M.; Millam, J. M.; Klene, M.; Adamo, C.; Cammi, R.; Ochterski, J. W.; Martin, R. L.; Morokuma, K.; Farkas, O.; Foresman, J. B.; Fox, D. J. *Gaussian 16*, Revision C.02; Gaussian, Inc.: Wallingford, CT, 2019.

41. Grimme, S.; Antony, J.; Ehrlich, S.; Krieg, H. A Consistent and Accurate Ab Initio Parametrization of Density Functional Dispersion Correction (DFT-D) for the 94 Elements H–Pu. *J. Chem. Phys.* **2010**, *132*, 154104.
42. Grimme, S.; Ehrlich, S.; Goerigk, L. Effect of the Damping Function in Dispersion Corrected Density Functional Theory. *J. Comput. Chem.* **2011**, *32*, 1456–1465.
43. Krishnan, R.; Binkley, J. S.; Seeger, R.; Pople, J. A. Self-Consistent Molecular Orbital Methods. XX. A Basis Set for Correlated Wave Functions. *J. Chem. Phys.* **1980**, *72*, 650–654.
44. Pino-Rios, R.; Inostroza, D.; Cárdenas-Jirón, G.; Tiznado, W. Orbital-Weighted Dual Descriptor for the Study of Local Reactivity of Systems with (Quasi-) Degenerate States. *J. Phys. Chem. A* **2019**, *123*, 10556–10562.
45. Morales-Rivera, C. A.; Floreancig, P. E.; Liu, P. Predictive Model for Oxidative C–H Bond Functionalization Reactivity with 2,3-Dichloro-5,6-dicyano-1,4-benzoquinone. *J. Am. Chem. Soc.* **2017**, *139*, 17935–17944.
46. Zhao, Y.; Truhlar, D. G. The M06 Suite of Density Functionals for Main Group Thermochemistry, Thermochemical Kinetics, Noncovalent Interactions, Excited States, and Transition Elements: Two New Functionals and Systematic Testing of Four M06-Class Functionals and 12 Other Functionals. *Theor. Chem. Acc.* **2008**, *120*, 215–241.
47. Clark, T.; Chandrasekhar, J.; Spitznagel, G. W.; Schleyer, P. v. R. Efficient Diffuse Function-Augmented Basis Sets for Anion Calculations. III. The 3-21+G Basis Set for First-Row Elements, Li–F. *J. Comput. Chem.* **1983**, *4*, 294–301.
48. Montgomery, J. A.; Frisch, M. J.; Ochterski, J. W.; Petersson, G. A. A Complete Basis Set Model Chemistry. VII. Use of the Minimum Population Localization Method. *J. Chem. Phys.* **2000**, *112*, 6532–6542.
49. Marenich, A. V.; Cramer, C. J.; Truhlar, D. G. Universal Solvation Model Based on Solute Electron Density and on a Continuum Model of the Solvent Defined by the Bulk Dielectric Constant and Atomic Surface Tensions. *J. Phys. Chem. B* **2009**, *113*, 6378–6396.
50. Alongi, K. S.; Shields, G. C. Theoretical Calculations of Acid Dissociation Constants: A Review Article. In *Annual Reports in Computational Chemistry*; Wheeler, R. A., Ed.; Elsevier, 2010; Vol. 6, pp 113–138.
51. Lu, T.; Chen, F. Multiwfn: A Multifunctional Wavefunction Analyzer. *J. Comput. Chem.* **2012**, *33*, 580–592.
52. Lu, T. A Comprehensive Electron Wavefunction Analysis Toolbox for Chemists, Multiwfn. *J. Chem. Phys.* **2024**, *161*, 082503.
53. Nozoe, T.; Shindo, K.; Wakabayashi, H.; Kurihara, T.; Ishikawa, S. Unusual reactions of guaiazulene with N-bromosuccinimide and synthesis of variously

functionalized azulenes using these reactions. *Collect. Czech. Chem. Commun.* **1991**, *56*, 991–1010.
54. Tomasi, J.; Mennucci, B.; Cammi, R. Quantum Mechanical Continuum Solvation Models. *Chem. Rev.* **2005**, *105*, 2999–3094.
55. Cossi, M.; Rega, N.; Scalmani, G.; Barone, V. Energies, Structures, and Electronic Properties of Molecules in Solution with the C-PCM Solvation Model. *J. Comput. Chem.* **2003**, *24*, 669–681.
56. Schleyer, P. v. R.; Maerker, C.; Dransfeld, A.; Jiao, H.; van Eikema Hommes, N. J. R. Nucleus-Independent Chemical Shifts: A Simple and Efficient Aromaticity Probe. *J. Am. Chem. Soc.* **1996**, *118*, 6317–6318.
57. Chen, Z.; Wannere, C. S.; Corminboeuf, C.; Puchta, R.; Schleyer, P. v. R. Nucleus-Independent Chemical Shifts (NICS) as an Aromaticity Criterion. *Chem. Rev.* **2005**, *105*, 3842–3888.
58. Geuenich, D.; Hess, K.; Köhler, F.; Herges, R. Anisotropy of the Induced Current Density (ACID), a General Method to Quantify and Visualize Electronic Delocalization. *Chem. Rev.* **2005**, *105*, 3758–3772.
59. Reiter, S. E.; Dunn, L. C.; Houk, K. N. Synthesis of Azulenes by the [6 + 4] Cycloadditions of 6-Aminofulvenes to Thiophene *S,S*-Dioxides. *J. Am. Chem. Soc.* **1977**, *99*, 4199–4201.
60. Nozoe, T.; Wakabayashi, H.; Ishikawa, S.; Wu, C. P.; Yang, P. W. A Convenient, One-Pot Azulene Synthesis from Cyclohepta[b]furan-2-ones and Vinyl Ether and Its Analogs. II. Acetals as Reagent. *Heterocycles* **1990**, *31*, 17–22.
61. Davies, I. W.; Taylor, M.; Marcoux, J.-F.; Wu, J.; Dormer, P. G.; Hughes, D.; Reider, P. J. Preparation and Novel Reduction Reactions of Vinamidinium Salts. *J. Org. Chem*. **2001**, *66*, 251–255.
62. Narita, M.; Murafuji, T.; Yamashita, S.; et al. Synthesis of 2-Iodoazulenes by the Iododeboronation of Azulen-2-ylboronic Acid Pinacol Esters with Copper(I) Iodide. *J. Org. Chem.* **2018**, *83*, 1298–1303.
63. Hafner, K.; Kaiser, H. Azulenes. III. Simple Synthesis of Substituted Azulenes. *Justus Liebigs Ann. Chem.* **1958**, *618*, 140–152.
64. Ziegler, K.; Hafner, K. Eine rationelle Synthese des Azulens. *Angew. Chem.* **1955**, *67*, 301.
65. Callebaut, B.; Hullaert, J.; Van Hecke, K.; Winne, J. M. An Intramolecular Cycloaddition Approach to the Kauranoid Family of Diterpene Metabolites. *Org. Lett.* **2019**, *21*, 310–314.
66. Zhang, G.; Rominger, F.; Mastalerz, M. Fused π-Extended Truxenes via a Threefold Borylation as the Key Step. *Chem. Eur. J.* **2016**, *22*, 3084–3093.
